# Mechanistic basis of post-treatment control of SIV after anti-α4β7 antibody therapy

**DOI:** 10.1371/journal.pcbi.1009031

**Published:** 2021-06-09

**Authors:** Chad R. Wells, Youfang Cao, David P. Durham, Siddappa N. Byrareddy, Aftab A. Ansari, Nancy H. Ruddle, Jeffrey P. Townsend, Alison P. Galvani, Alan S. Perelson

**Affiliations:** 1 Center for Infectious Disease Modeling and Analysis, Yale School of Public Health, New Haven, Connecticut, United States of America; 2 Theoretical Biology and Biophysics Group, Los Alamos National Laboratory, Los Alamos, New Mexico, United States of America; 3 Department of Pharmacology and Experimental Neuroscience, University of Nebraska Medical Center, Omaha, Nebraska, United States of America; 4 Department of Pathology and Laboratory Medicine, Emory University School of Medicine, Atlanta, Georgia, United States of America; 5 Epidemiology of Microbial Diseases, Yale School of Public Health, New Haven, Connecticut, United States of America; 6 Department of Biostatistics, Yale University, New Haven, Connecticut, United States of America; 7 Program in Computational Biology and Bioinformatics, Yale University, New Haven, Connecticut, United States of America; ETH Zurich, SWITZERLAND

## Abstract

Treating macaques with an anti-α4β7 antibody under the umbrella of combination antiretroviral therapy (cART) during early SIV infection can lead to viral remission, with viral loads maintained at < 50 SIV RNA copies/ml after removal of all treatment in a subset of animals. Depletion of CD8^+^ lymphocytes in controllers resulted in transient recrudescence of viremia, suggesting that the combination of cART and anti-α4β7 antibody treatment led to a state where ongoing immune responses kept the virus undetectable in the absence of treatment. A previous mathematical model of HIV infection and cART incorporates immune effector cell responses and exhibits the property of two different viral load set-points. While the lower set-point could correspond to the attainment of long-term viral remission, attaining the higher set-point may be the result of viral rebound. Here we expand that model to include possible mechanisms of action of an anti-α4β7 antibody operating in these treated animals. We show that the model can fit the longitudinal viral load data from both IgG control and anti-α4β7 antibody treated macaques, suggesting explanations for the viral control associated with cART and an anti-α4β7 antibody treatment. This effective perturbation to the virus-host interaction can also explain observations in other nonhuman primate experiments in which cART and immunotherapy have led to post-treatment control or resetting of the viral load set-point. Interestingly, because the viral kinetics in the various treated animals differed—some animals exhibited large fluctuations in viral load after cART cessation—the model suggests that anti-α4β7 treatment could act by different primary mechanisms in different animals and still lead to post-treatment viral control. This outcome is nonetheless in accordance with a model with two stable viral load set-points, in which therapy can perturb the system from one set-point to a lower one through different biological mechanisms.

## Author summary

Some macaques treated with an anti-α4β7 monoclonal antibody along with antiretroviral therapy during the early stages of simian immunodeficiency virus infection had their viral load become undetectable (below 50 SIV RNA copies/ml) after all treatment was stopped, whereas animals not given the antibody all had their viral loads rebound to high levels. Using a mathematical model, we examined four potential ways in which the antibody could have altered the balance between viral growth and immune control to maintain an undetectable viral load. We show that a shift to controlled infection can occur through multiple biologically reasonable mechanisms of action of the anti-α4β7 antibody.

## Introduction

Combination antiretroviral therapy (cART) can effectively suppress the viremia in people living with HIV. However, successful viral suppression requires life-long treatment, and no cure for HIV infection is currently available. The integrin α4β7 antibody has become a target for the development of a novel HIV therapy [1–4], as its expression at the cell surface increases the susceptibility of CD4^+^ T cells to HIV infection [5,6]. Intravenous infusion of an anti-α4β7 antibody can decrease the viral load both in plasma and in the gastrointestinal tract during primary simian immunodeficiency virus (SIV) infection [3,4], while prophylactic administration can substantially reduce the risk of SIV infection [3]. Treating SIV *nef-stop* infected macaques with a 90-day course of cART initiated at five weeks post-infection (p.i.) supplemented by the intravenous administration of a primatized monoclonal anti-α4β7 antibody started at nine weeks p.i. has been shown to result in plasma viral loads ultimately being maintained below 50 RNA copies/ml for more than nine months following the cessation of all treatment [2]. This sustained effect has raised the prospect of using anti-α4β7 antibodies as an adjunct to HIV preventive therapy and treatment.

**Fig 1:**
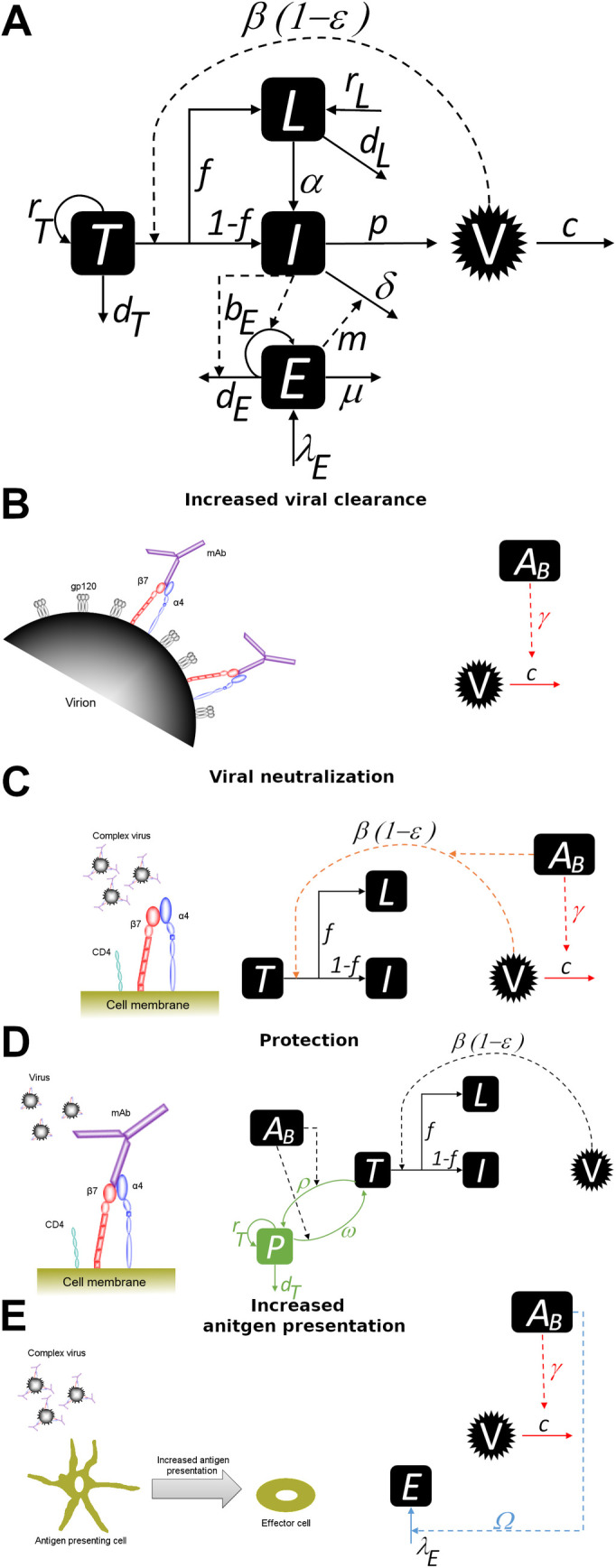
A visualization of the four different mechanisms of action of the anti-*α*4*β*7 antibody and corresponding model diagram. A) **Model schematic in the absence of the anti-***α***4***β***7 antibody.** Target cells (*T*) proliferate logistically at rate *r*_*T*_, die at rate *d*_*T*_ and become infected at rate (1- *ε*)*V*, where *β* is the transmission rate, *ε* is the efficacy of cART, and *V* is the viral concentration. Once target cells are infected, a fraction *f* become latent cells (*L*) and the remaining become productively infected cells (*I*). Latent cells proliferate at rate *r*_*L*_, die at rate *d*_*L*_, and activate at rate *α*. Infected cells produce virus at rate *p*, die due to viral cytopathic effects at rate *δ*, and are killed at rate *mE* by effector cells (*E*). Effector cells are produced at rate *λ*_*E*_, proliferate at a maximum rate *b*_*E*_, become exhausted at a maximum rate *d*_*E*_, and die at rate μ. Virus is cleared in the absence of the anti-*α*4*β*7 antibody at rate c. B) **Increased viral clearance.** The anti-*α*4*β*7 monoclonal antibody (purple) (*A*_*B*_) binds to *α*4*β*7 integrin (red and blue) expressed on the viral membrane and opsonizes the virus, increasing the clearance rate *γ*-fold or less depending on the *A*_*B*_ concentration. C) **Viral neutralization.** The anti-*α*4*β*7 monoclonal antibody (purple) binds to the *α*4*β*7 integrin (red and blue) expressed on the viral membrane, causing increased viral clearance and viral neutralization, i.e. inhibition of infection, while also contributing to increased viral clearance. D) **Protection from infection.** The anti-*α*4*β*7 monoclonal antibody binds to *α*4*β*7 integrin expressed on the surface of uninfected CD4+ T cells and protects them from infection. Target cells enter a protected state (*P*) at a maximum rate *ρ*, with protection waning at a maximum rate ⍵. E) **Increased antigen presentation.** Antibody-virus complexes are picked up by antigen presenting cells resulting in increased antigen presentation. We assume that this increased antigen presentation increases the effector cell source rate dependent on the *A*_*B*_ concentration and the parameter Ω. The formation of antibody-virus complexes may also contribute to increased viral clearance.

Here, we provide a potential explanation for how the addition of a monoclonal anti-α4β7 antibody to cART could lead to long-term viral control after treatment cessation. A follow-up study on a subset of the eight controller macaques from Byrareddy et al. [2] showed that SIV was still present in those animals as their virus transiently rebounded when CD8^+^ lymphocytes were depleted (S2 Text). This transient rebound in viremia suggests that long-term control was immune-mediated. Conway and Perelson [7] developed a viral kinetic model containing an effector cell response to explain the observation of post-treatment control in the VISCONTI study, where 14 HIV-infected individuals who started cART during primary infection were able to control virus levels to low or undetectable levels for years following cessation of treatment [8]. Here, we expand this model by including potential mechanisms by which anti-α4β7 antibodies may act. The mechanisms that we consider are described below and in Fig 1.

The α4β7 integrin is incorporated into the membrane of viruses produced by SIV-infected α4β7^+^ T cells and is functionally active [9]. It has been hypothesized that the anti-α4β7antibody can bind the α4β7 integrin and lead to increased viral opsonization and *increased viral clearance* [9] (Fig 1B) and possibly *virus neutralization* (Fig 1C).

Blocking of SIV infection might occur when the anti-α4β7 antibody binds to α4β7^+^ CD4^+^ T cells [4,10] (Fig 1D). We define this mechanism as *protection*, to differentiate it from neutralization in which the anti-α4β7 antibody binds to the virus to prevent infection. The anti-α4β7 antibody is known to restrict the trafficking of lymphocytes to the gut [2,4], as it inhibits the interaction of the α4β7 integrin expressed on the lymphocyte with MAdCAM-1 expressed on gut endothelial cells [11]. The gut is a region of high viral replication and cellular activation during HIV infection. Thus, limiting the number of α4β7^+^ CD4^+^ T cells that traffic to that region will offer some level of protection from infection—a reduction that is implicitly captured by the protection mechanism.

Giving an anti-α4β7 antibody could improve immune responses due to increased *antigen presentation*, as there would be a greater uptake of opsonized virus by antigen presenting cells [9] (Fig 1E).

Because obtaining virological control using anti-α4β7 monoclonal antibody adjunctive therapy carries a level of importance to the field of HIV treatment and cure, the original experiment was repeated by two different groups using the same viral stock. Neither experiment led to long-term viral control [12,13]. Byrareddy et al. [2] used a SIV *nef-stop* virus to better replicate chronic HIV infection [14]. After an animal is infected, mutation can remove the stop codon and subsequent selection can change the dominant viral species, leading to stochastic effects. We highlight the effects of a *nef-stop* in our results, as well as elaborate on several other possible explanations for the discrepancy between the original and repeat experiments [12,13,15], in the Discussion.

Distinguishing among the four mechanisms—each of which could plausibly lead to viral suppression—is challenging because it is an outcome of complex viral-immune system interactions. The interaction between the virus, infected cells and the immune system is critical in determining whether the virus rebounds to a high viral set-point or remains below the limit of detection (< 50 SIV RNA copies/ml) after the removal of all treatment. If the viral suppression induced by the anti-α4β7 antibody were too strong, the resulting levels of viral replication might not be sufficient to stimulate an effective antiviral response, enabling the virus to escape the immune system after the anti-α4β7 antibody dissipates. Conversely, if the effects of the antibody were too weak, then the virus-host interaction might not be perturbed enough to suppress viral replication after the removal of all therapy. A systematic understanding of how cART and anti-α4β7 antibody affects the viral immune system interactions to achieve viral suppression in the absence of treatment is critical to the design and implementation of new therapeutic strategies and to understand the mechanisms operating in different repeat studies.

## Methods

### Ethics statement

The experimental protocols were reviewed and approved by the Emory University Institutional Animal Care and Use Committee. The animals were bred and maintained at Yerkes National Primate Research Center (Emory University, Atlanta, Georgia) in their nonhuman primate facilities. The diets of the macaques consisted of a monkey diet (Purina, Gray Summit, Missouri), along with daily fresh fruit and/or vegetables and water ad libitum. To ensure to social enhancement and their well-being, macaques were housed in a temperature controlled indoor facility with a 12-hour light/dark cycle and caged with socially compatible same sex pairs. The Yerkes enrichment staff oversaw and provided appropriate safe toys for further social enrichment. Animal health was closely monitored, with veterinary staff directing treatment for any signs of distress or disease. If symptoms could not be alleviated by the directed treatment, the macaque was humanely euthanized according to the directed guidelines of the American Veterinary Medical Association.

### Mathematical model

To reproduce both the post-treatment high viral load set-point observed in the control IgG treated macaques and the maintained low viral load seen in the anti-α4β7 antibody treated macaques (S1 Fig), we adapted a previously developed mathematical model by Conway and Perelson that allows for multiple viral load set-points [7]. We incorporated the various anti-α4β7 antibody mechanisms of action into this model (Eqs (1)–(8)), which includes uninfected CD4^+^ T cells, productively infected cells, latently infected cells, cytotoxic effector cells, and virus.

First, we describe the base model that applies to the IgG control macaques, who did not receive the anti-α4β7 antibody, *A*_*B*_. We then describe the additional model terms involving the proposed anti-α4β7 antibody mechanisms of action. Lastly, we introduce a pharmacokinetic model to describe the time-dependent changes in the anti-α4β7 antibody concentration, which for ease of notation we denote *A*_*B*_ rather than *A*_*B*_*(t)*.


$$\begin{array}{l} \frac{dT}{dt}=r_{T}T\left(1-\frac{T+I+L+P}{k}\right)-d_{T}T-(1-\varepsilon )\beta TV\left(1-I_{N}\frac{\psi_{N}A_{B}}{1+\psi_{N}A_{B}}\right)\\ \;-\rho \frac{A_{B}}{A_{B}+EC_{50}}T(k_{\alpha 4\beta 7}-\frac{P}{P+T+I+L})+\omega \frac{EC_{50}}{A_{B}+EC_{50}}P, \end{array}$$ (1)

$$\frac{dI}{dt}=(1-f)(1-\varepsilon )\beta TV\left(1-I_{N}\frac{\psi_{N}A_{B}}{1+\psi_{N}A_{B}}\right)+\alpha L-I(\delta +mE),$$ (2)

$$\frac{dL}{dt}=f(1-\varepsilon )\beta TV\left(1-I_{N}\frac{\psi_{N}A_{B}}{1+\psi_{N}A_{B}}\right)-\alpha L-d_{L}L+r_{L}L,$$ (3)

$$\frac{dV}{dt}=pI-cV\left(1+(\gamma -1)\frac{\psi_{C}A_{B}}{1+\psi_{C}A_{B}}\right),$$ (4)

$$\frac{dE}{dt}=\lambda_{E}(1+I_{A}\frac{A_{B}}{A_{B}+\Omega })-\mu E+b_{E}E\frac{I}{I+K_{B}}-d_{E}E\frac{I}{I+K_{D}},$$ (5)

$$\frac{dP}{dt}=r_{T}P(1-\frac{T+I+L+P}{k})+\rho \frac{A_{B}}{A_{B}+EC_{50}}T(k_{\alpha 4\beta 7}-\frac{P}{P+T+I+L})-d_{T}P-\omega \frac{EC_{50}}{A_{B}+EC_{50}}P.$$ (6)

We define target cells, *T*, to be uninfected activated/proliferating CD4^+^ T cells [16–18]. Target cells proliferate logistically at maximum rate *r*_*T*_ [16,17,19], have a carrying capacity *k* and die at per capita rate *d*_*T*_.

Target cells become infected through cell-free virus infection at rate β*VT*, where β is the infection rate constant and *V* is the virus concentration, *i*.*e*., plasma SIV RNA/ml. A fraction 1-*f* of infected target cells become productively infected cells, *I*, while the remaining proportion *f* become latently infected cells, *L*. In the Byrareddy et al. [2] study, all macaques received an integrase inhibitor and two reverse transcriptase inhibitors between five and 19-weeks p.i. These antiretroviral drugs interfere with the infection process. More complex models have been used to describe the action of integrase inhibitors [20] but this level of detail is not needed here due to the infrequent viral sampling. Thus, we assume cART reduces the SIV infection rate by the factor *ε*, where *ε* ∈ [0,1] is the efficacy of cART.

Productively infected cells, *I*, produce virions at rate *p* per cell, which are naturally cleared at rate *c* per virion. Productively infected cells die due to viral cytopathic effects at per capita rate δ and are killed at rate *mE* by cytotoxic effector cells, *E*. Thus, the overall death rate of a productively infected cell is δ + *mE*. We consider only cytotoxic effector cells that contribute to infected cell death, which can consist of both natural killer (NK) and SIV-specific cytotoxic T cells.

The generation of a cell that is productively infected can also occur by activation of a latently infected cell, which occurs at rate *α*. There is no evidence indicating that latently infected cells have a carrying capacity to allow for homeostatic control, as seen in the total CD4 memory population. Thus, we assume latently infected cells proliferate at per capita rate *r*_*L*_ and die at per capita rate *d*_*L*_. The half-life of the latent cell population is then *ln* (2)/(*α*+*d*_*L*_-*r*_*L*_).

As described previously by Conway and Perelson [7], cytotoxic effector cells are produced at rate *λ*_*E*_ and die at per capita rate *μ*. The presence of infected cells leads to their proliferation at rate *b*_*E*_*EI*/(*I+K*_*B*_), with *b*_*E*_ denoting the maximum proliferation rate and *K*_*B*_ being a half-saturation constant for proliferation. During viral infection, effector cells can become exhausted [21–23] causing the cells to lose effector function [24]. Exhaustion of cytotoxic effector cells occurs at rate *d*_*E*_*EI*/(*I+K*_*D*_), where *d*_*E*_ represents the maximum rate of exhaustion, and *K*_*D*_ is the half-saturation constant for cytotoxic effector cell exhaustion.

Conway and Perelson used a constant source of effector cells [7]. As effector CD8^+^ T cell differentiate from naïve CD8^+^ T cells, we also examined two alternative models for the source of effector cells (S1 and S2 Texts).

### Mechanism 1: Anti-α4β7 antibody increases viral clearance

The anti-α4β7 antibody can increase the clearance of the virus through opsonization of the α4β7-coated viruses [9]. We assume that the kinetics of anti-α4β7 antibody binding to α4β7^+^ virus is much faster than the changes in viral load so that equilibrium binding relations apply. We further assume antibody binding enhances the viral clearance rate a maximum of γ-fold compared to the viral clearance rate of antibody-free virus and that the clearance rate depends on the amount of antibody bound. Thus, the overall viral clearance rate in the presence of anti-α4β7 is *c*(1 + (*γ*−1*)ψ*_*C*_*A*_*B*_ / (1 + *ψ*_*C*_*A*_*B*_)) (S1 Text), where 1/*ψ*_*C*_ is the half-maximal effective antibody concentration (Fig 1B). As the antibody is infused multiple times, the antibody concentration changes during the experiment and thus an antibody concentration dependent clearance rate needs to be used in the model.

### Mechanism 2: Anti -α4β7antibody reduces the infection rate by neutralizing the virus

With the anti-α4β7antibody having the potential to bind to α4β7^+^ virus, neutralization could be an additional mechanism of antibody action. We test this by setting the indicator variable 𝕀_*N*_ = 1 if we assume viral neutralization is present and 𝕀_*N*_ = 0 if we assume neutralization is absent in Eqs (1)–(3). The maximum neutralizing efficacy of the anti-α4β7 antibody is assumed to be 100%. As we assume anti-α4β7 antibody binding rapidly reaches equilibrium, we use (1- 𝕀_*N*_ *ψ*_*N*_ *A*_*B*_/ (1 + *ψ*_*N*_ *A*_*B*_)) (S1 Text) as the factor by which antibody reduces the infection rate, where 1/*ψ*_*N*_ is the antibody concentration for 50% neutralization. Since neutralization requires the anti-α4β7 antibody to bind to the virus, we account for increased viral clearance in addition to the inhibition of infection when we model this mechanism of action (Fig 1C). To limit the number of free parameters, we assumed that *ψ*_*N*_ = *ψ*_*C*_.

### Mechanism 3: Anti-α4β7 antibody can bind to α4β7^+^ CD4^+^ T cells and protect them from infection

The anti-α4β7 antibody can block infection of α4β7^+^ CD4^+^ T cells [4,10]. Also, the anti-α4β7 antibody decreases trafficking of CD4^+^ T cells to the gut [2,4]. As the gut is a site of high viral replication, reducing the trafficking of α4β7^+^ CD4^+^ T cells to this site could also contribute to the inhibition of infection. Not all CD4^+^ T cells express α4β7. During acute SIV infection, the maximum percentage of peripheral blood CD4^+^ T cells that express α4β7 is approximately 73% [1]. Thus, we restrict the fraction of target cells protected from infection by an anti-α4β7 antibody to be less than the fraction of α4β7^+^ CD4^+^ T cells—denoted by *k*_*α*4*β*7_. To limit the number of target cells in the protected state (*P*) to those that are α4β7^+^, we used a logistic function, $\rho T(k_{\alpha 4\beta 7}-\frac{P}{P+T+I+L})A_{B}/(EC_{50}+A_{B})$in Eqs (1) and (6), where *ρ* is the maximum rate target cells enter the protected state and EC_50_ is the antibody concentration needed for half-maximal effect. After anti-α4β7 treatment the antibody concentration wanes and protected cells can become target cells, as the antibody reversibly binds to cell-associated α4β7 and will dissociate. We assume the rate that protected cells become target cells again is dependent on the plasma concentration of the anti-α4β7 antibody according to $\omega \frac{EC_{50}}{A_{B}+EC_{50}}$,with protection waning at maximum rate ⍵ (Eqs (1) and (6)) (Fig 1D).

### Mechanism 4: Anti- α4β7 antibody increases antigen presentation

The anti-α4β7 antibody could increase antigen presentation due to greater uptake of α4β7^+^ virus by antigen presenting cells and lead to a more robust adaptive immune response [9]. We test this by setting the indicator variable 𝕀_*A*_ = 1 if we assume an increase in antigen presentation and 𝕀_*A*_ = 0 if we assume this mechanism is absent in Eq (5). We account for this improvement in the presence of the anti-α4β7 antibody by increasing the source rate *λ*_*E*_ by a factor of 1 + $I_{A}\frac{A_{B}}{A_{B}+\Omega }$, where Ω is the half-saturation constant for the antibody effect that enhances antigen presentation to generate more cytotoxic effector cells (Fig 1E). To limit the number of free parameters, we assume that the source rate is at most double based on *in vitro* experiments [25]. Also, this approach assumes that in the presence of the anti-α4β7 antibody, antigen presenting cells increase the rate effector cell precursors become activated, consistent with the observation that blocking the β7 integrin with a monoclonal antibody enhances MHC-I presentation [25].

In the supplementary material (S2 Text), we consider a version of this mechanism that requires the antibody to bind to the virus. We include the effects of increased viral clearance when studying mechanism 4 in this alternative scenario (Fig 1E and S2 Text). For the effector cell source model that includes antigen presenting cells, we explicitly model the interaction between virus in complex with the antibody and antigen-presenting cells within mechanism 4 (S1 Text).

### Pharmacokinetics of the anti-α4β7 antibody

To describe the pharmacokinetics of the anti-*α*4*β*7 antibody, we used a two-compartment pharmacokinetic (PK) model [26] in which the anti-*α*4*β*7 antibody is infused into the bloodstream at rate *A*_*α*4*β*7_(*t*), and disseminates to the tissue at rate *k*_12_. Once in the tissue, the antibody either re-enters the bloodstream at rate *k*_21_ or is eliminated at rate *k*_0_ (S1 Text). The equations describing the PK dynamics are

$$dX_{B}/dt=A_{\alpha 4\beta 7}(t)v_{B}-k_{12}X_{B}+k_{21}X_{T},\text{and}$$ (7)

$$dX_{T}/dt=k_{12}X_{B}-(k_{21}+k_{0})X_{T},$$ (8)

where *X*_*B*_ and *X*_*T*_ are the total amounts of antibody in the blood and tissues, respectively, *v*_*B*_ is the volume of blood, and thus the concentration of the antibody in the blood is *A*_*B*_ = *X*_*B*_/*v*_*B*_.

### Model parameters

We fixed many parameters with estimates from the literature (Table 1) and others were determined by fitting to the viral load data in Byrareddy et al. [2] and to macaque pharmacokinetic data [3] using maximum likelihood methods (S14–S19 Tables) (S1 Text).

**Table 1:** Parameter definitions and values.

Parameter	Description	Value	Units	Reference
*d*_*T*_	Uninfected CD4^+^ T-cell death rate	0.01	per day	[18,53]
*r*_*T*_	CD4^+^ T-cell proliferation rate		per day	Estimated for individual macaques
*κ*	Carrying capacity for the CD4^+^ T cell population	^a^	cells/ml	Calculated
*α*	Latent cell activation rate	0.001	per day	[7]
*d*_*L*_	Latent cell death rate	^b^	per day	Calculated
*r*_*L*_	Latent cell proliferation rate	^b^	per day	Calculated
*d*_*L*_+ *α* -*r*_*L*_	Decay rate of the latent cell reservoir	0.0182^b^	per day	[32]
*f*	Fraction of infections resulting in latency	10^−5^		Calibrated^c^
*m*	Rate cytotoxic effector cells kill infected cell		ml/cell per day	Estimated for individual macaques
*δ*	Infected cell death rate due to viral cytopathic effects in baseline model	0.60	per day	Calibrated
*p*	Viral production rate		RNA copies per cell per day	Estimated for individual macaques
*c*	Viral clearance rate	23	per day	[54–56]
*λ*_*E*_	Effector cell source rate	10^3^	cells / ml per day	Calibrated^d^
*μ*	Death rate of cytotoxic effector cells	0.32	per day	[30]
*b*_*E*_	Maximum proliferation rate for cytotoxic effector cells	1.62	per day	[30,31]
*d*_*E*_	Maximum exhaustion rate for cytotoxic effector cells in baseline model	1.35	per day	Calibrated
*K*_*B*_	Saturation constant for cytotoxic effector cell expansion		cells/ml	Estimated for individual macaques
*K*_*D*_	Saturation constant for cytotoxic effector cell exhaustion	55 *K*_*B*_	cells/ml	[32]
*ε*	Efficacy of cART	0.90		Assumed
*T*(0)*β*	Free virus infection rate	5 ⨉ 10^−3^	cells/SIV RNA per day	Assumed
*A*_*α*4*β*7_	Total amount of anti-*α*4*β*7 antibody infused	805	*μ*g/ml	50 mg/kg for a 5.3 kg [57] macaque with 329 ml of blood
*V*(0)	Initial virus concentration	413	SIV RNA copies/ml	Estimated
*k*_*0*_	Elimination rate of anti-*α*4*β*7 antibody	0.048	per day	Estimated
*k*_*12*_	Distribution rate of anti-*α*4*β*7 antibody to tissue	0.165	per day	Estimated
*k*_*21*_	Distribution rate of anti-*α*4*β*7 antibody to blood from tissue	0.008	per day	Calibrated^e^
*κ*_*α*4*β*7_	Fraction of CD4^+^ T cells that are *α*4*β*7^+^	0.73		[1]
EC_50_	Half-maximal concentration for anti-*α*4*β*7 antibody binding to CD4 T cells	2.76 ⨉ 10^-2^	*μ*g/ml	[58]
*K*_*P*_	Saturation constant for the source of effector cells in the saturated source model		cell/ml	Estimated for individual macaques
*γ*	Fold increase in viral clearance rate			Estimated for individual treated macaques
*1/ψ*_*C*_	Antibody concentration of half-maximal effect for increased viral clearance		*μ*g/ml	Estimated for individual treated macaques
*1/ψ*_*N*_	Antibody concentration of half-maximal effect for neutralization	*1/ψ*_*N*_ = *1/ψ*_*C*_	*μ*g/ml	Estimated for individual treated macaques
𝕀_*N*_	Indicator variable for the presence of the effect of viral neutralization from the anti-*α*4*β*7 antibody	1 when present; 0 otherwise		
𝕀_*A*_	Indicator variable for the presence of the effect of an increase in the effector cell source rate from the anti-*α*4*β*7 antibody	1 when present; 0 otherwise		
*⍵*	Maximum rate protection wanes		per day	Estimated for individual treated macaques
*⍴*	Maximum rate target cells become protected		per day	Estimated for individual treated macaques
Ω	Half-saturation constant for the effect the anti-*α*4*β*7 antibody has on increasing antigen presentation		*μ*g/ml	Estimated for individual treated macaques

*a* The carrying capacity was calculated such that in the absence of productively infected and latently infected cells the CD4^+^ T cell population would be in steady state at the initial target cell concentration.

*b* Specifying the latent cell activation rate, the difference between the latent cell death rate and the latent cell proliferation rate is such that the half-life of the latent cell reservoir is 38 days.

*c* The fraction of infections resulting in latency was calibrated during the initial stages of fitting such that the viral load under cART was greater than 1 SIV RNA copies/ml during chronic HIV infection.

*d* The source rate of effector cells was chosen such that the effector cell concentration in the baseline model was relatively comparable to the concentration of HIV specific CD8^+^ T cells during HIV infection.

*e* The distribution rate of the antibody to the blood from the tissue was calibrated at 0.001 intervals to maximize the likelihood of the antibody dynamics from the pharmacokinetic model.

According to Sachsenberg et al [19], 1.1% of the total CD4+ T cell population (10^6^ cells/ml) in an HIV individual is Ki67^+^ and likely in cell cycle. Thus, we assume the initial number of target cells, *T*(0) = 1.1 x 10^4^ cells/ml. We specify β*T*(0) = 5 × 10^−3^ cells/day, as estimates of the HIV infection rate β tend to vary between 10^−7^ and 10^−8^ ml/day [18,27,28]. To obtain an effector cell concentration in the absence of all therapy that was relatively comparable in scale to empirical estimates of HIV-specific CD8^+^ T -cell concentrations [29], we set the effector cell source rate λ_E_ = 10^3^ cells/ml/day. We set the maximum expansion rate of the effector cells *b*_*E*_ = 1.62/day based on past SIV studies [30,31] and the effector cell death rate μ = 0.32/day [30]. We specified the fraction of infections resulting in latency *f* = 10^-5^, so that the predicted viral load while on cART during chronic infection was that observed in chronic HIV infected patients on cART, where we assumed that the efficacy of cART is 90%. These values were later varied in a sensitivity study. Starting five weeks after infection, macaques were given cART for up to 14 weeks. As cART was given during acute SIV infection and for a short time, we used the prior estimate of a 38-day half-life of the SIV latent cell reservoir [32] based on a study in which reservoir decay was measured in pigtail macaques given four antiretroviral drugs starting at 12 days post infection for approximately 23 weeks [33].

### Parameter estimation

To determine the maximum effector cell exhaustion rate, *d*_*E*_, and the infected cell death rate due to viral cytopathic effects, δ, we conducted a primary grid search on these two parameters across the seven control macaques. The values of these two parameters that maximized the combined likelihood across the seven control macaques were used in the model fitting of the treated macaques and model simulations of the control and treated macaques (S1–S3 Tables).

**Fig 2:**
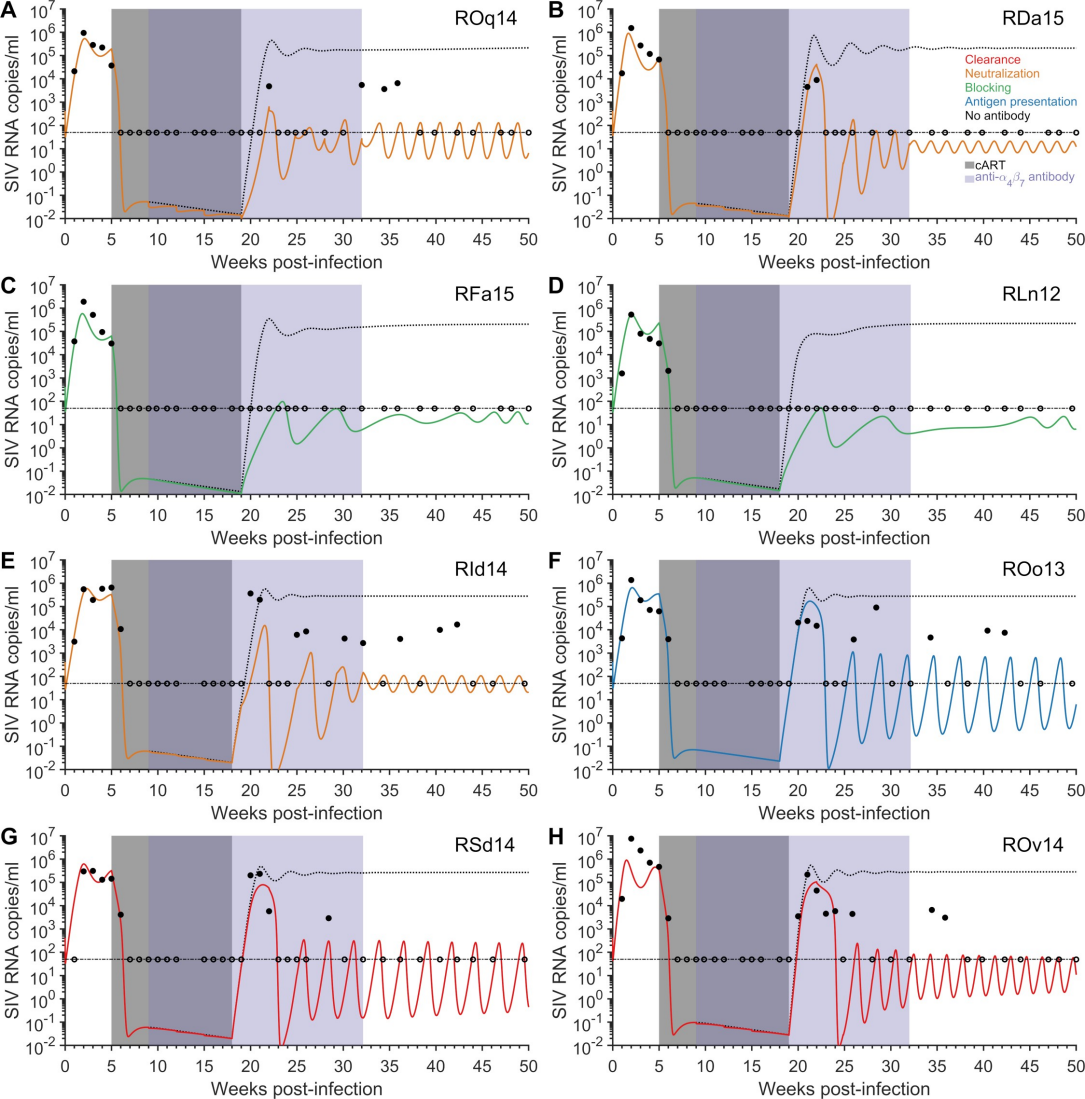
Fit of the model to the data. The measured (≥50 SIV RNA copies/ml solid circles and <50 SIV RNA copies/ml open circles) and model predicted viral loads (solid line) using the best-fit parameter estimates and model variation with the greatest AIC weight for each of the treated macaques and predicted viral dynamics in the absence of the anti-α4β7antibody (dotted black line), panels A)–H). The limit of detection is 50 SIV RNA copies/ml (thin horizontal dashed black line). Treatment with cART occurred between five weeks and 18/19 weeks post-infection (gray area), while eight infusions of the anti-α4β7antibody occurred between nine weeks post-infection and 32 weeks post-infection (purple area). The mechanisms considered include increased viral clearance (red line), virus neutralization (orange line), target cell protection (green line), and increased antigen presentation (blue line). Parameters for these simulations are in Tables 1 and S14–S18, for the AIC selected model (Table 2).

According to the mathematical model, each of the eight anti-α4β7 treated macaques (henceforth referred to as treated macaques) has one viral load set-point above 50 SIV RNA copies/ml and another other below 50 SIV RNA copies/ml (Fig 2 and S1–S3 Figs). The achievement of post-treatment control after the removal of cART was not observed in the seven IgG control macaques, and there is only moderate variability in viral dynamics among these animals after stopping cART (S6–S8 Figs). The viral dynamics among the treated macaques after the removal of cART is highly variable. Our fitting approach for integrating the IgG control viral load data and accounting for the individual heterogeneity in the treated macaques, is motivated by methods used in population sciences to identify heterogeneous treatment effects [34]. We paired each treated macaque with a control macaque that received normal IgG, simultaneously fitting the model to the viral load data of the two macaques, provided the opportunity to disentangle the effects of the effector cell response and the mechanism of action of the antibody. This fitting process using the one-to-one pairing was repeated for each IgG control macaque, such that all the treated macaque were paired with every IgG control macaque (S1 Text). We assumed that the IgG control macaques are a well-representative population of macaques who did not receive the anti-α4β7 antibody. Thus, we assume the viral dynamics of each of the eight treated macaques would approach the viral steady state above 50 SIV RNA copies/ml and resemble that of an IgG control macaque after cART was stopped and if the anti-α4β7 antibody were not given.

We computed the 95% confidence intervals using the profile likelihoods of the estimated parameters and a threshold for the log-likelihood based on half the value of the 95% percentile of a *χ*^2^distribution with *k* degrees of freedom [35], where *k* is the number of estimated parameters (S1 Text).

### Model selection

For model selection, we calculated the Akaike information criterion (AIC) weight [36]. The small sample size corrected AIC score for model *i* and macaque *j* is

$$AIC_{i,j}=-2\;ln\;(L_{i,j})+2k_{i}+2k_{i}(k_{i}+1)/(n_{j}-k_{i}-1),$$

where *L*_*i*,*j*_ is the likelihood for model *i* and macaque *j*, *n*_*j*_ is the number of observations, and *k*_*i*_ is the number of parameters for model *i* [37]. The AIC weight for model *i* and macaque *j* is

$$w_{i,j}=\frac{exp\{-\Delta AIC_{i,j}/2\}}{\sum_{i=1}^{n}exp\{-\Delta AIC_{i,j}/2\}},$$

where Δ*AIC*_*i*,*j*_ = *AIC*_*i*,*j*_−*min*_*k*_{*AIC*_*k*,*j*_} [36]. As the AIC weight of a model (i.e., mechanism) can be viewed as the probability that the model is the best one, we averaged the weights across all the macaques for each model to evaluate the relative importance of the model (i.e., mechanism) in explaining the observed dynamics.

### Sensitivity

As in other viral dynamic models, many of the estimated parameters are correlated [38,39]. The estimated value of the effector cell killing rate, *m*, scales with the assumed values of the effector cell source rate, λ_E_, and the rate antigen presenting cells encounter antigen, *b*_*D*_, for the model presented in S1 Text. The free-virus infection rate, *βT*(0), is correlated with the estimated value of the viral production rate, *p*. We performed a sensitivity analysis for the efficacy of cART, *ε*, the fraction of new infections that are latent, *f*, and the rate latent cells are activated (with the latent cell reservoir half-life fixed at 38 days), *a*, and the impact these parameters have on the average viral load dynamics in the IgG control and treated macaques.

We also conducted an extensive sensitivity analysis, on the fraction of new infections that are latent, *f*, the rate latent cells are activated, *a* (which influences the half-life of the latent cell reservoir because we keep the latent cell proliferation rate, *r*_*L*_, fixed), the efficacy of cART, *ε*, the maximum rate of effector cell expansion, *b*_*E*_, the ratio of *K*_*D*_ to *K*_*B*_, the effector cell death rate, μ, the percentage of CD4+ T cells that are targets, *T*(0) (which influences *β* as *βT*(0) = 5 × 10^−3^ cells/day), the initial viral inoculum, *V*(0), and the effector source rate, λ_E_, based on changes in the log-likelihood value (S3 Text).

### Simulating nef-competent SIV

In Byrareddy et al. [2], SIVmac_239_ *nef*-stop was used to infect the macaques. Major histocompatibility complex (MHC) class I molecules on the surface of SIV and HIV-infected cells are downregulated by *nef* [40,41], allowing infected cells to evade CD8^+^ T effector cell responses to some degree. Since peptide-MHC class I molecules are recognized by the T cell receptor on CD8^+^ T cells, we speculate that cells infected with a *nef*-competent virus will not stimulate effector cell expansion as well as the *nef*-defective virus used by Byrareddy et al. [2]. Therefore, to model a *nef*-competent virus, such as in the repeat experiment by Abbink et al. [15], we reduced the effector cell killing rate *m* and increased the half-saturation constant for effector cell proliferation *K*_*B*_, while fixing the remaining best-fit parameters for each treated macaque (i.e., the half-saturation constant for effector cell proliferation *K*_*D*_ remains fixed and is not increased with *K*_*B*_) (S1 Text). We implemented a grid search for the perturbation of these two parameters within fixed ranges that would give rise to viral rebound (S1 Text).

## Results

**Fig 3:**
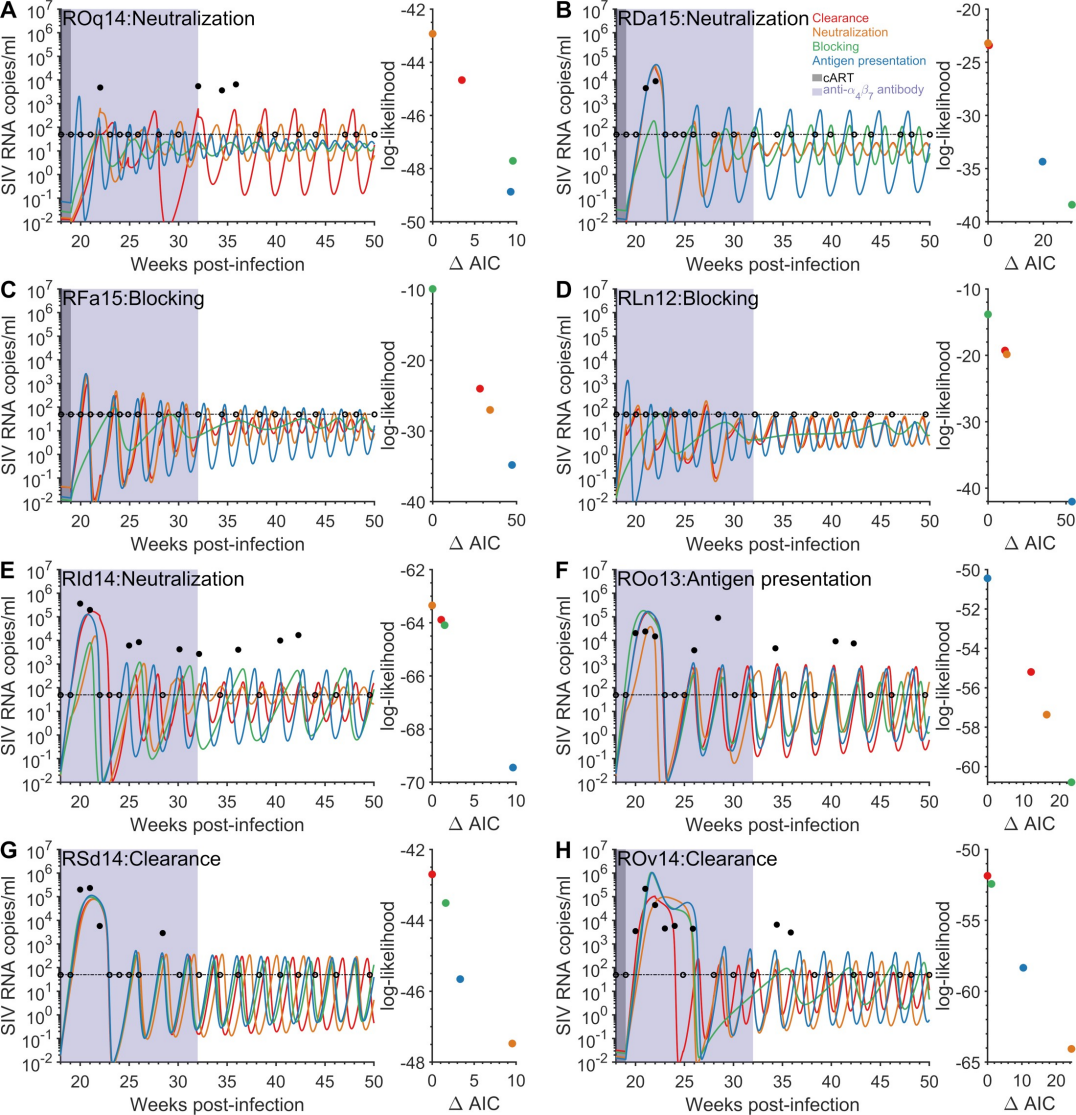
Viral dynamics after the removal of cART for the four anti-*α*4*β*7 antibody mechanisms of action. The measured (≥50 SIV RNA copies/ml solid circles and <50 SIV RNA copies/ml open circles) and model predicted viral loads for the AIC selected model (indicated after macaque) and three remaining models using the best-fit parameter estimates for the mechanisms of increased viral clearance (red line), viral neutralization (orange line), target cell protection (green line), and increased antigen presentation (blue line), panels A)–H). For each macaque, a scatter plot of the ΔAIC and the log-likelihood for each mechanism, panels A)–H). The limit of detection is 50 SIV RNA copies/ml (thin horizontal dashed black line). Treatment with cART occurred between five weeks and 18/19 weeks post-infection (gray area), while eight infusions of the anti-α4β7antibody occurred between nine weeks post-infection and 32 weeks post-infection (purple area). The mechanisms considered include parameters for these simulations are in Tables 1 and S14–S18.

The achievement of post-treatment virologic control after the removal of treatment is dependent upon obtaining a balance between viral growth and the immune control. Models that differed by the mechanism of action of the anti-α4β7 antibody were able to attain post-treatment control with the administration of the anti-α4β7 antibody, with most reproducing the observed viral dynamics (Figs 2 and 3 and S2–S5). In addition, the model could portray the viral dynamics in the IgG control macaques (S6–S8 Figs). With multiple models fitting the viral load after the removal of cART in both the IgG control and anti-α4β7 antibody treated macaques, we next determined which models gave the best fit for each individual animal.

### Viral load dynamics best reproduced with baseline source of effector cells

As described in the Supplemental Information (S1 Text), we tested three different models for the source of effector cells in the absence of anti-α4β7 antibody therapy: a baseline model where the source rate is independent of infected cells, virus, or antigen presenting cells; a saturated source dependent on the infected cell concentration or equivalently the virus concentration as the virus and infected cell levels rapidly establish a quasi-steady state in which they are proportional to each other; and a more mechanistic model where the source is dependent on the concentration of antigen presenting cells. We examined which model best fit the viral load dynamics of the IgG control and treated macaques using AIC weight, where models with the larger weights (lower AIC score) are selected [36]. Given an effector source model, the best mechanism of action of the anti-α4β7antibody based on AIC was used for each treated macaque in the model comparison (S5–S12 Tables). Overall, we found that the baseline model was the best option to generate the viral load dynamics seen in both the treated and IgG control macaques (S4 Table), showing that adding more biological complexity does not improve the fits enough to justify the inclusion of additional parameters. Given this finding, we only discuss the results obtained with the baseline model below. We note that this AIC selected model is not universally favored across the different mechanisms for the individual macaques (S5–S13 Tables).

### Viral dynamics in the treated group is best explained by the protection mechanism

We considered four mechanisms by which the anti-α4β7 antibody perturbs the balance between viral replication and immune control (Fig 1). To determine which mechanism best explains the viral load dynamics for the treated macaques, we fit the model to the viral load data from each monkey and compared the mechanisms using the AIC weight (Table 2 and Figs 2 and 3). We found that the protection mechanism provides the overall largest AIC weight on average for the treated macaques (Table 2), while some individual macaques have different optimal mechanisms (Table 2). Increased viral clearance and viral neutralization follow second and third, respectively, for explaining the observed viral load dynamics in the eight treated macaques.

**Table 2:** The AIC weight* for increased viral clearance, viral neutralization, protection, and improved antigen presentation mechanisms for the baseline source model.

Mechanism	ROq14	RDa15	RFa15	RLn12	RId14	ROo13	RSd14	ROv14	Average
Viral clearance	0.147	0.452	0.000	0.004	0.282	0.002	**0.609**	**0.637**	0.267
Virus neutralization	**0.838**	**0.548**	0.000	0.002	**0.484**	0.000	0.005	0.000	0.235
Protection	0.007	0.000	**1.000**	**0.993**	0.230	0.000	0.271	0.360	**0.358**
Antigen presentation	0.008	0.000	0.000	0.000	0.004	**0.997**	0.115	0.003	0.141

* AIC weights are rounded and as a result some columns may not sum to one.

The protection mechanism still had the largest average AIC weight when including increased viral clearance into the increased antigen presentation mechanism (Table A in S2 Text). The average AIC weight of increased antigen presentation improved slightly with the addition of increased viral clearance to this mechanism (Table A in S2 Text).

We examined the viral load dynamics predicted by each mechanism post-cART and compared them to the AIC selected mechanism for the individual macaque (Fig 3). For most macaques, the rebound dynamics are qualitatively similar for the different mechanisms. For the two macaques that had no detectable viremia post-cART (RFa15 and RLn12), the protection mechanism was more able to suppress the initial rebound immediately after cART was removed compared to the other four mechanisms which produced peaks slightly above detection. For macaque RId14, which had highly oscillatory viral load dynamics after cART was stopped, the four mechanisms produced qualitatively different oscillatory patterns. For macaque ROo13, the difference in the mechanisms were more apparent in capturing the initial viral load peak after cART was stopped, with relatively similar dynamics afterwards. Although some mechanisms may qualitatively be similar during the phase once cART is removed, the reduced log-likelihood can be attributed to i) poor representation of the viral load dynamics prior to the initiation of cART or ii) a substandard portrayal of the viral load dynamics in the absence of the anti-α4β7 antibody.

### Area under viral load curve post-treatment correlated with the level at which effector cells proliferate

We calculated the area under the predicted log_10_ viral load curve (AUC) for the 30 weeks after cART was removed. The time period of 30 weeks after cART was removed was used instead of a specified time post-infection because cART was stopped at either week 18 or 19 p.i., which would result in different durations in which the AUC is calculated. The estimated half-saturation constant for effector cell proliferation, *K*_*B*_, was a strong predictor of the predicted AUC for the treated macaques (*r =* 0.77, *p* = 0.025) (S9 Fig), suggesting that treated macaques with a faster responding effector cell response were better able to control the virus than those with a delayed effector cell response. A strong correlation was also found between the estimated *K*_*B*_ and the model predicted AUC for the 30 weeks following the last infusion of the antibody (*r =* 0.63, *p =* 0.096), although it was not statistically significant (S9 Fig).

### Sensitivity analysis

**Fig 4:**
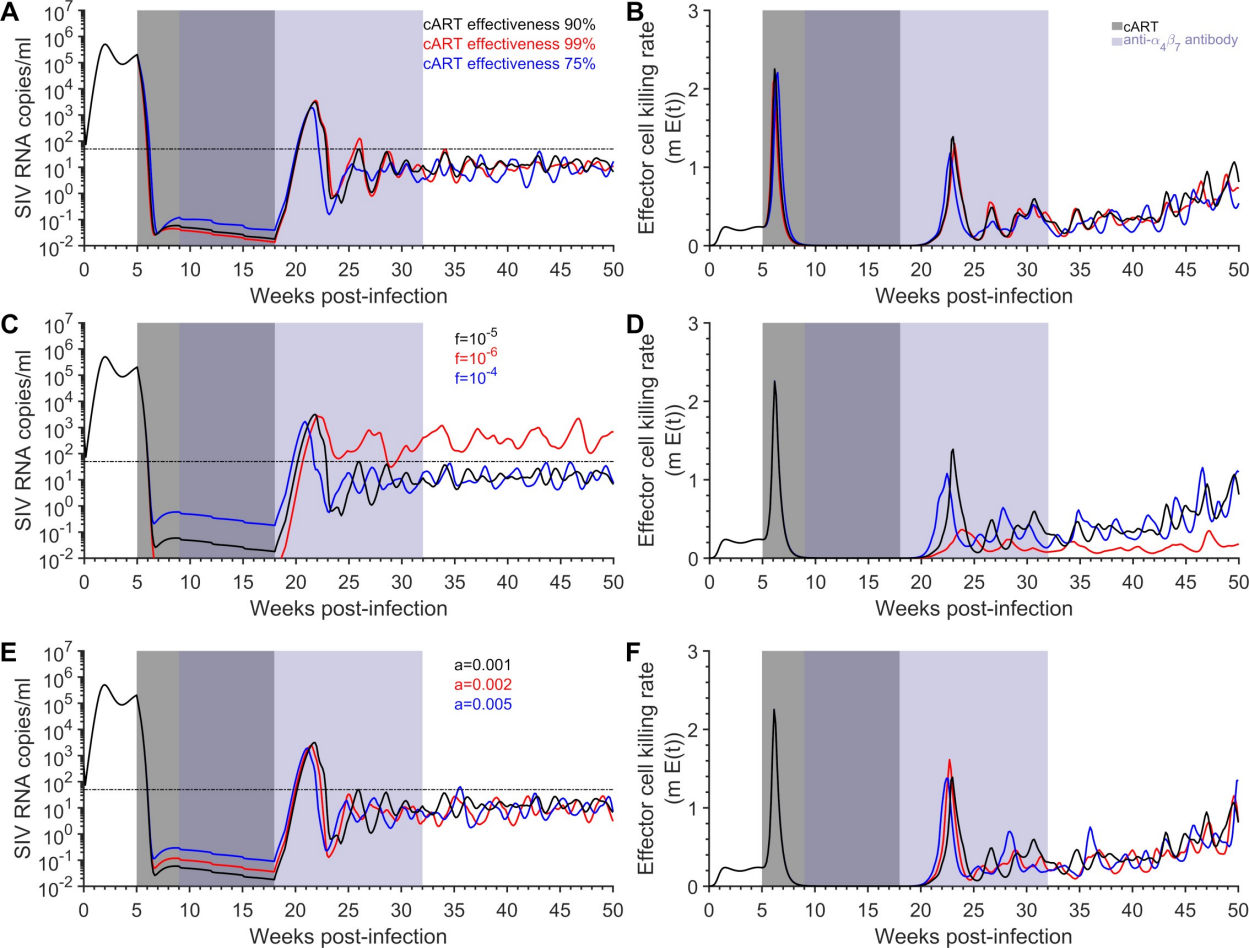
Sensitivity of the viral load dynamics and effector cell killing rate in the treated macaques predicted by the model. The geometric mean of the model predicted viral load (left panels) and the per day effector cell killing rate (right panels) using the best-fit parameter estimates under the baseline source model with the greatest AIC weight for each of the treated macaques. The sensitivity of the viral load and per day effector cell killing rate with respect to changing the A)-B) effectiveness of cART from 90% (black) to 99%(red) and 75% (blue), C)-D) the fraction of infections resulting in latency from 10^−5^ (black) to 10^−6^ (red) and 10^−4^ (blue), and E)-F) the activation rate of latent cells from 10^−3^ (black) to 2 ×10^−3^ (red) and 5×10^−3^ (blue). The limit of detection is 50 SIV RNA copies/ml (thin dashed black line, left panels). Treatment with cART occurred between 5 weeks and 18/19 weeks p.i. (gray area), while eight infusions of the anti-α4β7antibody occurred between nine weeks p.i. and 32 weeks p.i. (purple area). Parameters for these simulations are in Table 1 and S14–S18 Tables, for the AIC selected mechanism (Table 2).

Conducting one-way sensitivity analysis on the efficacy of cART, the fraction of infections resulting in latency and the activation rate of latent cells, the viral load dynamics and the infected cell death rate for the treated macaques are only moderately affected (Fig 4). Increasing the half-maximal concentration (EC_50_) associated with the protection mechanism increases both the breadth and magnitude of the viral peak after the removal of cART (S10 Fig).

Through analysis of the profile likelihood, we found that the value of log-likelihood was sensitive to small changes in the value of our best estimates of the viral production rate, *p*, and the effector cell killing rate, *m* (S3 Text). Moderate changes in parameters associated with the anti-α4β7 antibody mechanism can also produce rapid changes in the log-likelihood (S3 Text). In addition, small changes in fixed parameters pertaining to the effector cell response (ratio of *K*_*D*_ to *K*_*B*_, maximum rate of proliferation *b*_*E*_, and effector cell death rate *μ*) can also produce rapid changes in the log-likelihood (S3 Text). These results are somewhat expected, given the delicate balance between viral growth and immune control for the system to attain post-treatment control after the anti-α4β7 antibody is removed. We conducted similar sensitivity analysis for the IgG control macaques (S11 Fig and S3 Text).

We found that for most of the scenarios that were considered with different effector source models (Table C–H in S2 Text), the protection mechanism was found to be the dominant mechanism when explaining the population level viral dynamics (i.e., greatest average of AIC model weights). We found that increased antigen presentation was the other dominant mechanism in the instances that it performed equally well or outperformed the protection mechanism (Table F in S2 Text).

### Model simulation of nef-competent SIV

Abbink et al. [15] tried to replicate the Byrareddy et al. [2] experiment using a *nef*-competent virus rather than a *nef*-deficient virus, but found that when all treatment was stopped virus rebounded in their experimental animals. Since the presence of *nef* can affect the parameters governing the effector cell response in our model, particularly the effector killing rate constant *m* and the effector cell 50% maximum proliferation constant *K*_*B*_ (see Methods), we systematically varied these parameters for each of the eight treated macaques in the Byrareddy experiment. By changing these parameters, we converted the post-treatment control dynamics to viral rebound dynamics in all eight macaques (S12 and S13 Figs), as observed in the Abbink et al. experiment [15]. We also found that viral rebound occurs across a broader range of perturbations to the effector cell killing rate and the 50% maximum proliferation constant (S13 Fig).

## Discussion

We expanded a viral kinetic model previously used to explain the phenomenon of post-treatment control observed in the VISCONTI study [7,8]. Our generalization incorporated four possible mechanisms of action of the anti-α4β7 antibody: increased viral clearance, virus neutralization, protection of CD4+ T cells from infection, and increased antigen presentation. To examine the role of these mechanisms in explaining the viral dynamics seen in eight rhesus macaques treated with cART plus an anti-α4β7 antibody, we fit our mathematical model to the viral load data from these eight macaques as well as the viral load data from seven control macaques treated with normal IgG and cART [2]. Our mathematical model with best-fit parameters was largely able to reproduce the observed viral dynamics as well as the viral suppression observed in the treated macaques after cessation of all therapy.

Through our analysis, we determined the protection mechanism best explained the population level viral dynamics in all eight macaques treated with the anti-α4β7 antibody. However, there was substantial heterogeneity in the dominant mechanisms across individual animals, as at least one of the four mechanisms were suggested to be the dominant in at least one of the animals. In retrospect, this heterogeneity of mechanism may not be surprising if we view anti-α4β7 antibody treatment as a perturbation of the interactions between the virus and its host that leads to viral suppression and the strengthening of the host immune response so that in the absence of all therapy viral suppression can be maintained. In addition, the macaques were not genetically identical which would lead to differences in the immune responses they developed. Our results reflect these differences, as the model predicted log_10_ viral load AUC after cART and the last anti-α4β7 antibody infusion was strongly correlated with the half-saturation constant in the effector cell proliferation rate function. The individual heterogeneity also suggests that the anti-α4β7 antibody likely has multiple mechanisms of action by which it ultimately suppressed viral rebound. Also, our analysis showed that in some macaques there are secondary mechanisms of anti-α4β7 antibody action that should not be ignored, as they have some explanatory power for the observed viral dynamics (Tables 2 and S5–S12). The individual heterogeneity of the selected mechanism among the treated macaques suggests that less dominant mechanisms could also be present.

These multiple mechanisms likely affect viral replication in overlapping ways. For example, the increased viral clearance mechanism reduces the viral concentration, which subsequently produces fewer infected cells, which in turn could increase effector cell concentrations due to the lack of immune exhaustion. Therefore, the antibody will likely have both direct and indirect effects on viral replication that may not be appropriately quantified.

The characteristics of the viral dynamics post-cART were highly heterogeneous across the eight treated macaques. After removal of cART, two macaques experienced no viremia above the limit of detection, two exhibited viral peaks only while the antibody was administered, two had a broad viral peak after the last infusion of the anti-α4β7 antibody, and the remaining two exhibited large oscillations in the viral load. Some of the variability of these dynamics may be due to stochastic reactivation of cells in the latent reservoir, which was not accounted for in our deterministic model. Rather, our model produces the observed dynamics through the perturbation of the system and the complex interaction between target cells, the virus, and the effector cell response. The objective of the model was not to provide precise estimates of mechanistic parameters of the anti-α4β7 antibody, as we were limited by the frequency of viral sampling, but rather to show that post-treatment control can be achieved through multiple mechanisms of action.

The four mechanisms considered perturb the balance between viral growth and immune control in different ways. The protection mechanism allows for the accumulation of protected CD4+ T cells during cART, which can produce a lower viral peak that may be below the limit of detection when cART is removed. This buildup of protected CD4+ T cells slows viral expansion after cART is removed, allowing the effector response to quickly suppress viral replication. The effects of the increased viral clearance and the virus neutralization mechanisms during cART can further suppress the viral load and the level of infected cells. During the continued presence of the anti-α4β7 antibody after the removal of cART, these two mechanisms will reduce the rate of *de novo* infections which will aid the immune response in controlling infection but also indirectly influence effector cell dynamics. The increased antigen presentation mechanism perturbs the source of effector cells, which depending on the model is either constant or dependent on the infected cell concentration in the absence of the antibody. The temporal change in the effector cell source rate in the presence of the antibody has the potential to induce oscillations in the effector cell population as the antibody concentration fluctuates due to its periodic infusion, contributing to the variable viral concentrations observed after the removal of cART.

Within the model, post-treatment control is achieved through the combined effect of cART, the anti-α4β7 antibody, and the effector cell response. HIV treatment interruption studies revealed that lower viral load set-points after stopping cART could be attributed to an increased response from HIV-specific CD8^+^ T cells [42]. The effector-cell response is a critical component in maintaining viral suppression, as there was a large transient rebound of viremia when CD8α expressing NK cells, NK T cells, and classic CD8 T cells were depleted in the treated macaques (Fig A in S2 Text). After the effector cell population was restored in the macaques, control of viremia was regained (Fig A in S2 Text). Additionally, Cartwright et al. showed that the responses of CD8^+^ T cells are critical to the suppression of viremia in SIV-infected animals treated with short-term cART [43]. CD8 depletion of SIV-infected animals while still on cART lead to viral rebound and subsequent control of viremia when the CD8^+^ T cells repopulated the animals. Interestingly, the viral dynamics observed in that experiment could be reproduced by a generalization of the Conway-Perelson model [32]. A limitation of our model is that we only considered the cytolytic response of effector cells, even though CD8 T cells can also use non-cytolytic mechanisms to limit infection. This was done to avoid adding extra unknown parameters and over-fitting the data. Cao et al [32] modeled the Cartwright et. al. [43] experiment using a variant of the Conway and Perelson model and found that non-cytolytic mechanisms were unlikely to be the sole mechanism of CD8 control [32].

The anti-α4β7 antibody has been hypothesized to enhance antigen presentation, resulting in a better immune response [9]. Broadly neutralizing antibodies may also induce a potent effector cell response by a mechanism in which immune complexes activate antigen presenting cells [44]. Prior studies in mice have shown that passive administration of monoclonal anti-CD20 antibodies to treat melanoma can induce a vaccinal effect, whereby the antibody aids in the development of long-term durable anti-tumor T cell responses [45]. It is hypothesized that this vaccinal effect is a result of the antibody generating immune complexes that activate antigen presenting dendritic cells which then stimulate a cell-mediated immune response [45]. The critical component of the vaccinal effect is the presence of a long-term effective memory response after the antibody has been eliminated from the body. One can speculate that the anti-α4β7 antibody induces a vaccinal effect, as macaques not only maintain long-term suppression of viremia but also viremia rebounds after the depletion of CD8^+^ T cells (Fig A in S2 Text).

One action of an anti-α4β7 antibody is to reduce the trafficking of lymphocytes to the gut [2,4], a site where much viral replication occurs during primary infection [46]. Our results are consistent with this hypothesis, as the protection mechanism best explained population level viral dynamics. To keep our model tractable, we did not explicitly consider trafficking. However, the protection mechanism that we did study can capture the effect of reduced CD4^+^ T-cell trafficking to the gut. Thus, part of the protection mechanism could be viewed as being a consequence of changes in lymphocyte trafficking due to anti-α4β7 antibody administration.

Second to the protection mechanism, both increased viral clearance and virus neutralization were the next set of mechanisms to best explain the data. The Iwamoto et al. study [13] shows that an anti-α4β7 antibody may have little to no neutralizing capabilities. However, cell cultures not treated with retinoic acid (which induces expression of α4β7) can result in cultures producing substantially lower amounts of α4β7 integrated into virions than cell cultures treated with retinoic acid [9]. Girard et al [47] observed limited viral replication in retinoic acid treated α4β7^+^ CD4^+^ T cells when preincubated with an anti-α4β7 antibody, suggesting neutralization may be feasible mechanism. From a model mechanistic standpoint, the virus neutralization mechanism could be representative of both the increased viral clearance mechanism and the protection mechanism. Increased viral clearance is encompassed within the virus neutralization mechanism, which limits infection of target cells, albeit in a different manner than the protection mechanism. We further see a role of increased viral clearance when considering a combined role with the increased antigen presentation mechanism, as the average AIC weight increased for the latter mechanism (S2 Text). This evidence strengthens the suggestion that multiple mechanisms of action from the anti-α4β7 antibody could be perturbing the balance between viral growth and immune control to obtain post-treatment control.

Although there is substantial supportive evidence targeting the α4β7 integrin to alter the course of HIV infection [14], there has been concern about the robustness of the Byrareddy et al. [2] study as other groups were unable to replicate the outcome of the original experiment [12,13,15,48]. The virus used in the original experiment contained a stop codon in *nef*, which upon *in vivo* inoculation in rhesus macaques is stochastically repaired to generate wildtype virus. The stochastic nature of the *nef-stop* repair over the course of infection has been suggested as one potential reason the experiment might not have been replicated [14].

We can only speculate about the reasons underlying the failure to replicate the Byrareddy et al. [2] results. The repeat study by Abbink et al. used a different SIV strain, SIVmac251, and a different route of virus infection that was not optimized to determine the effect of anti-α4β7 on SIV infection and control [15]. Further, this virus is not *nef*-deficient and is more pathogenic than the virus used by Byrareddy et al. [2]. To understand whether *nef*-competence may have played a role in the inability to repeat the original experiment, we asked whether the ability of the model to achieve post-treatment control is sensitive to changes in parameters that *nef* is most likely to influence. Since the presence of *nef* leads to down-regulation of MHC class I, we expect that the effector cell killing rate constant, *m*, would be decreased and that the proliferation of CD8 T cells driven by interaction with infected cells through their T cell receptor would be decreased, i.e., the parameter *K*_*B*_ controlling the amount of stimulation by infected cells needed to induce 50% maximal proliferation would be increased. We showed using our model and best-fit parameters for each macaque that if we decreased *m* and increased *K*_*B*_ that instead of post-treatment control we would get viral rebound when all treatment was stopped as found in the experiment by Abbink et al. [15].

The Iwamoto et al. study [13] also failed to achieve virologic control observed in the Byrareddy et. al. [2] experiment. This failure could be attributed to the use of a different integrase inhibitor. When cART was applied in this system, the decline in the viral load was significantly slower than in the original study. As a result, cART was administered for an extra six weeks to obtain viral suppression before starting anti-α4β7 antibody administration. The authors speculated that more rapid repair of *nef* in this virus could explain the slower virologic control after cART was initiated. As we argued above for the Abbink et al. [15] experiment, having *nef*-competent virus could easily lead to viral rebound instead of post-treatment control.

This study also examined the effect of antibodies against the V2-loop of the SIV envelope protein on viral replication after the removal of cART. They observed that the broadly neutralizing antibody ITS103.01 delayed the rebound of viremia longer than the anti-α4β7 antibody and the non-neutralizing antibody ITS12.01. Unlike the original Byrareddy et al. [2] experiment, all animals rebounded [13]. These antibodies targeting the V2 loop likely would not influence the trafficking of CD4+ T cells to the gut and not interact with non-infected cells that express α4β7 [2]. As a result, the effects of these V2 antibodies might not induce the protection mechanism included in our model of anti-α4β7 antibody action. Further, not all virions express α4β7 and their concentration is likely reduced in later stages of the experiment due to preferential infection and subsequent loss of α4β7 expressing cells. Thus, even if anti-α4β7 antibody has neutralizing ability it would not be as broad as that of ITS103.01. Moreover, ITS103.01 is directed against the CD4 binding site on Env and shows complete neutralization against tier 1, 2 and 3 SIV, including the highly neutralization-resistant SIVmac239 [49]. These factors may explain why the ITS103.01 antibody led to a greater delay in viral rebound than the anti-α4β7 antibody.

The third study by Di Mascio et al. [12] also had issues in attaining post-treatment control. In addition to the stochastic nature of reversion of the *nef-stop* mutation, discussed by Di Mascio et al [12], this experiment was performed using monkeys housed at the NIH Animal Care Center that were fed either 5038-Monkey Diet Jumbo or 5045-High Protein Monkey Diet [12], while the Byrareddy et al. [2] animals were housed at the Yerkes National Primate Research Center and were fed a monkey diet supplemented by fresh fruits and vegetables [2]. This leads us to speculate that the microbiomes of the animals at the two facilities were different. As the composition of the microbiome can affect immune responses and the reliability of an animal model to mimic human immune responses [50] this could be another factor in the differing outcomes. Also, the genetics of the animals might have played a role. The animals used by Di Mascio et al. [12] were obtained from Morgan Island and were genetically typed for a limited number of MHC alleles whereas the animals utilized by Byrareddy et al. [2] were bred in the facilities at the Yerkes Primate Center, Emory University. These macaques were typed for detailed MHC alleles, KIR alleles, FcR polymorphisms and TRIM5alpha genes and the animals assigned to the experimental and control groups to preclude any of these genetic biases. It is also possible that there are other genetic polymorphisms that contribute to the control of viremia, a subject under current study.

In summary, treating acute SIV infection with cART and an anti-α4β7 antibody has led to post-treatment control with viremia levels maintained below 50 SIV RNA copies/ml in an experiment by Byrareddy et al. [2]. No matter what the detailed mechanisms of action of the antibody are, the critical feature of this experiment was that after all treatment was removed, in a majority of animals the virus was controlled to below the level of detection of the assay after an initial high burst of viral replication. A model that yields two stable viral set-points, one where the viral load is below the limit of detection, explains how the antibody can perturb the viral-host system in such a way that it moves the system from the basin of attraction of the high-viral-load set-point to that of the low-viral-load set-point. Based on our analysis, which involved fitting the observed viral load data to models incorporating different mechanisms of antibody action, it appears that multiple mechanisms of antibody action can generate this type of perturbation. Alternatively, one can say that the antibody is generating a vaccinal effect, as the long-term control of the virus in the absence of all therapy ultimately depends on having an effector-cell response in the model. The involvement of effector cells in maintaining virologic control was also shown experimentally using CD8 depletion experiments.

The work presented here introduced new models for four different possible mechanisms by which a monoclonal antibody against the α4β7 integrin could modulate immune responses. These models, although needing further validation, may prove useful in exploring the mechanism of action of other antibodies, such as broadly neutralizing antibodies [51,52], in generating long-term virologic control of SIV/HIV.

## Supporting information

S1 Text Supplementary Methods. (DOCX)

S2 Text Supplementary Results. (DOCX)

S3 Text Profile likelihood and sensitivity of model fit to assumed parameters. (PDF)

S1 Table The aggregated log-likelihood values for the seven control macaques from the primary grid search conducted on the infected cell death rate due to viral cytopathic effects (*δ*) and the maximum rate of effector cell exhaustion (*d*_*E*_) for the baseline effector cell source (BL) model. (PDF)

S2 Table The aggregated log-likelihood values for the seven control macaques from the primary grid search conducted on the infected cell death rate due to viral cytopathic effects (*δ*) and the maximum rate of effector cell exhaustion (*d*_*E*_) for the saturated effector cell source (SS) model. (PDF)

S3 Table The aggregated log-likelihood values for the seven control macaques from the primary grid search conducted on the infected cell death rate due to viral cytopathic effects (*δ*) and the maximum rate of effector cell exhaustion (*d*_*E*_) for the effector cell source model dependent on antigen presenting cells (APCS). (PDF)

S4 Table The average AIC weight for the baseline effector cell source (BL) model, the saturated effector source (SS) model, and the effector source model dependent on antigen presenting cells (APCS). (PDF)

S5 Table The AIC score for increased viral clearance, viral neutralization, protection, and improved antigen presentation mechanisms for the baseline effector cell source (BL) model, saturated source (SS) model, and antigen presenting cell source (APCS) model for macaque RId14. (PDF)

S6 Table The AIC score for increased viral clearance, viral neutralization, protection, and improved antigen presentation mechanisms for the baseline effector cell source (BL) model, saturated source (SS) model, and antigen presenting cell source (APCS) model for macaque RLn12. (PDF)

S7 Table The AIC score for increased viral clearance, viral neutralization, protection, and improved antigen presentation mechanisms for the baseline effector cell source (BL) model, saturated source (SS) model, and antigen presenting cell source (APCS) model for macaque ROo13. (PDF)

S8 Table The AIC score for increased viral clearance, viral neutralization, protection, and improved antigen presentation mechanisms for the baseline effector cell source (BL) model, saturated source (SS) model, and antigen presenting cell source (APCS) model for macaque RSd14. (PDF)

S9 Table The AIC score for increased viral clearance, viral neutralization, protection, and improved antigen presentation mechanisms for the baseline effector cell source (BL) model, saturated source (SS) model, and antigen presenting cell source (APCS) model for macaque RFa15. (PDF)

S10 Table The AIC score for increased viral clearance, viral neutralization, protection, and improved antigen presentation mechanisms for the baseline effector cell source (BL) model, saturated source (SS) model, and antigen presenting cell source (APCS) model for macaque RDa15. (PDF)

S11 Table The AIC score for increased viral clearance, viral neutralization, protection, and improved antigen presentation mechanisms for the baseline effector cell source (BL) model, saturated source (SS) model, and antigen presenting cell source (APCS) model for macaque ROq14. (PDF)

S12 Table The AIC score for increased viral clearance, viral neutralization, protection, and improved antigen presentation mechanisms for the baseline effector cell source (BL) model, saturated source (SS) model, and antigen presenting cell source (APCS) model for macaque ROv14. (PDF)

S13 Table The AIC weight for the baseline effector source (BL) model, saturated source (SS) model, and antigen presenting cell source (APCS) model for the seven IgG control macaques. (PDF)

S14 Table The estimate parameters for the eight treated macaques using the increased viral clearance mechanism for the three different effector cell source models. The intervals specify the range of the set of intervals to approximate the 95% confidence intervals (S1 and S3 Texts).
(PDF)

S15 Table The estimate parameters for the eight treated macaques using the viral neutralization mechanism for the three different effector cell source models. The intervals specify the range of the set of intervals to approximate the 95% confidence intervals (S1 and S3 Texts).
(PDF)

S16 Table The estimate parameters for the eight treated macaques using the protection mechanism for the three different effector cell source models. The intervals specify the range of the set of intervals to approximate the 95% confidence intervals (**S1 and S3 Texts**).
(PDF)

S17 Table The estimate parameters for the eight treated macaques using the increased antigen presentation mechanism for the three different effector cell source models. The intervals specify the range of the set of intervals to approximate 95% confidence intervals (**S1 and S3 Texts**).
(PDF)

S18 Table The estimate parameters for the eight treated macaques using the increased antigen presentation mechanism with increased viral clearance for the three different effector cell source models. The intervals specify the range of the set of intervals to approximate the 95% confidence intervals (S1 and S3 Texts).
(PDF)

S19 Table The estimate parameters for the seven IgG control macaques for the three different effector cell source models. The intervals specify the range of the set of intervals to approximate the 95% confidence intervals (S1 and S3 Texts).
(PDF)

S1 Fig The predicted viral load set-points for the treated macaques. The viral load set points predicted by the model using the best-fit parameter estimates and the mechanism with the greatest AIC weight from A) and B) with the baseline cell source model; C) and D) the model with a saturated source of effector cells; E) and F) a model where the source of effector cells is dependent on the concentration of antigen presenting cells. The maximum and minimum viral load equilibrium points predicted by the model (left column) and estimated by the predicted viral load at 81 weeks p.i. (right column). Parameters for these simulations are in Tables 1 and S14–S18, for the AIC selected mechanism (S5–S12 Tables). We do not consider the alternative mechanism of increased antigen presentation with increased viral clearance in this analysis.
(PDF)

S2 Fig Fit of model to the viral loads of the treated macaques using the saturated source model. The measured (≥50 SIV RNA copies/ml solid circles and <50 SIV RNA copies/ml open circles) and model predicted viral loads (solid line) using the best-fit parameter estimates and model variation with the greatest AIC weight for each of the treated macaques and predicted viral dynamics in the absence of the anti-α4β7antibody (dotted black line), panels A)–H). The limit of detection is 50 SIV RNA copies/ml (thin horizontal dashed black line). Treatment with cART occurred between five weeks and 18/19 weeks post-infection (gray area), while eight infusions of the anti-α4β7antibody occurred between nine weeks post-infection and 32 weeks post-infection (purple area). The mechanisms considered include increased viral clearance (red line), viral neutralization (orange line), target cell protection (green line), and increased antigen presentation (without increased viral clearance) (blue line). Parameters for these simulations are in Tables 1 and S14–S18 and S2 Text, for the AIC selected mechanism (S5–S12 Tables).
(PDF)

S3 Fig Fit of model to the viral loads of the treated macaques using the antigen presenting cell source model. The measured (≥50 SIV RNA copies/ml solid circles and <50 SIV RNA copies/ml open circles) and model predicted viral loads (solid line) using the best-fit parameter estimates and model variation with the greatest AIC weight for each of the treated macaques and predicted viral dynamics in the absence of the anti-α4β7antibody (dotted black line), panels A)–H). The limit of detection is 50 SIV RNA copies/ml (thin horizontal dashed black line). Treatment with cART occurred between five weeks and 18/19 weeks post-infection (gray area), while eight infusions of the anti-α4β7antibody occurred between nine weeks post-infection and 32 weeks post-infection (purple area). The mechanisms considered include increased viral clearance (red line), viral neutralization (orange line), target cell protection (green line), and increased antigen presentation (without increased viral clearance) (blue line). Parameters for these simulations are in Tables 1and S14–S18 and S2 Text, for the AIC selected mechanism (S5–S12 Tables).
(PDF)

S4 Fig Viral dynamics after the removal of cART for the saturated source model. The measured (≥50 SIV RNA copies/ml solid circles and <50 SIV RNA copies/ml open circles) and model predicted viral loads for the AIC selected model (indicated after macaque) and three remaining models using the best-fit parameter estimates for the mechanisms of increased viral clearance (red line), viral neutralization (orange line), target cell protection (green line), and increased antigen presentation (without increased viral clearance) (blue line), panels A)–H). For each macaque, a scatter plot of the ΔAIC and the log-likelihood for each mechanism, panels A)–H). The limit of detection is 50 SIV RNA copies/ml (thin horizontal dashed black line). Treatment with cART occurred between five weeks and 18/19 weeks post-infection (gray area), while eight infusions of the anti-α4β7antibody occurred between nine weeks post-infection and 32 weeks post-infection (purple area). The mechanisms considered include parameters for these simulations are in Tables 1 and S14–S18 and S2 Text.
(PDF)

S5 Fig Viral dynamics after the removal of cART for the antigen presenting cell source model. The measured (≥50 SIV RNA copies/ml solid circles and <50 SIV RNA copies/ml open circles) and model predicted viral loads for the AIC selected model (indicated after macaque) and three remaining models using the best-fit parameter estimates for the mechanisms of increased viral clearance (red line), viral neutralization (orange line), target cell protection (green line), and increased antigen presentation (without increased viral clearance) (blue line), panels A)–H). For each macaque, a scatter plot of the ΔAIC and the log-likelihood for each mechanism, panels A)–H). The limit of detection is 50 SIV RNA copies/ml (thin horizontal dashed black line). Treatment with cART occurred between five weeks and 18/19 weeks post-infection (gray area), while eight infusions of the anti-α4β7antibody occurred between nine weeks post-infection and 32 weeks post-infection (purple area). The mechanisms considered include Parameters for these simulations are in Tables 1and S14–S18 and S2 Text.
(PDF)

S6 Fig Fit of model to the viral loads of the IgG control macaques using the baseline source model. The observed (black dots) and model predicted viral load dynamics (solid line) using the best-fit parameter estimates for A)–G) each of the macaques. Parameters for these simulations are in Tables 1 and S19.
(PDF)

S7 Fig Fit of model to the viral loads of the IgG control macaques using the saturated source model. The observed (black dots) and model predicted viral load dynamics (solid line) using the best-fit parameter estimates for A)–G) each of the macaques. Parameters for these simulations are in Tables 1and S19 and S2 Text.
(PDF)

S8 Fig Fit of model to the viral loads of the IgG control macaques using the antigen presenting cell source model. The observed (black dots) and model predicted viral load dynamics (solid line) using the best-fit estimates for A)–G) each of the macaques. Parameters for these simulations are in Tables 1 and S19.
(PDF)

S9 Fig The correlation between the half-saturation constant for effector cell proliferation and area under the predicted log_10_ viral load curve. The correlation between the best parameter estimates for the half-saturation constant for effector cell proliferation for the AIC selected mechanism of the baseline model for each treated macaque and the area under the predicted log_10_ viral load curve for the 30 weeks following A) the removal of cART and B) the last infusion of the anti-α4β7antibody.
(PDF)

S10 Fig Sensitivity of the viral load dynamics and area under the curve for the protection mechanism. The model predicted viral load (solid line) using the best-fit parameter estimates and the baseline source model for the treated macaques A) RSd14, B) ROv14, C) RLn12, and D) RFa15 using the baseline half-maximal concentration, EC_50_, (black), 100-fold higher EC_50_ (red), and 1000-fold higher EC_50_ (blue). The limit of detection is 50 SIV RNA copies/ml (thin dashed black line, left panels). E) The area under the log_10_ predicted viral load curve for the 30 weeks after cART was stopped for the treated macaques RSd14 (black), ROv14 (red), RLn12 (blue), and RFa15 (purple). Treatment with cART occurred between five weeks and 18/19 weeks post-infection (gray area). Parameters for these simulations are in Tables 1 and S16.
(PDF)

S11 Fig Sensitivity of the viral load dynamics and effector cell killing rate of the IgG control macaques predicted by the model. The average model predicted viral load (left panels) and the per day effector cell killing rate (right panels) using the best-fit parameter estimates under the baseline source model with the greatest AIC weight for each of the treated macaques (solid line). The sensitivity of the viral load and per day effector cell killing rate with respect to changing A)−B) the effectiveness of cART from 90% (black) to 99%(red) and 75% (blue); C)−D) the fraction of infections resulting in latency from 10^−5^ (black) to 10^−6^ (red) and 10^−4^ (blue); E)−F) the activation rate of latent cells from 10^−3^ (black) to 2 ×10^−3^ (red) and 5×10^−3^ (blue). The limit of detection is 50 SIV RNA copies/ml (thin dashed black line, left panels) and the minimum infected cell death rate is the death rate due to viral cytopathic effects (thin dashed line, right panels). Treatment with cART occurred between five weeks and 18/19 weeks post-infection (gray area). The average was calculated using the geometric mean for the seven IgG control macaques. Parameters for these simulations are in Tables 1 and S19.
(PDF)

S12 Fig Viral dynamics for *nef*-competent virus among the eight treated macaques. The model predicted viral loads for SIV *nef*-competent virus with anti-α4β7antibody therapy (black) and without anti-α4β7antibody therapy (red) using the best-fit parameter estimates and model variation with the greatest AIC weight for each of the treated macaques, panels A)–H). The limit of detection is 50 SIV RNA copies/ml (thin dashed black line). Treatment with cART occurred between five weeks and 18/19 weeks post-infection (gray area), while eight infusions of the anti-α4β7antibody occurred between nine weeks post-infection and 32 weeks post-infection (purple area). Parameters for these simulations are in Tables 1 and S14–S18, for the AIC selected model (Table 2). The effector cell killing rate (*m*) and the saturation constant for effector cell proliferation was adjusted for each treated macaque for the *nef*-competent virus (S13 Fig).
(PDF)

S13 Fig Viral rebound among the eight treated macaques for a *nef*-competent virus. The proportion of macaques whose viral load rebounded after the removal of cART (color gradient) for various reductions in the effector cell killing rate (*m*) and increases in the half-saturation constant for effector cell proliferation (*K*_*B*_). Parameters for these simulations are in Tables 1 and S14–S18, for the AIC selected model (Table 2). Viral rebound was characterized by the model viral load not dropping below 50 RNA copies/ml any time after week 40 post-infection and had a viral load that exceeded 10,000 RNA copies/ml at week 81-post infection.
(PDF)
