# Supplementary material for: Mechanistic basis of post-treatment control of SIV after anti-α4β7 antibody therapy
Source: PLoS Comput Biol. 2021 Jun 9;17(6):e1009031. doi: 10.1371/journal.pcbi.1009031 (PMC8189501; doi:10.1371/journal.pcbi.1009031)
Supplement: S1 Text — (DOCX) [file pcbi.1009031.s001.docx]

**S1 Text. Supplementary Methods: Mechanistic basis of post-treatment control of SIV after anti-α4β7 antibody therapy**

Chad R. Wells, Youfang Cao, David P. Durham, Siddappa N. Byrareddy, Aftab A. Ansari, Nancy H. Ruddle, Jeffrey P. Townsend, Alison P. Galvani and Alan S. Perelson

**Table of Contents**

Anti-𝛼4𝛽7 antibody dynamics 2

Quasi-steady state approximation for viral clearance and viral neutralization 3

Alternative effector source models 4

System of equations for the model of the anti-𝛼4𝛽7 antibody experiment 5

Scaling of the effector cell killing rate with the source rate of effector cells 6

Estimate of the initial viral concentration 6

Timeline of the anti-𝛼4𝛽7 antibody experiment 7

Fitting the model to the viral load data from individual macaques 7

Ranges of values for the IgG control macaque parameters 12

Ranges of values for the antibody mechanism parameters 13

Profile likelihood, confidence intervals, and sensitivity analysis 16

Modeling nef-competent virus 17

Tables 19

Table A: The parameter ranges used in the process of fitting the multiple mechanisms to the viral load data. 19

Figures 20

Fig A: The pharmacokinetic model fit to the data. 20

Fig B: A flow chart of the fitting process taken for an anti-𝛼4𝛽7 antibody treated macaque. 21

References 22

# **Anti-𝛼4𝛽7 antibody dynamics**

We model the anti-𝛼4𝛽7 antibody plasma concentration in the blood, denoted *A_B_*, and the anti-𝛼4𝛽7 antibody concentration in the tissue, denoted *A*_T_:

$dA_{B}/dt=A_{\alpha4\beta7}(t)-k_{12}A_{B}+k_{21}A_{T}\nu$, (S1)

$dA_{T}/dt=k_{12}A_{B}/\nu-(k_{21}+k_{0})A_{T}$. (S2)

In this model, the anti-𝛼4𝛽7 antibody is infused into the blood at rate $A_{\alpha4\beta7}(t)$, and disseminates to the tissue at rate *k*_12_. Since the tissue has a different volume than the blood, we adjust the concentration of antibody in the blood when entering the tissue by specifying the volume ratio $\nu$, which is the volume of the tissue divided by the volume of blood. Once in the tissue, the antibody either re-enters the blood at rate *k*_21_ (again scaled by $\nu$) or is eliminated at rate *k*_0_.

The volume ratio $\nu$ is not known because of the lack of information about the volume of distribution of the tissue compartment. For fitting and simulating the antibody concentration, we converted our system of equations to represent the total amount of antibody in the blood and tissue, where $X_{i}=v_{i}A_{i}$ is the total amount of antibody in region *i*, and *v_i_* is the volume of region *i*. Thus

$dX_{B}/dt=A_{\alpha4\beta7}(t)\nu_{B}-k_{12}X_{B}+k_{21}X_{T}$, and (S3)

$dX_{T}/dt=k_{12}X_{B}-(k_{21}+k_{0})X_{T}$, (S4)

where *v_B_* is the volume of blood and the volume of the tissue is arbitrary since we are only modeling the absolute amount of anti-𝛼4𝛽7 antibody in each compartment. The average blood volume of an adult macaque is 62.1 ml / kg [1]. Given the average weight of an adult female macaque is 5.3 kg [2], we set *v_B_* = 329 ml.

We fit our pharmacokinetic model to anti-𝛼4𝛽7 antibody concentration data from an *in vivo* SIV challenge study using female macaques [3]. The amount of antibody infused was 50 mg/kg [3] or 265 mg (or 2.65 ⨉ 10^5^ μg) for an average female macaque. Thus, $\int_{0}^{\tau} A_{\alpha4\beta7}(s)\nu_{B}ds=2.65\times10^{5}\mu g.$ We express the rate of infusion as $A_{\alpha4\beta7}(t)=\frac{2.65 \times10^{5}\mu g}{v_{B}\tau}$ for $t\in[0,\tau]$, otherwise $A_{\alpha4\beta7}(t)=0.$ Although the empirical data is measured in plasma, our concentration after infusion (from dividing by the volume of blood) is consistent with the concentrations of anti-𝛼4𝛽7 antibody measured in plasma and serum observed after infusion in other studies [4,5].

We assumed that the duration of the infusion was one hour, 𝜏 = 1/24 days. Infusions occurred on day 0, day 21, day 42, and day 56 in the SIV challenge study [3].

To estimate the model parameters, we maximize the log-likelihood

$L=\sum_{j=1}^{12} \sum_{i=1}^{n} ln\left( f({ln(B}_{i,j})|ln(X_{B,j}(t_{i})/329),(\sigma_{A})^{2}) \right)$, (S5)

where *B_i,j_* is the *i^th^* measurement of the antibody concentration in the *j^th^* macaque at time point *t_i_* , *n* is the number of data points, and $\sigma_{A}$is the standard deviation. The function *f*(*x*|𝜇,𝜎^2^) is the normal distribution with mean 𝜇 and variance 𝜎^2^. To obtain the value of our standard deviation in our likelihood, we calculated the average standard deviation among the 12 macaques at the 11 measurements at times consistent among all macaques (${ln(B}_{i,j})$at weeks one to nine, and weeks 11 and 12): $\sigma_{A}=0.24.$

We maximized the log-likelihood with respect to *k*_0_ and *k*_12_ using fixed values of *k*_21_, that varied at 0.001per day intervals within the range of [0.001,0.01]. We then selected the three values of *k*_0_, *k*_12_, and *k*_21_ that yielded the highest likelihood (Table 1 and Fig A).

# **Quasi-steady state approximation for viral clearance and viral neutralization**

We assumed that the kinetics between antibody and virus were much faster than the decline in viral load, such that we could apply a quasi-steady state approximation. We denote the binding rate of the antibody to the virus as *k_on_* and the dissociation rate as *k_off_*. Thus

$dV_{N}/dt=pI{-k}_{on}V_{N}A_{B}-cV_{N}+k_{off}V_{C}$, and (S6)

$dV_{C}/dt=k_{on}V_{N}A_{B}-c(1+(\gamma-1))V_{C}-k_{off}V_{C}$, (S7)

where *V_N_* is the virus not in complex with the antibody, *V_C_* is the virus in complex with the antibody, and $\gamma$ is the fold increase of the clearance rate for the complex virus. Under the quasi-steady state approximation,

$k_{on}V_{N}A_{B}-c(1+(\gamma-1))V_{C}-k_{off}V_{C}=0\Rightarrow V_{C}=\left( \frac{k_{on}}{k_{off}+c(1+(\gamma-1))} \right)A_{B}V_{N}$. (S8)

Let $V=V_{N}+V_{C}$ be the total virus and $\psi=\left( \frac{k_{on}}{k_{off}+c(1+(\gamma-1))} \right)$ be the parameter that describes the binding kinetics between the antibody and the virus. Then $V_{N}=V\frac{1}{(1+\psi A_{B})}$ and $V_{C}=V\left( \frac{\psi A_{B}}{1+\psi A_{B}} \right)$. Thus, for the clearance mechanism, the differential equation for the total viral concentration is

$dV/dt=pI-cV-c(\gamma-1)V_{C}=pI-cV-c(\gamma-1)\left( \frac{\psi A_{B}}{1+\psi A_{B}} \right)V=pI-c(1+(\gamma-1)\left( \frac{\psi A_{B}}{1+\psi A_{B}} \right))V$. (S9)

Virus in antigen-antibody complex, *V_C_*, is assumed to be neutralized. Thus, the infection rate for the total viral concentration under the neutralization mechanism is

$\beta(V-V_{C})=\beta V(1-\left( \frac{\psi A_{B}}{1+\psi A_{B}} \right))=\frac{\beta V}{1+\psi A_{B}}$. (S10)

# **Alternative effector source models**

We examined two additional models for the source of effector cells. The first uses a saturated source (SS) model, where in addition to the possible effect of anti-𝛼4𝛽7 antibody on increasing antigen presentation, in which case the indicator variable $\mathbb{I}_{A}=1,$ the production of effector cells is dependent on the concentration of productively infected cells that interact with precursors that we assume are present at excess and maintain a roughly constant level. In the SS model, Eq. (S19), effector cells are generated at rate $\lambda_{E}(1+\mathbb{I}_{A}\frac{A_{B}}{A_{B}+\Omega})I / (I+K_{P}),$ where *K_P_* is the half-saturation constant for producing cytotoxic effector cells, the antibody term is the same as in the baseline model, Eq. (S15) and $\lambda_{E}$ is the maximum source rate of cytotoxic effector cells .

The second approach explicitly considers antigen presenting cells (APC) being activated by antigen and then stimulating effector cells. A model of this process was introduced by Desikan et al. [6]. We use this model and call it the antigen-dependent source (APCS) model. In this model, the activated APCs, *D*, obey Eq. (S20) taken from Desikan et al. [6]. In the effector cell equation, new effectors are generated from a pool of precursors at rate $\lambda_{E}D$(Eq. S21), where the precursor pool is implicitly assumed to be constant and incorporated into the rate constant $\lambda_{E}.$

# **System of equations for the model of the anti-𝛼4𝛽7 antibody experiment**

The system of equations that encompass all the mechanisms of the antibody are

$$dT/dt=r_{T}T\left( 1-\frac{T+I+L+P}{\kappa} \right)-d_{T}T-\left( 1-\varepsilon\right)\beta TV\left( 1-\mathbb{I}_{N}\frac{\psi A_{B}}{1+\psi A_{B}} \right)$$

$-\rho\frac{A_{B}}{A_{1}+EC_{50}}T\left( \kappa_{\alpha4\beta7}-\frac{P}{P+T+I+L} \right)+\omega\frac{EC_{50}}{A_{B}+EC_{50}}P$ (S11)

$dI/dt=(1-f)(1-\varepsilon)\beta TV\left( 1-\mathbb{I}_{N}\frac{\psi A_{B}}{1+\psi A_{B}} \right)+\alpha L-I(\delta+mE)$, (S12)

$dL/dt=f(1-\varepsilon)\beta TV\left( 1-\mathbb{I}_{N}\frac{\psi A_{B}}{1+\psi A_{B}} \right)-\alpha L-d_{L}L+r_{L}L$, (S13)

$dV/dt=pI-cV\left( 1+(\gamma-1)\frac{\psi A_{B}}{1+\psi A_{B}} \right)$, (S14)

$dE/dt=\lambda_{E}(1+\mathbb{I}_{A}\frac{A_{B}}{A_{B}+\Omega})-\mu E+b_{E}E\frac{I}{I+K_{B}}-d_{E}E\frac{I}{I+K_{D}}$, (S15)

$dP/dt=r_{T}P\left( 1-\frac{T+I+L+P}{\kappa} \right)+\rho\frac{A_{B}}{A_{B}+EC_{50}}T\left( \kappa_{\alpha4\beta7}-\frac{P}{P+T+I+L} \right) -d_{T}P-\omega\frac{EC_{50}}{A_{B}+EC_{50}}P$, (S16)

$dX_{B}/dt=A_{\alpha4\beta7}(t)\nu_{B}-k_{12}X_{B}+k_{21}X_{T}$, and (S17)

$dX_{T}/dt=k_{12}X_{B}-(k_{21}+k_{0})X_{T}$, (S18)

where *A_B_* = *X_B_* / 329 ml is the concentration of anti-𝛼4𝛽7 antibody in the peripheral blood.

The effector cell concentration for a saturated source is

$dE/dt=\lambda_{E}(1+\mathbb{I}_{A}\frac{A_{B}}{A_{B}+\Omega})\frac{I}{I+K_{P}}-\mu E+b_{E}E\frac{I}{I+K_{B}}-d_{E}E\frac{I}{I+K_{D}}$. (S19)

For the antigen presenting cell source model, the equation for activated antigen presenting cells, *D*, is

$dD/dt=b_{D}I+\mathbb{I}_{A}\Omega\frac{\psi_{A}A_{B}}{1+\psi_{A}A_{B}}V-d_{D}D,$ (S20)

where ${1/\psi}_{A}$ is the antibody concentration of half-maximal effect for increased antigen presentation for the APCS model. To limit the number of free parameters, we assumed that *ψ_A_* = *ψ_C_* when increased viral clearance was included to the increased antigen presentation mechanism.

The effector cell concentration for the APCS model is represented by

$dE/dt=\lambda_{E}D-\mu E+b_{E}E\frac{I}{I+K_{B}}-d_{E}E\frac{I}{I+K_{D}}$. (S21)

# **Scaling of the effector cell killing rate with the source rate of effector cells**

The estimated effector cell killing rate (*m*) scales with the source rate of effector cells (λ_E_). Let *E* = mE* then $dE^{*}/dt=m dE/dt={m\lambda}_{E}-\mu E^{*}+b_{E}E^{*}\frac{I}{I+K_{B}}-d_{E}E^{*}\frac{I}{I+K_{D}}.$ This scaling holds for the other two effector cell source models as well. We also have the estimated effector cell killing rate (*m*) scaling with the rate antigen presenting cell encounter antigen (*b_D_*) as well as the removal rate of antigen presenting cells (*d_D_*). Let *D** = *b_D_I*/*d_D_*, which is the SIV specific antigen presenting cell concentration under a quasi-steady state assumption. Thus,

$$dE^{*}/dt=m dE/dt\approx{m\lambda}_{E}b_{D}I /d_{D}-\mu E^{*}+b_{E}E^{*}\frac{I}{I+K_{B}}-d_{E}E^{*}\frac{I}{I+K_{D}}.$$

# **Estimate of the initial viral concentration**

The macaques were infected intravenously with 200 TCID_50_ of SIVmac239. To estimate the initial number of RNA copies/ml, we used the conversion factor from an alternative study where a challenge of 5 ⨉ 10^5^ TCID_50_/ml was equivalent to 3.4 ⨉ 10^8^ copies/ml for SIVmac239 [7], yielding a conversion factor of 680 RNA copies/TCID_50_. To be consistent with our pharmacokinetics model we again assume 329 ml of blood. Thus, we estimated that the initial viral concentration was 413 SIV RNA copies/ml (680 copies/TCID_50_ ⨉ (200 TCID_50_/329 ml)).

# **Timeline of the anti-𝛼4𝛽7 antibody experiment**

The anti-𝛼4𝛽7 antibody experiment is broken up into five different phases [8]. In phase I (weeks 0–5), the macaques are infected at week zero with SIVmac239 and acute infection proceeded for five weeks. Phase II (weeks 5–9), the macaques are given daily cART. After four weeks of cART, phase III begins (weeks 9–18), during which the macaques are given daily cART and injected with infusions of an anti-𝛼4𝛽7 antibody approximately every three weeks. Phase IV (weeks 18–32) macaques were no longer given the daily cART but continue receiving infusions of the anti-𝛼4𝛽7 antibody. Specifically, cART was stopped at week 18 post infection in one group of macaques and at week 19 post infection in the other group. During phases III–IV, the macaques receive a total of eight infusions of the anti-𝛼4𝛽7 antibody. The final phase (after 32 weeks), the infusions of the anti-𝛼4𝛽7 antibody are stopped and the macaques no longer receive any treatment.

# **Fitting the model to the viral load data from individual macaques**

We used a maximum likelihood approach to estimate the parameters $r_{T}, m, p, K_{B}, \omega,\rho, \psi, \gamma, \Omega,K_{p}$ and $\sigma$(S14–S19 Tables). Fitting the specified model with the corresponding parameters to the data on viral load, we maximized the log-likelihood

$L_{A}=\left[ \max_{j=\{1,\cdots,7\}}\sum_{i=1}^{4} ln(C_{i}) \right]+ln\left( C_{5} \right)+ln\left( C_{6} \right)+ ln \left( C_{7} \right)+\sum_{i=1}^{3} ln\left( F_{i} \right),$ (S22)

where the *C_i_* and *F_i_* are defined by Eqs. (S24-S31).

Among the seven IgG control macaques, there was little variability in their viral dynamics, thus, we speculate that the viral dynamics of the eight treated macaques would resemble those of an IgG control macaque in the absence of receiving the anti-α4β7 antibody. Because of the variability in the viral dynamics among the treated macaques after the removal of cART, our approach for using the viral load data from the control macaques to inform the model is motivated from methods used in population science to identify heterogeneous treatment effects [9]. In our approach, we pair each treated macaque with an IgG control macaque such that the viral load of the model in the absence of the anti-𝛼4𝛽7 antibody maximized the likelihood of the viral dynamics of the control macaque. We only use the viral load dynamics after removal of cART (phase IV and phase V) from the IgG control macaque as explained below. For each of the seven IgG control macaques, we compute macaque specific log-likelihood

$L_{j}=\sum_{i=1}^{4} ln (C_{i}) ,$ (S23)

where *L_j_* is the log-likelihood for IgG control macaque *j.* We note that for two of the IgG control macaques there is an extra viral load measurement after the removal of cART compared to the other five IgG control macaques.

Fitting to the pairing of viral dynamics observed in an anti-𝛼4𝛽7 antibody treated macaque and an IgG control macaque (Fig B), allows for better inference of the parameters not impacted by the anti- 𝛼4𝛽7antibody. We assume that the viral load dynamics observed in the seven IgG control macaques is representative sample of the population. Thus, the viral dynamics post-cART for one of the seven IgG control macaques would best represent the viral dynamics post-cART of an anti- 𝛼4𝛽7antibody treated macaque in a simulation if they had not received the anti- 𝛼4𝛽7antibody infusions. The individual pairing provides some level of variability in the possible dynamics for a treated macaque, where fitting to the dynamics of all seven IgG control macaques eliminates individual level heterogeneity as the post-cART dynamics would be representative of a population average.

For the uncensored viral load data points (empirical viral load above the detection limit of 50 SIV RNA copies/ml) we used:

$C_{1}=\prod_{i=1}^{n_{U}} exp\left\{ -\frac{\left( {log}_{10}(V_{j,i})-{log}_{10}(V(t_{i},\vec{x})) \right)^{2}}{2\sigma^{2}} \right\}/\sqrt{2\pi\sigma^{2}}$ (S24)

where $V(t_{i},\vec{x})$is the model predicted viral load at time *t_i_*, *n_U_* is the number of uncensored data points for the IgG control macaque, and *V_i_* is the *i*^th^ uncensored data point for the IgG control macaque.

For evaluating the likelihood of the censored viral load data points (empirical viral load below the detection limit of 50 SIV RNA copies / ml) we used:

$C_{2}=\prod_{i=1}^{n_{C}} \left[ \Phi\left( \frac{\left( {log}_{10}(50)-{log}_{10}(V(t_{i},\vec{x})) \right)}{\sigma} \right)-\Phi\left( \frac{\left( {log}_{10}(\lambda_{j,i})-{log}_{10}(V(t_{i},\vec{x})) \right)}{\sigma} \right) \right]$, (S25)

where the limit of detection is 50 RNA copies / ml and $\lambda_{j,i}$RNA copies / ml is the lower bound for the viral load for the *i*^th^ censored data point for the IgG control macaque [10]. The viral set-point for individuals on standard cART during chronic infection is of the order of 1 RNA copy/ml [11–14]. However, since the macaques started cART early on in acute infection it is plausible the latent reservoir may be too small for the viremia level to go above 1 RNA copy/ml when cART is administered during phase II and III —compared to the size of the reservoir in chronic infection when patients on cART have viral loads of the order of 1 RNA copy/ml [11–14]. It is uncertain as to what the magnitude of the viral load is during early infection while on cART. Thus, we chose a lower bound of 0 SIV RNA copies / ml for the period the macaques are on cART (phases II and III). The median viral load under treatment with the recommended cART regime is above 1 SIV RNA copy/ml [11–14]. Using conventional cART as the benchmark for the suppression of viremia by the anti-𝛼4𝛽7 antibody and the immune response, we used a lower bound of 1 SIV RNA copy/ml for the remaining phases (I, IV and V). We do not use the lower bound of zero because there is ongoing viral replication after the removal of cART (Phase IV and V).

The IgG control macaques did not achieve viral suppression beyond 50 weeks post-infection [8], indicating they approached a viral load equilibrium above the limit of detection. To ensure that the viral dynamics in the IgG control setting does not achieve viral suppression after 50 weeks post-infection once cART is removed, we used

$C_{3}=exp\left\{ -\frac{\left( {log}_{10}(V_{C}(567,\vec{x}))-\mu_{S} \right)^{2}}{2{\sigma_{S}}^{2}} \right\}/\sqrt{2\pi{\sigma_{S}}^{2}}$ (S26)

where *V*(567) is the viral load predicted by the model at week 81 p.i. (day 567) , *𝜇_S_* is the mean log_10_ viral concentration for the IgG control macaque for the empirical measurements taken from 40 weeks p.i. onward., and σ_S_ is the standard deviation of the log_10_ viral concentration for the IgG control macaque for the empirical measurements taken from 40 weeks p.i. onward. We used the viral load data from 40 weeks p.i. onward as an approximation to the viral set-point at 81 weeks p.i. in the empirical data, which is the last time point of macaque monitoring [8].

Control macaques in other challenge studies, where cART was not administered, did not suppress the virus [3,4,15]. To account for no suppression in the absence of any treatment in our model, we used

$C_{4}=exp\left\{ -\frac{\left( {log}_{10}(V_{0}(567,\vec{x}))-\mu_{S} \right)^{2}}{2{\sigma_{S}}^{2}} \right\}/\sqrt{2\pi{\sigma_{S}}^{2}}$ (S27)

where $V_{0}(567,\vec{x})$ is the viral load when there is no cART or antibody treatment at 81 weeks post-infection. Here we also used the viral concentrations from 40-weeks p.i onward in the IgG control macaque to approximate the viral set-point at 81 weeks post-infection for macaques not given cART or the anti-𝛼4𝛽7 antibody.

None of the treated macaques had detectable viremia after 50 weeks post-infection [8], suggesting they approached a viral equilibrium below 50 SIV RNA copies/ml. Thus, we used a conditional likelihood where if the equilibrium solution of the model for the minimum viral set-point is 50 SIV RNA copies / ml or greater, then *C*_5_ = 0, otherwise

$C_{5}=\left[ \Phi\left( \frac{\left( {log}_{10}(50)-{log}_{10}(\underline{V} \right)}{\sigma} \right)-\Phi\left( \frac{\left( {log}_{10}(1)-{log}_{10}(\underline{V} \right)}{\sigma} \right) \right],$ (S28)

where $\underline{V}$ denotes the minimum viral set-point below 50 SIV RNA copies / ml predicted by the equilibrium solution of the model. In addition, none of the IgG control macaques achieved viral suppression, suggesting they approached a viral equilibrium above 50 SIV RNA copies/ml. Thus, if the maximum viral set-point is below 50 SIV RNA copies/ml then *C*_6_ = 0, otherwise *C*_6_ = 1.

The effector cell killing rate (*m*) and the infected cell death rate (𝛿) in the model can be informed by the estimated overall infected cell death rates *in vivo*, which range between 0.5/day to 1.5/day [16,17]. The likelihood function for the infected cell death rate is *C_7_* = 1 if $m\bar{E}+\delta_{v}\in[0.5,1.5]$, where $\bar{E}$ is the equilibrium value for the cytotoxic effector cells predicted by the equilibrium solution of the model for an IgG control macaque (i.e., the equilibrium solution of the model for the maximum viral load). Otherwise, if $m\bar{E}+\delta_{v}$>1.5 or $m\bar{E}+\delta_{v}<0.5$ then *C_7_ =* 0.

For the viral dynamics observed in the macaques treated with the anti-𝛼_4_𝛽_7_ antibody, the likelihood of the uncensored data

$F_{1}=\prod_{i=1}^{n_{U}} exp\left\{ -\frac{\left( {log}_{10}(V_{j,i})-{log}_{10}(V(t_{j,i},\vec{x})) \right)^{2}}{2\sigma^{2}} \right\}/\sqrt{2\pi\sigma^{2}}$ (S29)

and censored data

$F_{2}=\prod_{i=1}^{n_{D}} \left[ \Phi\left( \frac{\left( {log}_{10}(50)-{log}_{10}(V(t_{i},\vec{x})) \right)}{\sigma} \right)-\Phi\left( \frac{\left( {log}_{10}(\lambda)-{log}_{10}(V(t_{i},\vec{x})) \right)}{\sigma} \right) \right].$ (S30)

In addition, we know that the virus remained suppressed up to 81 weeks p.i. in all the treated macaques. We examine the likelihood that the predicted viral load at week 81 p.i. is between 1 SIV RNA copy/ml and 50 SIV RNA copies ml

$F_{3}=\left[ \Phi\left( \frac{\left( {log}_{10}(50)-{log}_{10}(V(567,\vec{x})) \right)}{\sigma} \right)-\Phi\left( \frac{\left( {log}_{10}(1)-{log}_{10}(V(567,\vec{x})) \right)}{\sigma} \right) \right]$, (S31)

where $V(567,\vec{x})$is the model predicted viral load at 81 weeks p.i..

The likelihood for the IgG control macaques consists of *C_1_*, *C_2_*, *C_3_*, *C_4_*, *C_5_*, *C_6_,* and *C_7_* and requires the viral load data from only the control macaque, unlike the anti-𝛼_4_𝛽_7_ antibody treated macaques that require both the viral load data of an IgG control macaque and a treated macaque.

# **Ranges of values for the IgG control macaque parameters**

For the range of the maximum proliferation rate of target cells, we used estimates from a BrdU labeling study [18]. For a lower bound estimate of the proliferation rate, we used the mean proliferation rate of 0.001 per day estimated from uninfected macaques [18]. However, we have based our target cell population on Ki67^+^ CD4^+^ T cells and these estimates include both Ki67^+^ CD4^+^ T cells and Ki67^-^ CD4^+^ T cells. In an HIV uninfected individual approximately 1.1% of CD4^+^ T cells are Ki67^+^  [19]. Assuming Ki67^-^ CD4^+^ T cells do not proliferate, we estimate the lower bound for the maximum proliferation rate to be 0.09/day (0.001/0.011 per day). We used the mean proliferation rate from highly viremic SIV infected macaques (0.031/day) to estimate an upper bound. In the BrdU study, the average CD4^+^ T-cell count in these highly viremic macaques was 468 cells/ul. Thus, we used the percentage of Ki67^+^ CD4^+^ T cells for a CD4 count between 200 to 500 cells/ul (5.6%). Our estimated upper bound for the maximum proliferation rate is 0.55 per day (0.031/0.056 per day).

One experiment estimates the viral production rate to be of the order of 10^3^ [20], while another experiment estimates an average viral production rate of approximately 5 × 10^4^ [21]. Thus, our initial range for the viral production rate to be within 10^3^ and 5 × 10^4^ SIV RNA copies per cell/day and later was calibrated to 4 × 10^3^ and 1.5 × 10^4^ SIV RNA copies per cell/day after exploratory fitting. For the lower bound of the proliferation half-saturation constant, we assumed a value of 0.001 cells/ml and an upper bound of 10 cells/ml. We also calibrated the ranges of the parameters *m* and 𝜎, based on the IgG control viral dynamics.

We conducted a primary grid search for the infected cell death rate due to viral cytopathic effects and the maximum rate of effector cell exhaustion for the seven IgG control macaques. We specified the range for the infected cell death rate to be 0.05/day to 0.75/day and the maximum rate of exhaustion to be between 1/day and 6/day, increasing each at 0.05 increments. The combination of these two parameters that provided the greatest overall likelihood were selected as the baseline values in the fitting of the anti-𝛼4𝛽7 treated macaques and the model simulations.

# **Ranges of values for the antibody mechanism parameters**

The rate at which CD4^+^ T cells lose protection from the protection mechanism (⍵) can be bounded at the lower end by the rate of 𝛼4𝛽7 re-expression on the surface of lymphocytes [22]. After one day of internalizing 𝛼4𝛽7, 54% of the lymphocytes were expressing 𝛼4𝛽7 (or 46% were still not expressing 𝛼4𝛽7), while after four days, 83% of the lymphocytes were expressing 𝛼4𝛽7 (or 17% were still not expressing 𝛼4𝛽7). These percentages correspond to exponential loss of protection of 0.44/day and 0.78/day, respectively. To specify the lower bound for the rate at which CD4^+^ T cells lose protection (⍵), we therefore used 0.44/day. We set the upper bound for the rate at which CD4^+^ T cells lose protection based on the amount that the expression of 𝛼4𝛽7 can be induced by retinoic acid under the condition where the immune response is skewed towards the production of Th1 cells [23]. Retinoic acid can increase expression of 𝛼4𝛽7 approximately 100-fold compared to the control setting. This increase in expression would suggest an exponential loss rate of 78/day for protection, based on the estimated maximum loss rate *in vitro*. We thus chose the upper bound for the rate at which CD4^+^ T cells lose protection to be 78/day.

For the maximum rate at which a target cell enters the protected state, we use estimates for the time required for 𝛼4𝛽7 to be internalized that found 50% of 𝛼4𝛽7 receptors were internalized within 3.5 hours [22]. Thus, we estimate an upper bound of 4.75/day for the maximum rate in which target cells become protected. For the concentration of the half-maximal effect in the protection mechanism, we use the EC_50_ value from an *in-vitro* study that quantified Vedolizumab ability to bind to rhesus monkey memory helper T lymphocytes (27.6 ng/ml = 2.76 ⨉ 10^-2^ 𝜇g / ml) [24].

We conservatively assumed a lower bound of 10^-9^ ml/µg for the inverse half maximal effective concentration of the antibody (*ψ*)for enhanced viral clearance. This lower bound at an antibody concentration of 805 µg/ml and viral load of 10^5^ RNA copies/ml indicates ~0.08 RNA copies/ml of virus would be affected by clearance or neutralization. This assumption is consistent with the concentration of 𝛼4𝛽7^+^ virus observed at 21 weeks p.i. being substantially below 1000 copies/ml, with the overall concentration of virus being approximately 10^5^ RNA copies/ml [8,25]. The highest number of cells expressing 𝛼4𝛽7 occurs during the early stages of acute infection, providing the best opportunity for 𝛼4𝛽7 to be incorporated into the membrane of SIV. The maximum concentration of 𝛼4𝛽7^+^ virus was roughly 450,00 copies/ml, with the viral peak at roughly 10^7^ RNA copies/ml [8,25]. This concentration of virus would suggest that 0.45% of virus at the viral peak would be 𝛼4𝛽7^+^. However, the macaques treated with the anti-𝛼4𝛽7 antibody exhibited increased levels of retinoic acid compared to the IgG control macaques. This increase in retinoic acid would increase the expression of 𝛼4𝛽7 on CD4^+^ T cells. Since 𝛼4𝛽7 is incorporated into SIV from the host cell [25], the increased expression of 𝛼4𝛽7 on CD4^+^ T cells would directly increase the amount of expression on virus; thus we assume the amount of 𝛼4𝛽7 expression would increase 100-fold (comparable to the increase in expression on lymphocytes [23]). We assume that this 100-fold increase in 𝛼4𝛽7 expression due to retinoic acid leads to linear increases the concentration of 𝛼4𝛽7^+^ virus in the treated macaques, suggesting 45% of viruses expresses 𝛼4𝛽7. Using the initial concentration of the antibody after the first infusion (805 µg/ml), we estimate the inverse half-maximal effective concentration of the antibody to be 10^-3^ ml/µg such that *ψA_b_* / (*ψA_b_* + 1) ≈0.45. Thus, the inverse half- half maximal effective concentration of the antibody for viral clearance was specified as being within the range of 10^-9^ ml/µg to 10^-3^ ml/µg.

To determine an upper bound for the fold change in viral clearance in the enhanced viral clearance mechanism, we utilized estimates of the viral half-life of HIV-1 in naive rhesus macaques and that estimated for rhesus macaques with high titers of neutralizing antibodies [26]. The longest half-life for the naive macaques was 19.3 minutes and the shortest half-life under high titers of neutralizing antibodies was 3.9 minutes. Using these half-lives, we specify an upper bound of 5 ((ln(2)/3.9)/(ln(2)/19.3)=4.95) for the fold-change on the viral clearance rate in the presence of antibodies. For a lower bound, we assume that the antibody would not impact the baseline viral clearance rate.

Mature dendritic cells that were exposed to an anti-𝛽7 antibody before encountering HIV *in vitro* and then incubated with CD8+ T cells exhibited an increase of up to approximately 100% in interferon-𝛾 spot forming CD8^+^ T cells compared to mature dendritic cells in a control setting [27]. Thus, we assume the antibody can at most increase the effector source rate by 100%. For the half-saturation constant of this mechanism, we chose an upper bound for Ω of 10^6^ for the BL and SS model, which corresponds to no effect, and a lower bound of 10^-8^ which prolongs the impact of the mechanism. For the APC model that does include saturation of the antibody effect, we assumed an upper bound for Ω of 40.

# **Profile likelihood, confidence intervals, and sensitivity analysis**

We constructed profile likelihoods to examine the robustness of our model to changes in the estimated parameters and conducted sensitivity analysis on the fixed parameters (S3 Text). If the initial optimization algorithm failed to converge to the optimum, i.e., a better solution was found during the construction of the profile likelihood, we updated the optimal solution for the macaque. We found calculating the profile likelihood through conventional re-optimization at a granular scale was computationally intensive for our specified objective function. To obtain an approximation of the profile likelihood, we utilized the likelihood values obtained from MATLAB’s global optimization solver *surrogateop* [28]. Constructing the profile likelihood for a specified parameter, we provided pre-determined bins for the value of the parameter to fall into and take on during the optimization process. We ran the global optimization for 10^4^ iterations, using all recorded likelihood values. After the optimization was complete, we used the maximum likelihood value (minimum negative log-likelihood) for each bin to approximate the profile likelihood. We supplemented the estimates for each bin from the profile likelihood analysis of other parameters to help improve our approximation of the profile likelihood. To provide a continuous profile, polynomial functions were fit to both the raw log_10_ of the negative log-likelihood and smoothed log_10_ of the negative log-likelihoods to the left and the right of the maximum likelihood estimate. The order of the polynomial was determined through AIC selection using the residual sum of squares. We found that large negative log-likelihood values dramatically influenced the behavior of the polynomial in the area of interest. To decrease their influence on the polynomial, values exceeding three times the 95% percentile for the 𝜒^2^ distribution for $k$ degrees of freedom were set to this threshold, where *k* is the number of free parameters in the model. These approximations to the profile likelihood are also used to estimate the confidence intervals for the estimated parameters, which is where the difference in the change in the log-likelihood is half the 95% percentile for the 𝜒^2^ distribution for $k$ degrees of freedom [29]. For the construction of the confidence intervals, we use the raw likelihoods from the optimization process to estimate the maximum range in which the parameter falls below the specified threshold.

We use a similar approach to determine the sensitivity of the likelihood to changes in the fixed parameter values of the frequency of infections resulting in latency, the latent cell activation rate, the efficacy of cART, the viral inoculum, the percentage of CD4+ T cells that would be classified as target cells, the maximum rate of effector cell proliferation, the ratio of the half-saturation constants for effector cell proliferation and exhaustion, and the source rate of effector cells. Specifically, we vary the parameter of interest and keep all other parameters fixed (i.e., the model is not re-optimized).

# **Modeling nef-competent virus**

*Nef-*competent virus downregulates major histocompatibility complex (MHC) class I molecules on the surface of infected cells, making them poor targets for effector cells [30,31]. In addition, we speculate that due to its downregulation MHC-I will not stimulate the expansion of the effector response. Based on these characteristics, we decreased the best estimated effector cell killing rate (*m*) and increased the half-saturation constant for effector cell expansion (*K_B_*) for each of the eight treated macaques, while keeping all other parameters fixed (i.e. the half-saturation constant for effector cell exhaustion (*K_D_*) does not increase). For macaque *i*, we denote the decrease in the effector cell killing rate by Δ*m_i_* and the increase in the half-saturation constant for effector cell expansion by Δ*K_i_*.

For each macaque, we conducted a grid search of these two parameters between 0% and 95% (at 2.5% intervals) for the killing rate and 0 to 29 factor increase (i.e., 1 to 30-fold increase) (at 0.5 intervals) for the half-saturation constant. The upper bound for the fold-increase was calibrated to obtain roughly a maximum value of 20 cells/ml after the adjustment, twice the assumed upper bound in fitting. Based on the AIC selected mechanism for the constant source model, the maximum half-saturation constant after maximal adjustment was 20.63 cell/ml. For each parameter combination for macaque *k*, viral rebound for *nef*-competent virus in the treated macaques was classified as the viral load not falling below 50 RNA copies/ml after 40 weeks post-infection and exceeding 10,000 RNA copies/ml at week 81 post-infection, based on the observed viral setpoint for SIVmac251 in Abbink et al. [5]. We denote this classification of viral rebound by *L_R_* = 1, otherwise *L_R_* = 0. To identify the minimum perturbation to these two parameters that will produce viral rebound, we first scale the change to the half-saturation constant

$\tilde{\Delta K}=\Delta K/29.5$,

such that the effect is not 100% at the boundary value of 29 of the perturbation. After this scaling, we choose the change in the killing rate *m* and half-saturation constant *K_B_* for each individual macaque by maximizing $L_{R}\left( 1-\tilde{\Delta K} \right)\left( 1-\Delta m \right).$

# Tables

## Table A: The parameter ranges used in the process of fitting the multiple mechanisms to the viral load data.

| Parameter | Description | Range | Units | Reference |
| --- | --- | --- | --- | --- |
| *r*_T_ | Maximum proliferation of target cells | (0.09, 0.55) | per day | [18] |
| *m* | Rate cytotoxic effector cells kill infected cells for BL and SS model | (10^﹣8^, 10^﹣2^) | per effector cell/ml per day | Assumed |
| *m* | Rate cytotoxic effector cells kill infected cells for APCS model | (10^﹣12^, 10^﹣6^) | per effector cell/ml per day | Assumed |
| *K*_B_ | Proliferation half-saturation constant | (0.001, 10) | cells/ml | Assumed |
| *K*_P_ | Half-saturation constant for producing cytotoxic effector cells for the SS model | (10^﹣5^, 10^﹣1^) | cells/ml | Assumed |
| *p* | Infected cell viral production rate | (4000, 15000) | SIV RNA copies/ cell per day | Calibrated |
| 𝜎 | Standard deviation for viral load | (0.01, 2) | log_10_ SIV RNA copies / ml | Assumed |
| ⍵ | Rate protection wanes | (0.44, 78) | per day | [22] |
| 𝜓 | Binding kinetics of the antibody to the virus | (10^﹣9^, 10^﹣3^) | ml/𝜇g | Estimated |
| Ω | Half-saturation constant for antigen presentation mechanism in the BL and SS model | (10^﹣8^,10^6^) | ml/𝜇g | Estimated |
| Ω | Increase in antigen presentation for APS model | (0, 40) | ml/𝜇g | Assumed |
| 𝜌 | Rate CD4+ T cells become protected | (0, 4.75) |  | [4,22] |
| 𝛾 | Fold increase of viral clearance | (1, 5) |  | [26] |
|  | | | | |
|  |  |  |  |  |

# Figures


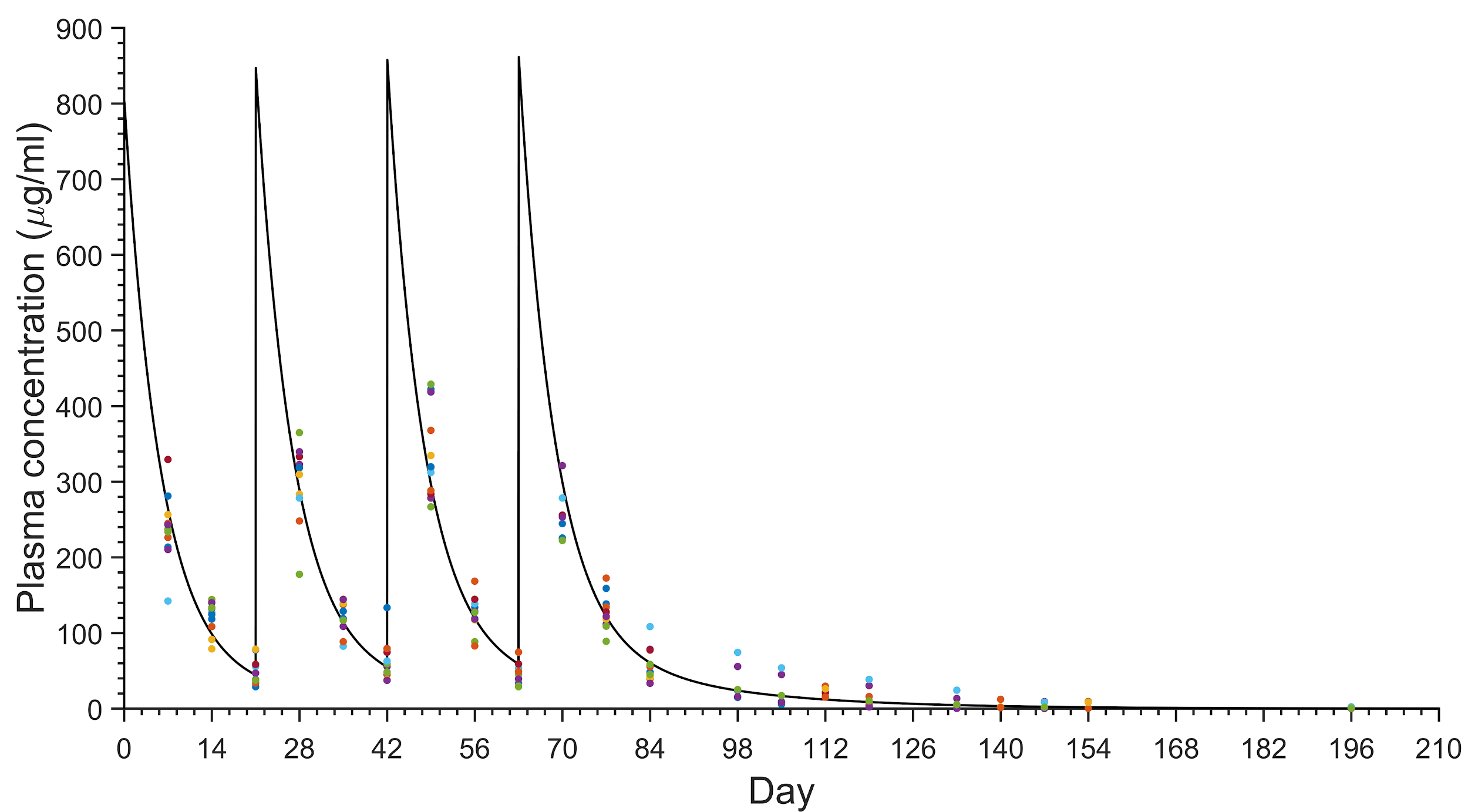


## Fig A: The pharmacokinetic model fit to the data.

The best-fit antibody concentration profile, *A_B_(t)*, in the peripheral blood from our two-compartment pharmacokinetic model with parameters described in Table 1. The black line denotes the best fit, while the colored dots denote the antibody plasma concentration for the individual macaques from [3].


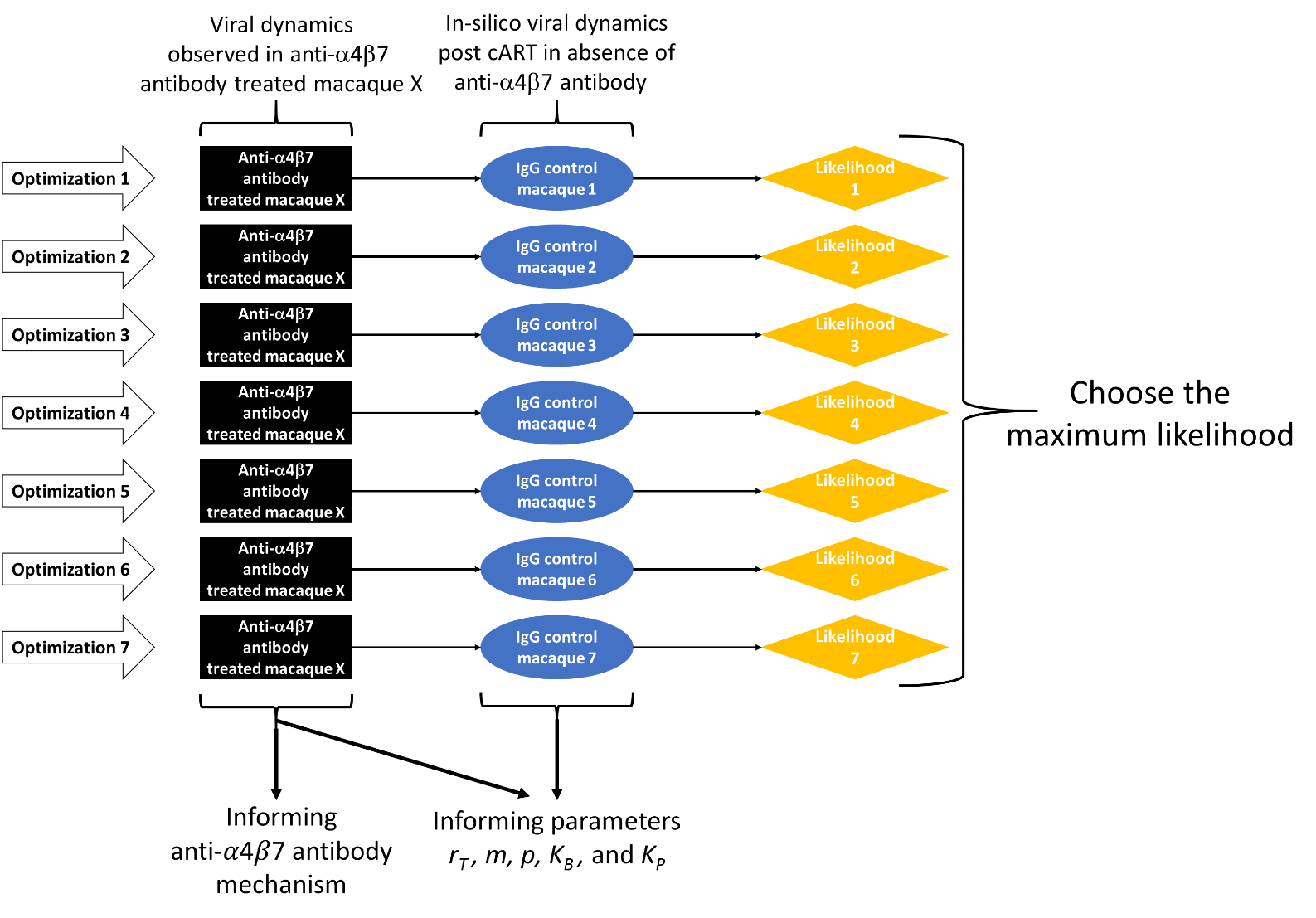


## Fig B: A flow chart of the fitting process taken for an anti-𝛼4𝛽7 antibody treated macaque.

For a single treated macaque for a specified mechanism there are seven independent log-likelihood optimizations. The optimizations account for the viral dynamics of the treated macaque (black box) as well as one of the IgG control macaques (blue circle), providing the overall likelihood that is maximized (yellow diamond). From these seven independent optimizations, the maximum likelihood estimated parameter set (which includes “IgG control parameters” and antibody mechanism parameters) for the treated macaques is selected from the pairing that provides the greatest overall likelihood is used for that single treated macaque for a specified mechanism. The pairing of the treated macaques with the IgG control macaque helps inform the “IgG control parameters”: the target cell proliferation, *r_T_*; the effector cell killing rate, m; the viral production rate, *p*; the half-saturation effector cell proliferation constant, *K_B_*; and for the saturated source model the half-saturation effector cell source constant, *K_P_*.

# References

1. Hobbs TR, Blue SW, Park BS, Greisel JJ, Conn PM, Pau FK-Y. Measurement of Blood Volume in Adult Rhesus Macaques (Macaca mulatta). J Am Assoc Lab Anim Sci. 2015;54: 687–693.

2. Fooden J. Comparative Review of Fascicularis-group Species of Macaques (primates: Macaca). Fieldiana . 2006; 1–43.

3. Byrareddy SN, Kallam B, Arthos J, Cicala C, Nawaz F, Hiatt J, et al. Targeting α4β7 integrin reduces mucosal transmission of simian immunodeficiency virus and protects gut-associated lymphoid tissue from infection. Nat Med. 2014;20: 1397–1400.

4. Pereira LE, Onlamoon N, Wang X, Wang R, Li J, Reimann KA, et al. Preliminary in vivo efficacy studies of a recombinant rhesus anti-alpha(4)beta(7) monoclonal antibody. Cell Immunol. 2009;259: 165–176.

5. Abbink P, Mercado NB, Nkolola JP, Peterson RL, Tuyishime H, McMahan K, et al. Lack of therapeutic efficacy of an antibody to αβ in SIVmac251-infected rhesus macaques. Science. 2019;365: 1029–1033.

6. Desikan R, Raja R, Dixit NM. Modeling how early passive immunization with broadly neutralizing antibodies elicits lasting control of SHIV infection. doi:10.1101/548727

7. Chen S, Lai C, Wu X, Lu Y, Han D, Guo W, et al. Variability of bio-clinical parameters in Chinese-origin Rhesus macaques infected with simian immunodeficiency virus: a nonhuman primate AIDS model. PLoS One. 2011;6: e23177.

8. Byrareddy SN, Arthos J, Cicala C, Villinger F, Ortiz KT, Little D, et al. Sustained virologic control in SIV+ macaques after antiretroviral and α4β7 antibody therapy. Science. 2016;354: 197–202.

9. Xie Y, Brand JE, Jann B. Estimating Heterogeneous Treatment Effects with Observational Data. Sociol Methodol. 2012;42: 314–347.

10. Di Mascio M, Dornadula G, Zhang H, Sullivan J, Xu Y, et al. In a Subset of Subjects on Highly Active Antiretroviral Therapy, Human Immunodeficiency Virus Type 1 RNA in Plasma Decays from 50 to <5 Copies per Milliliter, with a Half-Life of 6 Months. Journal of Virology. 2003. pp. 2271–2275. doi:10.1128/jvi.77.3.2271-2275.2003

11. Palmer S, Maldarelli F, Wiegand A, Bernstein B, Hanna GJ, Brun SC, et al. Low-level viremia persists for at least 7 years in patients on suppressive antiretroviral therapy. Proceedings of the National Academy of Sciences. 2008. pp. 3879–3884. doi:10.1073/pnas.0800050105

12. Gandhi RT, Zheng L, Bosch RJ, Chan ES, Margolis DM, Read S, et al. The effect of raltegravir intensification on low-level residual viremia in HIV-infected patients on antiretroviral therapy: a randomized controlled trial. PLoS Med. 2010;7. doi:10.1371/journal.pmed.1000321

13. Dinoso JB, Kim SY, Wiegand AM, Palmer SE, Gange SJ, Cranmer L, et al. Treatment intensification does not reduce residual HIV-1 viremia in patients on highly active antiretroviral therapy. Proceedings of the National Academy of Sciences. 2009. pp. 9403–9408. doi:10.1073/pnas.0903107106

14. McMahon D, Jones J, Wiegand A, Gange SJ, Kearney M, Palmer S, et al. Short‐Course Raltegravir Intensification Does Not Reduce Persistent Low‐Level Viremia in Patients with HIV‐1 Suppression during Receipt of Combination Antiretroviral Therapy. Clinical Infectious Diseases. 2010. pp. 912–919. doi:10.1086/650749

15. Ansari AA, Reimann KA, Mayne AE, Takahashi Y, Stephenson ST, Wang R, et al. Blocking of α4β7 gut-homing integrin during acute infection leads to decreased plasma and gastrointestinal tissue viral loads in simian immunodeficiency virus-infected rhesus macaques. The Journal of Immunology. 2011;186: 1044–1059.

16. Brandin E, Thorstensson R, Bonhoeffer S, Albert J. Rapid viral decay in simian immunodeficiency virus-infected macaques receiving quadruple antiretroviral therapy. J Virol. 2006;80: 9861–9864.

17. Althaus CL, De Boer RJ. Implications of CTL-Mediated Killing of HIV-Infected Cells during the Non-Productive Stage of Infection. PLoS One. 2011;6: e16468.

18. Mohri H, Bonhoeffer S, Monard S, Perelson a. S, Ho DD. Rapid turnover of T lymphocytes in SIV-infected rhesus macaques. Science. 1998;279: 1223–1227.

19. Sachsenberg N, Perelson AS, Yerly S, Schockmel GA, Leduc D, Hirschel B, et al. Turnover of CD4+ and CD8+ T lymphocytes in HIV-1 infection as measured by Ki-67 antigen. J Exp Med. 1998;187: 1295–1303.

20. Reilly C, Wietgrefe S, Sedgewick G, Haase A. Determination of simian immunodeficiency virus production by infected activated and resting cells. AIDS. 2007;21: 163–168.

21. Chen HY, Di Mascio M, Perelson AS, Ho DD, Zhang L. Determination of virus burst size in vivo using a single-cycle SIV in rhesus macaques. Proc Natl Acad Sci U S A. 2007;104: 19079–19084.

22. Wyant T, Yang L, Fedyk E. In vitro assessment of the effects of vedolizumab binding on peripheral blood lymphocytes. MAbs. 2013;5: 842–850.

23. Iwata M, Hirakiyama A, Eshima Y, Kagechika H, Kato C, Song S-Y. Retinoic Acid Imprints Gut-Homing Specificity on T Cells. Immunity. 2004;21: 527–538.

24. Haanstra KG, Hofman SO, Lopes Estêvão DM, Blezer ELA, Bauer J, Yang L-L, et al. Antagonizing the α4β1 integrin, but not α4β7, inhibits leukocytic infiltration of the central nervous system in rhesus monkey experimental autoimmune encephalomyelitis. J Immunol. 2013;190: 1961–1973.

25. Guzzo C, Ichikawa D, Park C, Phillips D, Liu Q, Zhang P, et al. Virion incorporation of integrin α4β7 facilitates HIV-1 infection and intestinal homing. Science Immunology. 2017;2: eaam7341.

26. Igarashi T, Brown C, Azadegan A, Haigwood N, Dimitrov D, Martin MA, et al. Human immunodeficiency virus type 1 neutralizing antibodies accelerate clearance of cell-free virions from blood plasma. Nat Med. 1999;5: 211–216.

27. Tjomsland V, Ellegård R, Burgener A, Mogk K, Che KF, Westmacott G, et al. Complement opsonization of HIV-1 results in a different intracellular processing pattern and enhanced MHC class I presentation by dendritic cells. Eur J Immunol. 2013;43: 1470–1483.

28. Surrogate optimization for global minimization of time-consuming objective functions - MATLAB surrogateopt. [cited 31 Mar 2021]. Available: https://www.mathworks.com/help/gads/surrogateopt.html

29. Meeker WQ, Escobar LA. Teaching about Approximate Confidence Regions Based on Maximum Likelihood Estimation. The American Statistician. 1995;49: 48–53.

30. Stéphane Basmaciogullari MP. The activity of Nef on HIV-1 infectivity. Front Microbiol. 2014;5. doi:10.3389/fmicb.2014.00232

31. Minang JT, Trivett MT, Coren LV, Barsov EV, Piatak M, Ott DE, et al. Nef-mediated MHC class I down-regulation unmasks clonal differences in virus suppression by SIV-specific CD8 T cells independent of IFN-γ and CD107a responses. Virology. 2009. pp. 130–139. doi:10.1016/j.virol.2009.06.008
