# Supplementary material for: Mechanistic basis of post-treatment control of SIV after anti-α4β7 antibody therapy
Source: PLoS Comput Biol. 2021 Jun 9;17(6):e1009031. doi: 10.1371/journal.pcbi.1009031 (PMC8189501; doi:10.1371/journal.pcbi.1009031)
Supplement: S2 Text — (DOCX) [file pcbi.1009031.s002.docx]

**S2 Text. Supplementary Results: Mechanistic basis of post-treatment control of SIV after anti-α4β7 antibody therapy**

Chad R. Wells, Youfang Cao, David P. Durham, Siddappa N. Byrareddy, Aftab A. Ansari, Nancy H. Ruddle, Jeffrey P. Townsend, Alison P. Galvani and Alan S. Perelson

**Table of Contents**

Including increased viral clearance in the antigen presentation mechanism 1

Alternative effector cell source models 2

*Saturated effector cell source model* 2

*Antigen presenting cell effector cell source model* 2

Consideration of all source models and mechanisms 3

Correlation among estimated parameter values 3

Depletion of CD8 lymphocytes 4

Tables 6

Figures 10

References 11

# **Including increased viral clearance in the antigen presentation mechanism**

In addition to the anti-α4β7 antibody possibly increasing the source rate of effector cells, we account for the contribution of increased viral clearance in the antigen presentation mechanism. We found that the protection mechanism still had the greatest average AIC weight among the four mechanisms (Table A). With the addition of the viral clearance to the increased antigen presentation mechanism, we observed a moderate increase in the average AIC weight but no direct change in the dominant mechanism among the individual macaques.

# **Alternative effector cell source models**

We consider two alternative effector cell source models. One is a saturated source dependent on the infected cell concentration and the other is a source is dependent on the concentration of antigen presenting cells.

## *Saturated effector cell source model*

We found that the protection mechanism to be the dominant mechanism, with increased antigen presentation (in the absence of viral clearance) having a relatively similar average AIC weight (Table C). Considering the effects of increased viral clearance with the increased antigen presentation mechanism, the protection mechanism had a substantially larger average AIC weight than the other mechanisms (Table D).

We found the dominant mechanism was different for some treated macaques when compared to the baseline model. Specifically, RDa15 switched from viral neutralization to increased viral clearance; RId14 from virus neutralization to protection, RSd14 from increased viral clearance to increased antigen presentation (assuming no increased viral clearance), and ROv14 from increased viral clearance to increased antigen presentation (assuming no increase viral clearance).

## *Antigen presenting cell effector cell source model*

We found the protection mechanism to be the dominant mechanism based on the average AIC weight (Table E). We found that under this specified source of effector cells that the protection mechanism was the dominant one in five out of the eight treated macaques, with increased antigen presentation (assuming no increased viral clearance) best explaining the viral dynamics in the other three macaques. Considering the effects of increased viral clearance with the increased antigen presentation mechanism, the dominance shifted to increased antigen presentation (Table F), with the viral dynamics for four out of eight best explained by protection and the remaining four by increased antigen presentation with viral clearance.

# **Consideration of all source models and mechanisms**

Other mechanisms fit the viral load data better in some macaques with an alternative effector source model (saturated source (SS) model or antigen presenting cell source (APCS) model) when compared to the baseline (BL) model (S5−S12 Table). Using all models, an effector source model was selected for each mechanism in the individual macaque based on the lowest AIC score among the three effector source models (S5−S12 Table).

We found that the protection mechanism was the dominant overall mechanism based on the average AIC weight (Table G and H). The dominant mechanism deviates from that specified in the baseline effector cell source model in two out of the eight treated macaques when antigen presentation is considered in the absence of the effects of increased viral clearance. When considering the impact of increased viral clearance, two out of the eight treated macaques mechanism changed compared to the baseline effector cell source model (Table A versus Table H).

# **Correlation among estimated parameter values**

In addition to the profile likelihood analysis (S3 Text), we examined the relationship between the estimated parameter values by calculating the correlations among them for each mechanism across the eight treated macaques. (Table I). These correlations provide an understanding of the factors contributing to interacting components for each mechanism, where the profile likelihood analysis provides information about identifiability of parameters.

For the baseline model, there was a significant and strong correlation between the effector cell killing rate, *m*, and the viral production rate, *p*. This relationship is somewhat expected, since with greater amount of viral replication, i.e., a larger *p*, the effector cell response needs to be stronger to attain post-treatment control. This correlation is not as apparent in the other two effector source models, as the level of viral replication influences the source rate of the effector cell population.

For the protection mechanism, there was a significant correlation between the maximum rate at which protection is attained, 𝜌, and the rate protection wanes, 𝜔, for the baseline model and the antigen presenting cell source model. The faster protection wanes the faster target cells must enter the protected state to limit the viral replication.

Many of the correlations can likely be attributed to the delicate balance required to obtain two viral load set points, such that with the anti-α4β7antibody treatment post-treatment control is achieved and rebound occurs with the anti-α4β7antibody treatment.

# **Depletion of CD8 lymphocytes**

Macaques treated with an anti-α4β7antibody, under the umbrella of cART, maintained low level of viremia in the plasma for over a year. To evaluate the role of the immune response in long term suppression of viral replication in these macaques, CD8 lymphocyte depletion studies were conducted (Fig A). Of the macaques that achieved post-treatment control, six where first administered an anti-CD8α antibody to deplete CD8^+^ T cells and NK cells. One week after the depletion of CD8α^+^ lymphocytes, the viral load rapidly increased in the peripheral blood of the macaques. Four macaques regained control of viremia eight weeks following the depletion, with two experiencing viral blips.

The six macaques were then given an anti-CD8β antibody to deplete CD8^+^ T cells and not NK cells 13 weeks after the administration of the anti-CD8α antibody. Following this depletion, the viral load in two of the macaques remained below the limit of detection, two transiently rebounded and two exhibited substantial viral rebound. Six weeks after the administration of the anti-CD8β antibody, five of the six macaques had no detectable viremia.

We ran our model out to 81 weeks post-infection, removed 100% of effector cells, and then allowed the effector cells to repopulate through the source term. This temporary depletion of effector cells resulted in the resurgence of viremia (Fig B). These simulations were not aimed to reflect the viral dynamics observed in the CD8 depletion study, as this understanding is outside the scope of the project. This analysis and data highlight the importance of the effector cell population in maintaining post-treatment control.

# **Tables**

**Table A:** AIC weights* for enhanced viral clearance, viral neutralization, protection, improved antigen presentation with increased viral clearance mechanisms for the baseline source model.

| **Mechanism** | **ROq14** | **RDa15** | **RFa15** | **RLn12** | **RId14** | **ROo13** | **RSd14** | **ROv14** | **Average** |
| --- | --- | --- | --- | --- | --- | --- | --- | --- | --- |
| Viral clearance | 0.143 | 0.404 | 0.000 | 0.004 | 0.240 | 0.032 | **0.632** | **0.546** | 0.250 |
| Virus neutralization | **0.813** | **0.490** | 0.000 | 0.002 | **0.412** | 0.004 | 0.005 | 0.000 | 0.216 |
| Protection | 0.007 | 0.000 | **1.000** | **0.985** | 0.196 | 0.000 | 0.282 | 0.308 | **0.347** |
| Antigen presentation  with viral clearance | 0.038 | 0.106 | 0.000 | 0.008 | 0.153 | **0.965** | 0.081 | 0.146 | 0.187 |

* AIC weights are rounded and as a result some columns may not sum to one.

**Table B:** Specified parameter values for the saturated effector cell source model and the antigen presenting cell source model.

| **Parameter** | **Description** | **Value** | **Units** | **Reference** |  |
| --- | --- | --- | --- | --- | --- |
| 𝛿 | Infected cell death rate due to viral cytopathic effects in saturated source model | 0.60 | per day | Calibrated | |
| 𝛿 | Infected cell death rate due to viral cytopathic effects in antigen presenting cell model | 0.20 | per day | Calibrated |  |
| *d_E_* | Maximum exhaustion rate for cytotoxic effector cells in saturated source model | 1.35 | per day | Calibrated |  |
| *d_E_* | Maximum exhaustion rate for cytotoxic effector cells in antigen presenting cell model | 5.85 | per day | Calibrated |  |
| *b_D_* | Rate antigen presenting cells encounter antigen for antigen presenting cell source model | 24 | per day per cell / ml | Assumed |  |
| *d_D_* | Death rate of antigen presenting cells for antigen presenting cell source model | 0.347 | per day | [1] |  |
| *λ_E_* | Maximum effector cell source rate for saturated source model | 10^3^ | cells / ml per day | Assumed ^a^ |  |
| *λ_E_* | Rate of effector cell production for antigen presenting cell model | 10^3^ | per day | Assumed ^a^ |  |
| ^a^ Assumed based on the calibration of *λ_E_* in the baseline effector cell source model. The effector cell killing rate, *m*, will scale accordingly based on changes to these values (S1 Text). | | | | |  |

**Table C:** AIC weights* for the enhanced viral clearance, the viral neutralization, the protection, and the improved antigen presentation mechanisms for *the saturated source model*.

| **Mechanism** | **ROq14** | **RDa15** | **RFa15** | **RLn12** | **RId14** | **ROo13** | **RSd14** | **ROv14** | **Average** |
| --- | --- | --- | --- | --- | --- | --- | --- | --- | --- |
| Viral clearance | 0.022 | **0.902** | 0.000 | 0.001 | 0.072 | 0.000 | 0.000 | 0.000 | 0.125 |
| Virus neutralization | **0.977** | 0.026 | 0.000 | 0.000 | 0.279 | 0.000 | 0.000 | 0.000 | 0.160 |
| Protection | 0.000 | 0.004 | **1.000** | **0.999** | **0.517** | 0.003 | 0.000 | 0.380 | **0.363** |
| Antigen presentation | 0.001 | 0.068 | 0.000 | 0.000 | 0.133 | **0.997** | **1.000** | **0.620** | 0.352 |

* AIC weights are rounded and as a result some columns may not sum to one.

**Table D:** AIC weights* for the enhanced viral clearance, the viral neutralization, the protection, and the improved antigen presentation with increased viral clearance mechanisms for *the saturated source model*.

| **Mechanism** | **ROq14** | **RDa15** | | **RFa15** | | **RLn12** | | **RId14** | | | **ROo13** | | **RSd14** | | **ROv14** | | **Average** |
| --- | --- | --- | --- | --- | --- | --- | --- | --- | --- | --- | --- | --- | --- | --- | --- | --- | --- |
| Viral clearance | 0.022 | | **0.779** | | 0.000 | | 0.001 | | 0.081 | 0.001 | | 0.001 | | 0.000 | | 0.111 | |
| Virus neutralization | **0.974** | | 0.022 | | 0.000 | | 0.000 | | 0.315 | 0.002 | | 0.000 | | 0.000 | | 0.164 | |
| Protection | 0.000 | | 0.004 | | **0.999** | | **0.998** | | **0.584** | 0.041 | | 0.001 | | **0.900** | | **0.441** | |
| Antigen presentation  with viral clearance | 0.004 | | 0.195 | | 0.001 | | 0.000 | | 0.020 | **0.957** | | **0.998** | | 0.099 | | 0.284 | |

* AIC weights are rounded and as a result some columns may not sum to one.

**Table E:** AIC weights* for the enhanced viral clearance, the viral neutralization, the protection, and the improved antigen presentation mechanisms for *the antigen presenting cell source model*.

| **Mechanism** | **ROq14** | **RDa15** | **RFa15** | **RLn12** | **RId14** | **ROo13** | **RSd14** | **ROv14** | **Average** |
| --- | --- | --- | --- | --- | --- | --- | --- | --- | --- |
| Viral clearance | 0.057 | 0.042 | 0.000 | 0.000 | 0.028 | 0.000 | 0.000 | 0.000 | 0.016 |
| Virus neutralization | 0.000 | 0.006 | 0.000 | 0.000 | 0.193 | 0.000 | 0.000 | 0.000 | 0.025 |
| Protection | **0.943** | 0.261 | **0.822** | **0.885** | **0.425** | **0.949** | 0.028 | 0.000 | **0.539** |
| Antigen presentation | 0.000 | **0.691** | 0.178 | 0.115 | 0.354 | 0.051 | **0.972** | **1.000** | 0.420 |

* AIC weights are rounded and as a result some columns may not sum to one.

**Table F:** AIC weights* for the enhanced viral clearance, the viral neutralization, the protection, and the improved antigen presentation with increased viral clearance mechanisms for *the antigen presenting cell source model*.

| **Mechanism** | **ROq14** | **RDa15** | **RFa15** | **RLn12** | **RId14** | **ROo13** | **RSd14** | **ROv14** | **Average** |
| --- | --- | --- | --- | --- | --- | --- | --- | --- | --- |
| Viral clearance | 0.056 | 0.086 | 0.000 | 0.000 | 0.022 | 0.000 | 0.000 | 0.000 | 0.020 |
| Virus neutralization | 0.000 | 0.012 | 0.000 | 0.000 | 0.148 | 0.000 | 0.000 | 0.000 | 0.020 |
| Protection | **0.929** | **0.530** | **0.946** | 0.002 | 0.328 | **0.922** | 0.100 | 0.000 | 0.470 |
| Antigen presentation with viral clearance | 0.015 | 0.372 | 0.054 | **0.998** | **0.502** | 0.078 | **0.900** | **1.000** | **0.490** |

* AIC weights are rounded and as a result some columns may not sum to one.

**Table G:** AIC weights* for the enhanced viral clearance, the viral neutralization, the protection, and the improved antigen presentation mechanisms for *the AIC selected effector cell source model.*

| **Mechanism** | **ROq14** | **RDa15** | **RFa15** | **RLn12** | **RId14** | **ROo13** | **RSd14** | **ROv14** | **Average** |
| --- | --- | --- | --- | --- | --- | --- | --- | --- | --- |
| Viral clearance | 0.078 | 0.452 | 0.000 | 0.004 | 0.279 | 0.002 | 0.235 | **0.541** | 0.199 |
| Virus neutralization | 0.442 | **0.548** | 0.000 | 0.002 | **0.479** | 0.000 | 0.002 | 0.000 | 0.184 |
| Protection | **0.476** | 0.000 | **1.000** | **0.993** | 0.228 | 0.001 | 0.105 | 0.305 | **0.388** |
| Antigen presentation | 0.004 | 0.000 | 0.000 | 0.001 | 0.013 | **0.996** | **0.658** | 0.154 | 0.228 |

* AIC weights are rounded and as a result some columns may not sum to one.

**Table H:** AIC weights* for the enhanced viral clearance, the viral neutralization, the protection, and the improved antigen presentation with increased viral clearance mechanisms for *the AIC selected effector cell source model*.

| **Mechanism** | **ROq14** | **RDa15** | **RFa15** | **RLn12** | **RId14** | **ROo13** | **RSd14** | **ROv14** | **Average** |
| --- | --- | --- | --- | --- | --- | --- | --- | --- | --- |
| Viral clearance | 0.076 | 0.404 | 0.000 | 0.001 | 0.240 | 0.031 | **0.611** | **0.546** | 0.239 |
| Virus neutralization | 0.435 | **0.490** | 0.000 | 0.001 | **0.412** | 0.004 | 0.005 | 0.000 | 0.168 |
| Protection | **0.468** | 0.000 | **1.000** | 0.242 | 0.196 | 0.020 | 0.272 | 0.308 | **0.313** |
| Antigen presentation with viral clearance | 0.020 | 0.106 | 0.000 | **0.757** | 0.153 | **0.946** | 0.112 | 0.146 | 0.280 |

* AIC weights are rounded and as a result some columns may not sum to one.

| **Table I: Significant correlations among the estimated parameter values for the different effector cell source models and the different mechanisms.** | | | |
| --- | --- | --- | --- |
|  | **Effector cell source model** | | |
| **Mechanism** | **Baseline** | **Saturated source** | **Antigen presenting cell source** |
| IgG Control  Animals (No antibody) | *r_T_* and *m*: *r=* 0.95 (*p*=0.001)  *r_T_* and *p*: *r=* 0.82 (*p*=0.024)  *K_B_* and 𝜎: *r=* -0.91 (*p*=0.005) | *r_T_* and *p*: *r=* 0.80 (*p*=0.030) | *m* and *K_B_*: *r=* -0.87 (*p*=0.010) |
| Increased clearance | *m* and *p*: *r=* 0.94 (p<0.001) | *r_T_* and *K_B_*: *r=* 0.86 (*p=*0.006)  *m* and *K_P_*: *r=* 0.84 (*p=*0.010)  *p* and ψ: *r=* -0.76 (*p=*0.029)  *r_T_* and 𝜎: *r=* 0.77 (*p=*0.024)  *K_B_* and 𝜎: *r=* 0.78 (*p=*0.023) | *p* and *K_B_*: *r=* -0.84 (*p=*0.010)  *m* and 𝛾: *r=* -0.71 (*p=*0.049) |
| Viral neutralization | *m* and *p*: *r=* 0.94 (*p<*0.001)  *r_T_* and 𝛾: *r=* -0.75 (*p=*0.032)  *p* and 𝜎: *r=* -0.77 (*p=*0.026)  *K_B_* and 𝜎: *r=* 0.77 (*p=*0.024) | *m* and *K_B_*: *r=* -0.72 (*p=*0.043)  *r_T_* and 𝜎: *r=* 0.78 (*p=*0.022) | *r_T_* and *p*: *r=* -0.73 (*p=*0.039)  *K_B_* and ψ: *r=* -0.89 (*p=*0.003)  *m* and 𝛾: *r=* -0.75 (*p=*0.034) |
| Protection | *m* and *p*: *r=* 0.91 (*p=*0.002)  *r_T_* and 𝜔: *r=* 0.77 (*p=*0.025)  *m* and 𝜌: *r=* 0.78 (*p=*0.022)  𝜔 and 𝜌: *r=* 0.81 (*p=*0.016) | *r_T_* and *m*: *r=* -0.71 (*p=*0.048)  *r_T_* and *K_B_*: *r=* 0.75 (*p=*0.031)  *r_T_* and 𝜎: *r=* 0.82 (*p=*0.013)  *m* and 𝜎: *r=* -0.89 (*p=*0.003)  *K_B_* and 𝜎: *r=* 0.90 (*p=*0.002) | *r_T_* and *p*: *r=* -0.77 (*p=*0.024)  𝜔 and 𝜌: *r=* 0.83 (*p=*0.011)  *r_T_* and 𝜎: *r=* -0.86 (*p=*0.006)  *p* and 𝜎: *r=* 0.73 (*p=*0.038) |
| Antigen presentation | *r_T_* and *m*: *r=* 0.73 (*p=*0.041)  *m* and *p*: *r=* 0.97 (*p*<0.001) | No significant correlations | *m* and 𝜎: *r=* -0.79 (*p=*0.020)  𝛺 and 𝜎: *r=* -0.72 (*p=*0.044) |
| Antigen presentation with increased clearance | *m* and *p*: *r=* 0.91 (*p=*0.001)  *r_T_* and *K_B_*: *r=* 0.93 (*p<*0.001)  *r_T_* and 𝜎: *r=* 0.78 (*p=*0.021)  *K_B_* and 𝜎: *r=* 0.73 (*p=*0.041) | *r_T_* and *K_B_*: *r=* 0.86 (*p=*0.006)  *m* and *K_P_*: *r=* 0.86 (*p=*0.006)  ψ and 𝛾: *r=* 0.88 (*p=*0.004)  *r_T_* and 𝜎: *r=* 0.85 (*p=*0.008)  *K_B_* and 𝜎: *r=* 0.88 (*p=*0.004) | *p* and ψ: *r=* -0.75 (*p=*0.033)  *p* and 𝜎: *r=* 0.87 (*p=*0.005)  ψ and 𝜎: *r=* -0.83 (*p=*0.010) |

# **Figures**


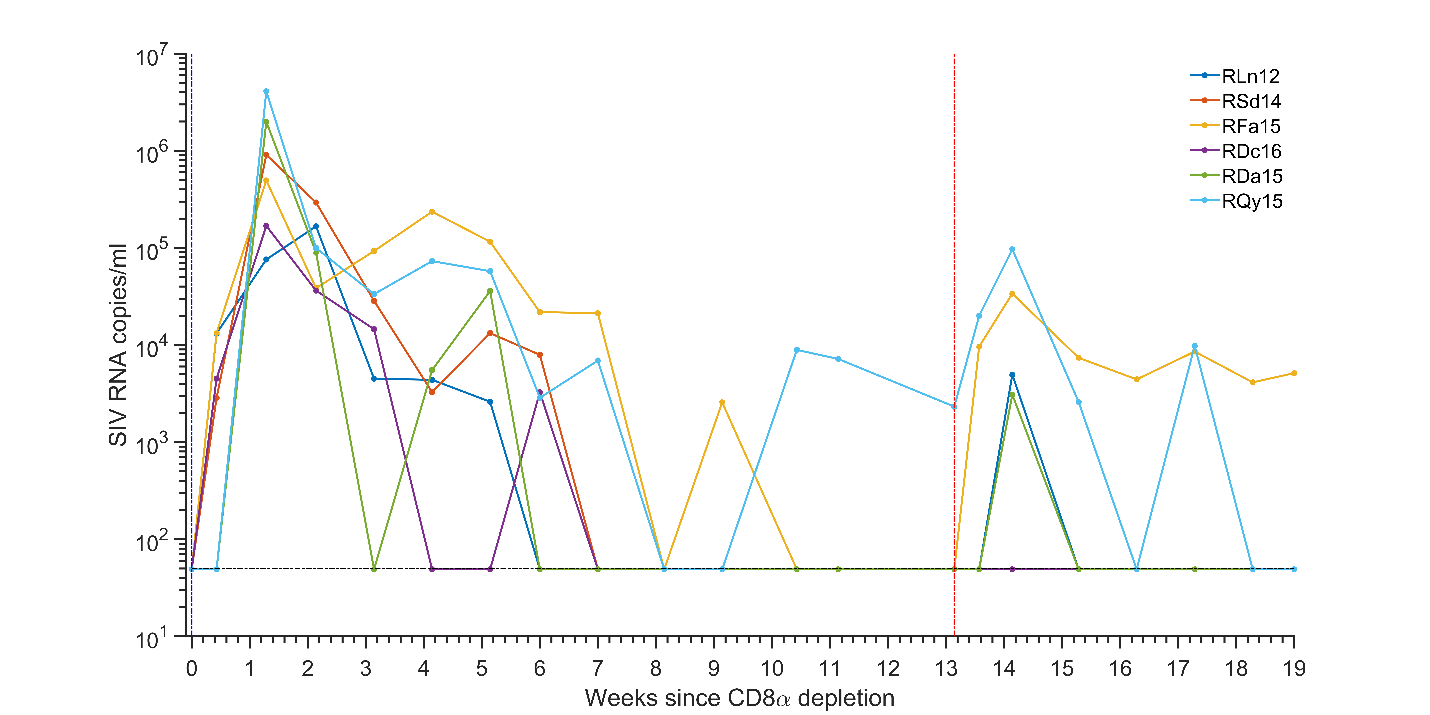


**Fig A:** SIV viral load dynamics in the peripheral blood of six macaques that underwent CD8α (vertical blue-dashed line) and CD8β depletion (vertical red-dashed line) after achieving post-treatment control after the administration of an anti-𝛼4𝛽7 antibody under the umbrella of cART during acute SIV infection.

**
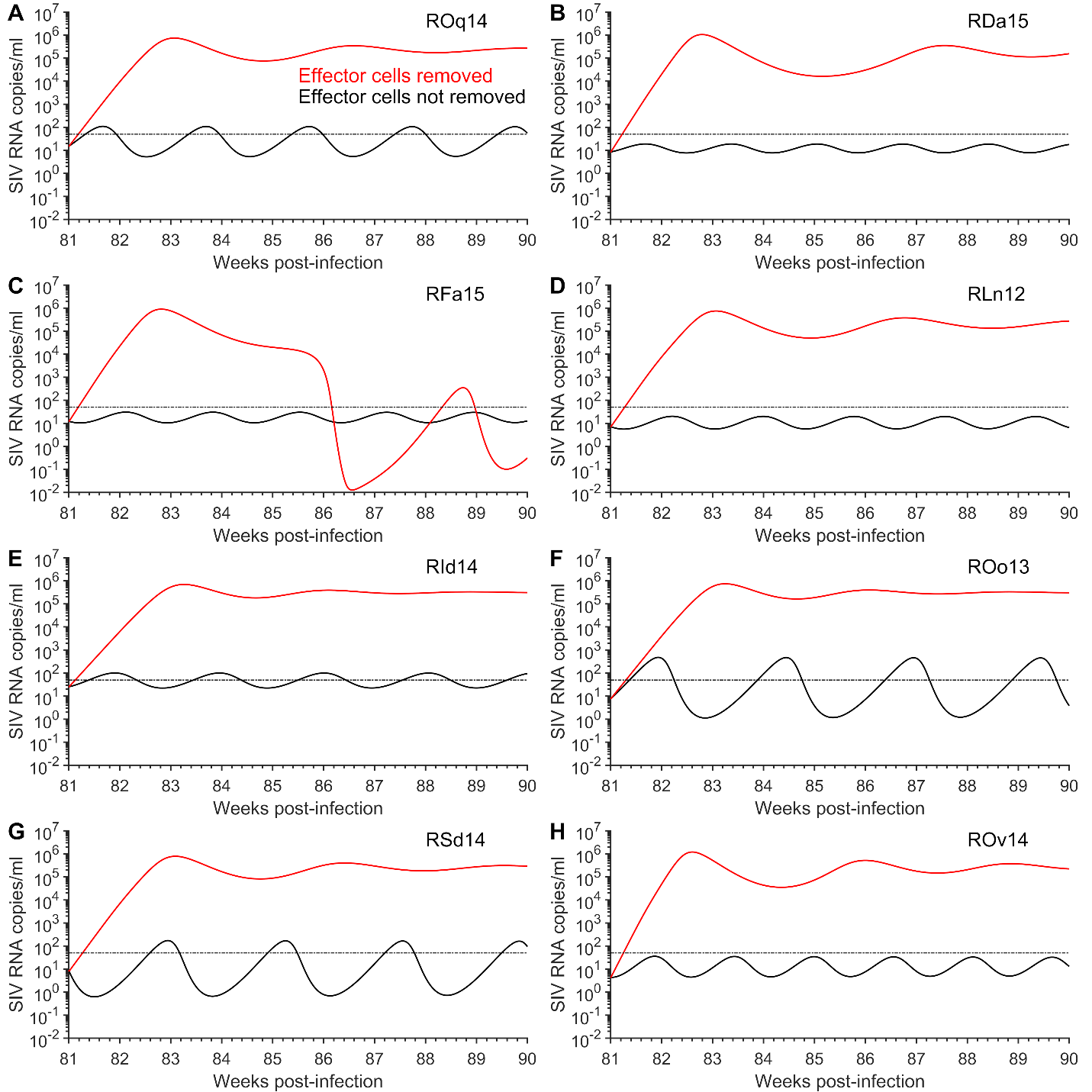
**

**Fig B:** Model simulated viral load dynamics of the eight treated macaques when 100% of the effector cell population is temporarily removed at 81 weeks post-infection (red) compared to when the effector cells remain present (black) for the baseline model.

# **References**

1. Min Chen JW. Programmed cell death of dendritic cells in immune regulation. Immunol Rev. 2010;236: 11.
