# Supplementary material for: Mechanistic basis of post-treatment control of SIV after anti-α4β7 antibody therapy
Source: PLoS Comput Biol. 2021 Jun 9;17(6):e1009031. doi: 10.1371/journal.pcbi.1009031 (PMC8189501; doi:10.1371/journal.pcbi.1009031)

S3 Text. Profile likelihood and sensitivity of model fit to assumed parameters

In the profile likelihood plots, the red dot indicates the maximum likelihood estimate, the black dots are the best binned output from the global optimization process, the black line shows the continuous approximation to the profile likelihood, and the blue dashed line indicates the threshold value for the confidence interval (the maximum log-likelihood value plus half the 95% percentile for the χ^2 distribution for k degrees of freedom; S1 Text). In the sensitivity analysis plots, the red dot indicates the maximum likelihood estimate for the baseline value (Table 1), the black line shows the log-likelihood for the alternative parameter value, and the blue dashed line (the maximum log-likelihood value plus half the 95% percentile for the χ^2 distribution for one degree of freedom) is an indicator of when the quality of the fit becomes significantly worse.

Contents

1 Likelihood profiles: Control macaques **3**
1.1 Baseline effector cell source model . . . 3
1.2 Saturated effector cell source model . . . 7
1.3 Antigen presenting cell effector cell source model . . . 14

2 Sensitivity anlaysis: Control macaques **18**
2.1 Baseline effector cell source model . . . 18
2.2 Saturated effector cell source model . . . 22
2.3 Antigen presenting cell effector cell source model . . . 26

3 Likelihood profiles: Increased viral clearance **30**
3.1 Baseline effector cell source model . . . 30
3.2 Saturated effector cell source model . . . 35
3.3 Antigen presenting cell effector cell source model . . . 40

4 Sensitivity anlaysis: Increased viral clearance **45**
4.1 Baseline effector cell source model . . . 45
4.2 Saturated effector cell source model . . . 50
4.3 Antigen presenting cell effector cell source model . . . 55

5 Likelihood profiles:Viral neutralization **60**
5.1 Baseline effector cell source model . . . 60
5.2 Saturated effector cell source model . . . 65
5.3 Antigen presenting cell effector cell source model . . . 70

6 Sensitivity anlaysis: Viral neutralization **75**
6.1 Baseline effector cell source model . . . 75
6.2 Saturated effector cell source model . . . 80
6.3 Antigen presenting cell effector cell source model . . . 85

7 Likelihood profiles: Protection **90**
7.1 Baseline effector cell source model . . . 90
7.2 Saturated effector cell source model . . . 95
7.3 Antigen presenting cell effector cell source model . . . 100

8 Sensitivity anlaysis: Protection **105**
8.1 Baseline effector cell source model . . . 105
8.2 Saturated effector cell source model . . . 110
8.3 Antigen presenting cell effector cell source model . . . 115

9 Likelihood profiles: Increased antigen presentation **120**
9.1 Baseline effector cell source model . . . 120
9.2 Saturated effector cell source model . . . 124
9.3 Antigen presenting cell effector cell source model . . . 129

10 Sensitivity anlaysis: Increased antigen presentation **134**
10.1 Baseline effector cell source model . . . 134
10.2 Saturated effector cell source model . . . 139
10.3 Antigen presenting cell effector cell source model . . . 144

11 Likelihood profiles: Increased antigen presentation with increased viral clearance **149**
11.1 Baseline effector cell source model . . . 149
11.2 Saturated effector cell source model . . . 154
11.3 Antigen presenting cell effector cell source model . . . 159

12 Sensitivity anlaysis: Increased antigen presentation with increased viral clearance **164**
12.1 Baseline effector cell source model . . . 164
12.2 Saturated effector cell source model . . . 169
12.3 Antigen presenting cell effector cell source model . . . 174

1 Likelihood profiles: Control macaques

1.1 Baseline effector cell source model

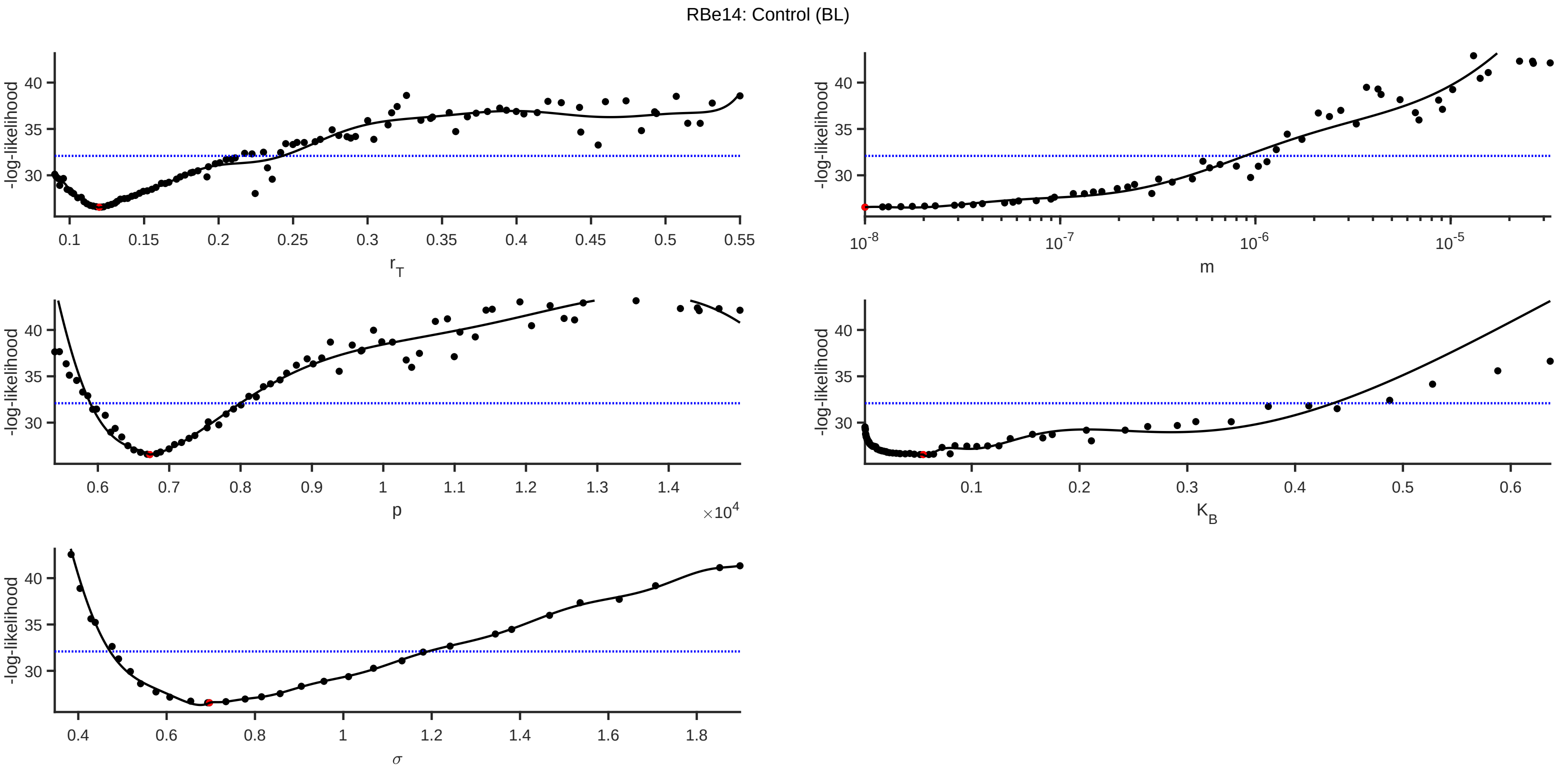

RIt11: Control (BL)

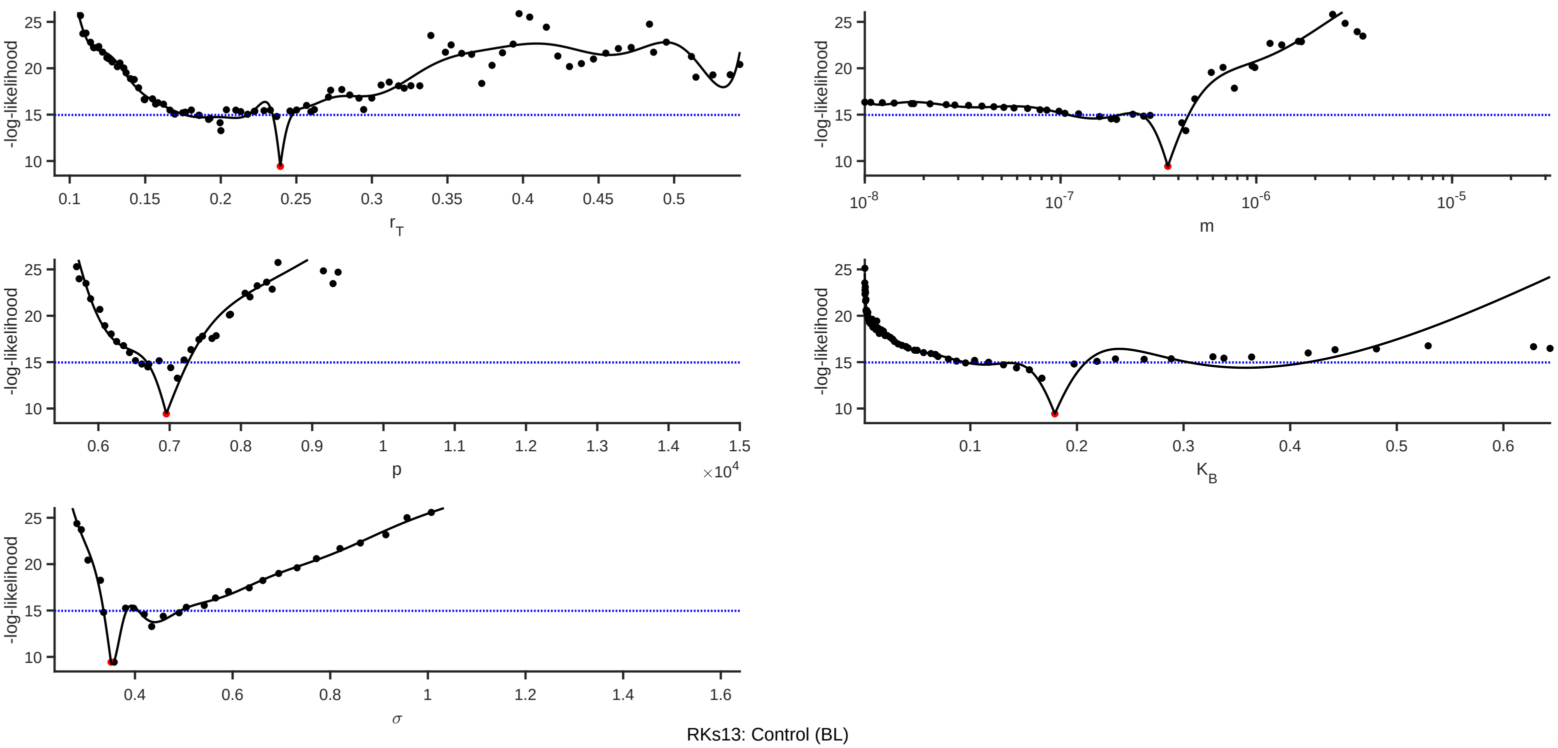

RKs13: Control (BL)

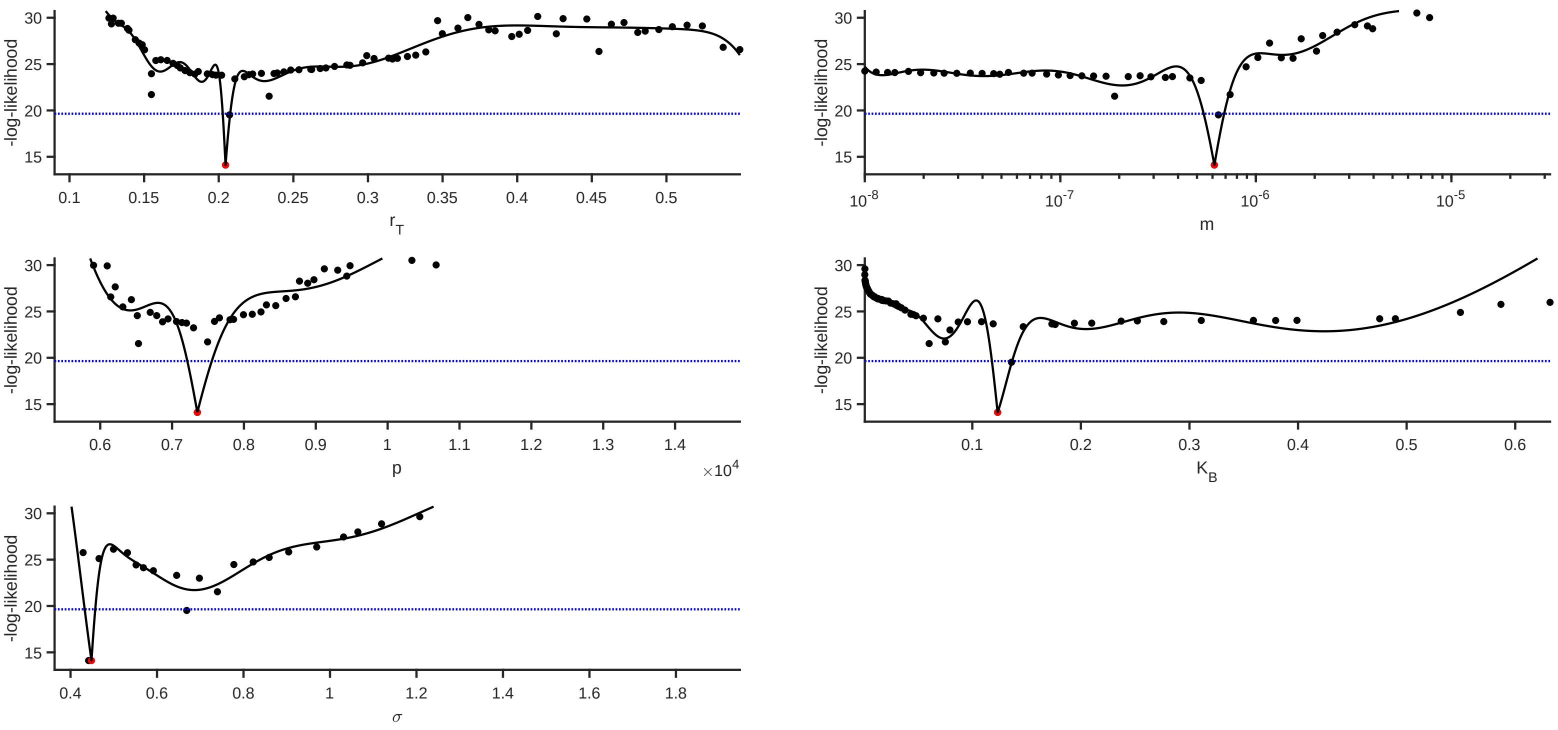

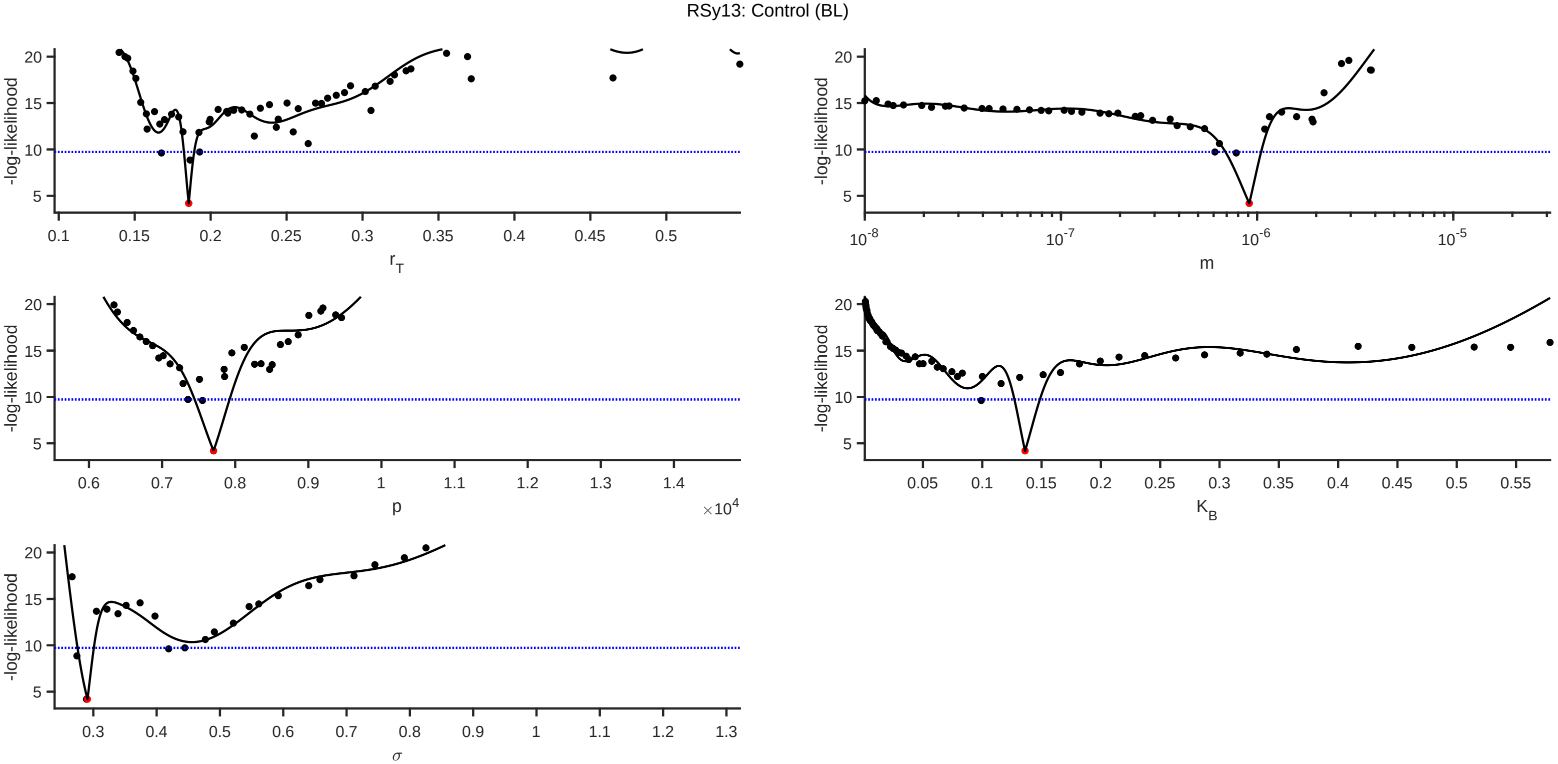
RSy13: Control (BL)
-log-likelihood
r_T
m
p
$\times 10^4$
K_B
σ

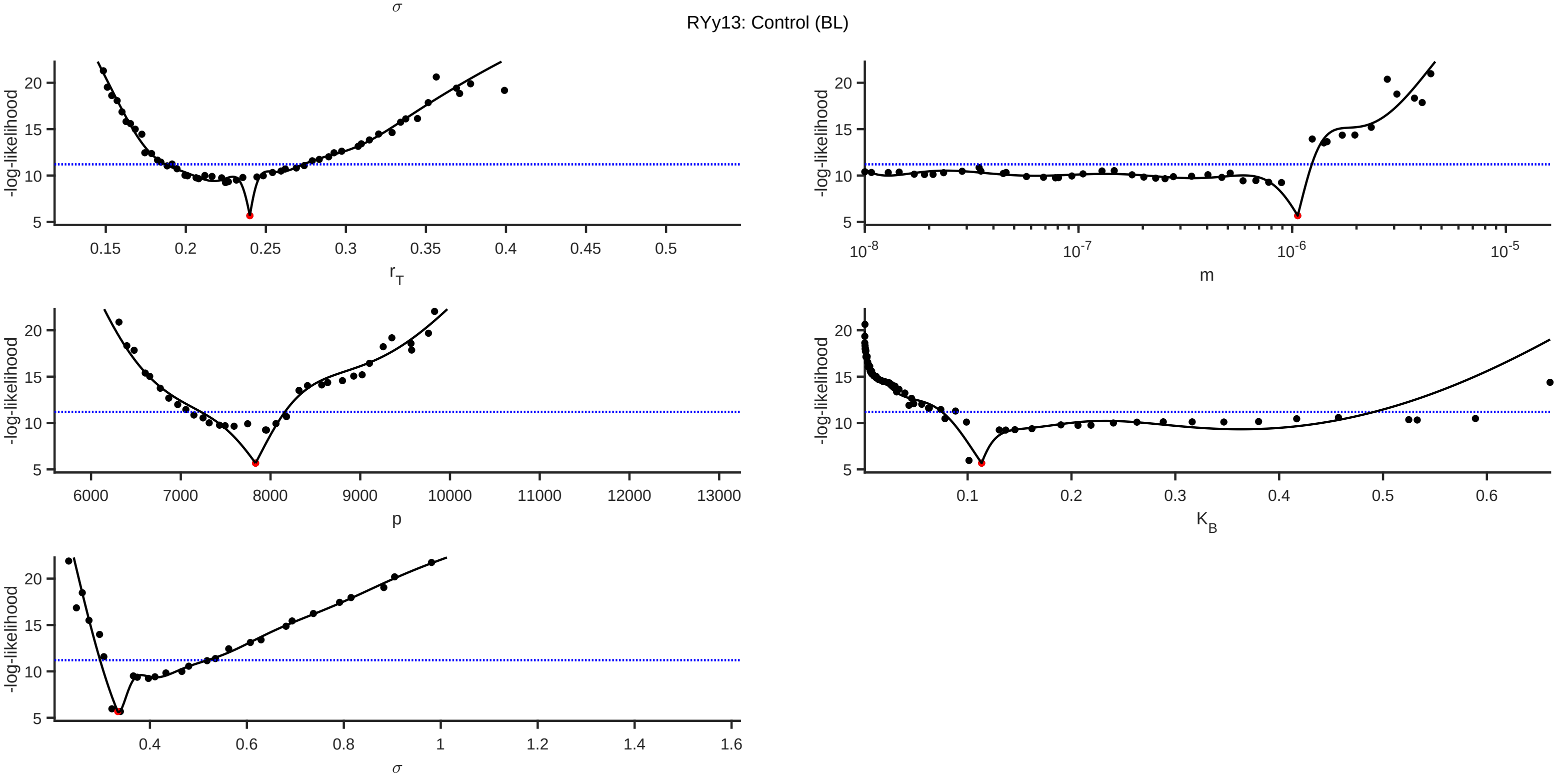
RYy13: Control (BL)
-log-likelihood
r_T
m
p
K_B
σ

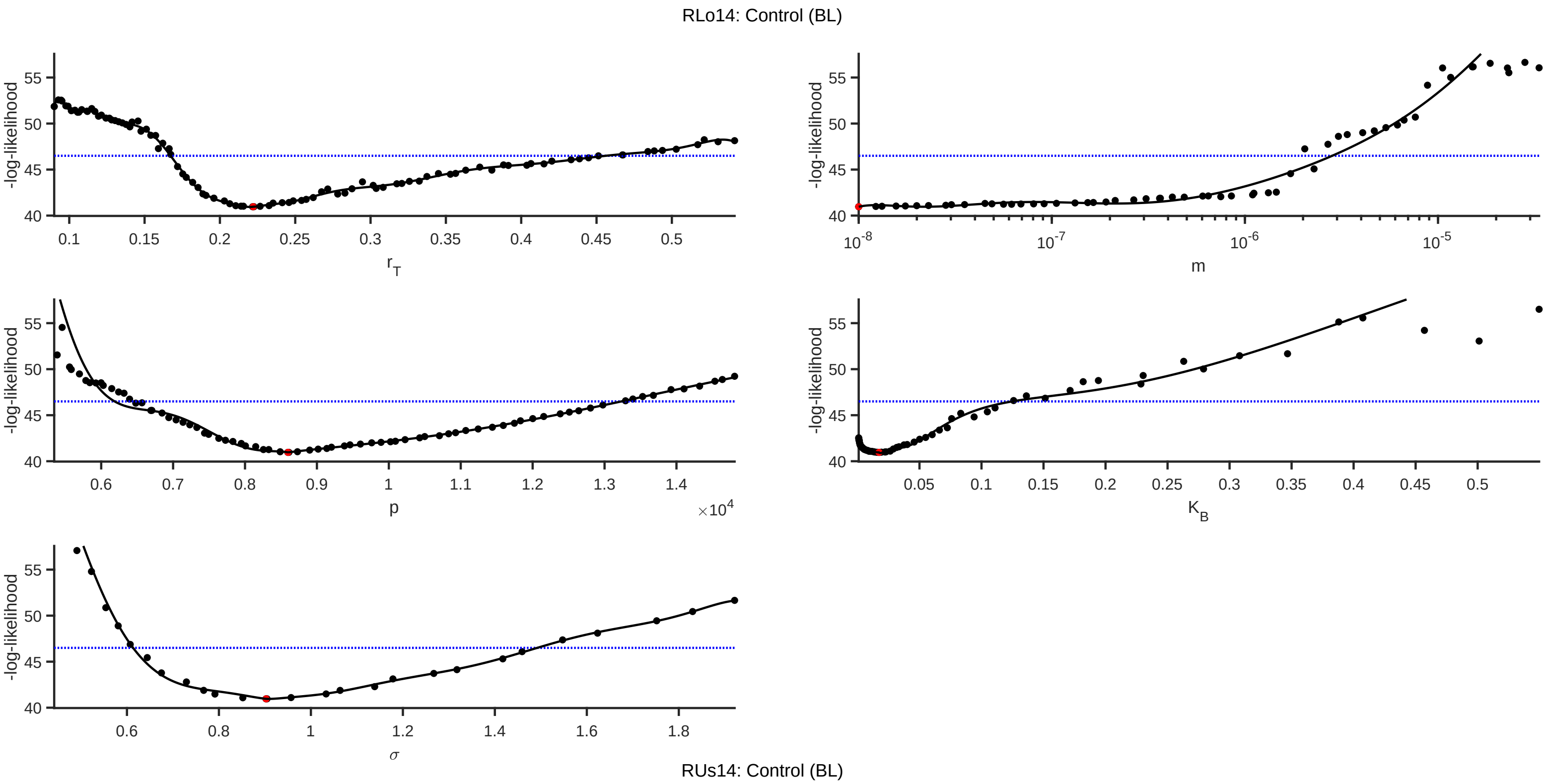
RLo14: Control (BL)
-log-likelihood
r_T
m
p
×10^4
K_B
σ

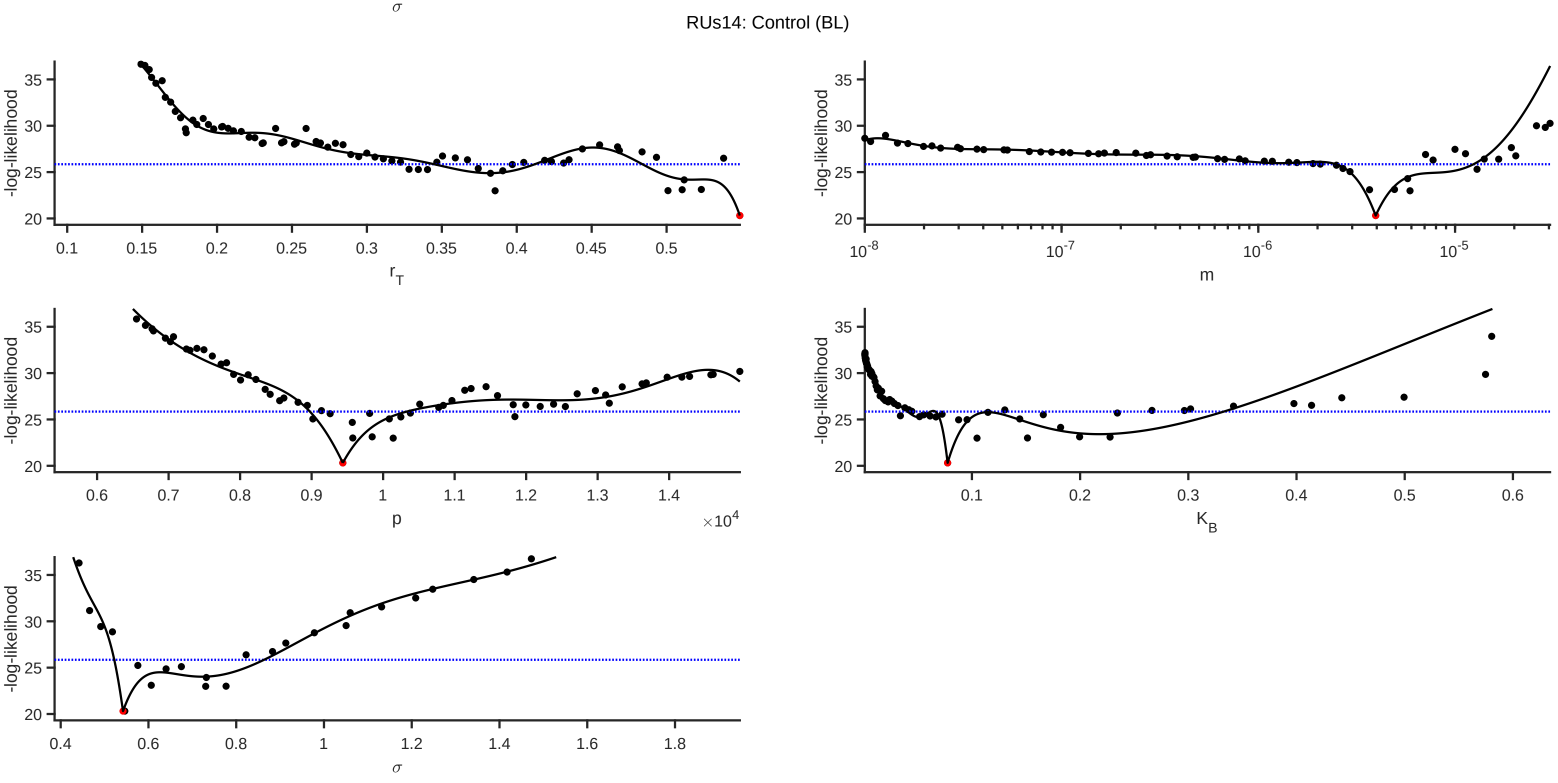
RUs14: Control (BL)
-log-likelihood
r_T
m
p
×10^4
K_B
σ

1.2 Saturated effector cell source model

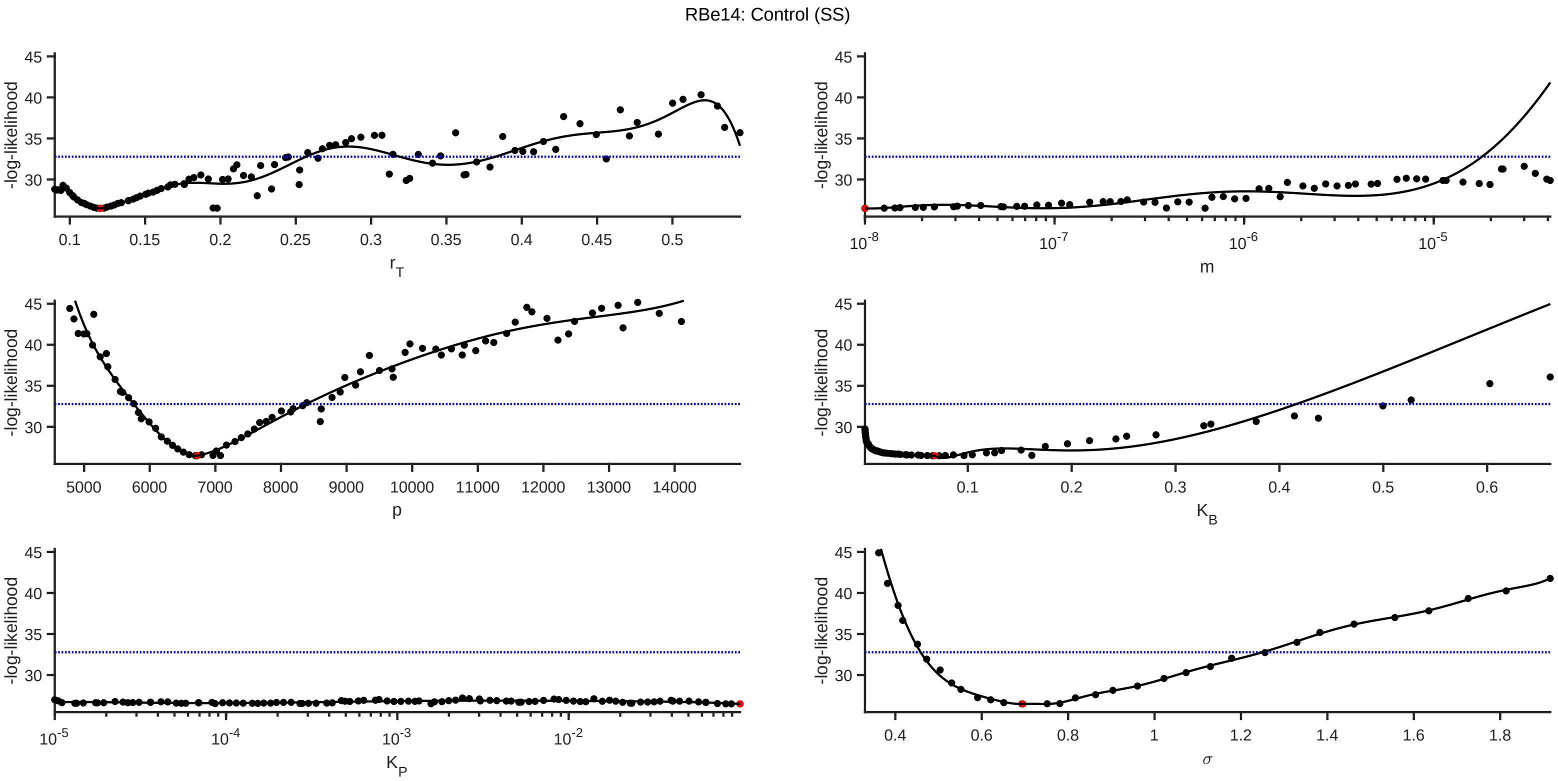

RIt11: Control (SS)

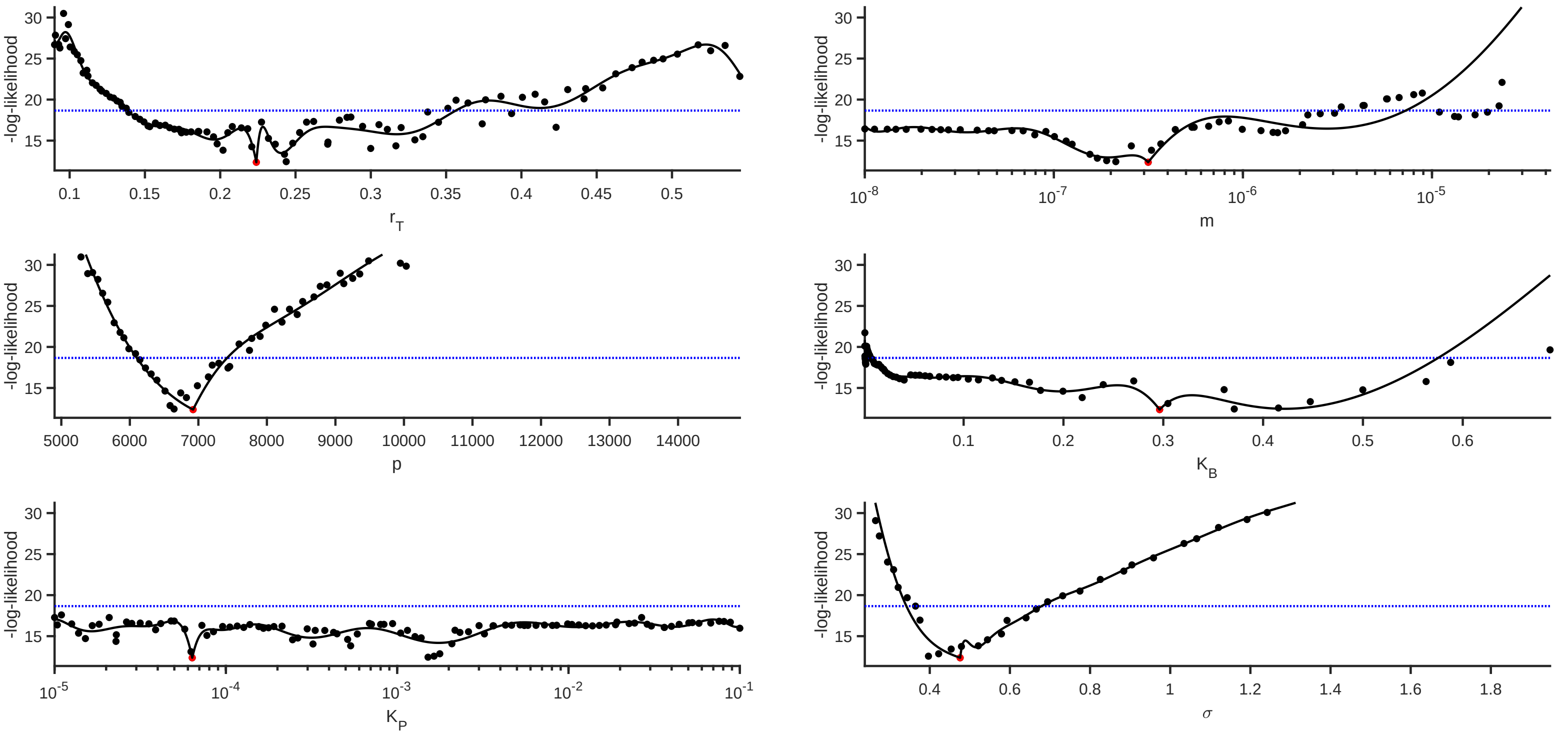

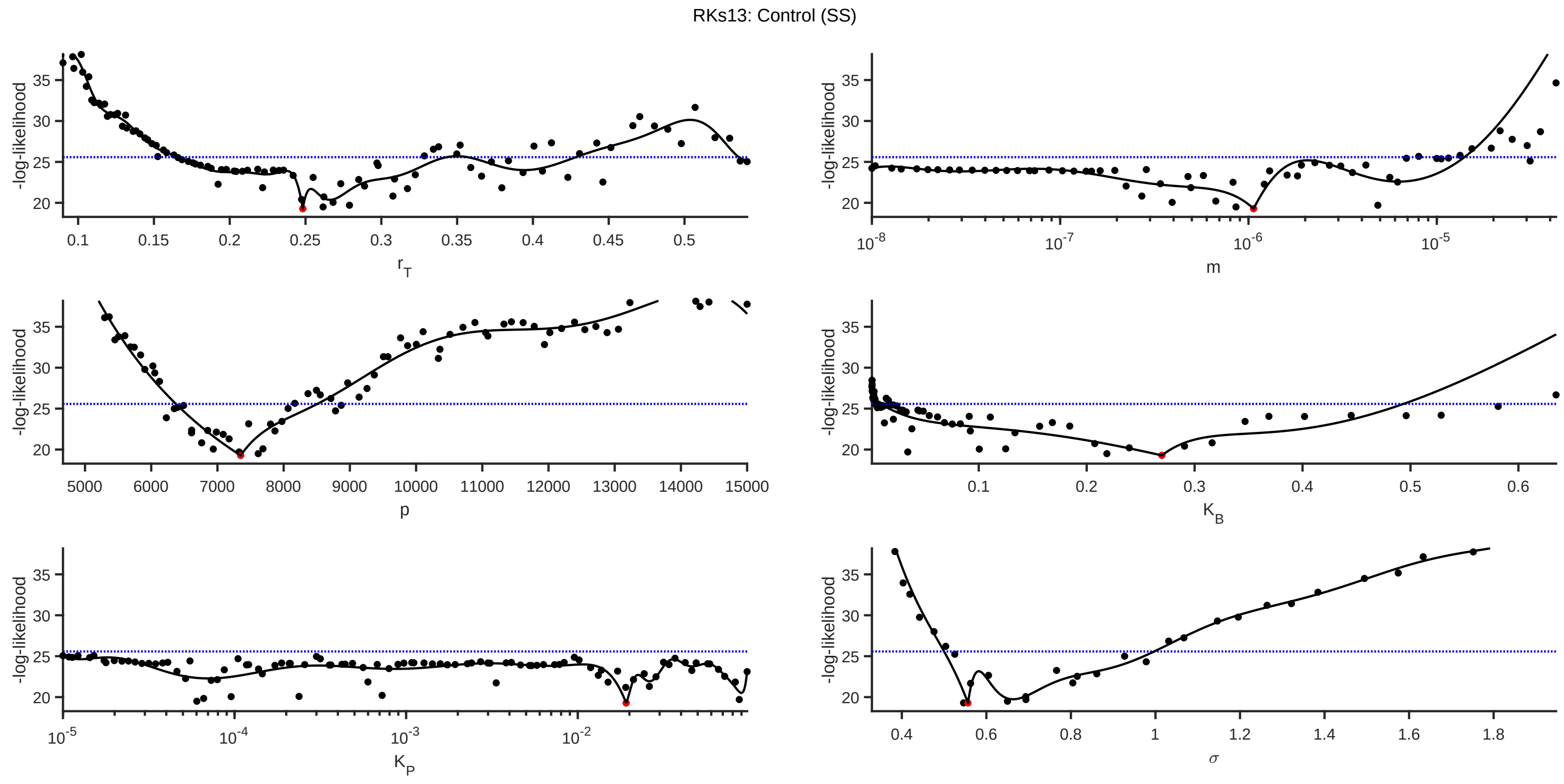

RKs13: Control (SS)
-log-likelihood
r_T
m
p
K_B
K_P
σ

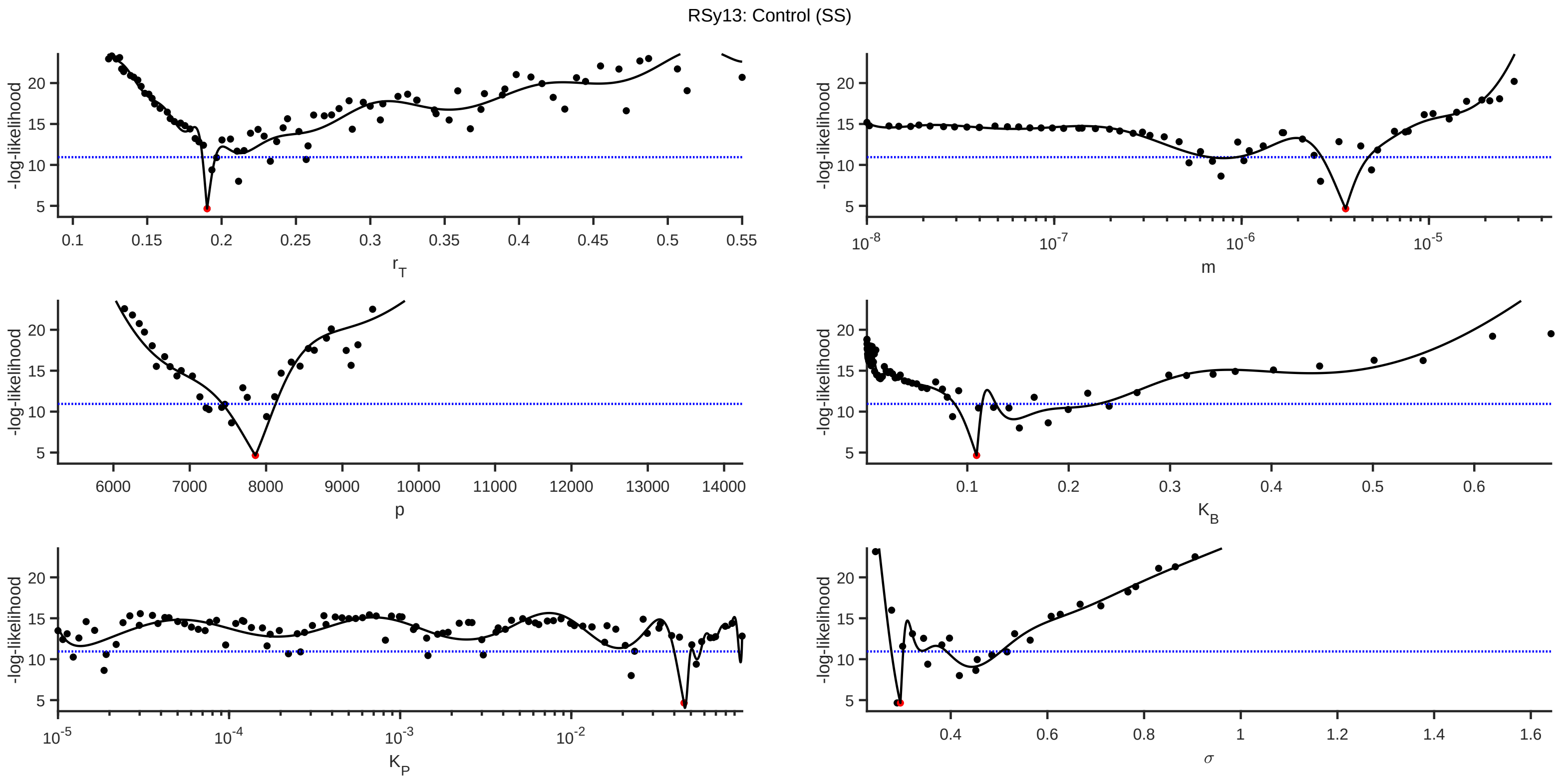
RSy13: Control (SS)
-log-likelihood
r_T
m
p
K_B
K_P
σ

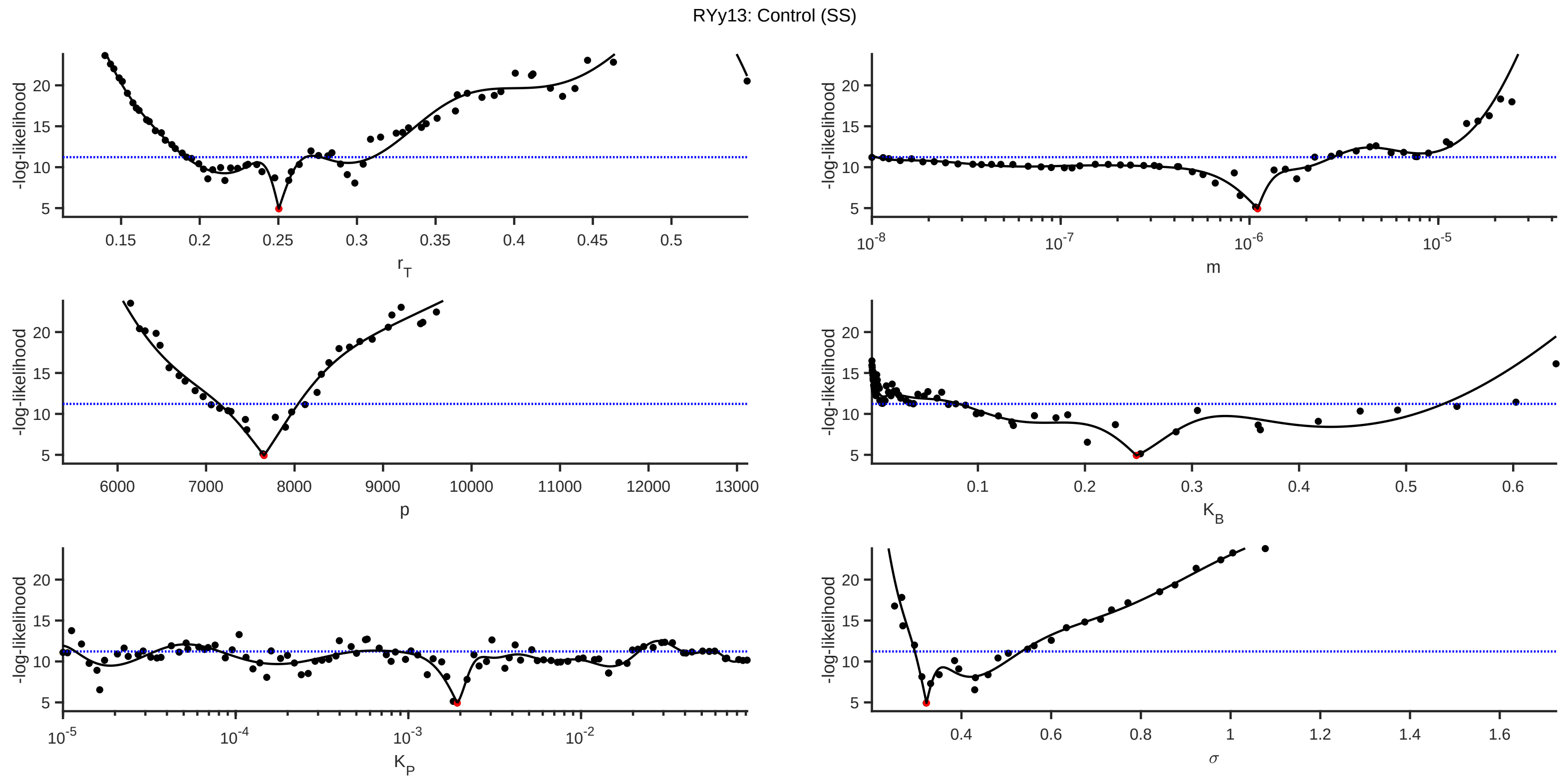

RYy13: Control (SS)
-log-likelihood
r_T
m
p
K_B
K_P
σ

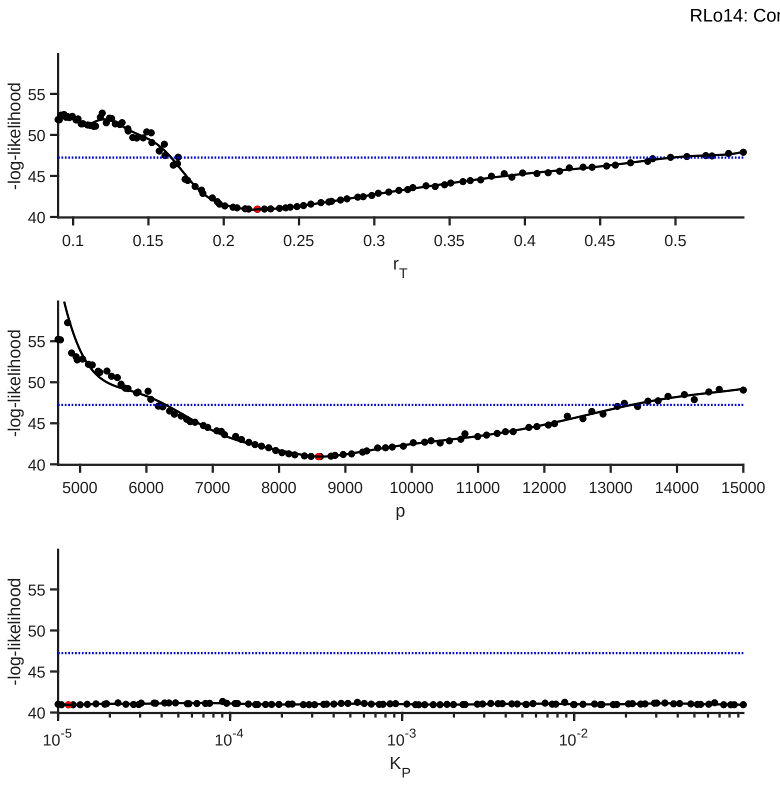
RLo14: Control (SS)
-log-likelihood
r_T
m
p
K_B
K_P
σ

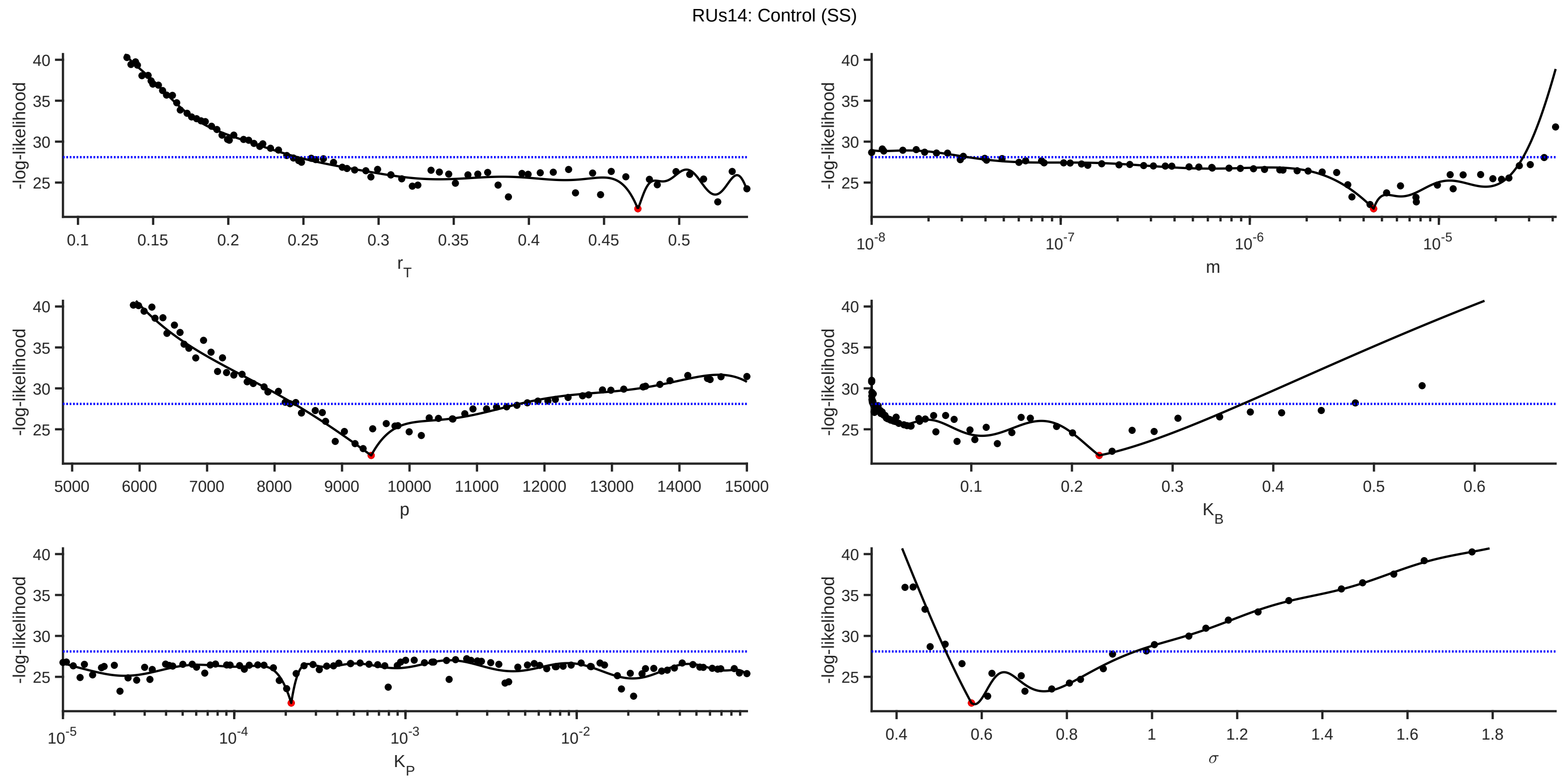
RUs14: Control (SS)
-log-likelihood
r_T
m
p
K_B
K_P
σ

1.3 Antigen presenting cell effector cell source model

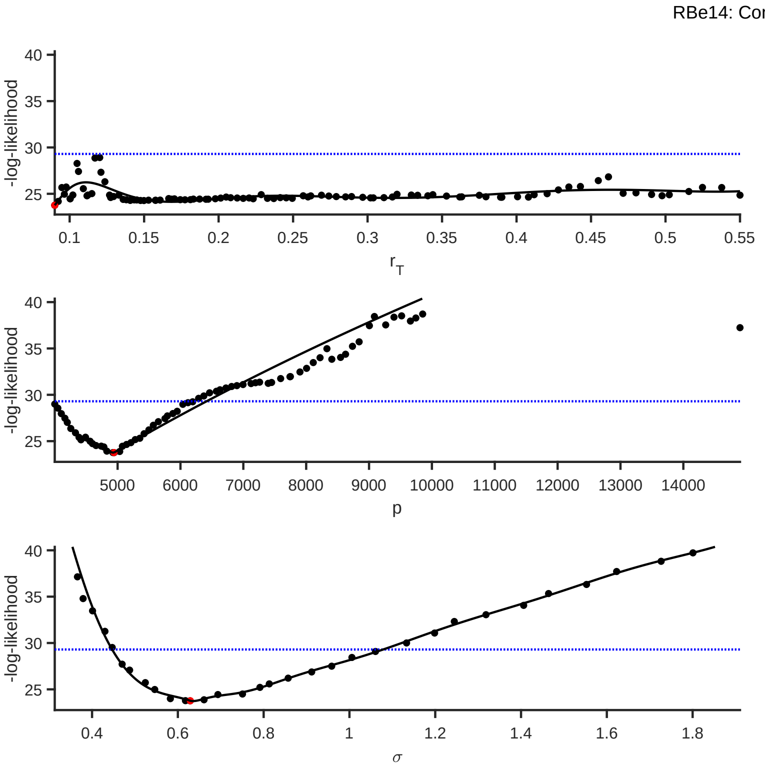

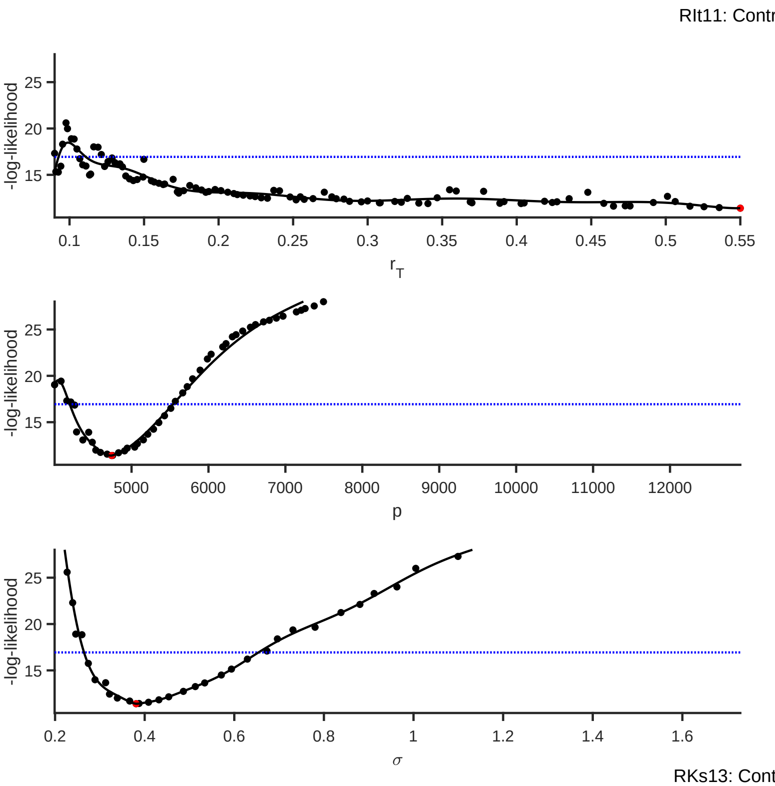
RIt11: Control (APCS)
-log-likelihood
r_T
m
10^{-7}
p
K_B
σ

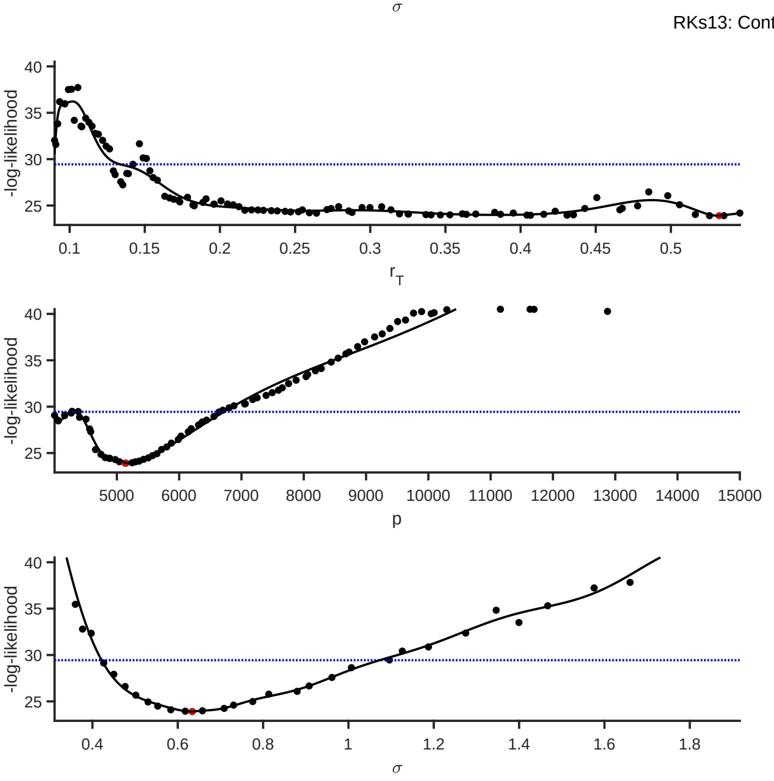
RKs13: Control (APCS)
-log-likelihood
r_T
m
10^{-7}
p
K_B
σ

RSy13: Control (APCS)

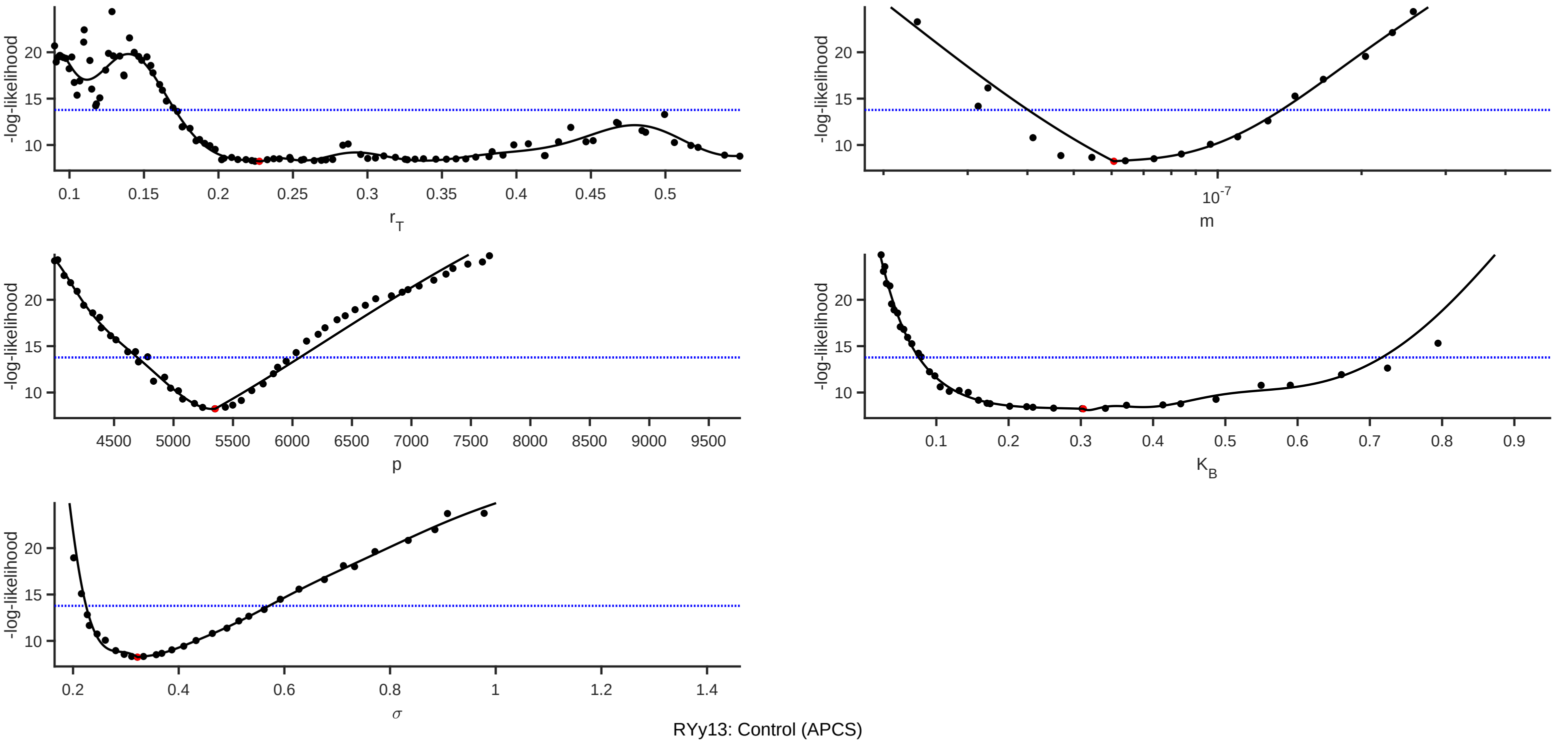

RYy13: Control (APCS)

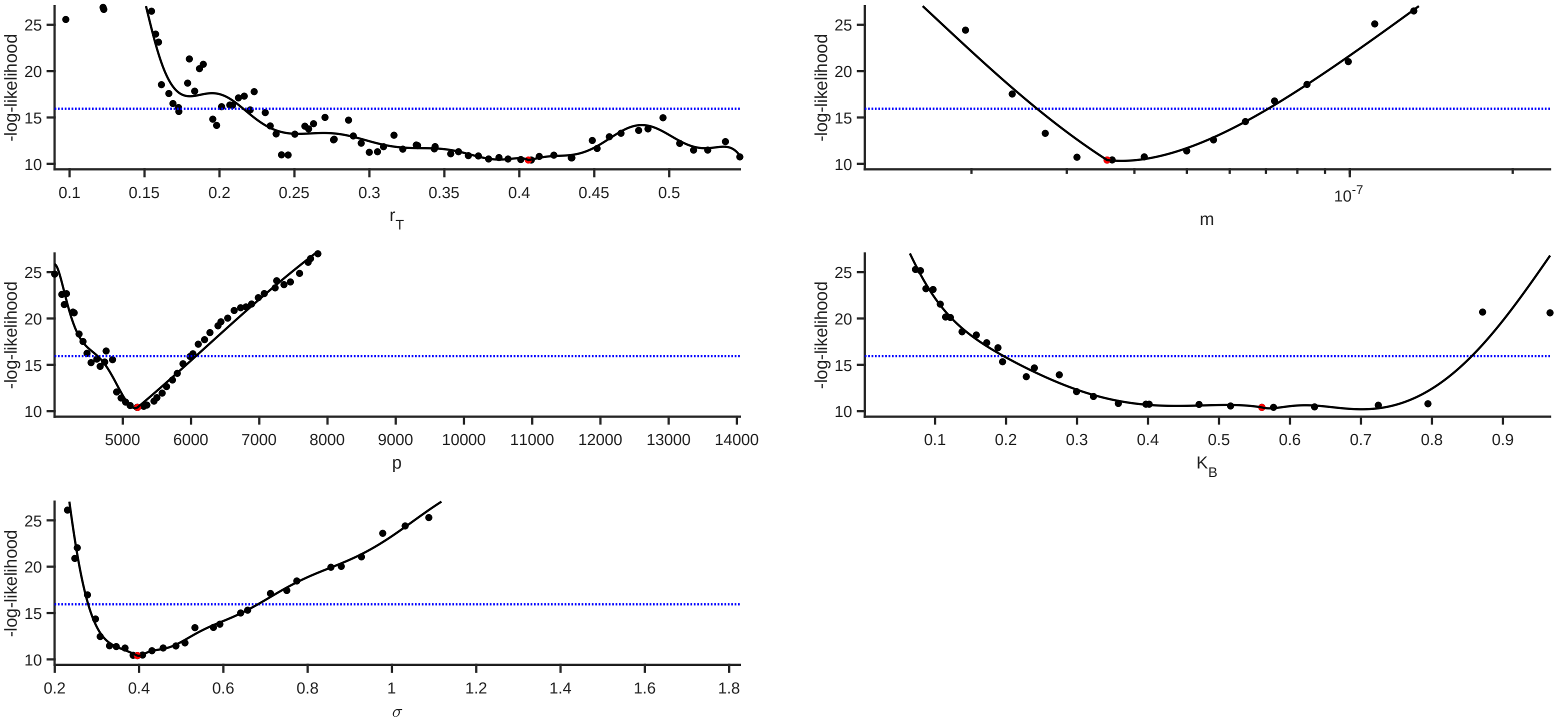

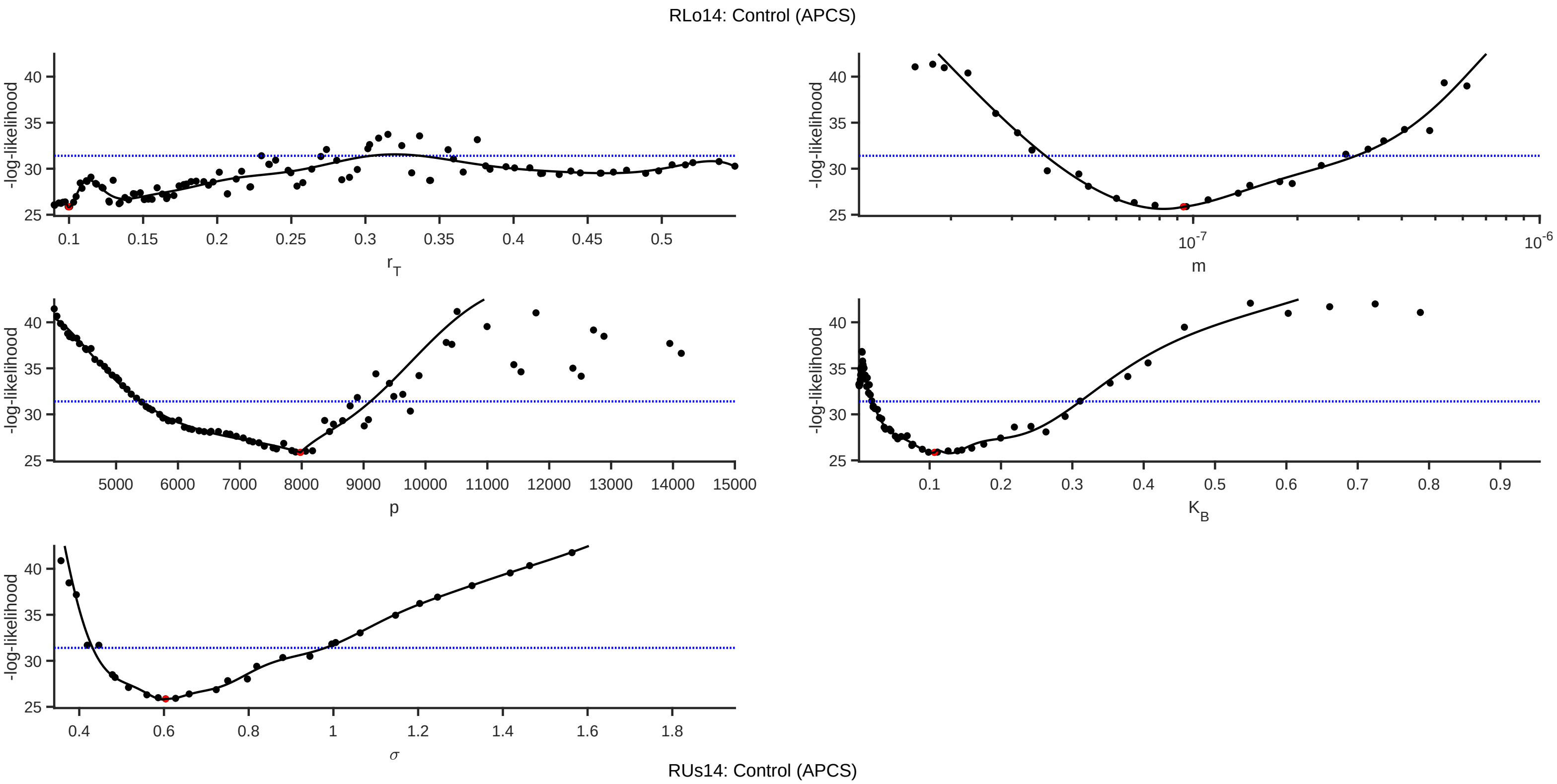
RLo14: Control (APCS)
-log-likelihood
r_T
m
p
K_B
σ

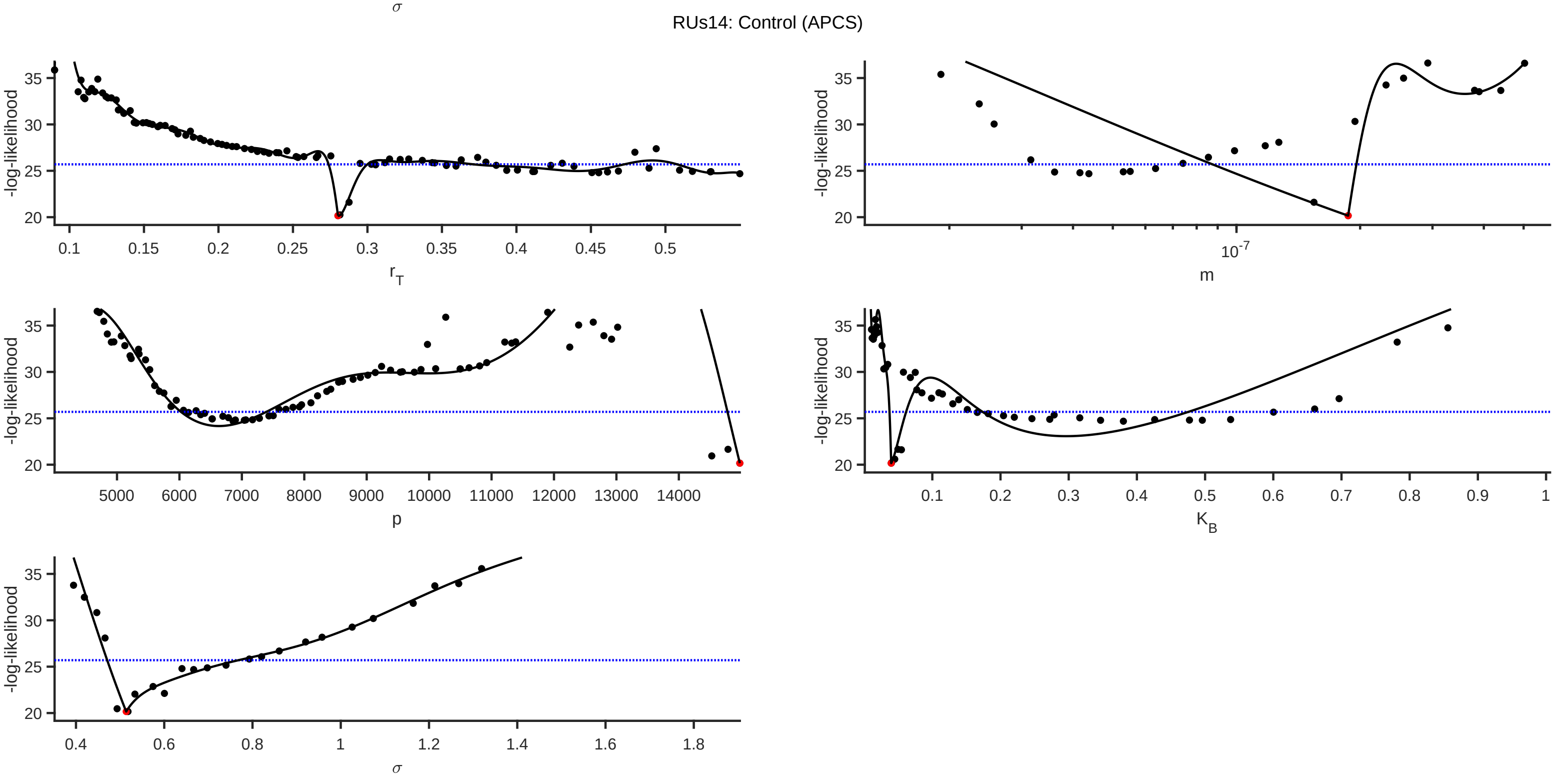
RUs14: Control (APCS)
-log-likelihood
r_T
m
p
K_B
σ

2 Sensitivity anlaysis: Control macaques

2.1 Baseline effector cell source model

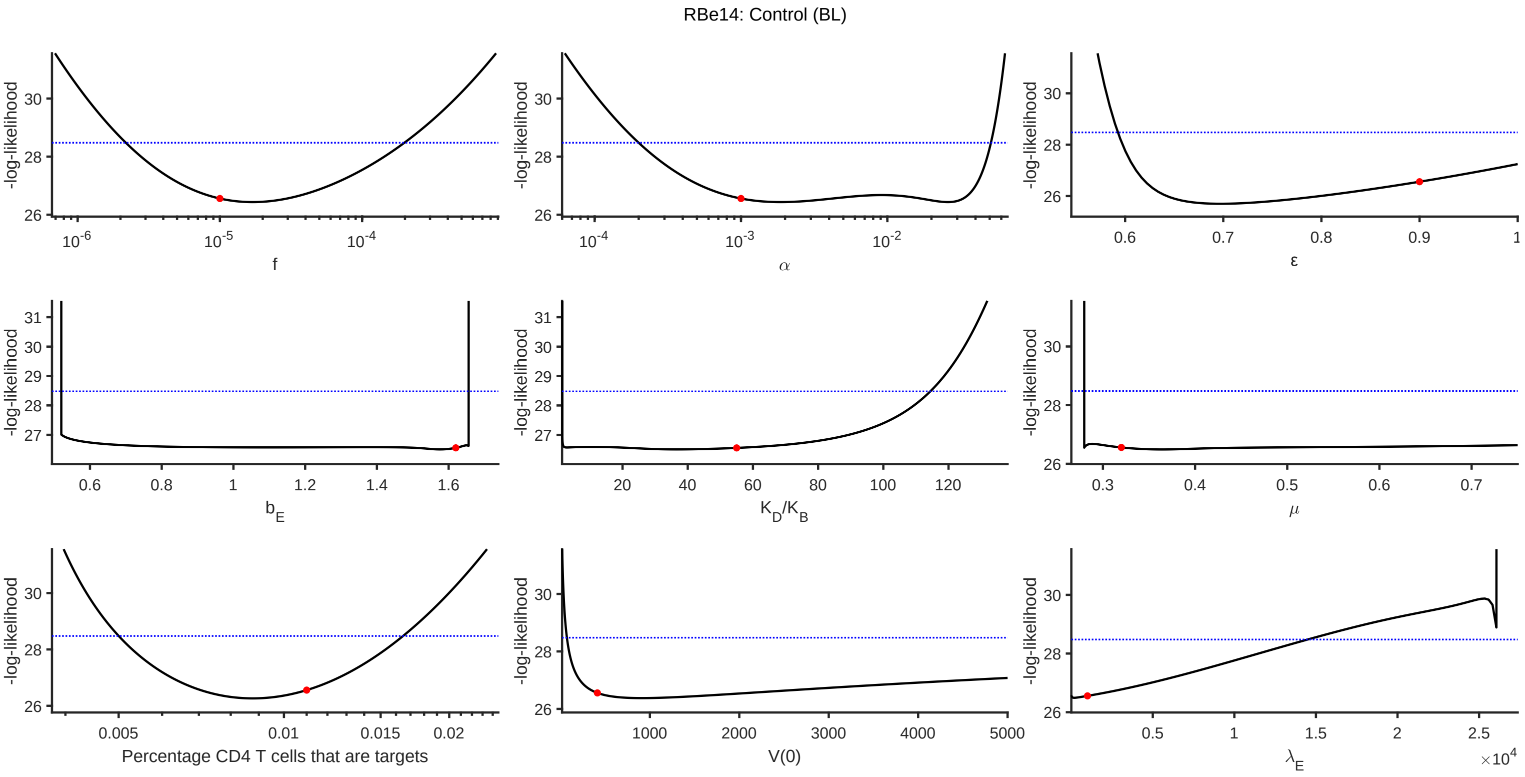

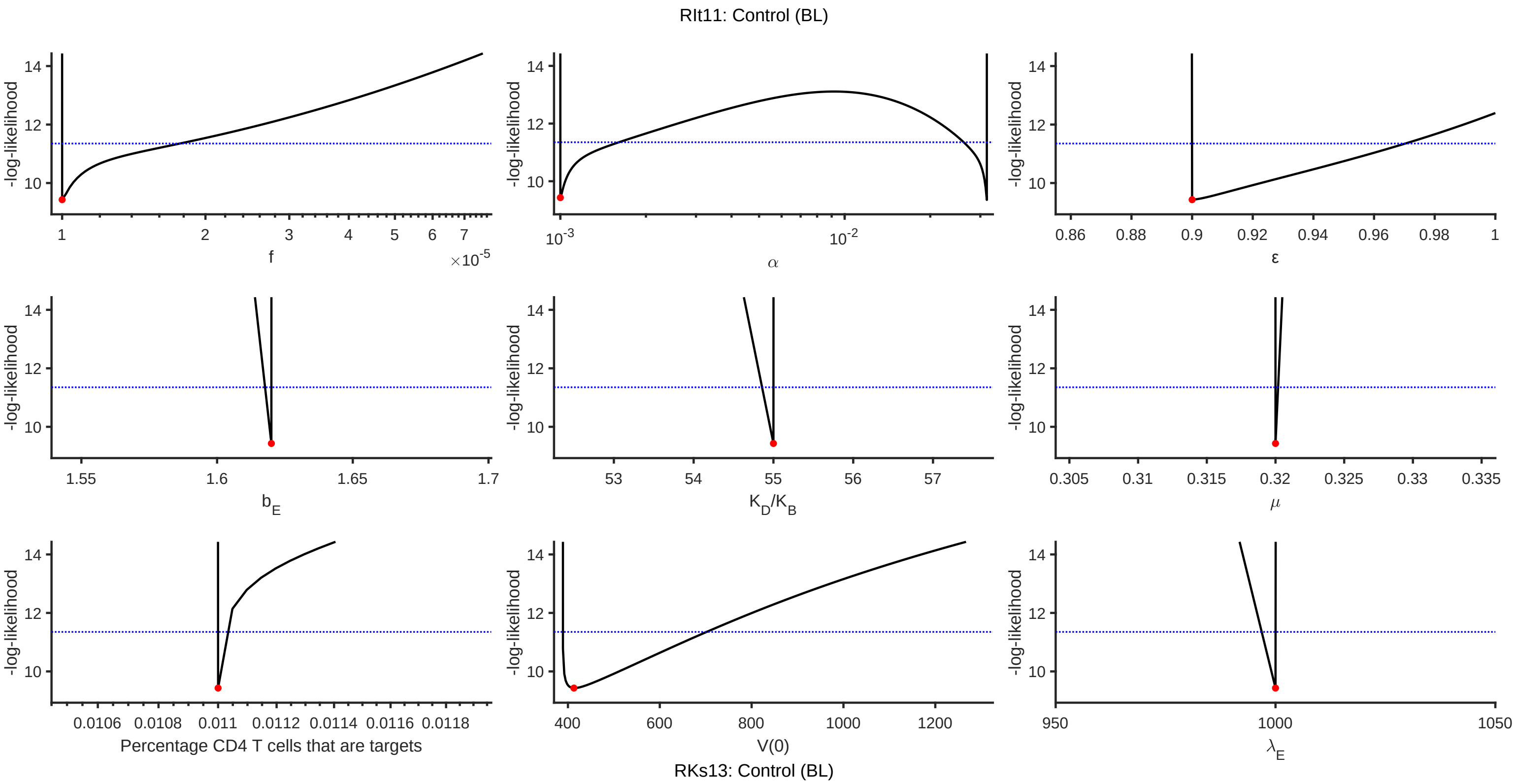
RIt11: Control (BL)
-log-likelihood
f
×10^-5
α
ε
b_E
K_D/K_B
μ
Percentage CD4 T cells that are targets
V(0)
λ_E

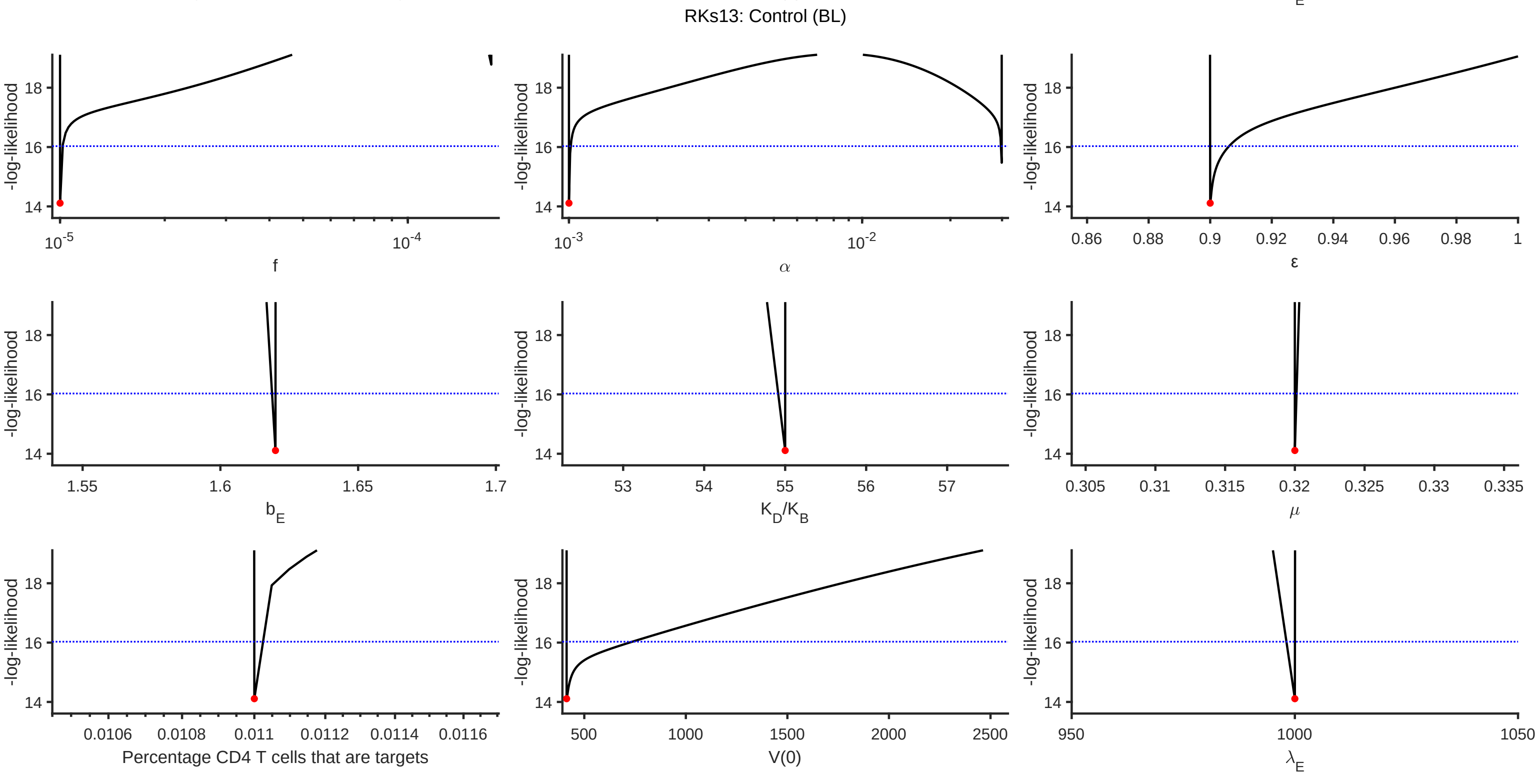
RKs13: Control (BL)
-log-likelihood
f
α
ε
b_E
K_D/K_B
μ
Percentage CD4 T cells that are targets
V(0)
λ_E

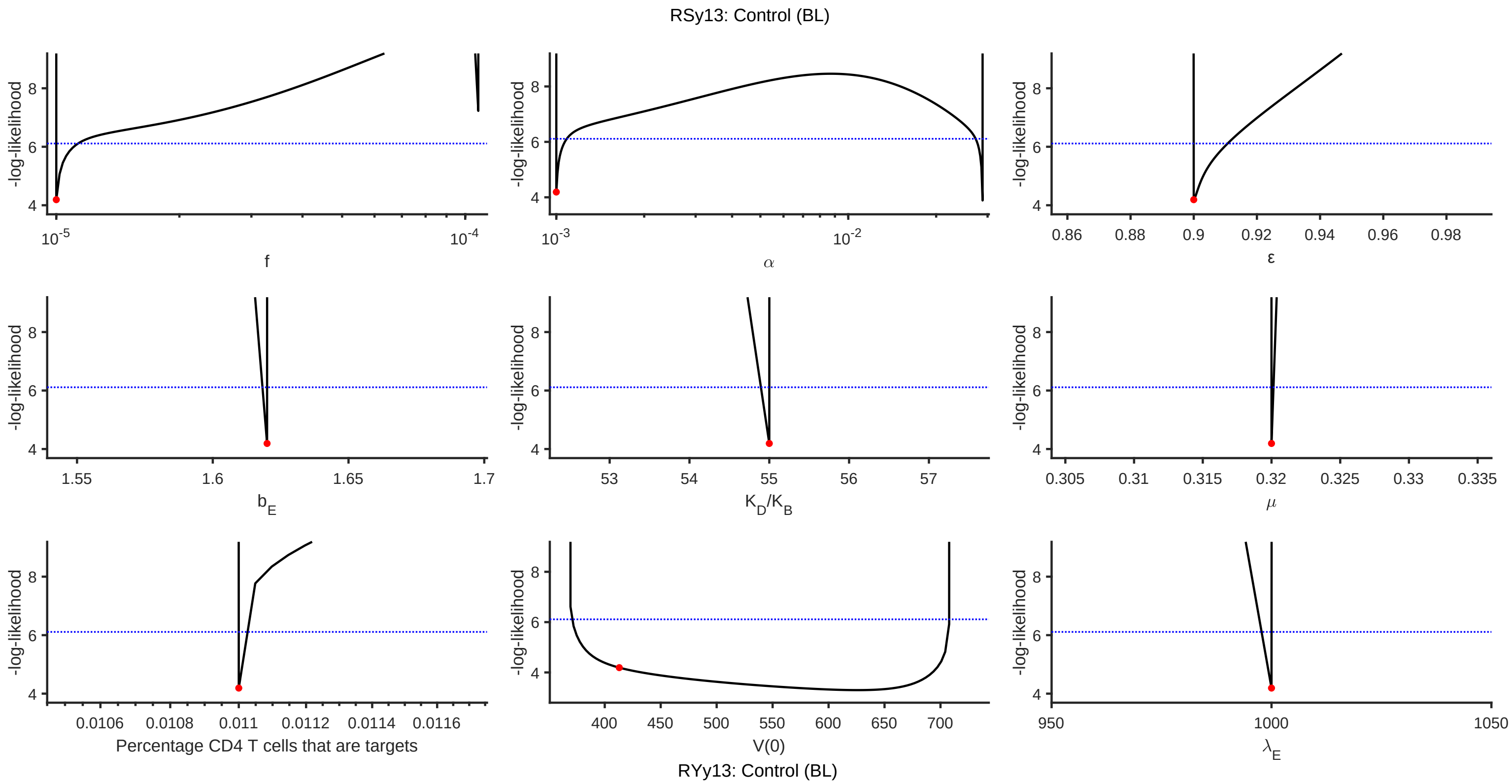
RSy13: Control (BL)
-log-likelihood
f
α
ε
b_E
K_D/K_B
μ
Percentage CD4 T cells that are targets
V(0)
λ_E

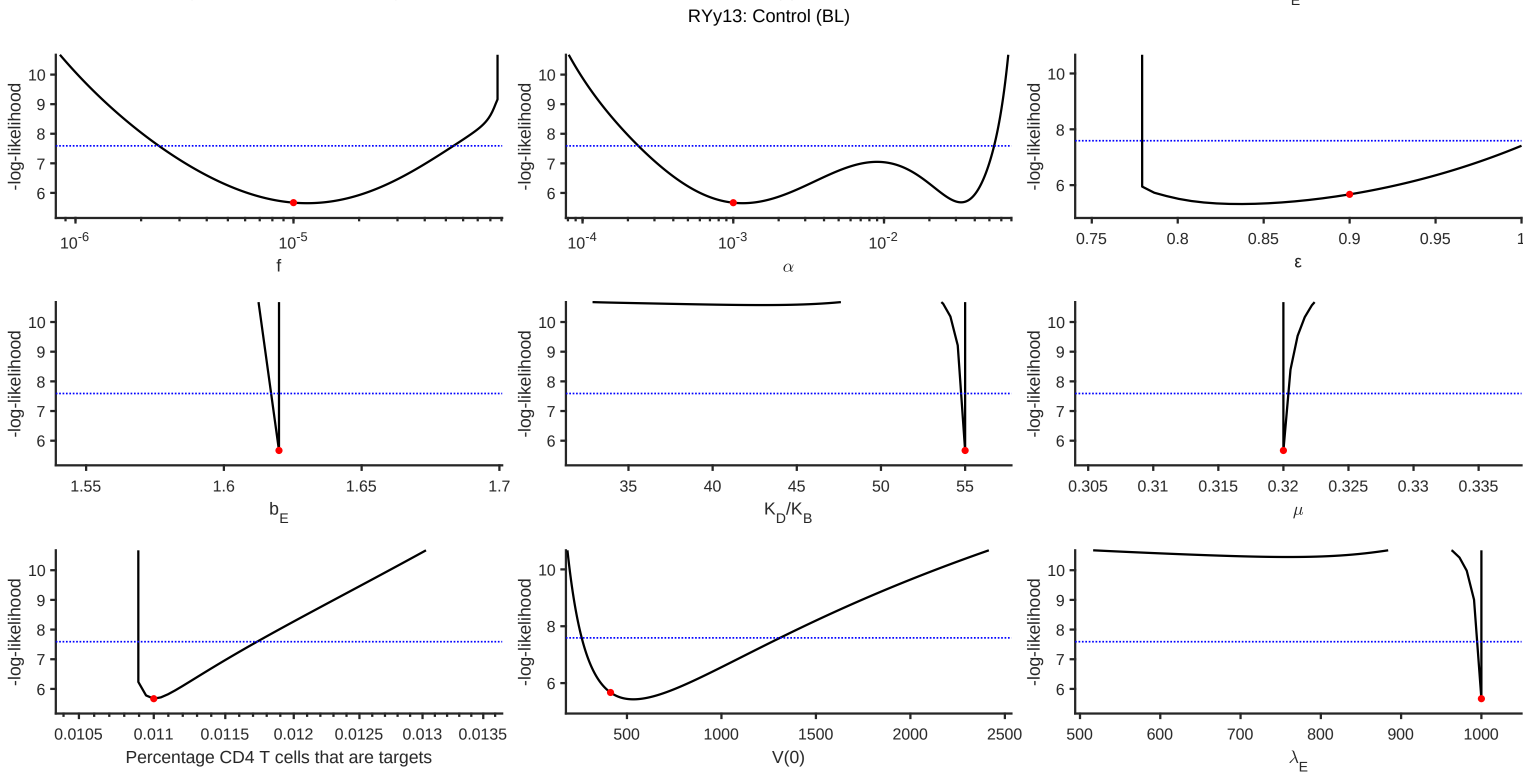
RYy13: Control (BL)
-log-likelihood
f
α
ε
b_E
K_D/K_B
μ
Percentage CD4 T cells that are targets
V(0)
λ_E

RLo14: Control (BL)

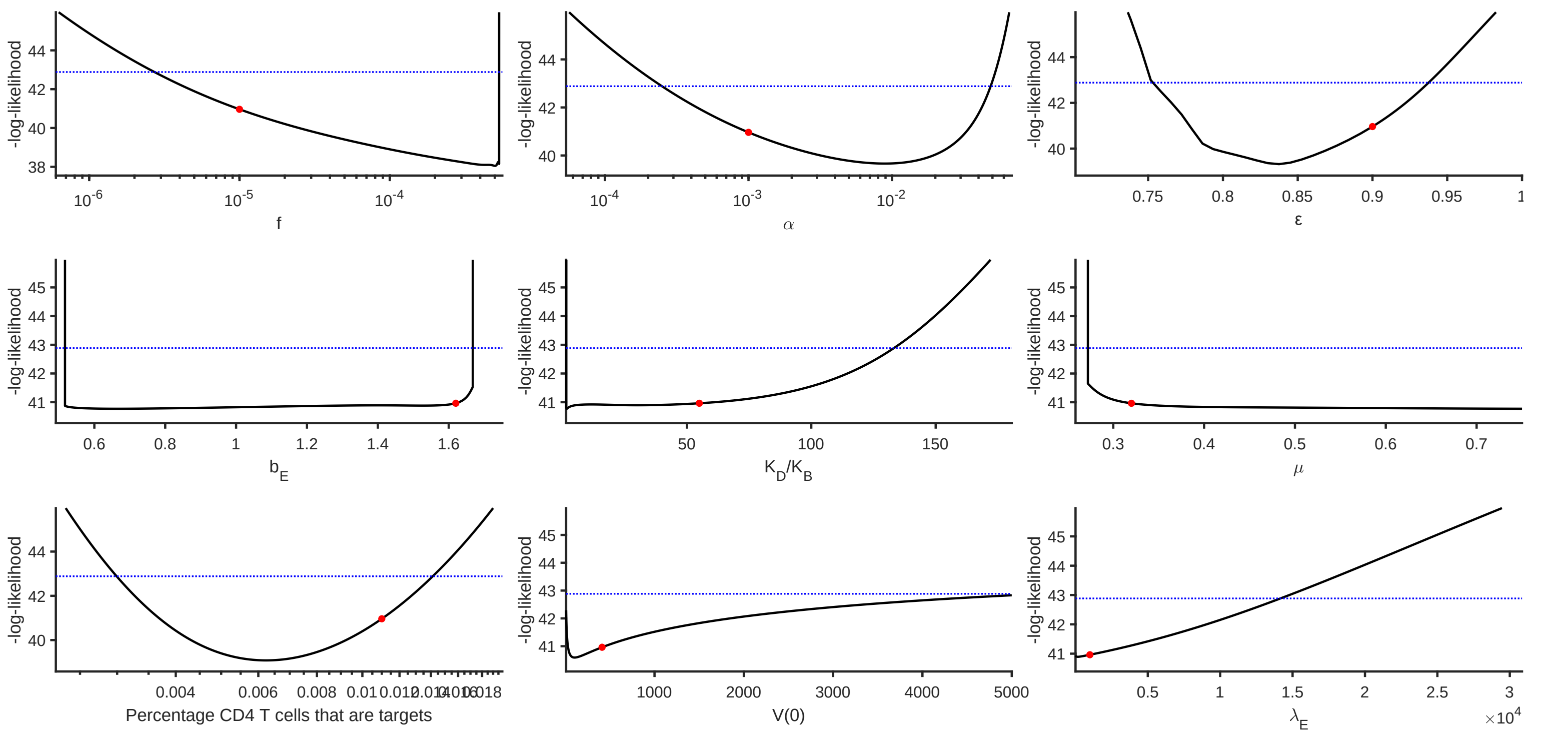

RUs14: Control (BL)

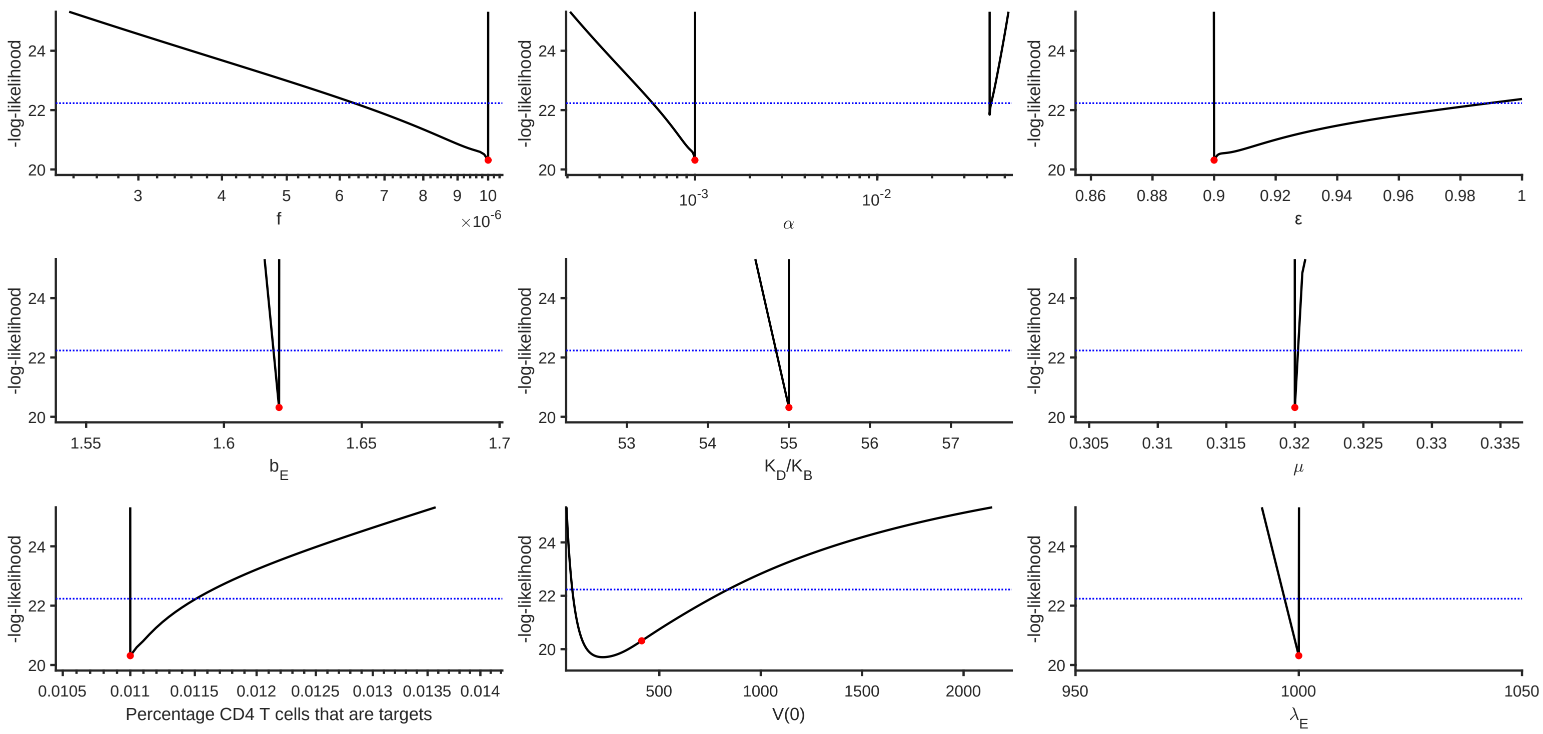

2.2 Saturated effector cell source model

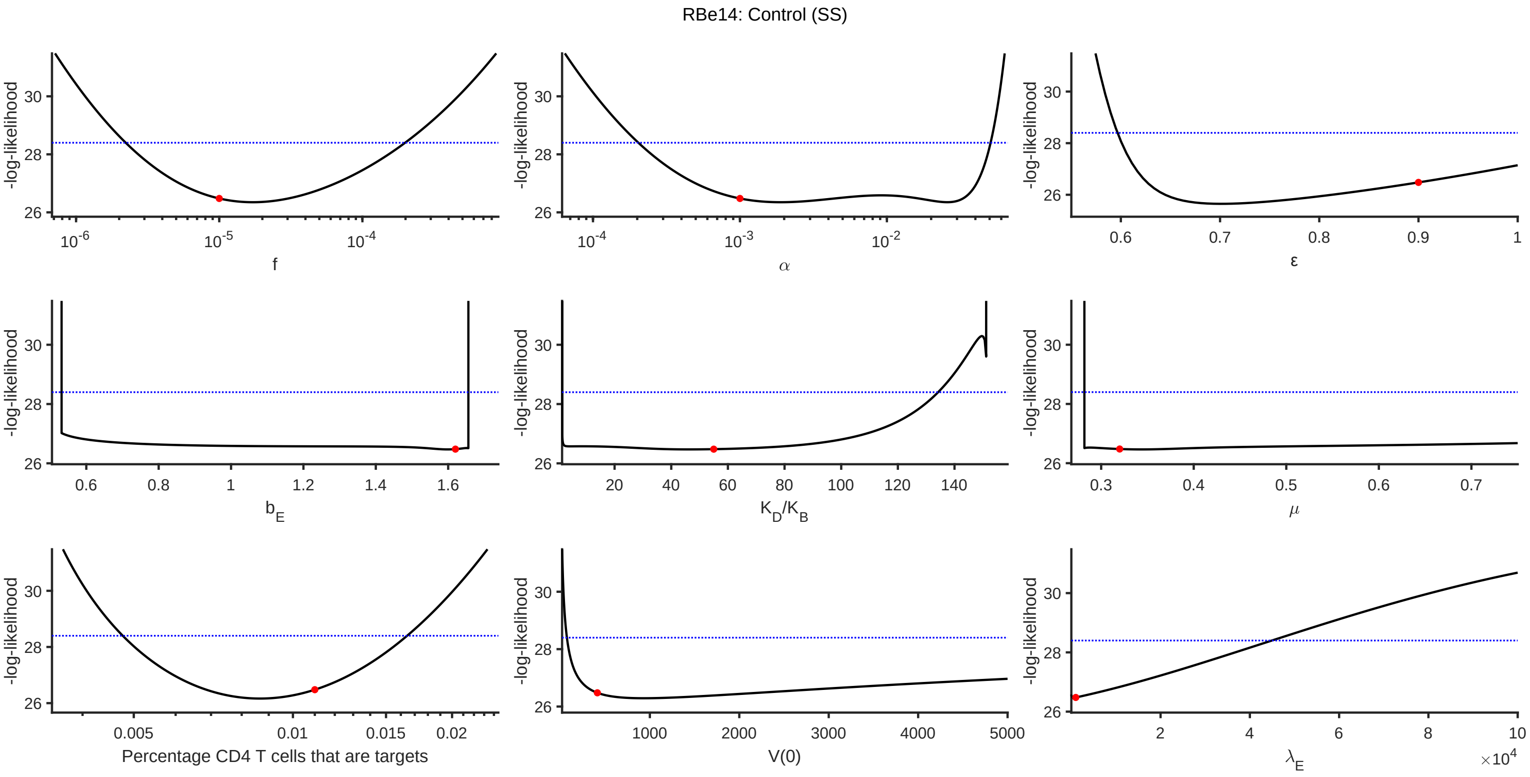

RIt11: Control (SS)

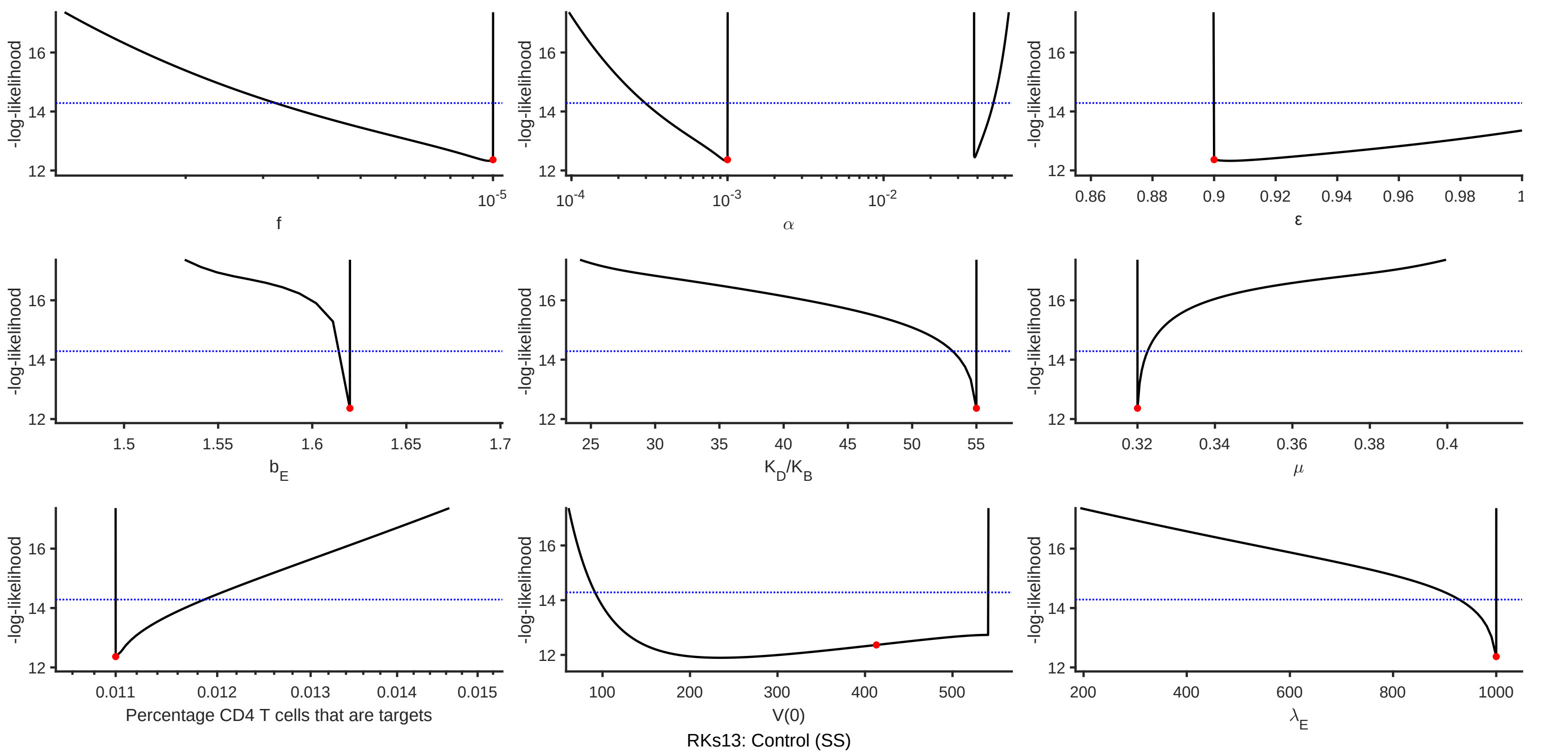

RKs13: Control (SS)

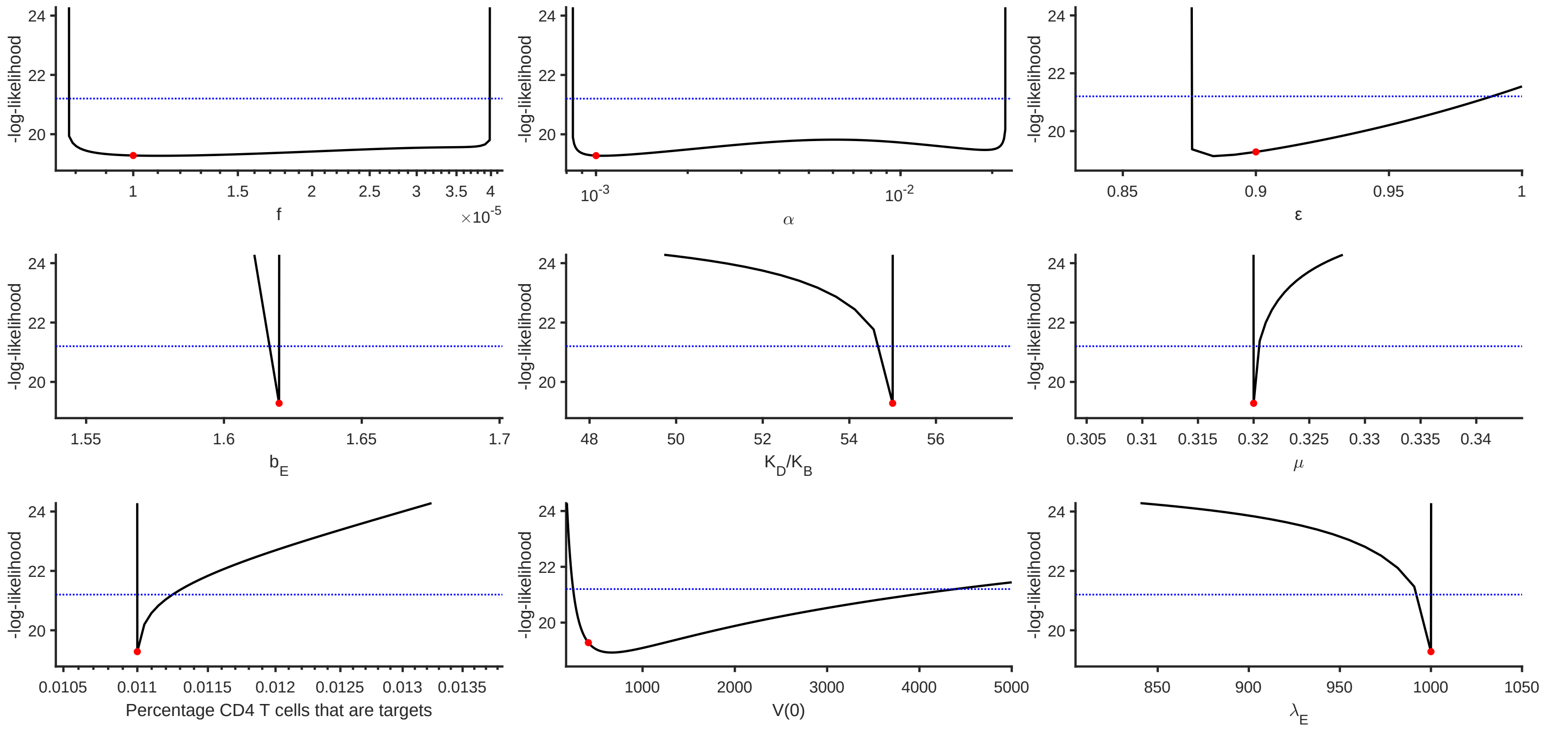

RSy13: Control (SS)

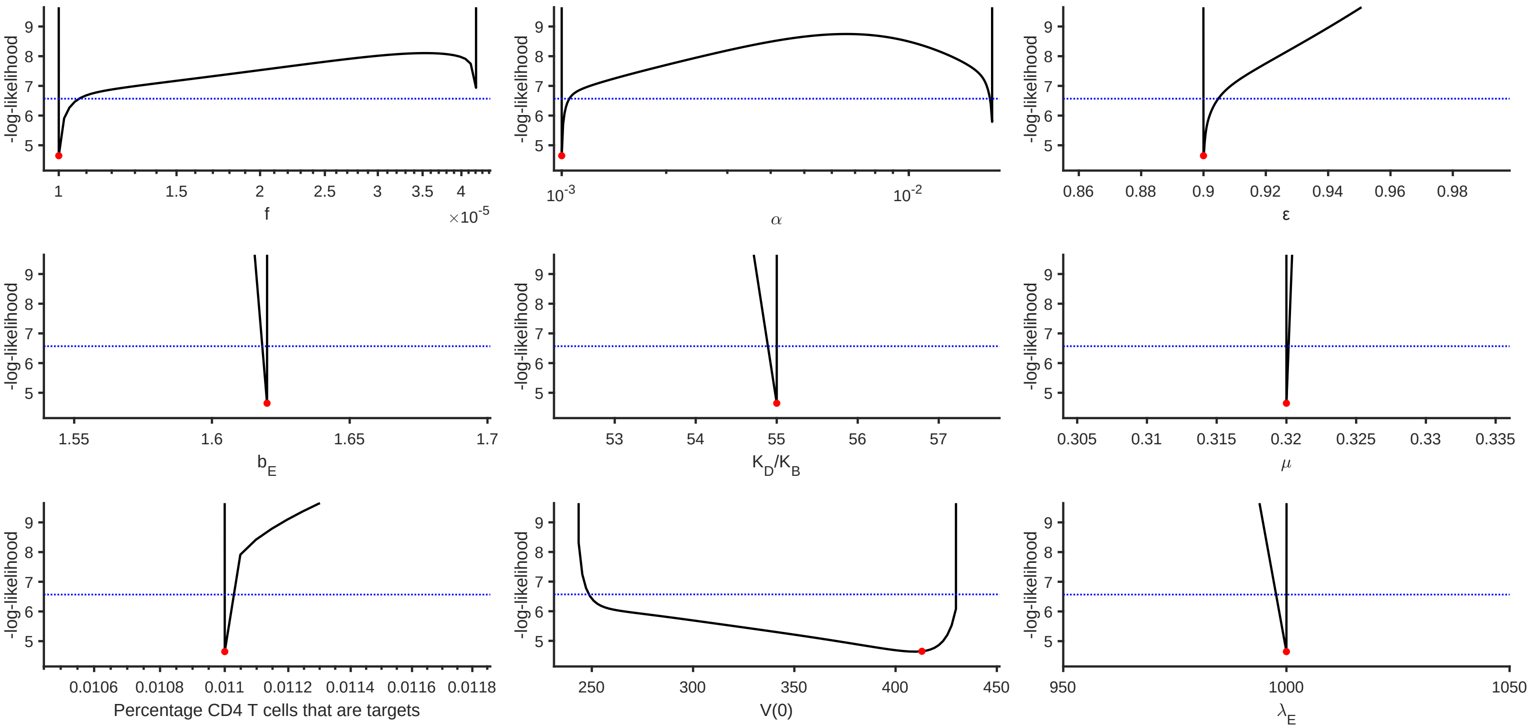

RYy13: Control (SS)

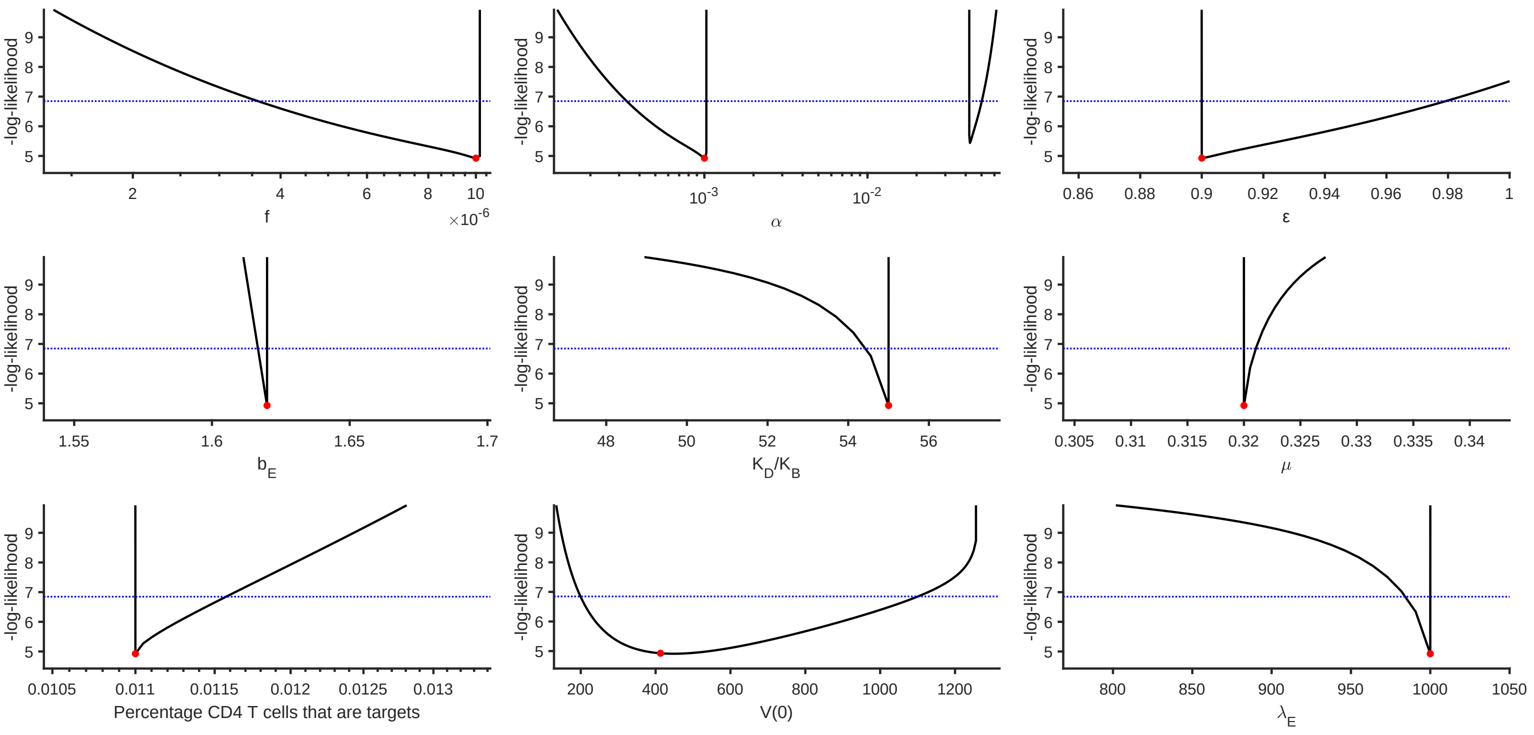

RLo14: Control (SS)

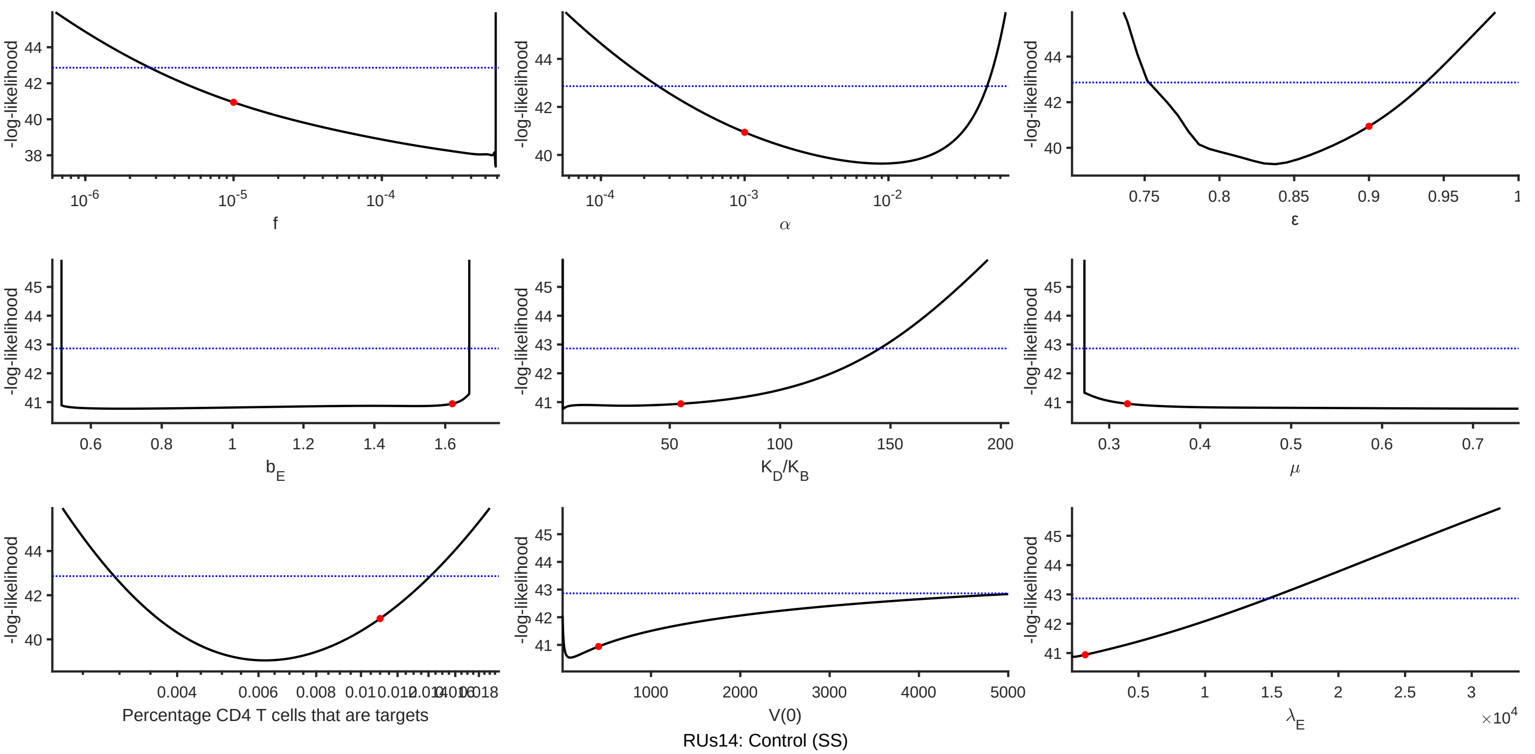

RUs14: Control (SS)

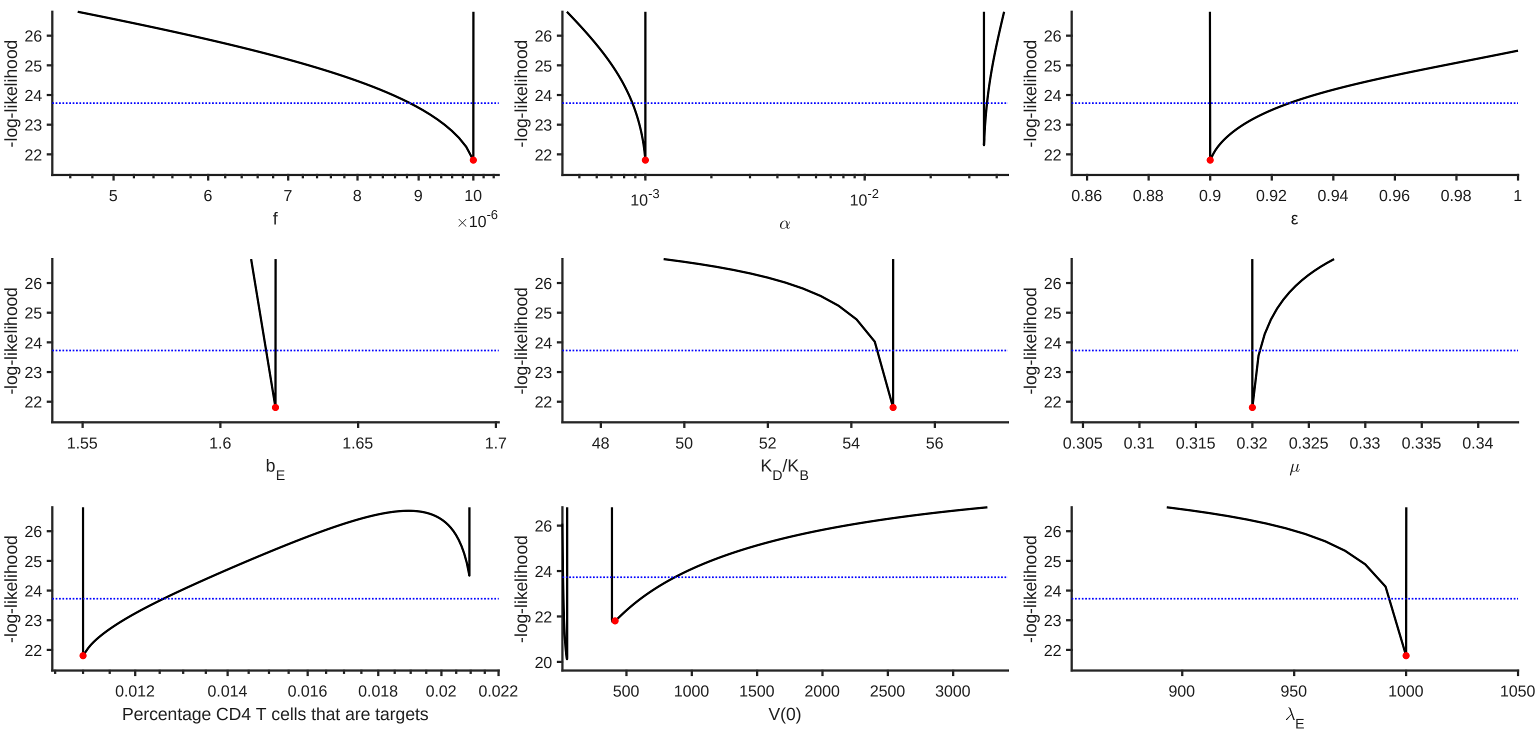

2.3 Antigen presenting cell effector cell source model

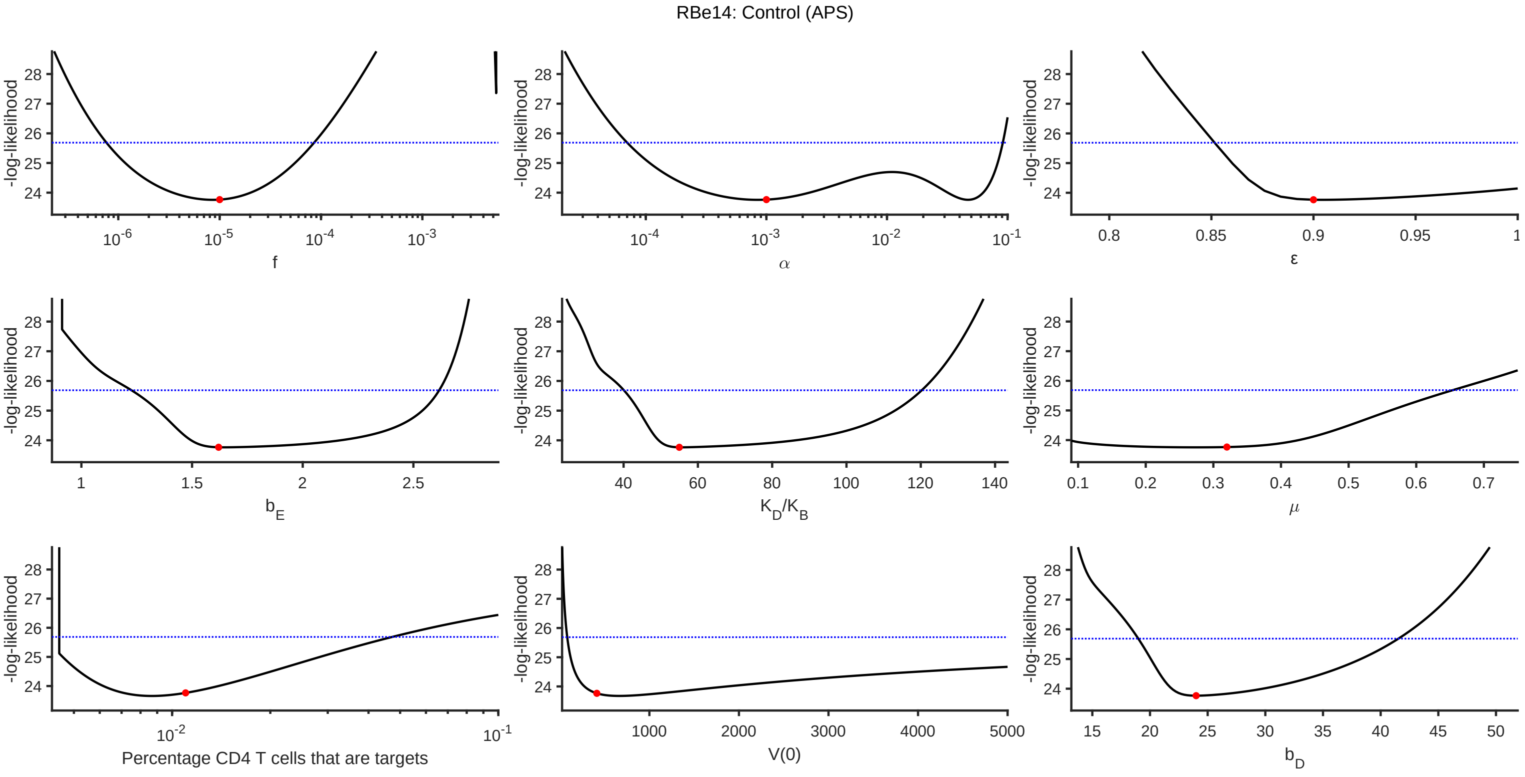

RIt11: Control (APS)

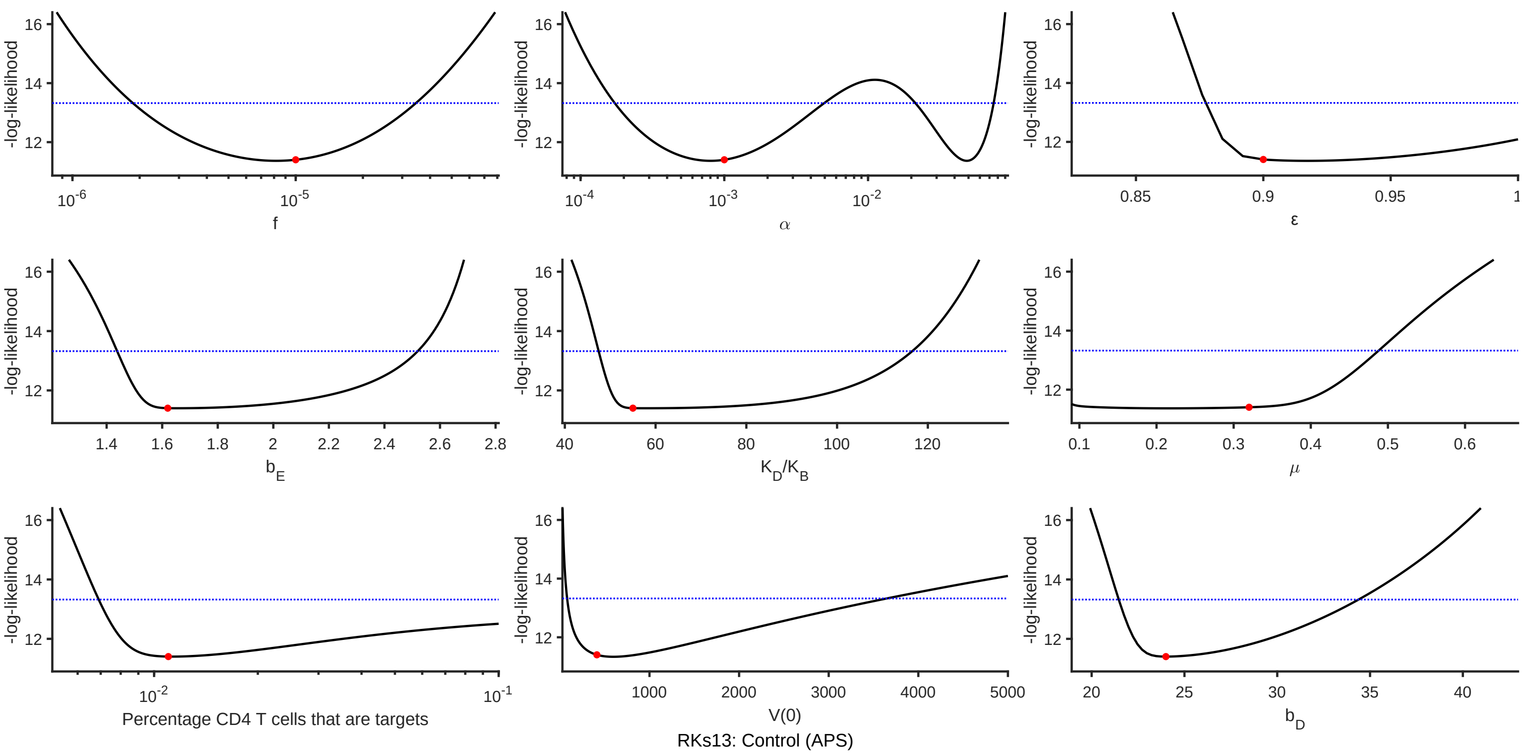

RKs13: Control (APS)

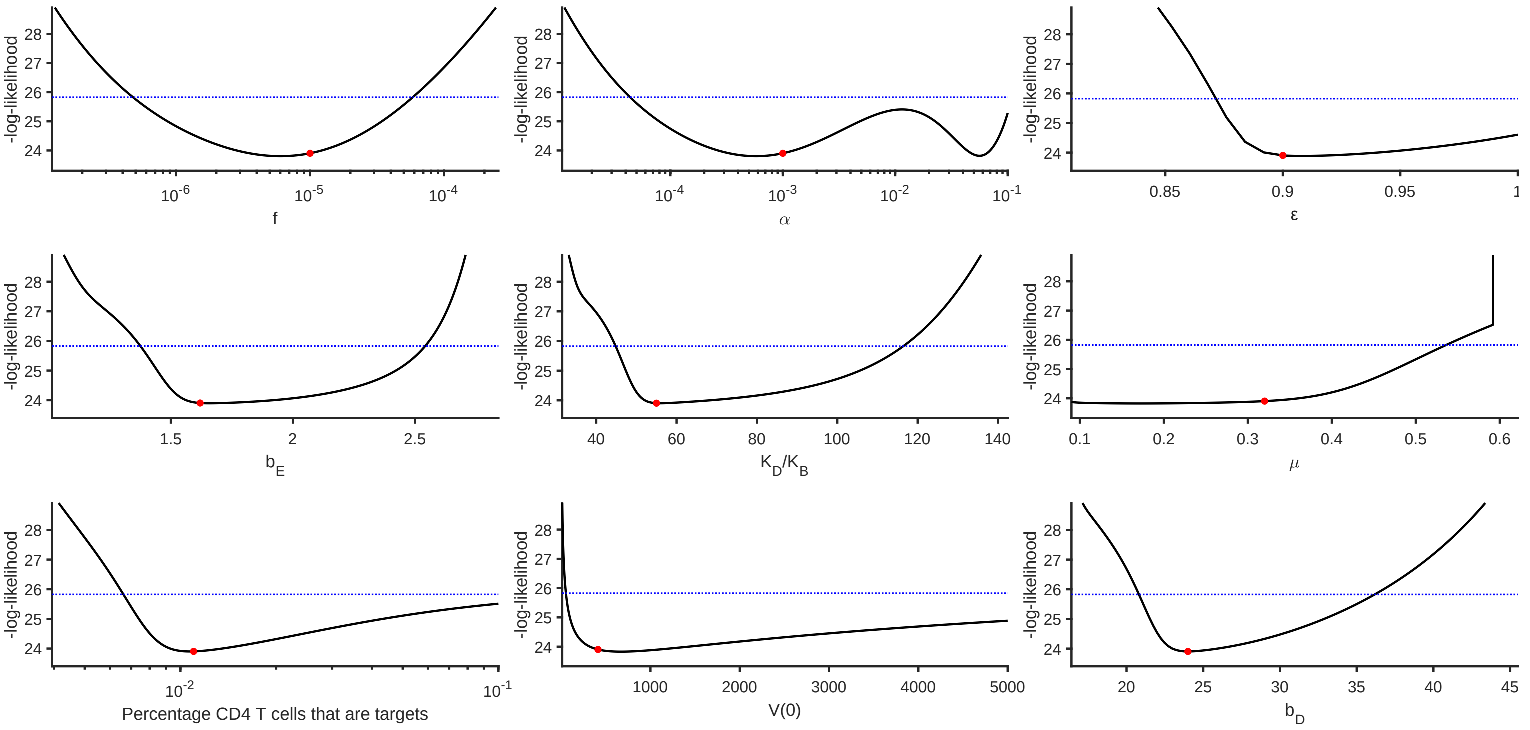

RSy13: Control (APS)

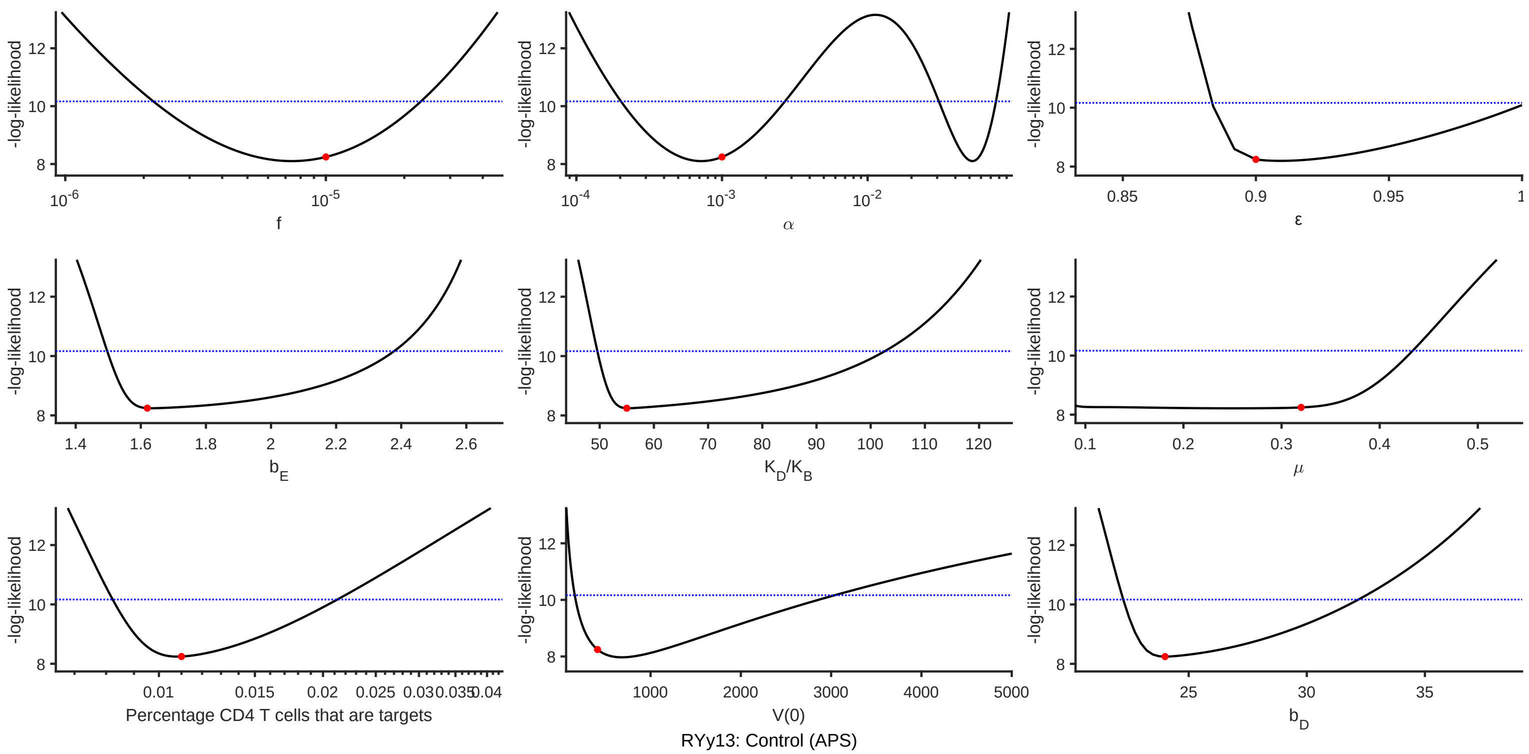

RYy13: Control (APS)

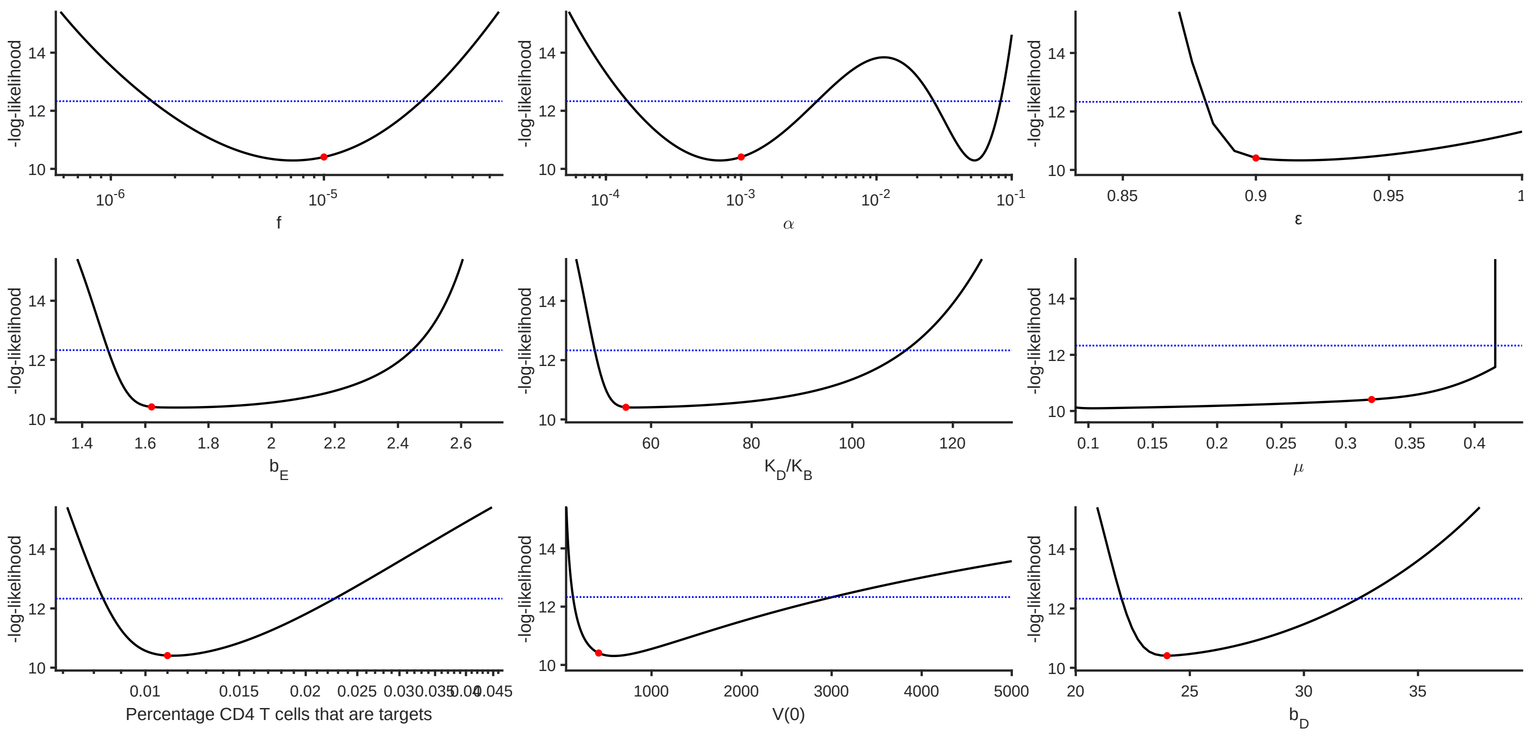

RLo14: Control (APS)

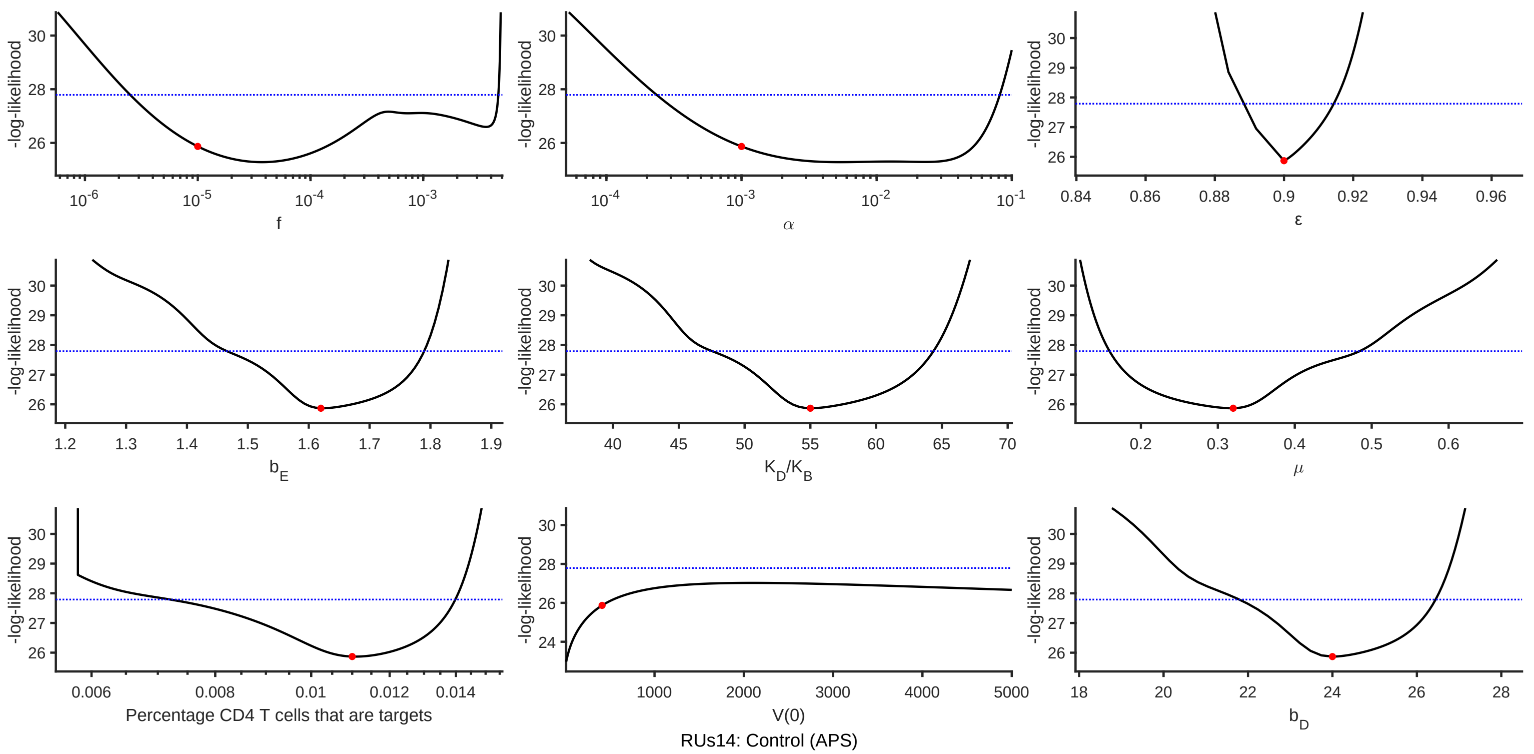

RUs14: Control (APS)

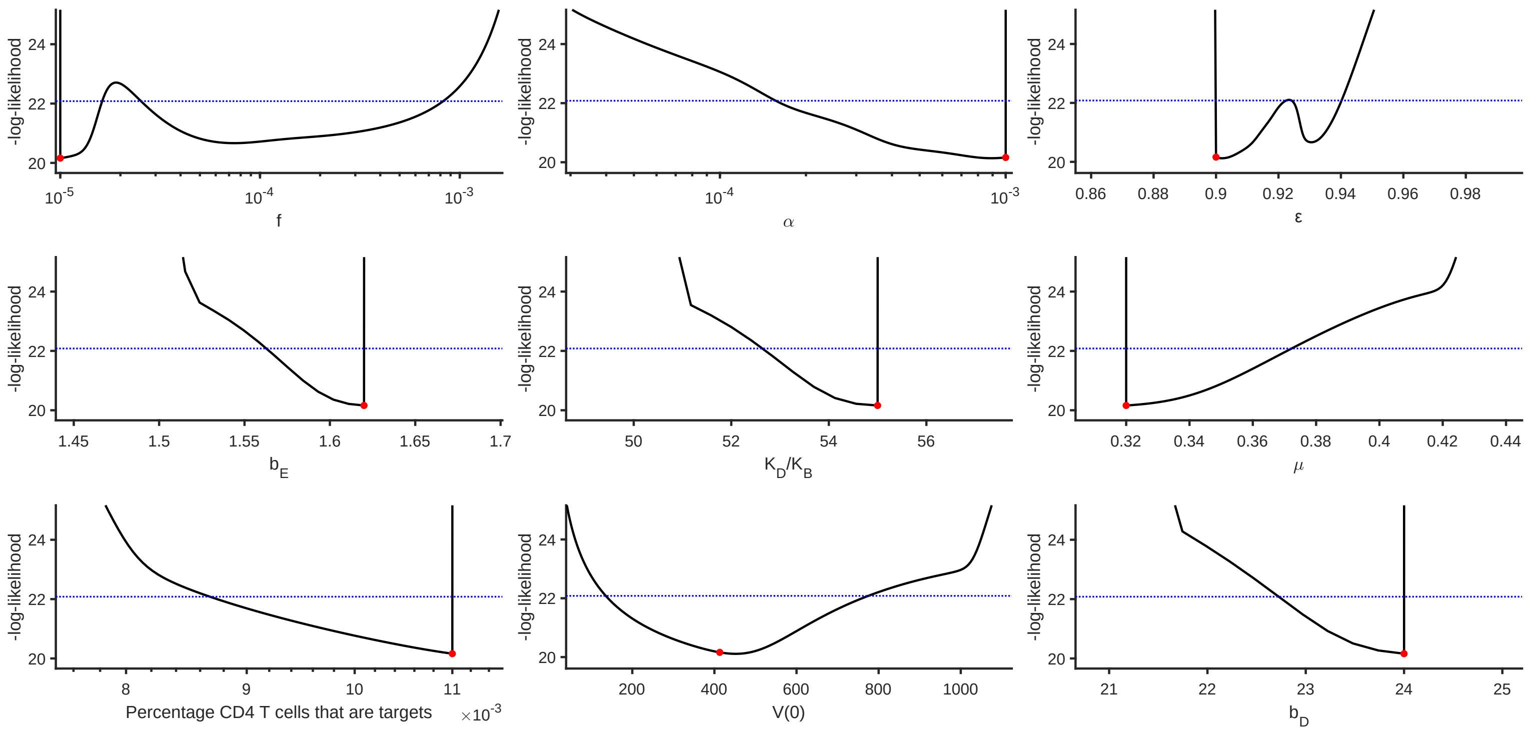

3 Likelihood profiles: Increased viral clearance

3.1 Baseline effector cell source model

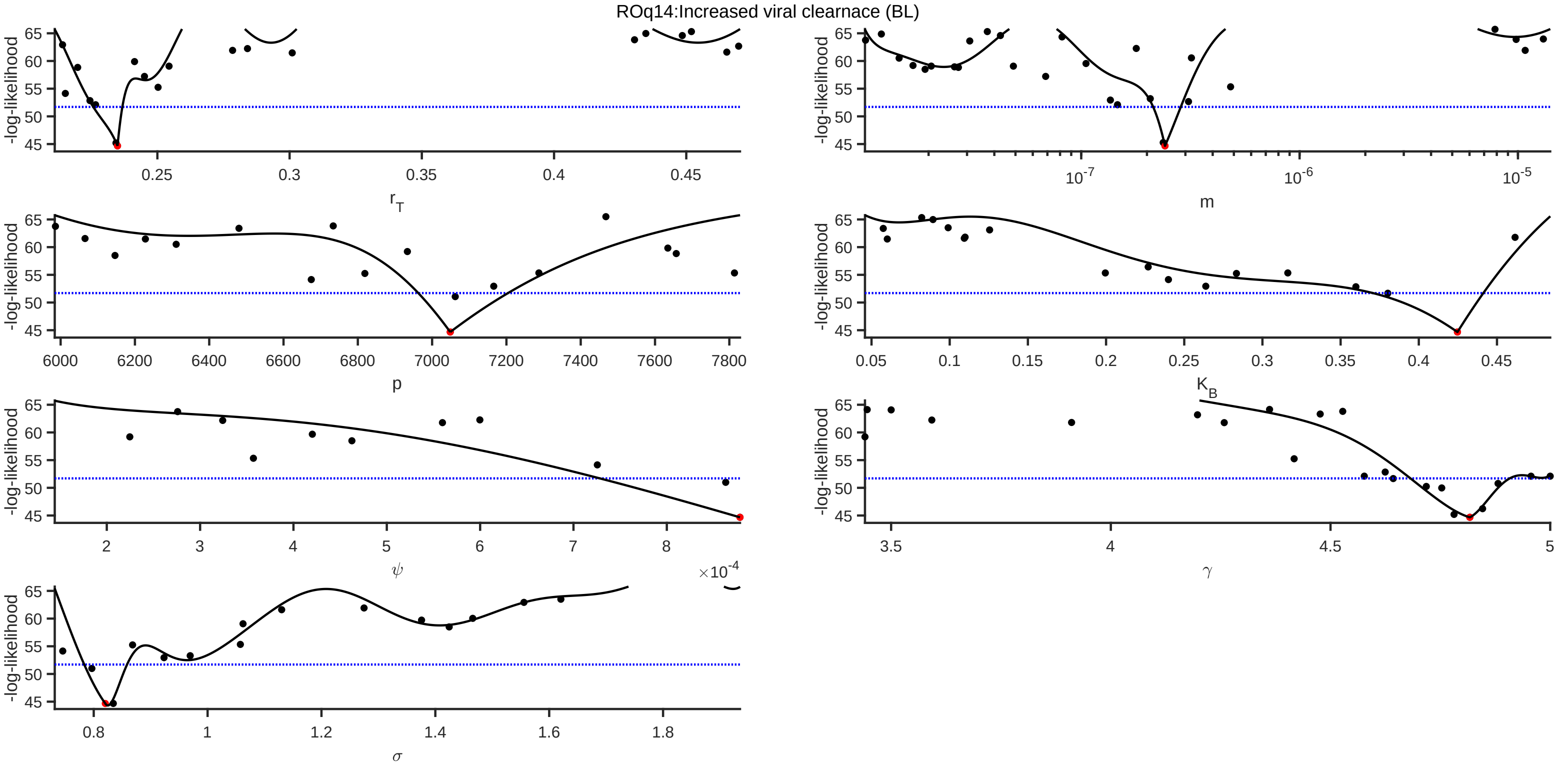

RDa15:Increased viral clearnace (BL)

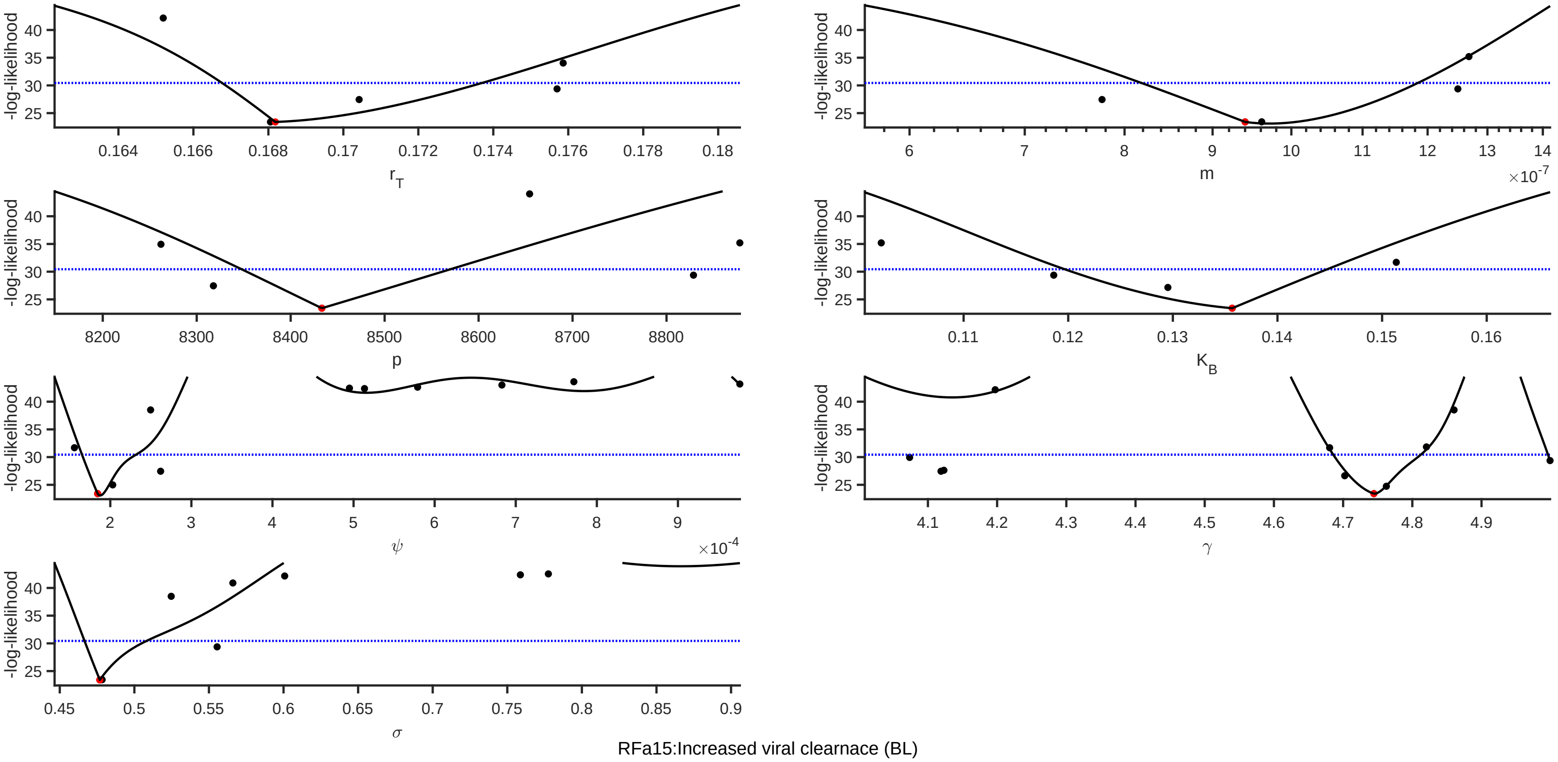

RFa15:Increased viral clearnace (BL)

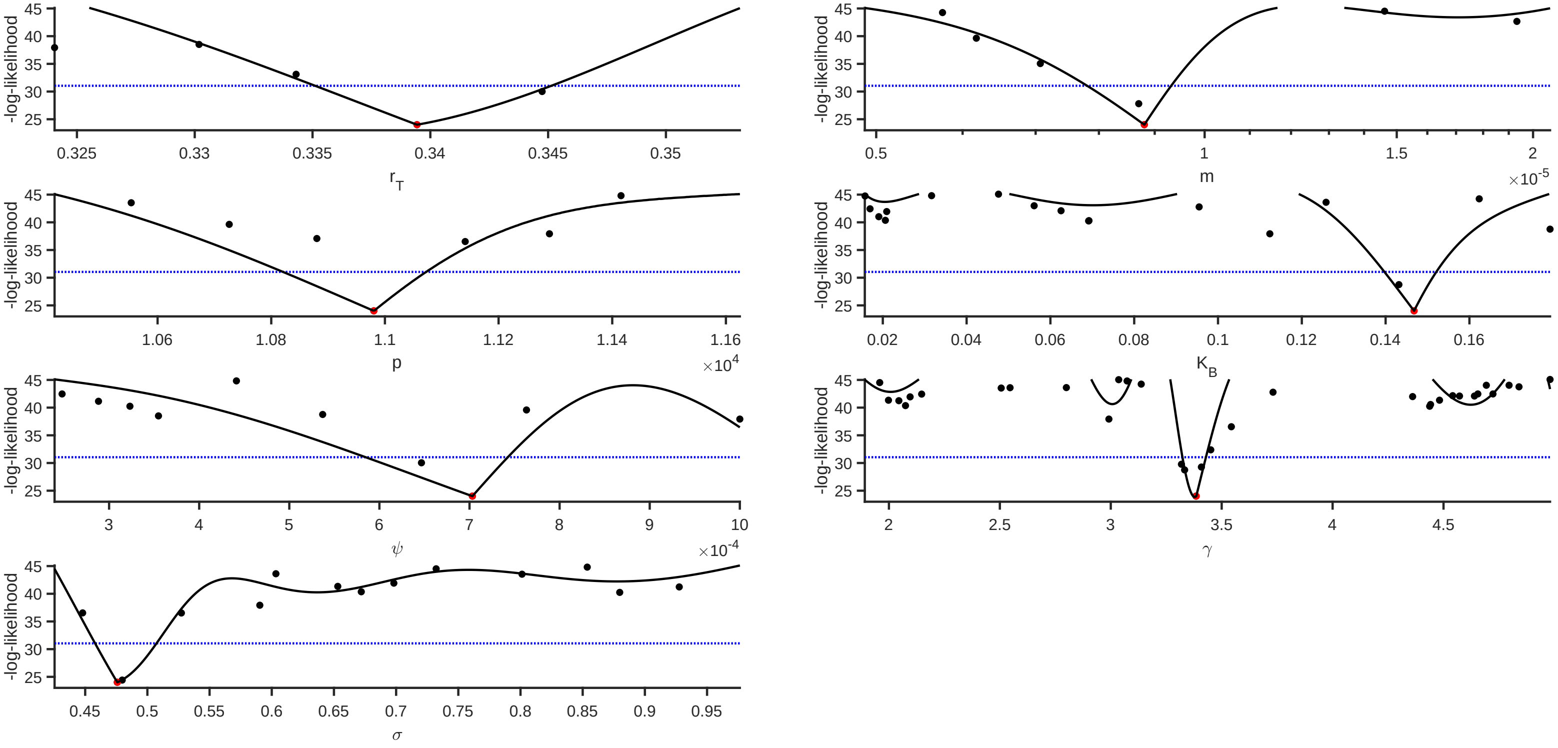

RLn12:Increased viral clearnace (BL)

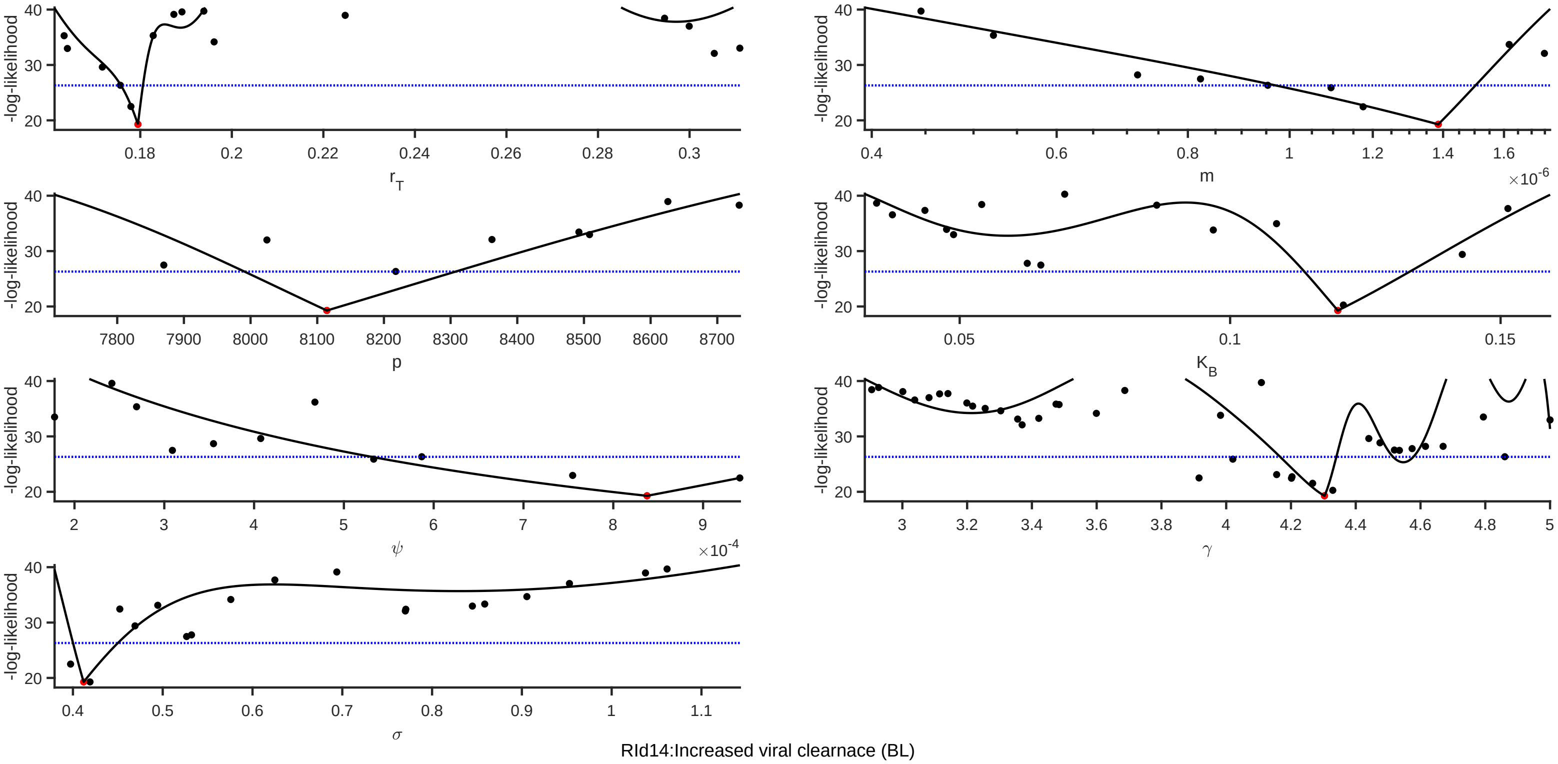

RId14:Increased viral clearnace (BL)

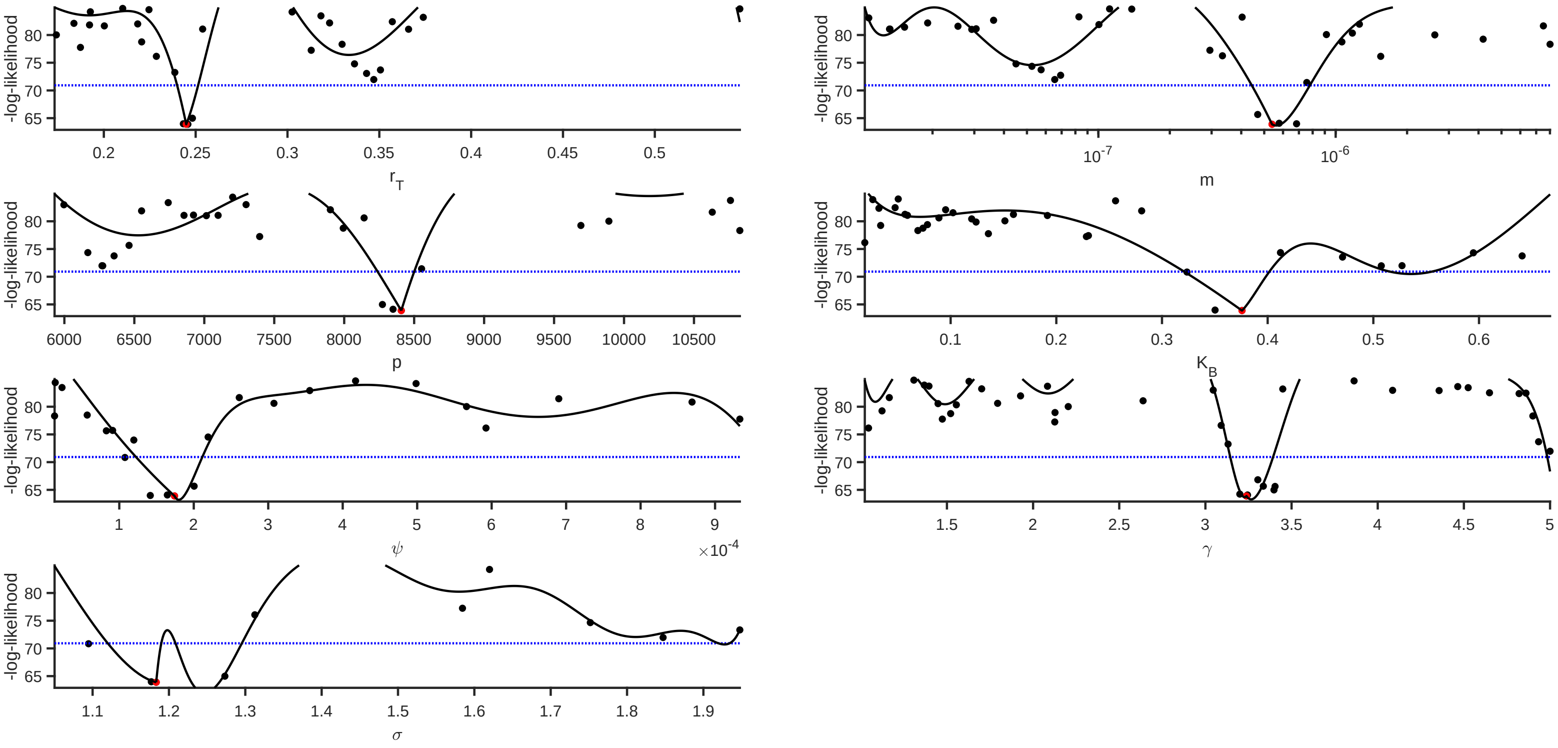

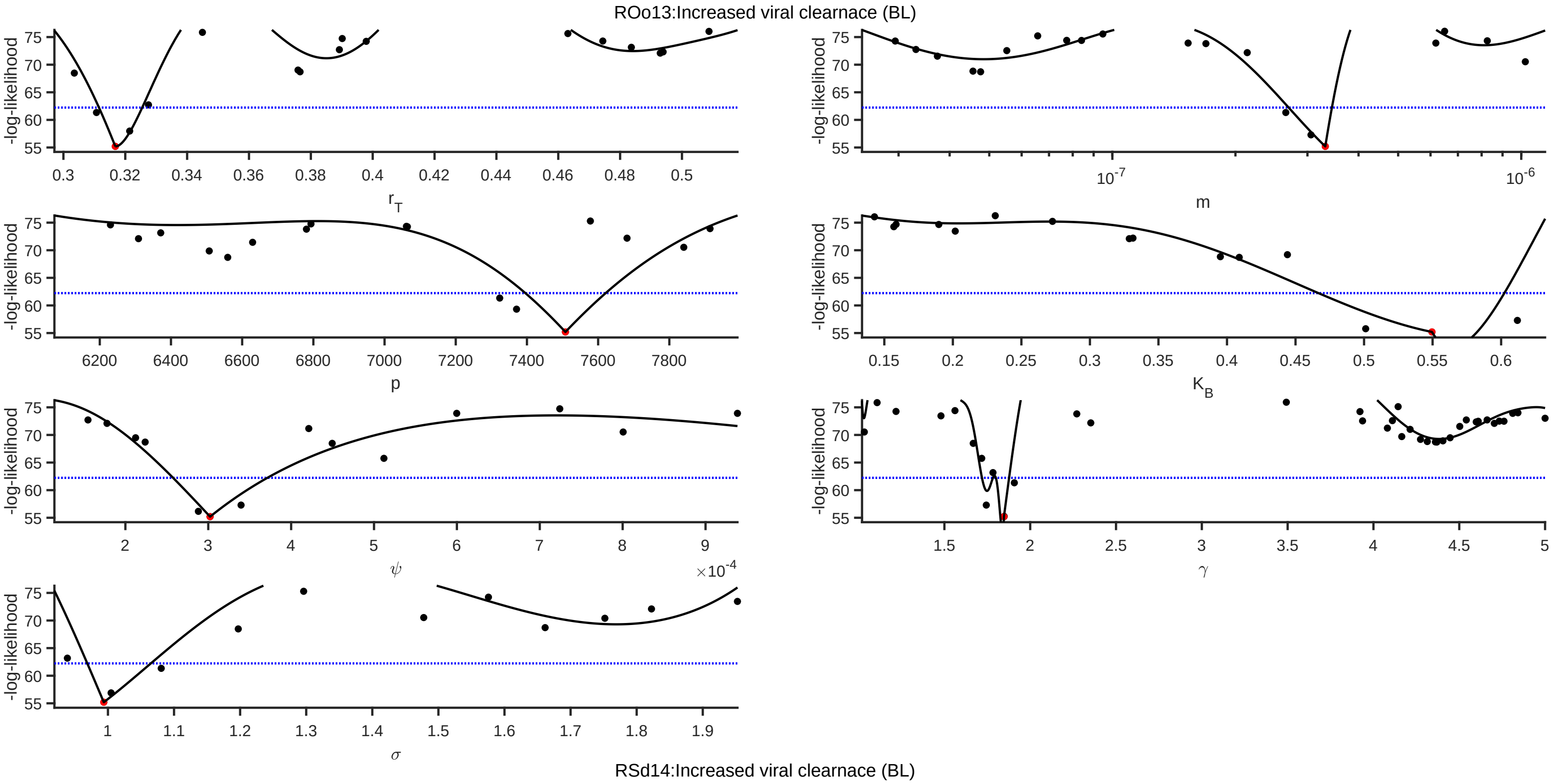
ROo13:Increased viral clearnace (BL)
-log-likelihood
r_T
m
p
K_B
ψ
γ
σ
$\times 10^{-4}$

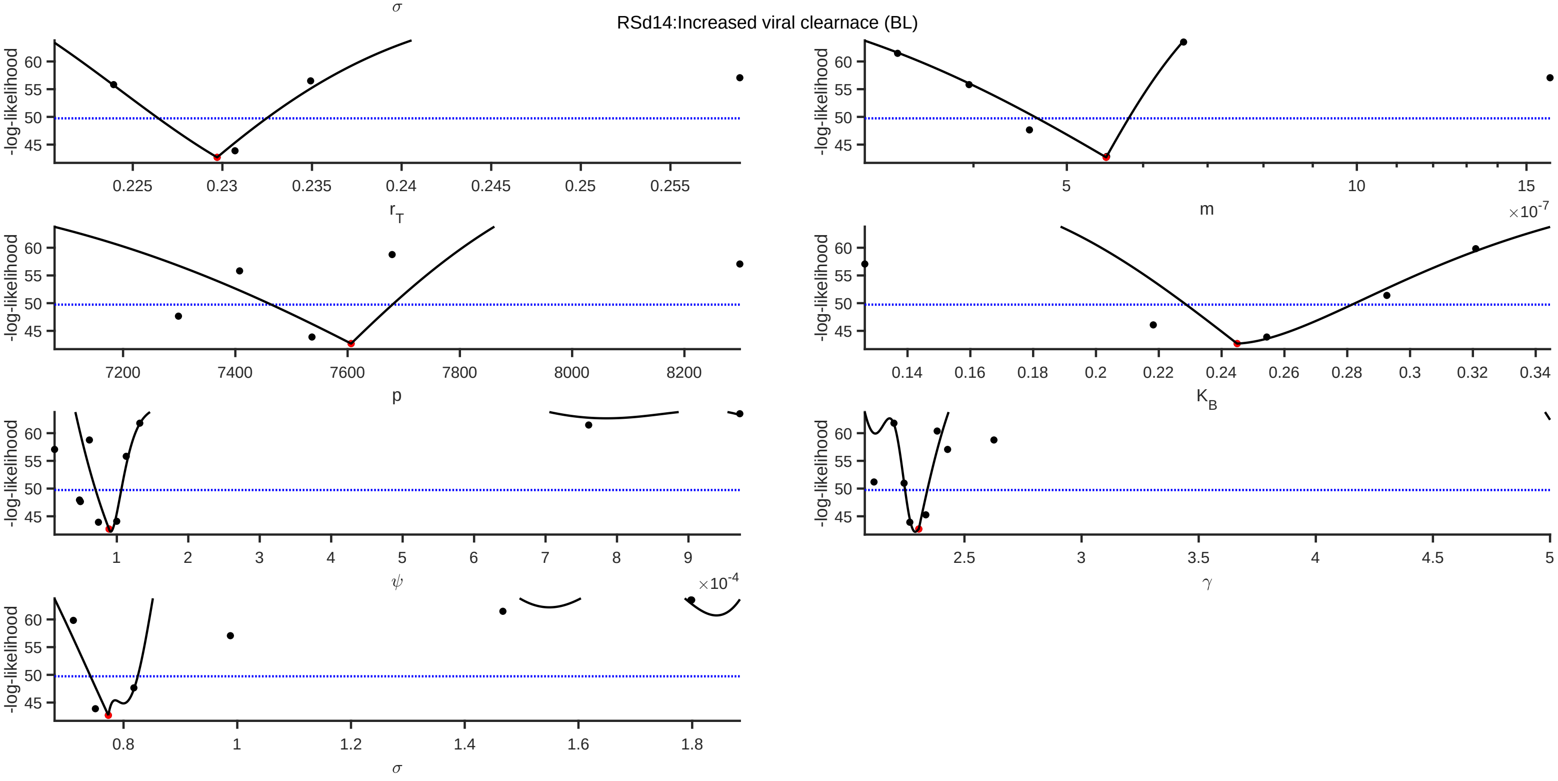
RSd14:Increased viral clearnace (BL)
-log-likelihood
r_T
m
$\times 10^{-7}$
p
K_B
ψ
γ
σ
$\times 10^{-4}$

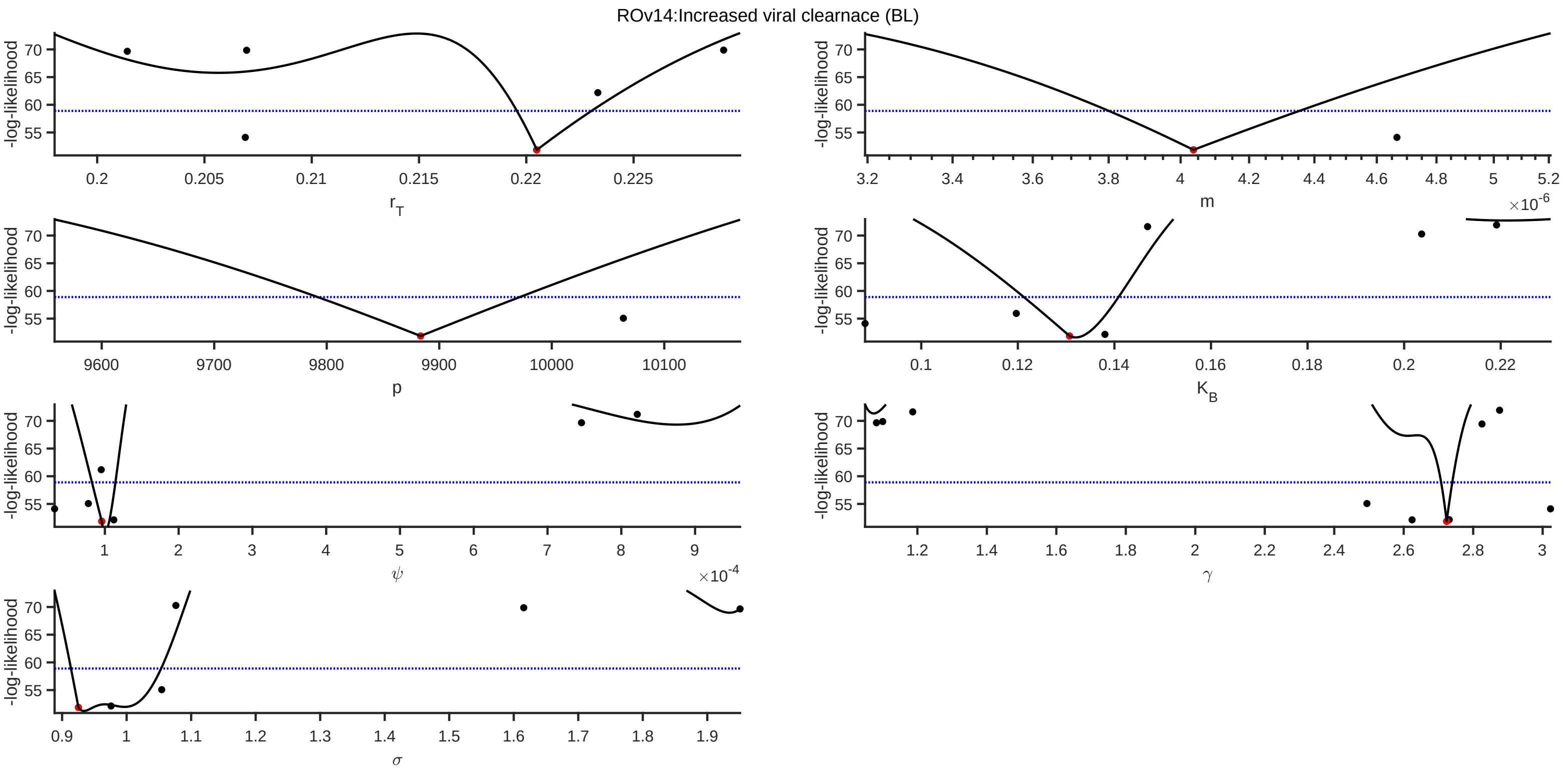

ROv14:Increased viral clearnace (BL)
-log-likelihood
r_T
m
$\times 10^{-6}$
p
K_B
ψ
$\times 10^{-4}$
γ
σ

3.2 Saturated effector cell source model

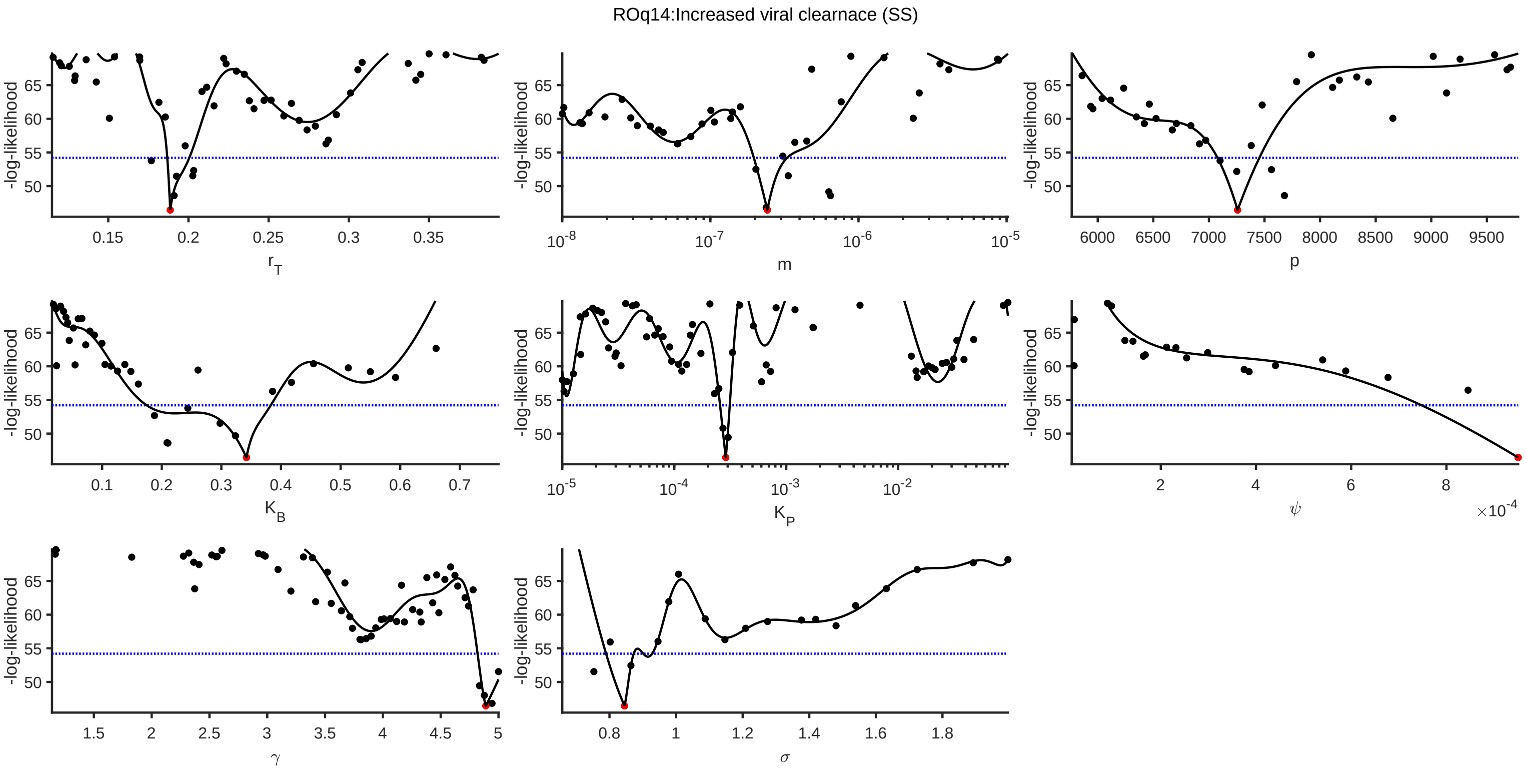

RDa15:Increased viral clearnace (SS)

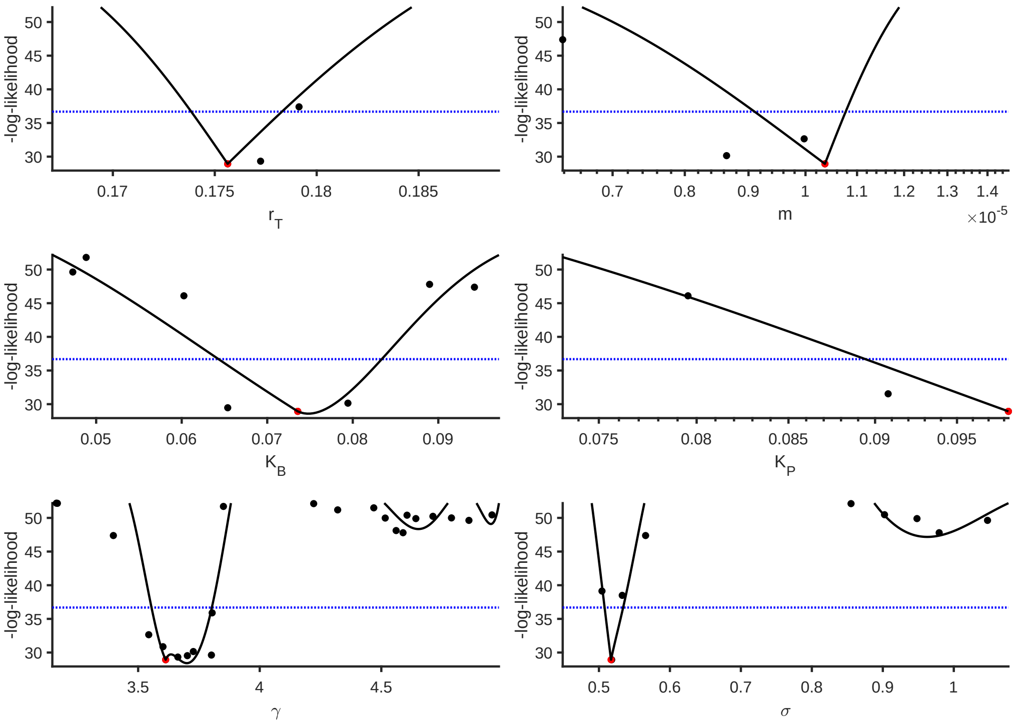

RFa15:Increased viral clearnace (SS)

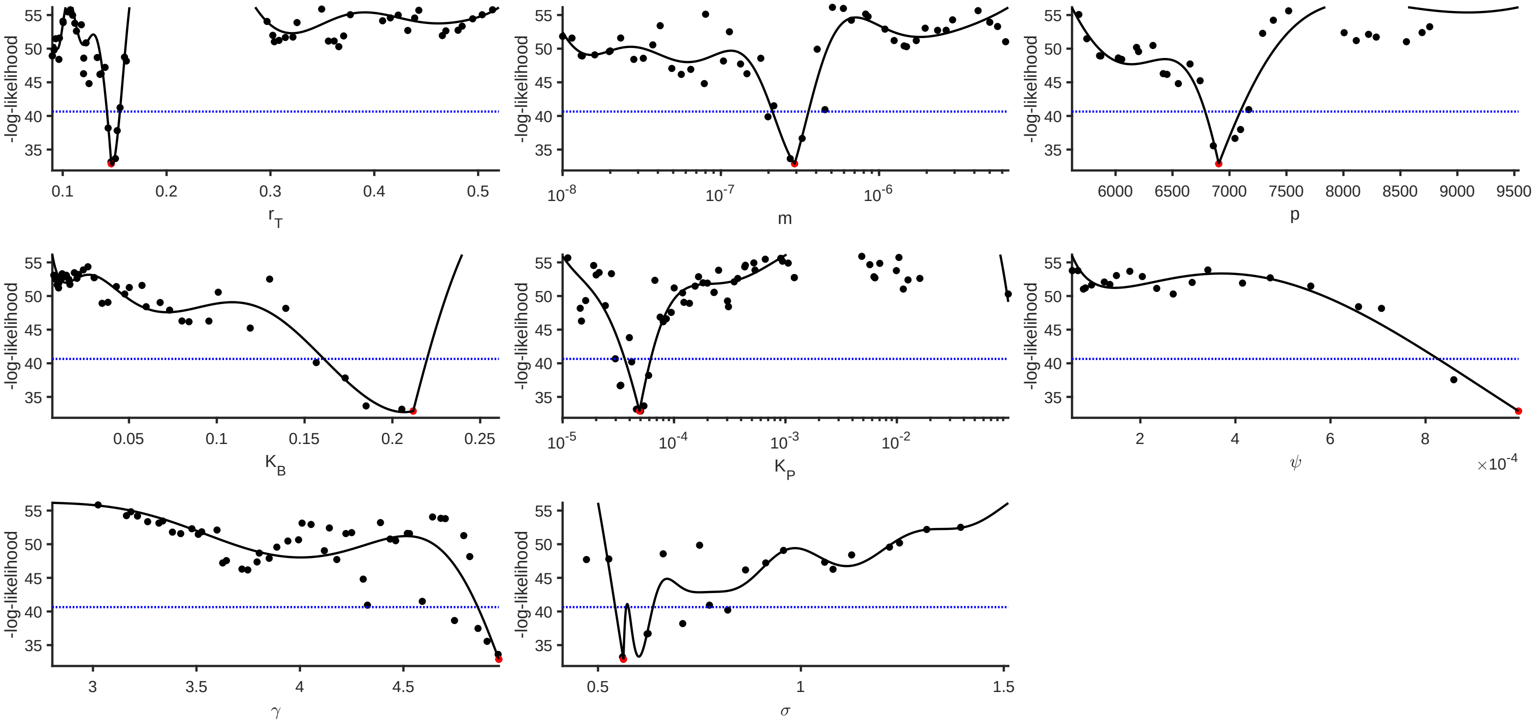

RLn12:Increased viral clearnace (SS)

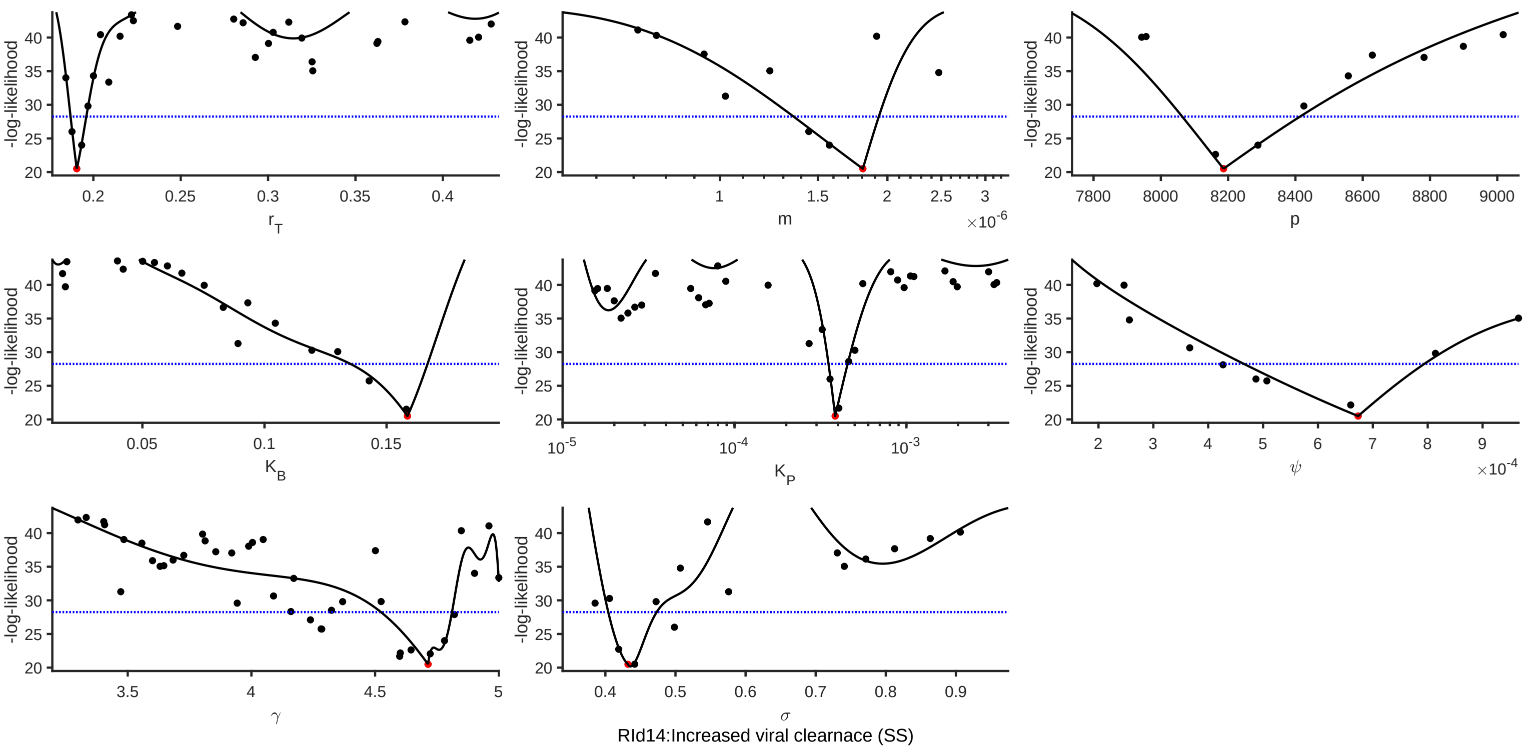

RId14:Increased viral clearnace (SS)

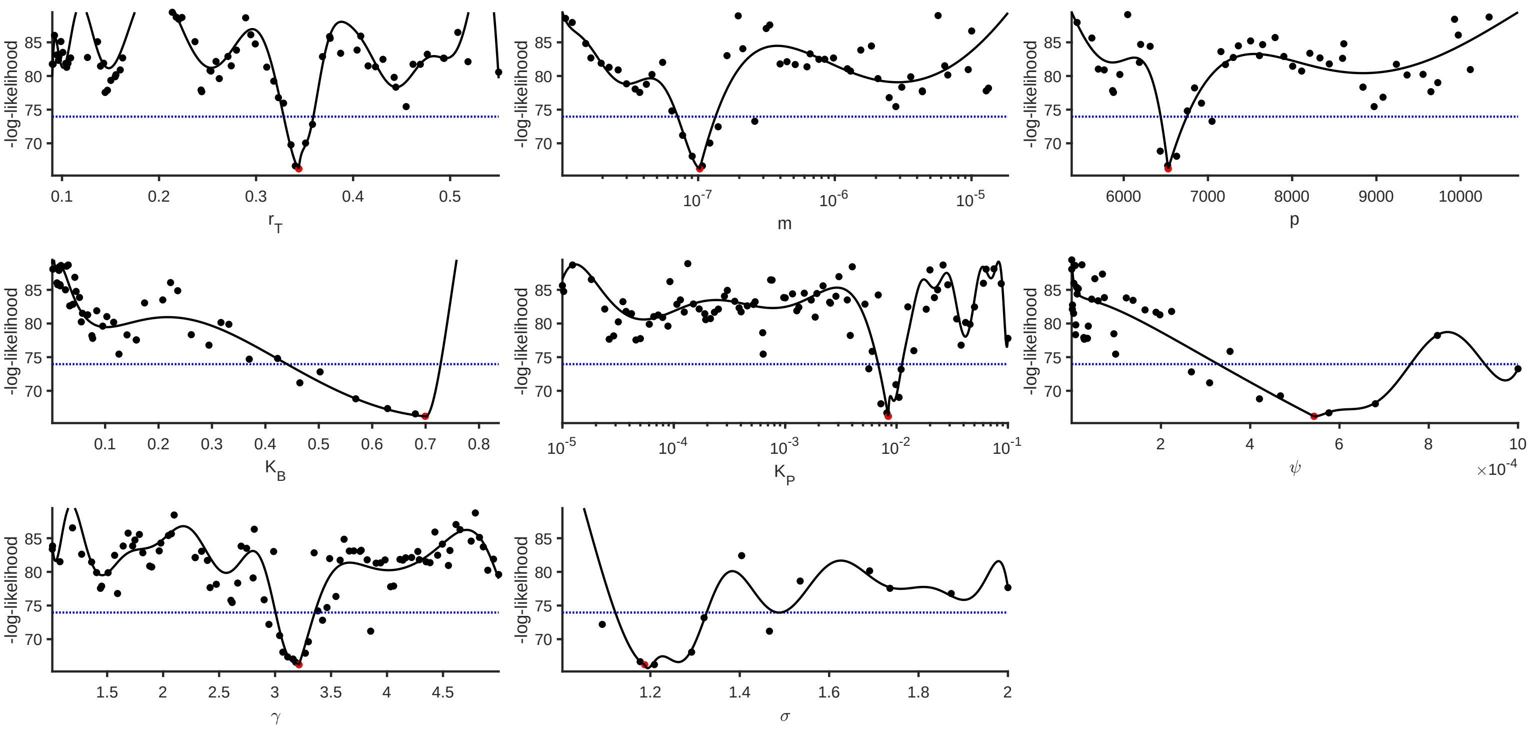

ROo13:Increased viral clearnace (SS)

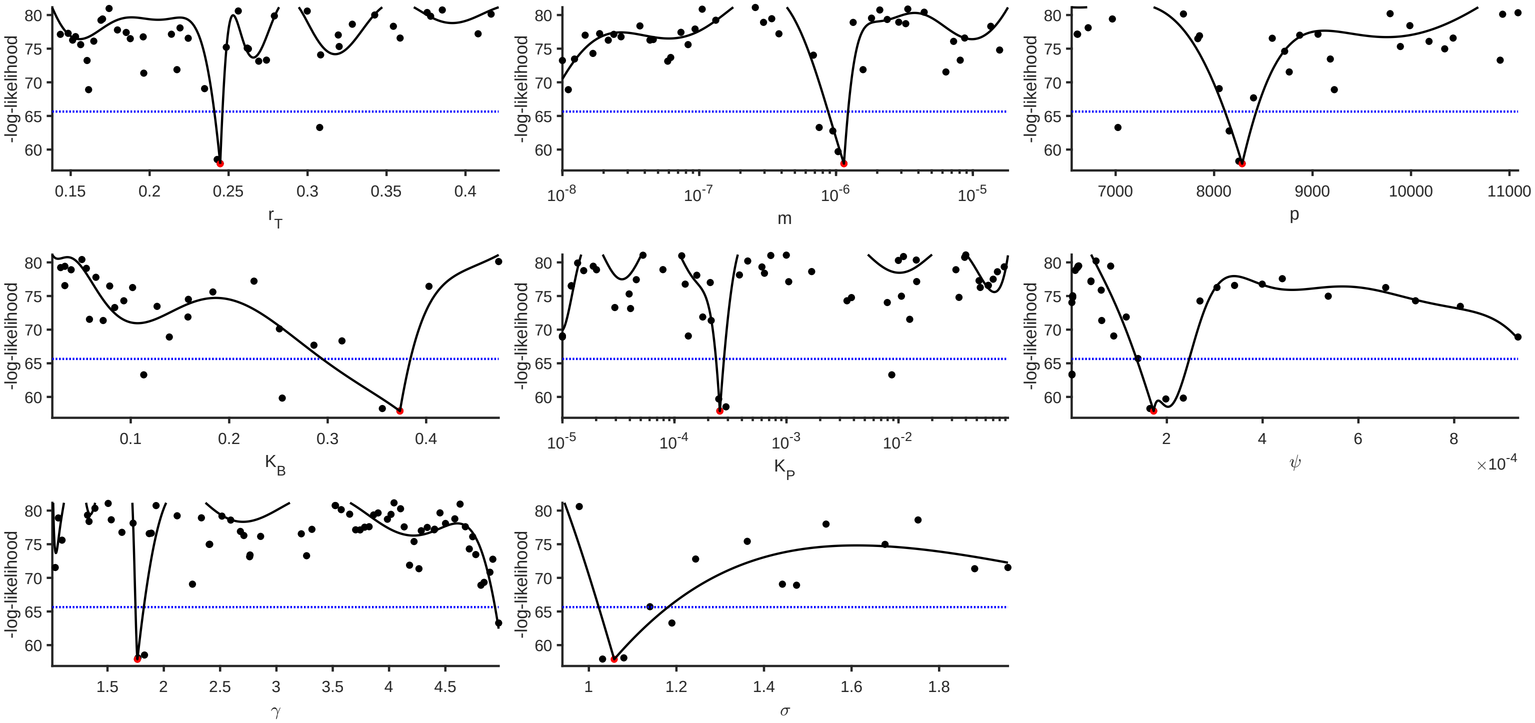

RSd14:Increased viral clearnace (SS)

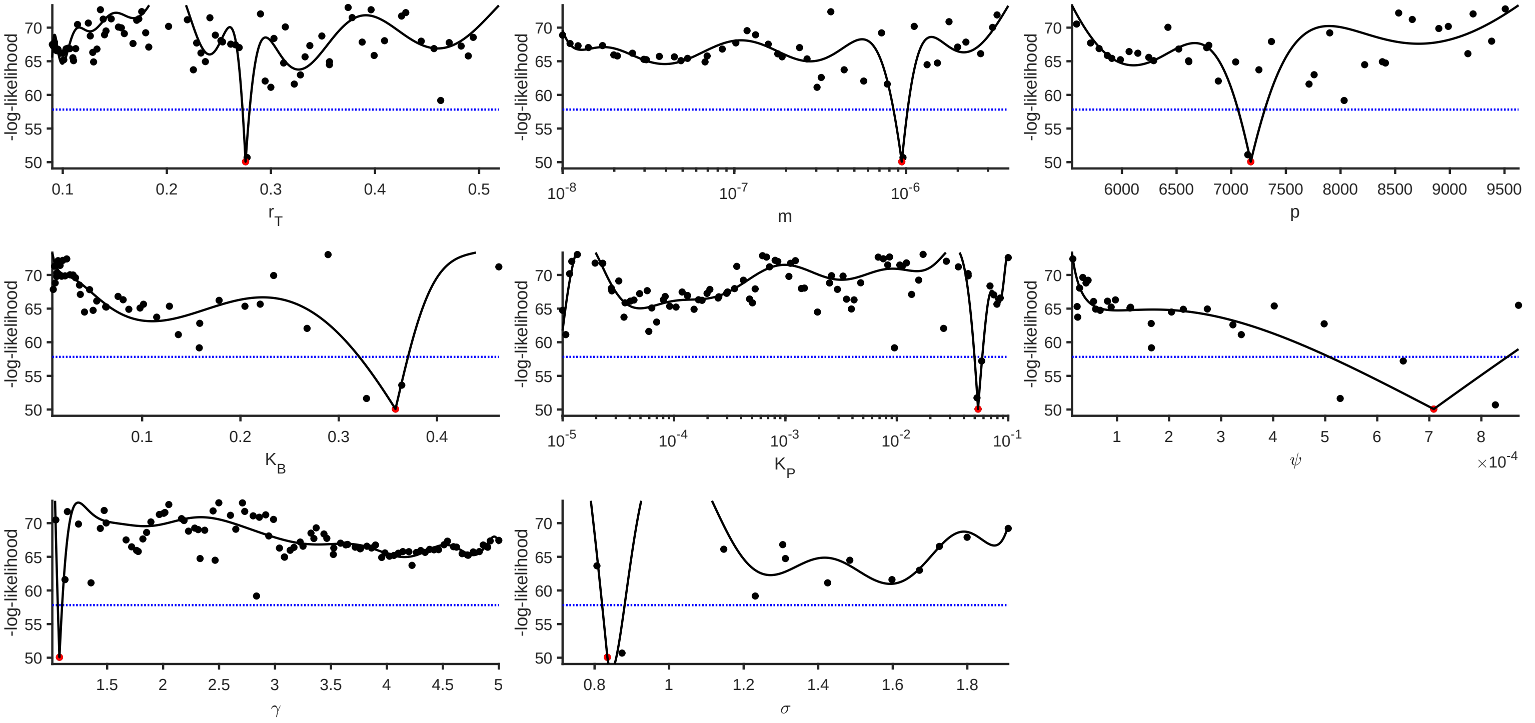

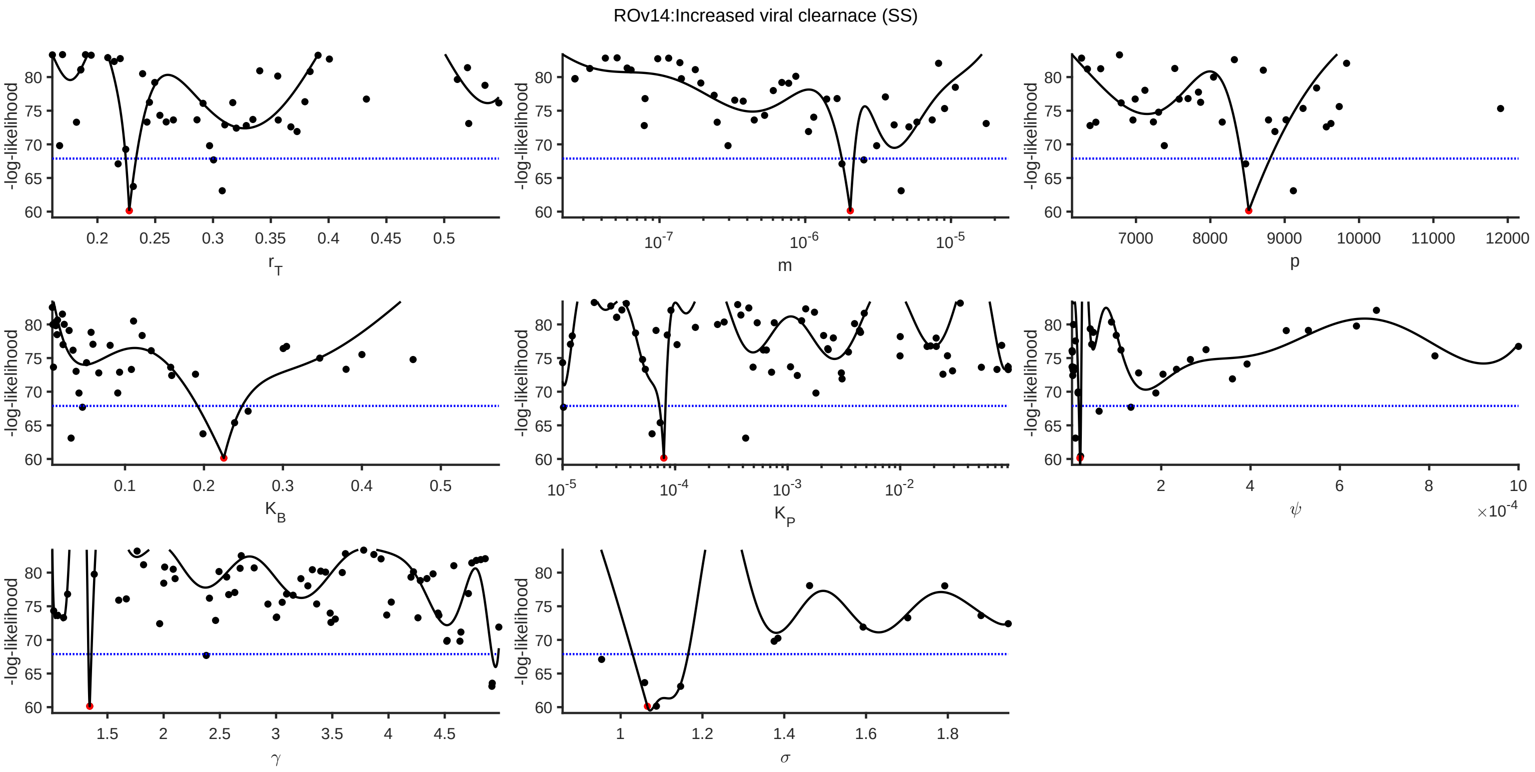
ROv14:Increased viral clearnace (SS)
-log-likelihood
r_T
m
p
K_B
K_P
ψ
×10^-4
γ
σ

3.3 Antigen presenting cell effector cell source model

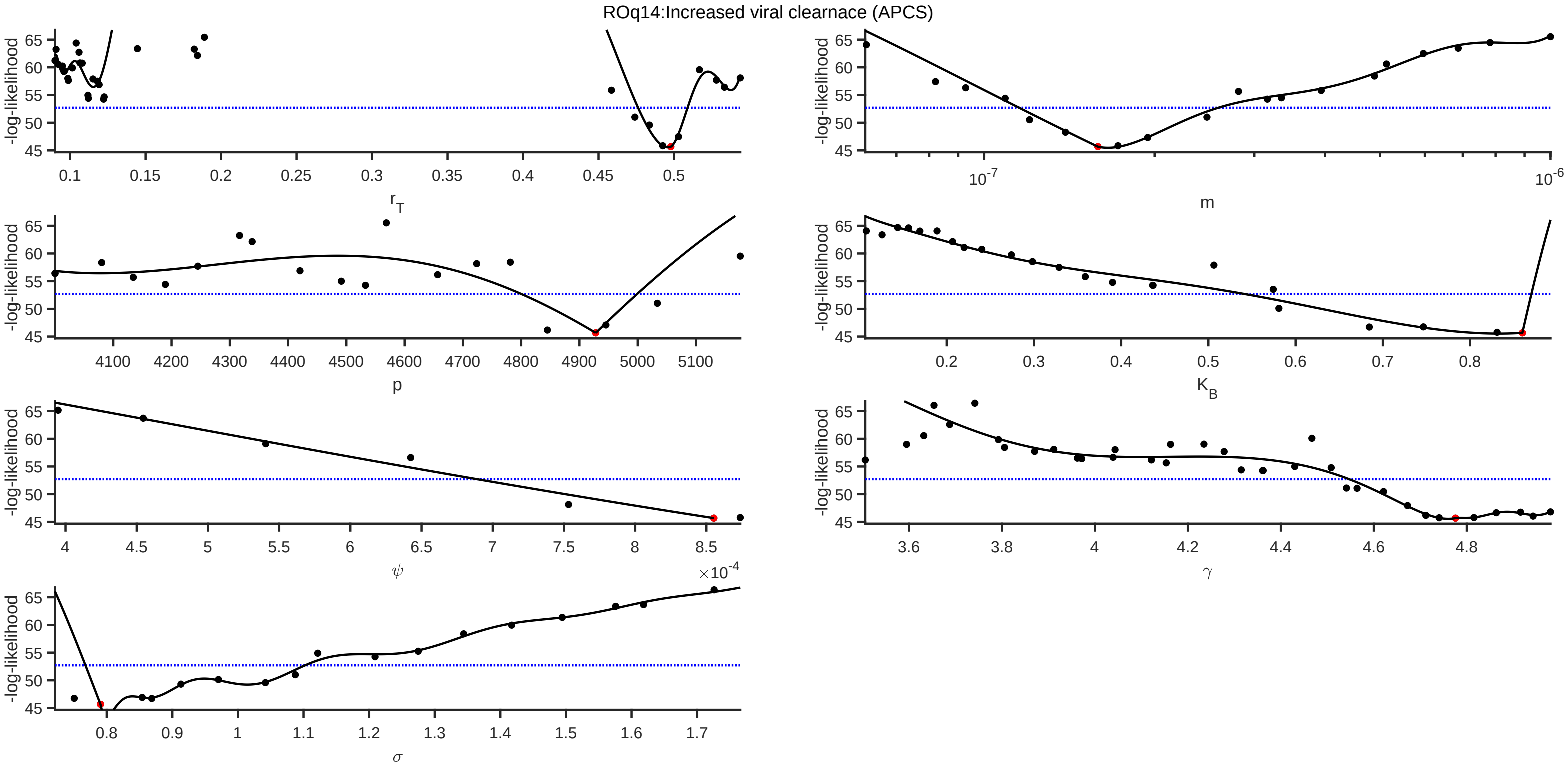

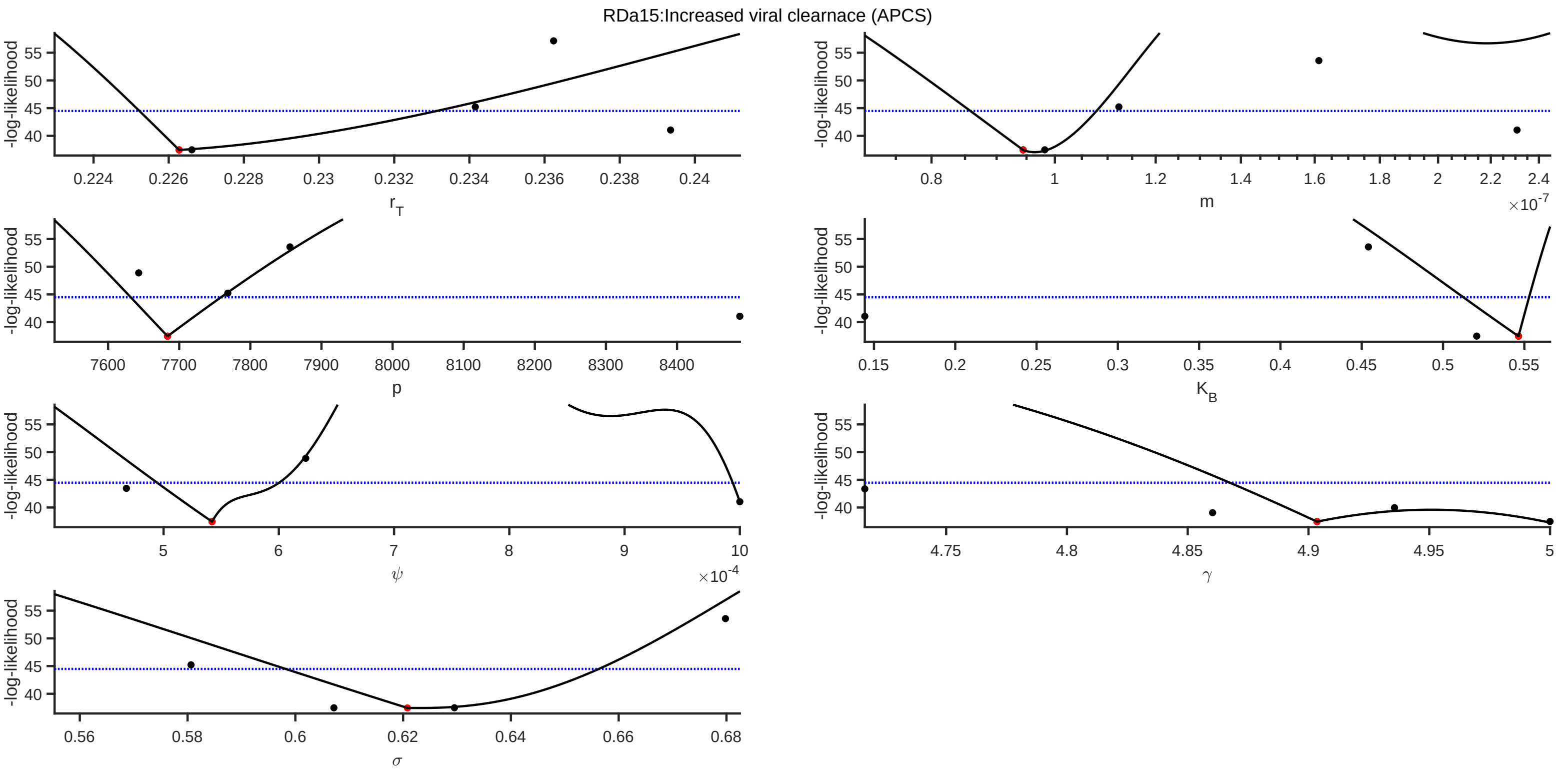
RDa15:Increased viral clearnace (APCS)
-log-likelihood
r_T
m
$\times10^{-7}$
p
K_B
ψ
$\times10^{-4}$
γ
σ

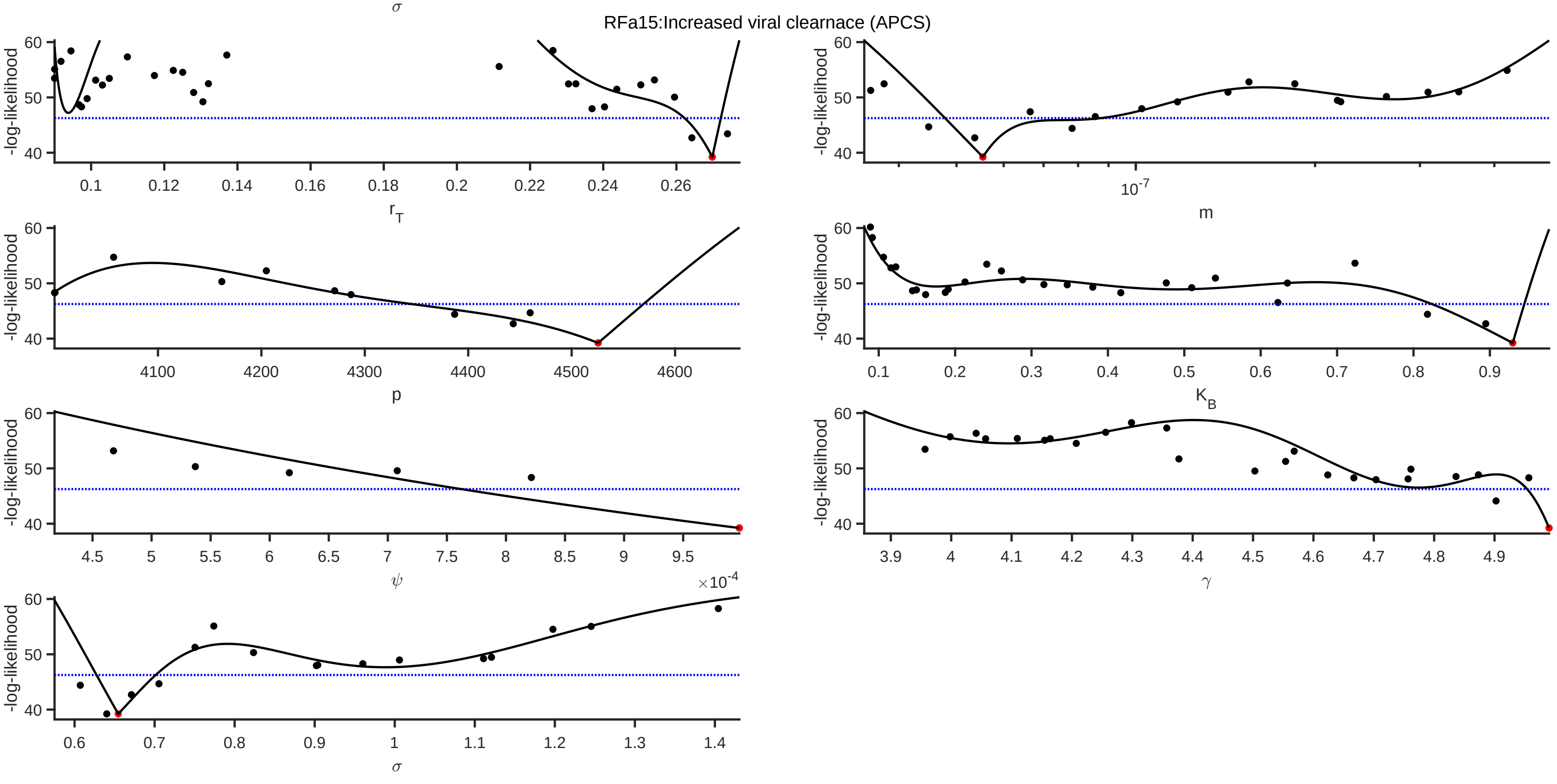
RFa15:Increased viral clearnace (APCS)
-log-likelihood
r_T
m
10^{-7}
p
K_B
ψ
$\times10^{-4}$
γ
σ

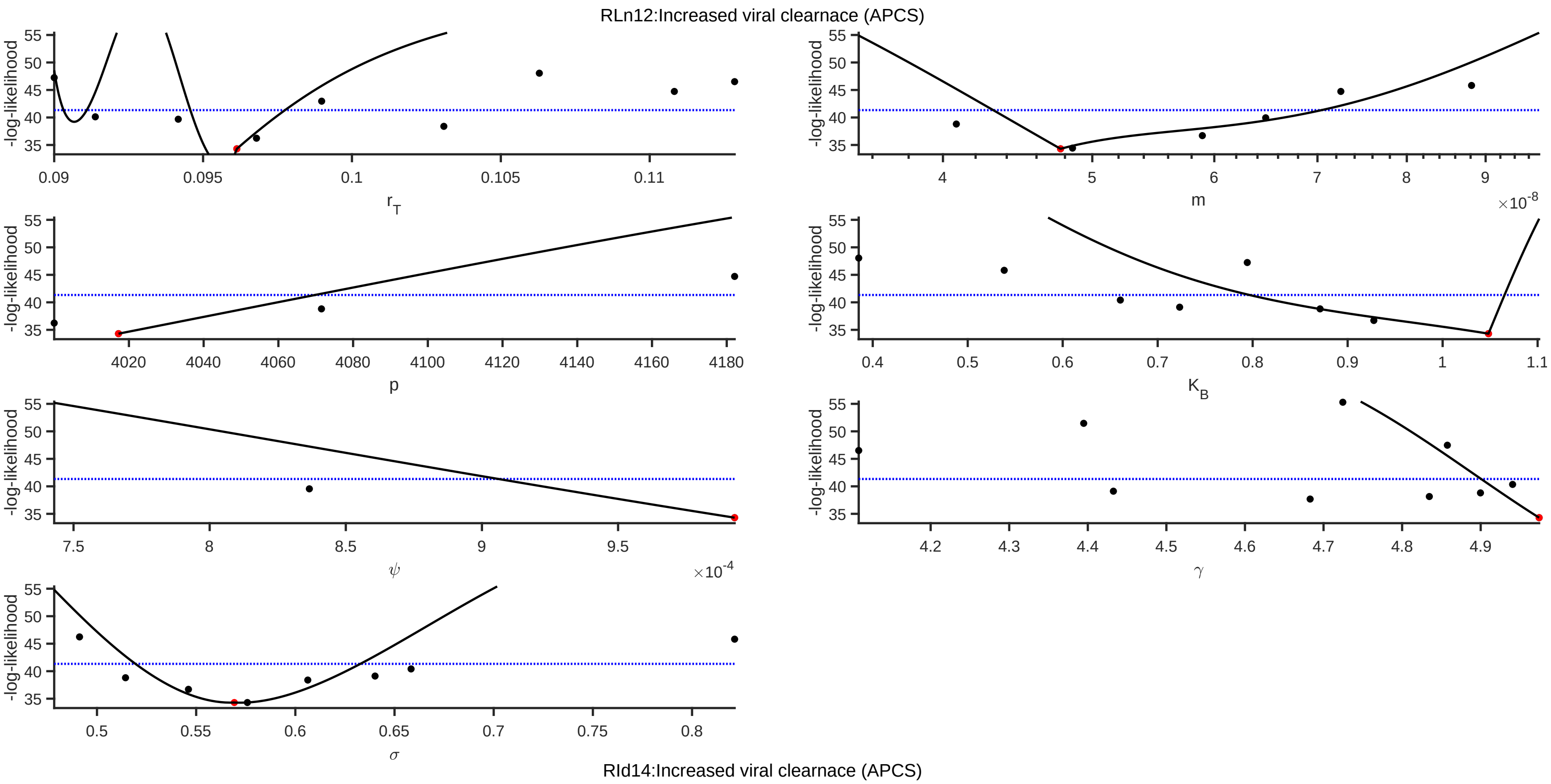
RLn12:Increased viral clearnace (APCS)
-log-likelihood
r_T
m
×10^-8
p
K_B
ψ
×10^-4
γ
σ

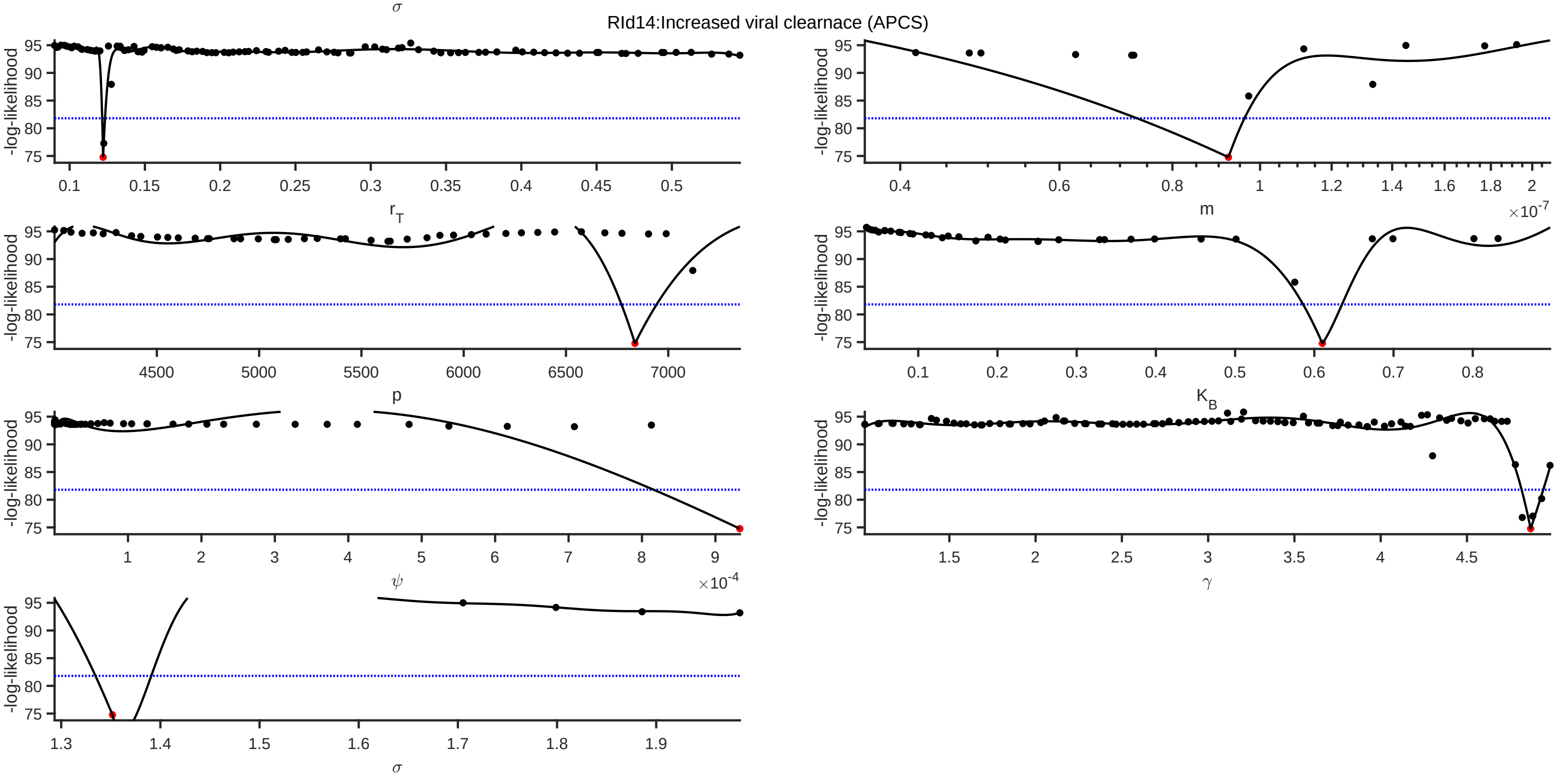
RId14:Increased viral clearnace (APCS)
-log-likelihood
r_T
m
×10^-7
p
K_B
ψ
×10^-4
γ
σ

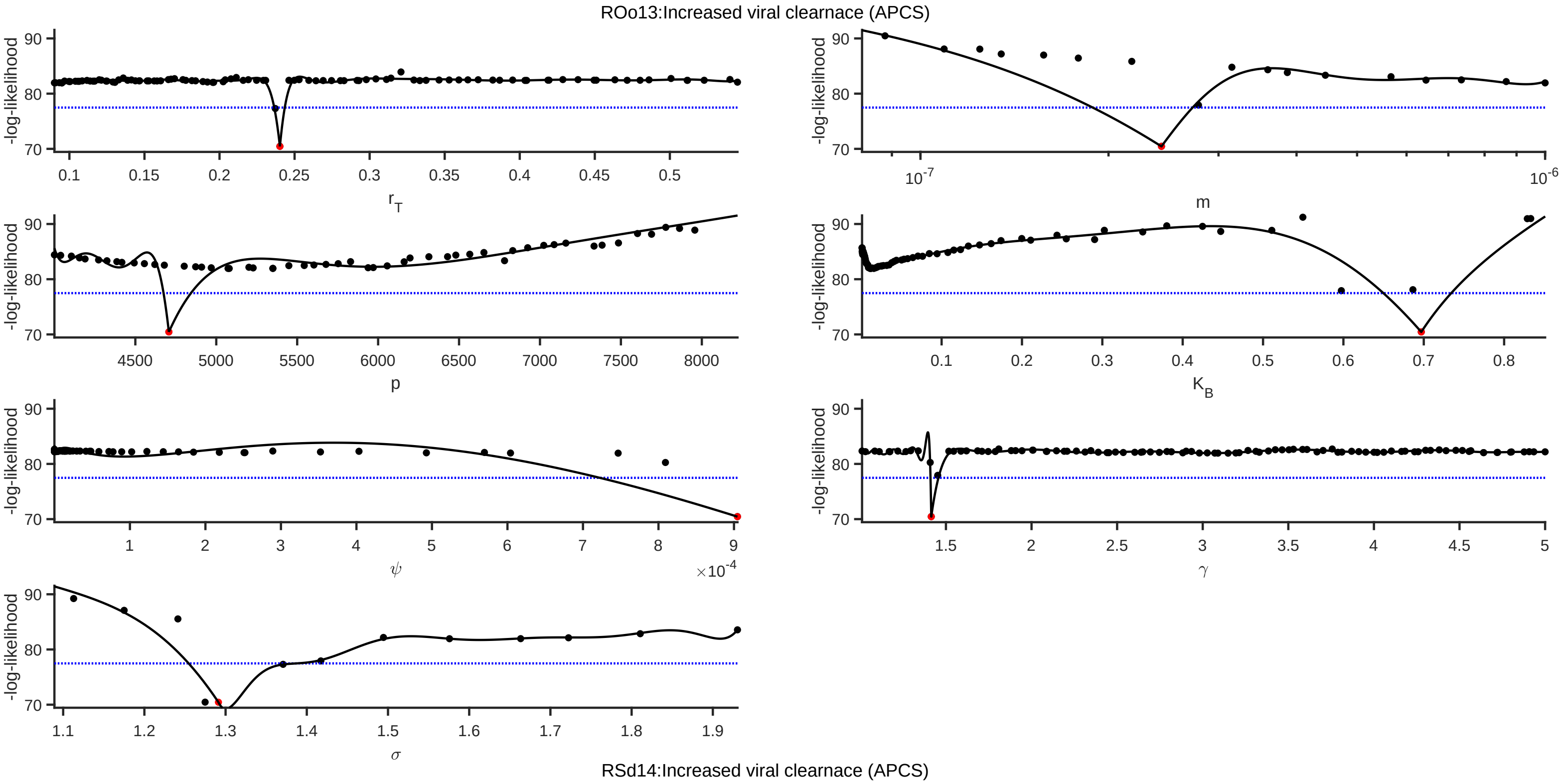
ROo13:Increased viral clearnace (APCS)
-log-likelihood
r_T
m
p
K_B
ψ
×10^-4
γ
σ

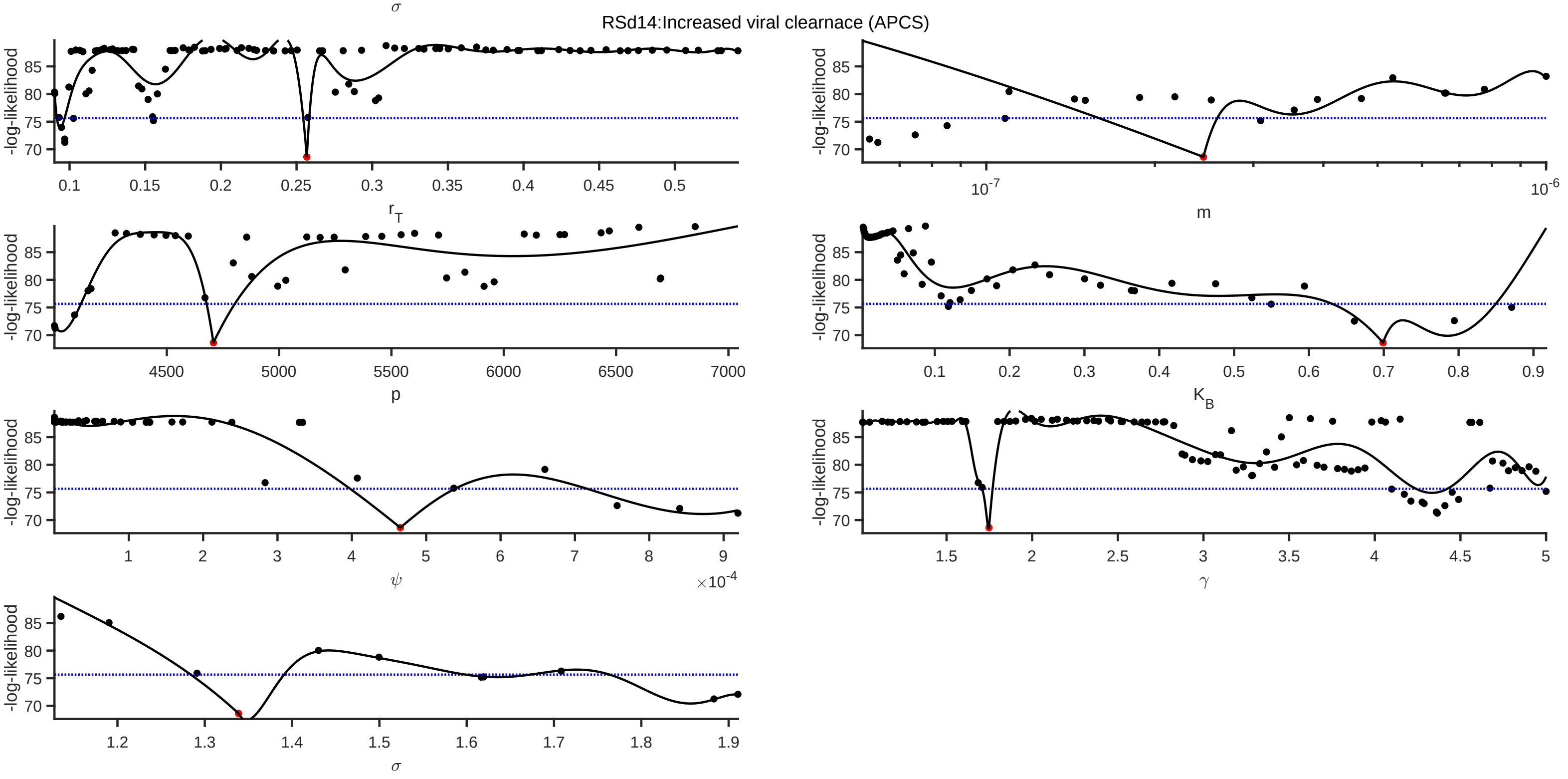
RSd14:Increased viral clearnace (APCS)
-log-likelihood
r_T
m
p
K_B
ψ
×10^-4
γ
σ

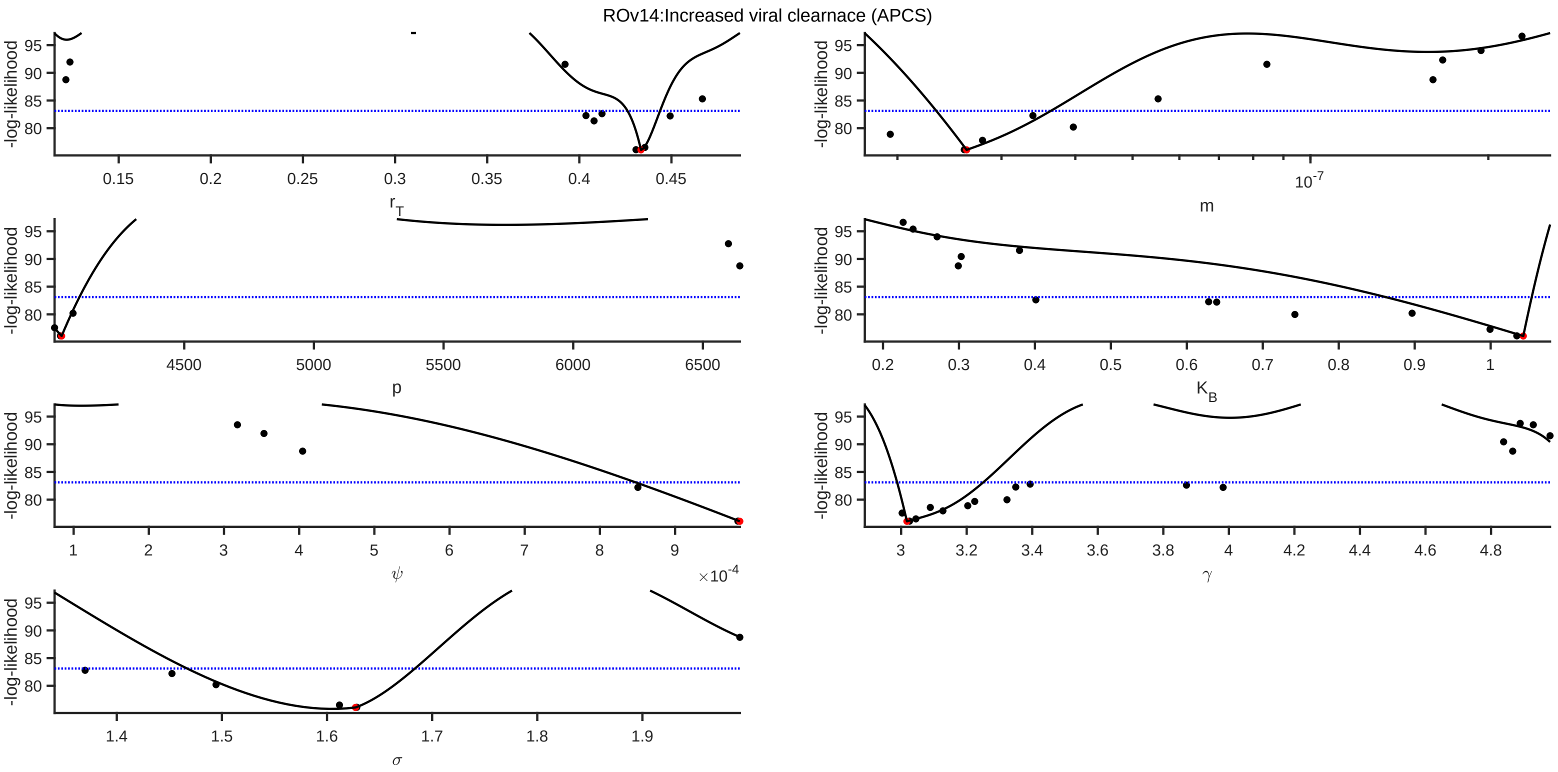

ROv14:Increased viral clearnace (APCS)
-log-likelihood
95
90
85
80
0.15
0.2
0.25
0.3
0.35
0.4
0.45
r_T
10^-7
m
4500
5000
5500
6000
6500
p
0.2
0.3
0.4
0.5
0.6
0.7
0.8
0.9
1
K_B
1
2
3
4
5
6
7
8
9
×10^-4
ψ
3
3.2
3.4
3.6
3.8
4
4.2
4.4
4.6
4.8
γ
1.4
1.5
1.6
1.7
1.8
1.9
σ

4 Sensitivity anlaysis: Increased viral clearance

4.1 Baseline effector cell source model

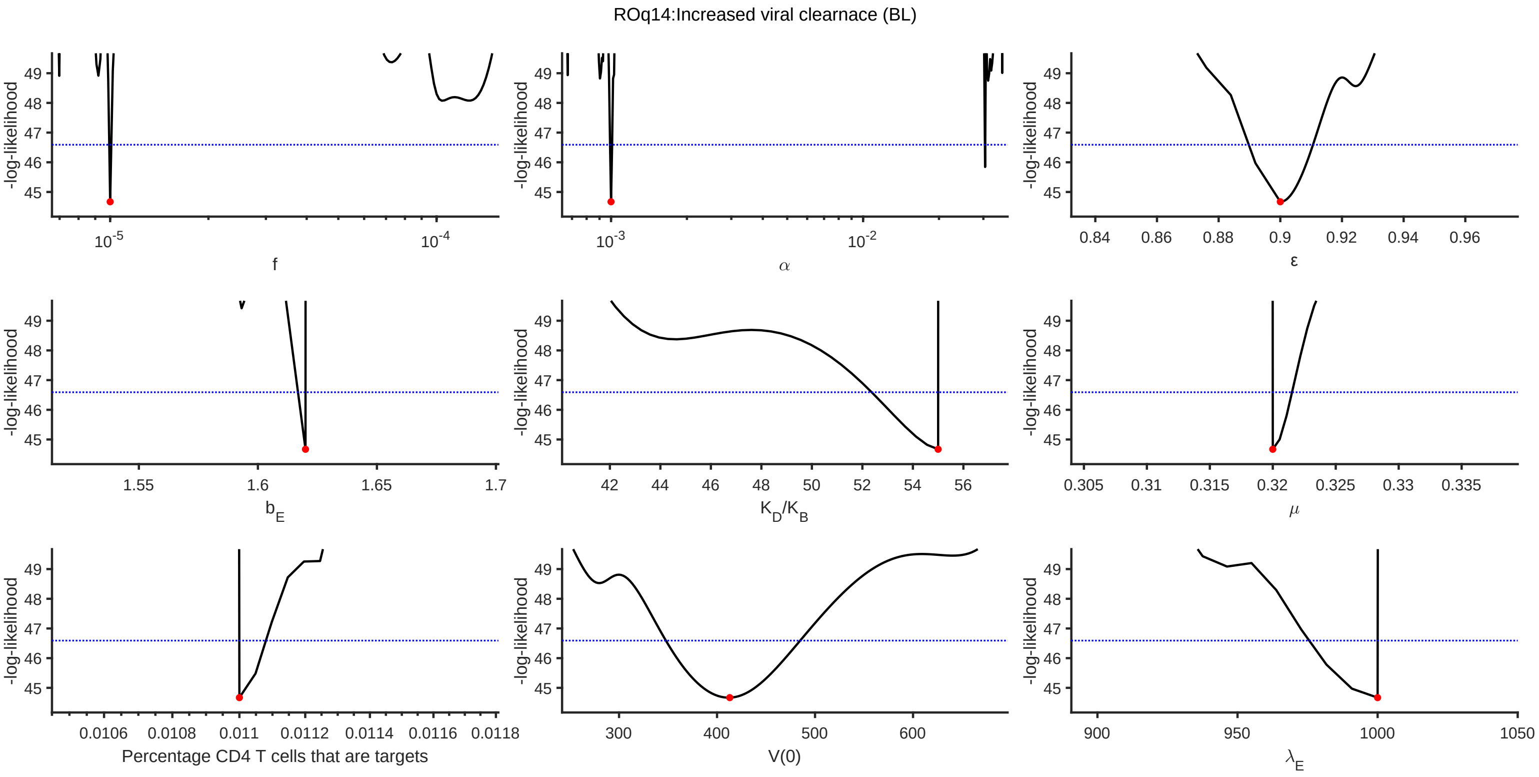

RDa15:Increased viral clearnace (BL)

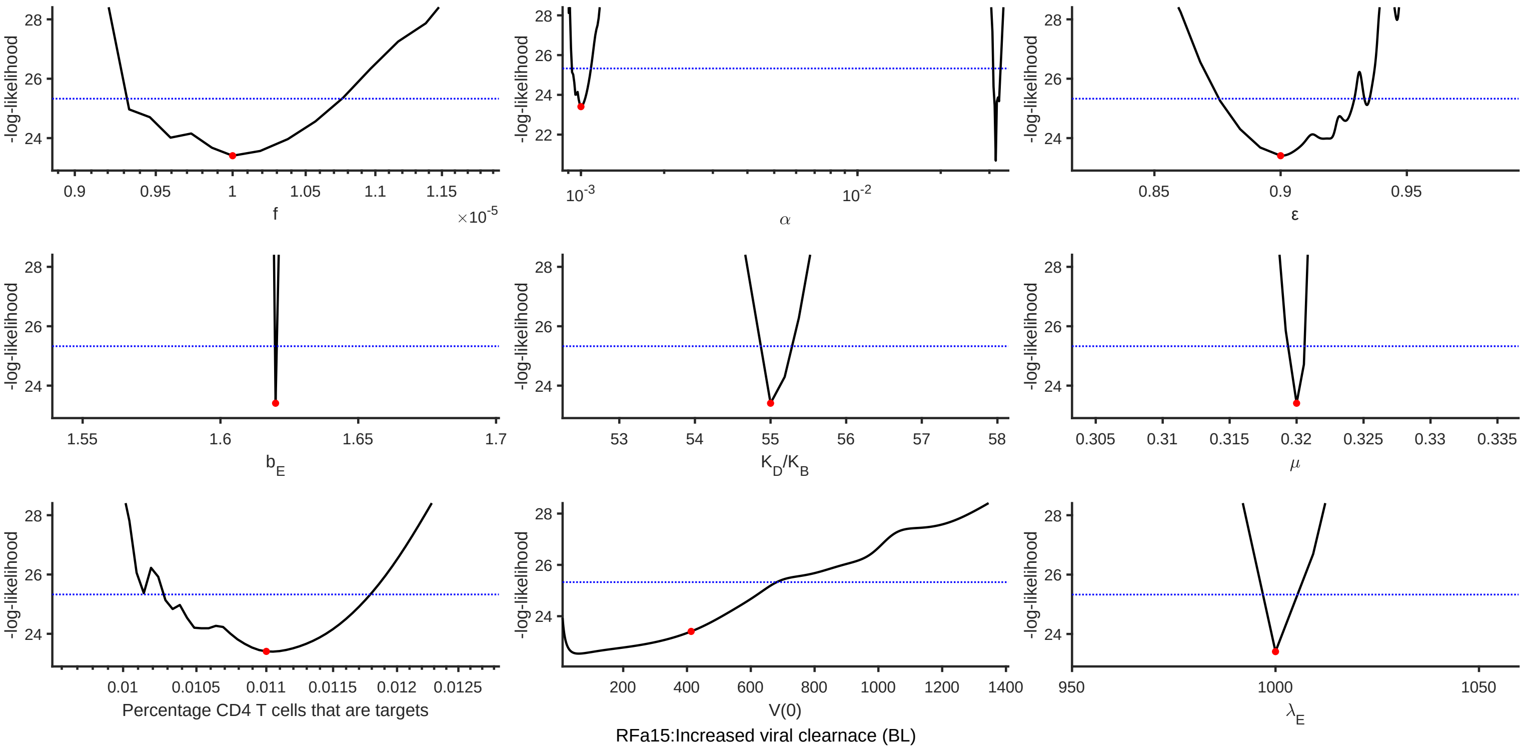

RFa15:Increased viral clearnace (BL)

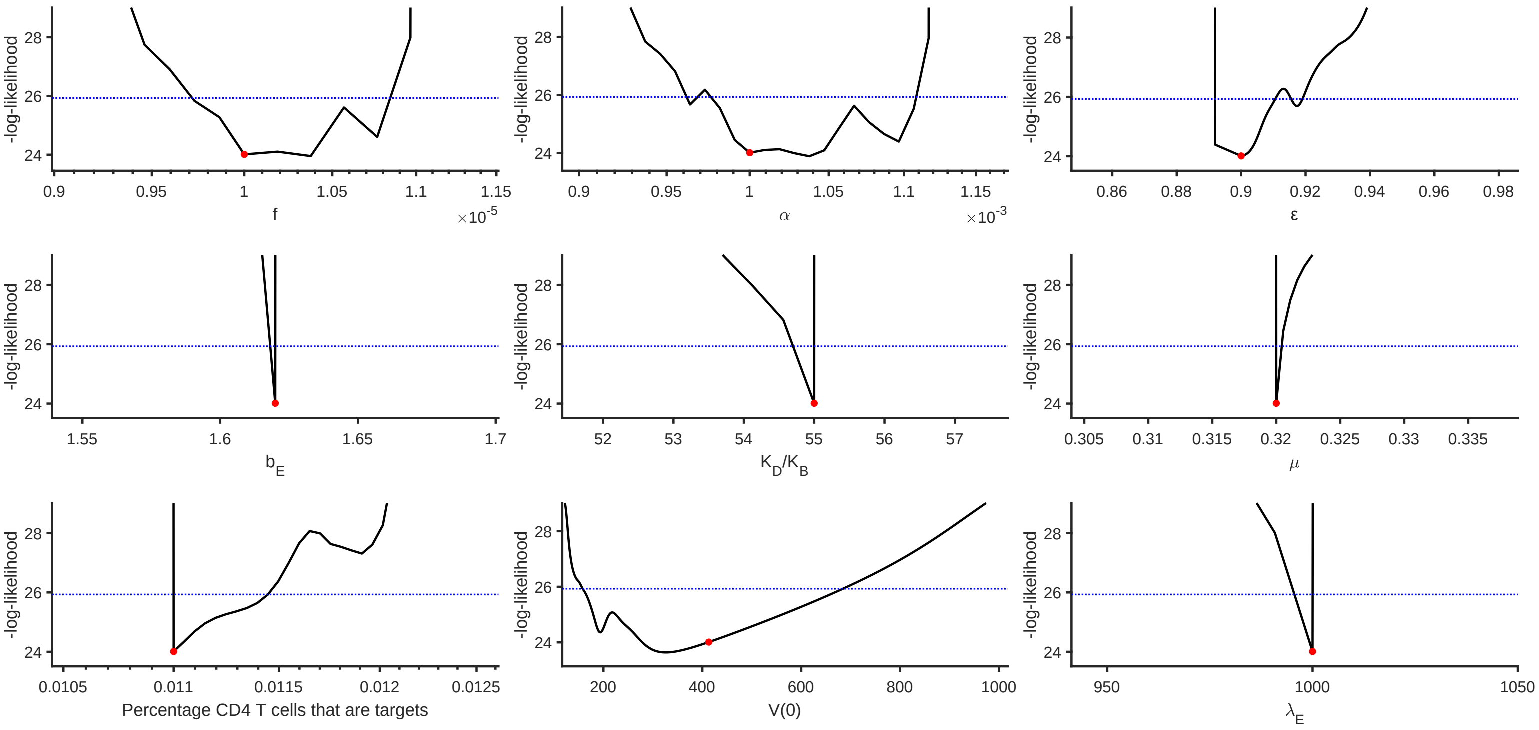

RLn12:Increased viral clearnace (BL)

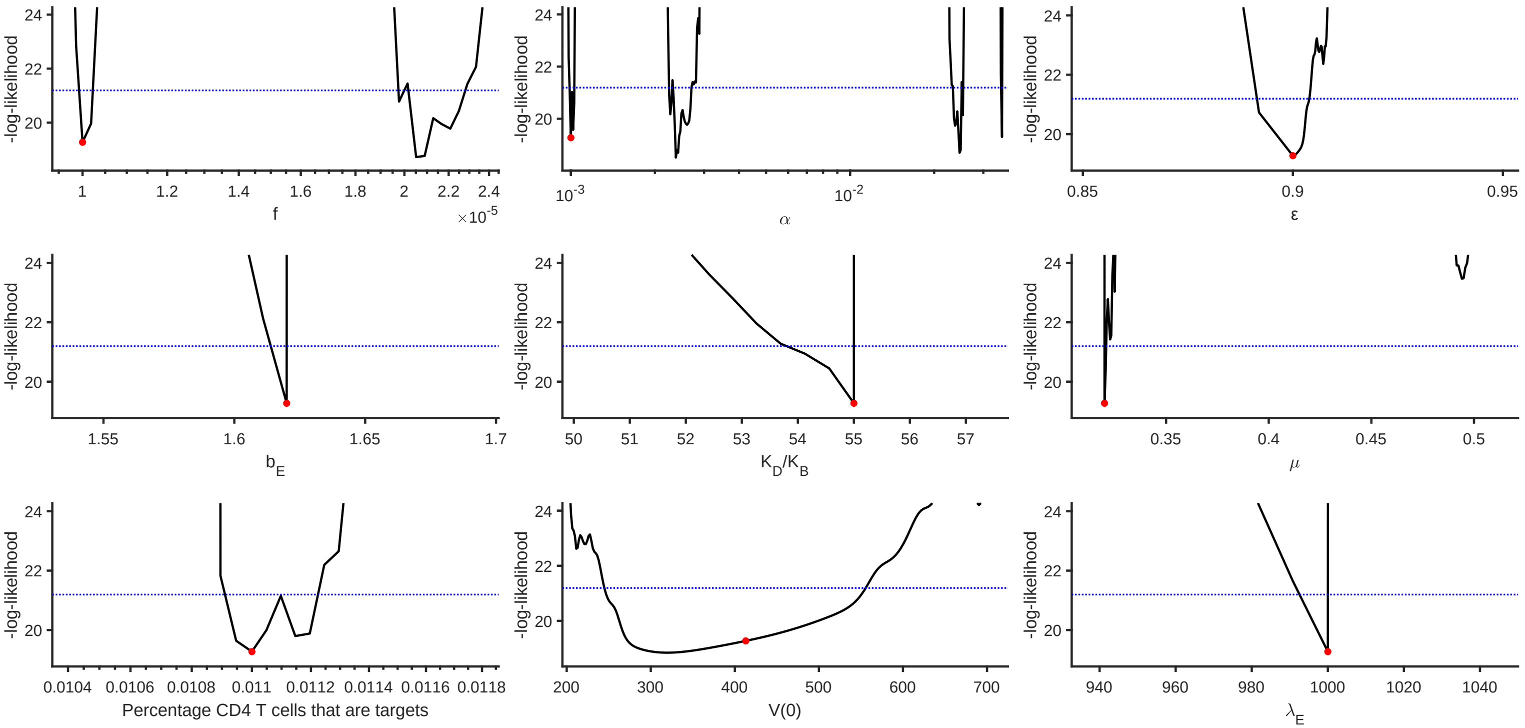

RId14:Increased viral clearnace (BL)

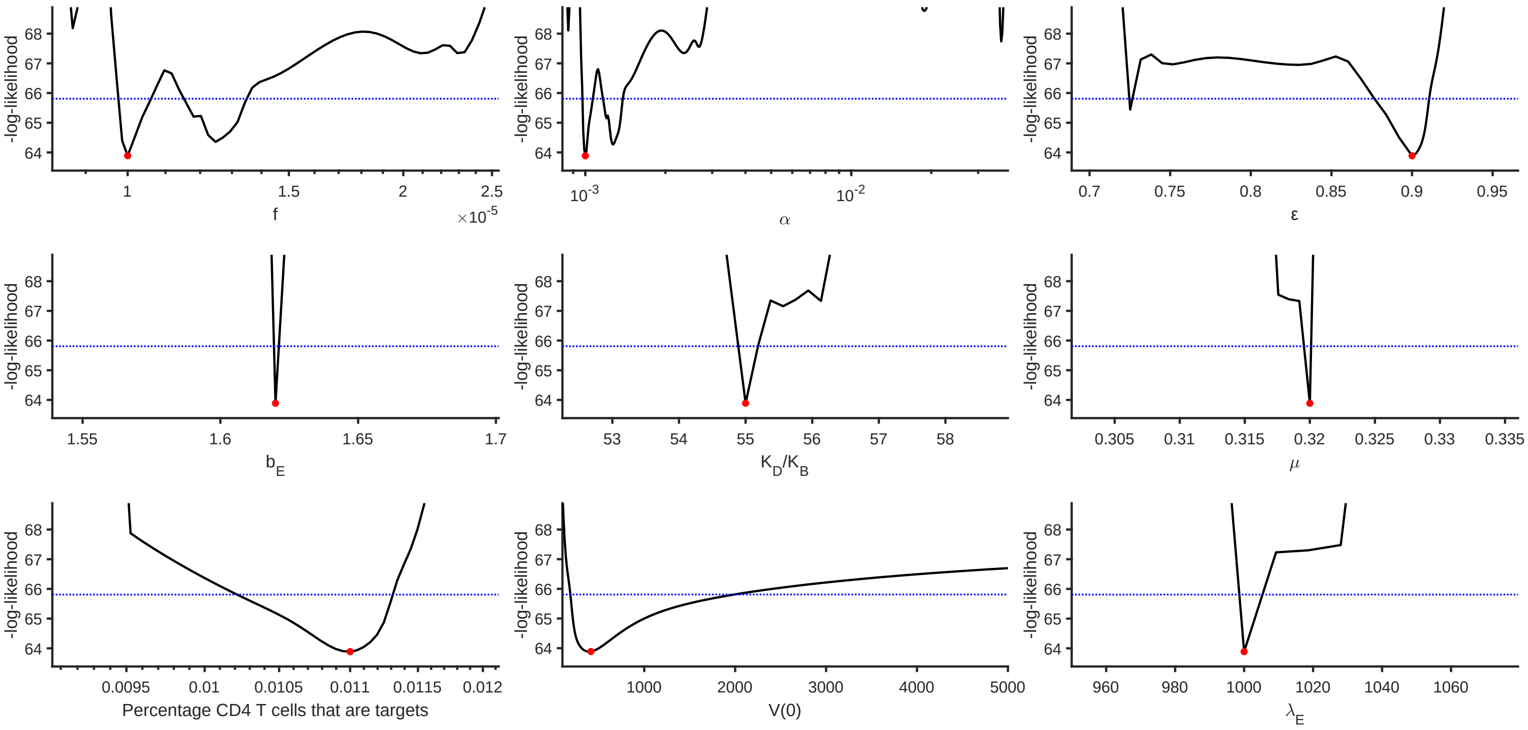

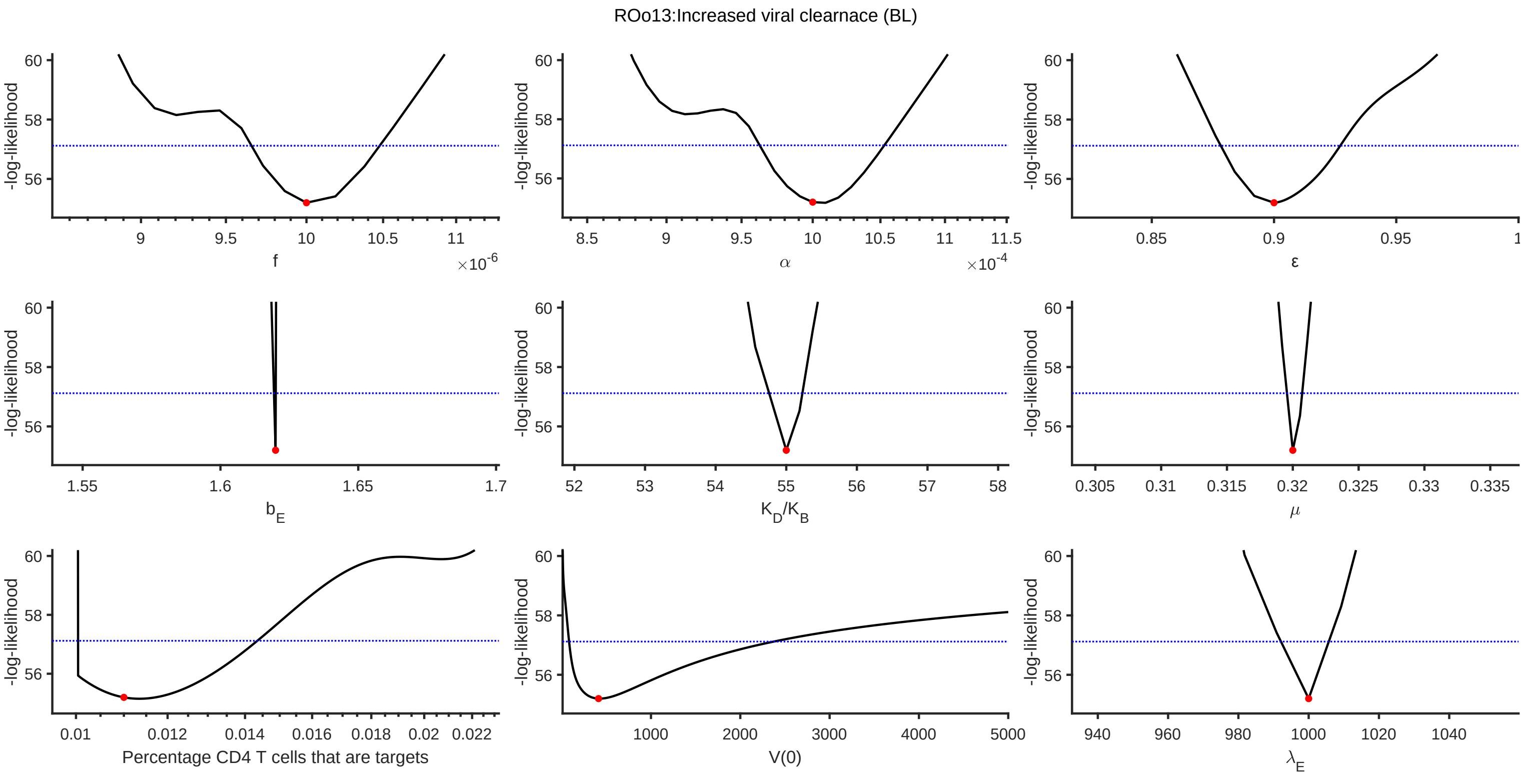
ROo13:Increased viral clearnace (BL)
-log-likelihood
f
×10^-6
α
×10^-4
ε
b_E
K_D/K_B
μ
Percentage CD4 T cells that are targets
V(0)
λ_E

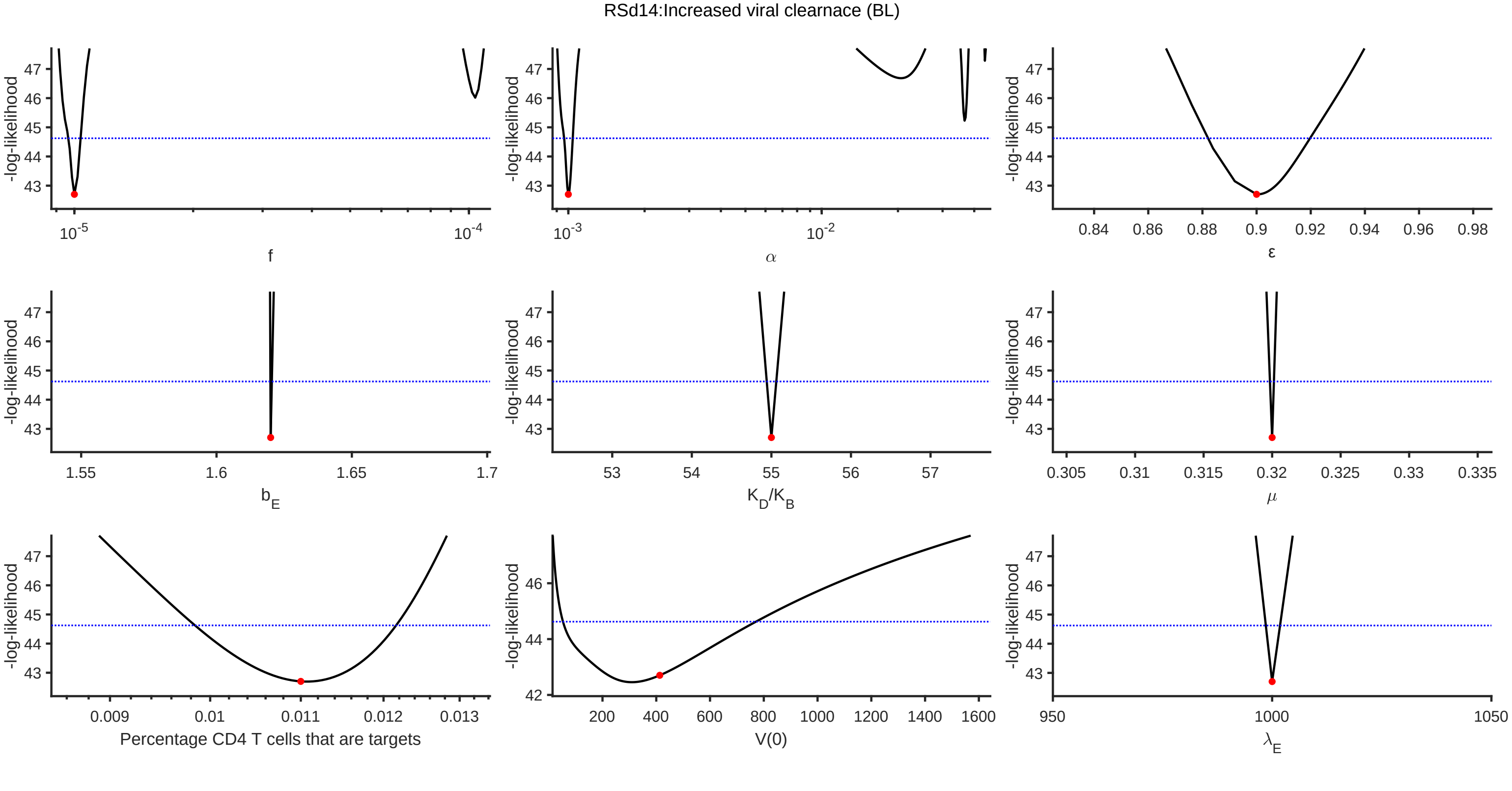
RSd14:Increased viral clearnace (BL)
-log-likelihood
f
α
ε
b_E
K_D/K_B
μ
Percentage CD4 T cells that are targets
V(0)
λ_E

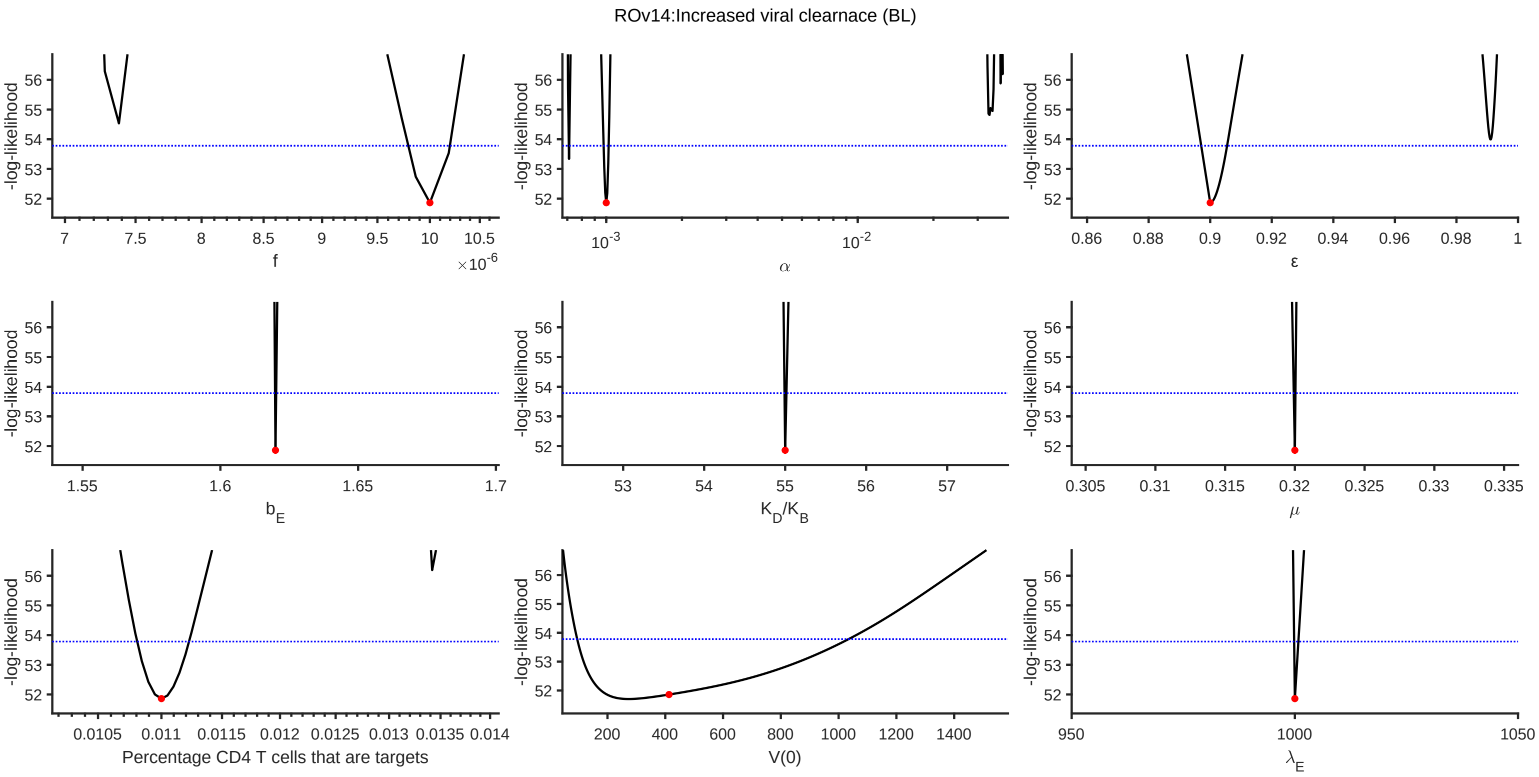
ROv14:Increased viral clearnace (BL)
-log-likelihood
f
×10^-6
α
ε
b_E
K_D/K_B
μ
Percentage CD4 T cells that are targets
V(0)
λ_E

4.2 Saturated effector cell source model

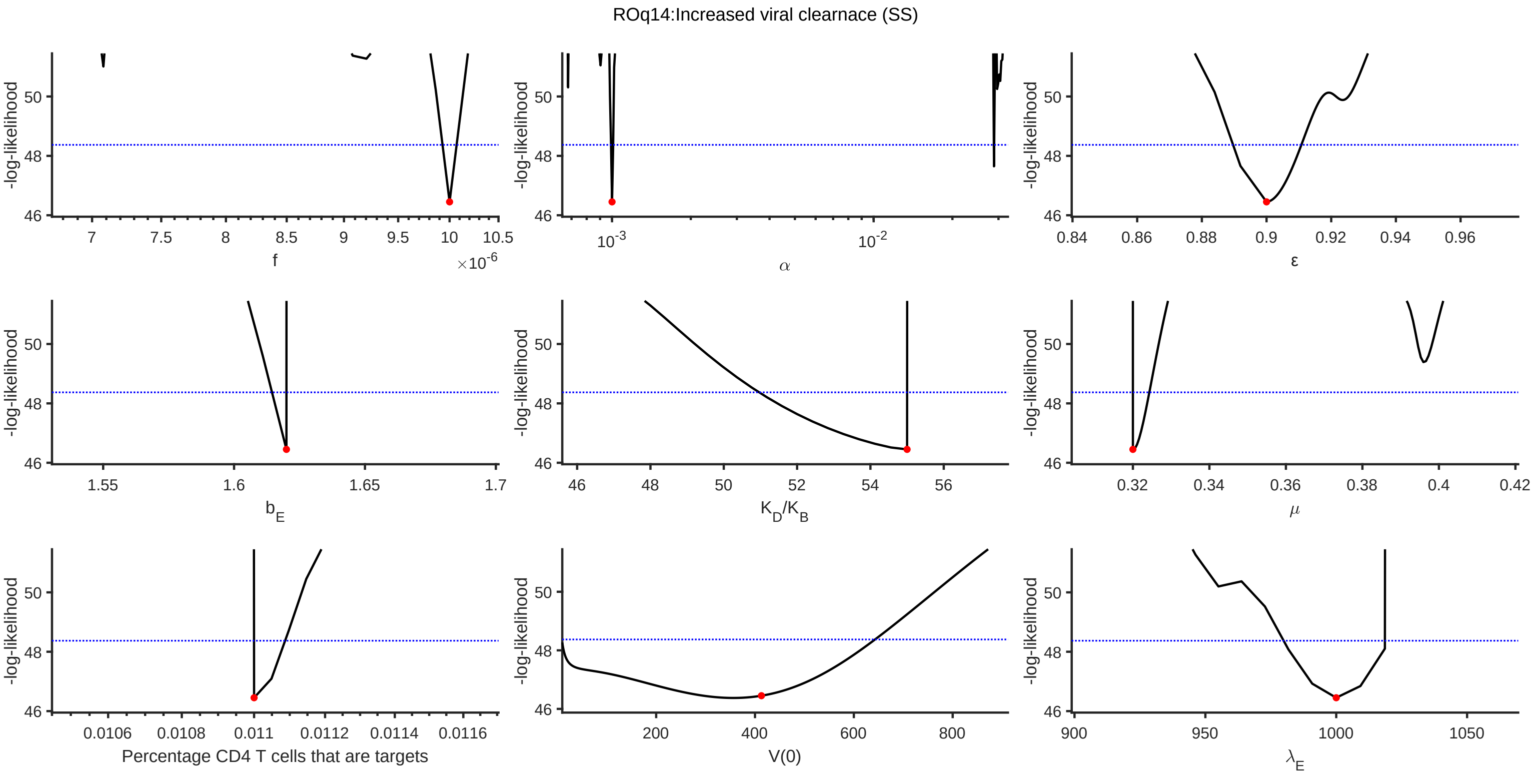

RDa15:Increased viral clearnace (SS)

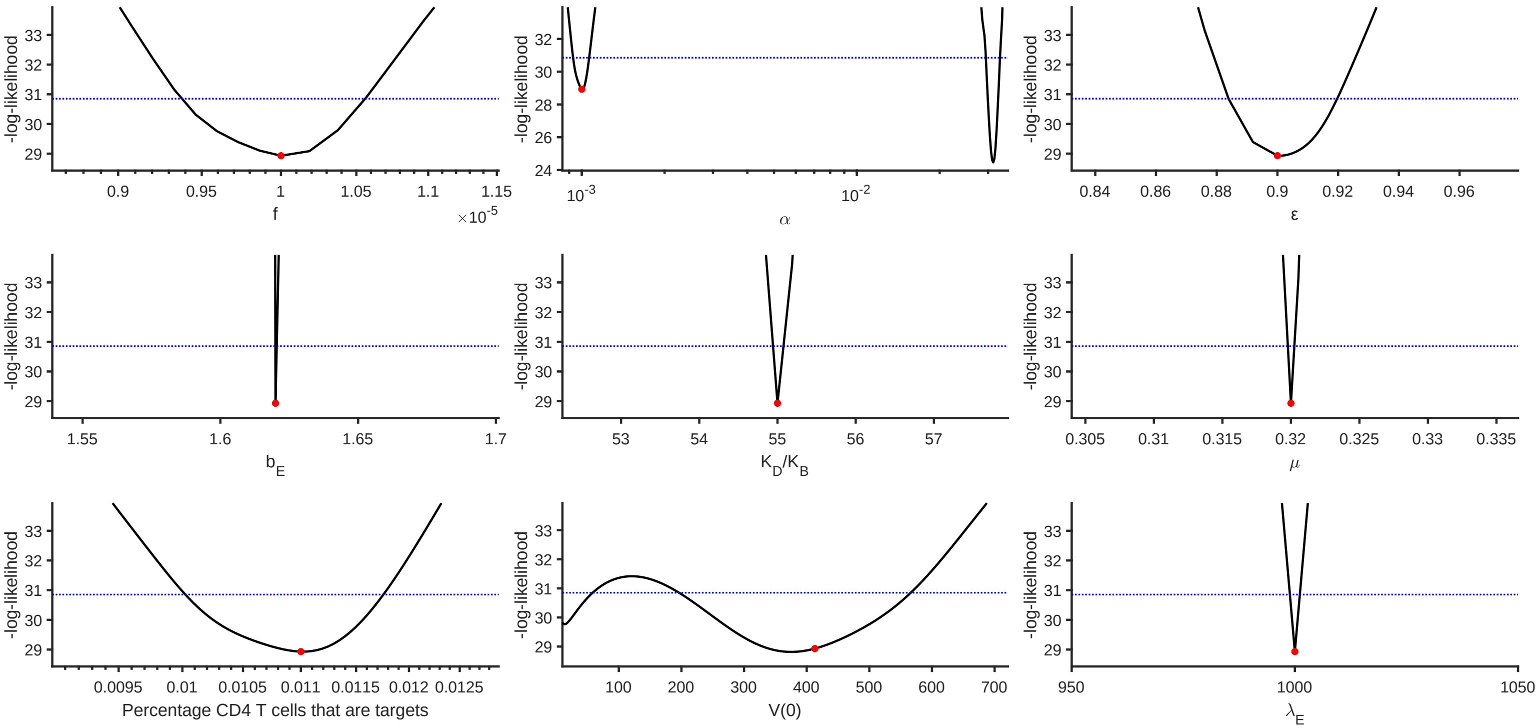

RFa15:Increased viral clearnace (SS)

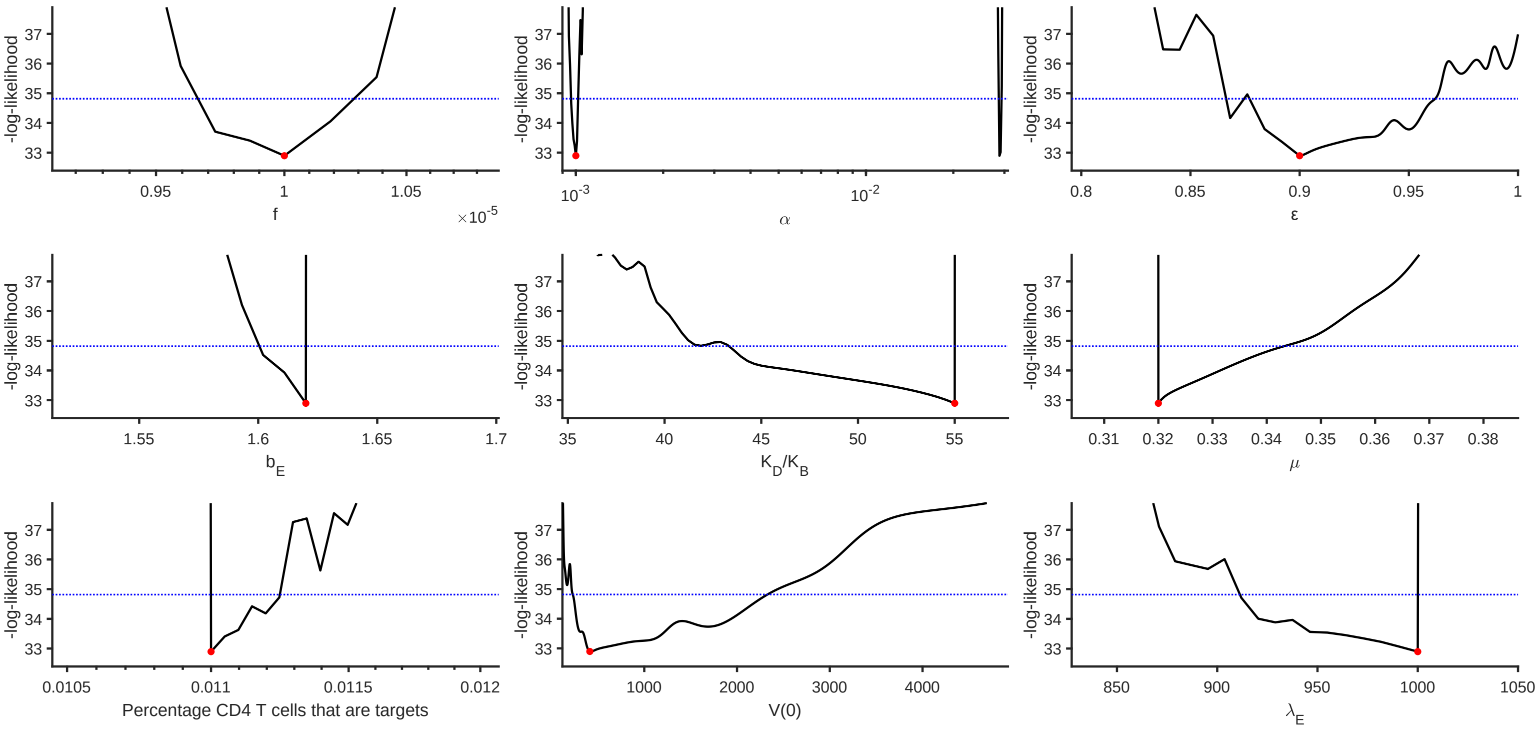

RLn12:Increased viral clearnace (SS)

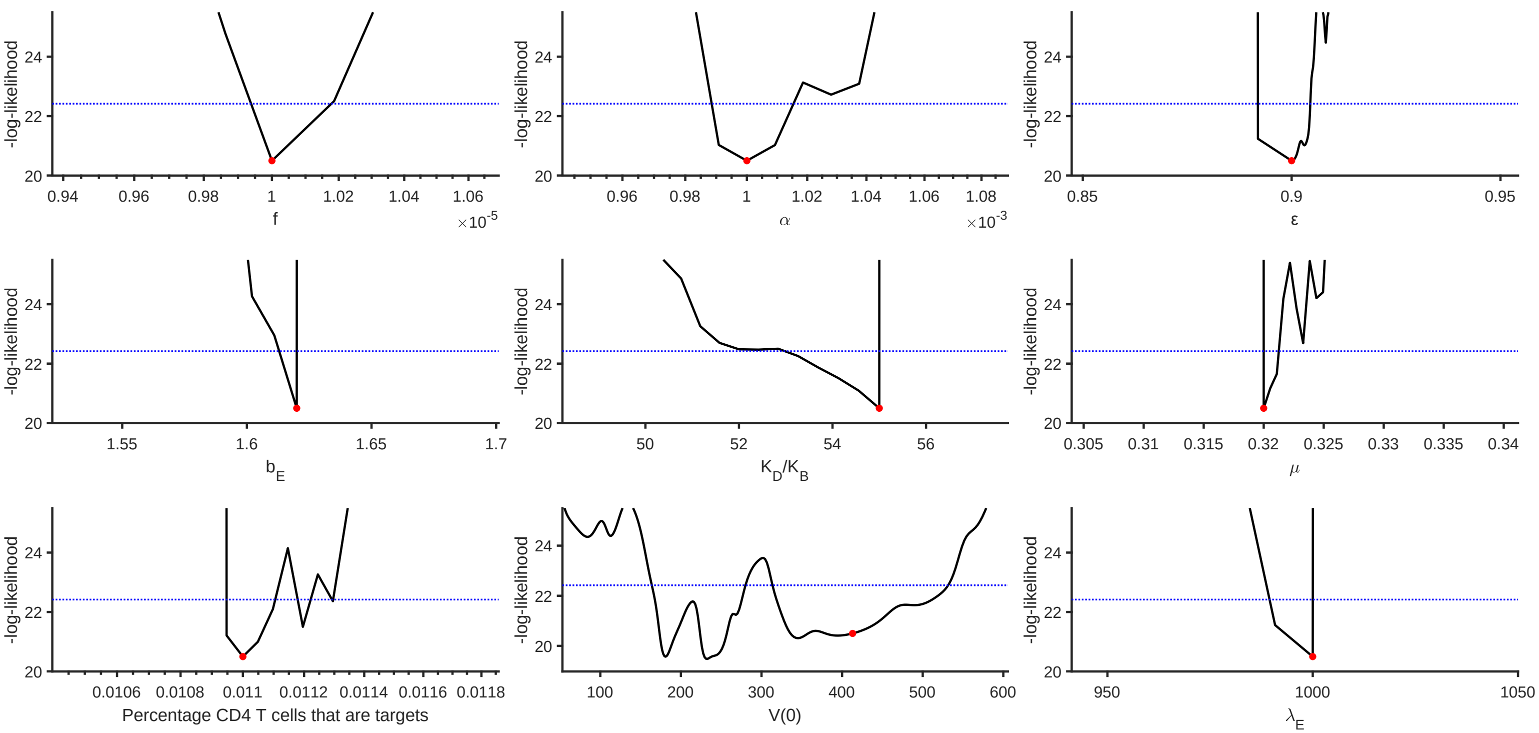

RId14:Increased viral clearnace (SS)

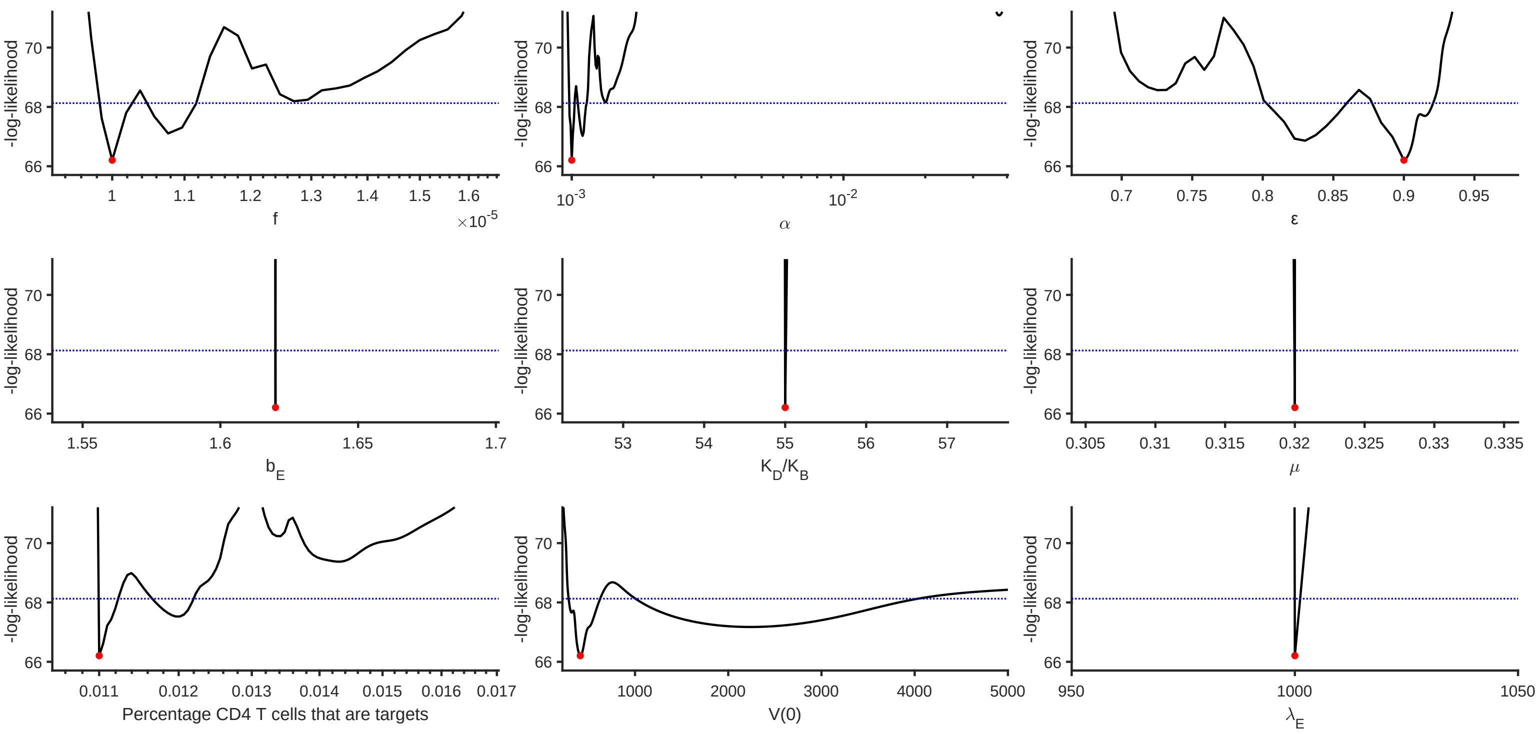

ROo13:Increased viral clearnace (SS)

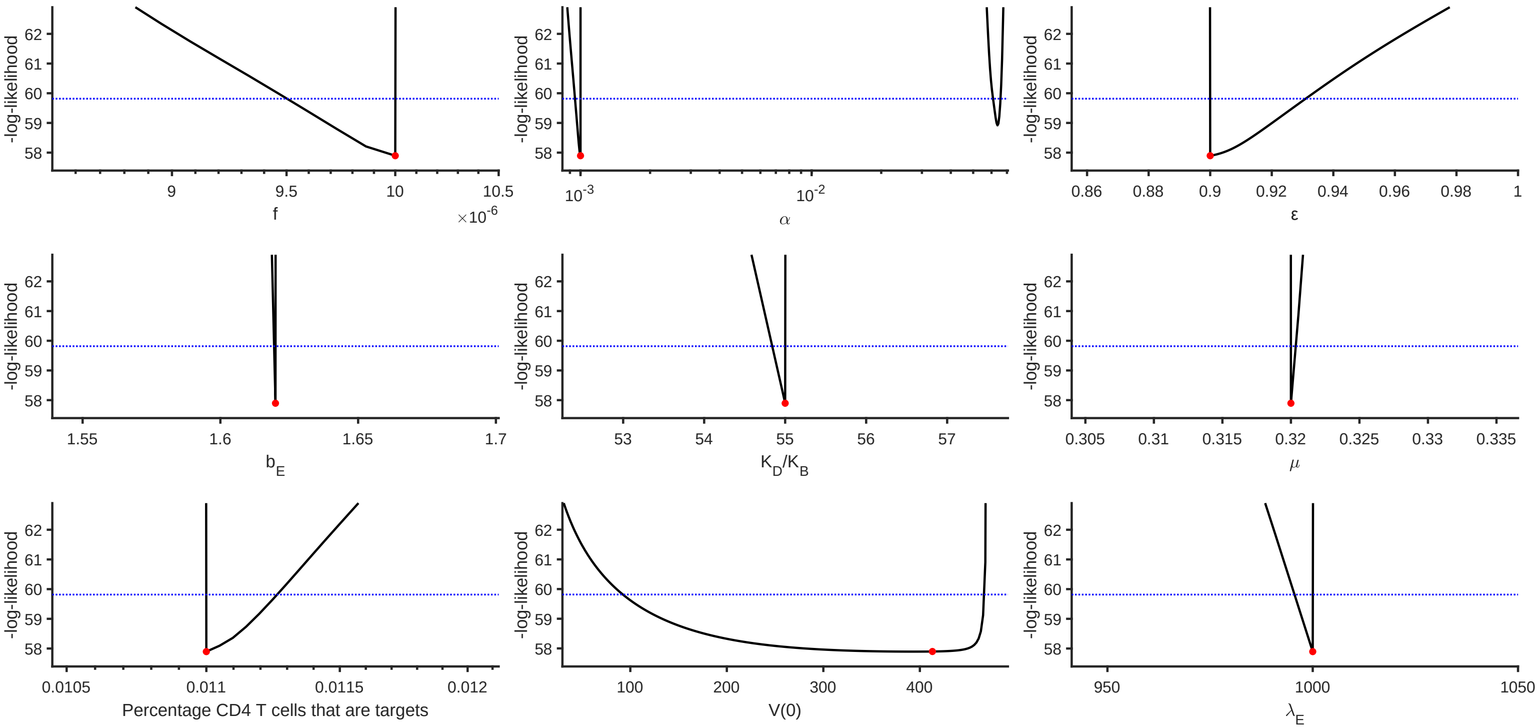

RSd14:Increased viral clearnace (SS)

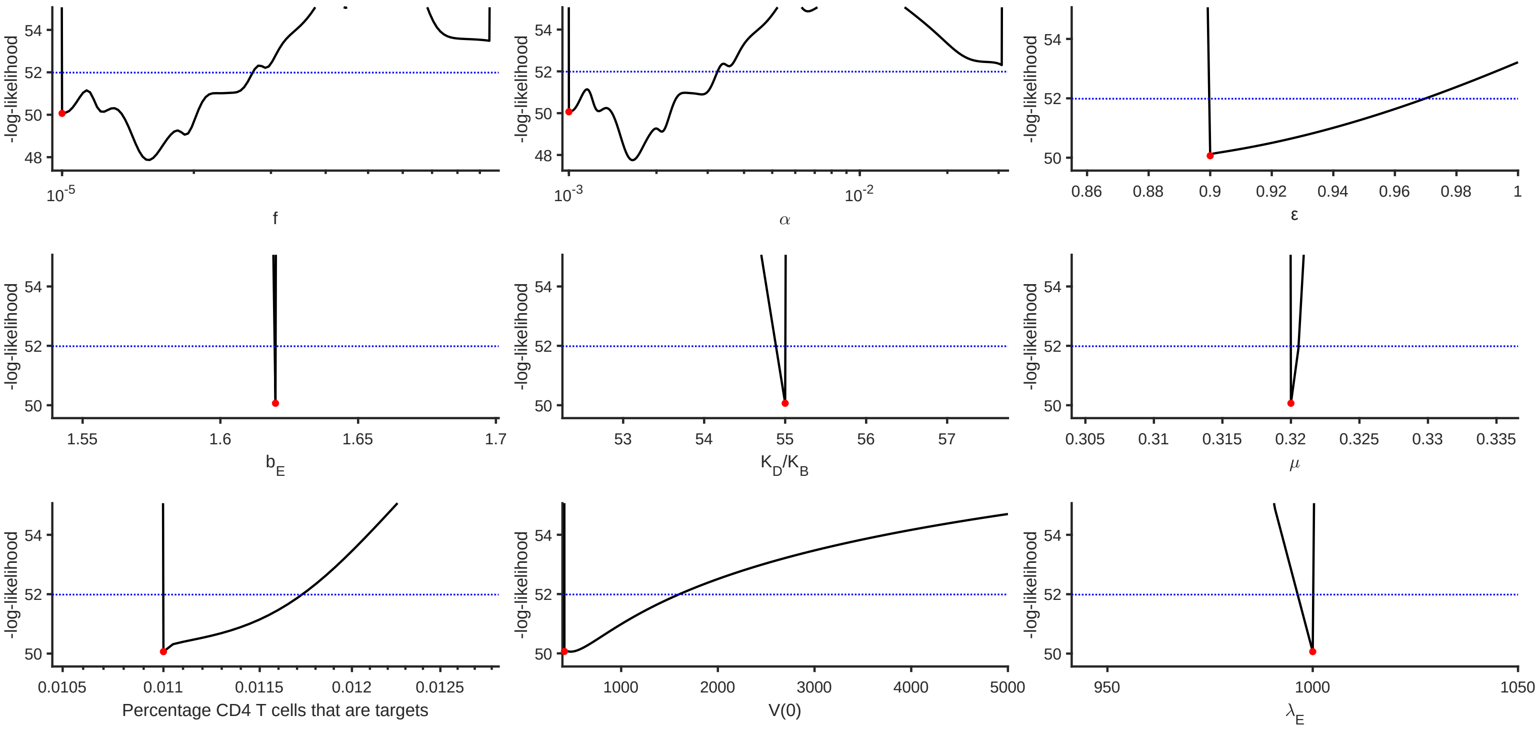

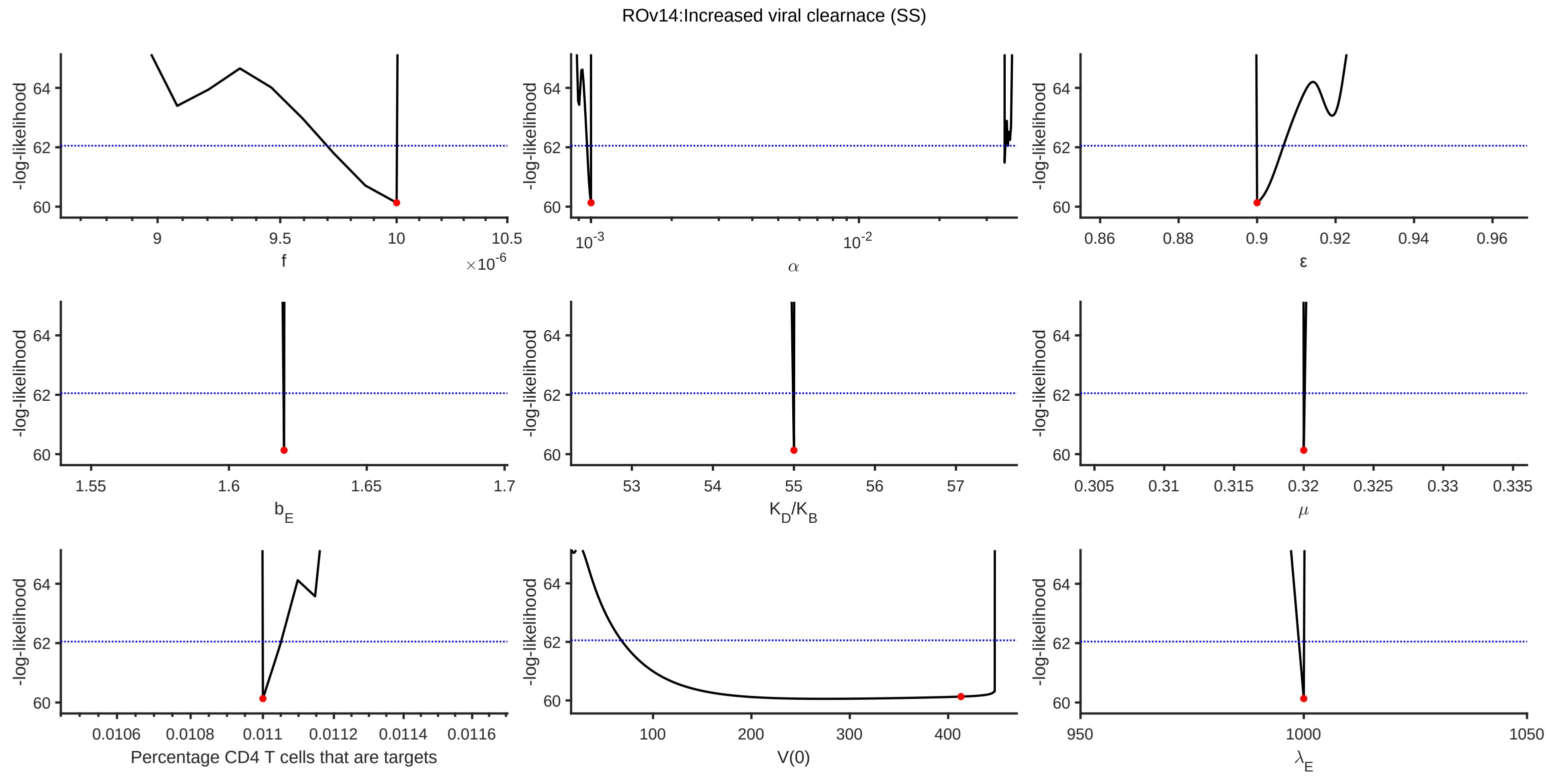
ROv14:Increased viral clearnace (SS)
-log-likelihood
f
×10^-6
α
ε
b_E
K_D/K_B
μ
Percentage CD4 T cells that are targets
V(0)
λ_E

4.3 Antigen presenting cell effector cell source model

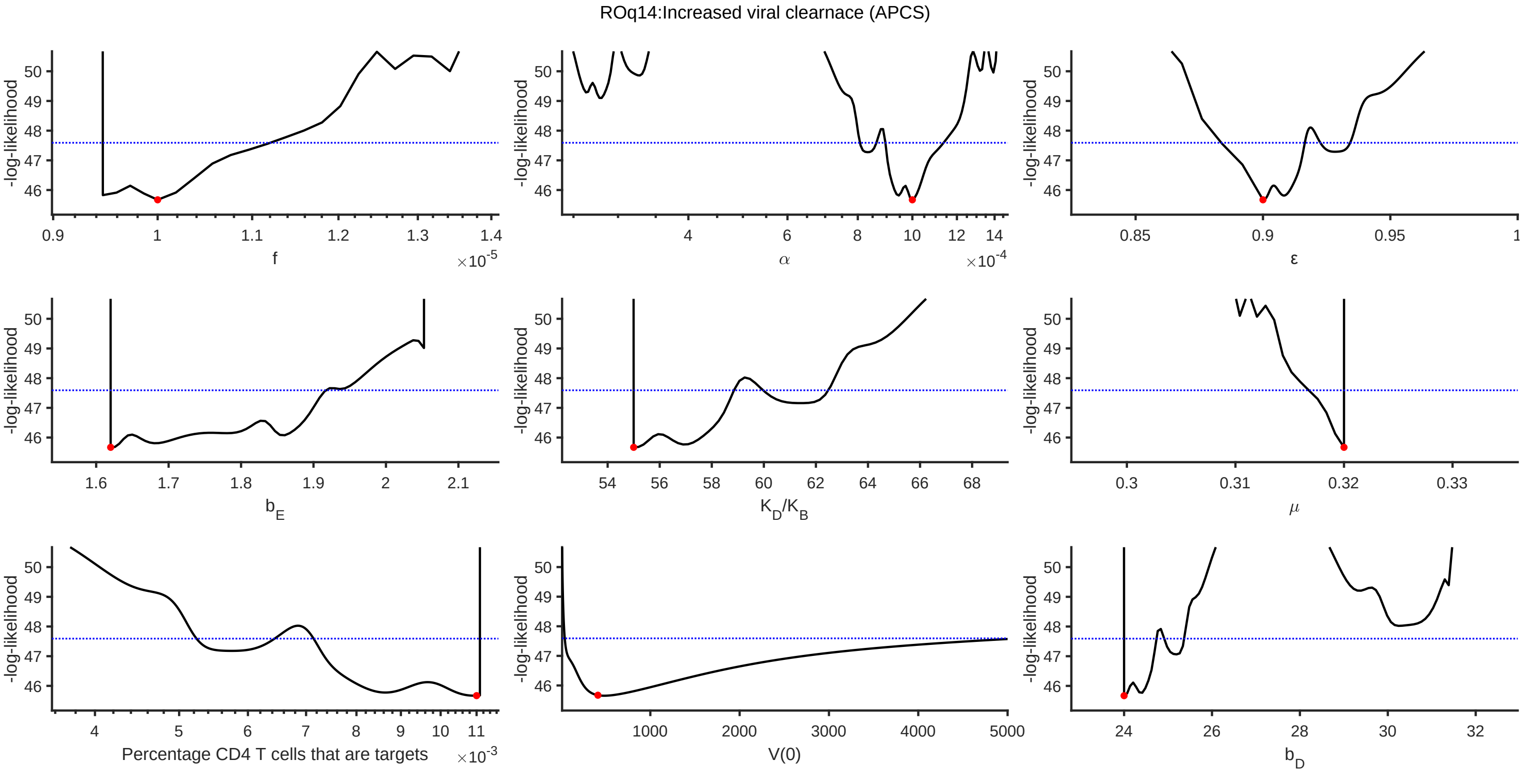

RDa15:Increased viral clearnace (APCS)

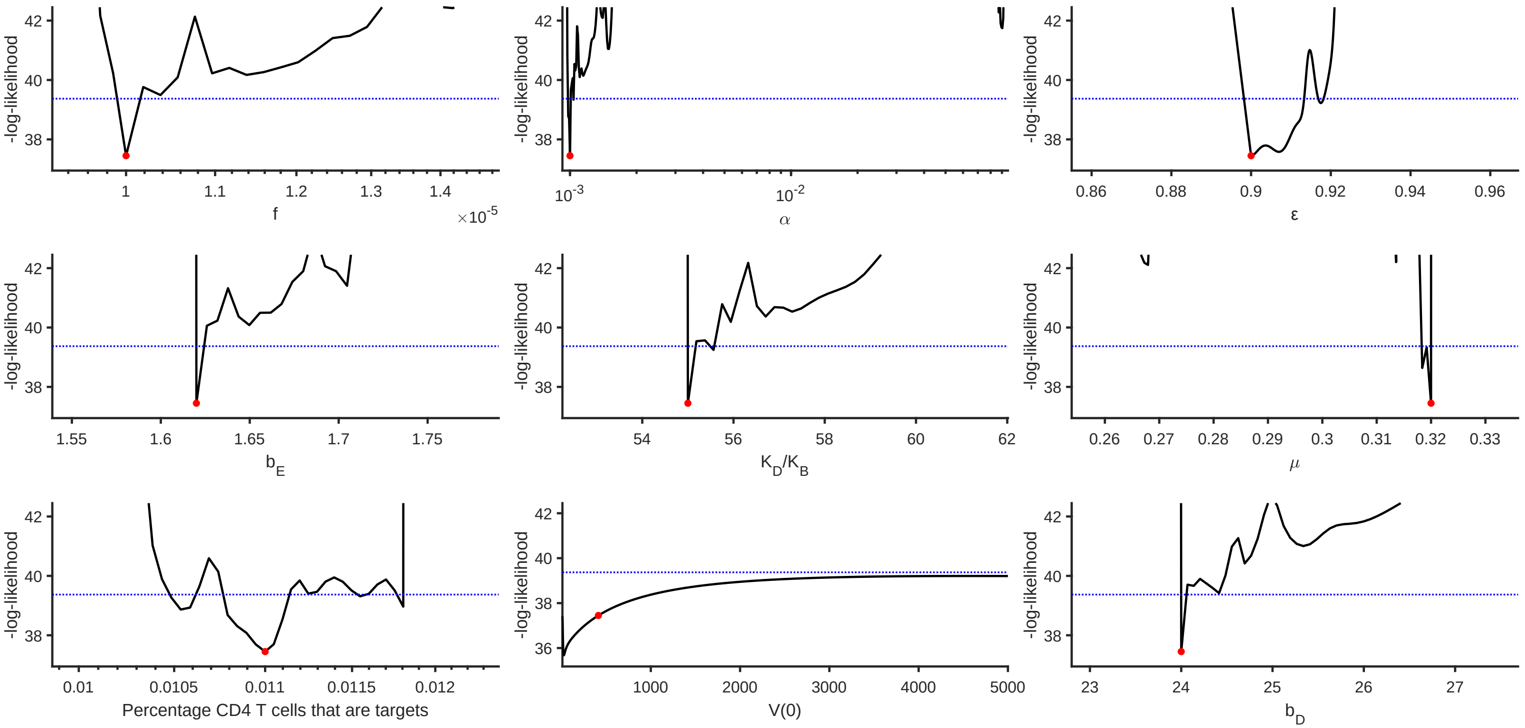

RFa15:Increased viral clearnace (APCS)

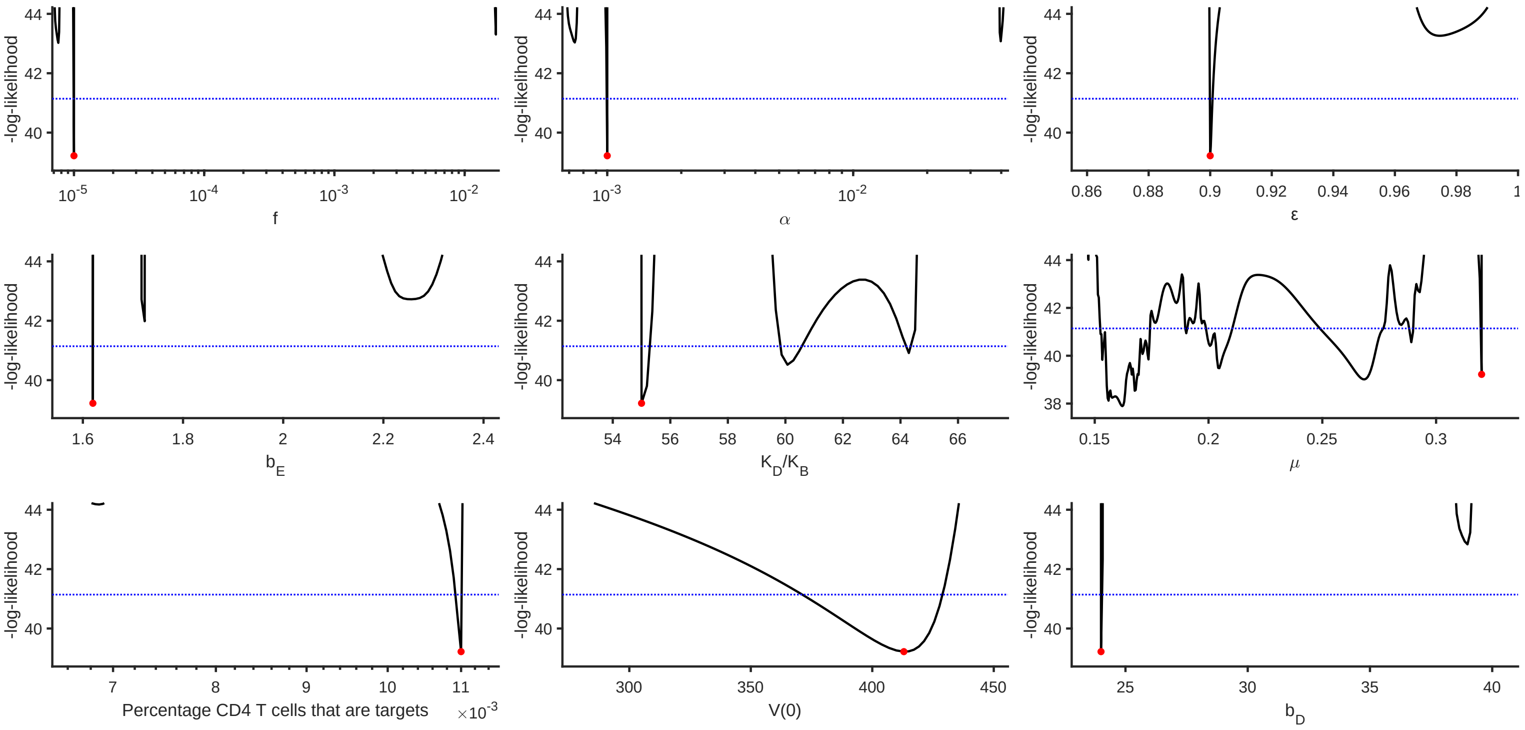

RLn12:Increased viral clearnace (APCS)

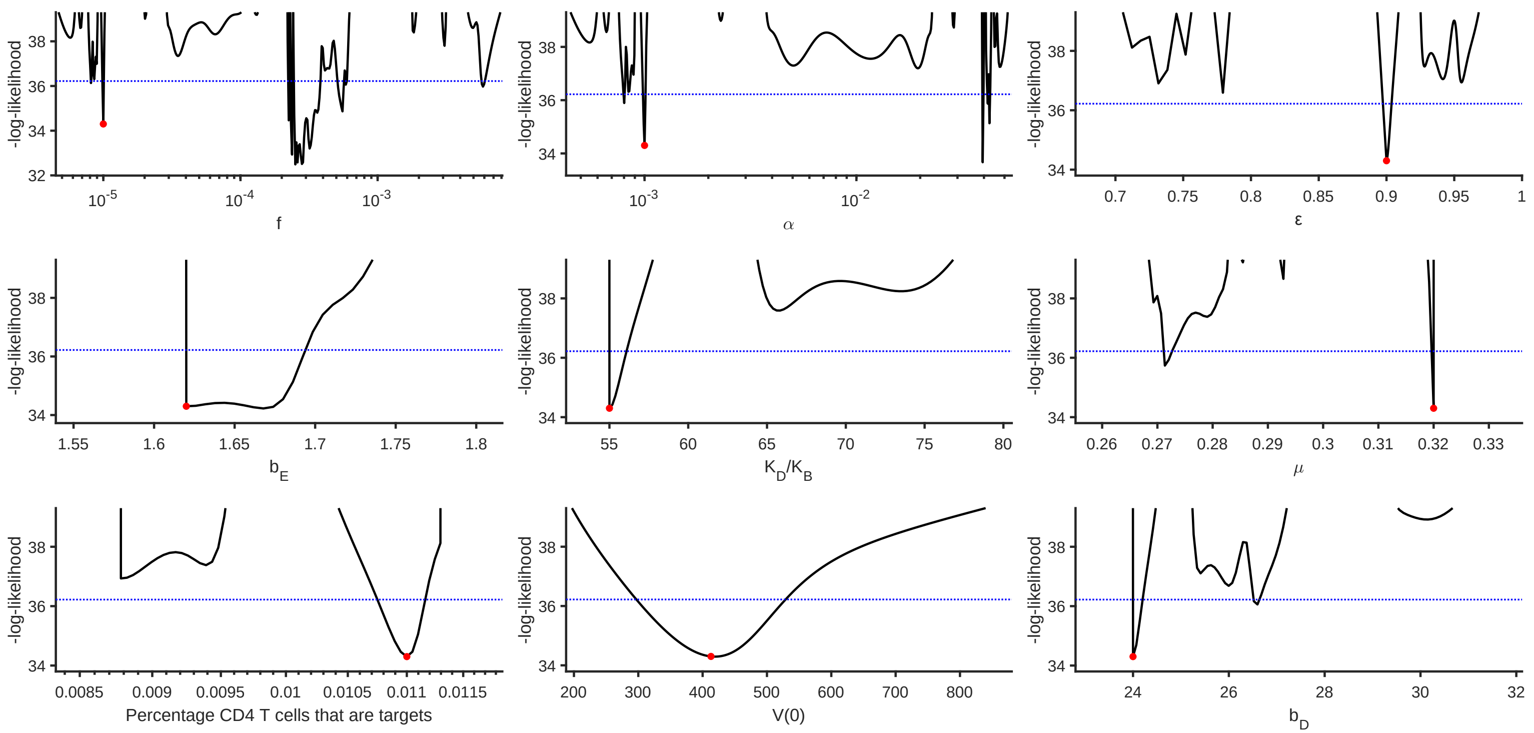

RId14:Increased viral clearnace (APCS)

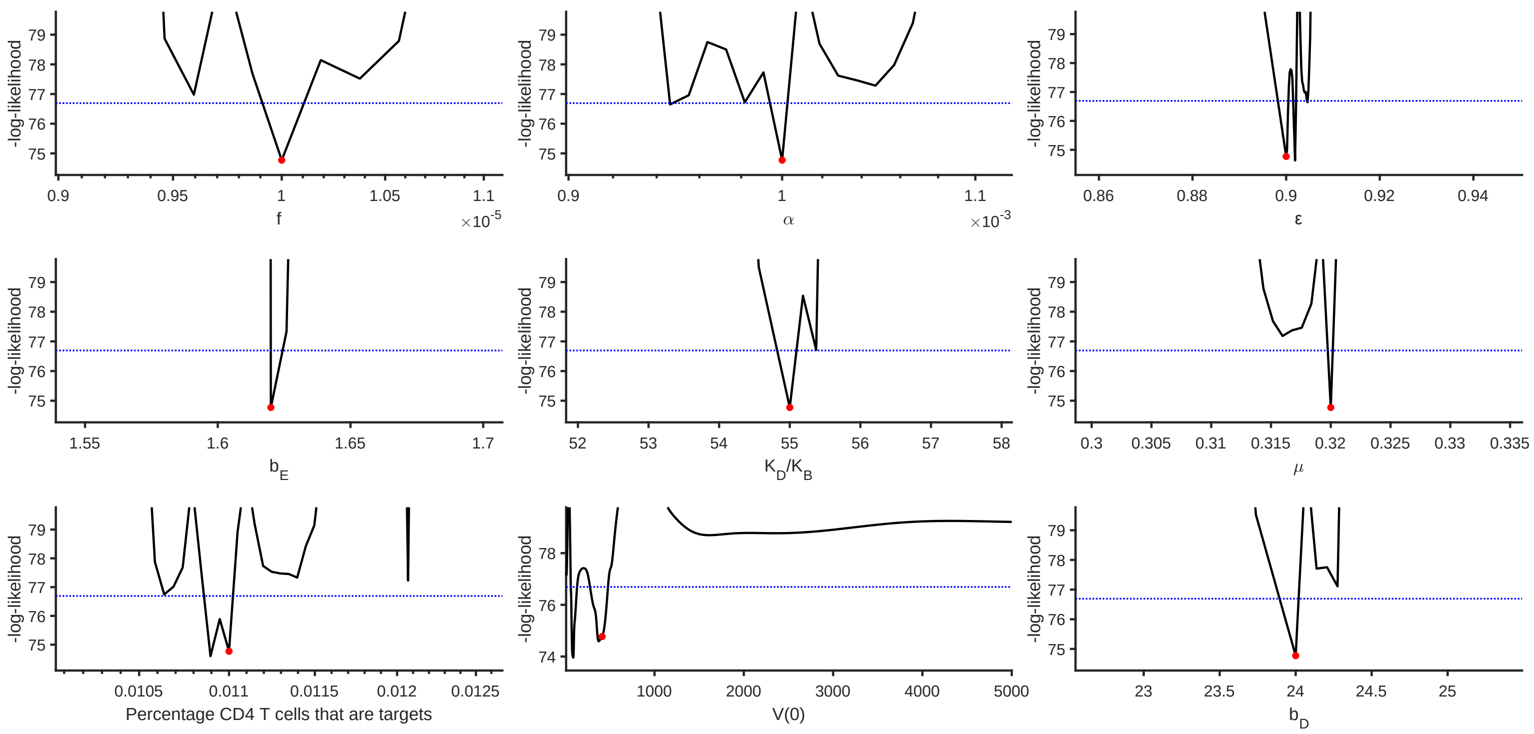

ROo13:Increased viral clearnace (APCS)

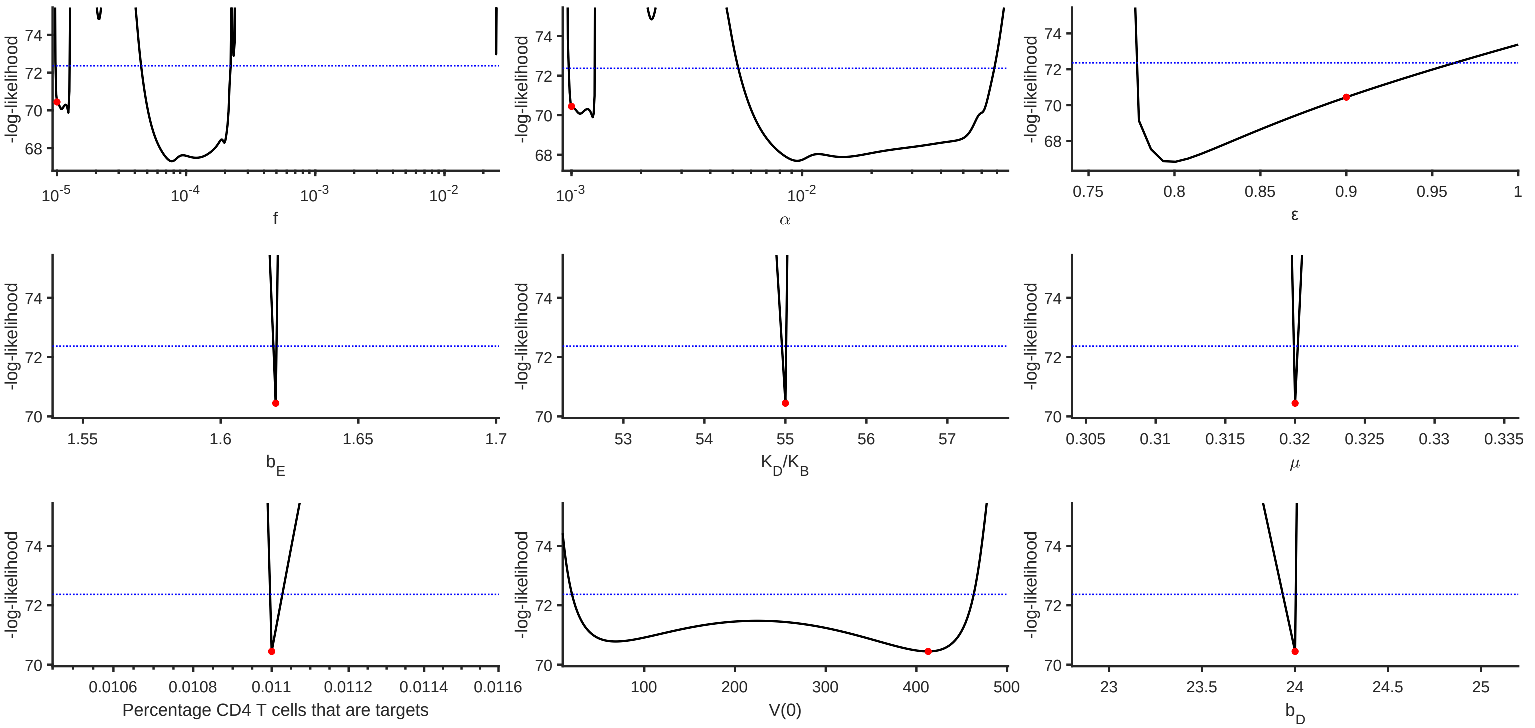

RSd14:Increased viral clearnace (APCS)

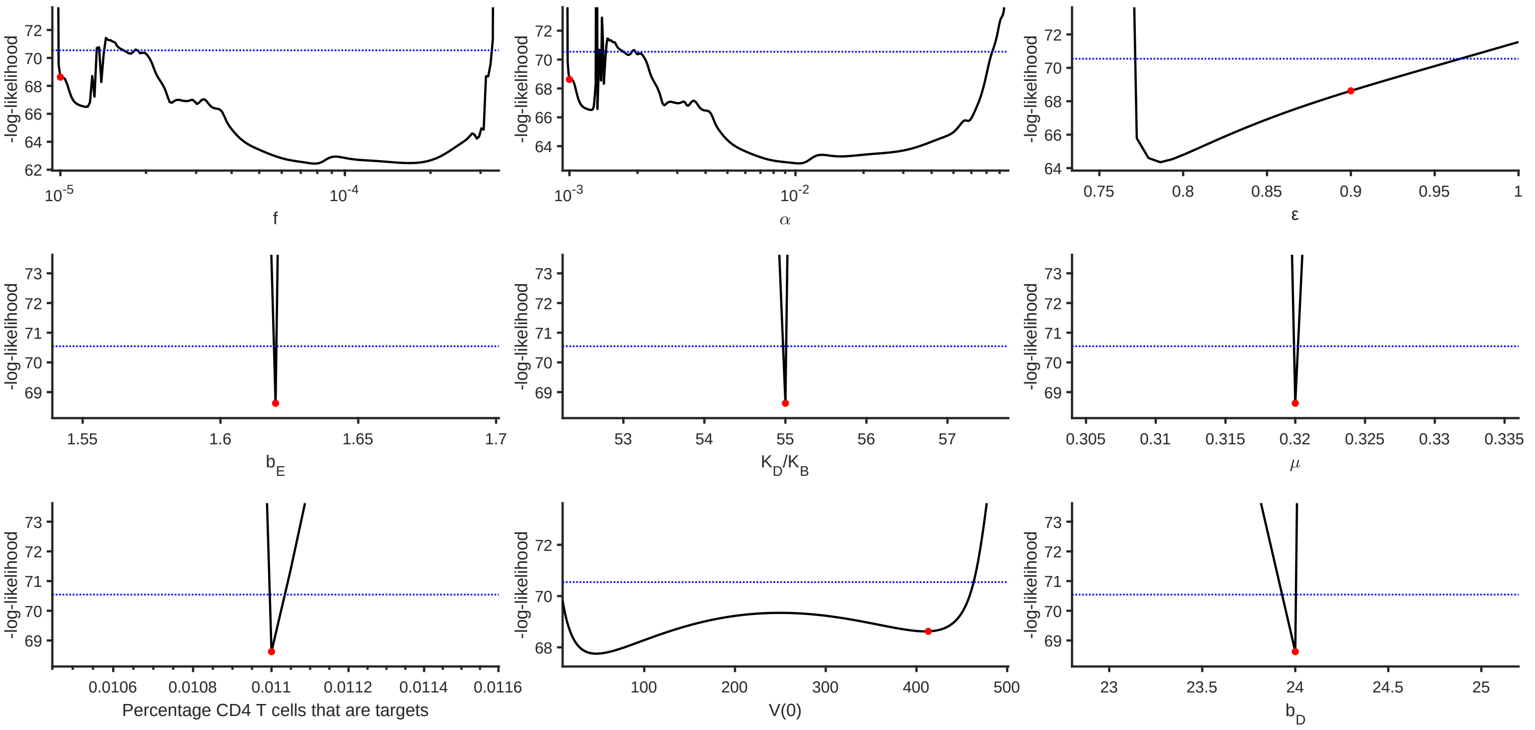

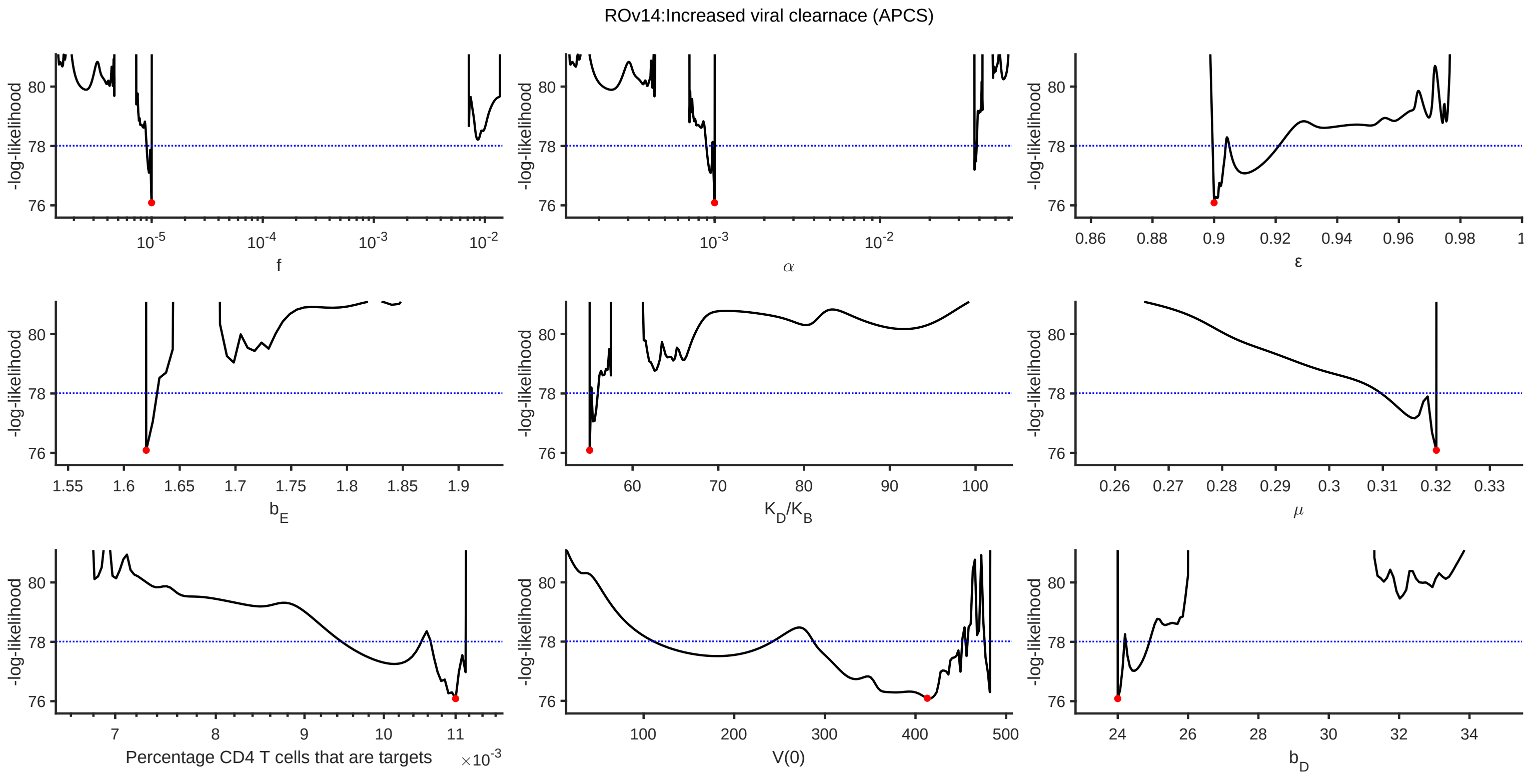

ROv14:Increased viral clearnace (APCS)
-log-likelihood
f
α
ε
b_E
K_D/K_B
μ
Percentage CD4 T cells that are targets
×10^-3
V(0)
b_D

5 Likelihood profiles:Viral neutralization

5.1 Baseline effector cell source model

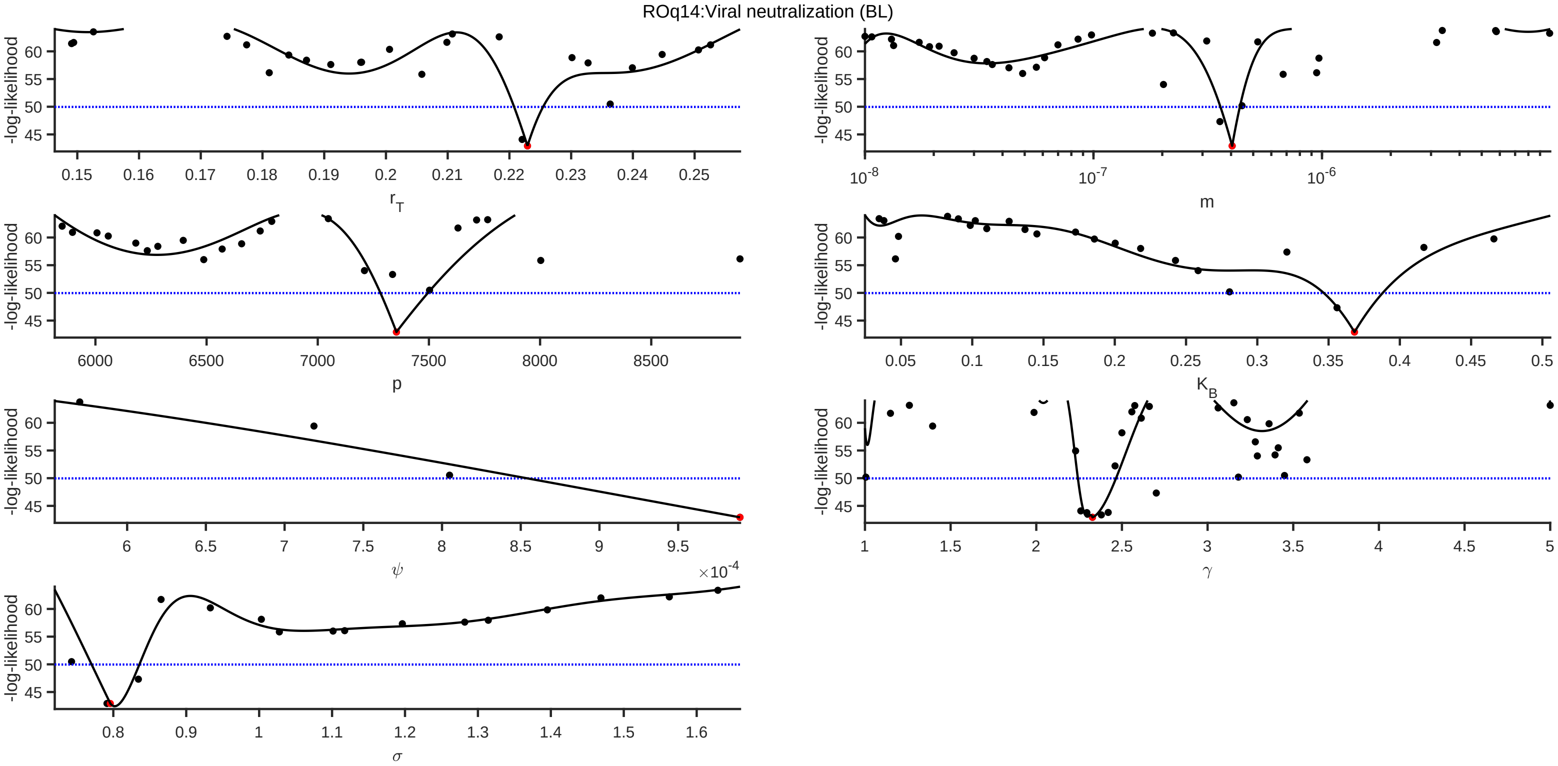

RDa15:Viral neutralization (BL)

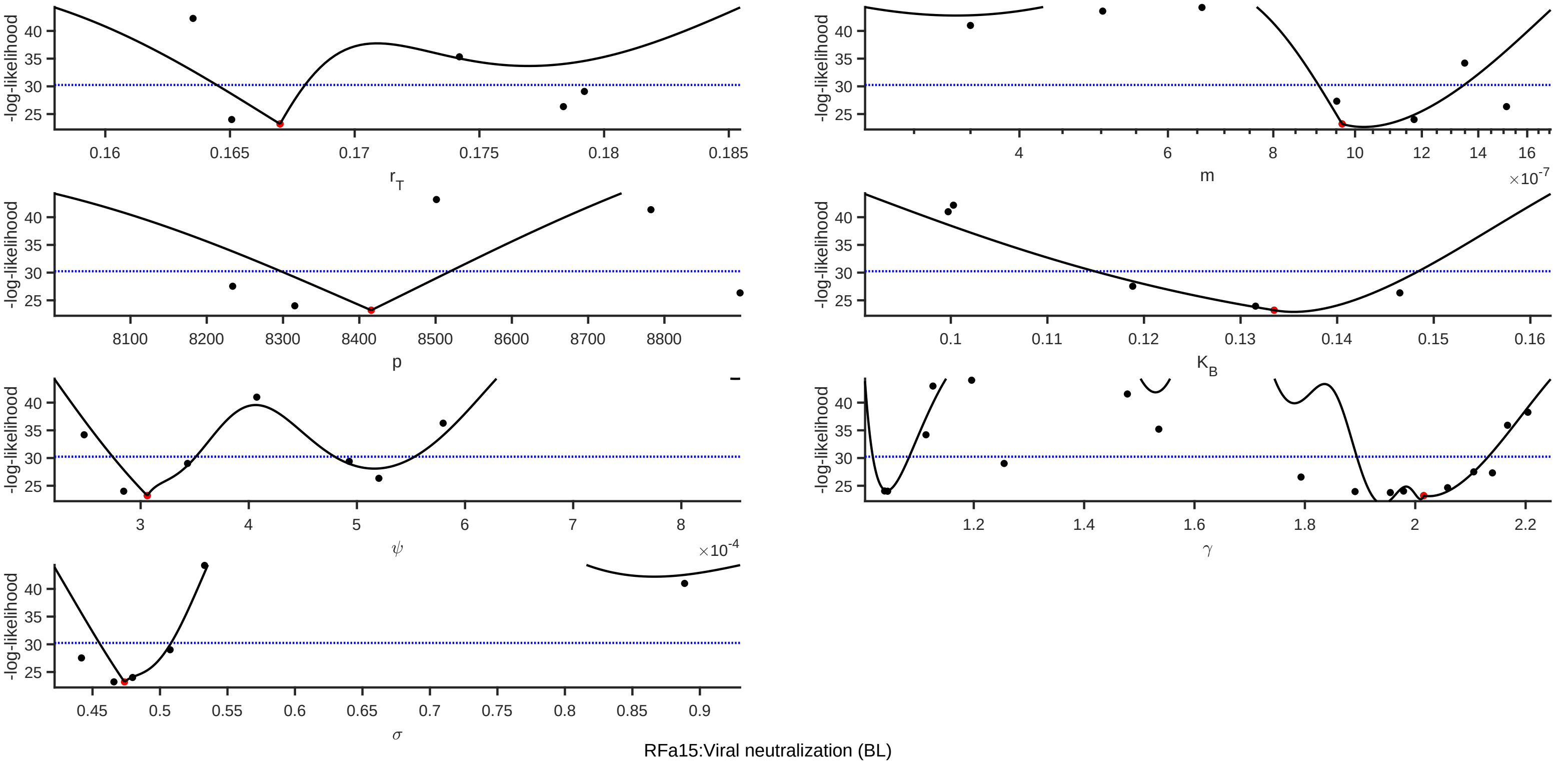

RFa15:Viral neutralization (BL)

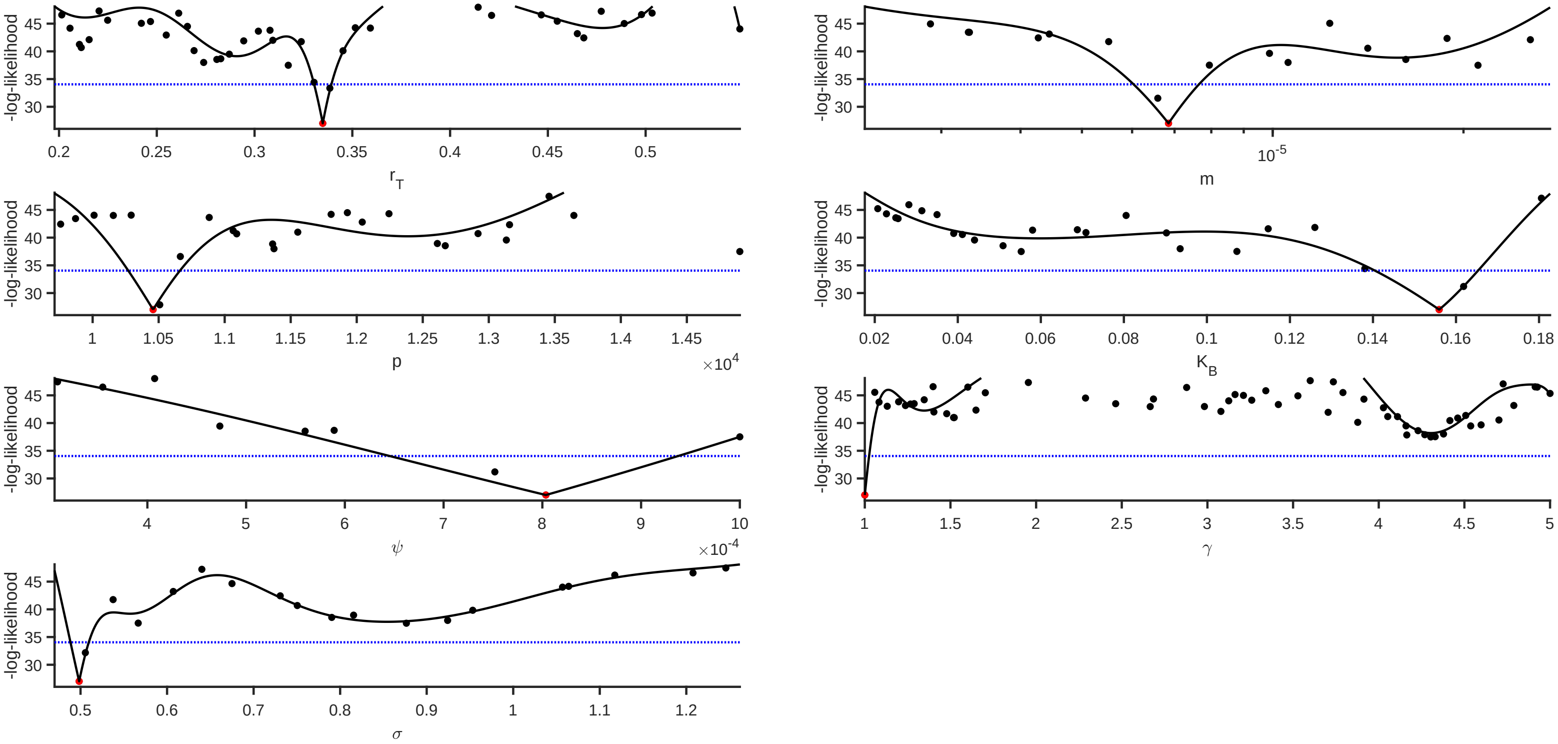

RLn12:Viral neutralization (BL)

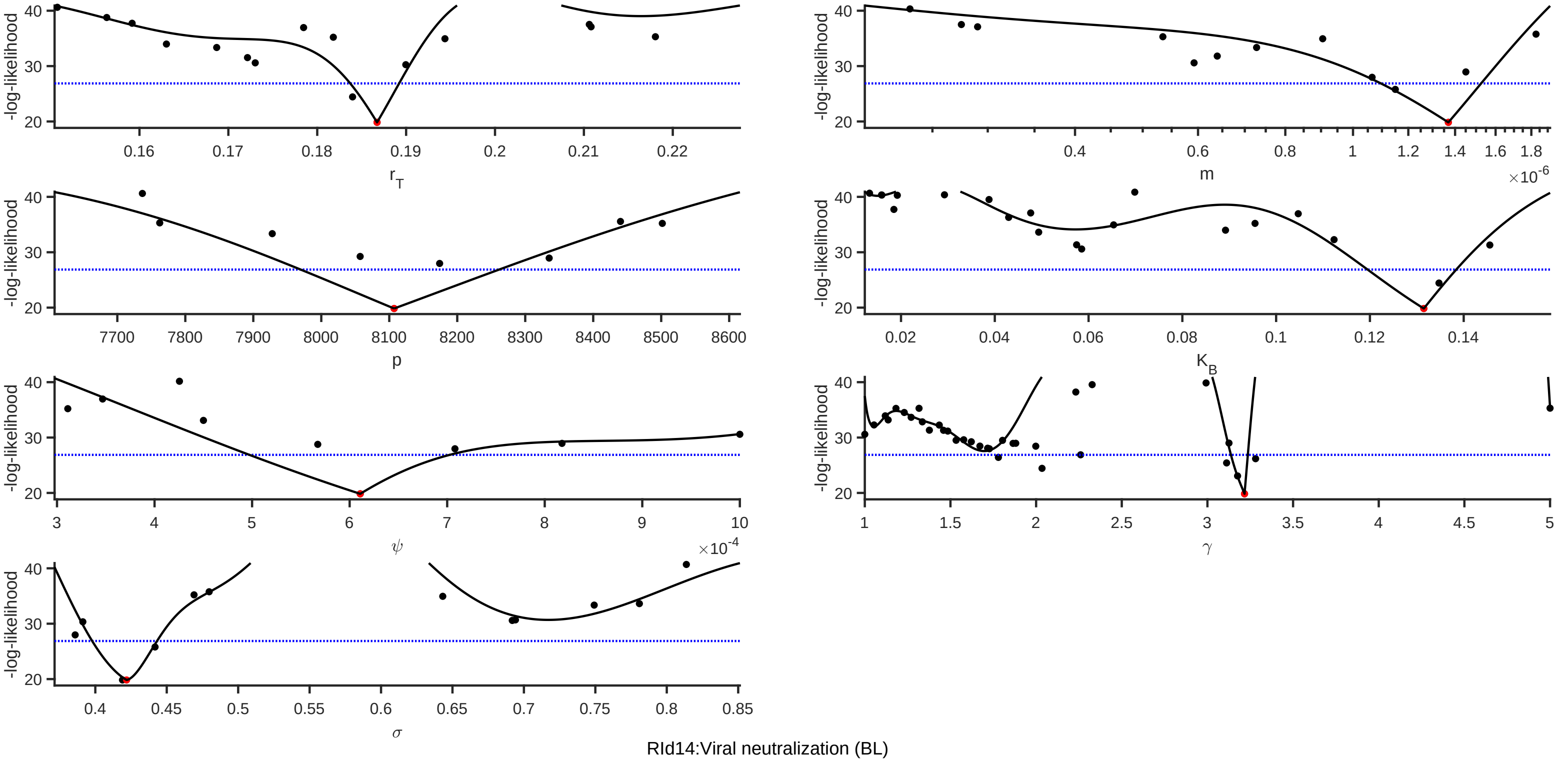

RId14:Viral neutralization (BL)

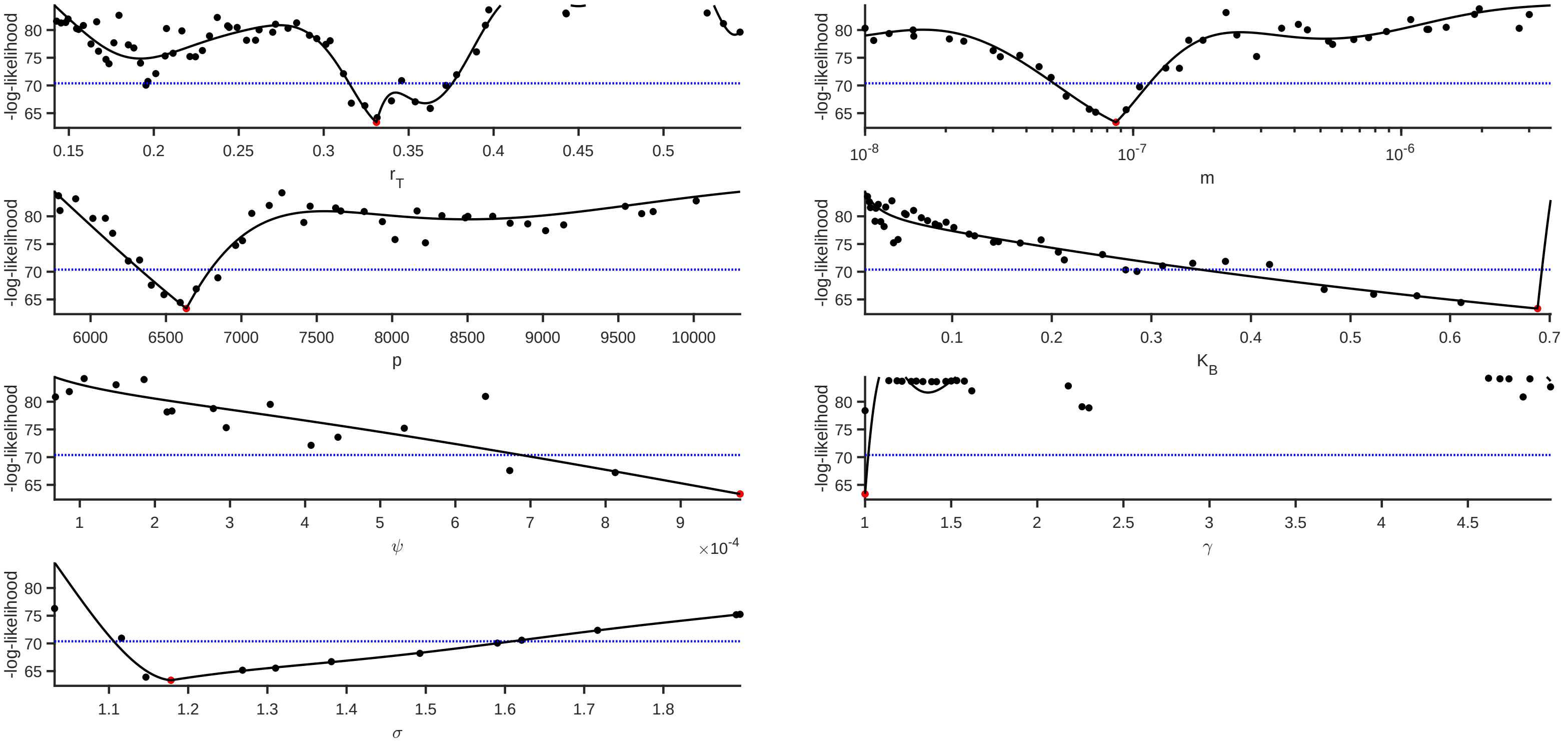

ROo13:Viral neutralization (BL)

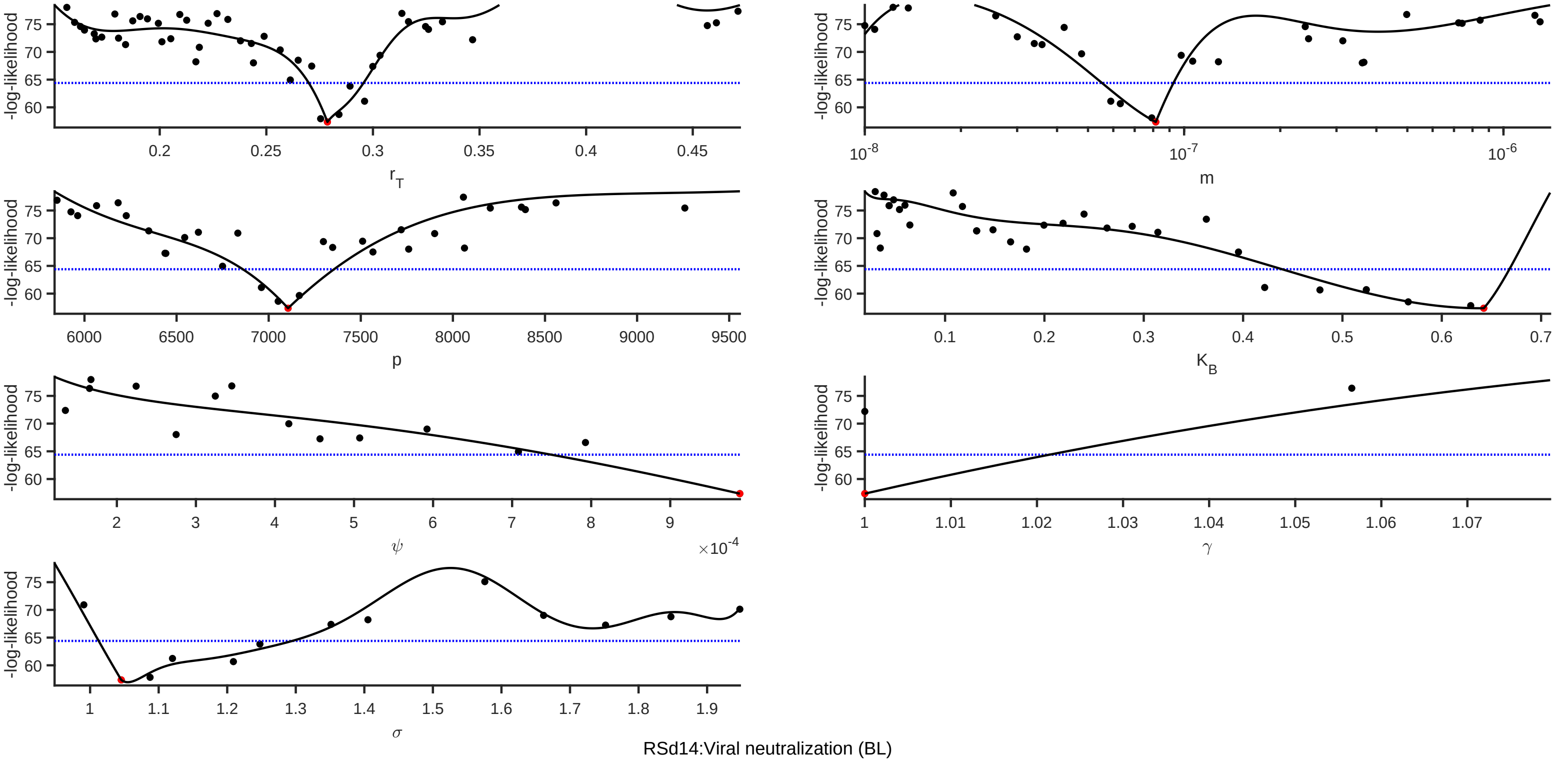

RSd14:Viral neutralization (BL)

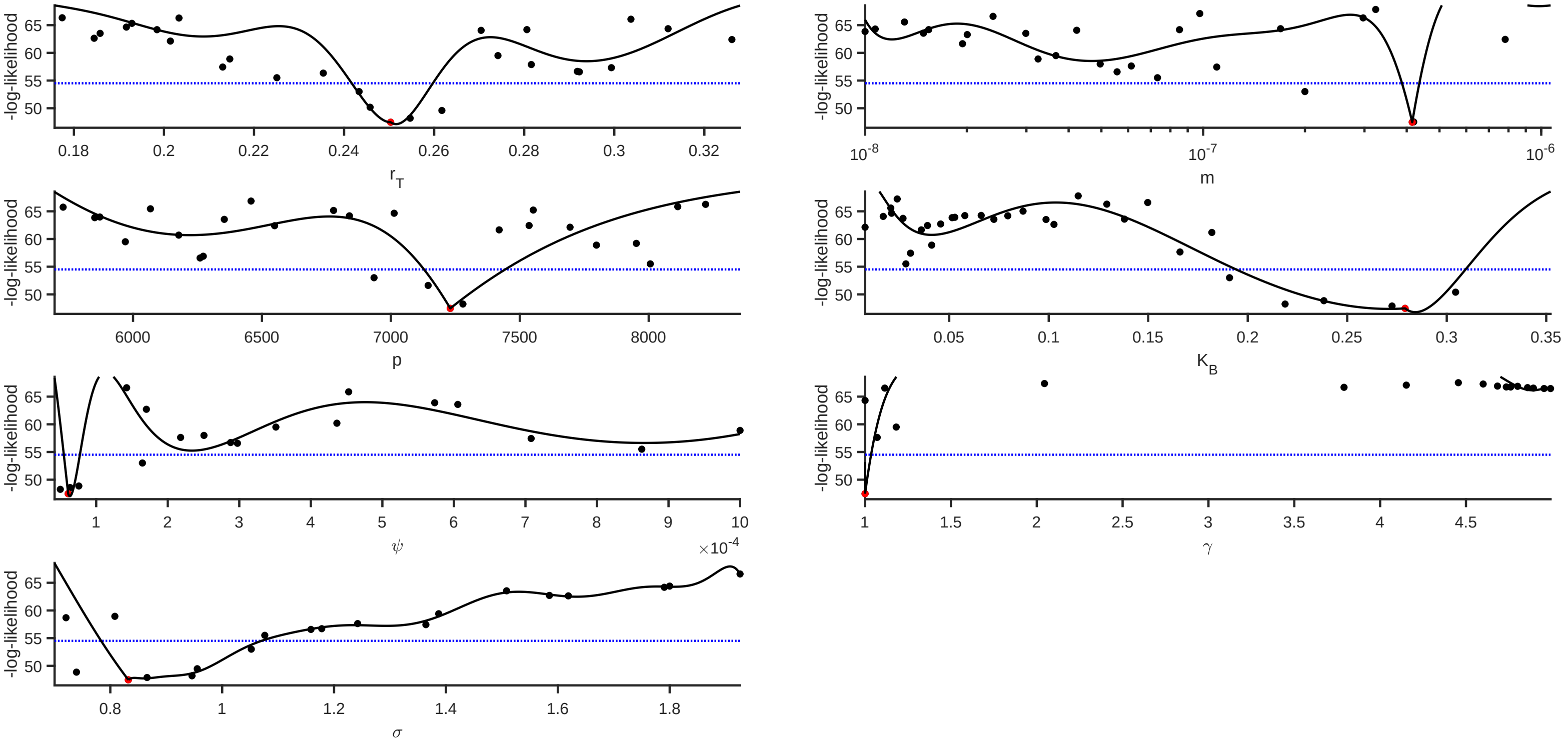

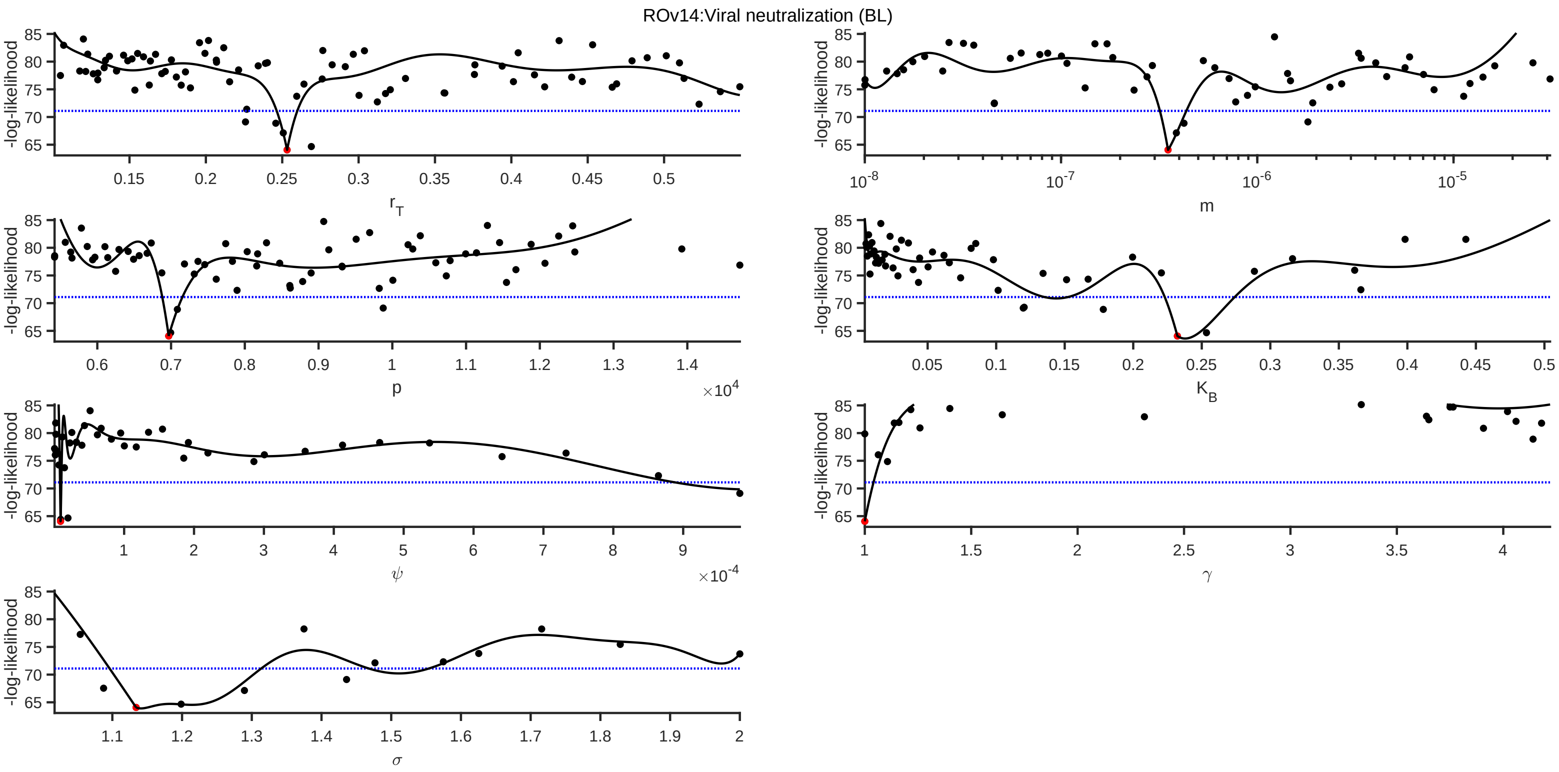
ROv14:Viral neutralization (BL)
-log-likelihood
r_T
m
p
K_B
$\times 10^4$
ψ
γ
$\times 10^{-4}$
σ

5.2 Saturated effector cell source model

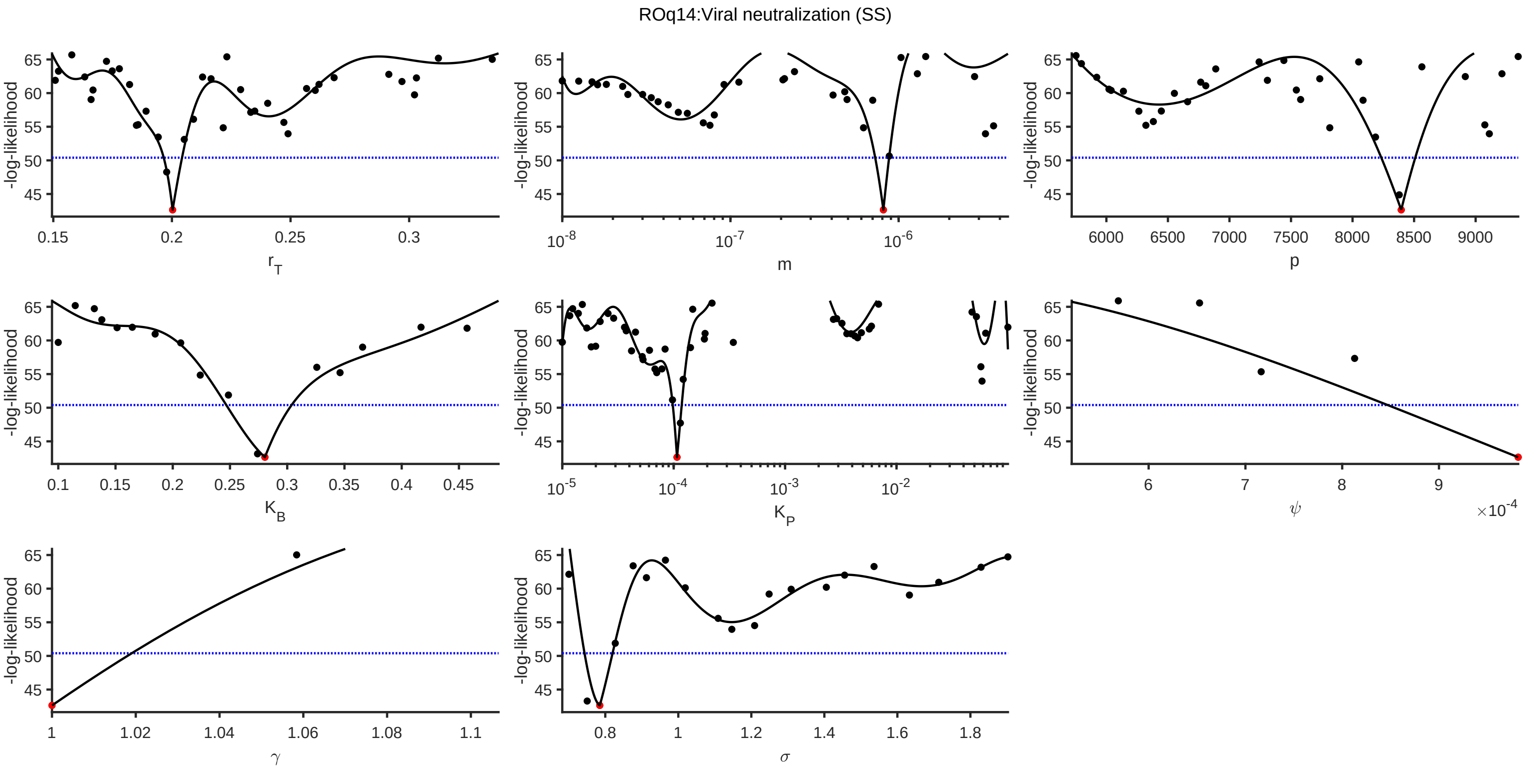

RDa15:Viral neutralization (SS)

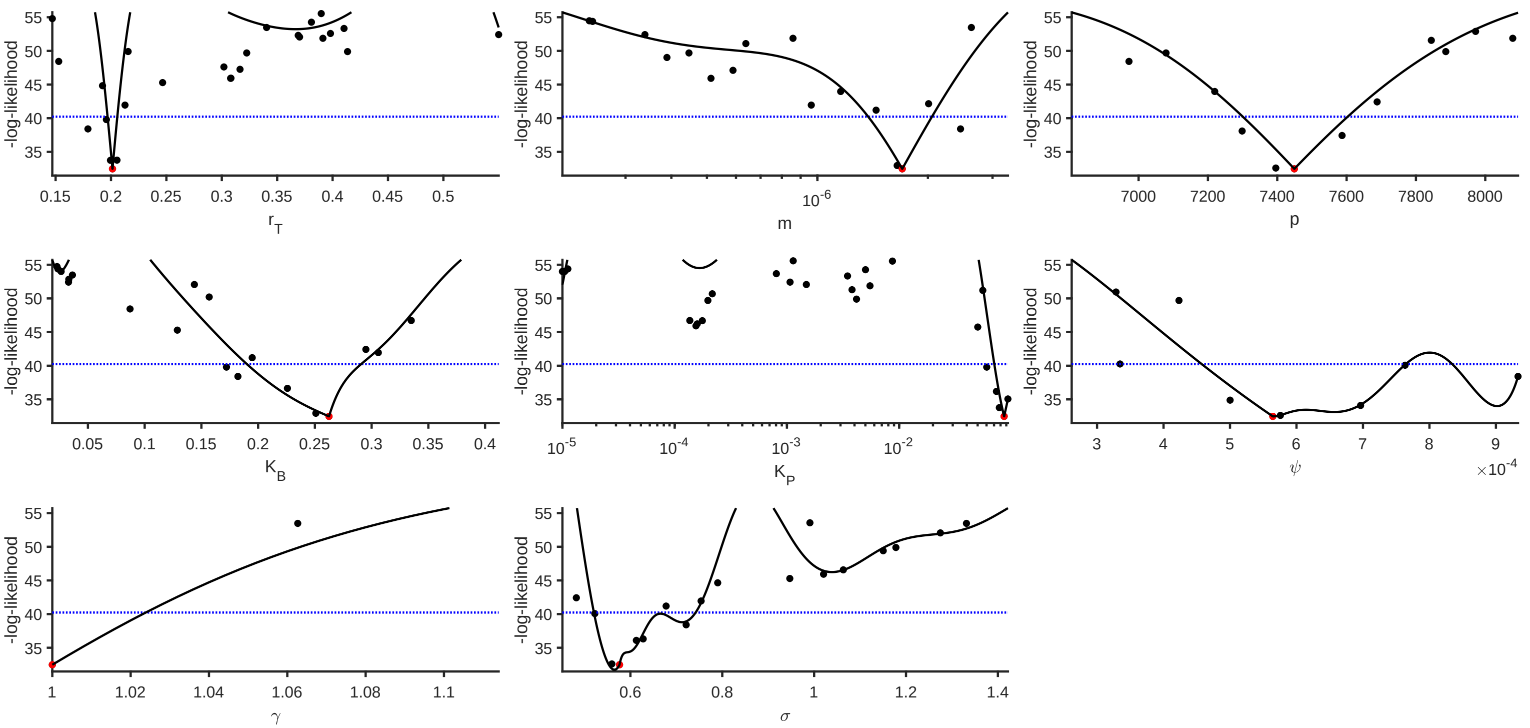

RFa15:Viral neutralization (SS)

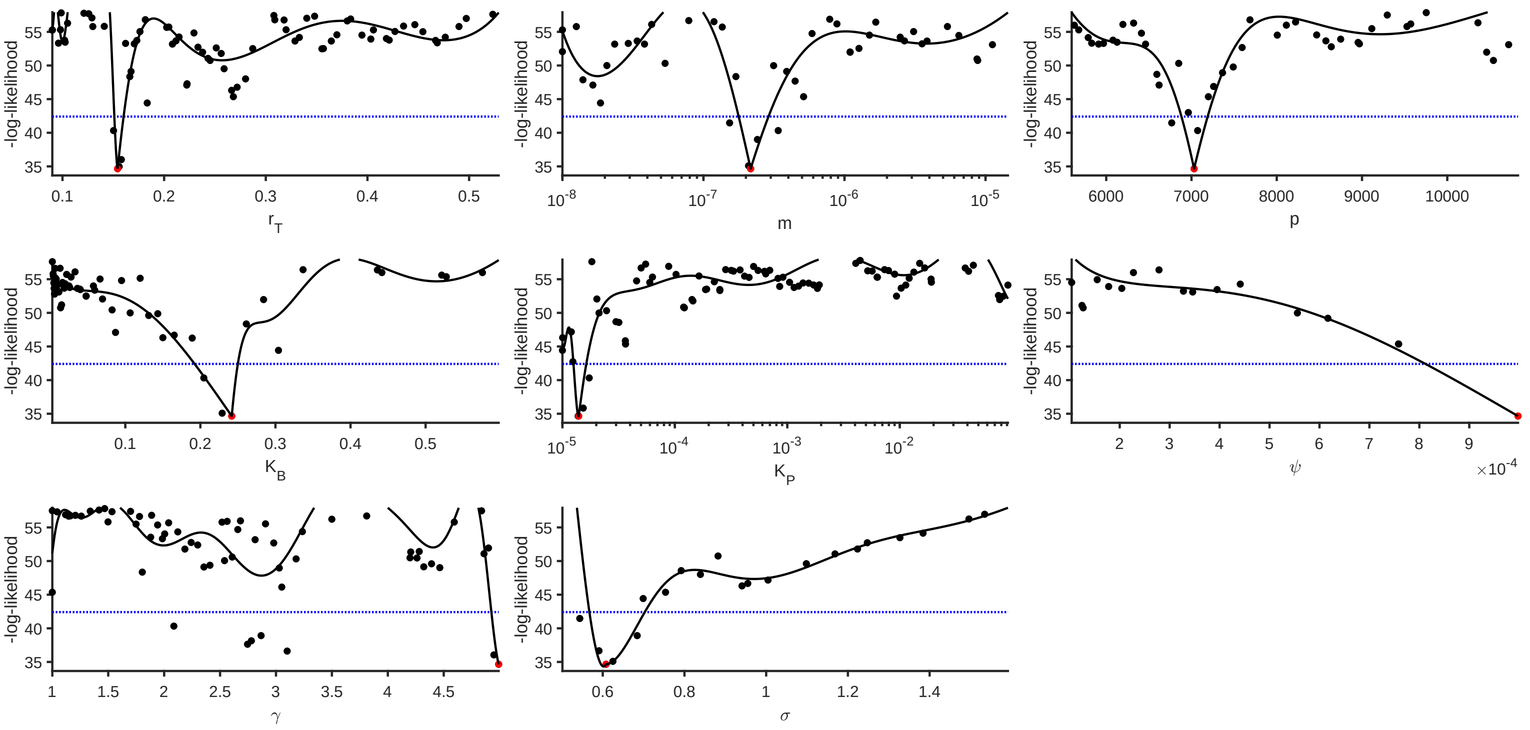

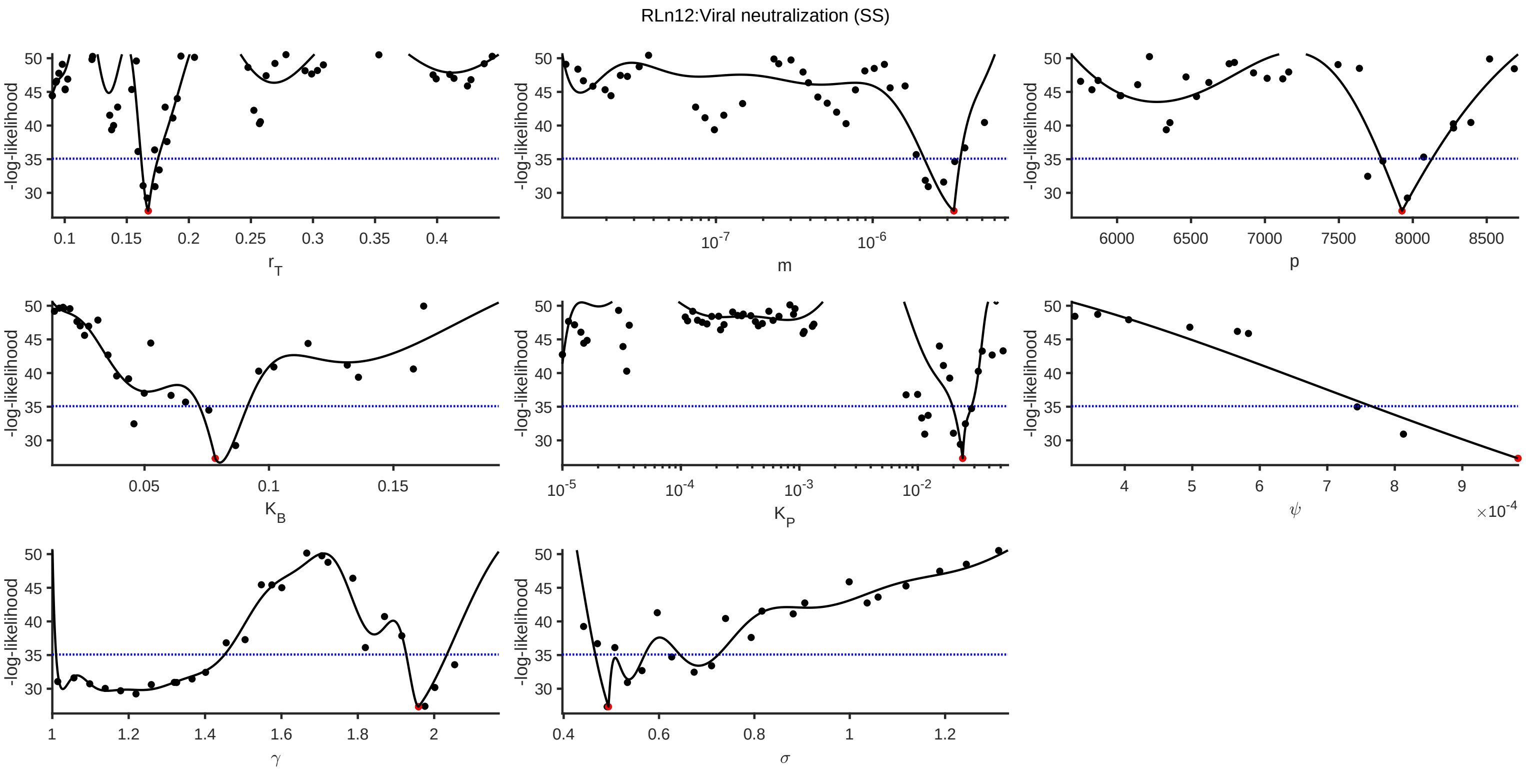
RLn12:Viral neutralization (SS)
-log-likelihood
r_T
m
p
K_B
K_P
ψ
×10^-4
γ
σ

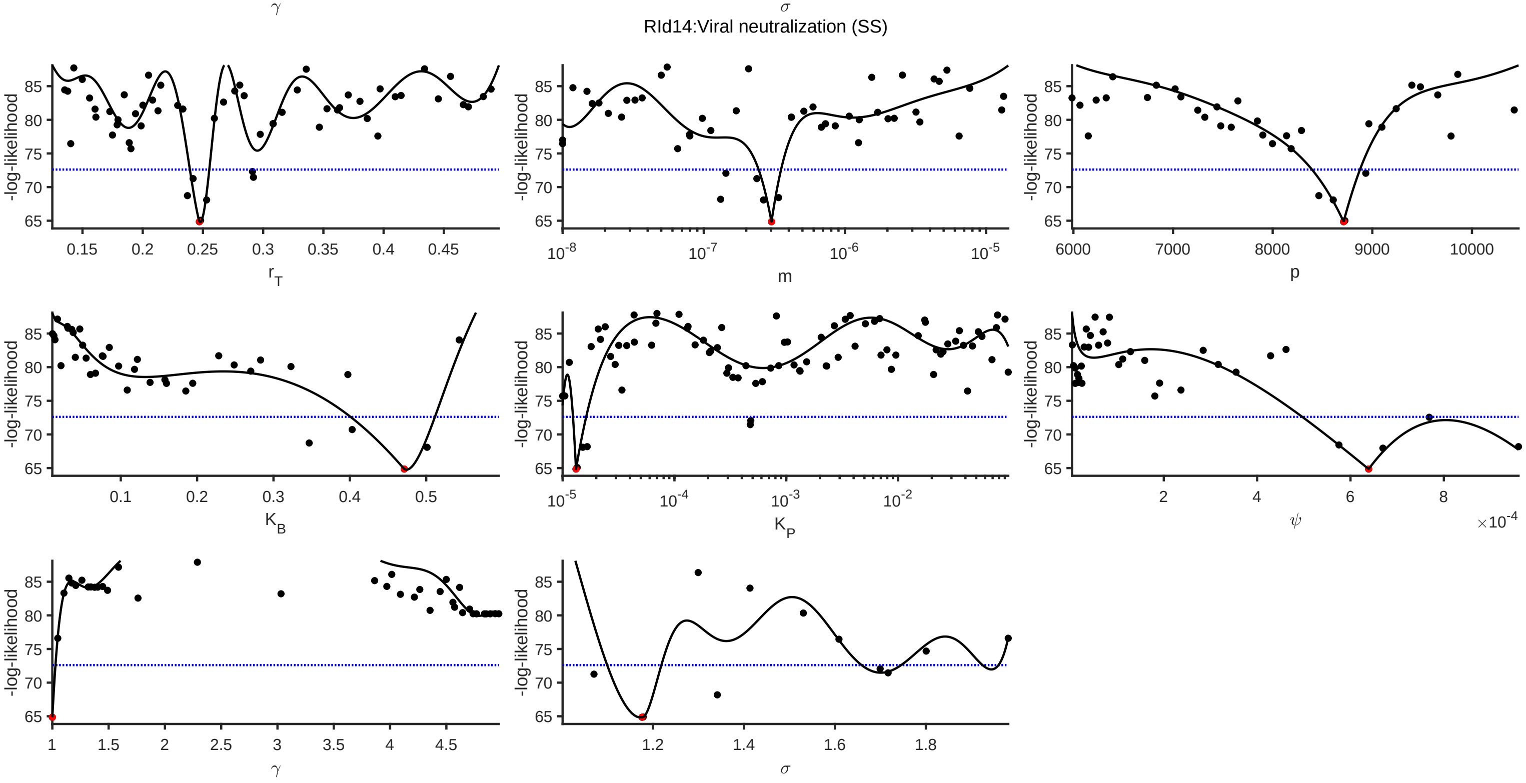
RId14:Viral neutralization (SS)
-log-likelihood
r_T
m
p
K_B
K_P
ψ
×10^-4
γ
σ

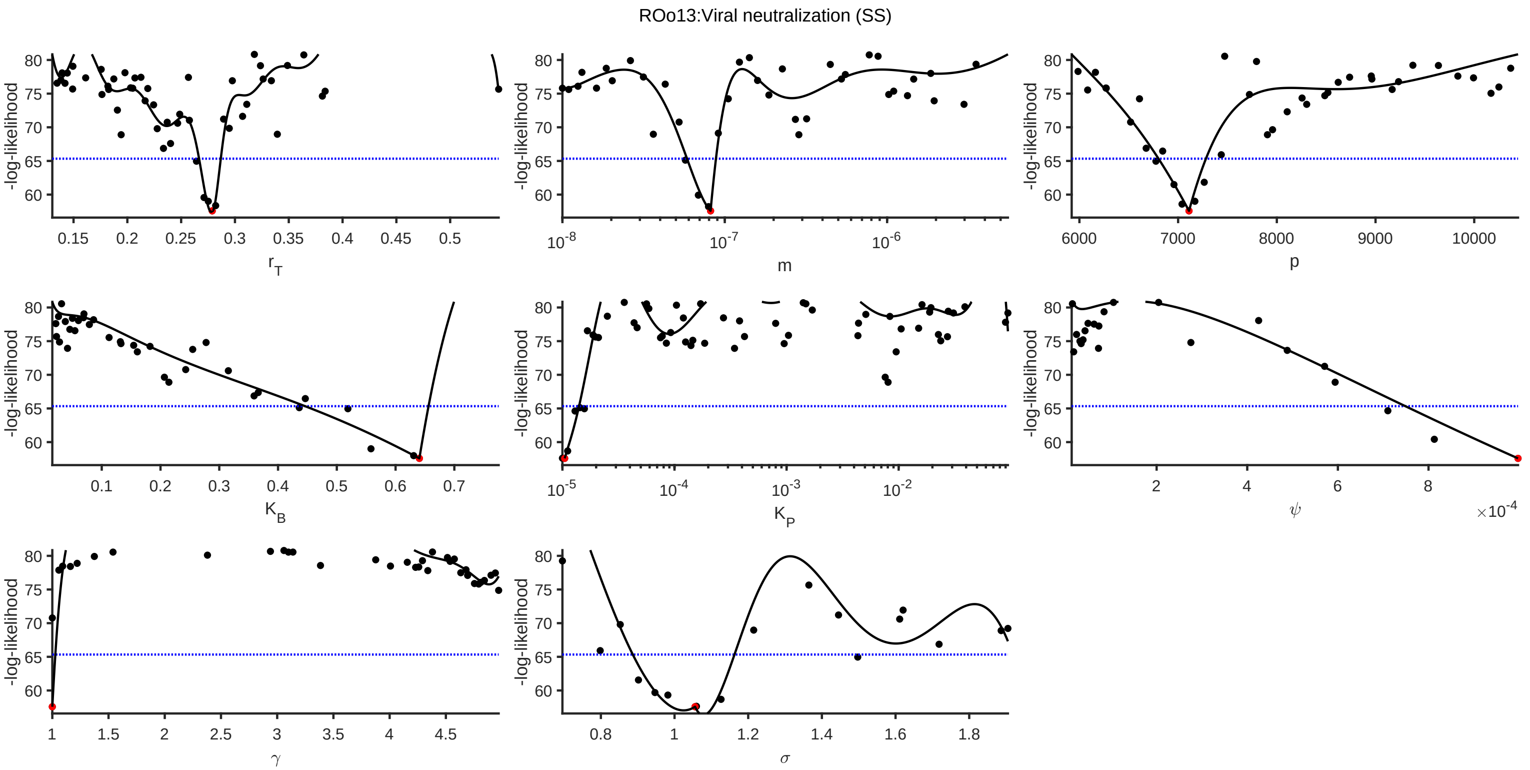
ROo13:Viral neutralization (SS)
-log-likelihood
r_T
m
p
K_B
K_P
ψ
×10^-4
γ
σ

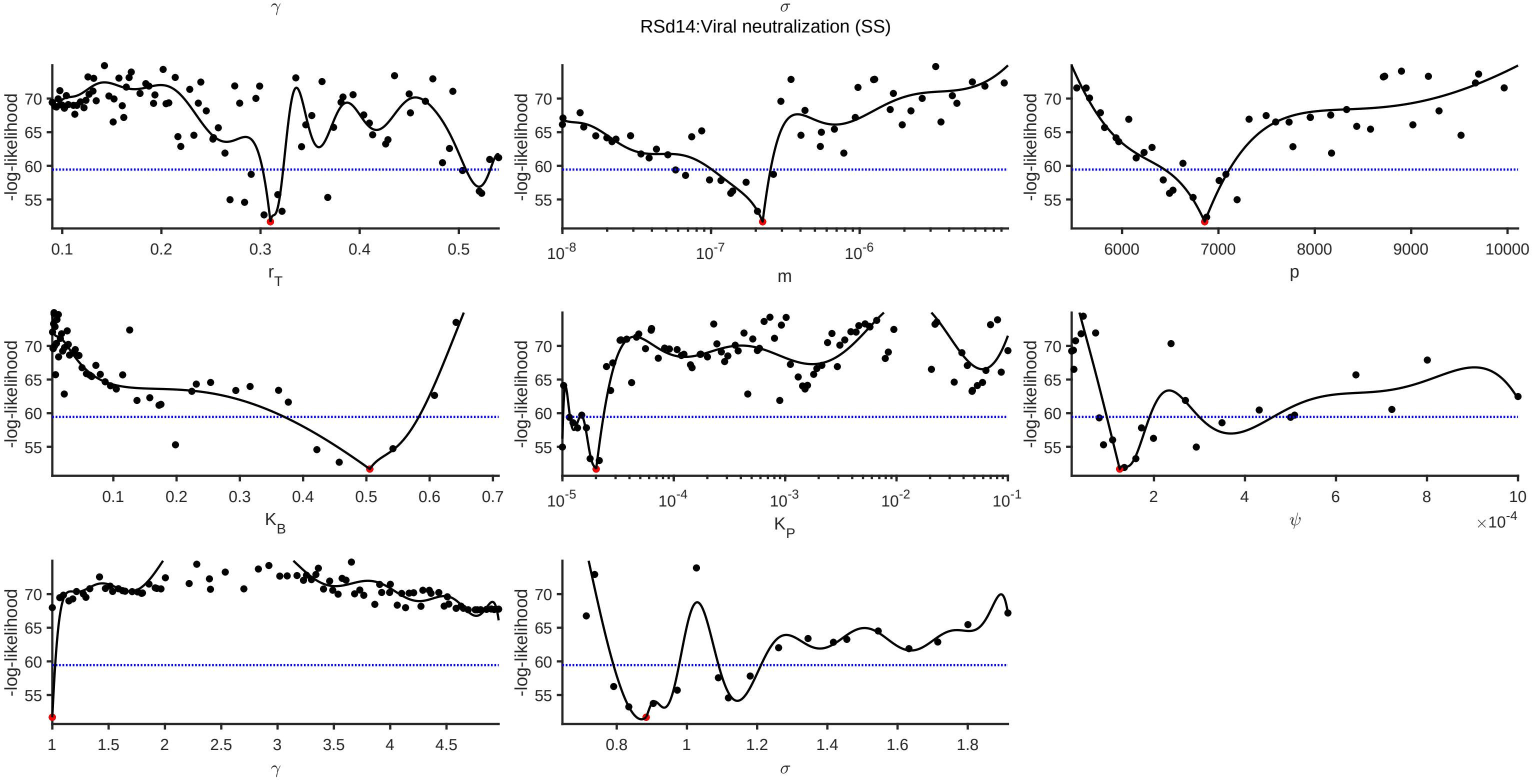
RSd14:Viral neutralization (SS)
-log-likelihood
r_T
m
p
K_B
K_P
ψ
×10^-4
γ
σ

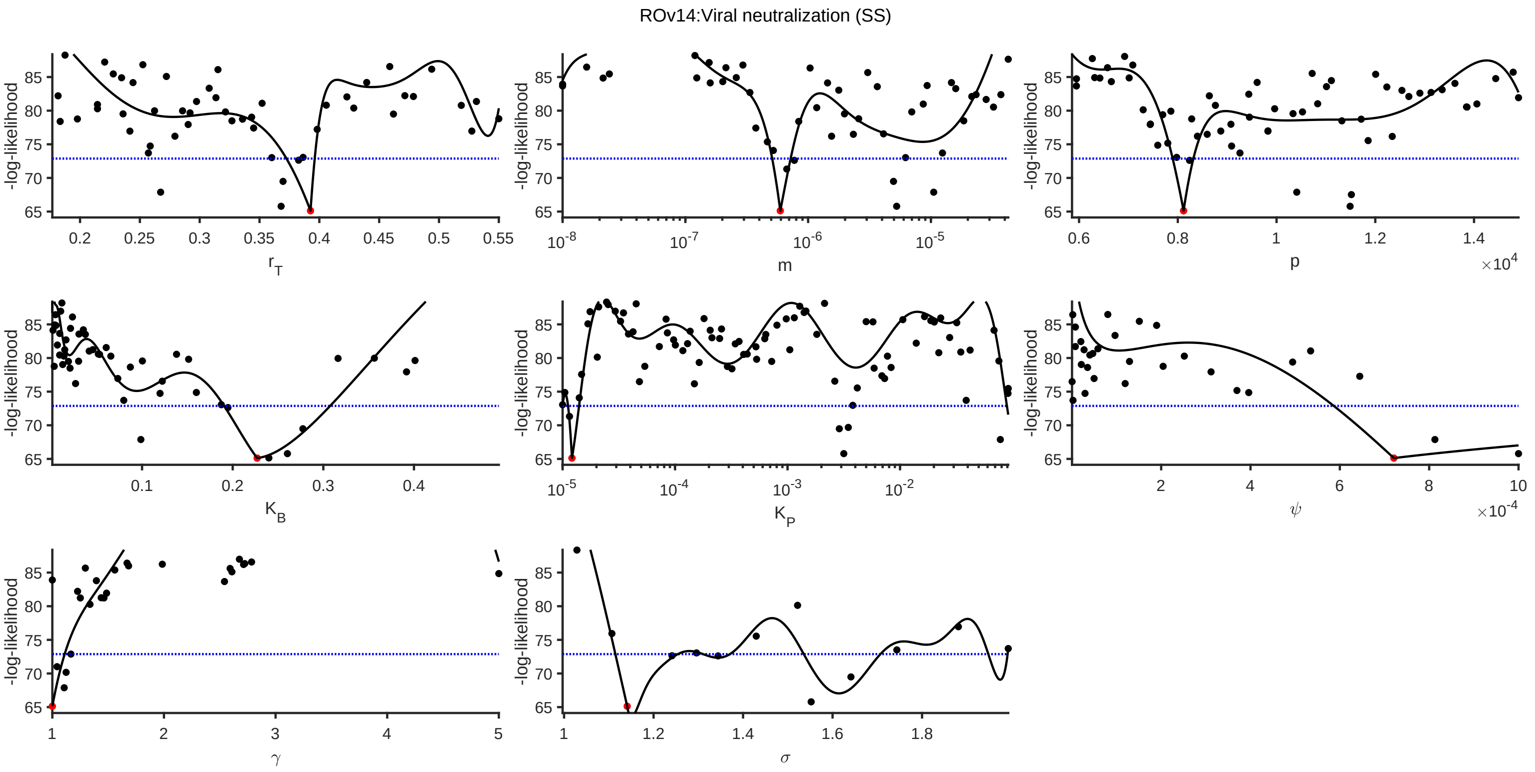
ROv14:Viral neutralization (SS)
-log-likelihood
r_T
m
p
×10^4
K_B
K_P
ψ
×10^-4
γ
σ

5.3 Antigen presenting cell effector cell source model

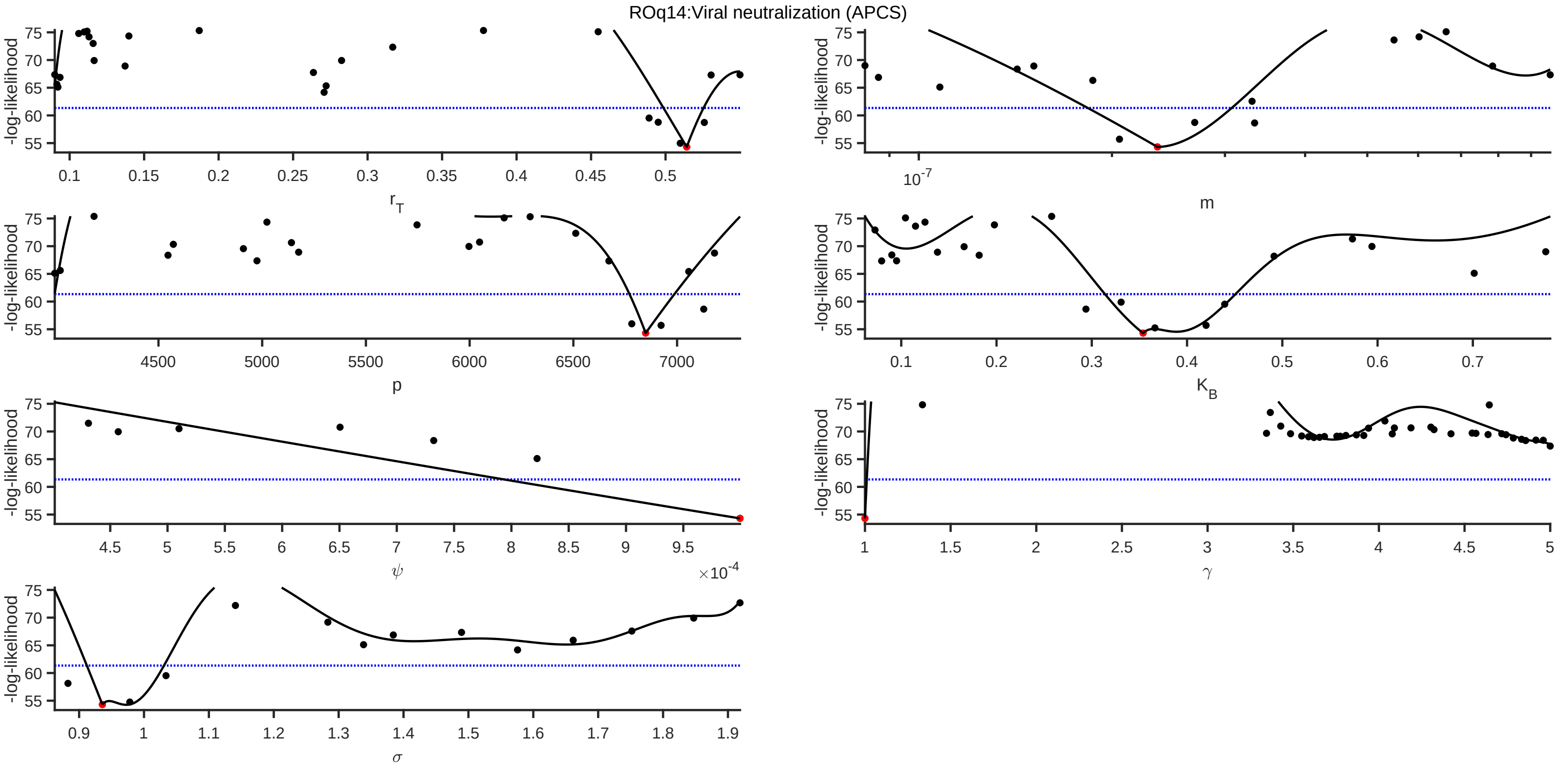

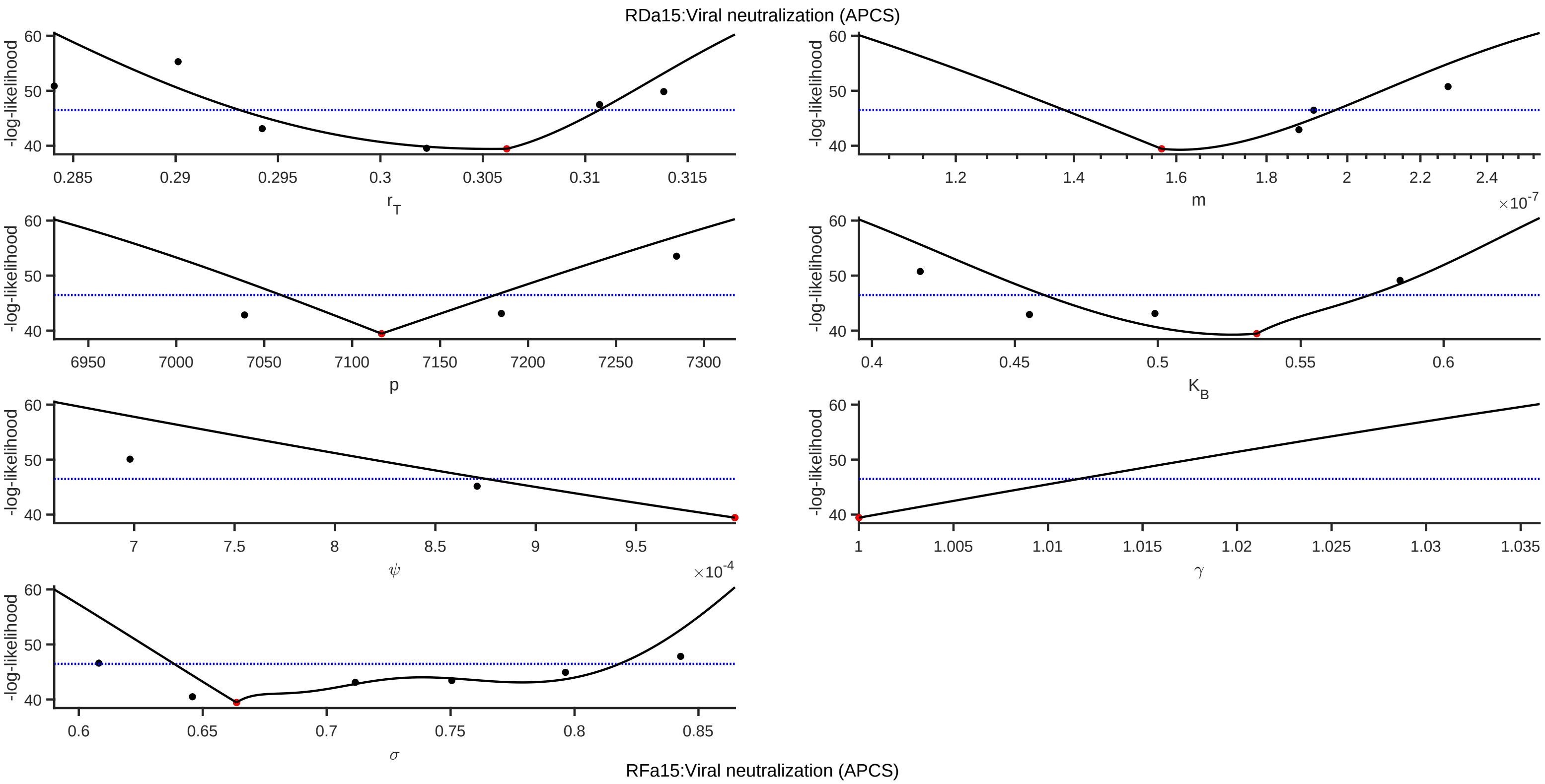
RDa15:Viral neutralization (APCS)
-log-likelihood
r_T
m
$\times 10^{-7}$
p
K_B
ψ
$\times 10^{-4}$
γ
σ

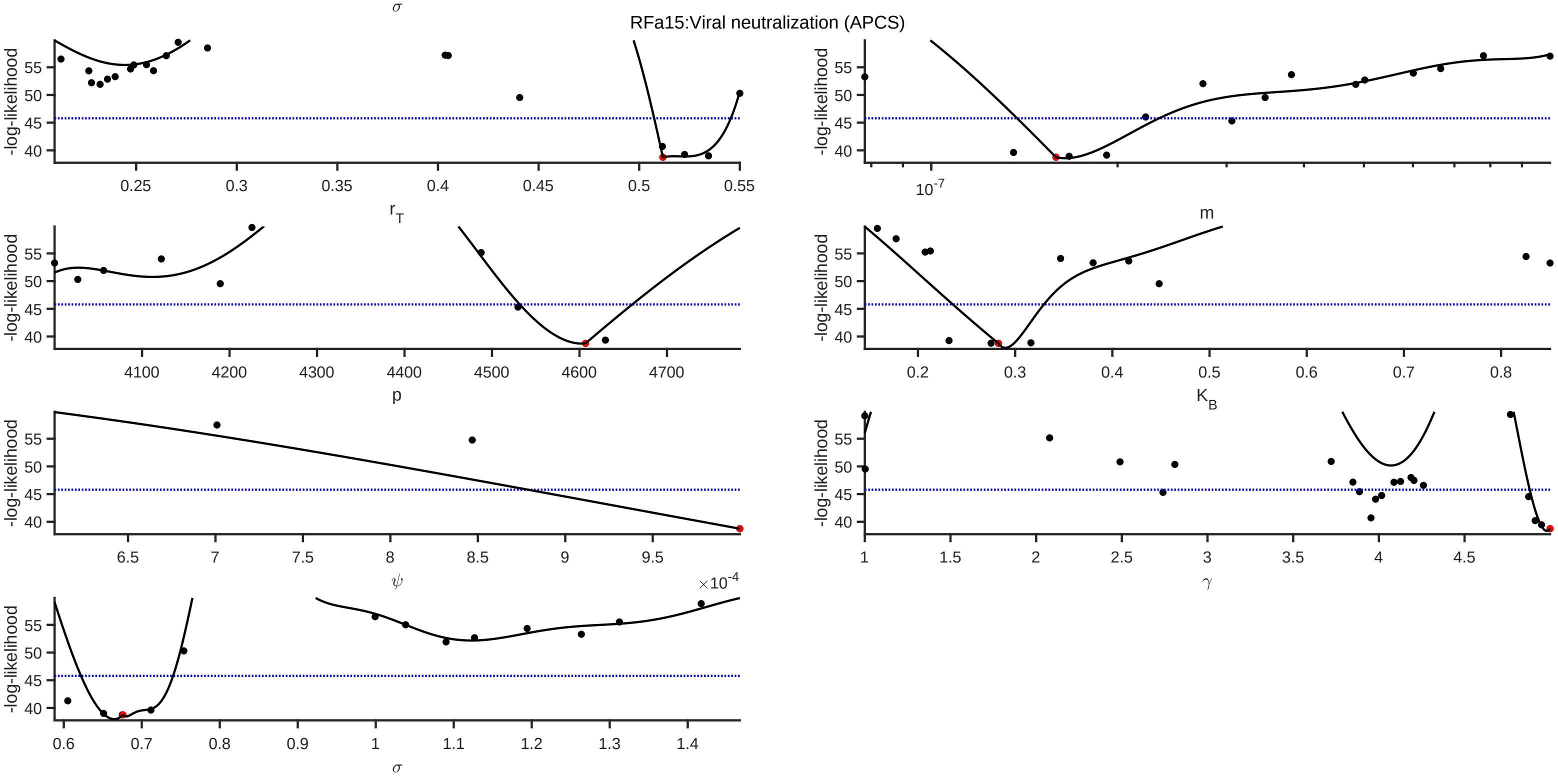
RFa15:Viral neutralization (APCS)
-log-likelihood
r_T
m
10^{-7}
p
K_B
ψ
$\times 10^{-4}$
γ
σ

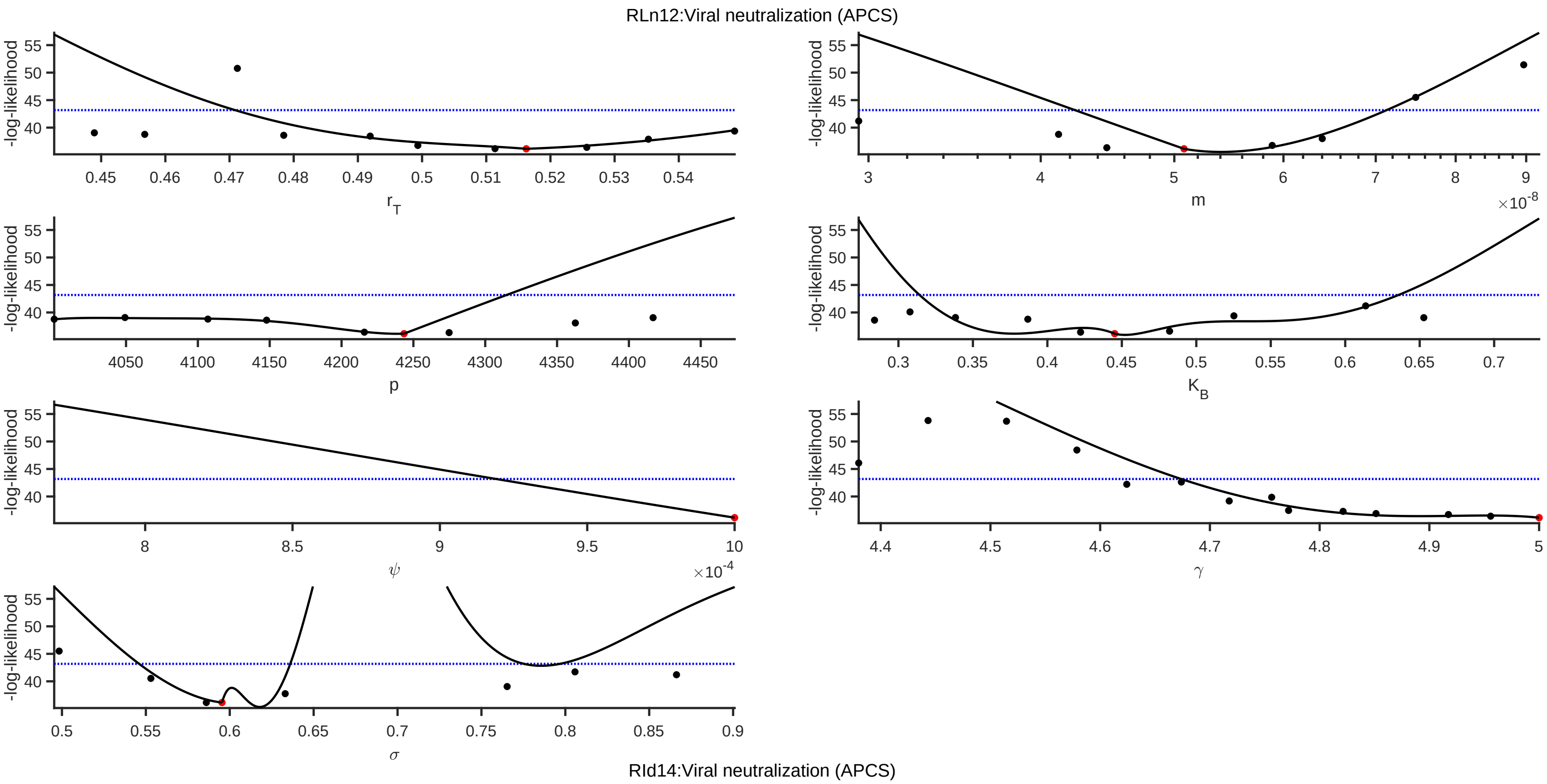
RLn12:Viral neutralization (APCS)
-log-likelihood
r_T
m
×10^-8
p
K_B
ψ
×10^-4
γ
σ

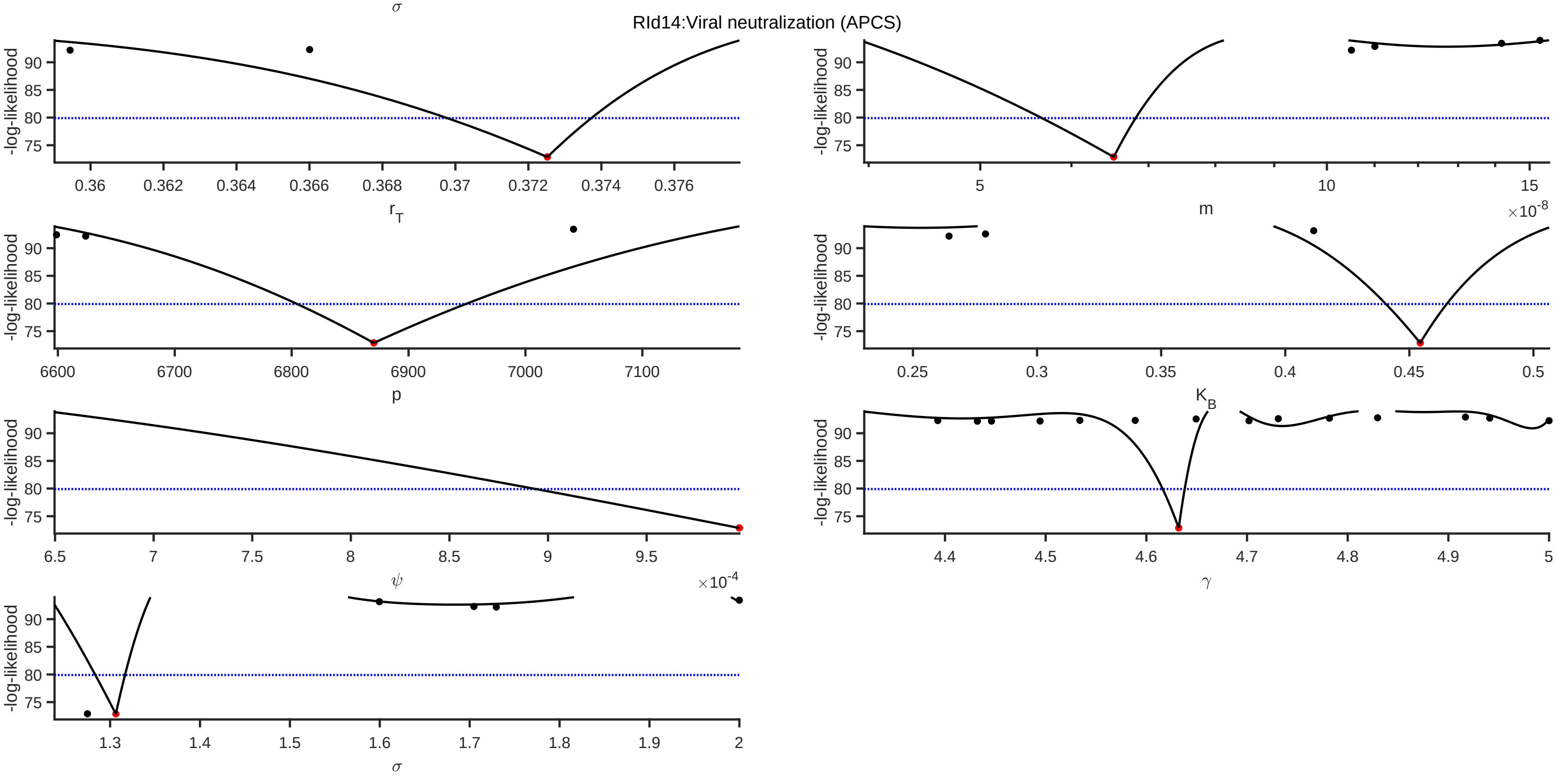
RId14:Viral neutralization (APCS)
-log-likelihood
r_T
m
×10^-8
p
K_B
ψ
×10^-4
γ
σ

ROo13:Viral neutralization (APCS)

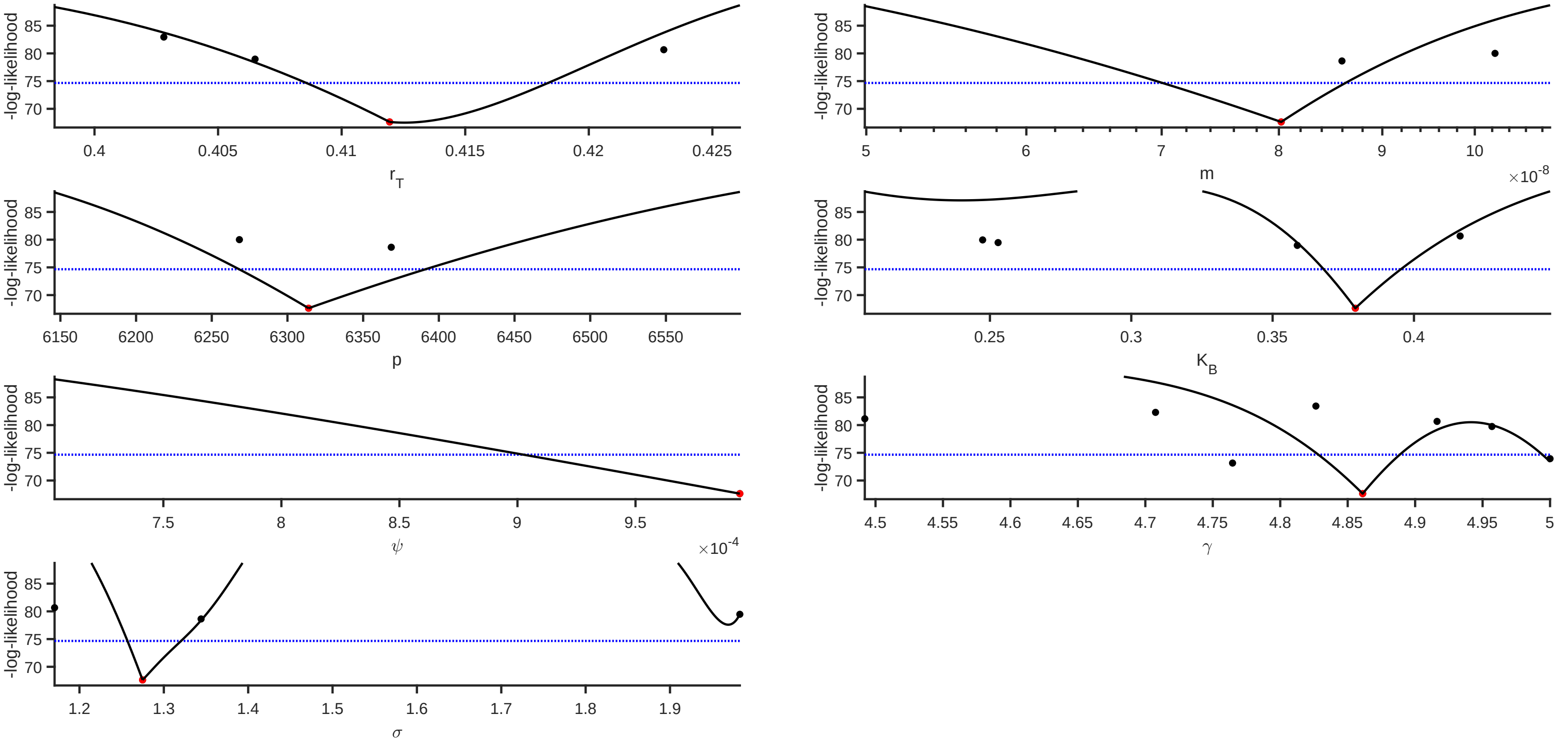

RSd14:Viral neutralization (APCS)

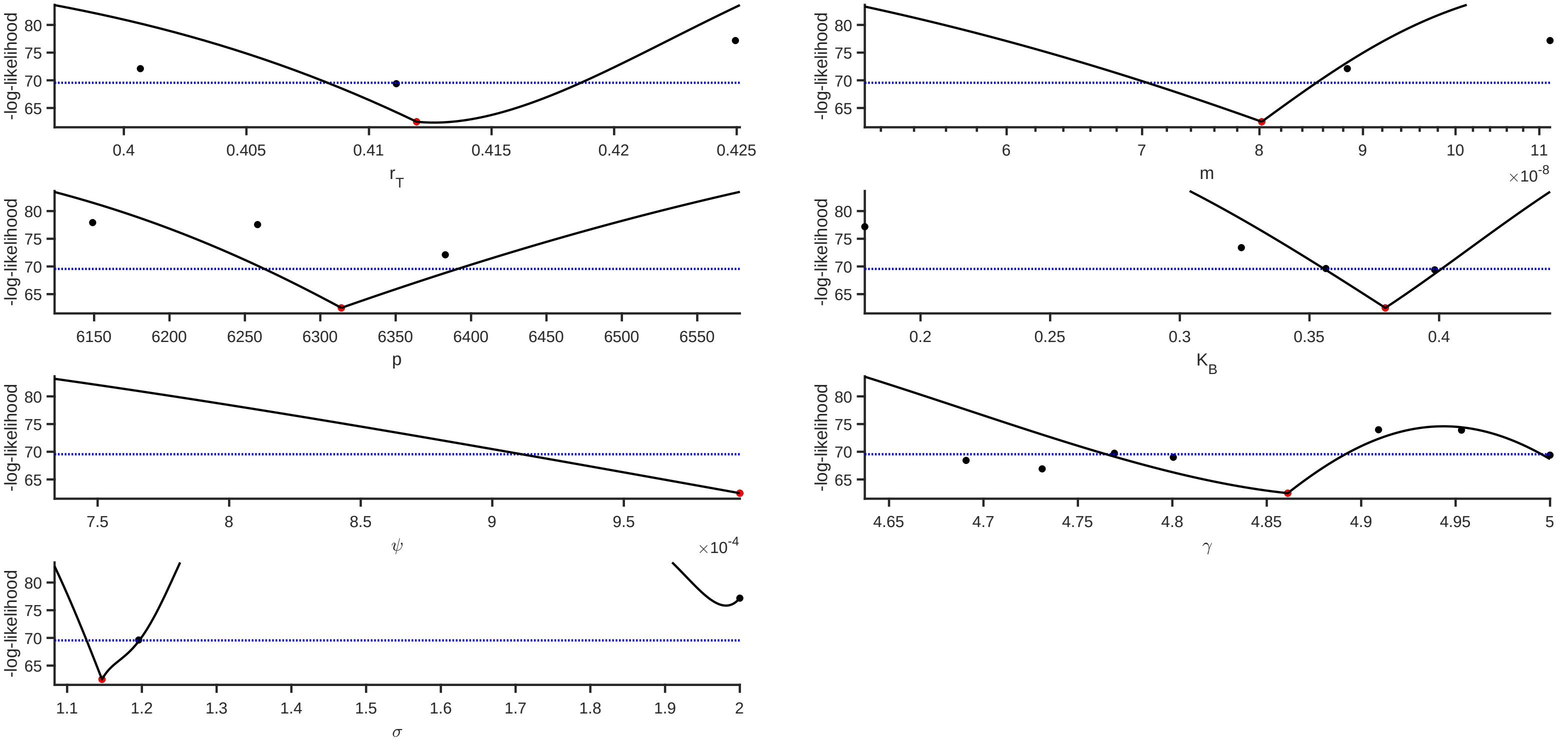

ROv14:Viral neutralization (APCS)

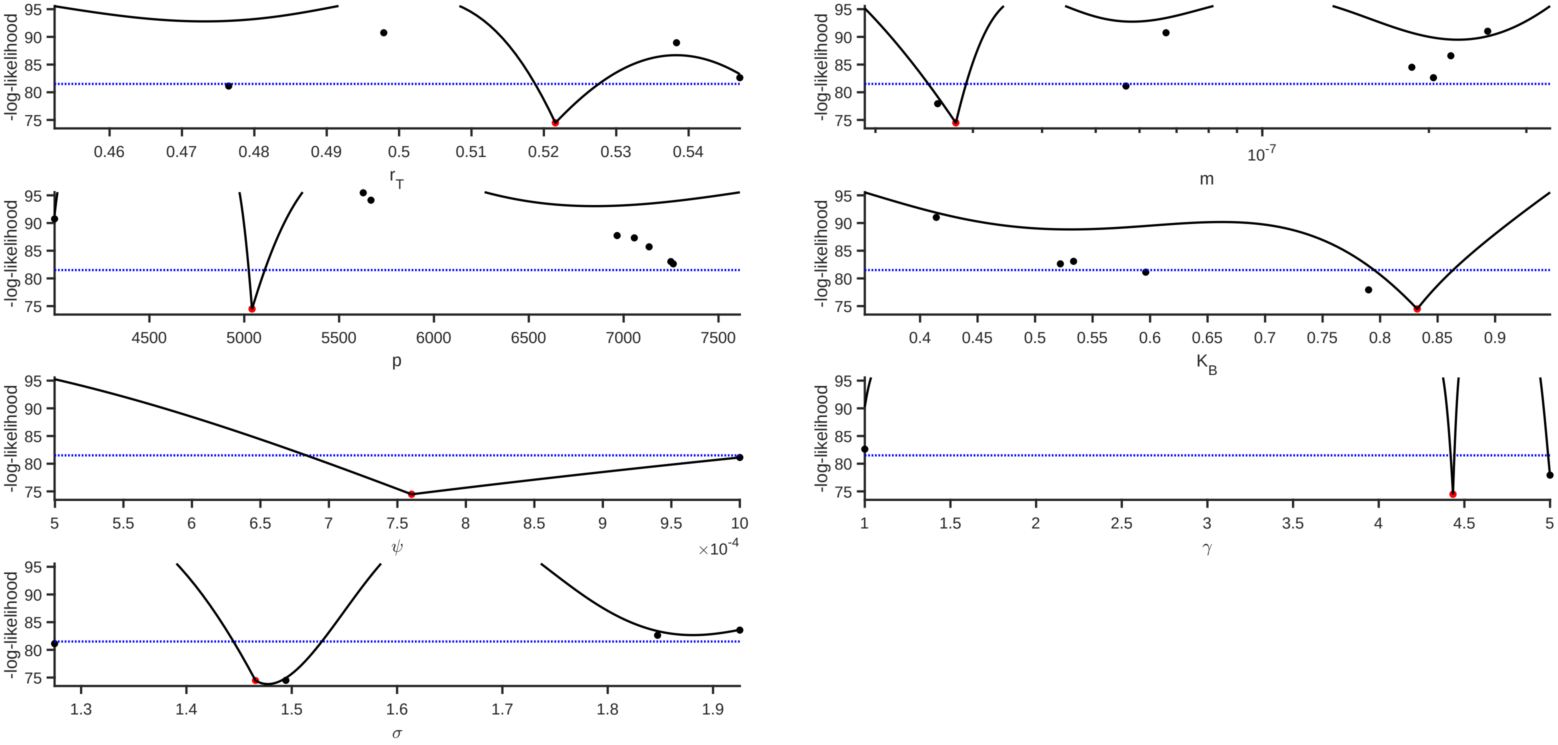

6 Sensitivity anlaysis: Viral neutralization

6.1 Baseline effector cell source model

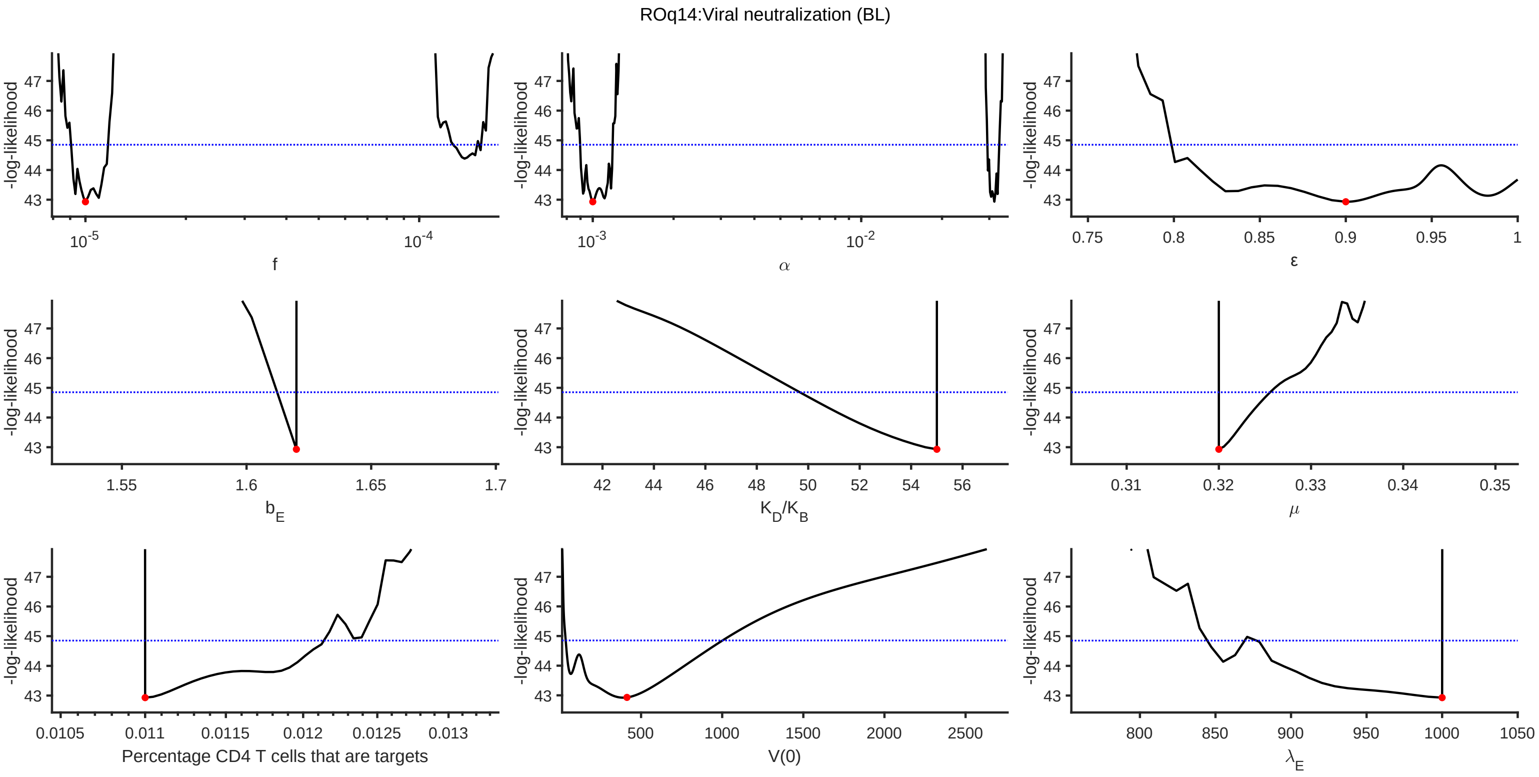

RDa15:Viral neutralization (BL)

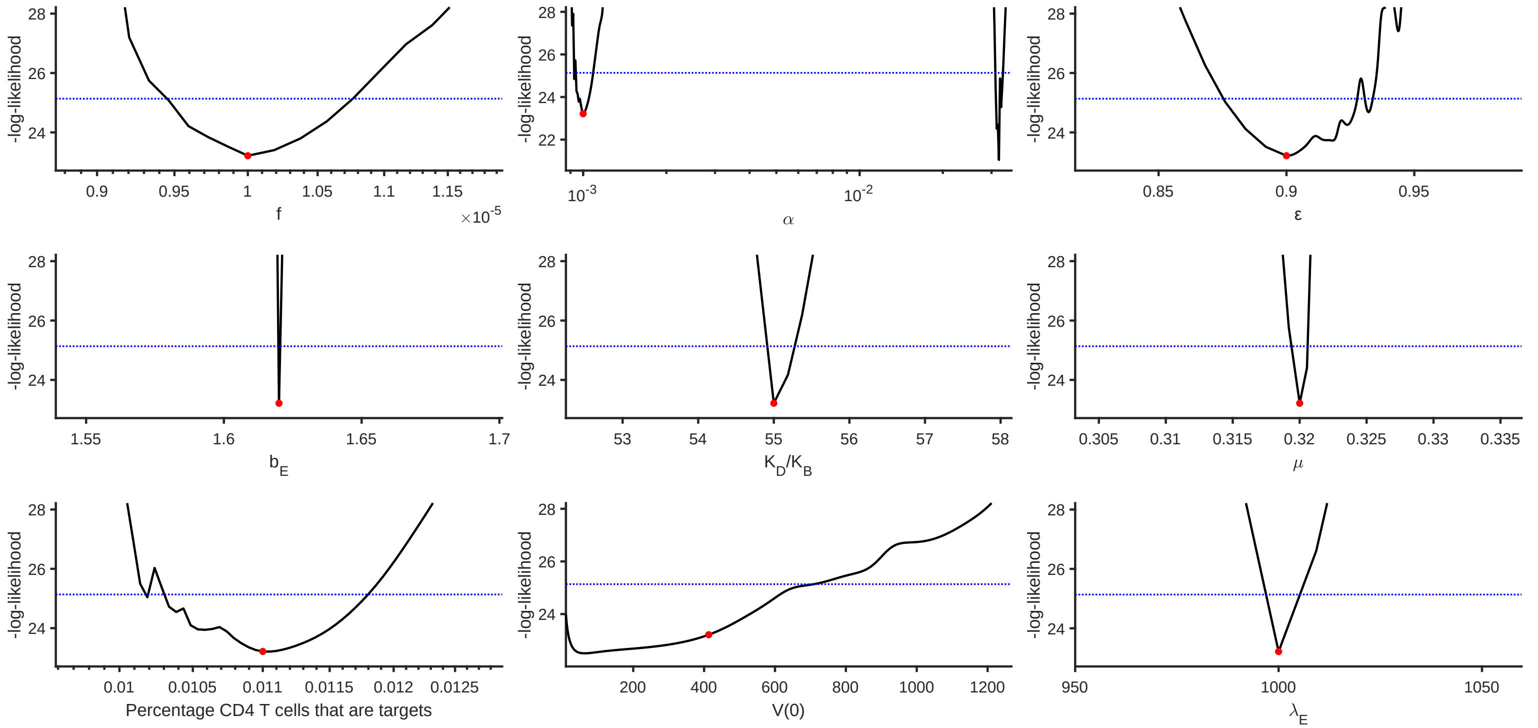

RFa15:Viral neutralization (BL)

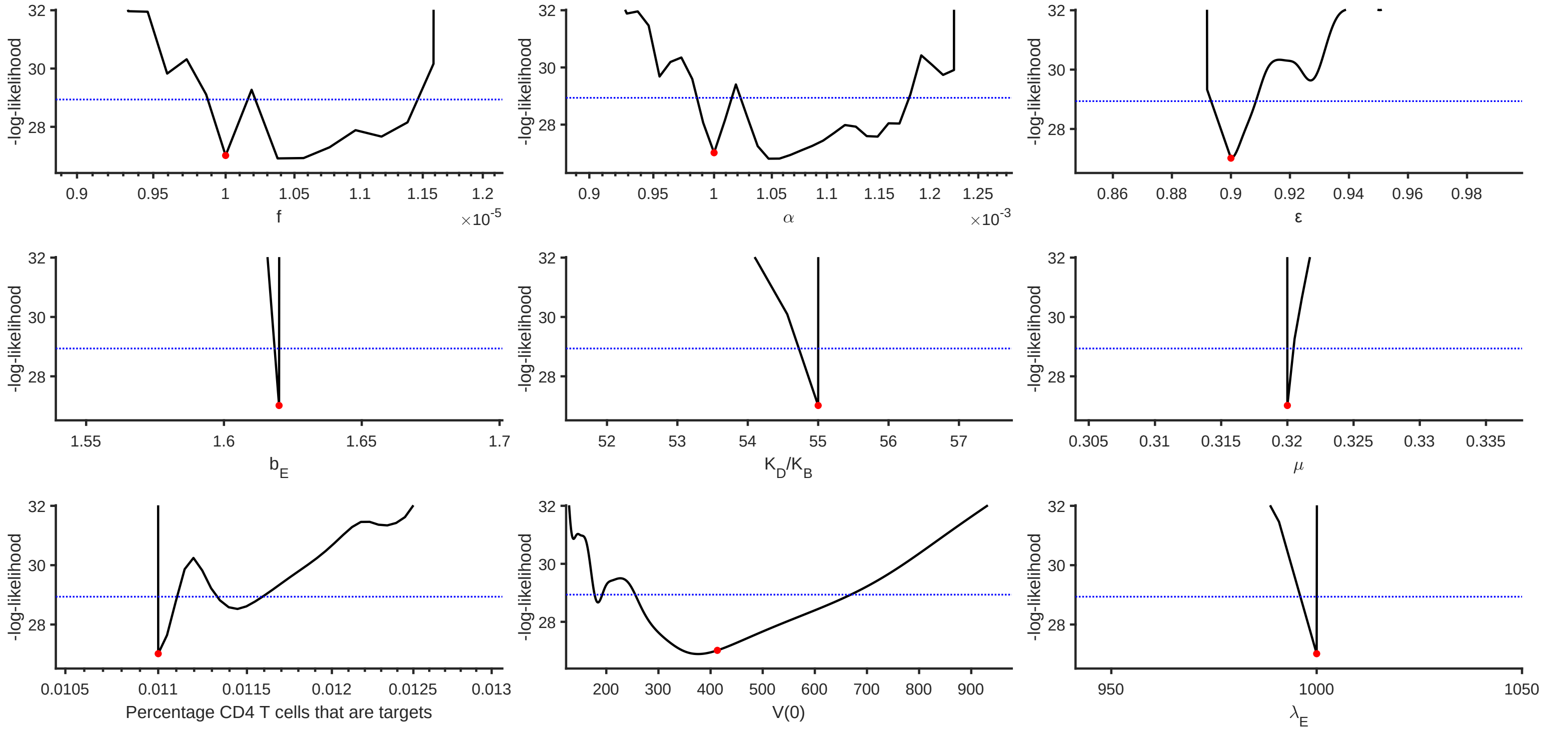

RLn12:Viral neutralization (BL)

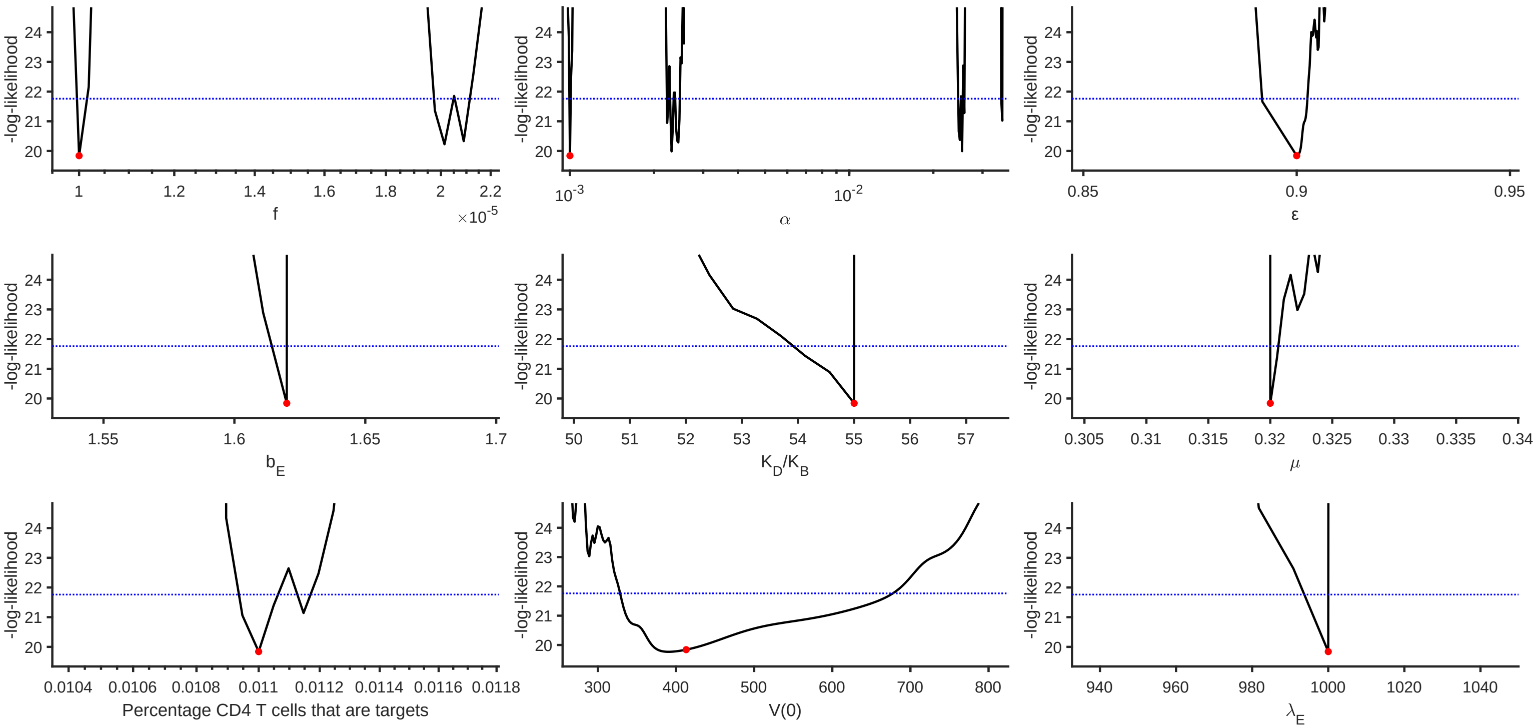

RId14:Viral neutralization (BL)

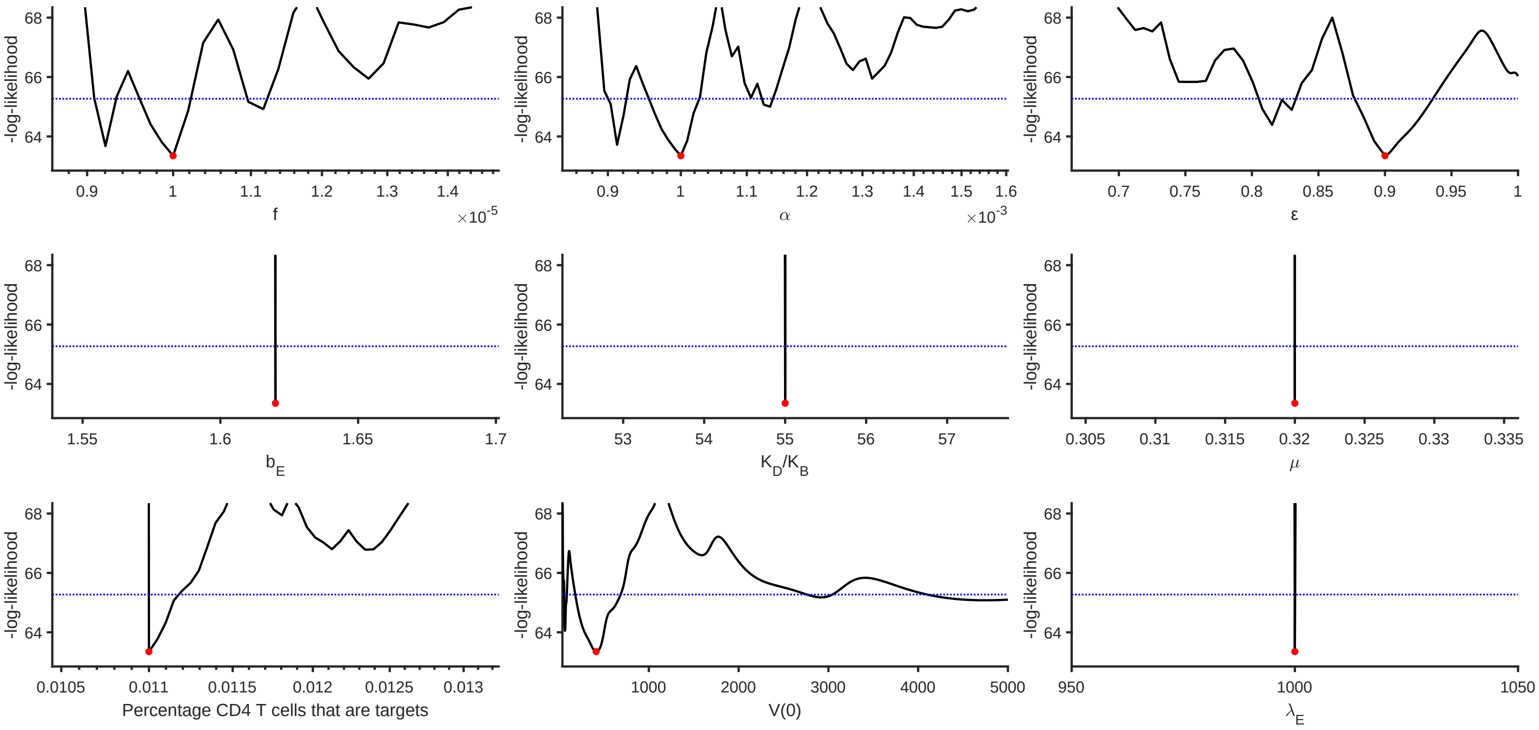

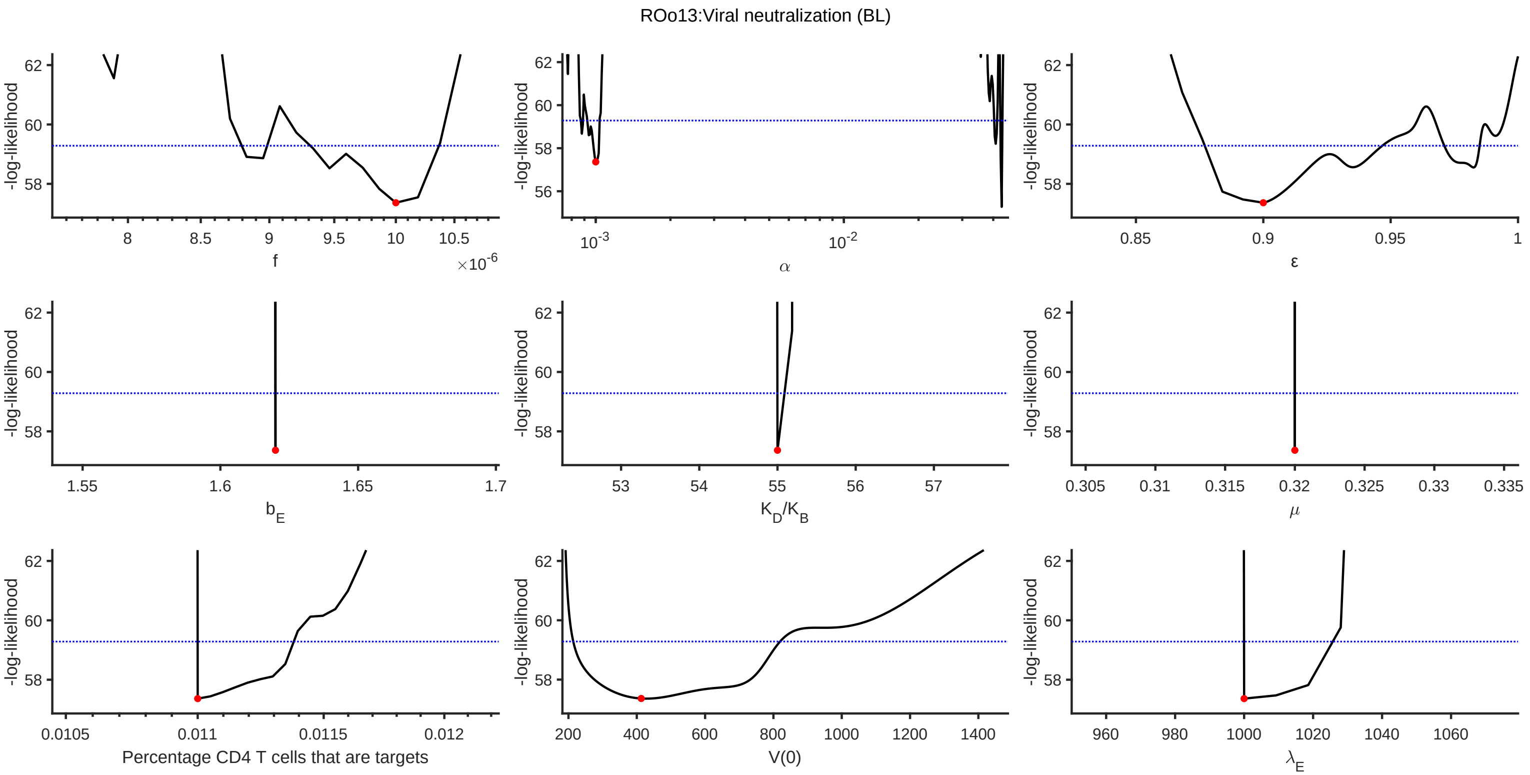
ROo13:Viral neutralization (BL)
-log-likelihood
f
×10^-6
α
ε
b_E
K_D/K_B
μ
Percentage CD4 T cells that are targets
V(0)
λ_E

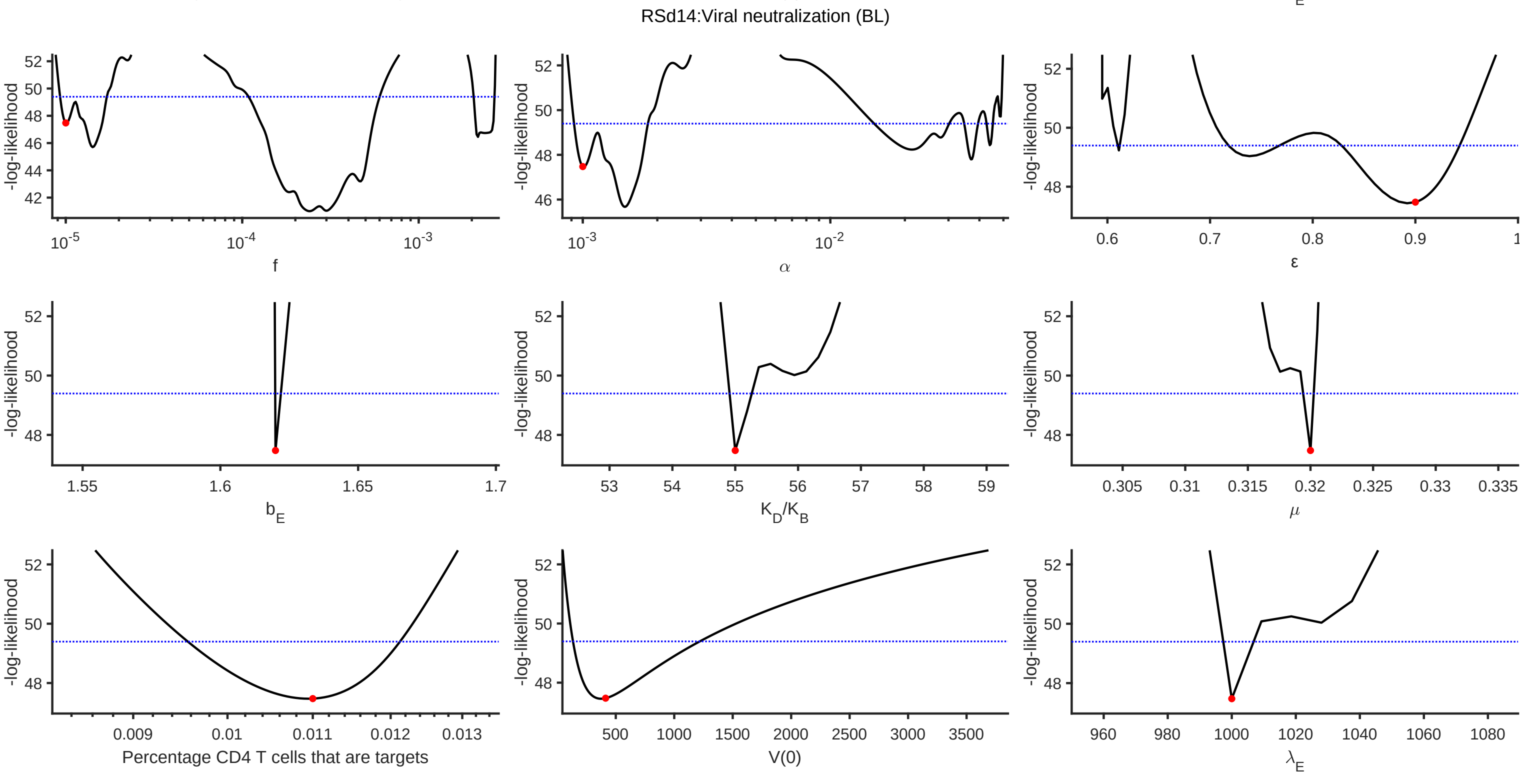
RSd14:Viral neutralization (BL)
-log-likelihood
f
α
ε
b_E
K_D/K_B
μ
Percentage CD4 T cells that are targets
V(0)
λ_E

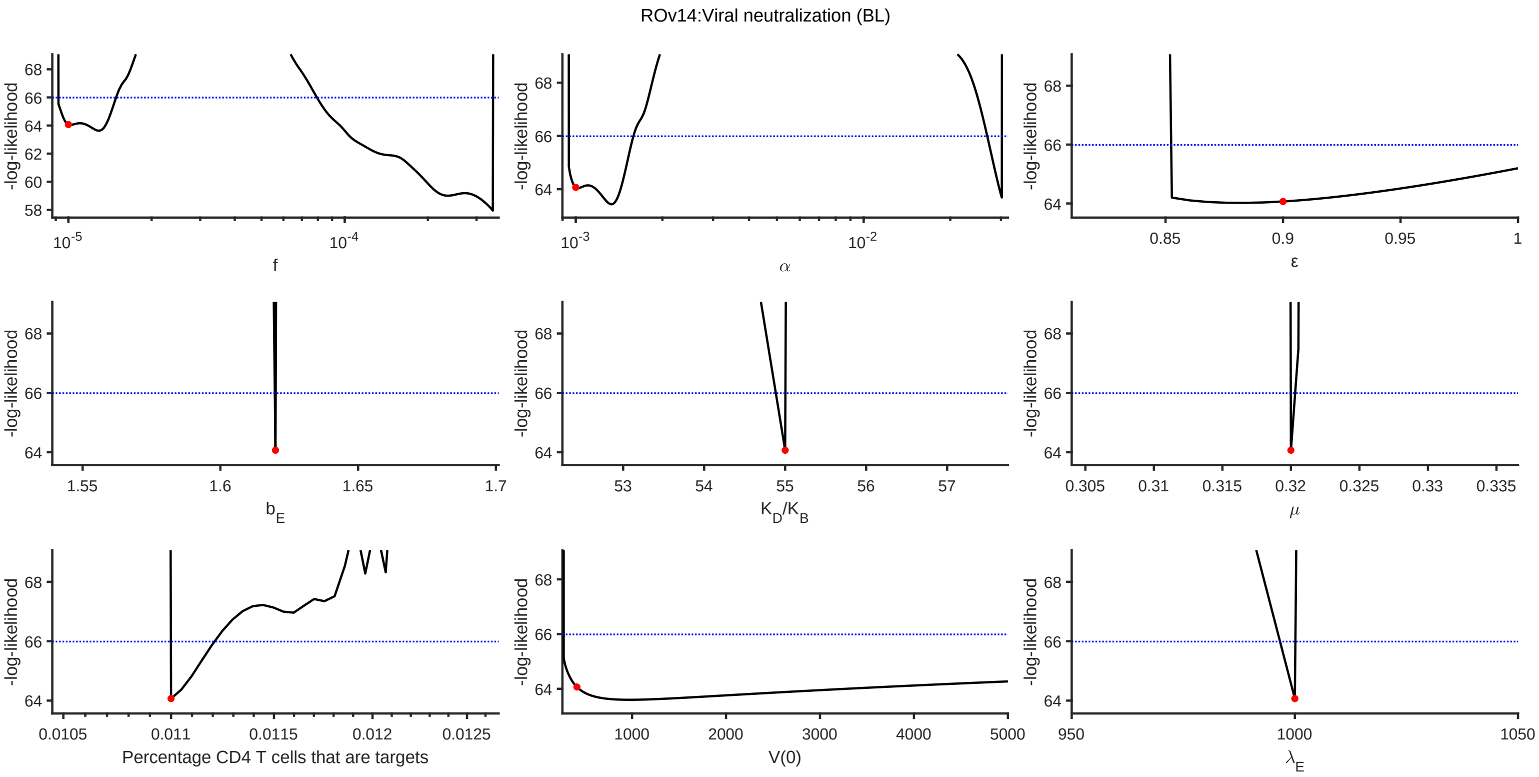
ROv14:Viral neutralization (BL)
-log-likelihood
f
α
ε
b_E
K_D/K_B
μ
Percentage CD4 T cells that are targets
V(0)
λ_E

6.2 Saturated effector cell source model

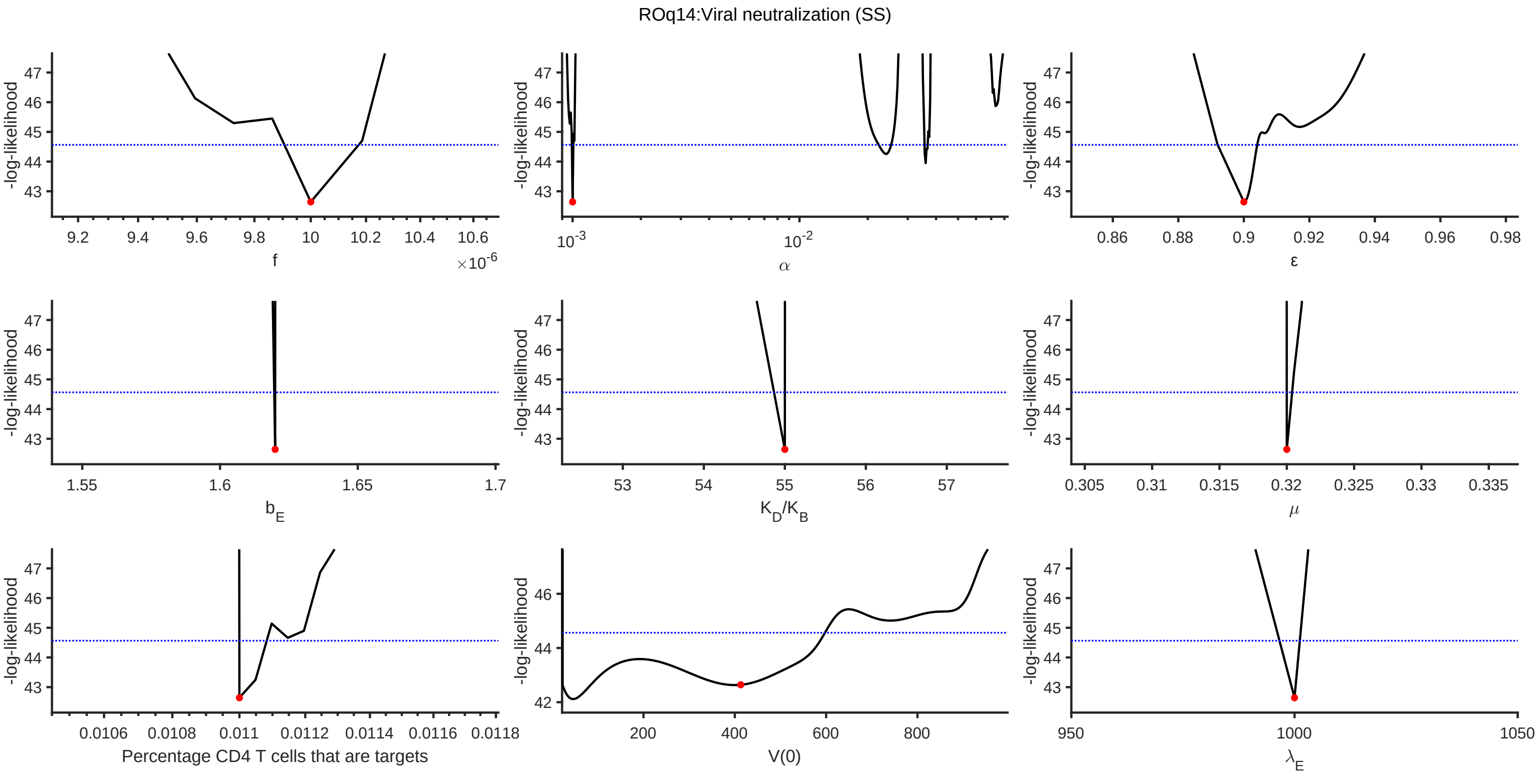

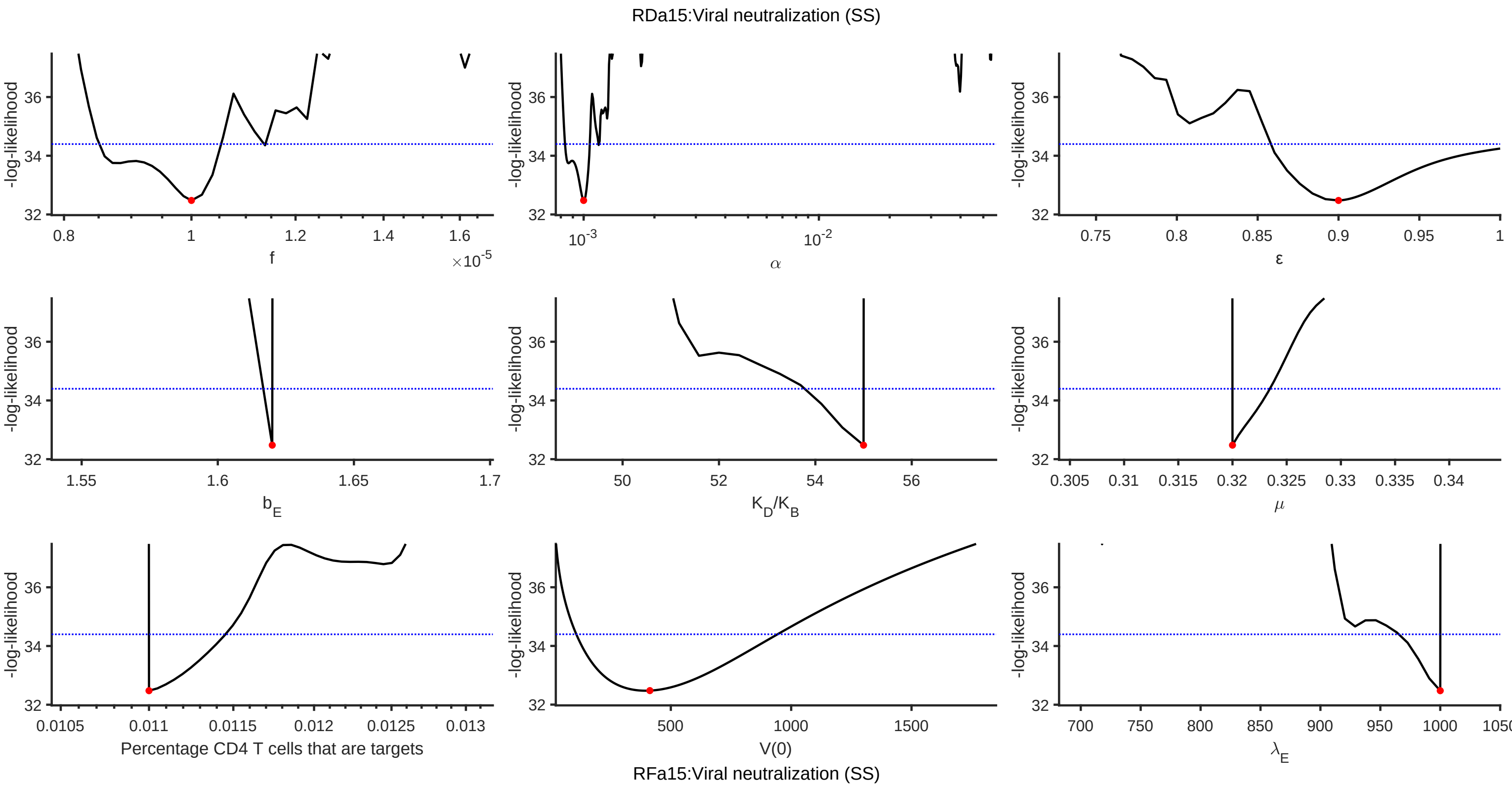
RDa15:Viral neutralization (SS)
-log-likelihood
f
×10^-5
α
ε
b_E
K_D/K_B
μ
Percentage CD4 T cells that are targets
V(0)
λ_E

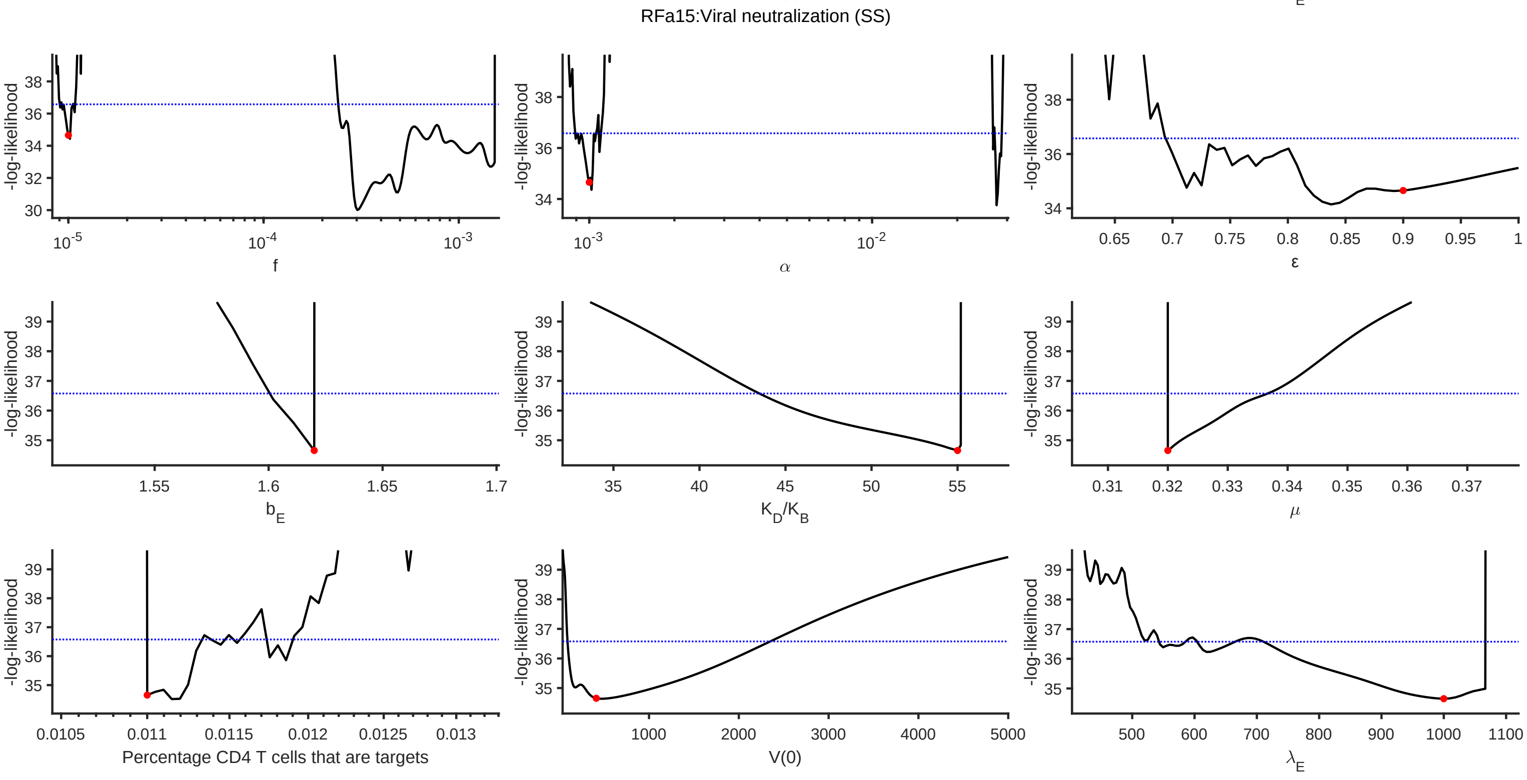
RFa15:Viral neutralization (SS)
-log-likelihood
f
α
ε
b_E
K_D/K_B
μ
Percentage CD4 T cells that are targets
V(0)
λ_E

RLn12:Viral neutralization (SS)

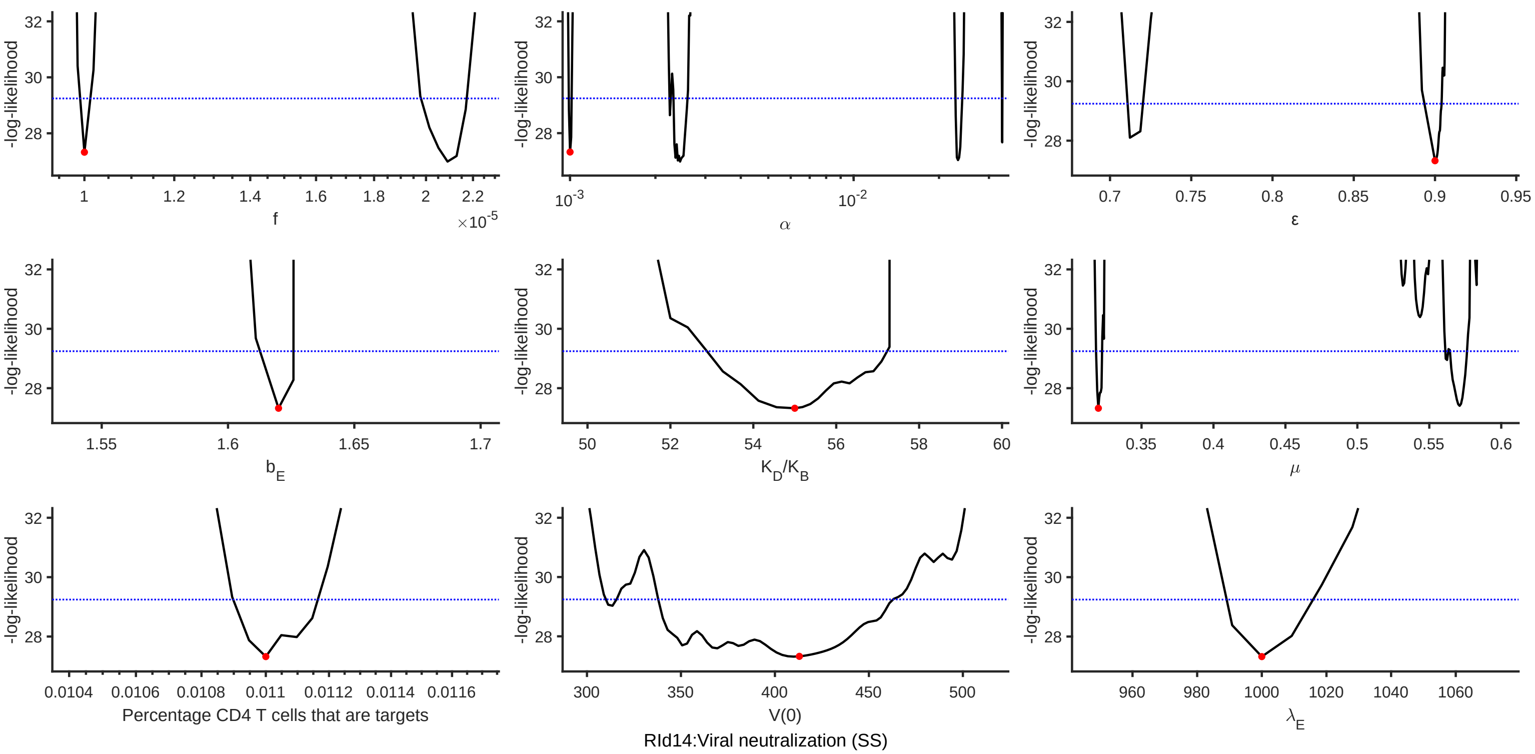

RId14:Viral neutralization (SS)

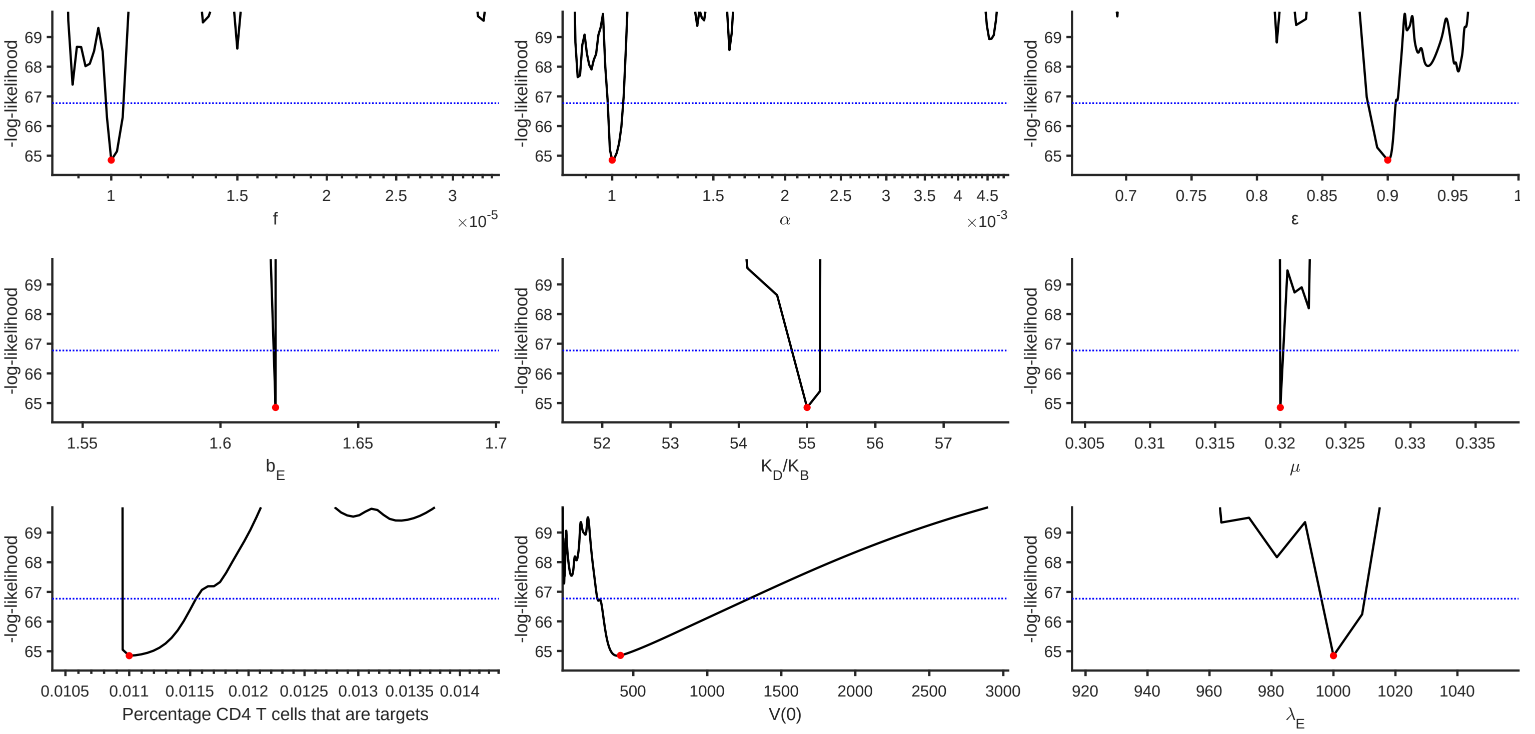

ROo13:Viral neutralization (SS)

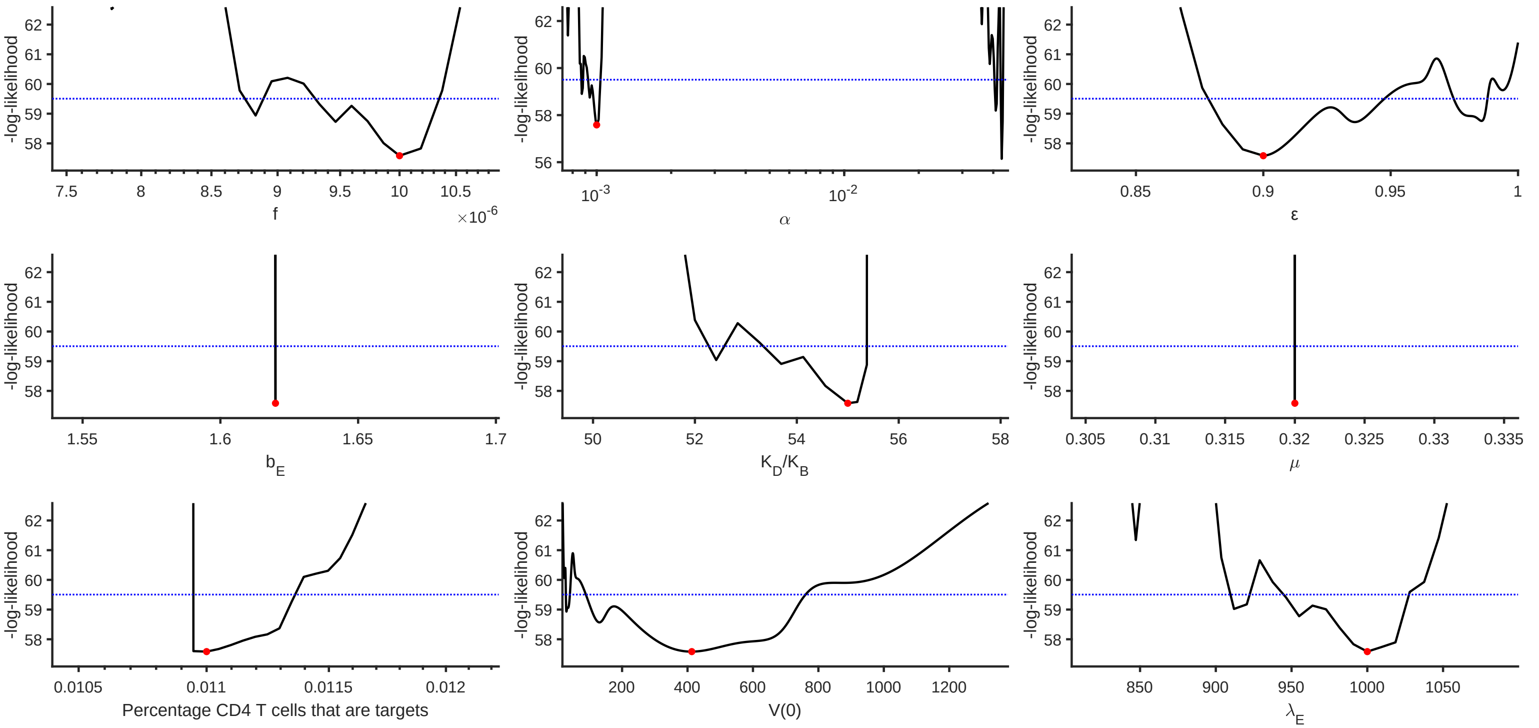

RSd14:Viral neutralization (SS)

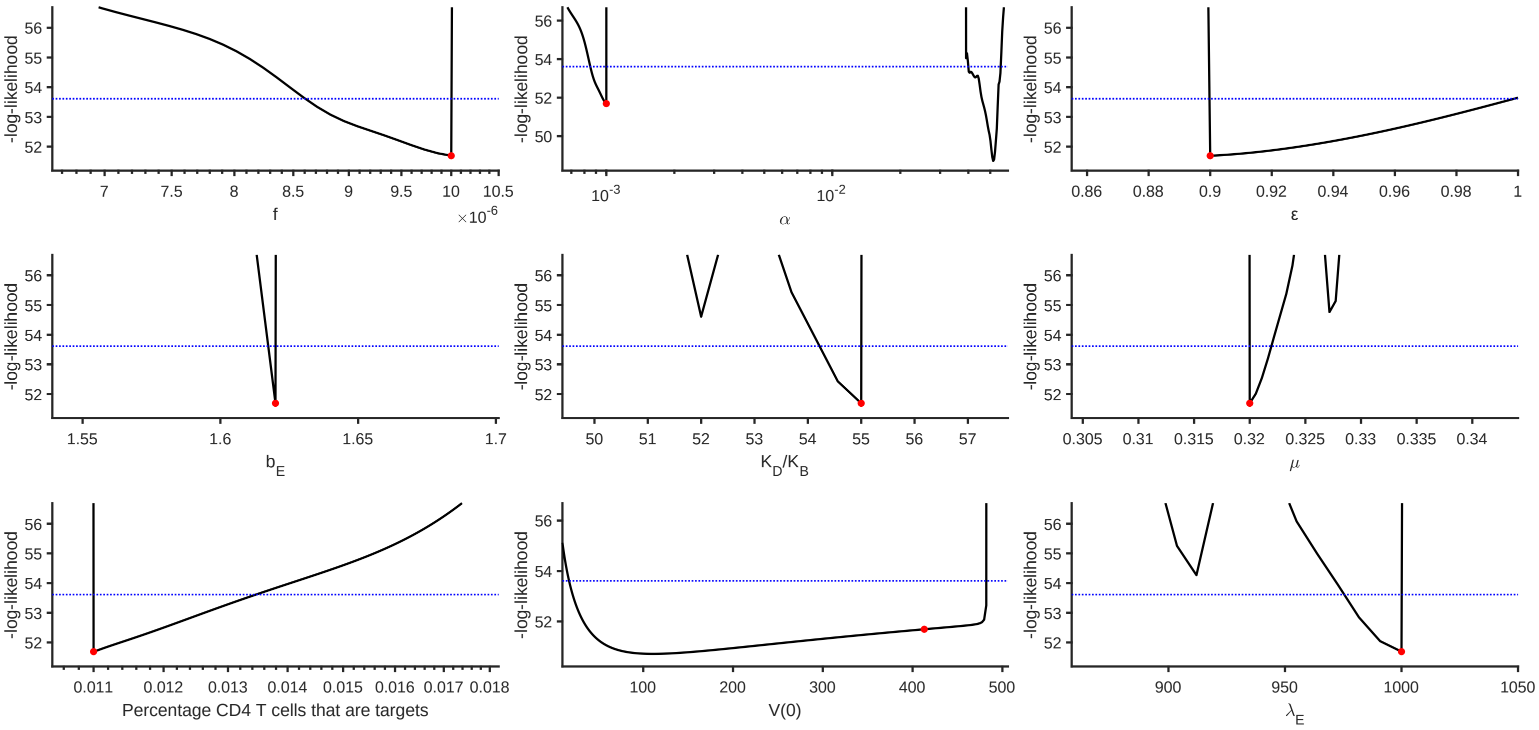

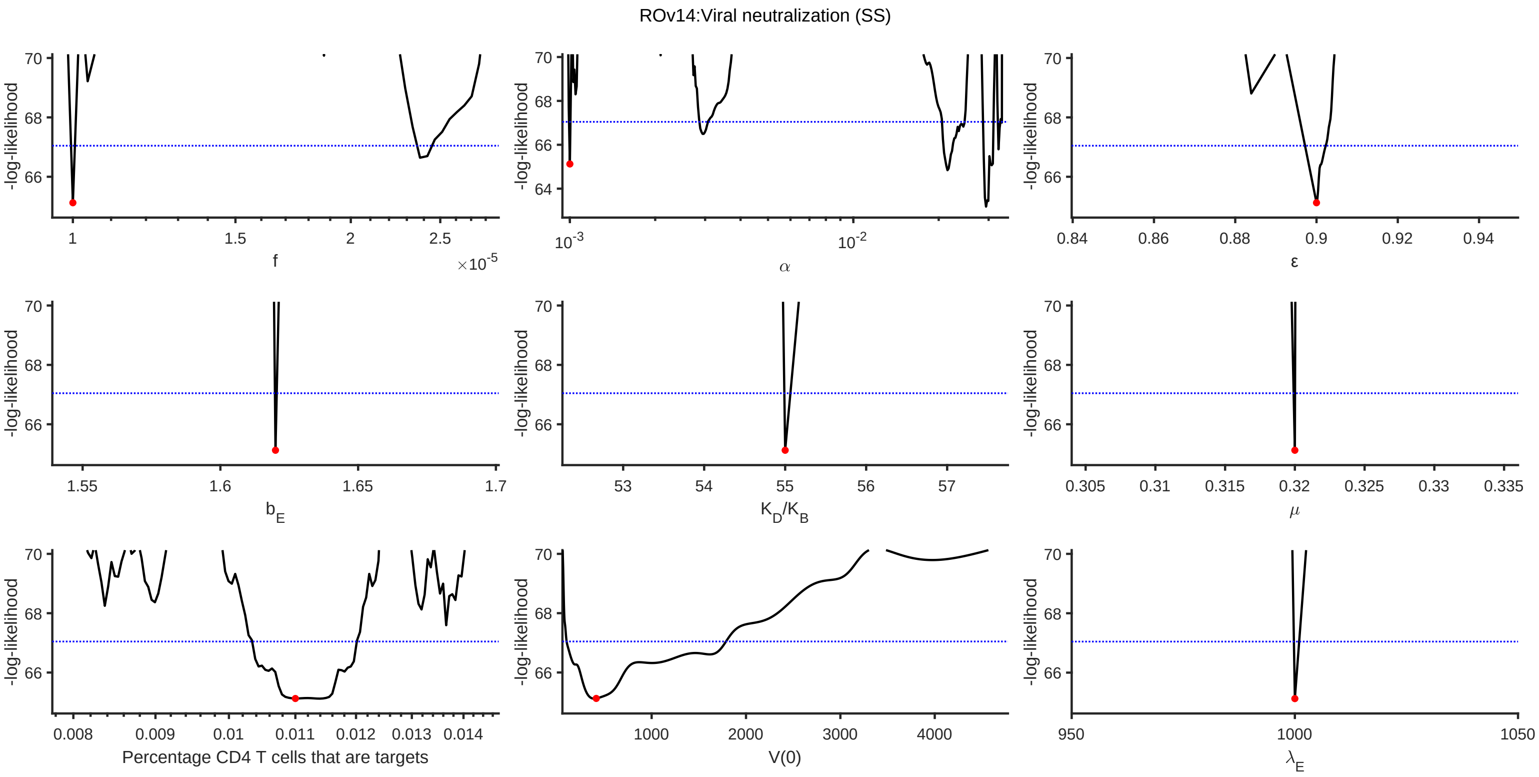
ROv14:Viral neutralization (SS)
-log-likelihood
f
×10^-5
α
ε
b_E
K_D/K_B
μ
Percentage CD4 T cells that are targets
V(0)
λ_E

6.3 Antigen presenting cell effector cell source model

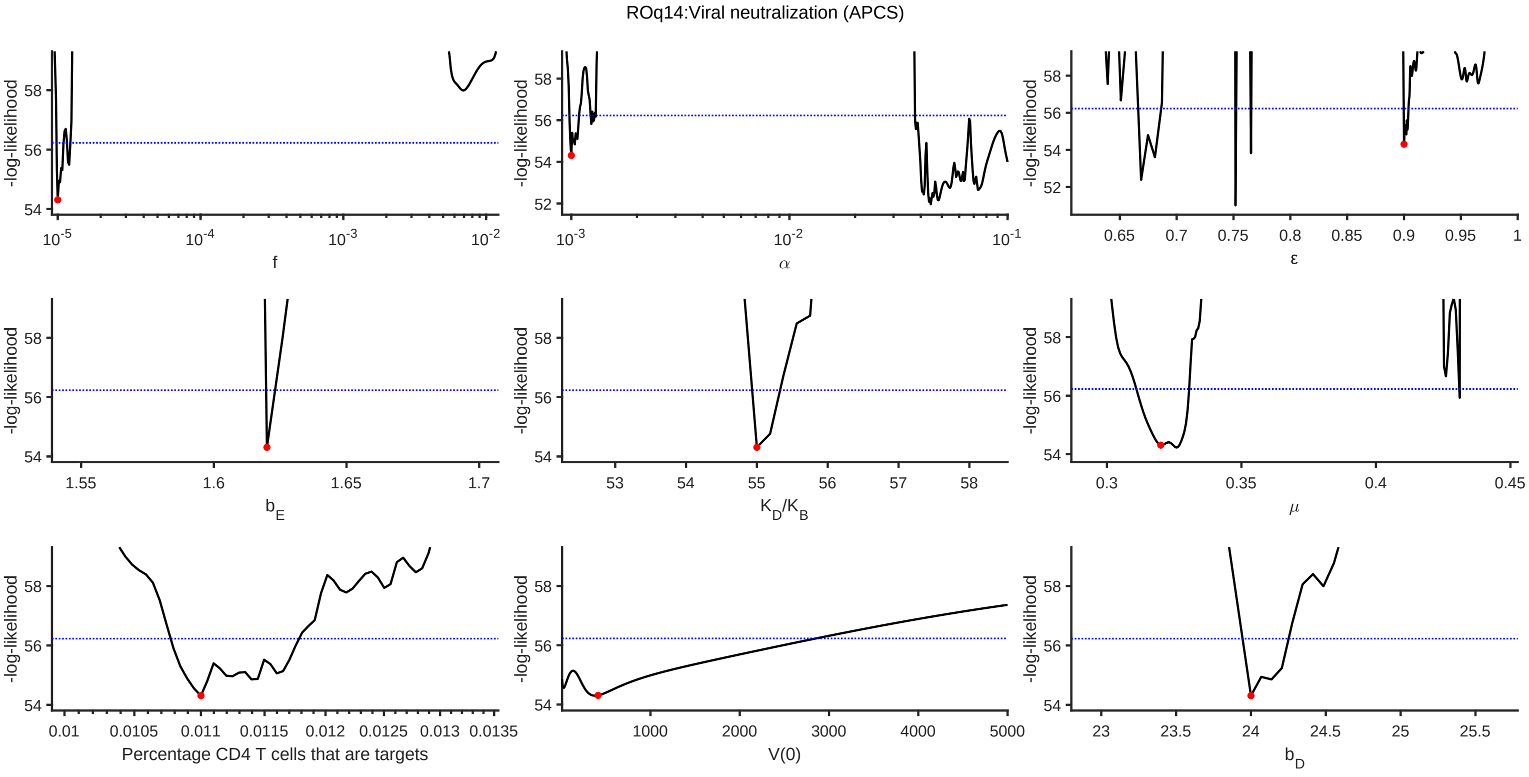

RDa15:Viral neutralization (APCS)

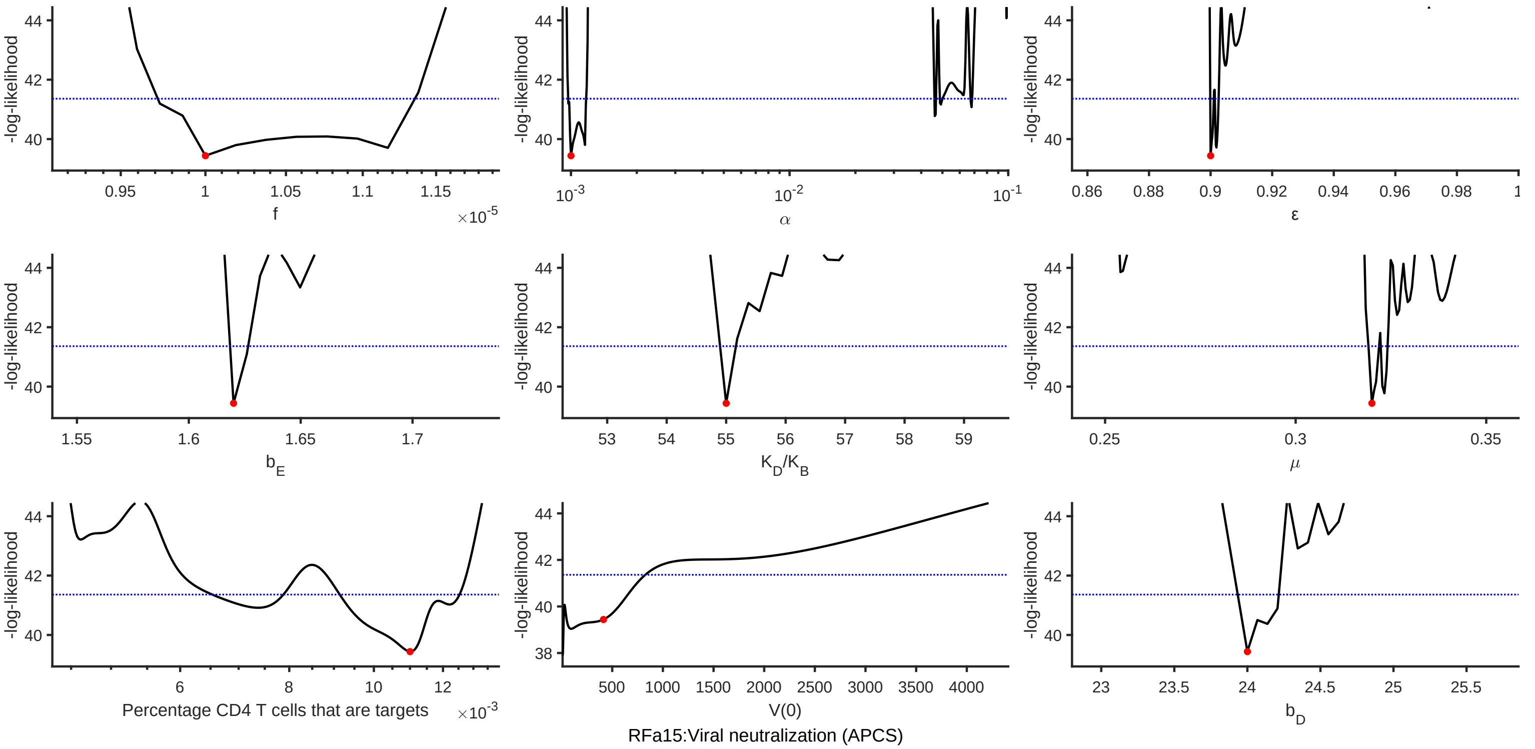

RFa15:Viral neutralization (APCS)

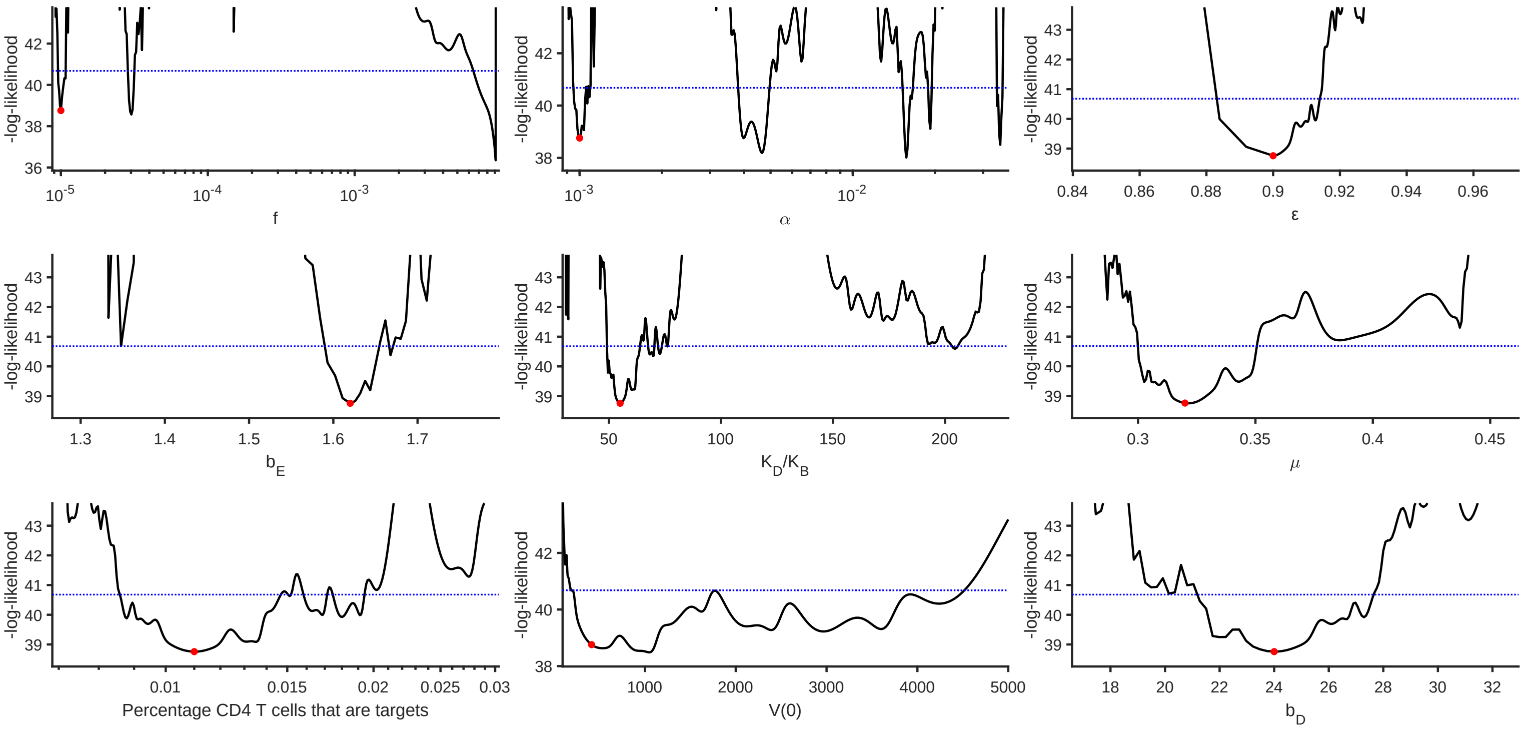

RLn12:Viral neutralization (APCS)

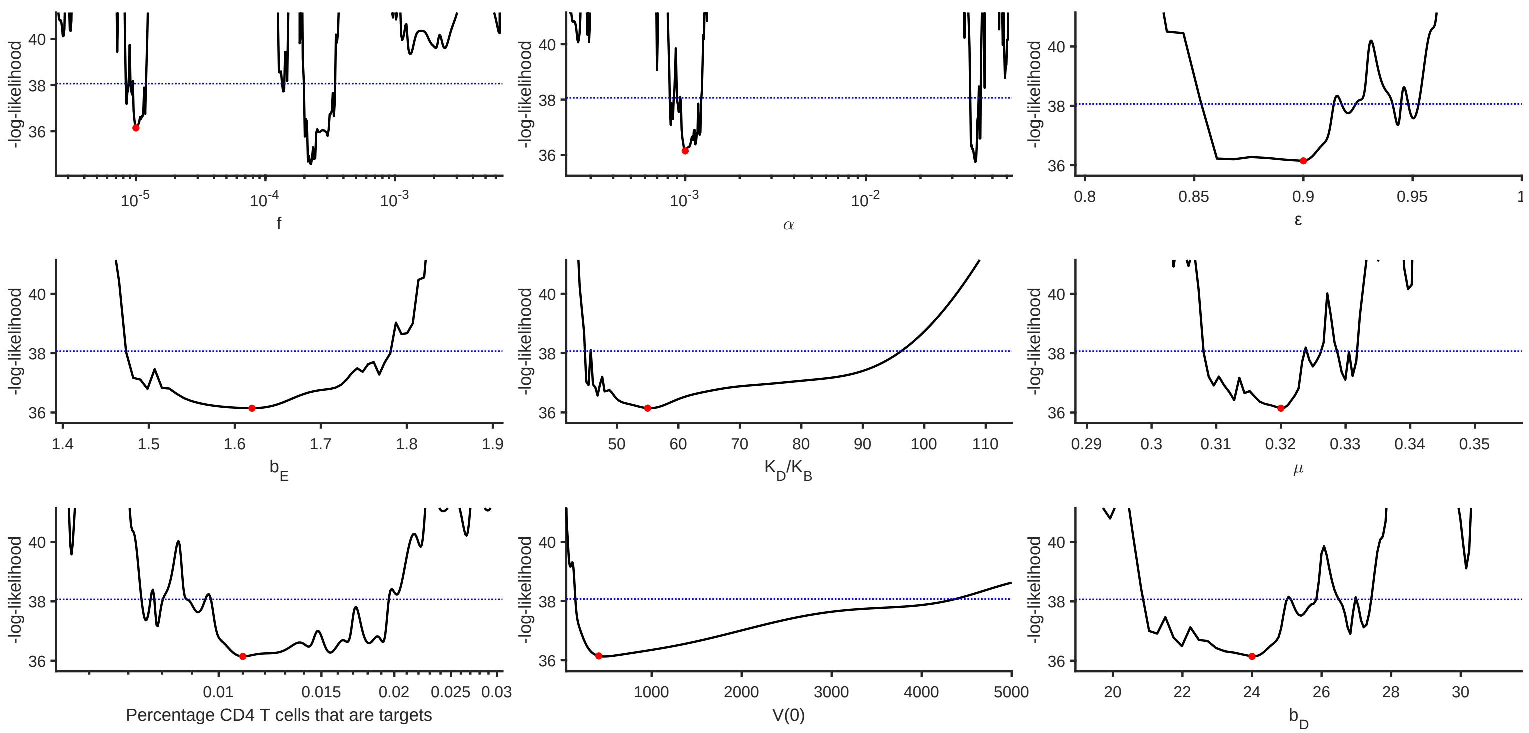

RId14:Viral neutralization (APCS)

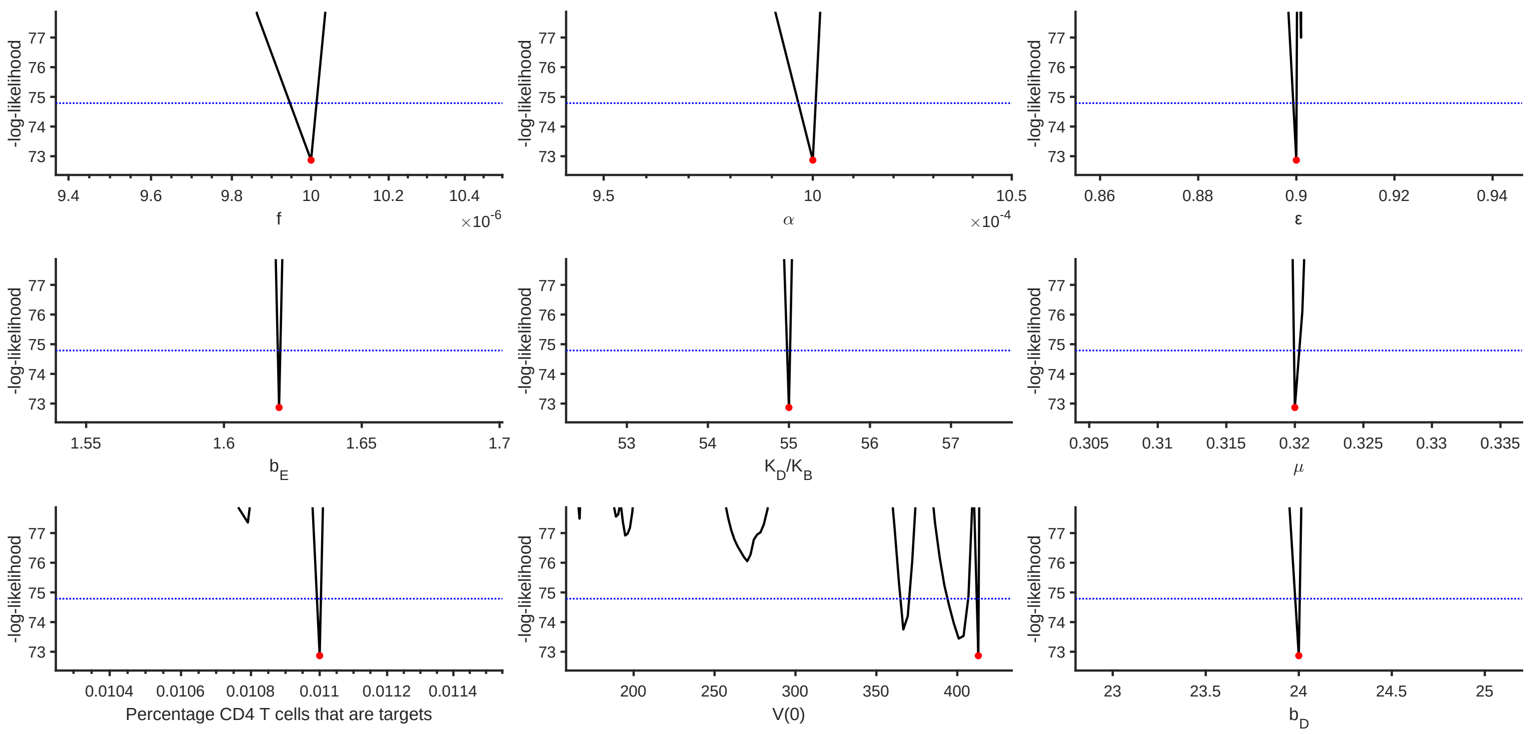

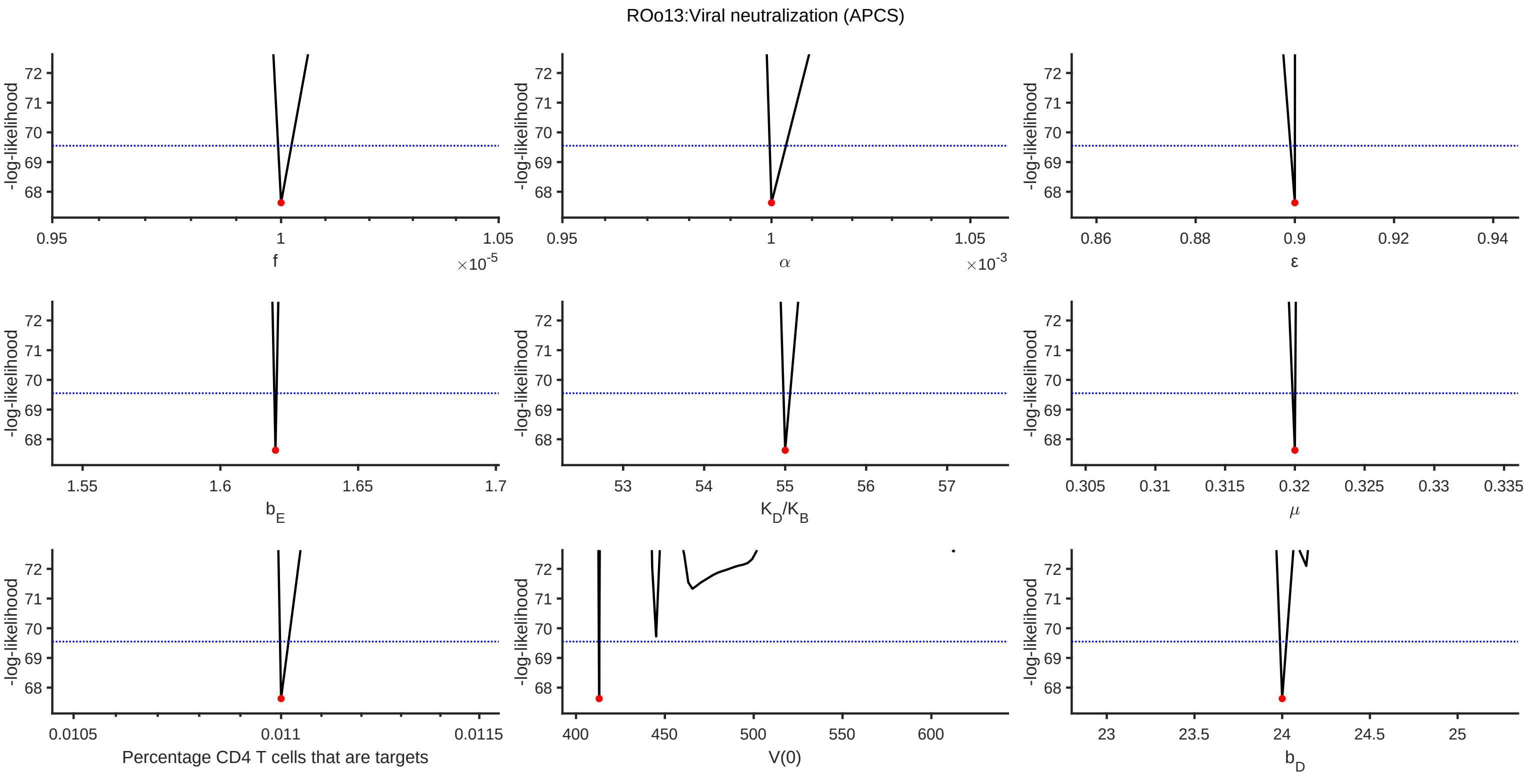
ROo13:Viral neutralization (APCS)
-log-likelihood
f
α
ε
b_E
K_D/K_B
μ
Percentage CD4 T cells that are targets
V(0)
b_D

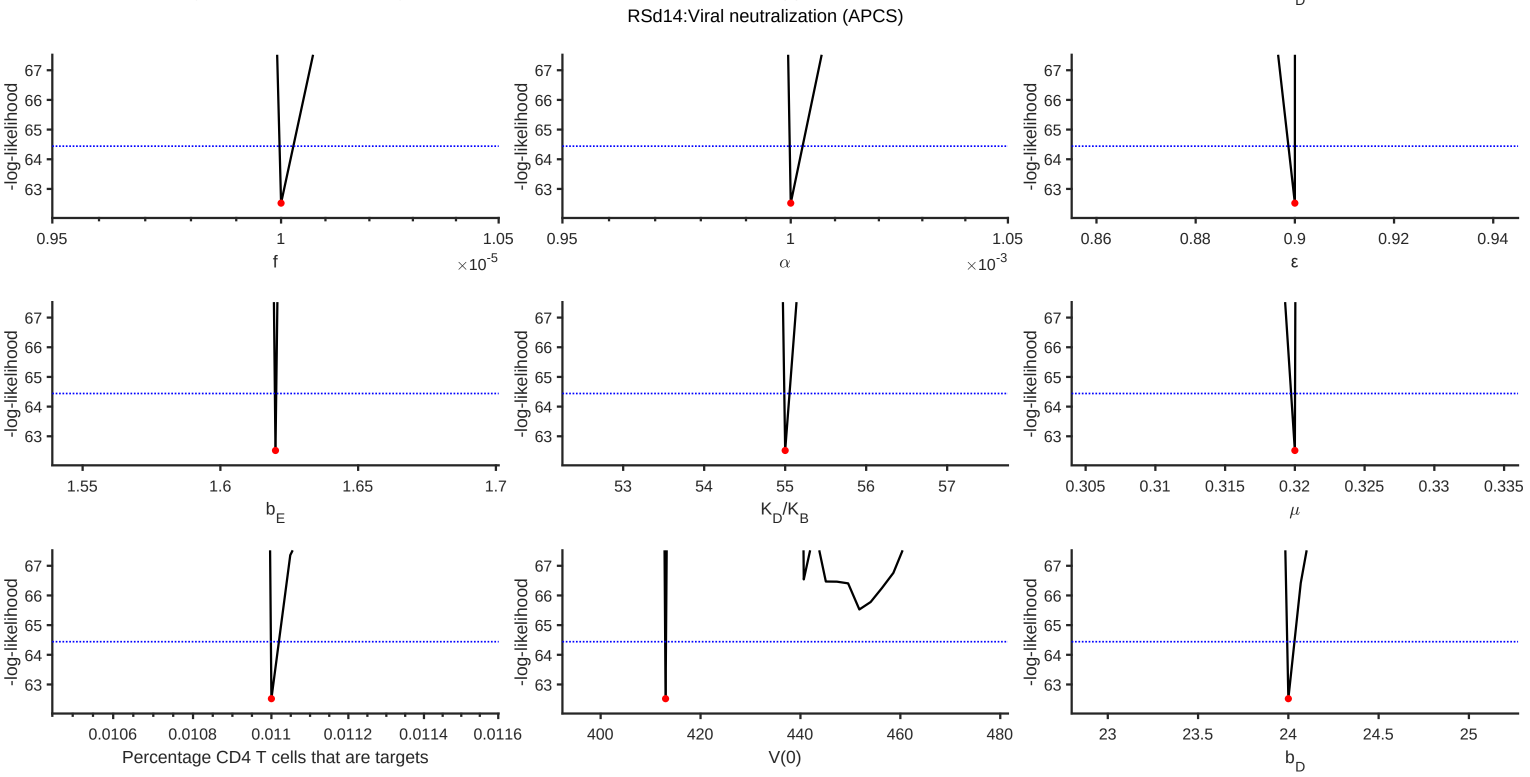
RSd14:Viral neutralization (APCS)
-log-likelihood
f
α
ε
b_E
K_D/K_B
μ
Percentage CD4 T cells that are targets
V(0)
b_D

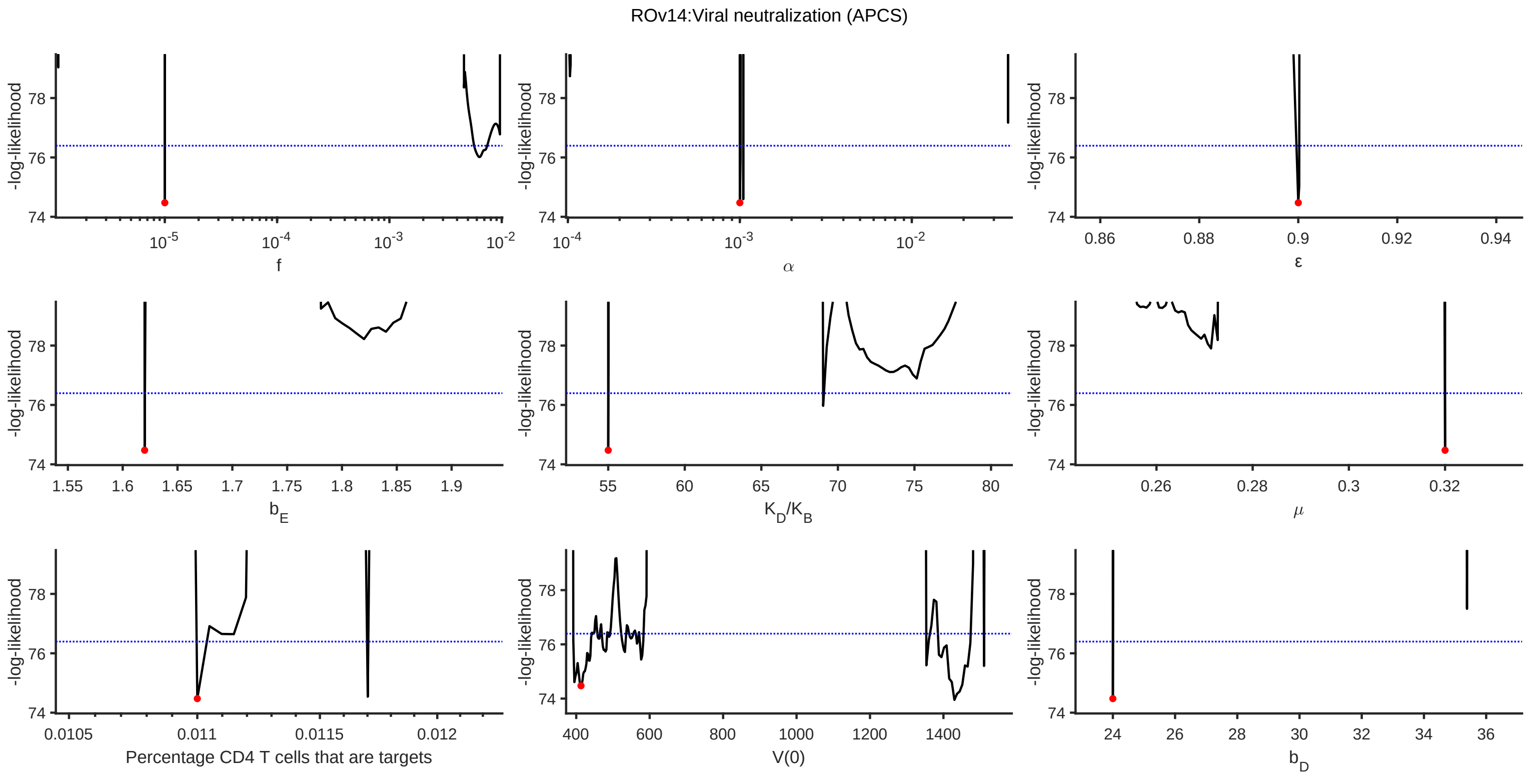
ROv14:Viral neutralization (APCS)
-log-likelihood
f
α
ε
b_E
K_D/K_B
μ
Percentage CD4 T cells that are targets
V(0)
b_D

7 Likelihood profiles: Protection

7.1 Baseline effector cell source model

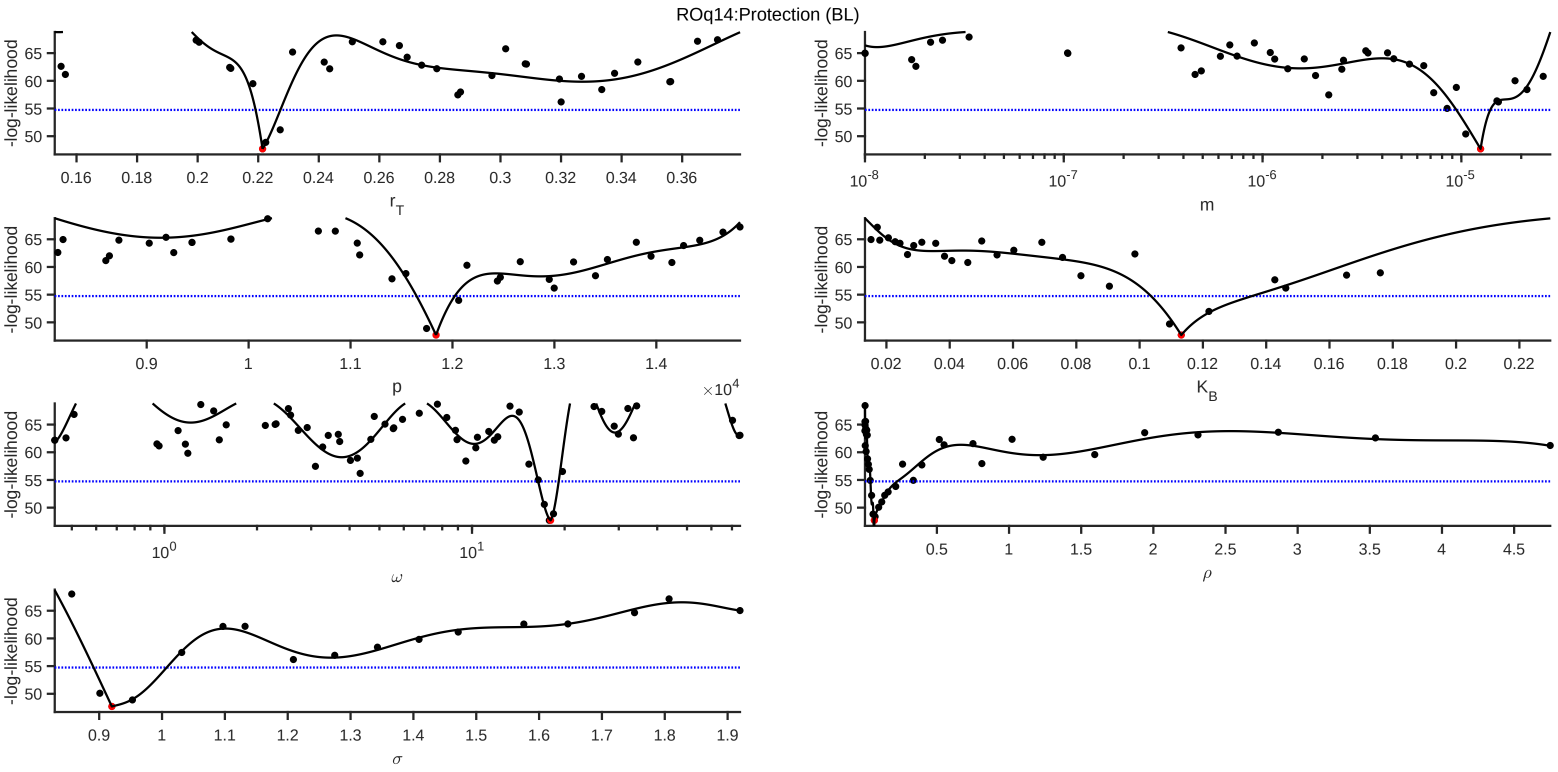

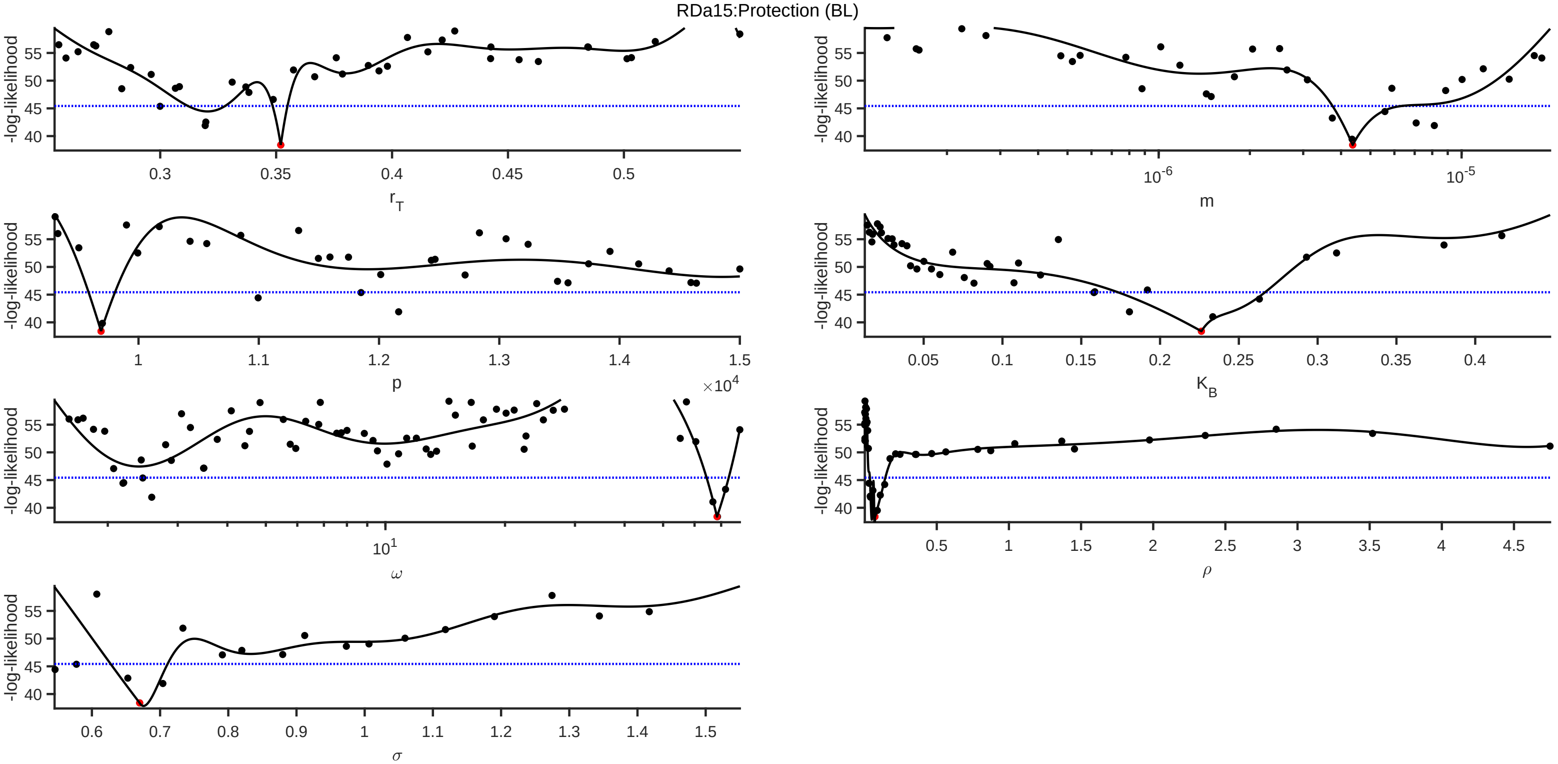
RDa15:Protection (BL)
-log-likelihood
r_T
m
p
K_B
ω
ρ
σ

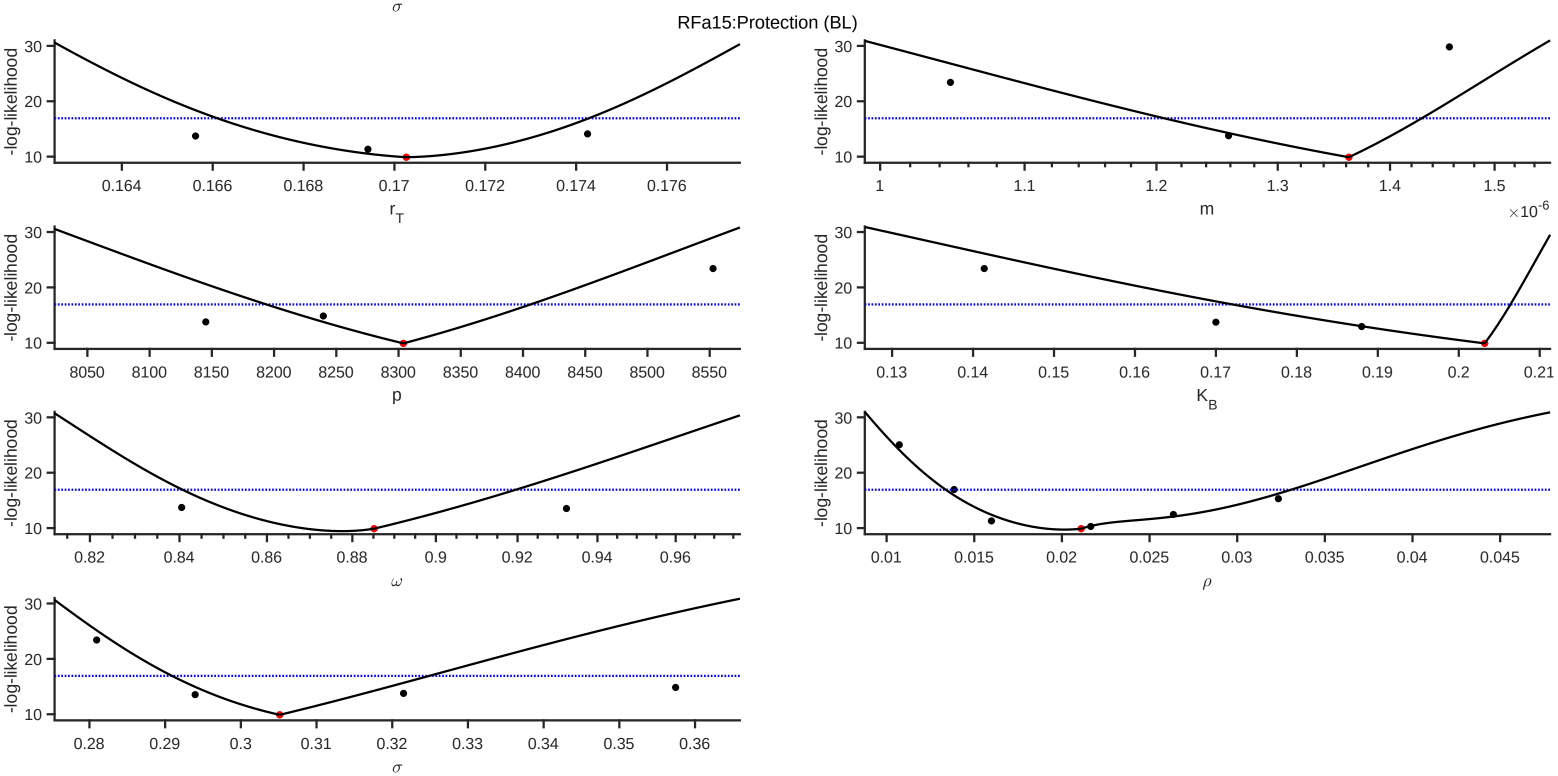
RFa15:Protection (BL)
-log-likelihood
r_T
m
p
K_B
ω
ρ
σ

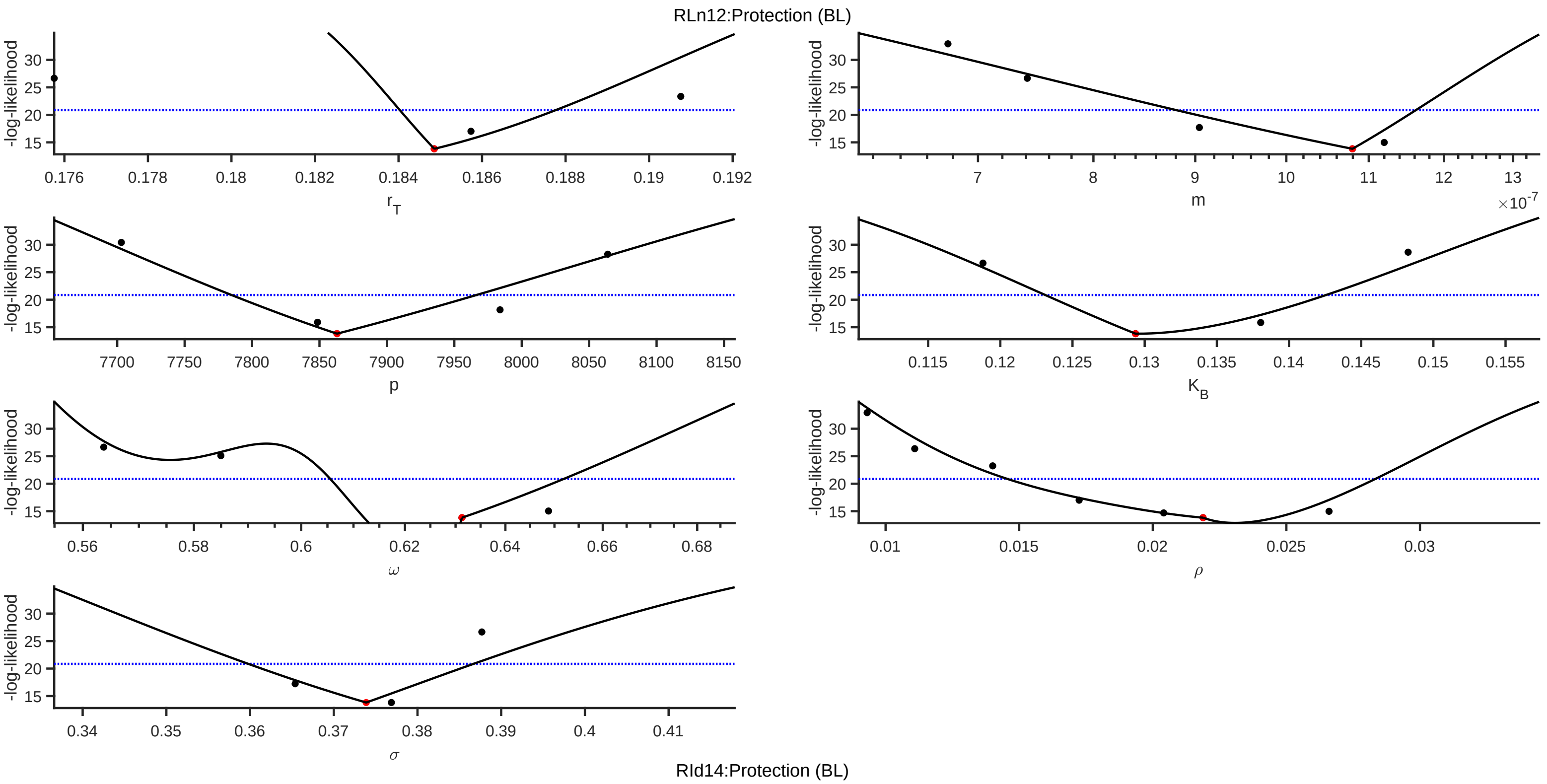
RLn12:Protection (BL)
-log-likelihood
r_T
m
×10^-7
p
K_B
ω
ρ
σ

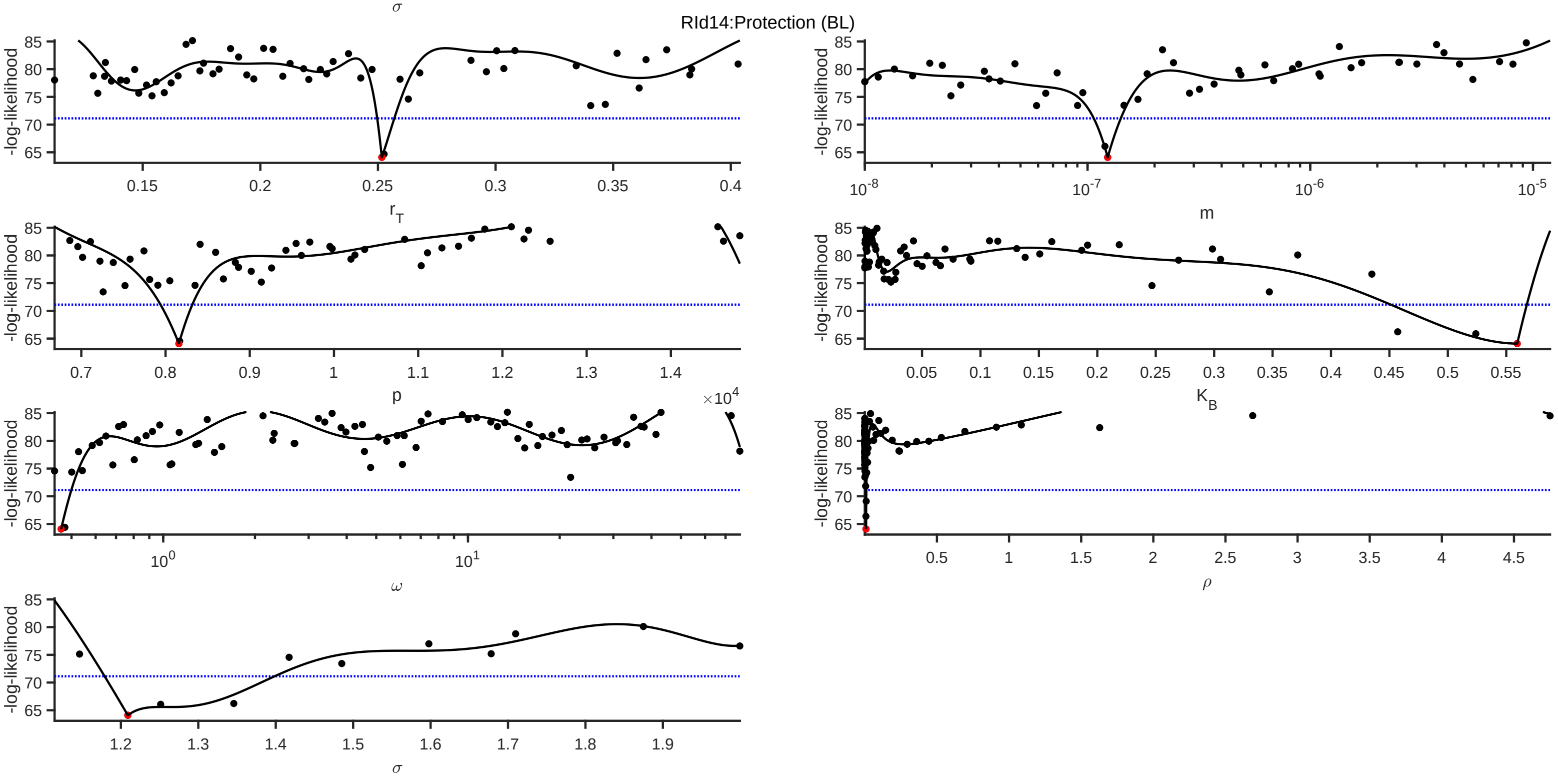
RId14:Protection (BL)
-log-likelihood
r_T
m
p
×10^4
K_B
ω
ρ
σ

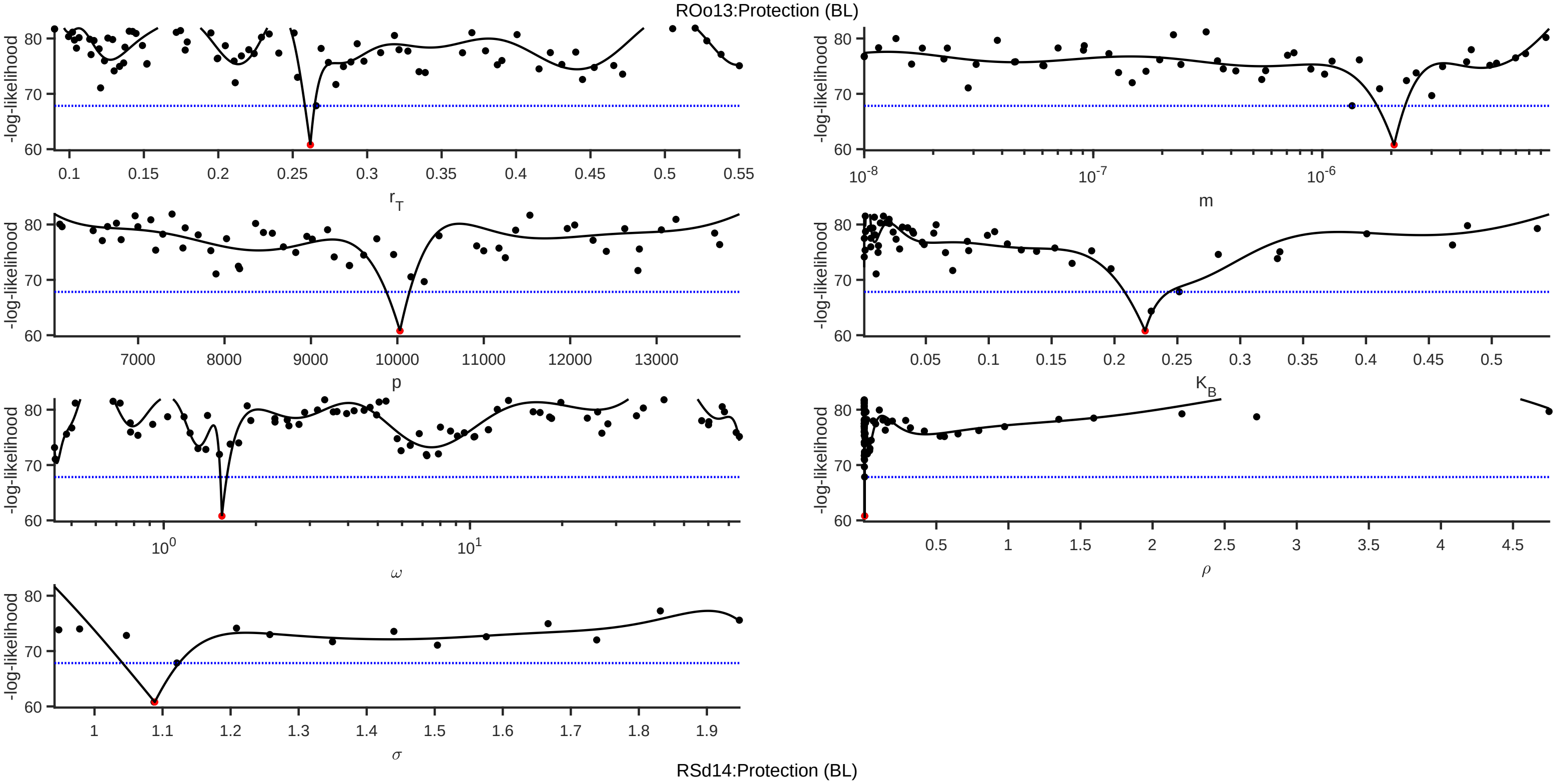
ROo13:Protection (BL)
-log-likelihood
r_T
m
p
K_B
ω
ρ
σ

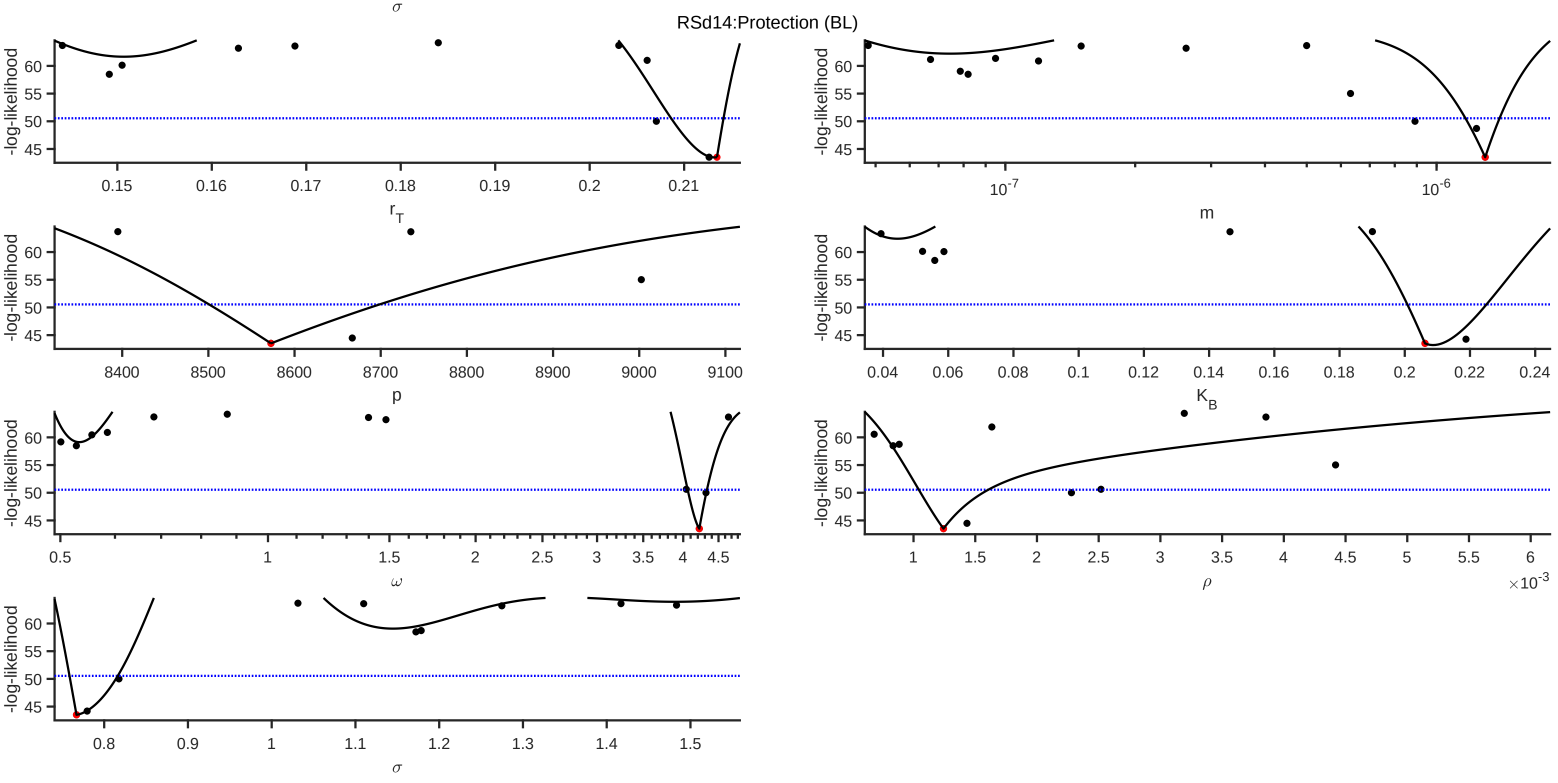
RSd14:Protection (BL)
-log-likelihood
r_T
m
p
K_B
ω
ρ
$\times 10^{-3}$
σ

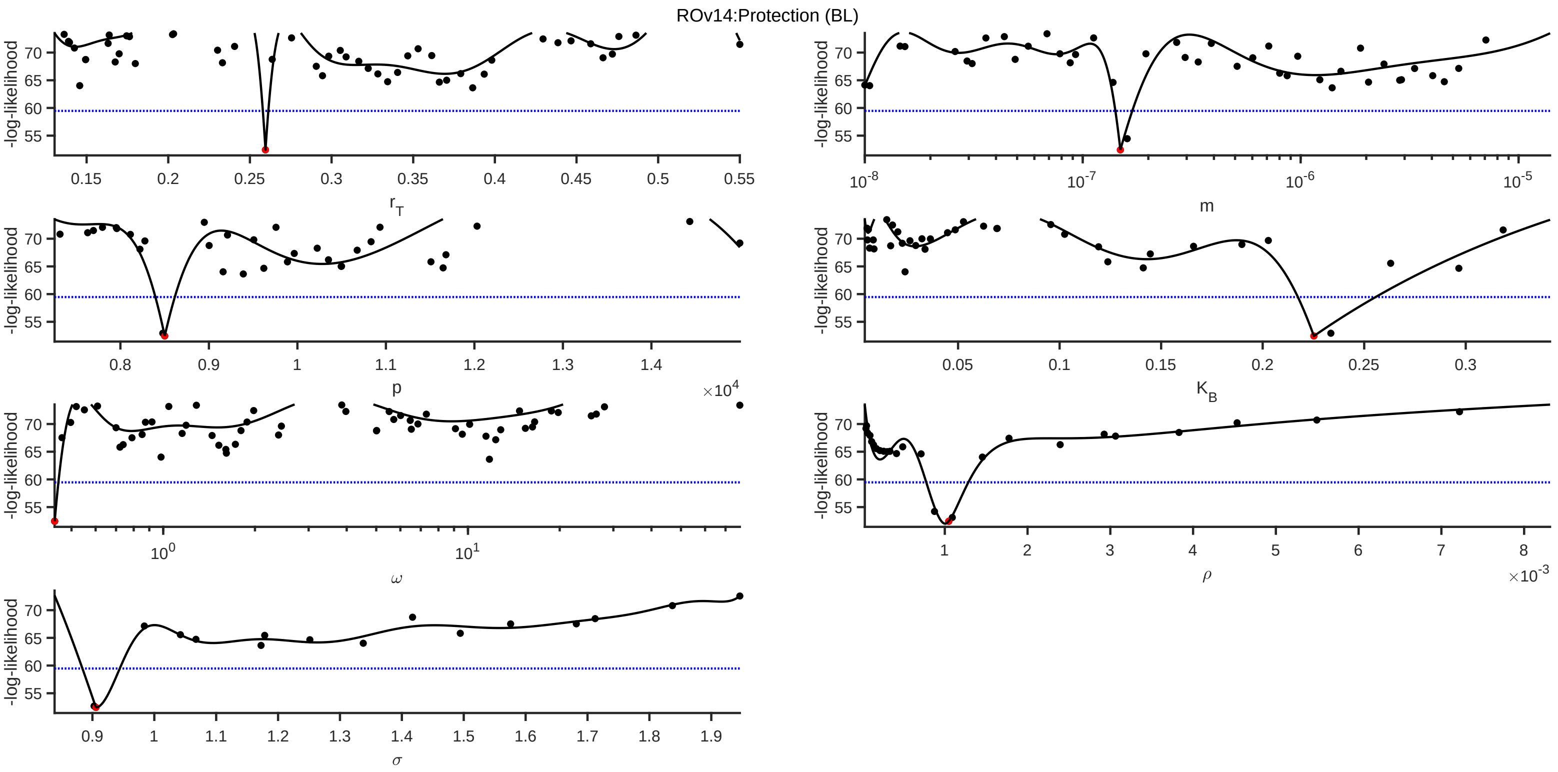

ROv14:Protection (BL)
-log-likelihood
r_T
m
p
K_B
ω
ρ
σ
×10^4
×10^-3

7.2 Saturated effector cell source model

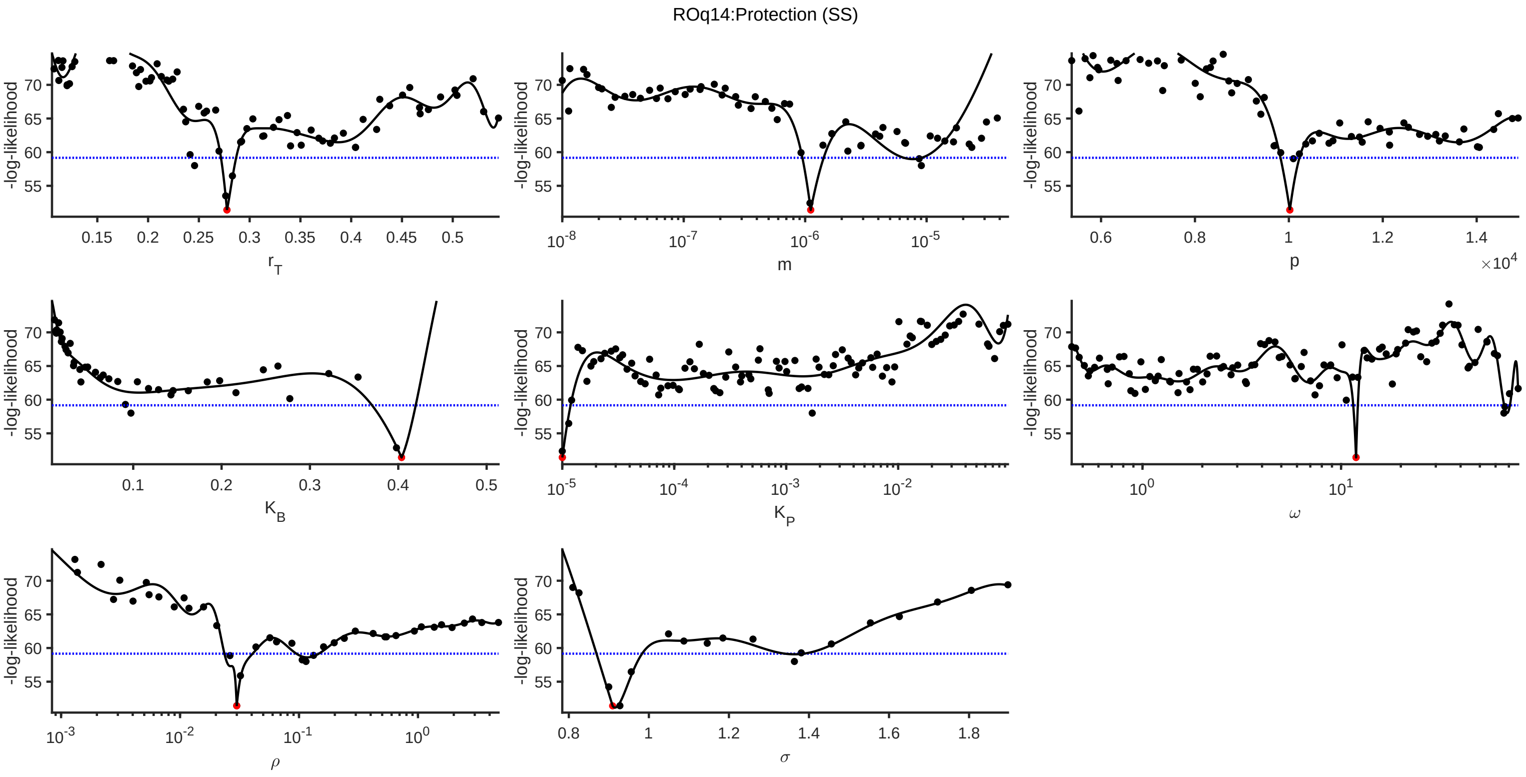

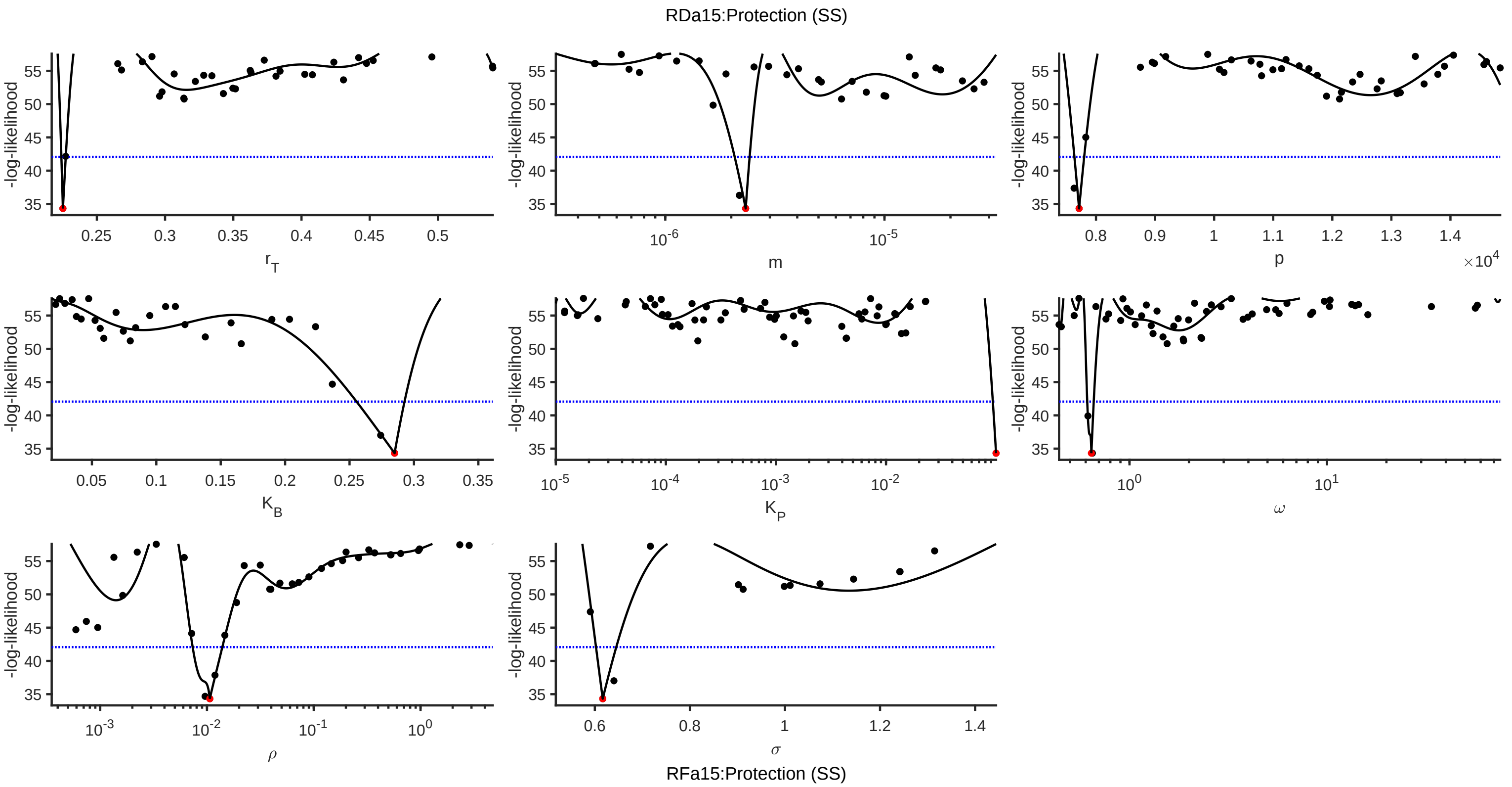
RDa15:Protection (SS)
-log-likelihood
r_T
m
p
$\times 10^4$
K_B
K_P
ω
ρ
σ

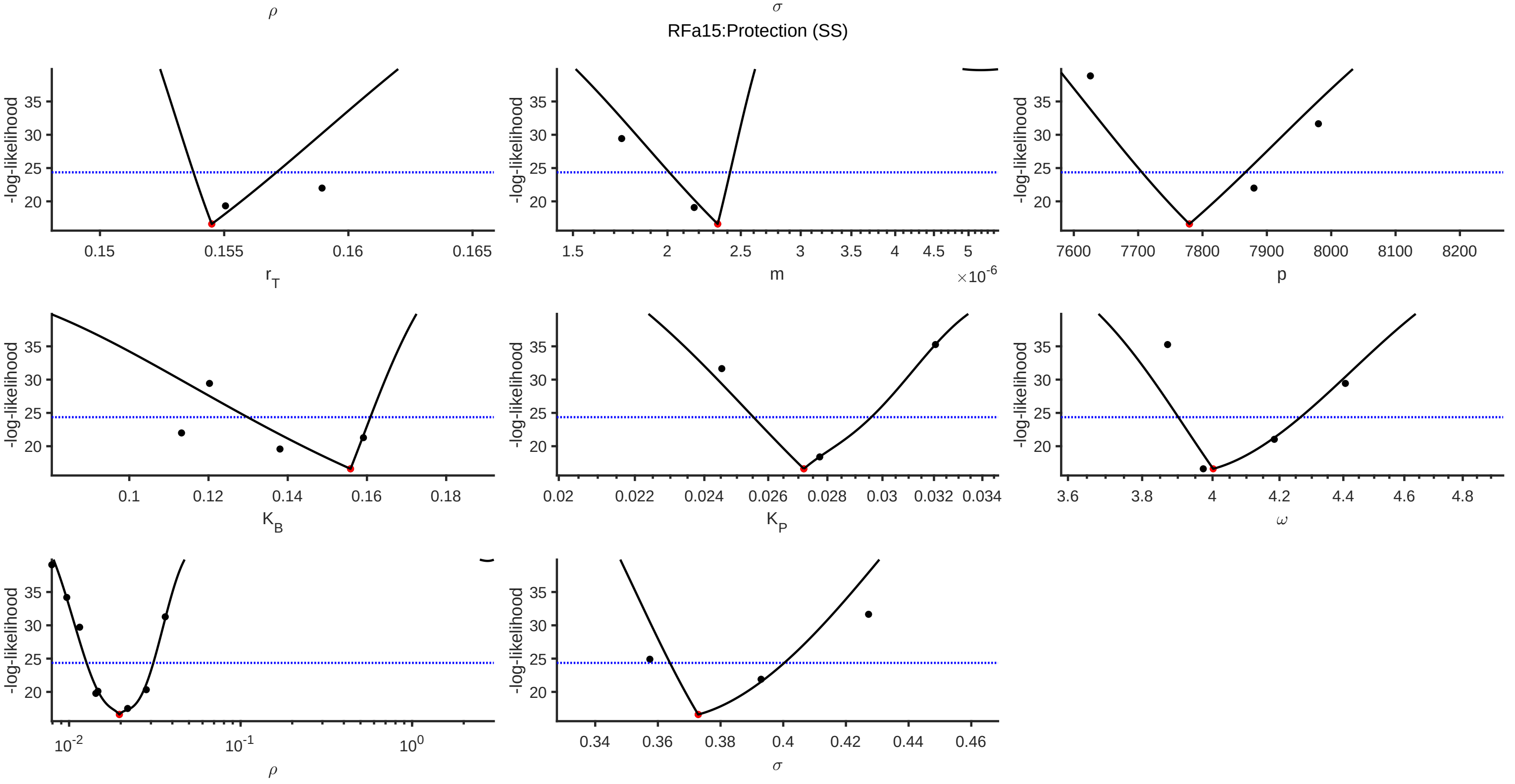
RFa15:Protection (SS)
-log-likelihood
r_T
m
$\times 10^{-6}$
p
K_B
K_P
ω
ρ
σ

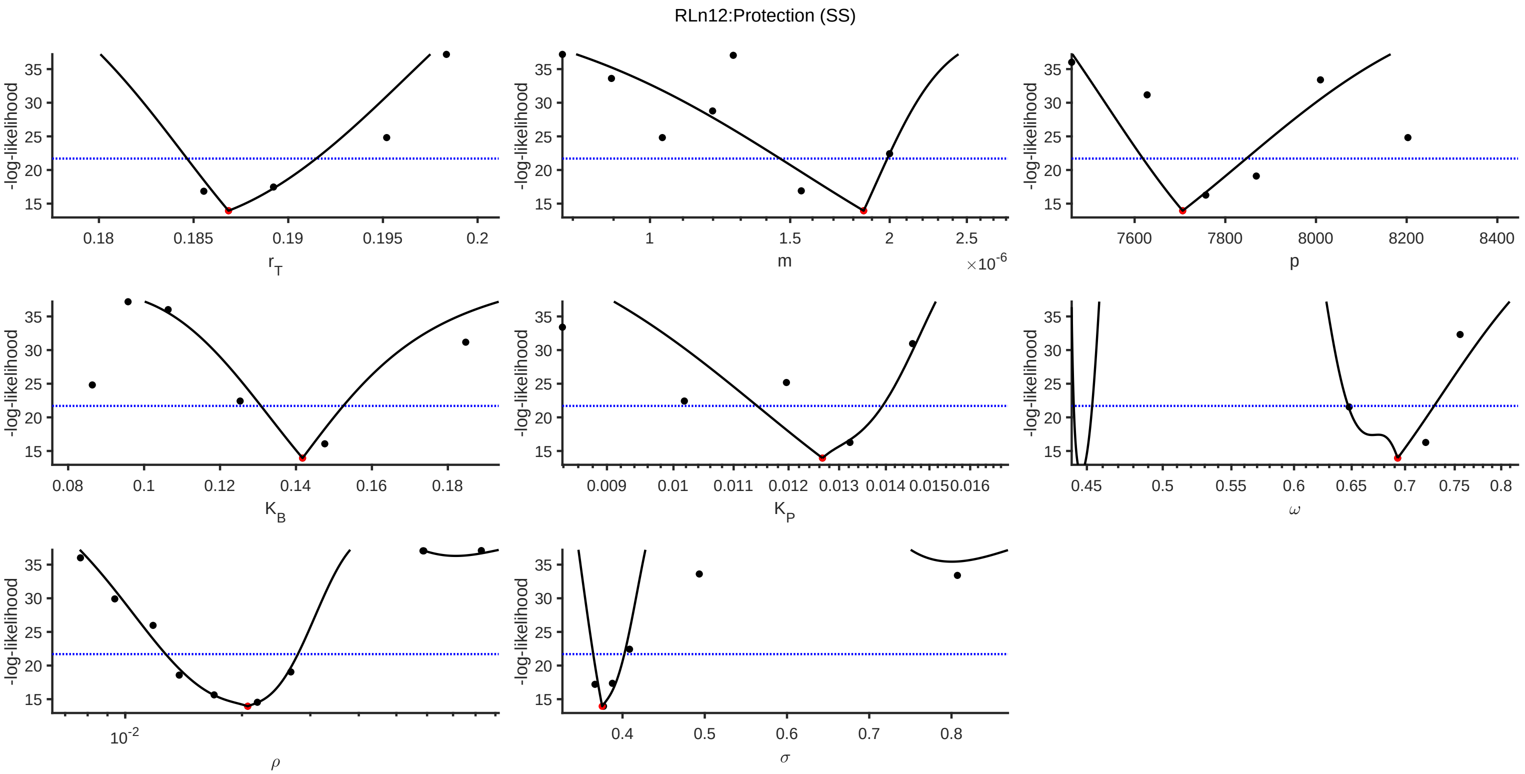
RLn12:Protection (SS)
-log-likelihood
r_T
m
×10^-6
p
K_B
K_P
ω
ρ
σ

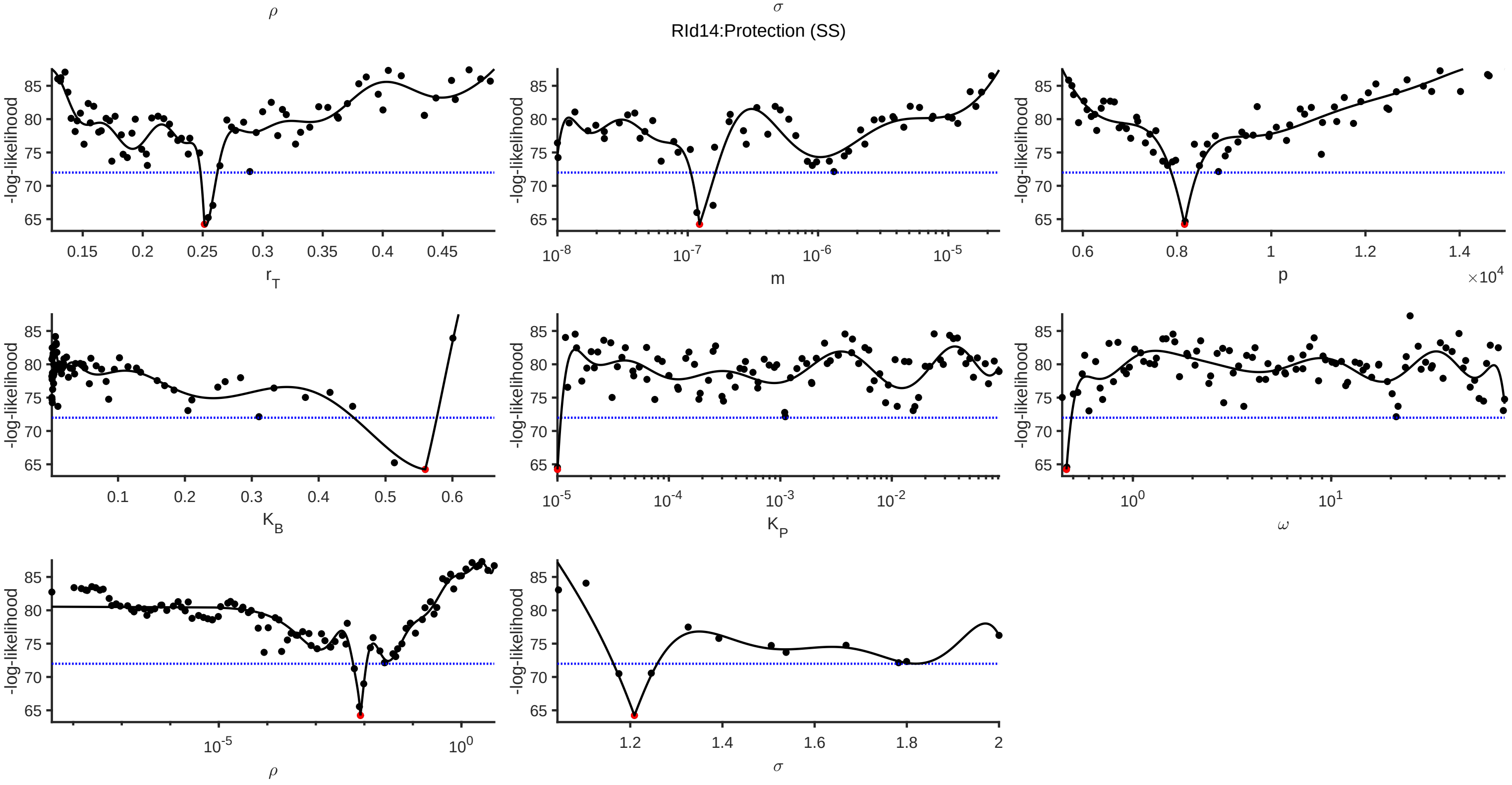
RId14:Protection (SS)
-log-likelihood
r_T
m
p
×10^4
K_B
K_P
ω
ρ
σ

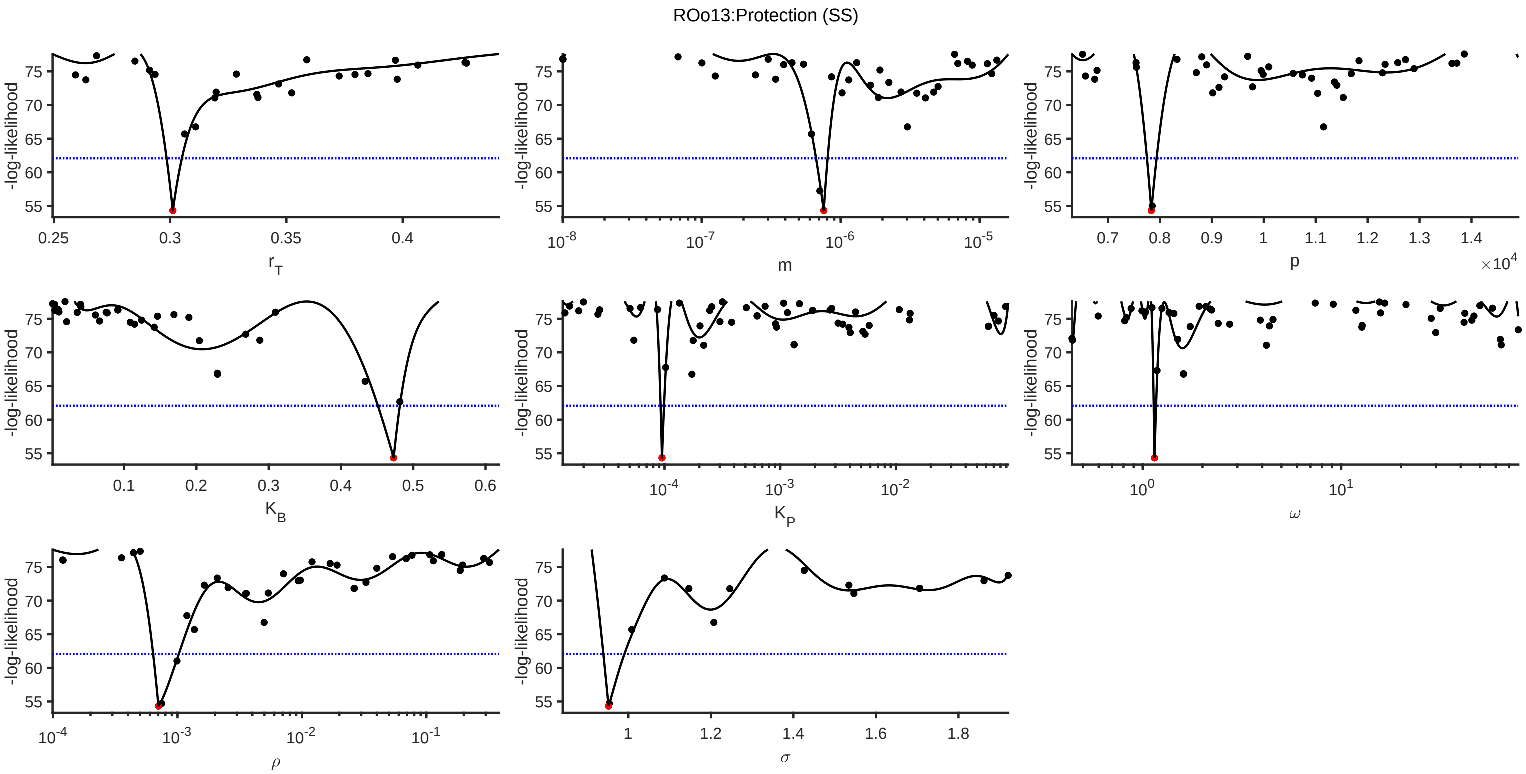
ROo13:Protection (SS)
-log-likelihood
r_T
m
p
$\times 10^4$
K_B
K_P
ω
ρ
σ

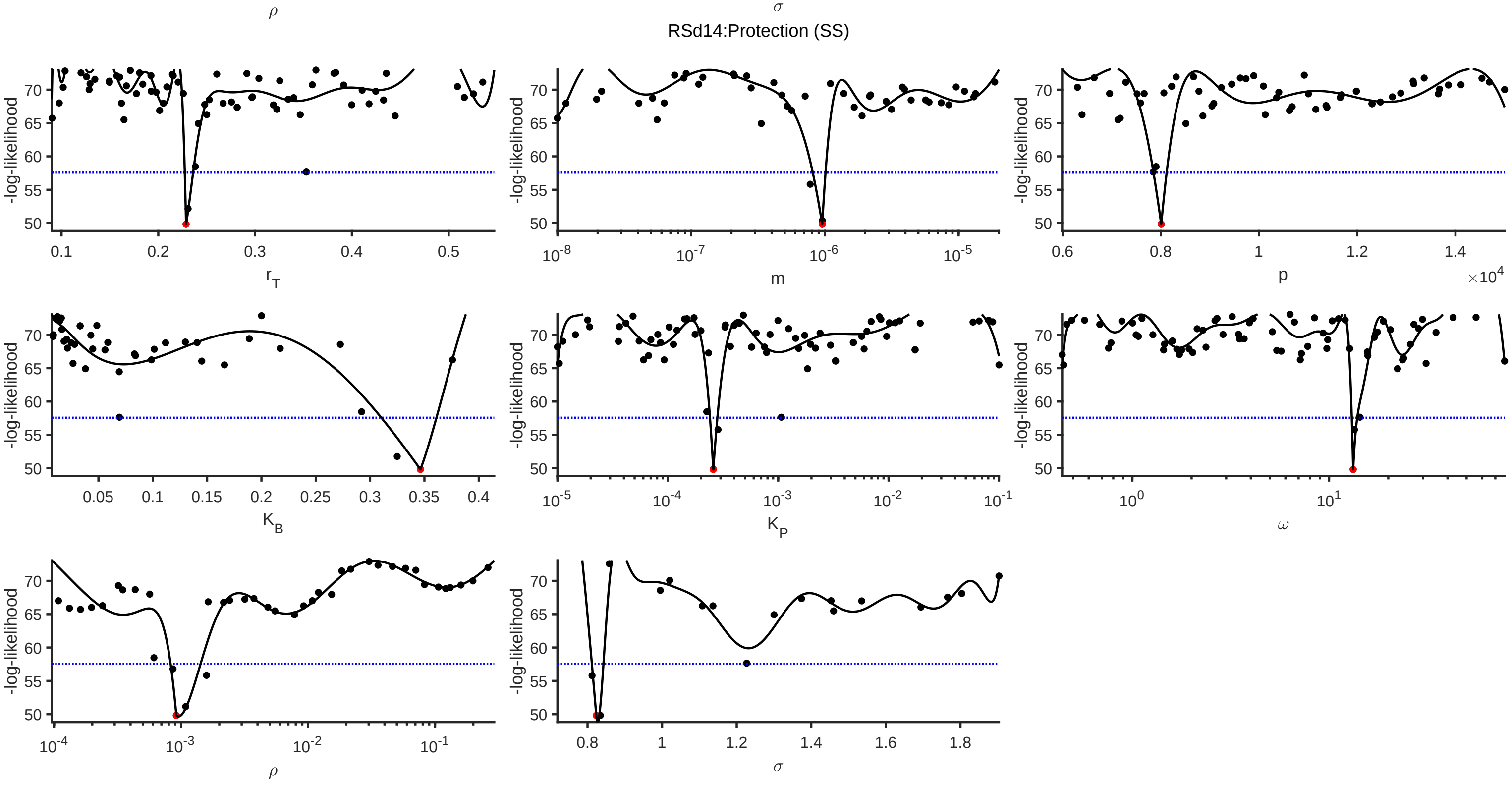
RSd14:Protection (SS)
-log-likelihood
r_T
m
p
$\times 10^4$
K_B
K_P
ω
ρ
σ

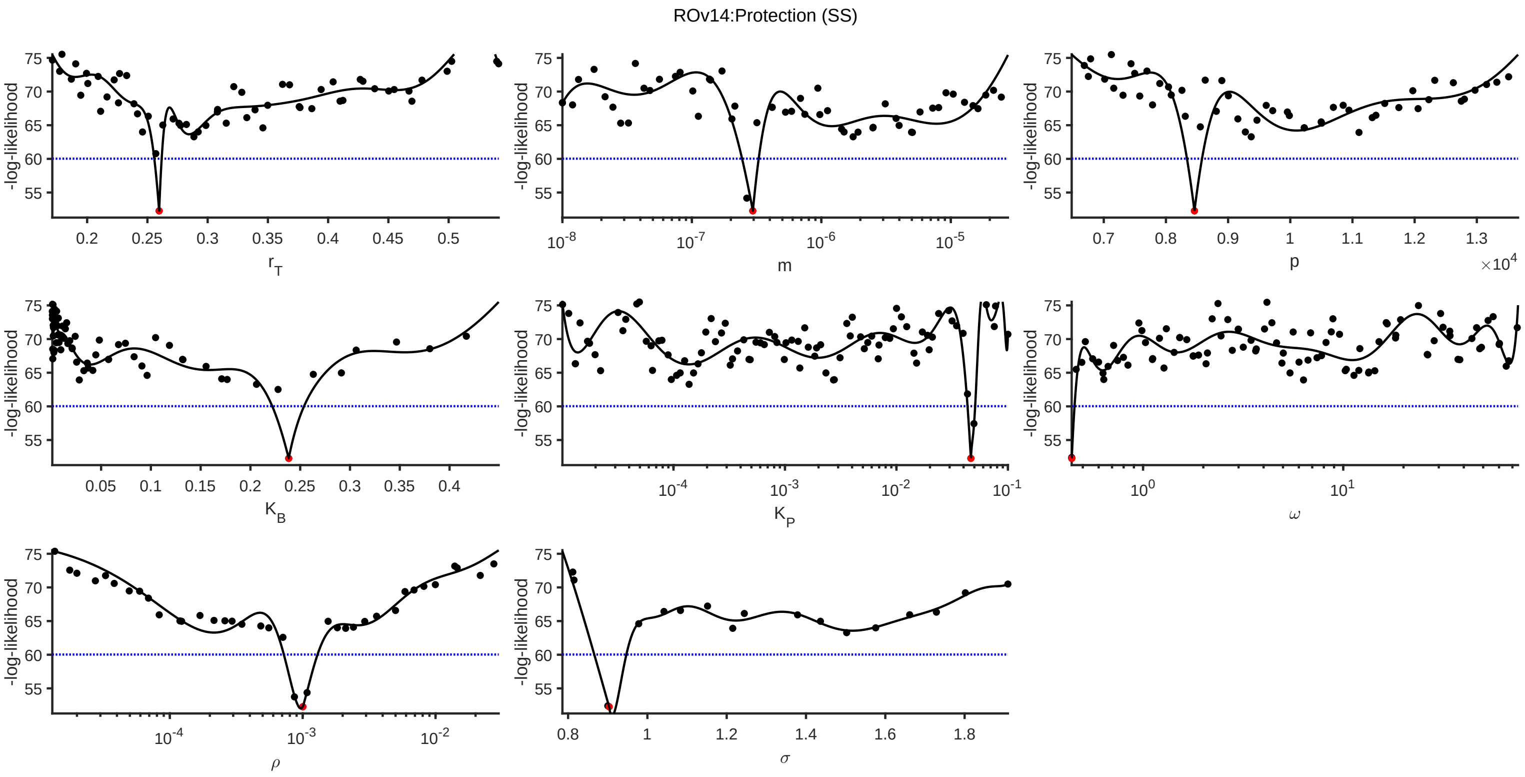

ROv14:Protection (SS)
-log-likelihood
r_T
m
p
$\times 10^4$
K_B
K_P
ω
ρ
σ

7.3 Antigen presenting cell effector cell source model

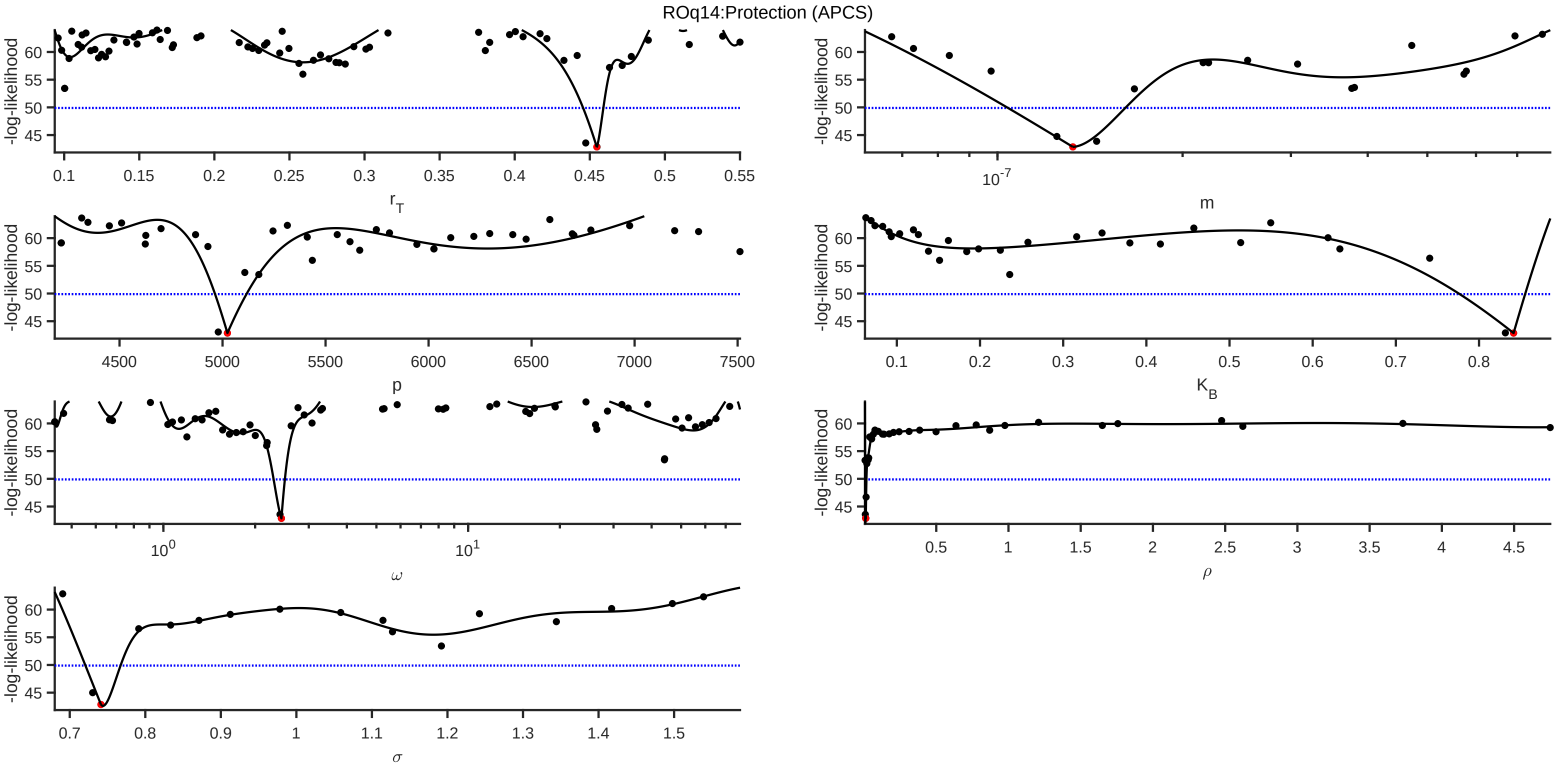

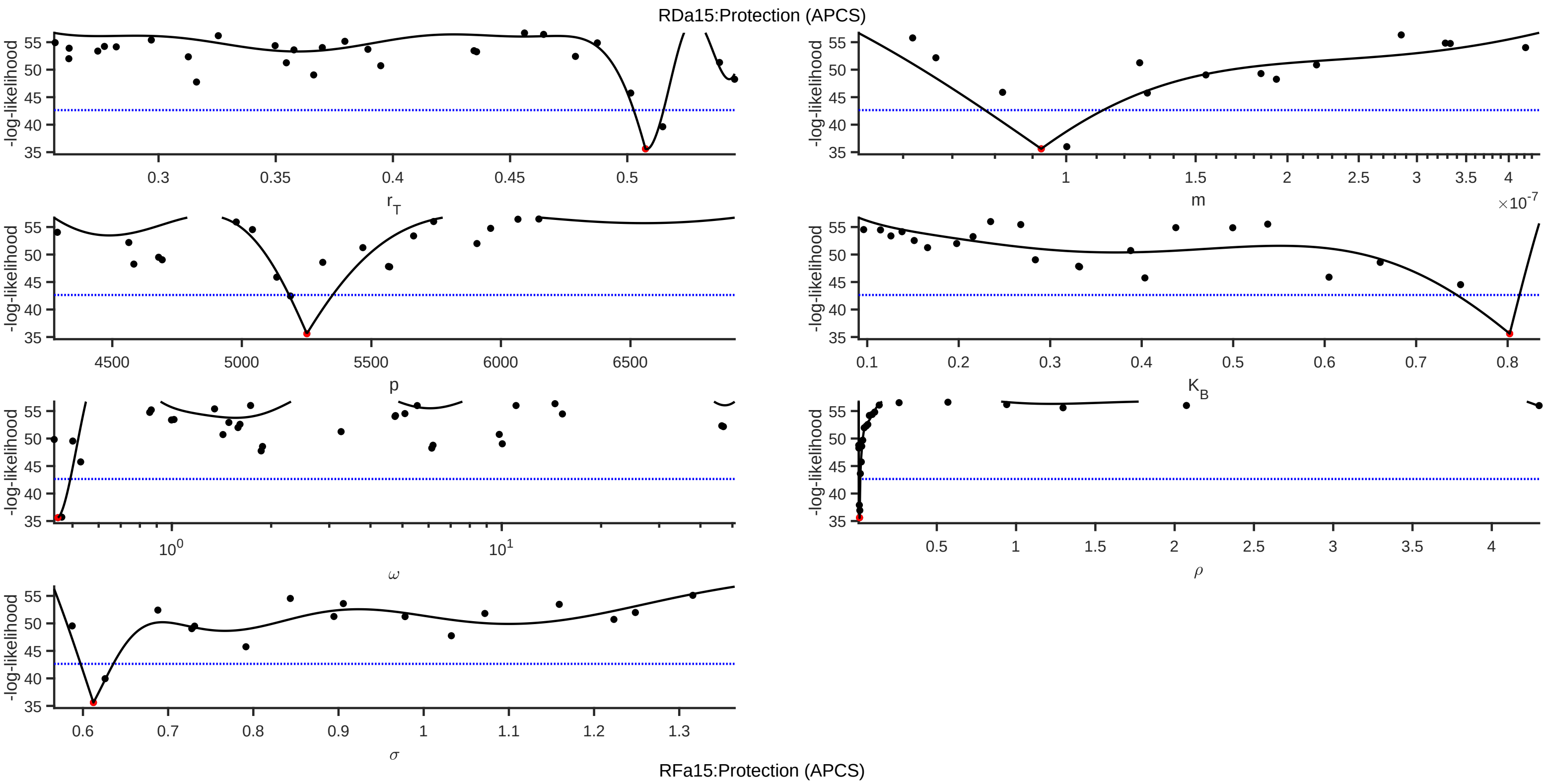
RDa15:Protection (APCS)
-log-likelihood
r_T
m
$\times 10^{-7}$
p
K_B
ω
ρ
σ

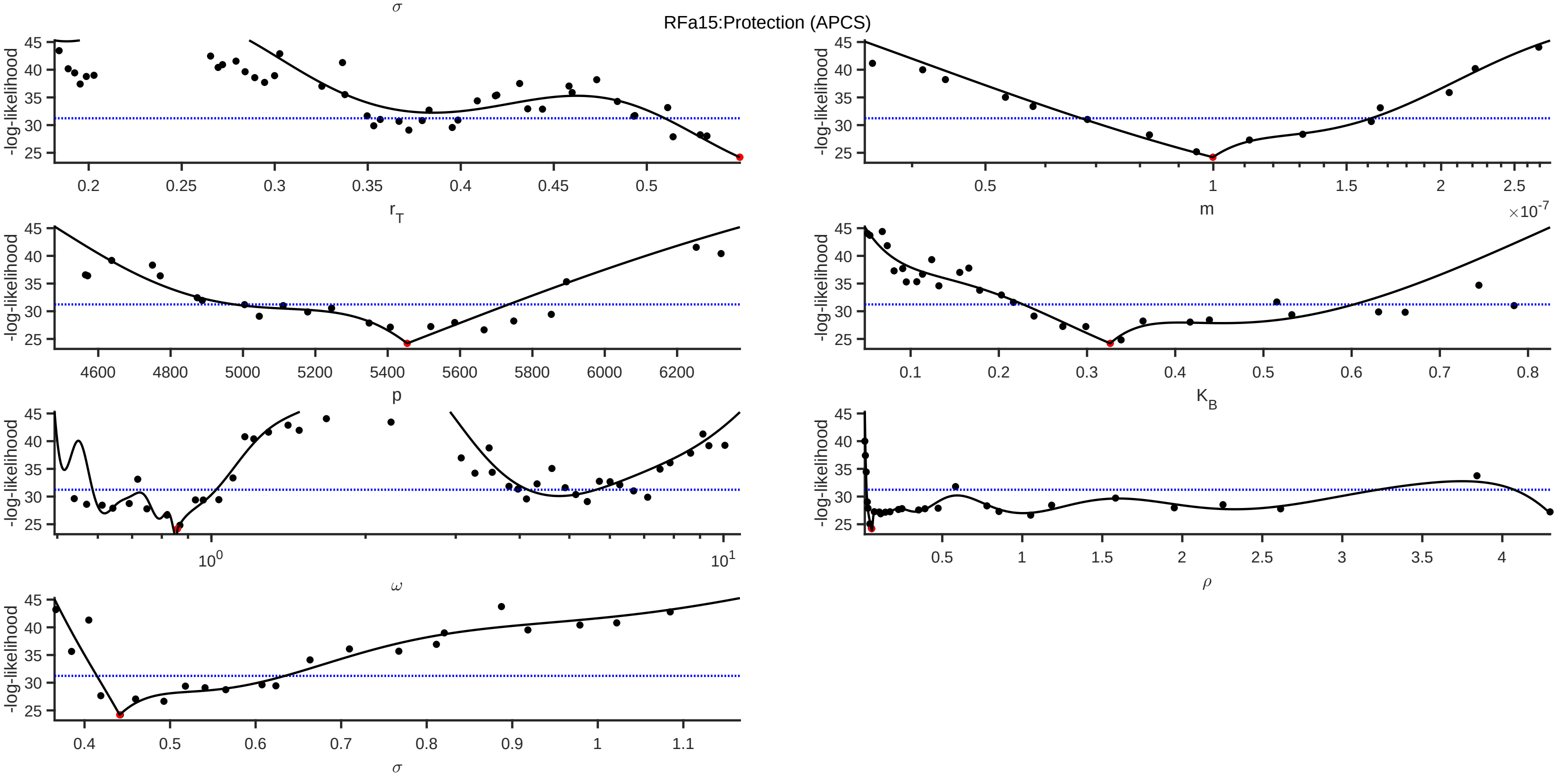
RFa15:Protection (APCS)
-log-likelihood
r_T
m
$\times 10^{-7}$
p
K_B
ω
ρ
σ

RLn12:Protection (APCS)

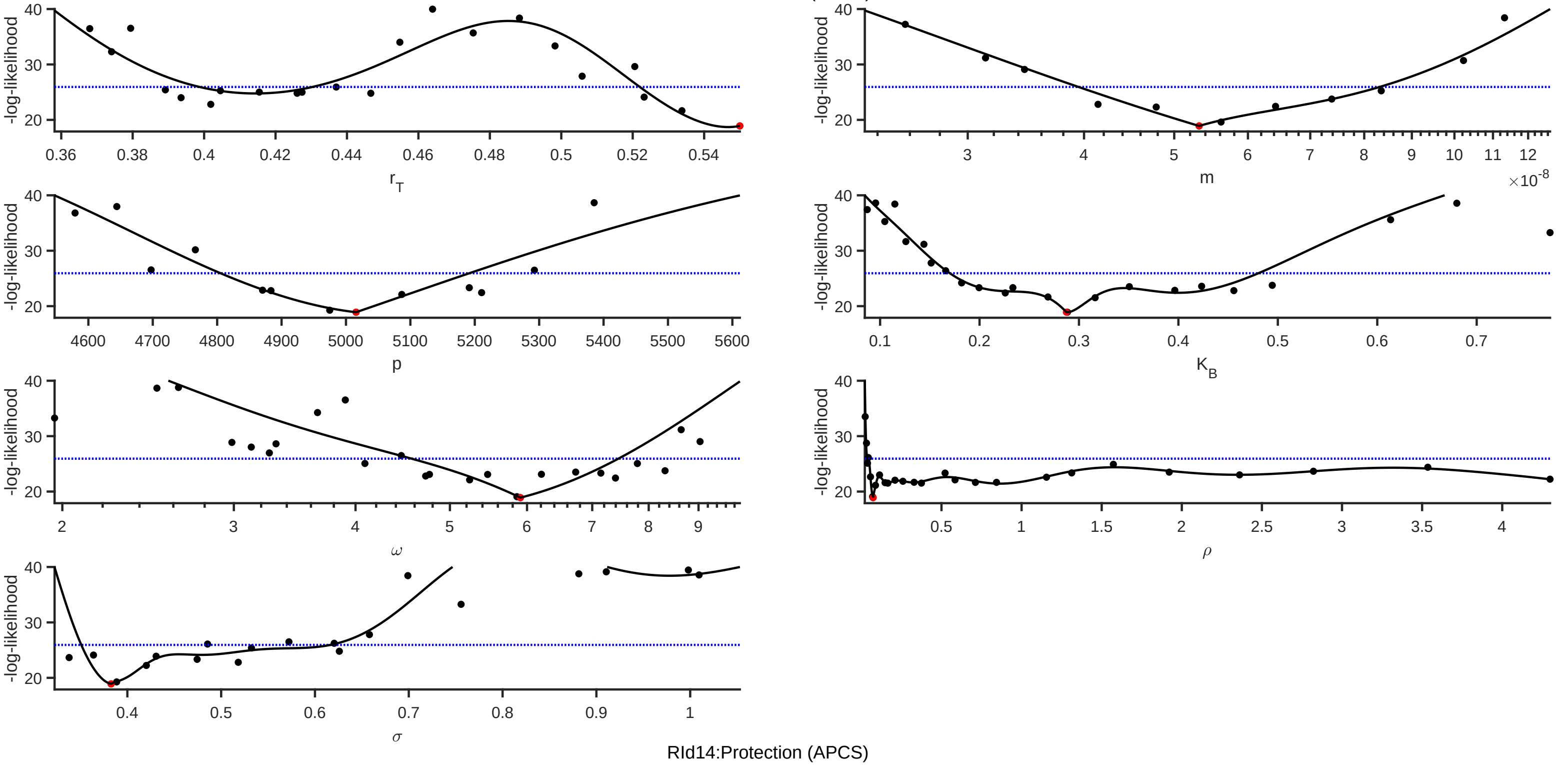

RId14:Protection (APCS)

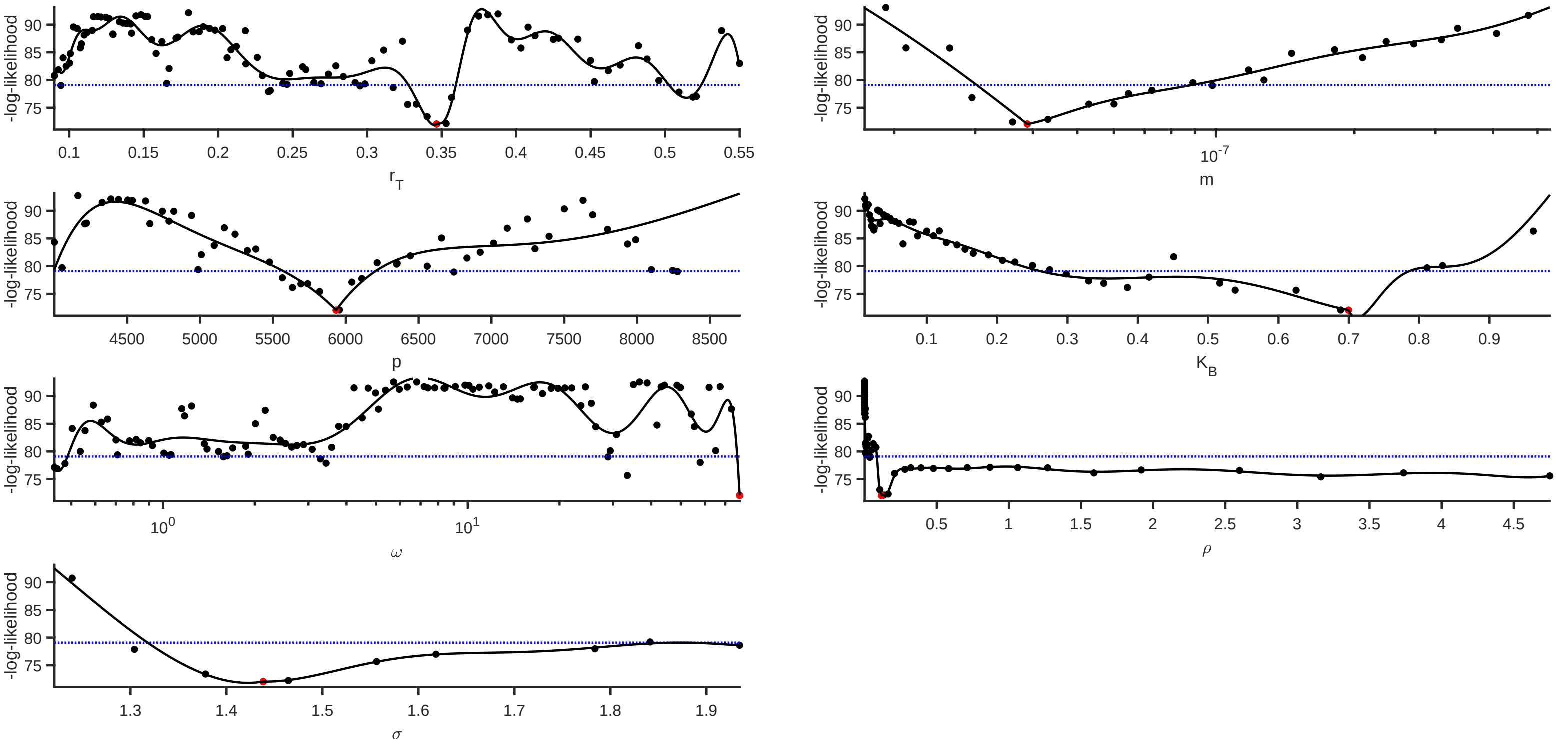

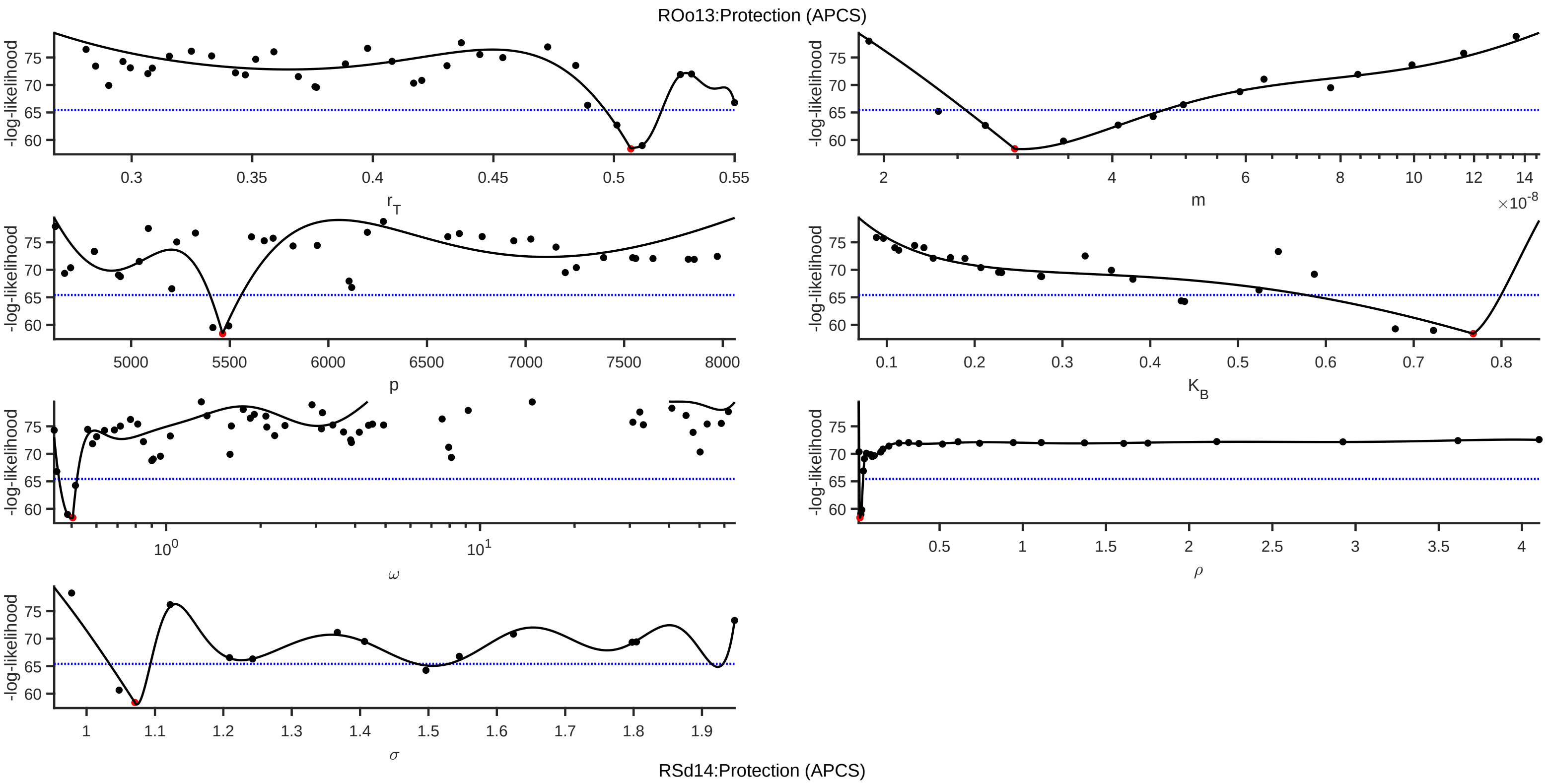
ROo13:Protection (APCS)
-log-likelihood
r_T
m
×10^-8
p
K_B
ω
ρ
σ

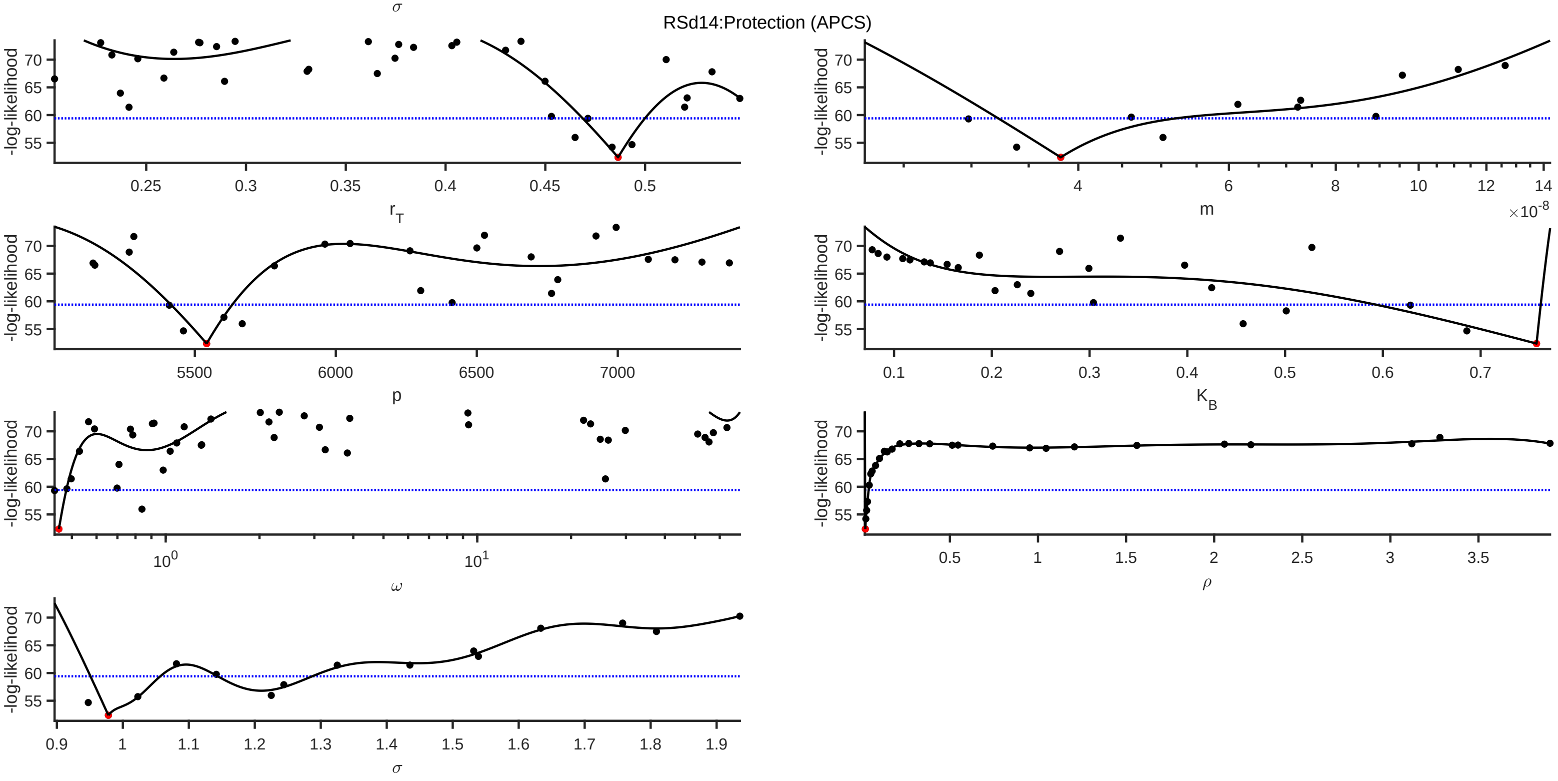
RSd14:Protection (APCS)
-log-likelihood
r_T
m
×10^-8
p
K_B
ω
ρ
σ

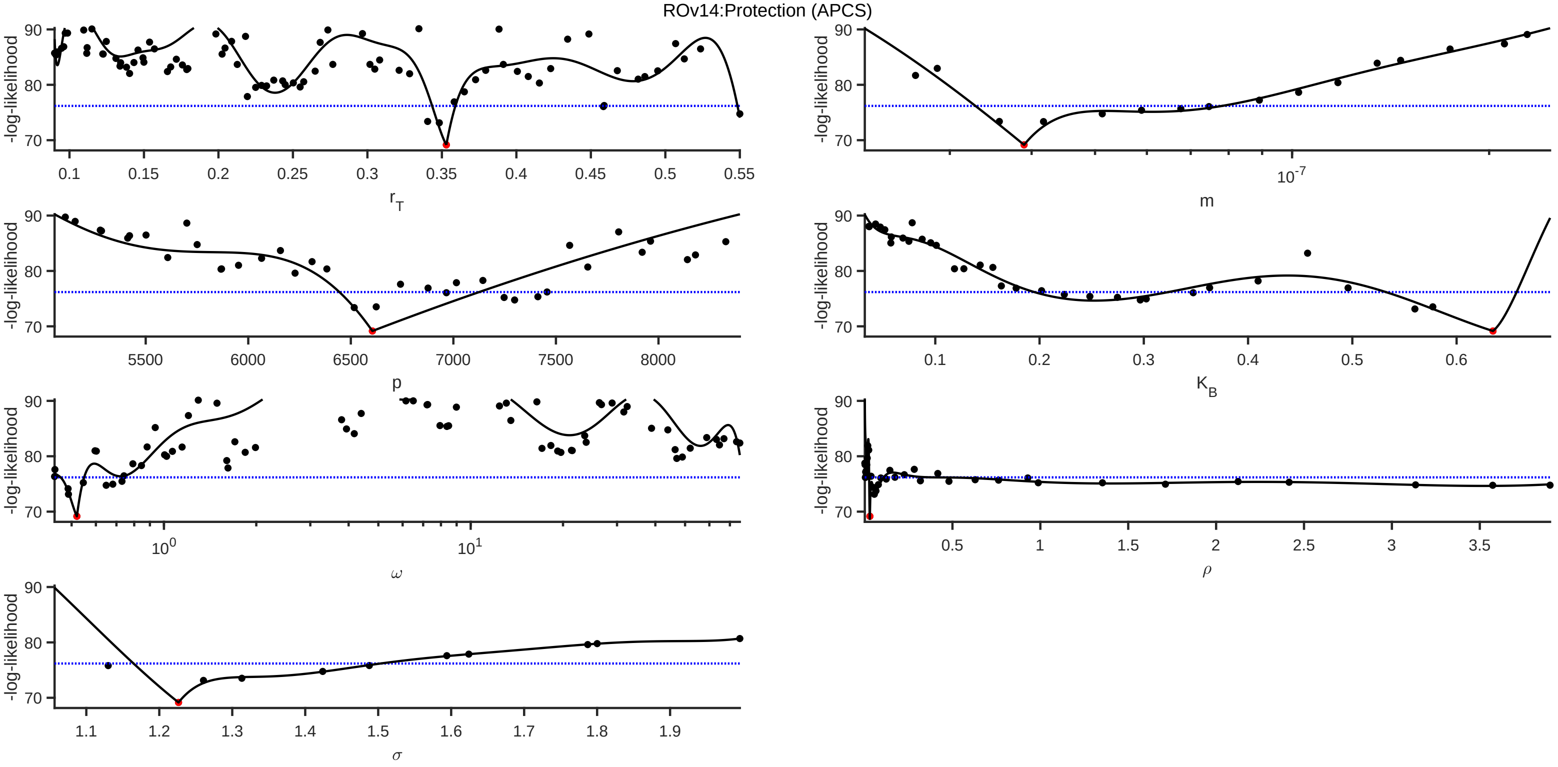
ROv14:Protection (APCS)
-log-likelihood
r_T
m
p
K_B
ω
ρ
σ

8 Sensitivity anlaysis: Protection

8.1 Baseline effector cell source model

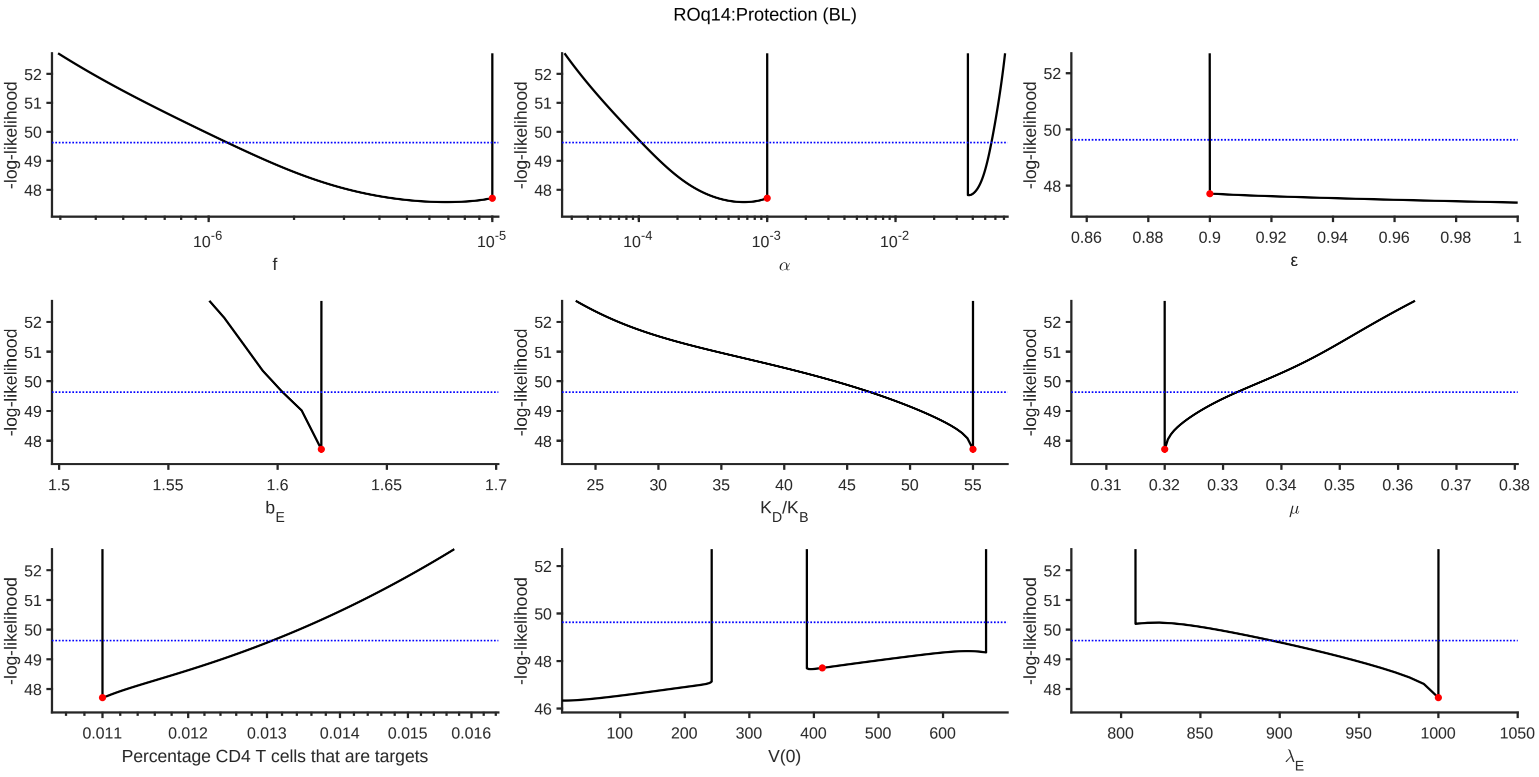

RDa15:Protection (BL)

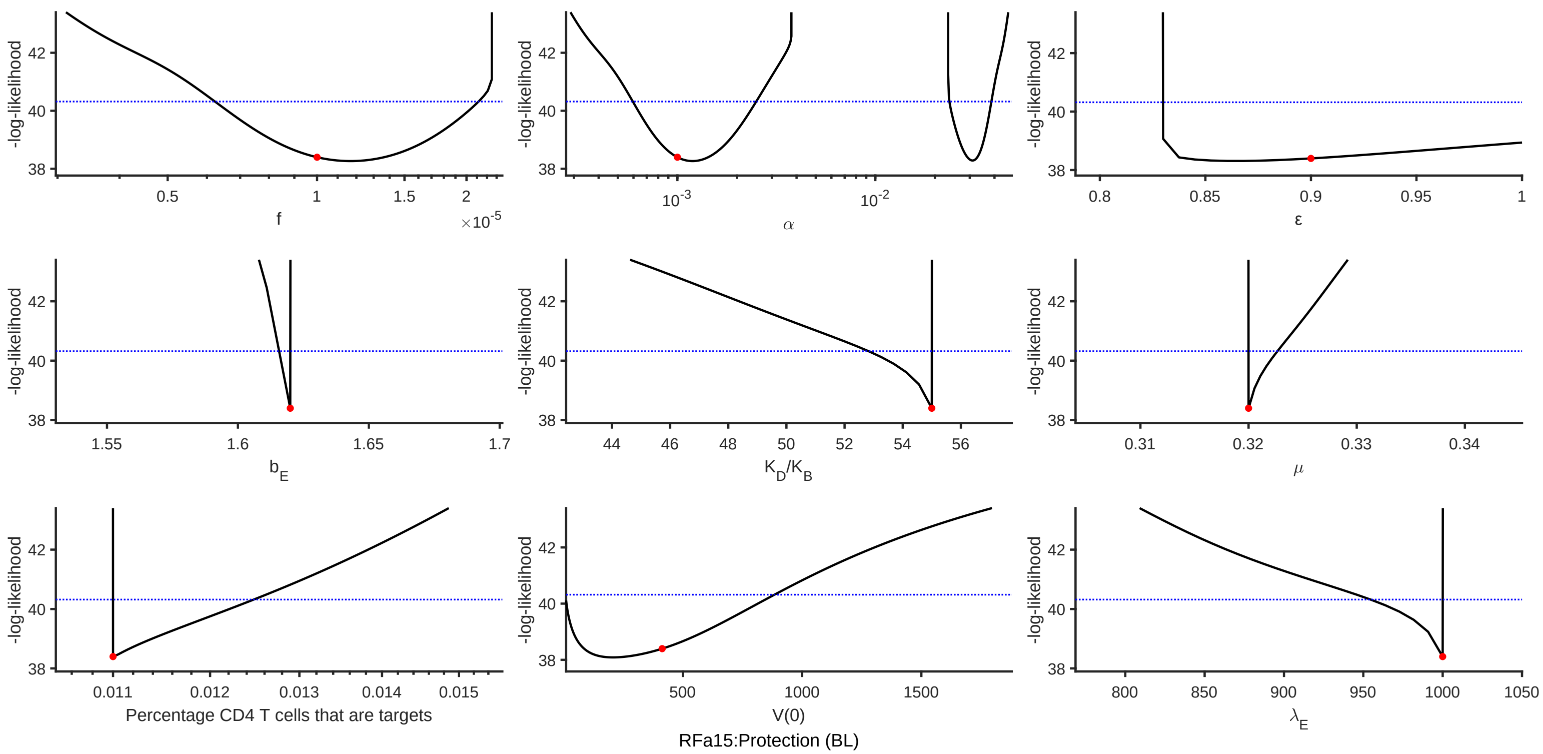

RFa15:Protection (BL)

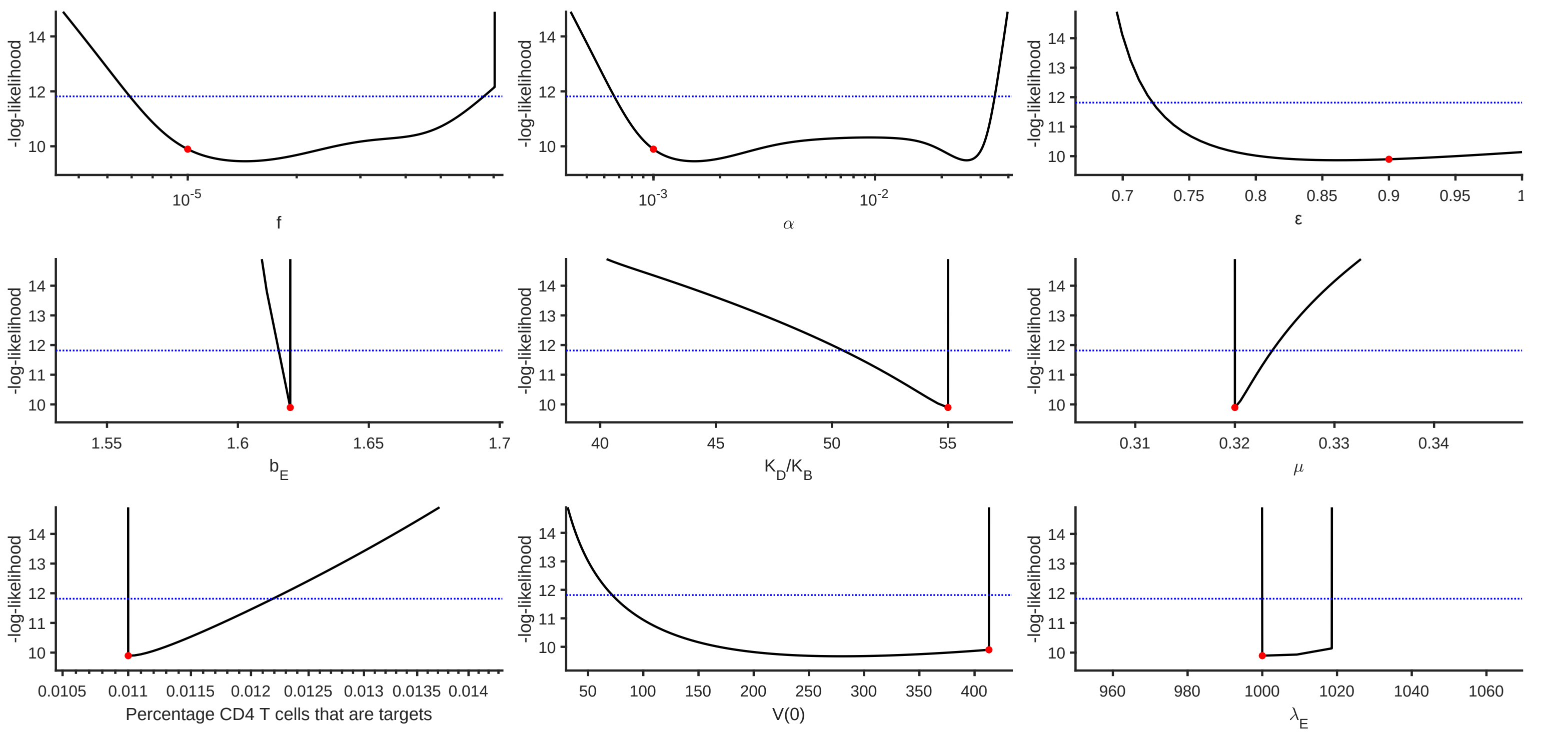

RLn12:Protection (BL)

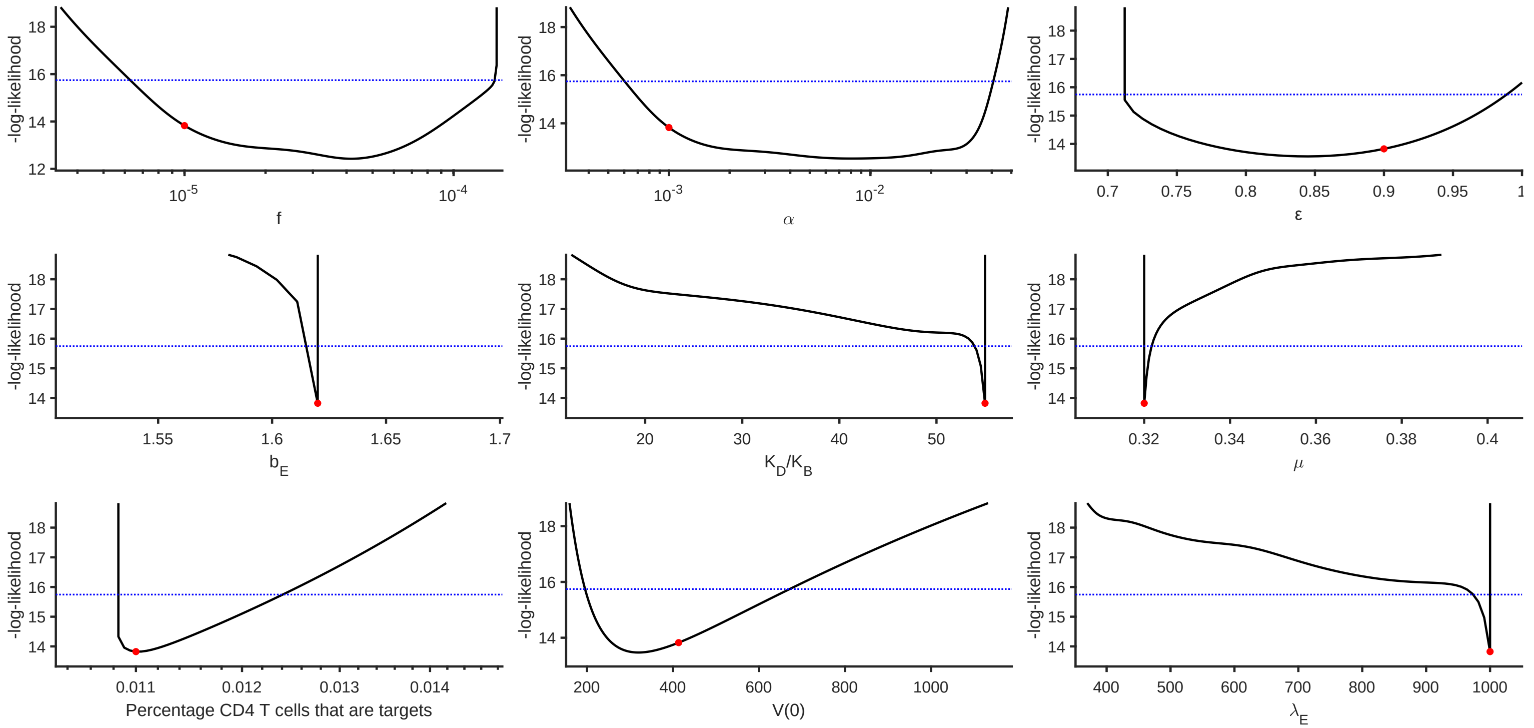

RId14:Protection (BL)

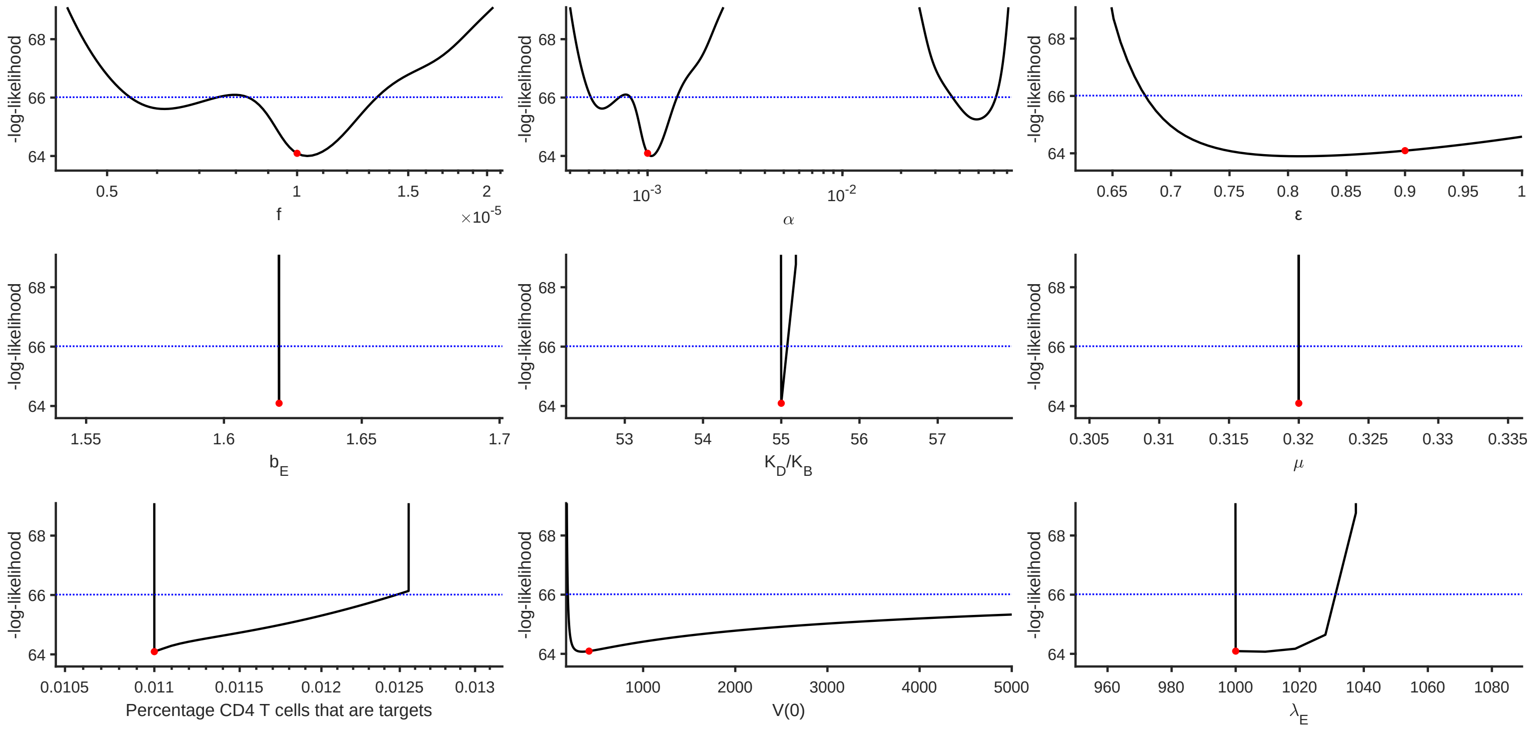

ROo13:Protection (BL)

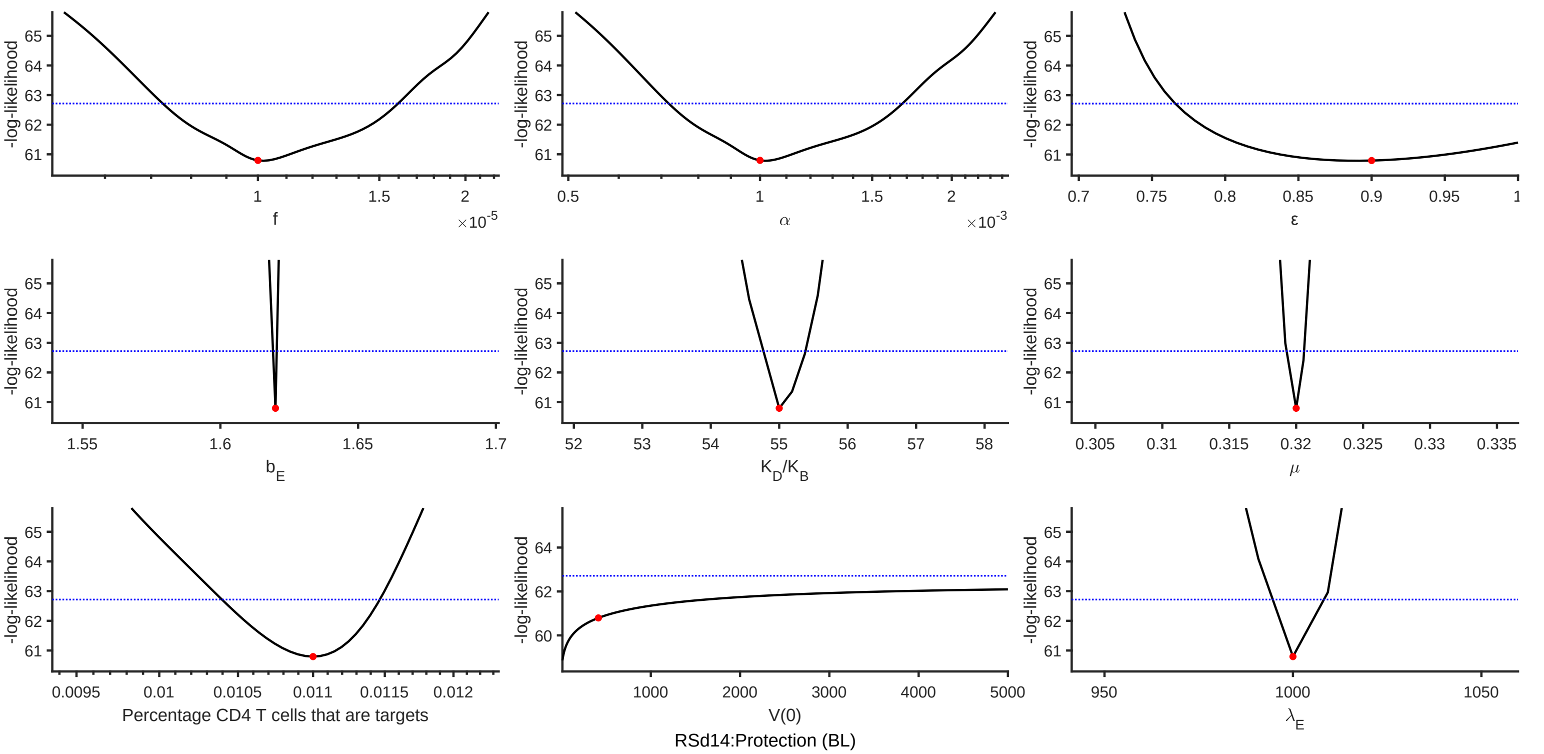

RSd14:Protection (BL)

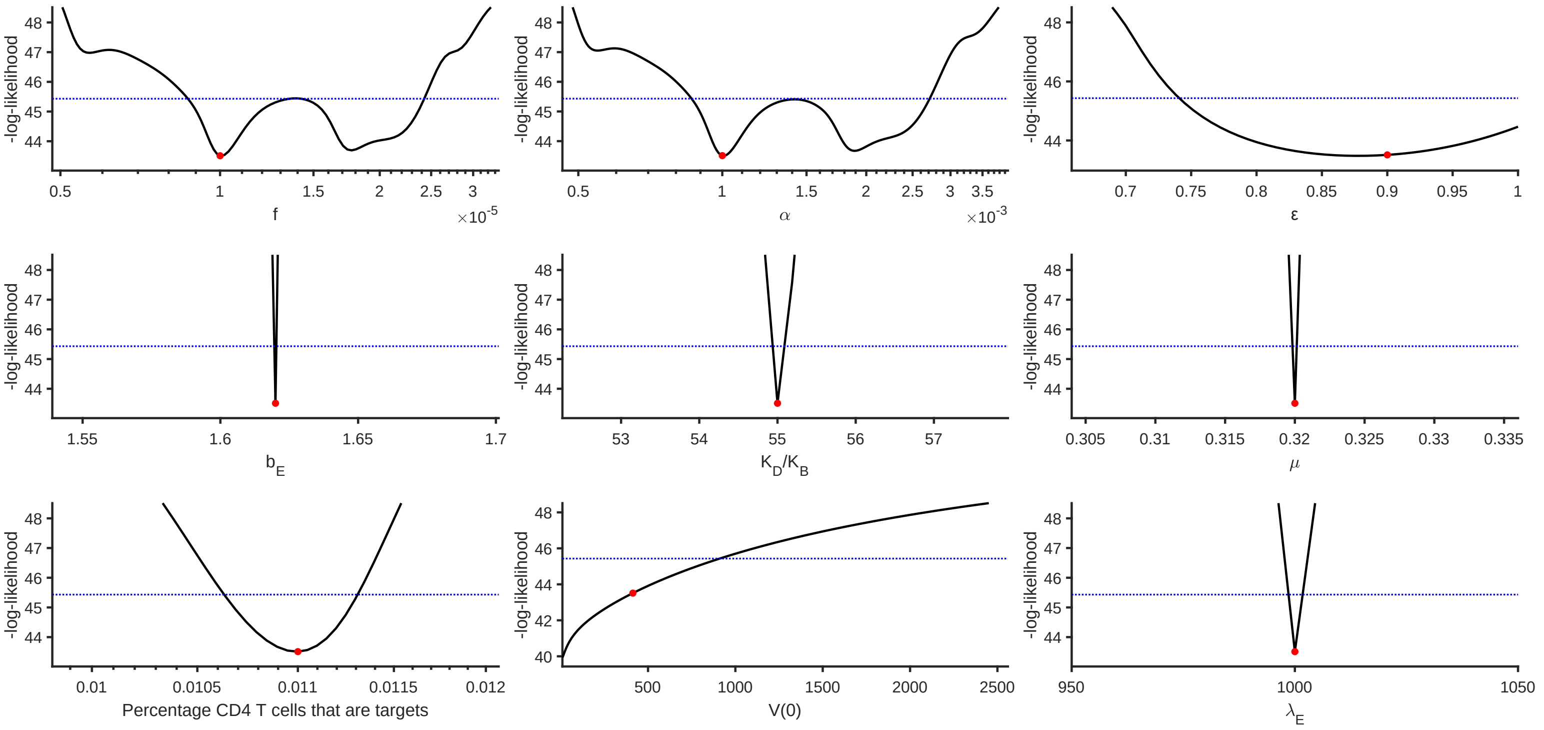

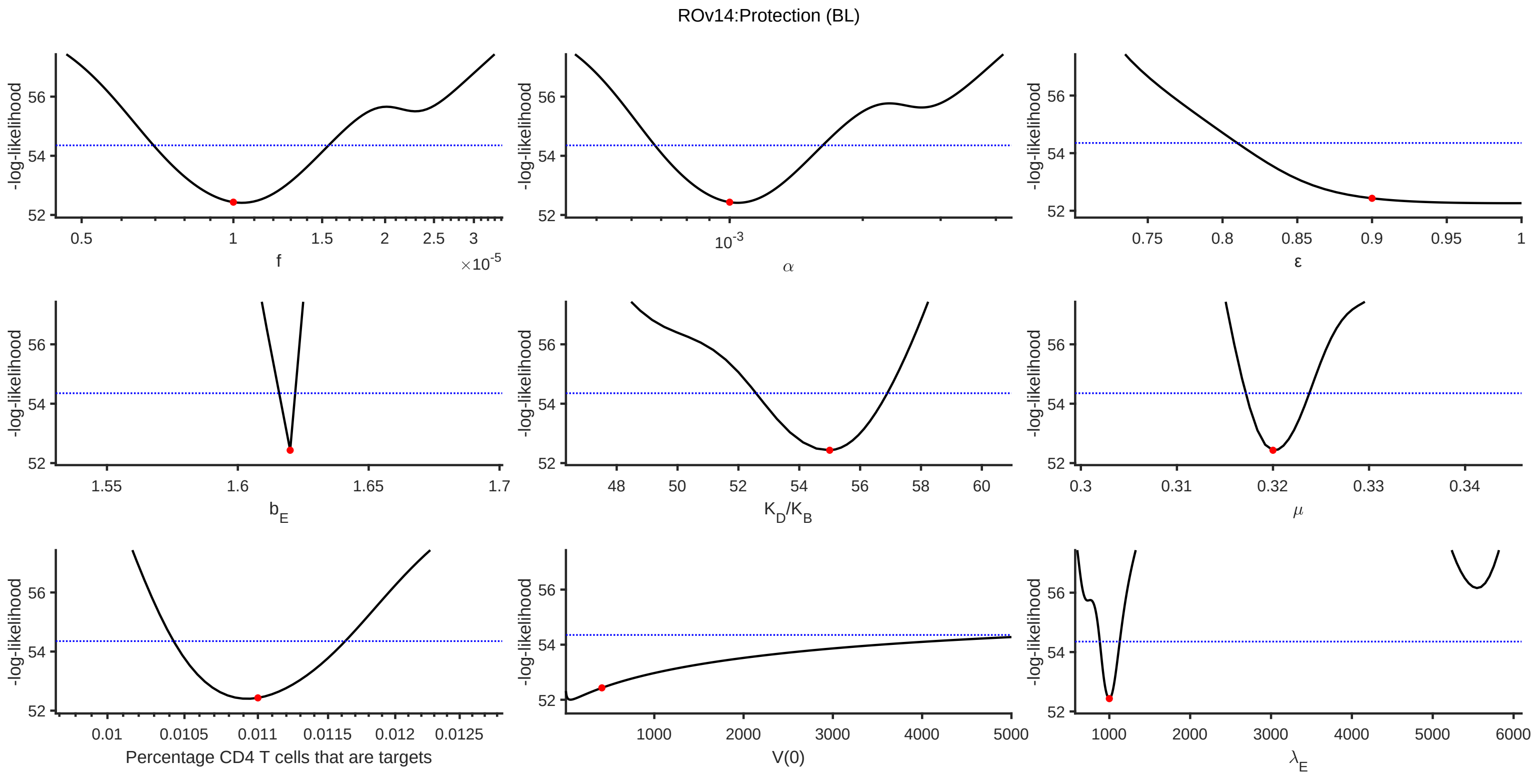

ROv14:Protection (BL)
-log-likelihood
52
54
56
0.5
1
1.5
2
2.5
3
f
×10^-5
10^-3
α
0.75
0.8
0.85
0.9
0.95
1
ε
1.55
1.6
1.65
1.7
b_E
48
50
52
54
56
58
60
K_D/K_B
0.3
0.31
0.32
0.33
0.34
μ
0.01
0.0105
0.011
0.0115
0.012
0.0125
Percentage CD4 T cells that are targets
1000
2000
3000
4000
5000
V(0)
6000
λ_E

8.2 Saturated effector cell source model

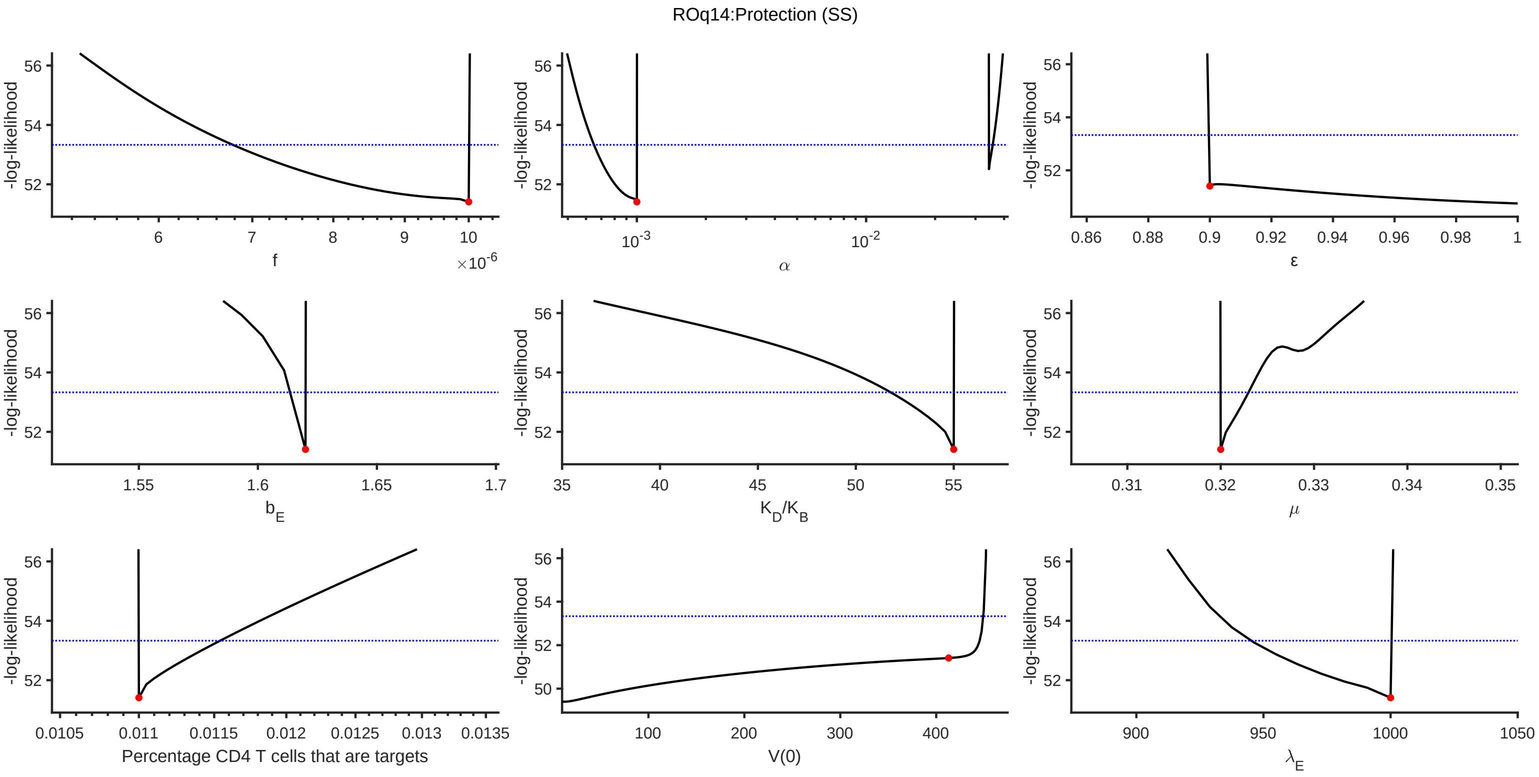

RDa15:Protection (SS)

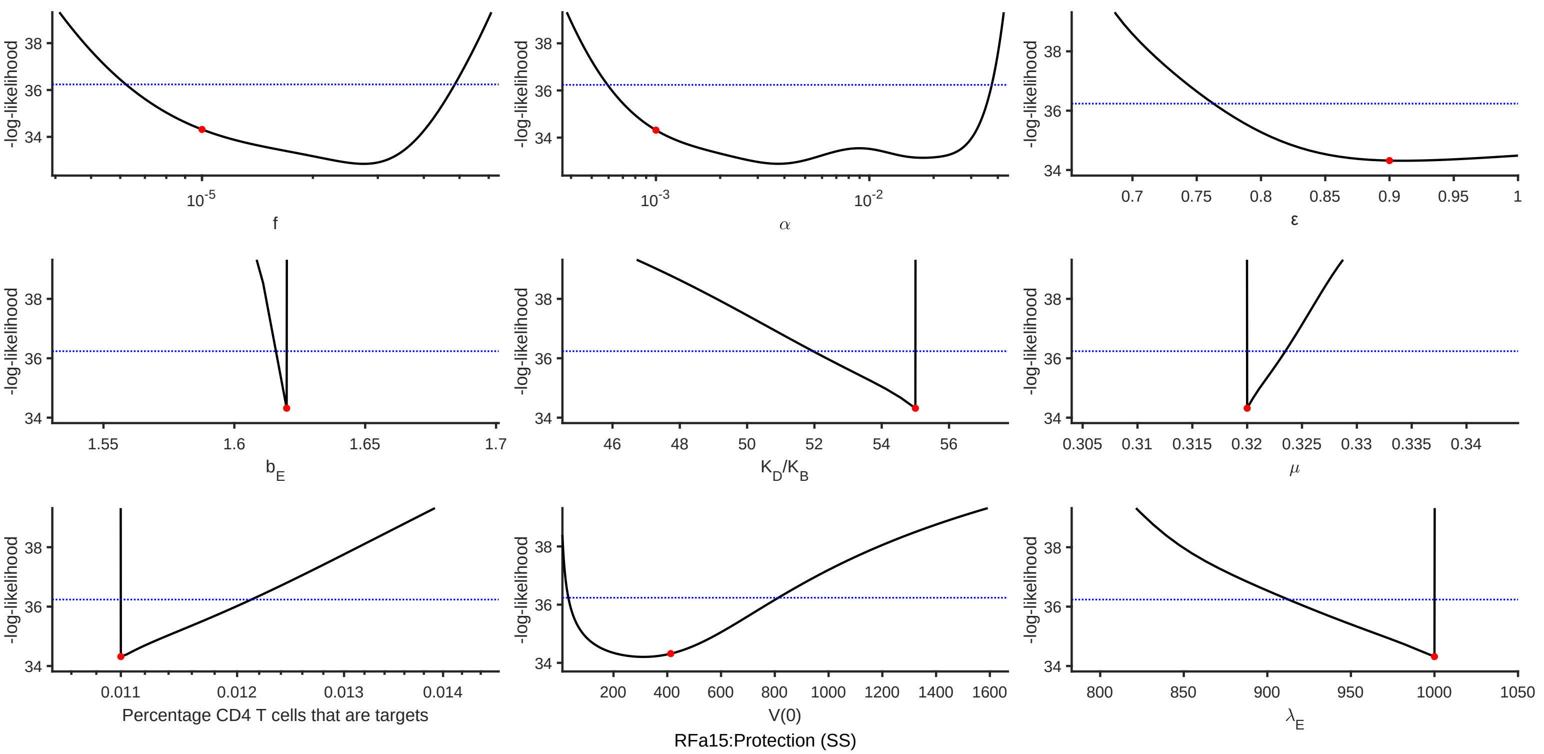

RFa15:Protection (SS)

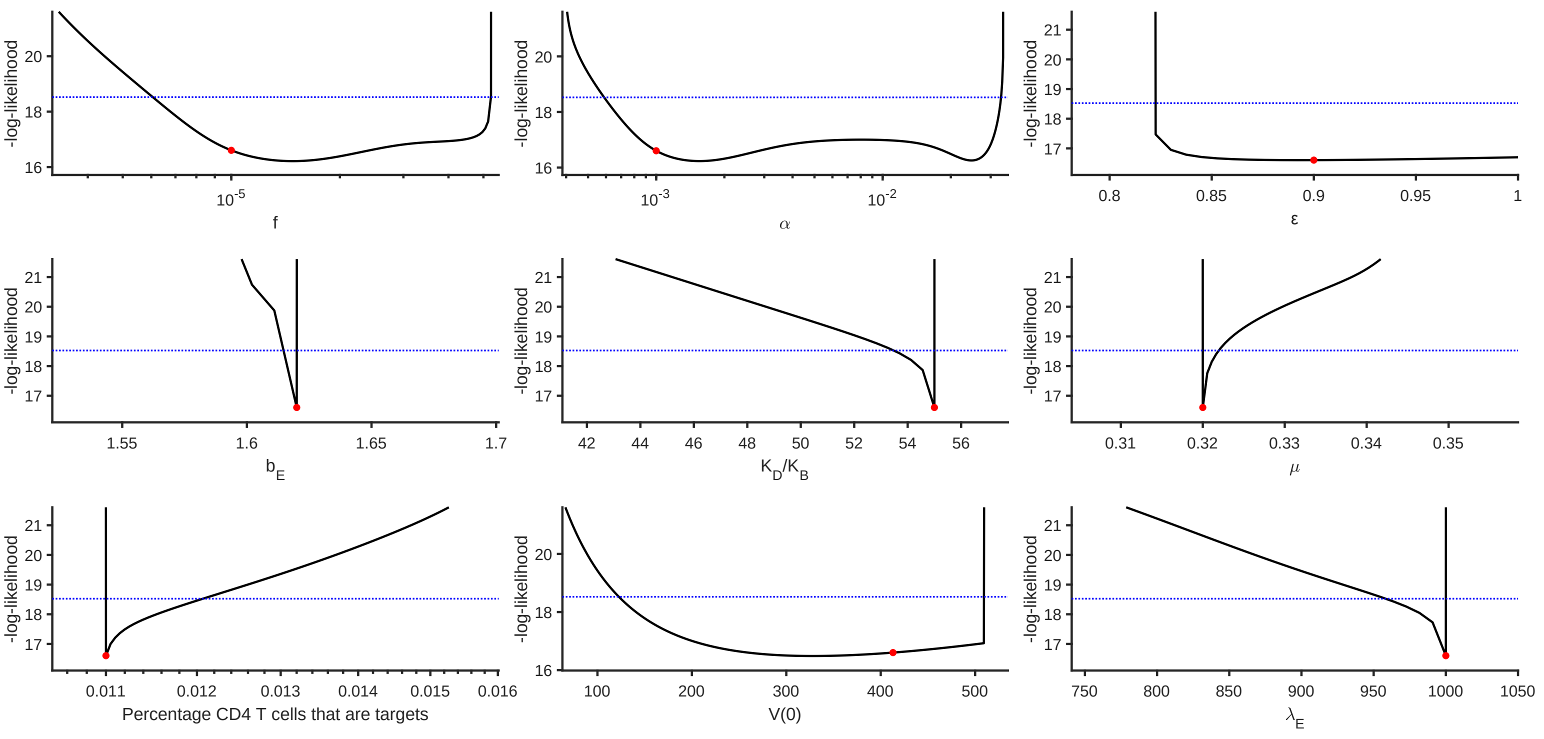

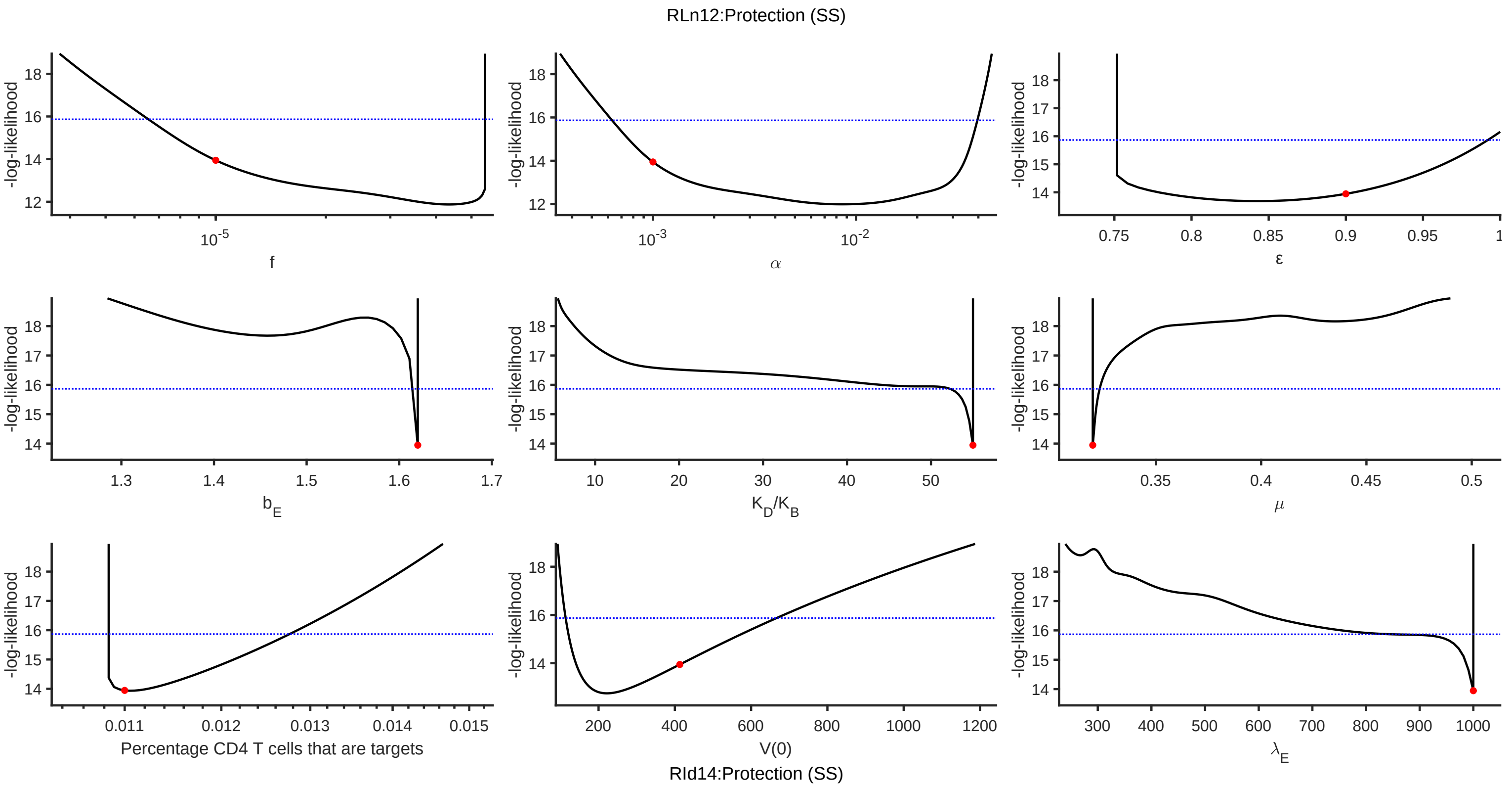
RLn12:Protection (SS)
-log-likelihood
f
α
ε
b_E
K_D/K_B
μ
Percentage CD4 T cells that are targets
V(0)
λ_E

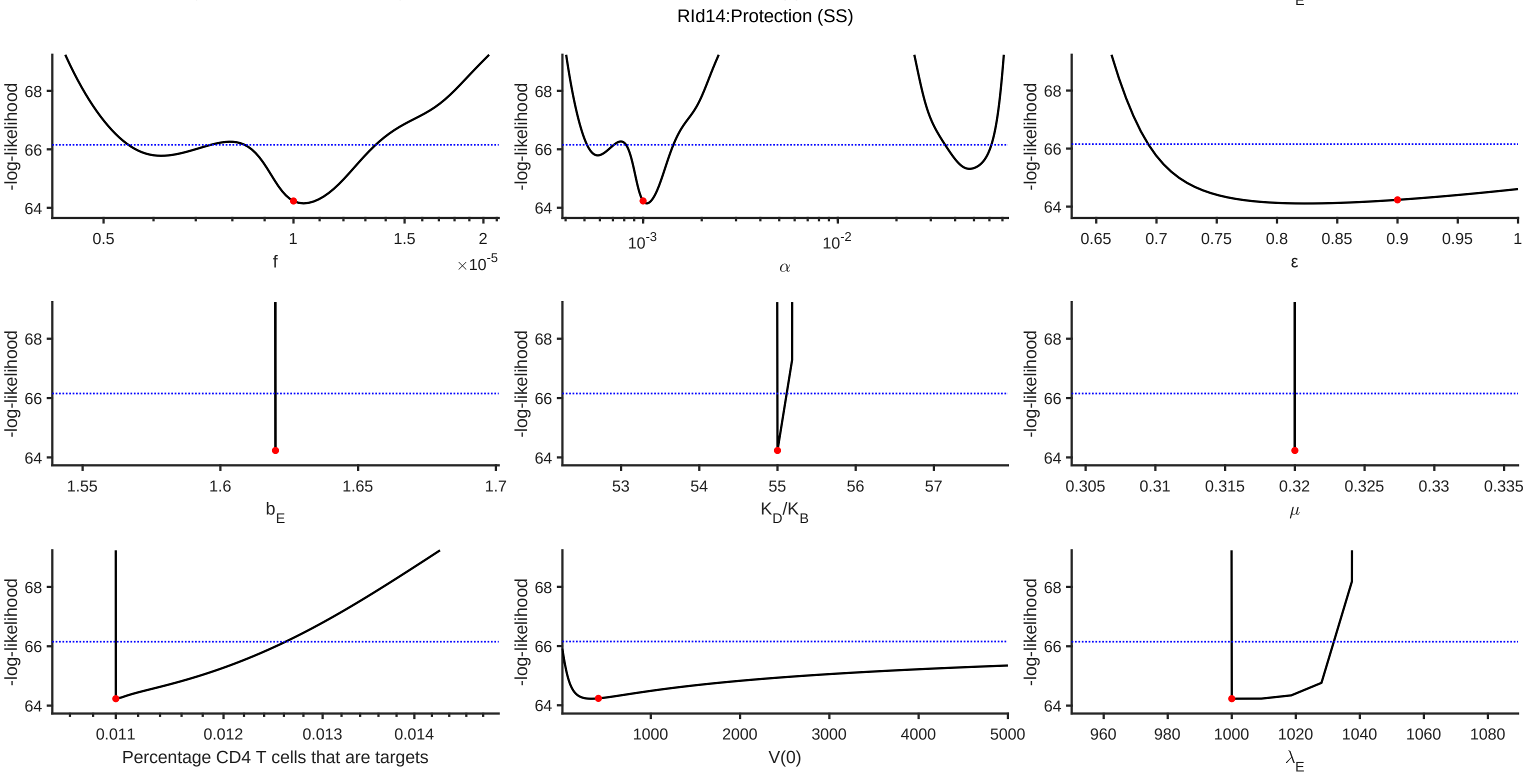
RId14:Protection (SS)
-log-likelihood
f
×10^-5
α
ε
b_E
K_D/K_B
μ
Percentage CD4 T cells that are targets
V(0)
λ_E

ROo13:Protection (SS)

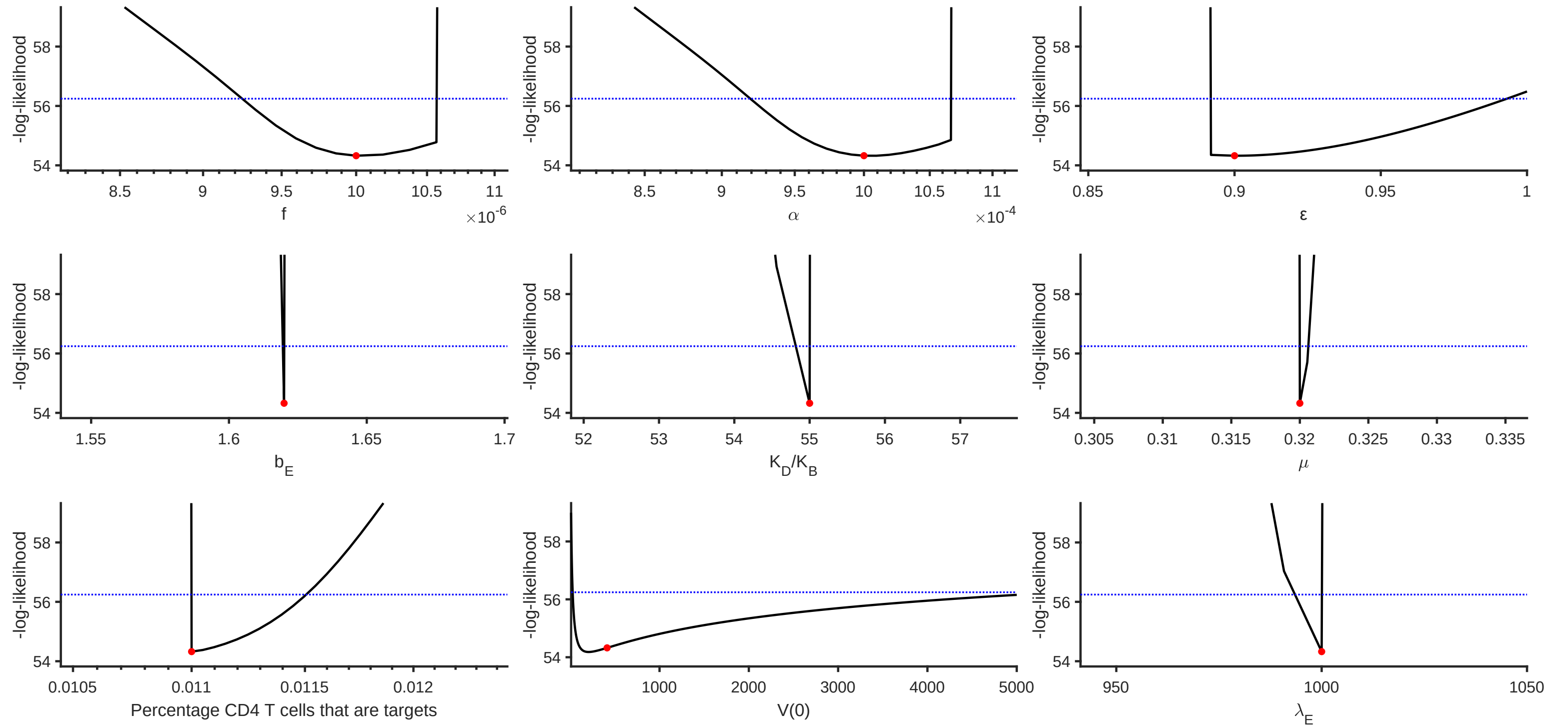

RSd14:Protection (SS)

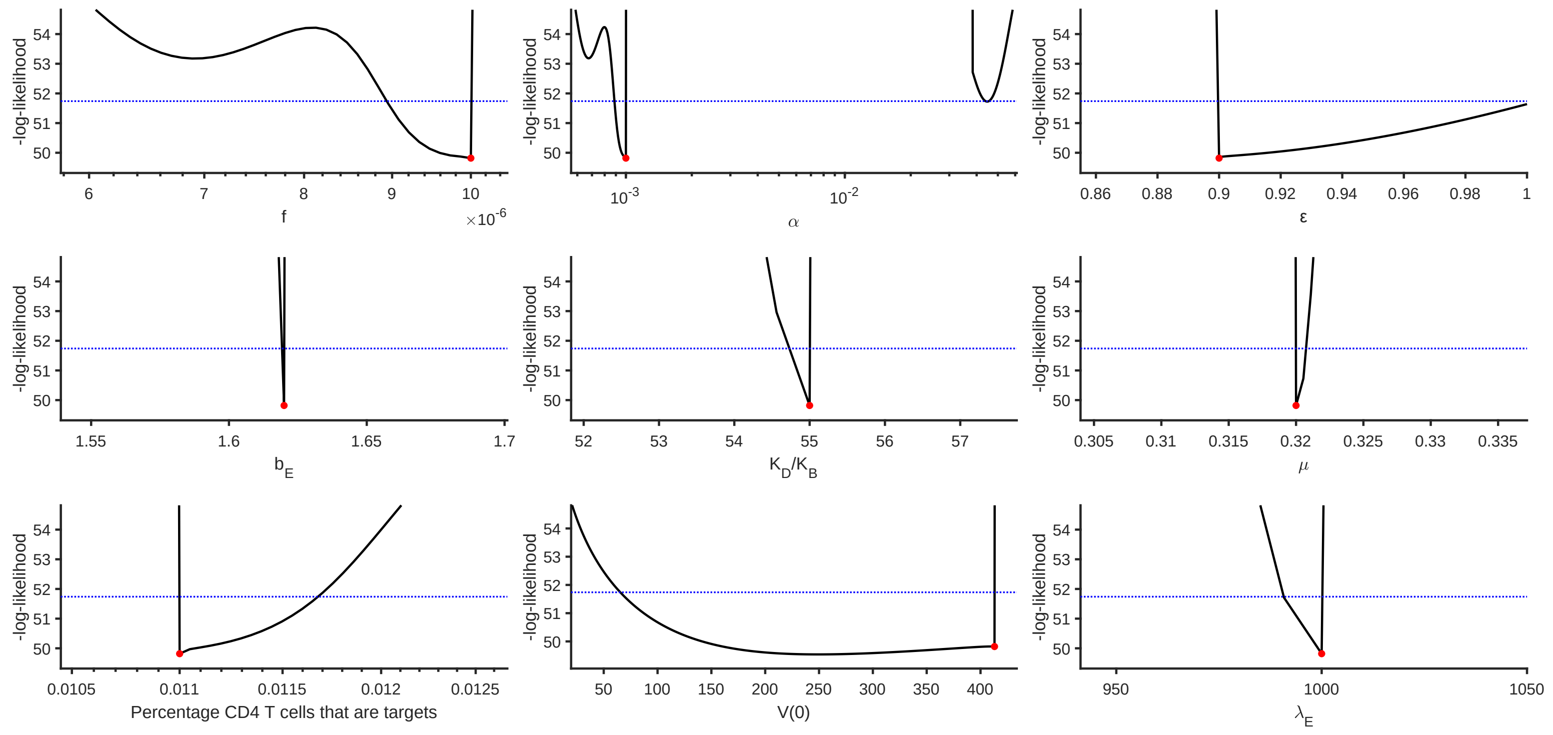

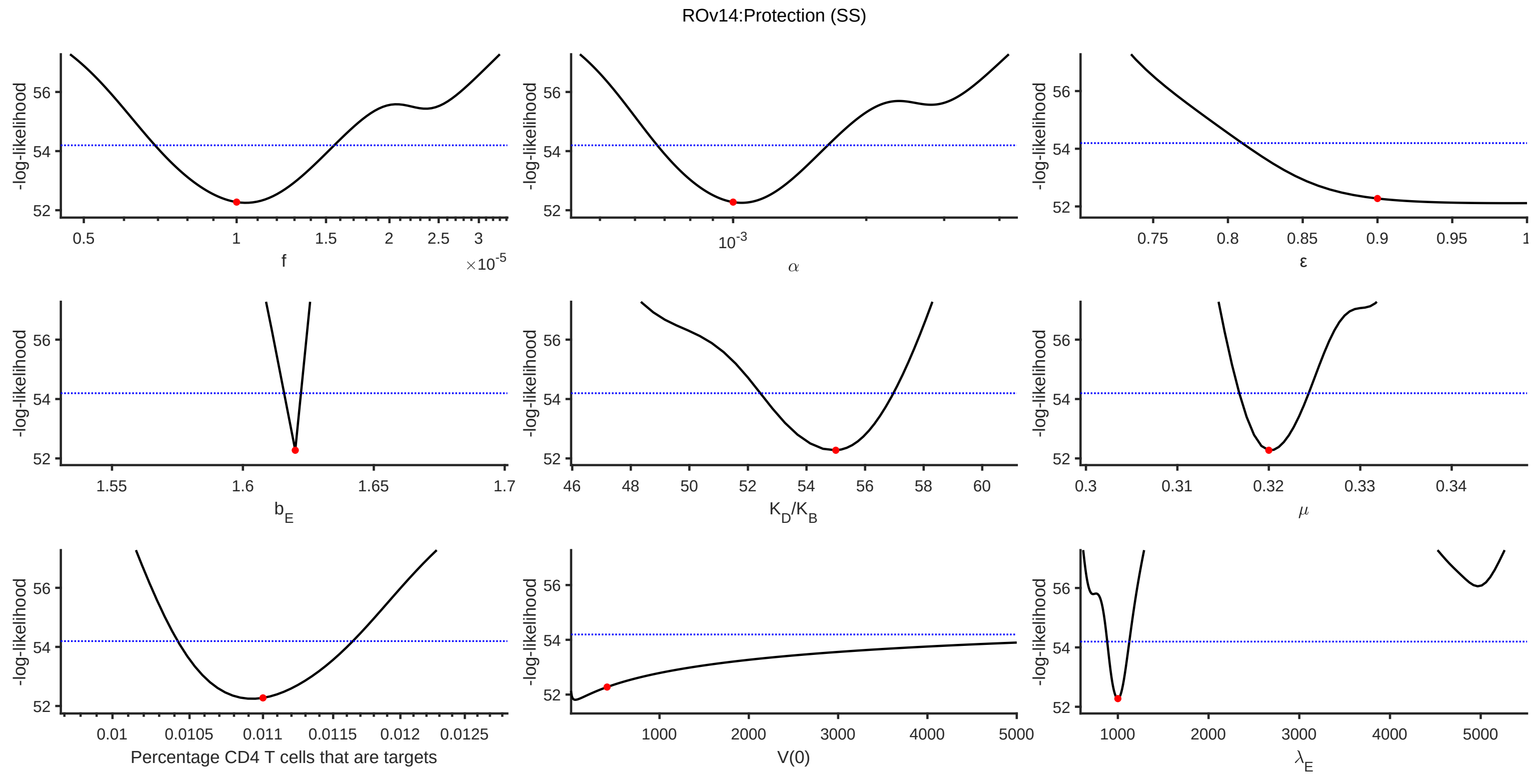
ROv14:Protection (SS)
-log-likelihood
f
×10-5
α
10-3
ε
b_E
K_D/K_B
μ
Percentage CD4 T cells that are targets
V(0)
λ_E

8.3 Antigen presenting cell effector cell source model

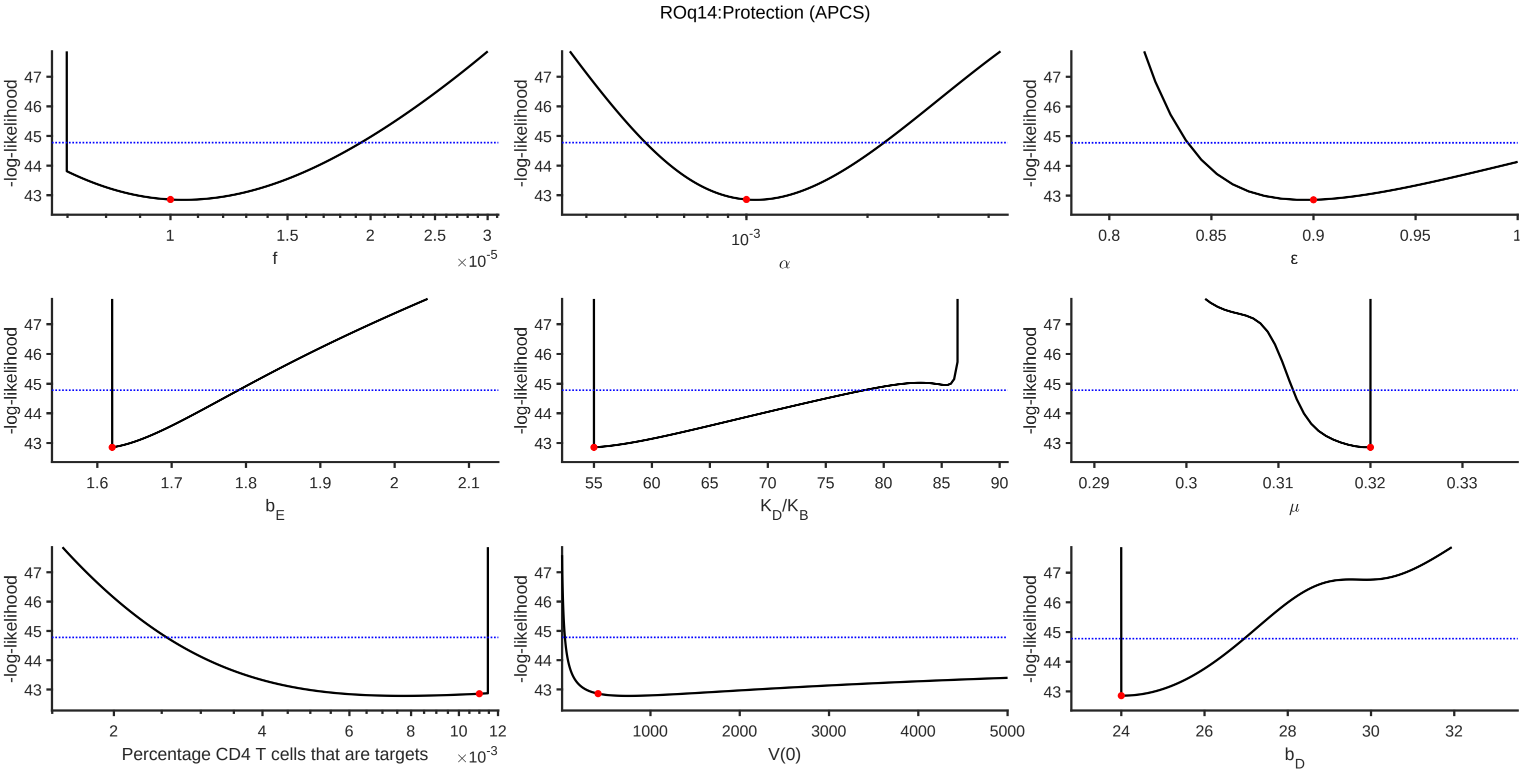

RDa15:Protection (APCS)

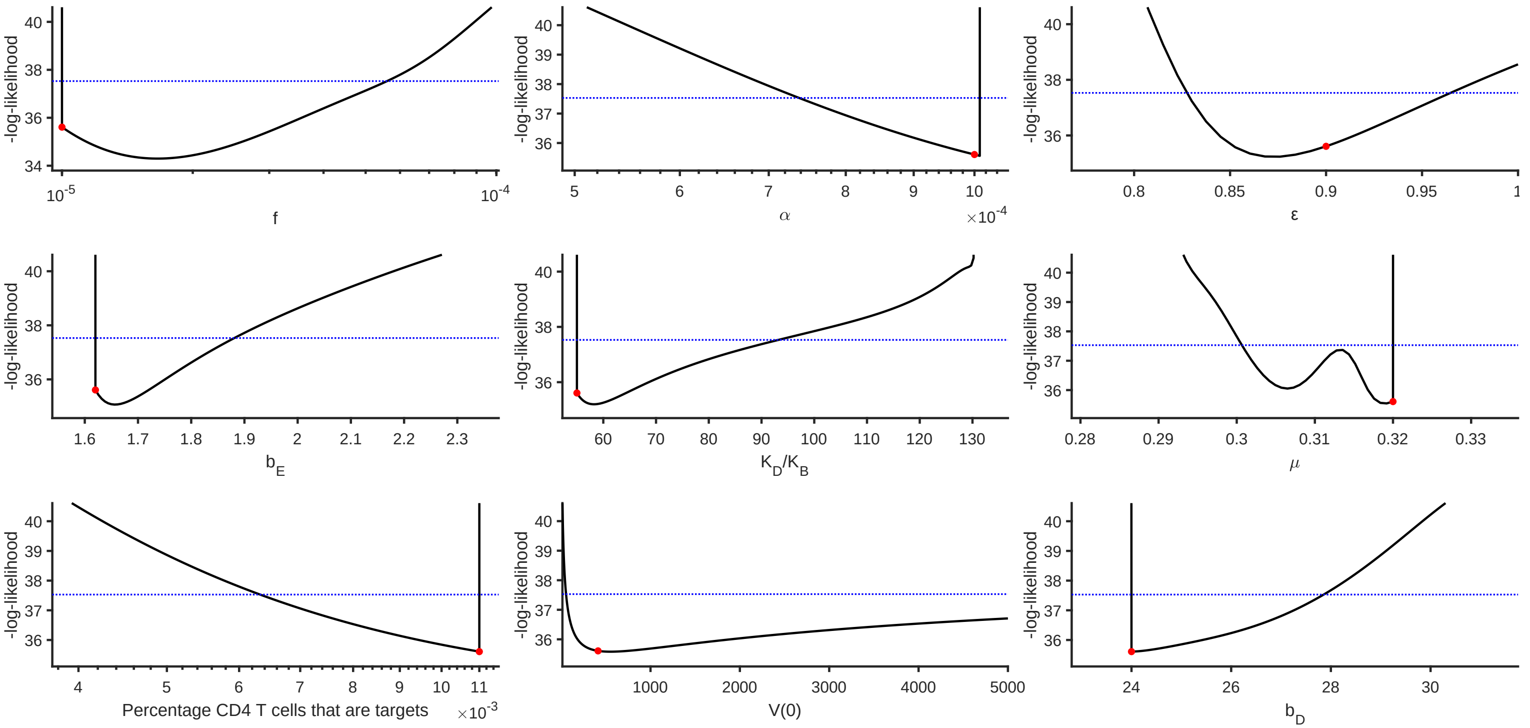

RFa15:Protection (APCS)

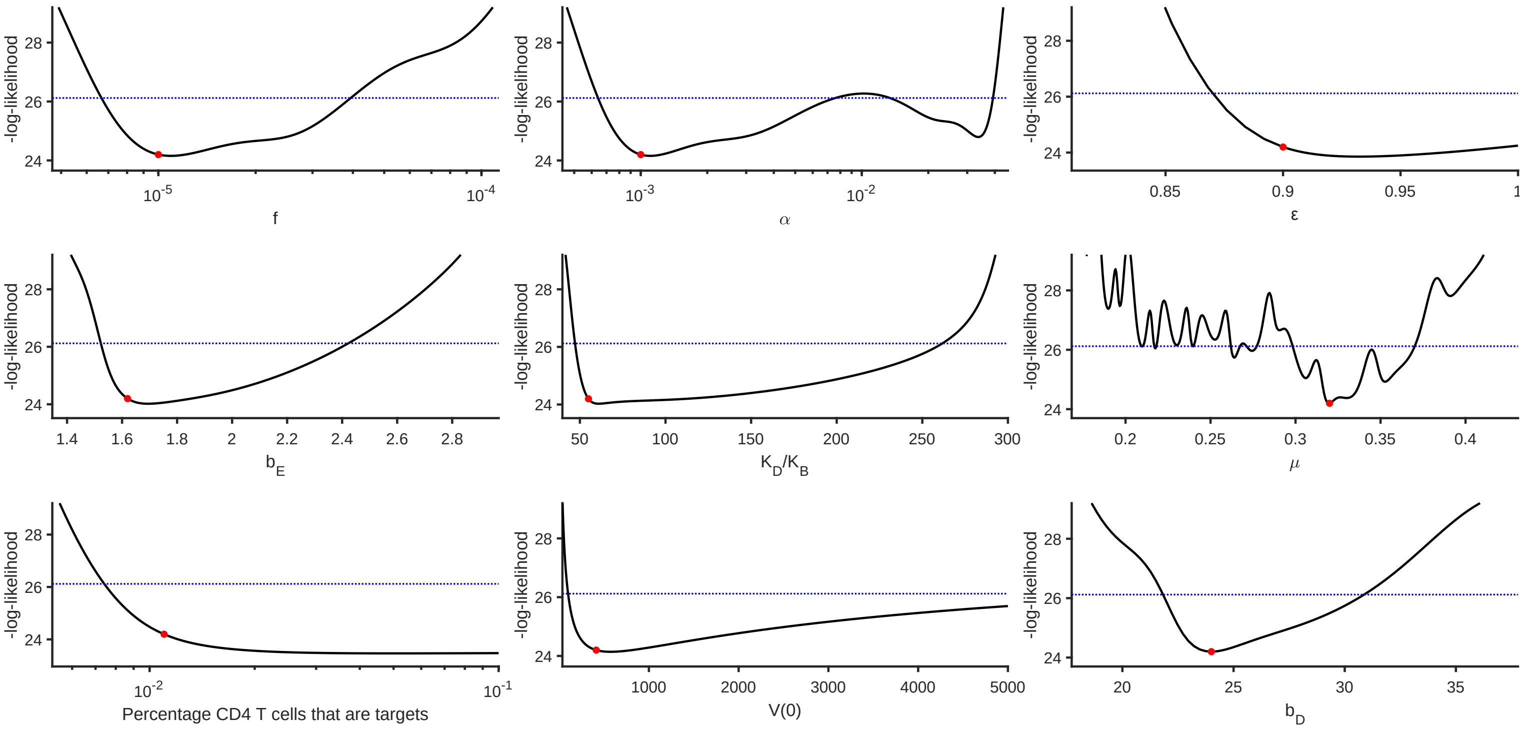

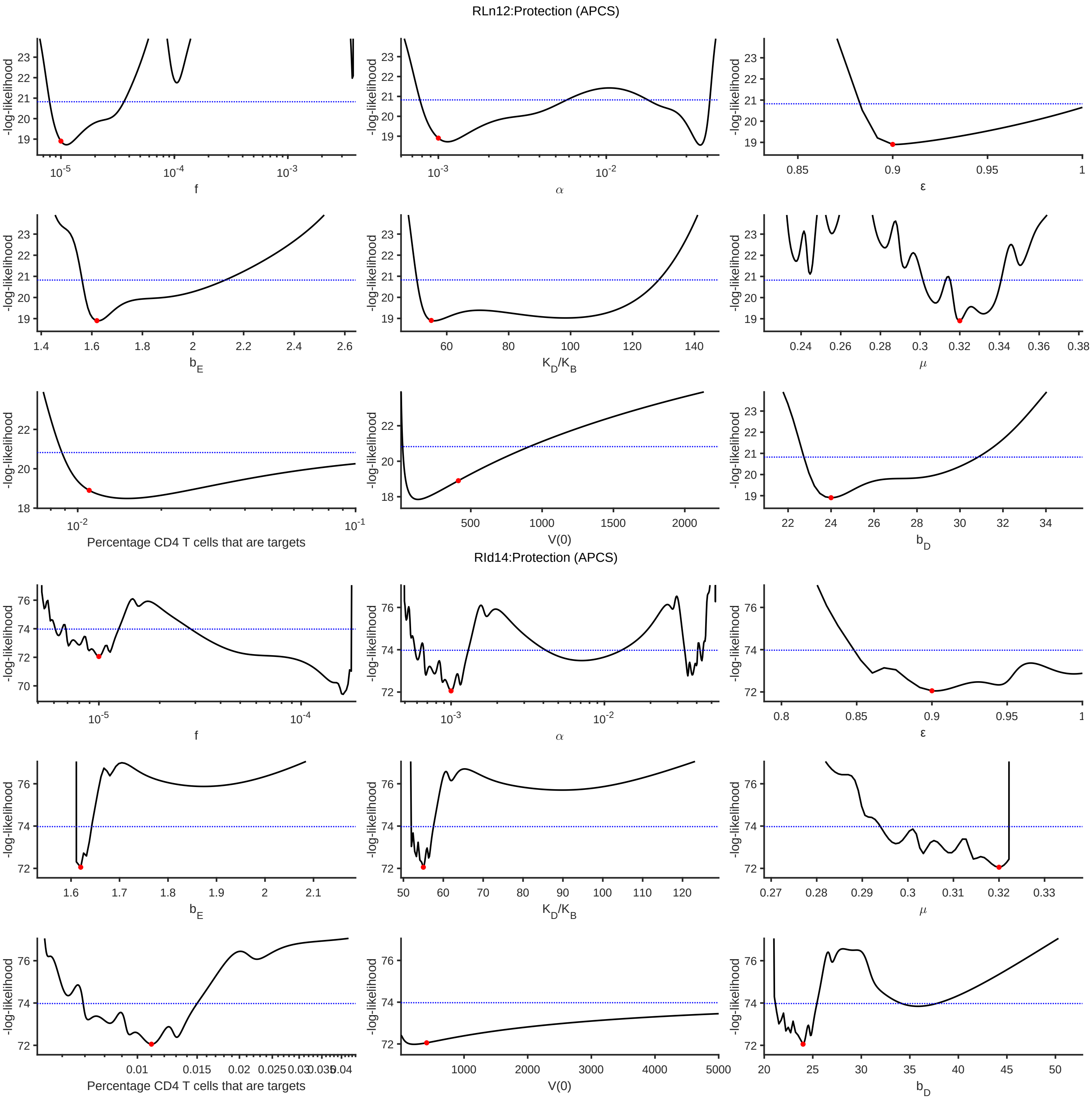
RLn12:Protection (APCS)
-log-likelihood
f
α
ε
b_E
K_D/K_B
μ
Percentage CD4 T cells that are targets
V(0)
b_D
RId14:Protection (APCS)
-log-likelihood
f
α
ε
b_E
K_D/K_B
μ
Percentage CD4 T cells that are targets
V(0)
b_D

ROo13:Protection (APCS)

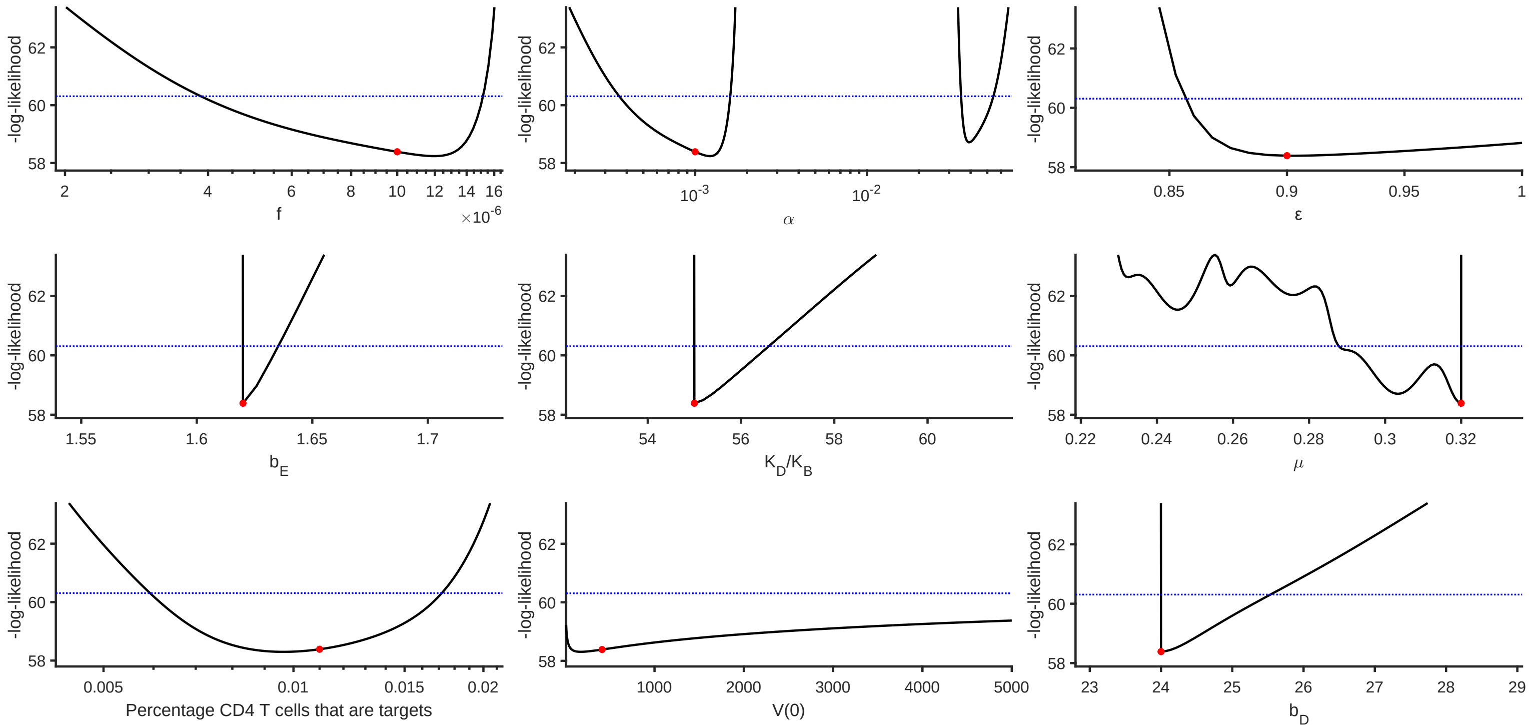

RSd14:Protection (APCS)

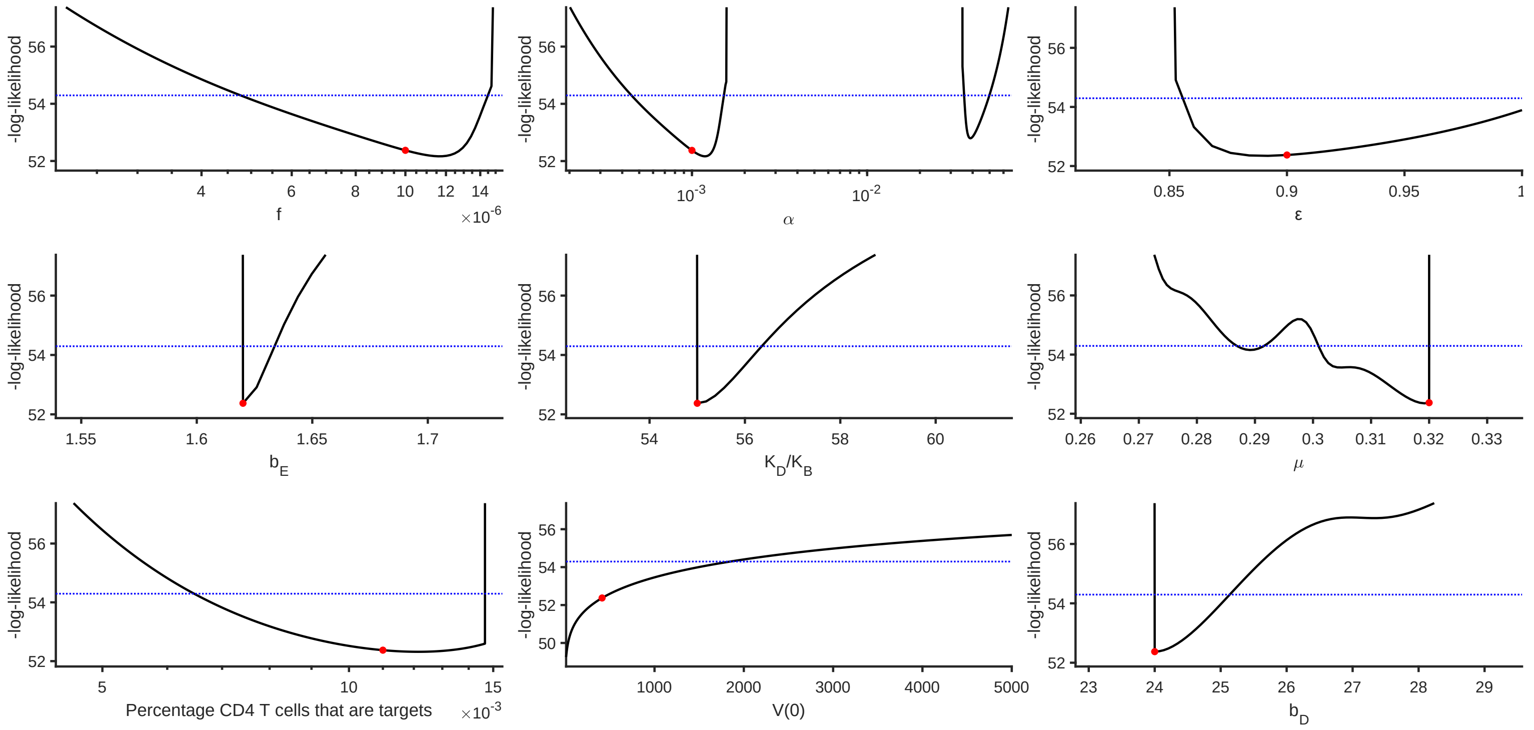

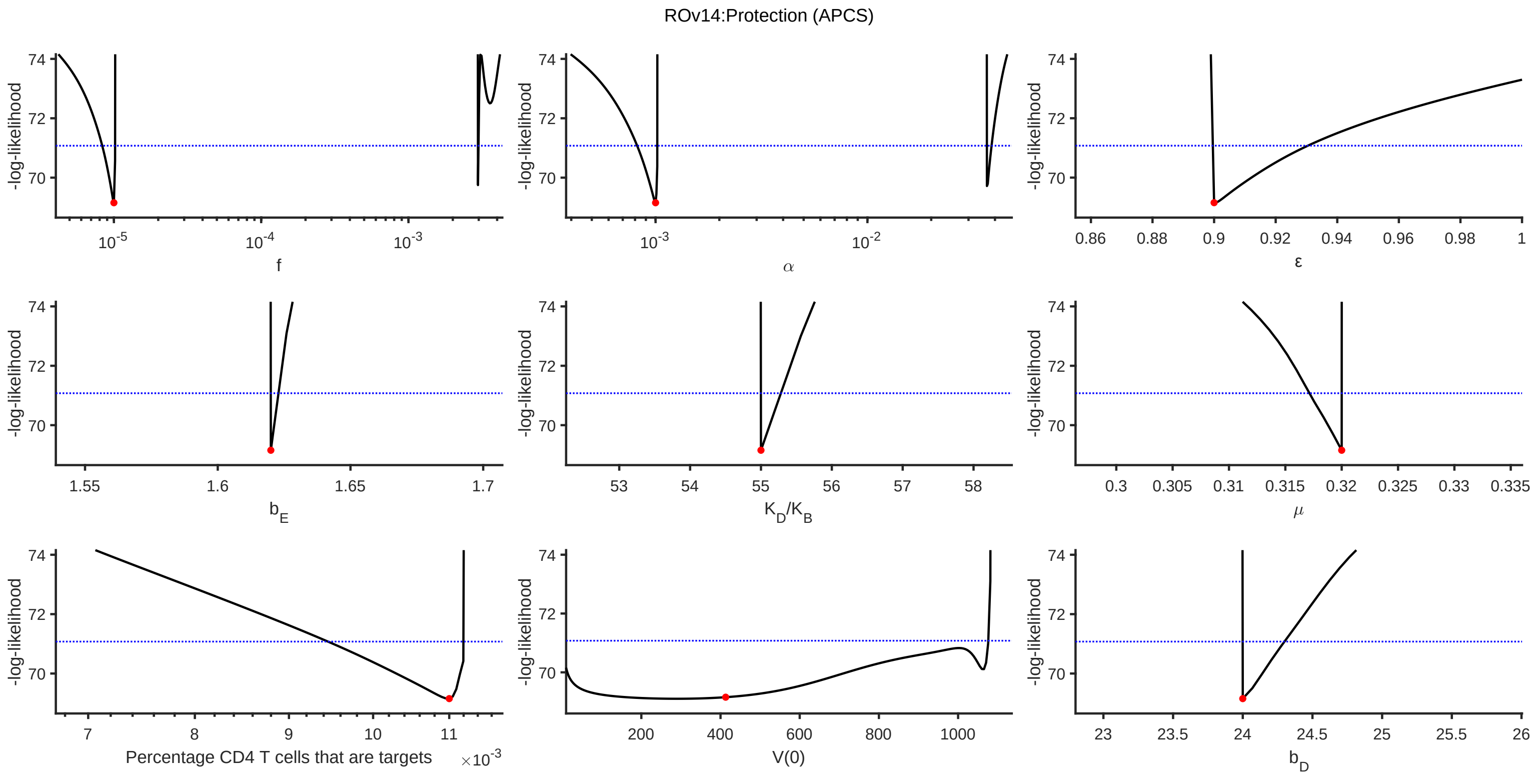
ROv14:Protection (APCS)
-log-likelihood
f
α
ε
b_E
K_D/K_B
μ
Percentage CD4 T cells that are targets
$\times 10^{-3}$
V(0)
b_D

9 Likelihood profiles: Increased antigen presentation

9.1 Baseline effector cell source model

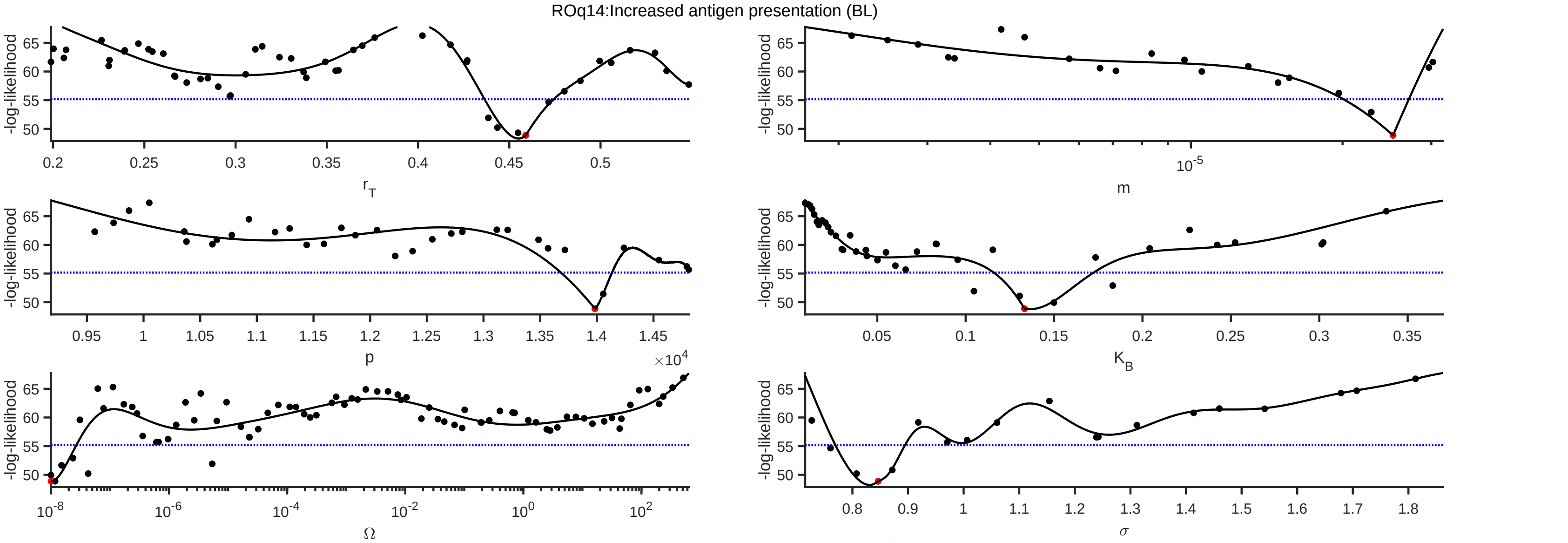

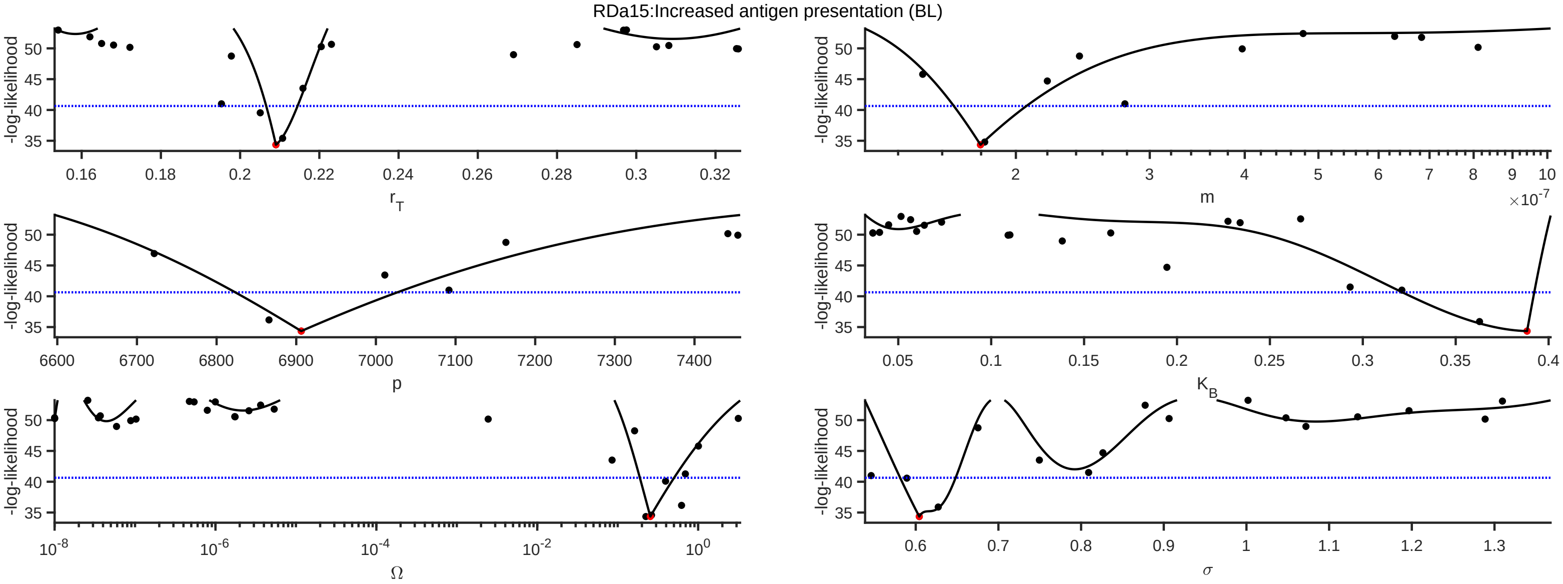

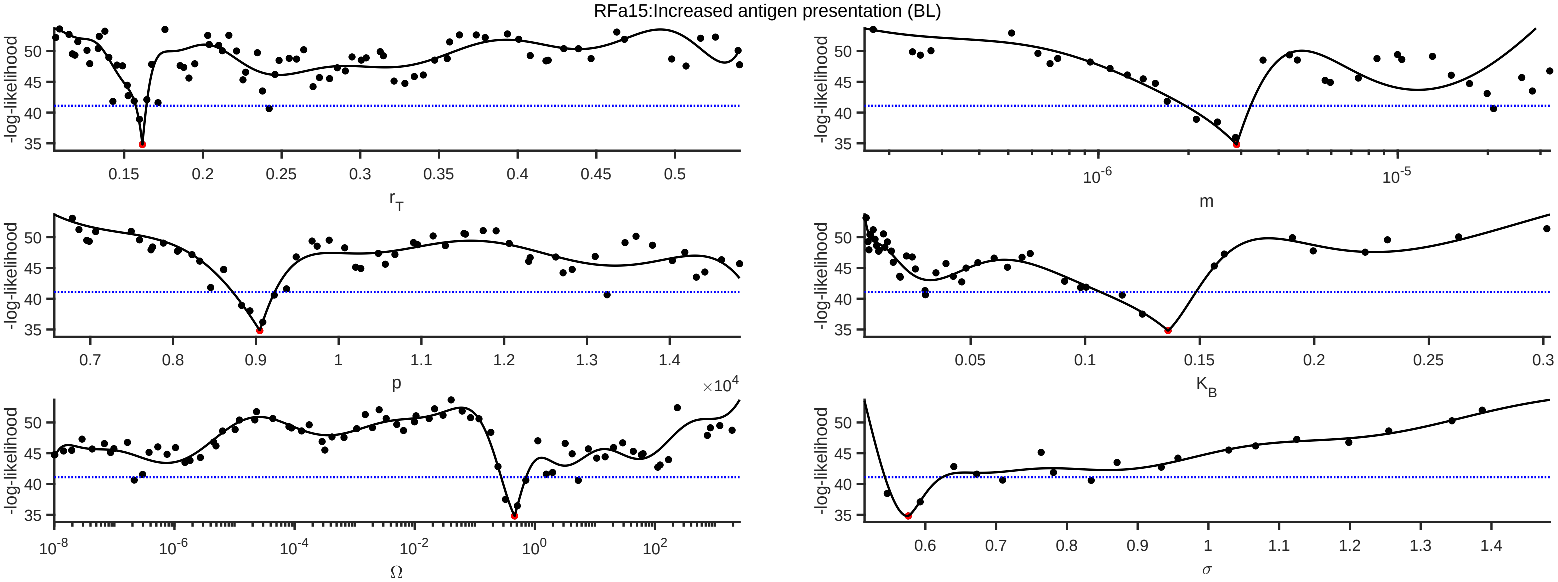
RFa15:Increased antigen presentation (BL)
-log-likelihood
r_T
m
p
K_B
Ω
σ
×10^4

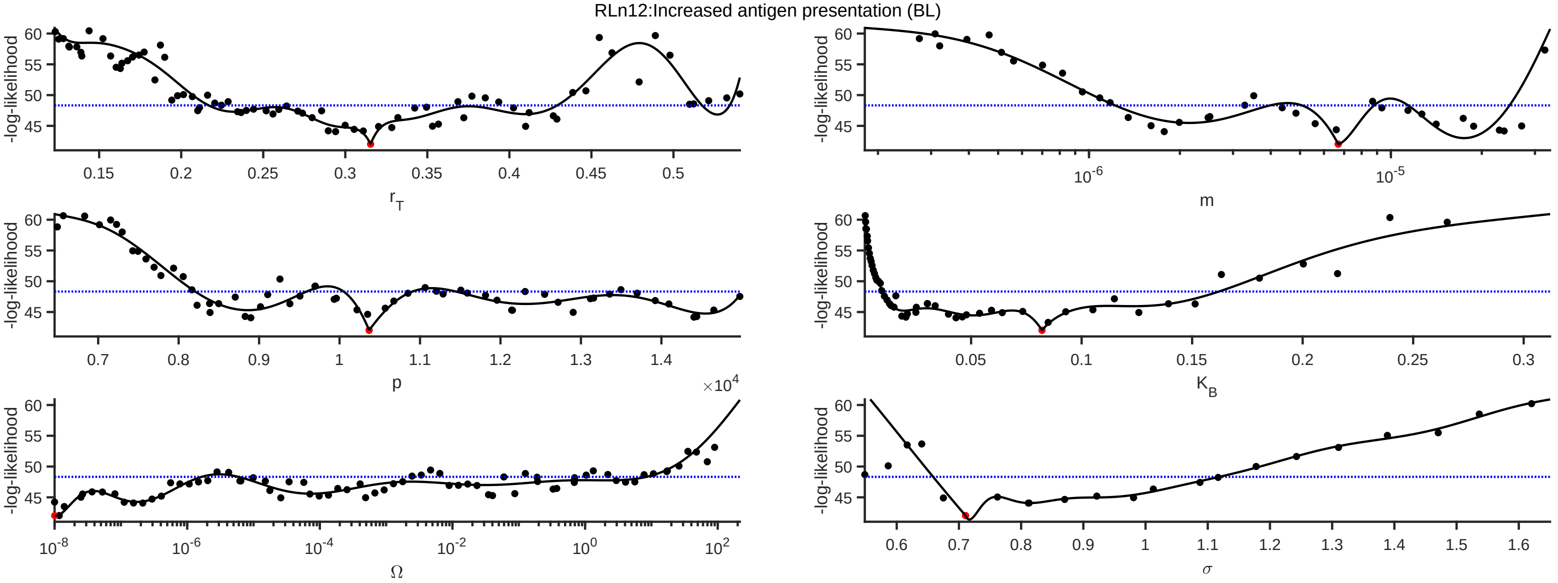
RLn12:Increased antigen presentation (BL)
-log-likelihood
r_T
m
p
K_B
Ω
σ
×10^4

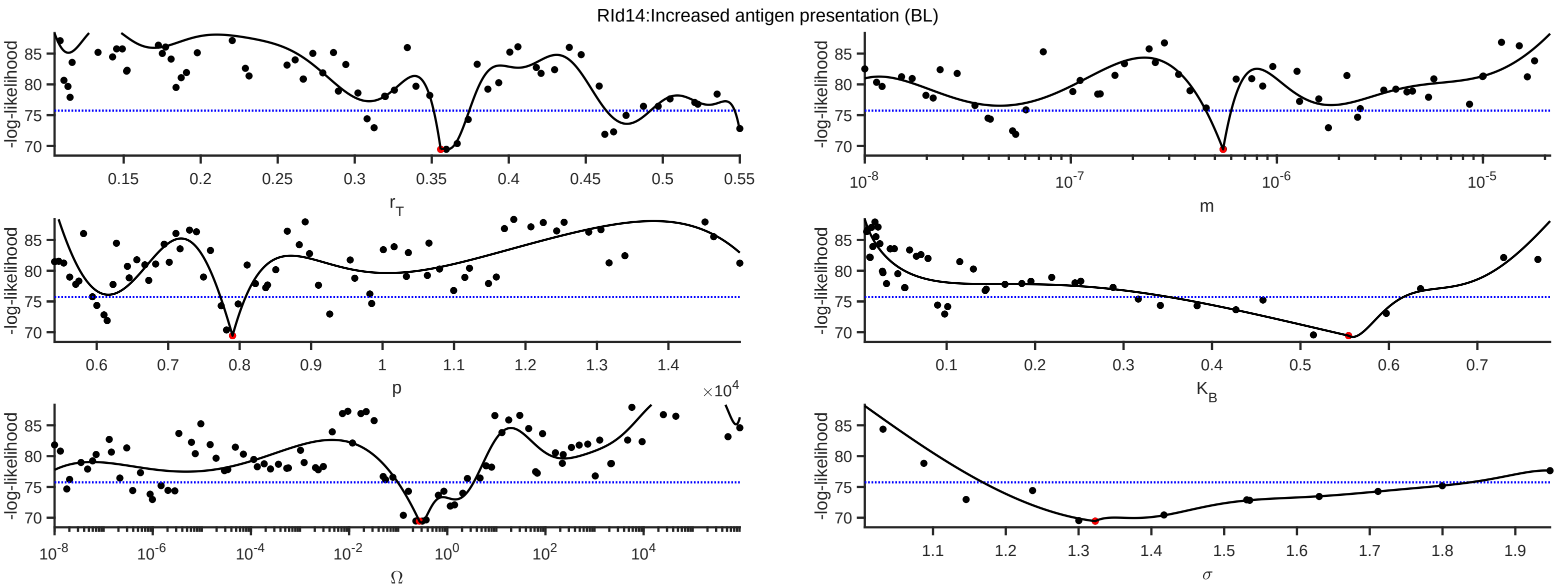
RId14:Increased antigen presentation (BL)
-log-likelihood
r_T
m
p
K_B
Ω
σ

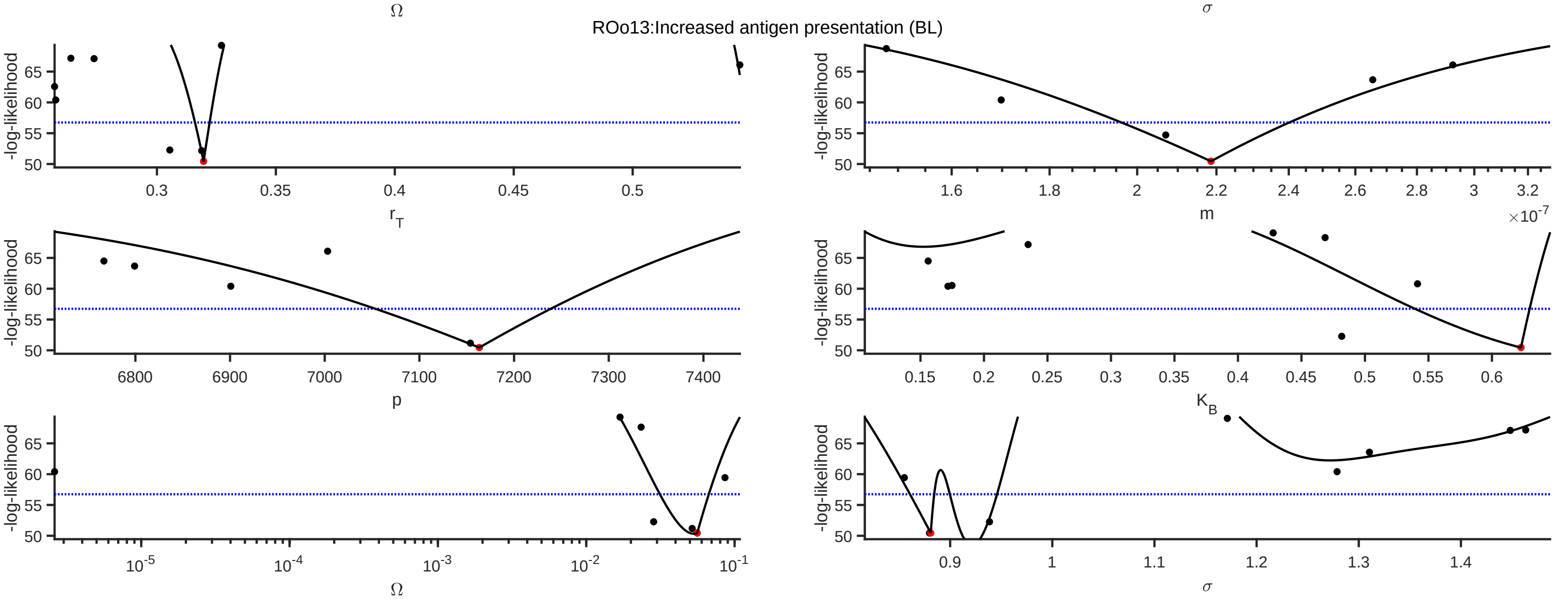
ROo13:Increased antigen presentation (BL)
-log-likelihood
r_T
m
p
K_B
Ω
σ

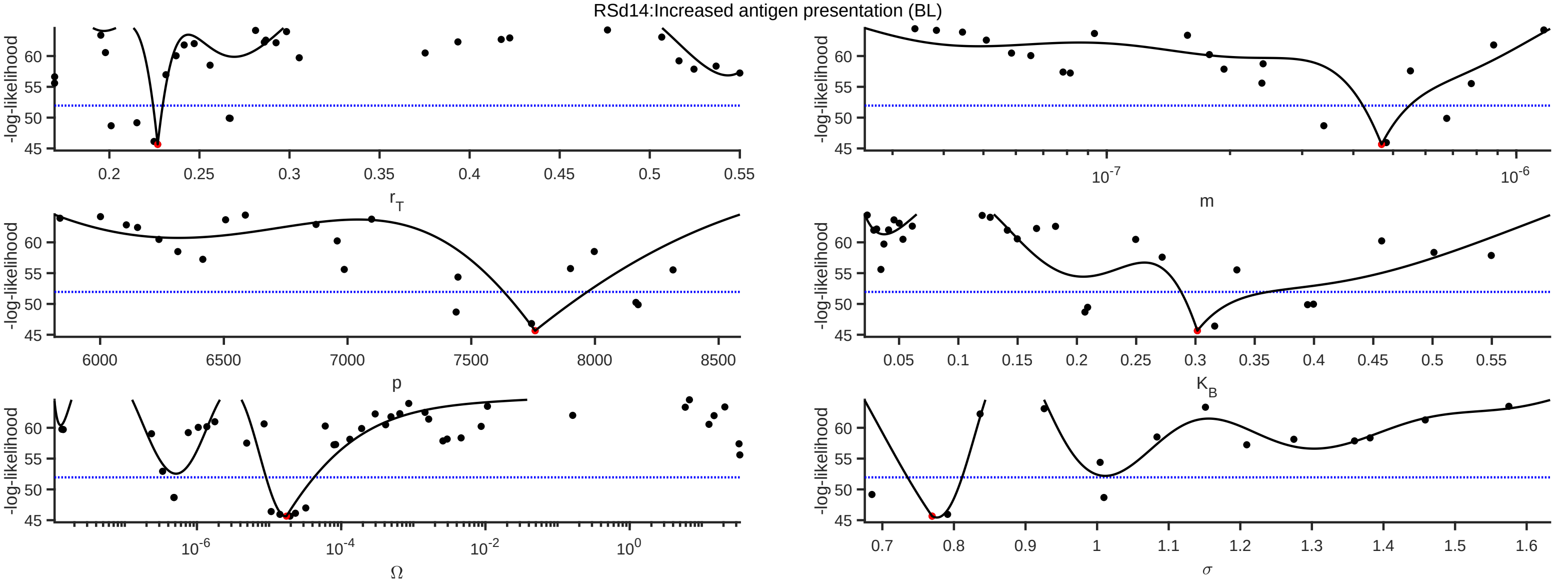
RSd14:Increased antigen presentation (BL)
-log-likelihood
r_T
m
p
K_B
Ω
σ

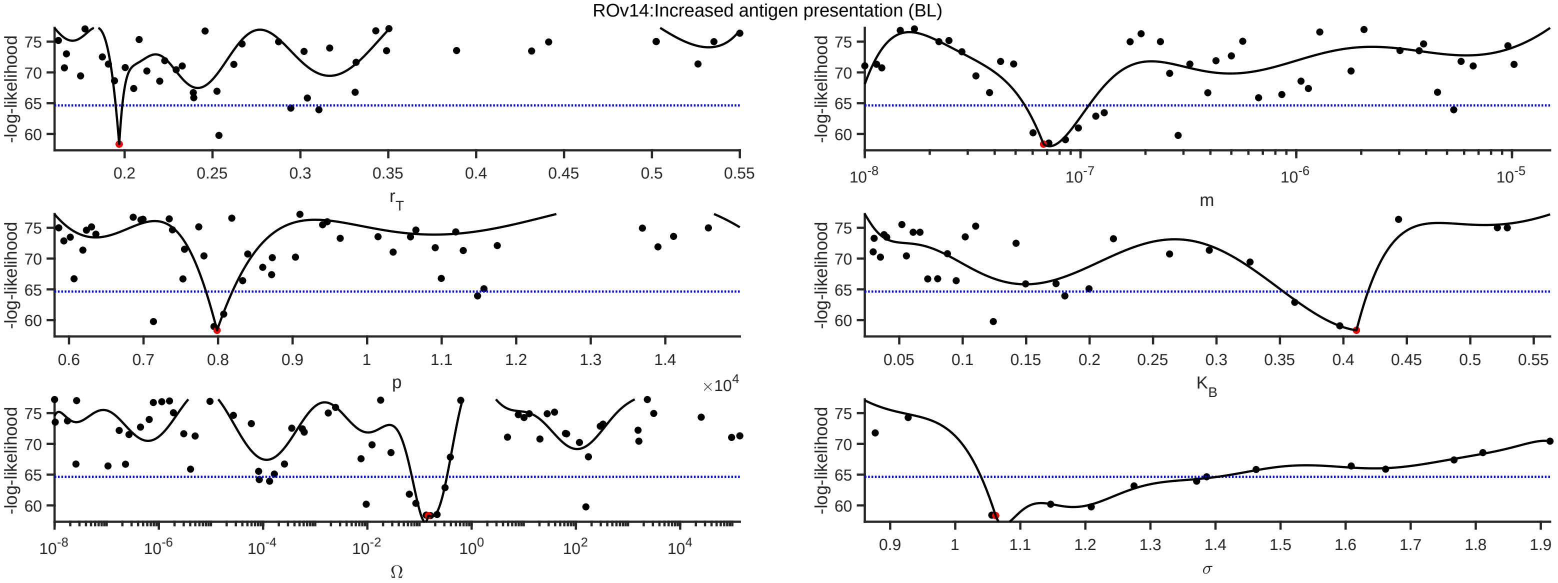
ROv14:Increased antigen presentation (BL)
-log-likelihood
r_T
m
p
K_B
Ω
σ
×10^4

9.2 Saturated effector cell source model

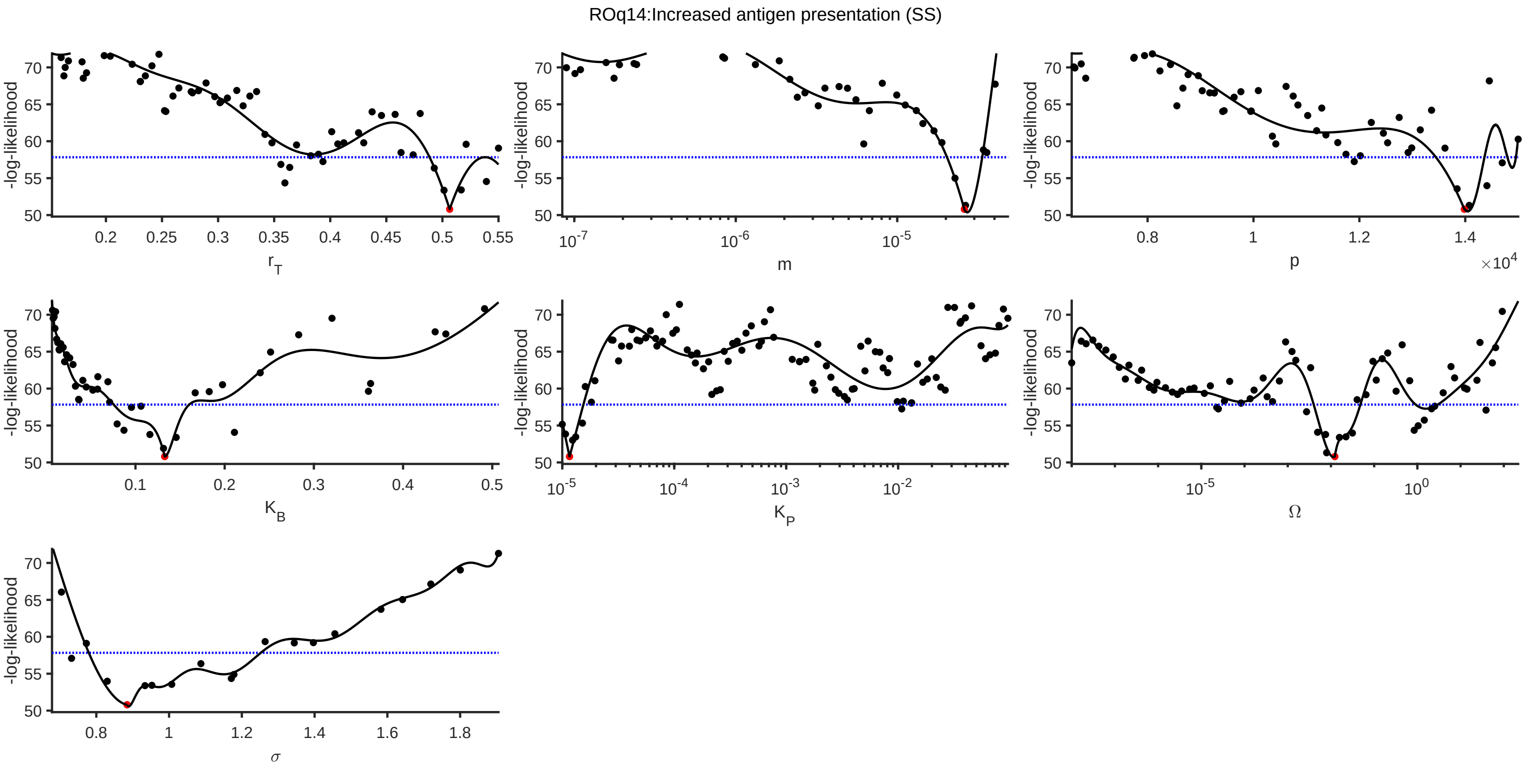

RDa15:Increased antigen presentation (SS)

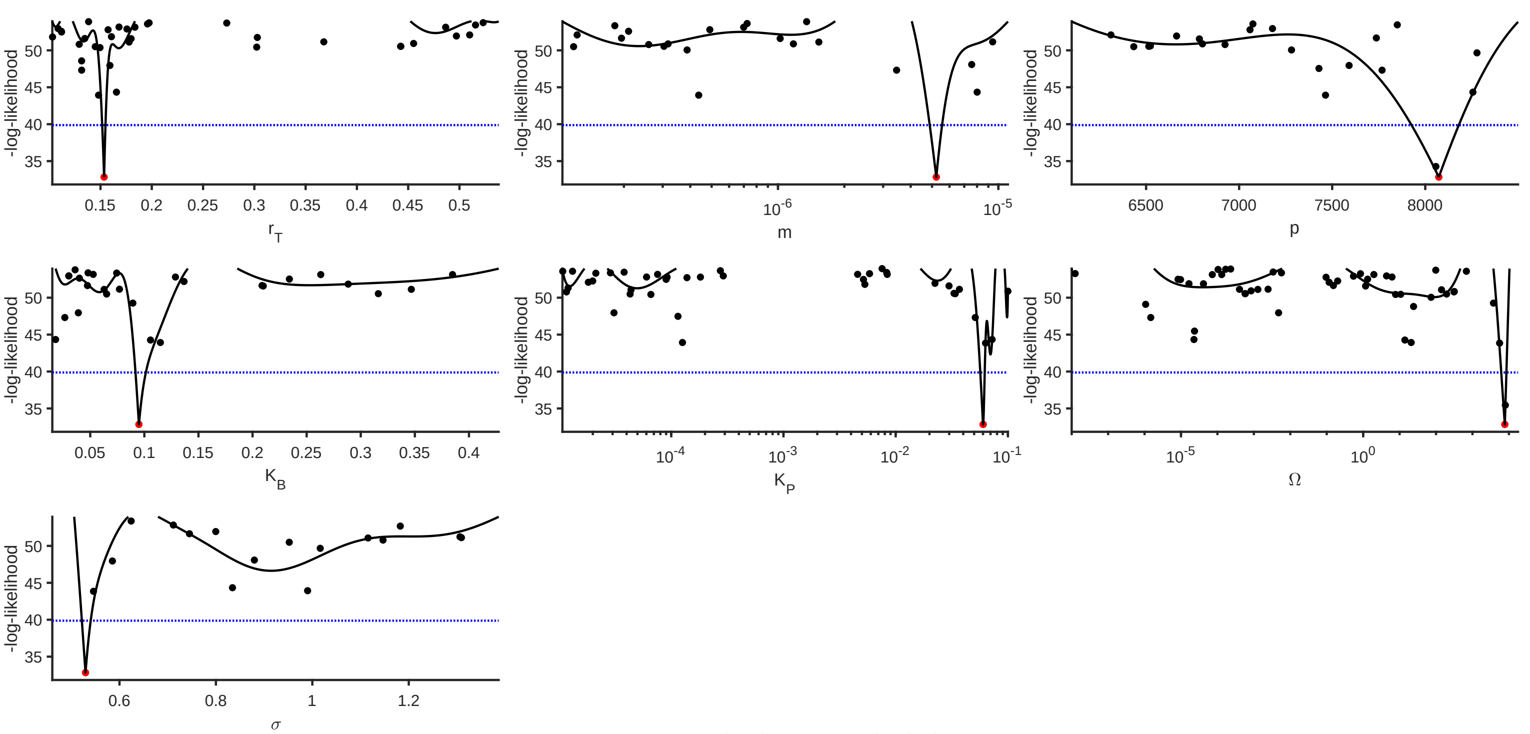

RFa15:Increased antigen presentation (SS)

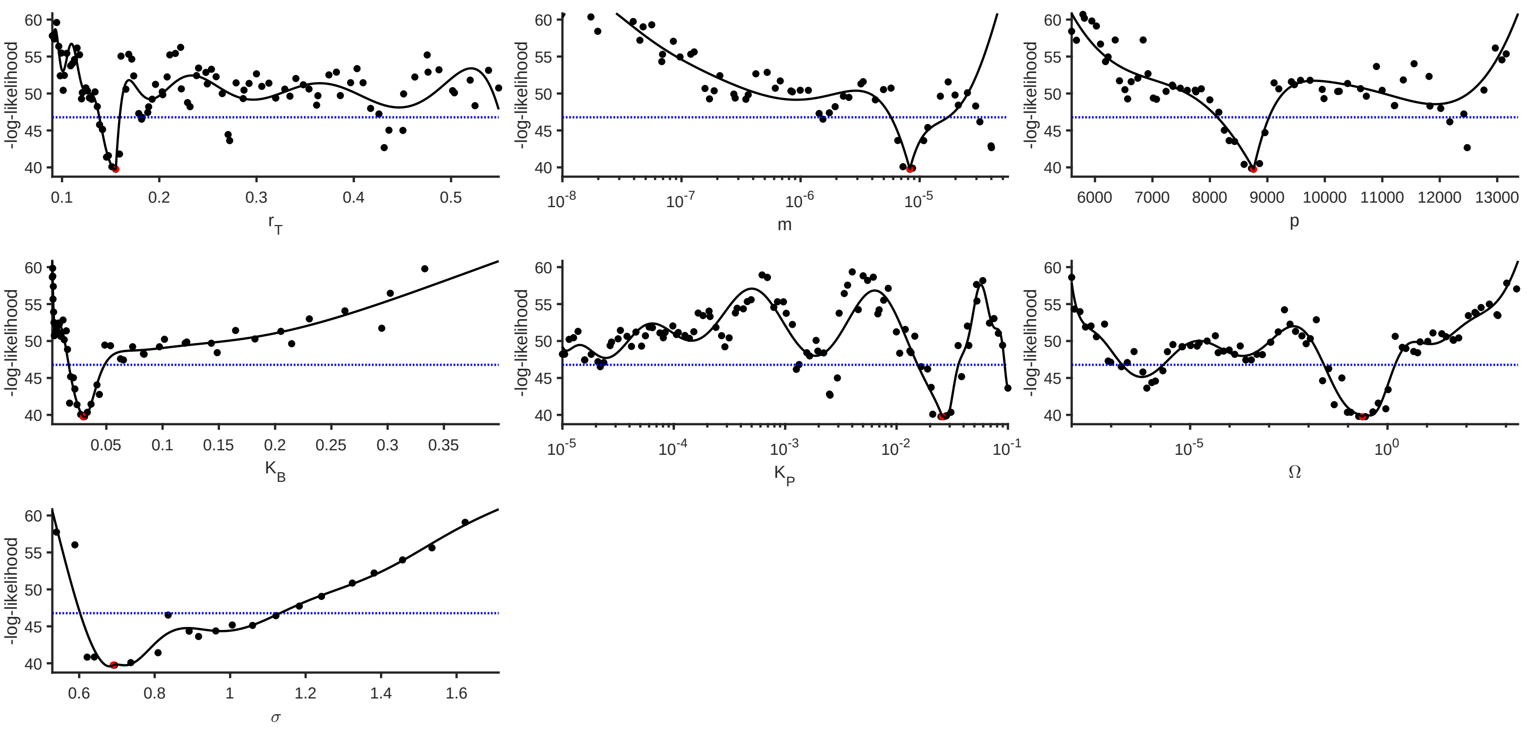

RLn12:Increased antigen presentation (SS)

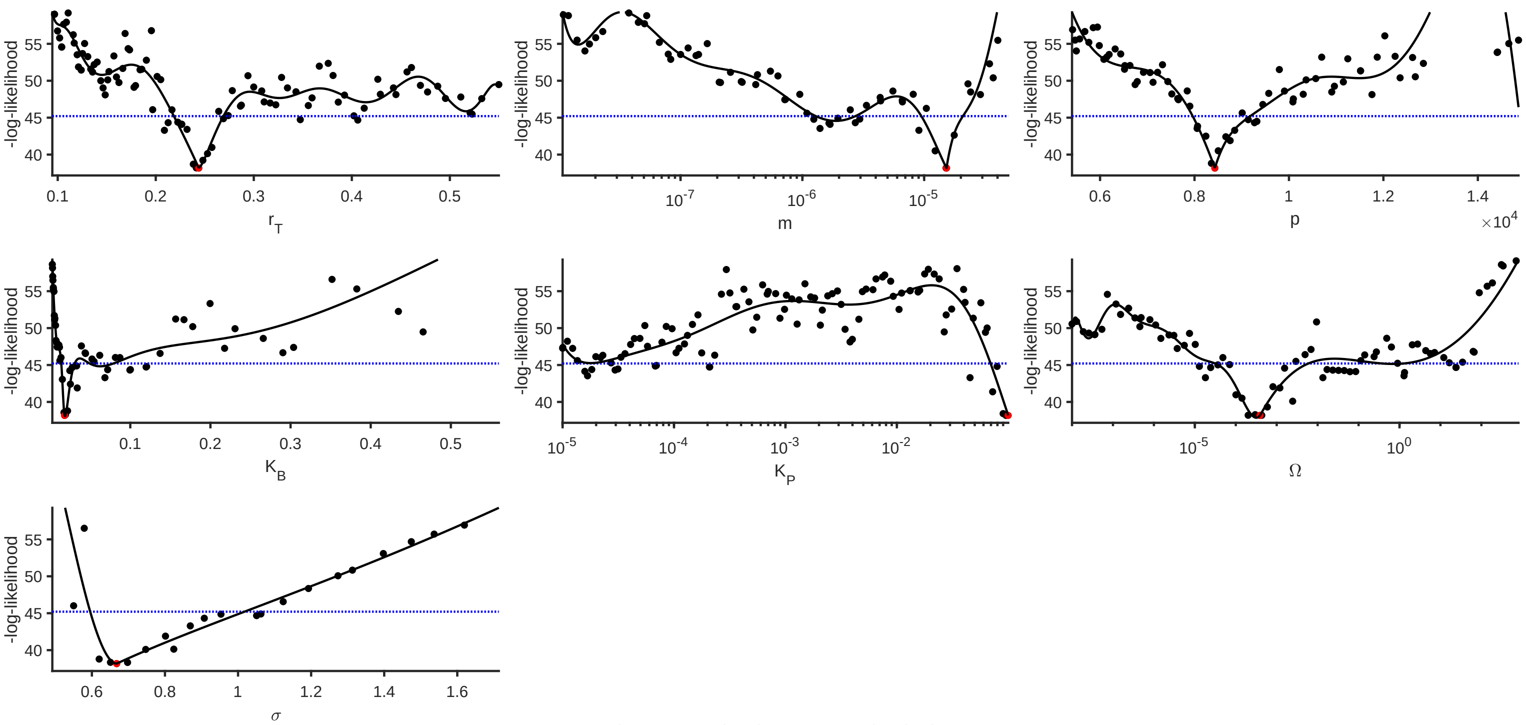

RId14:Increased antigen presentation (SS)

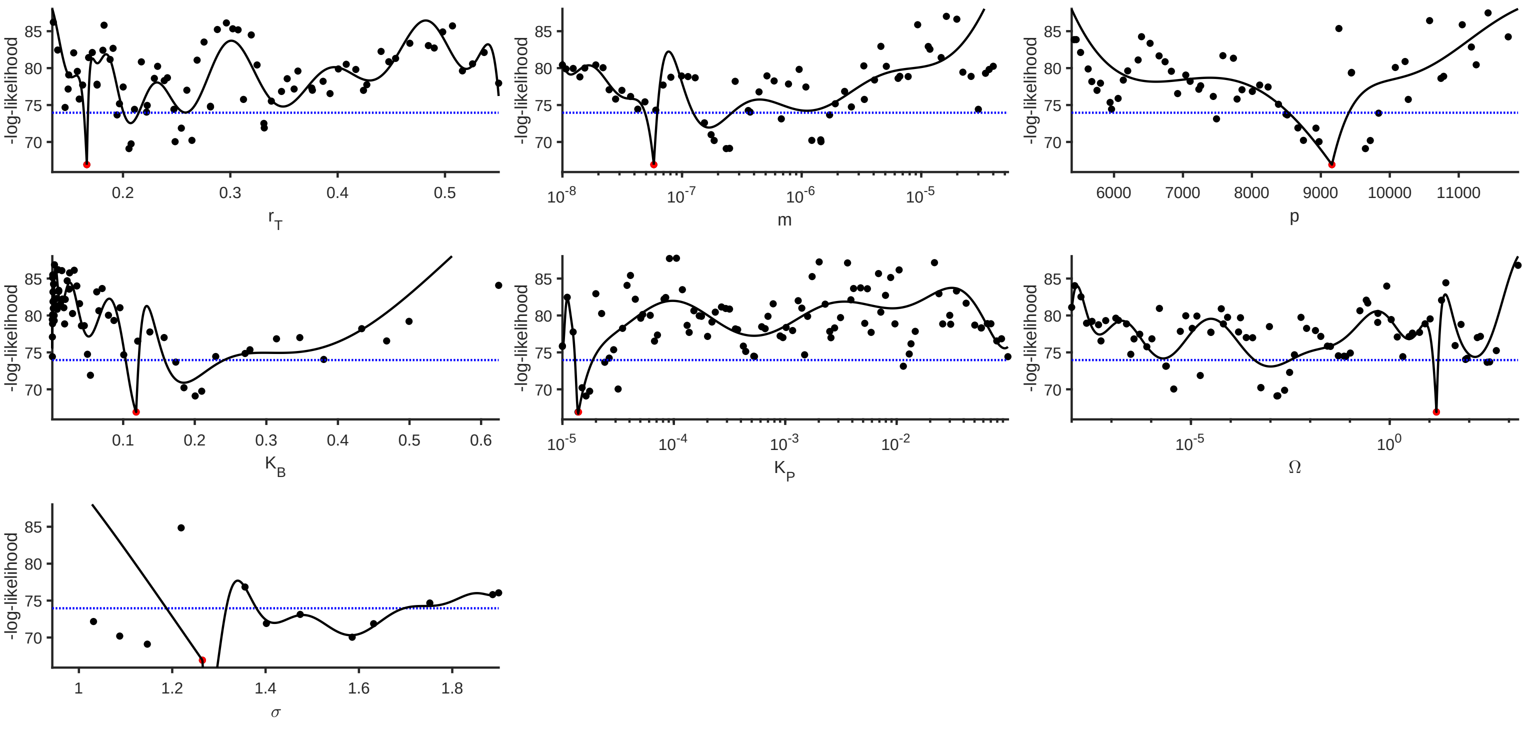

ROo13:Increased antigen presentation (SS)

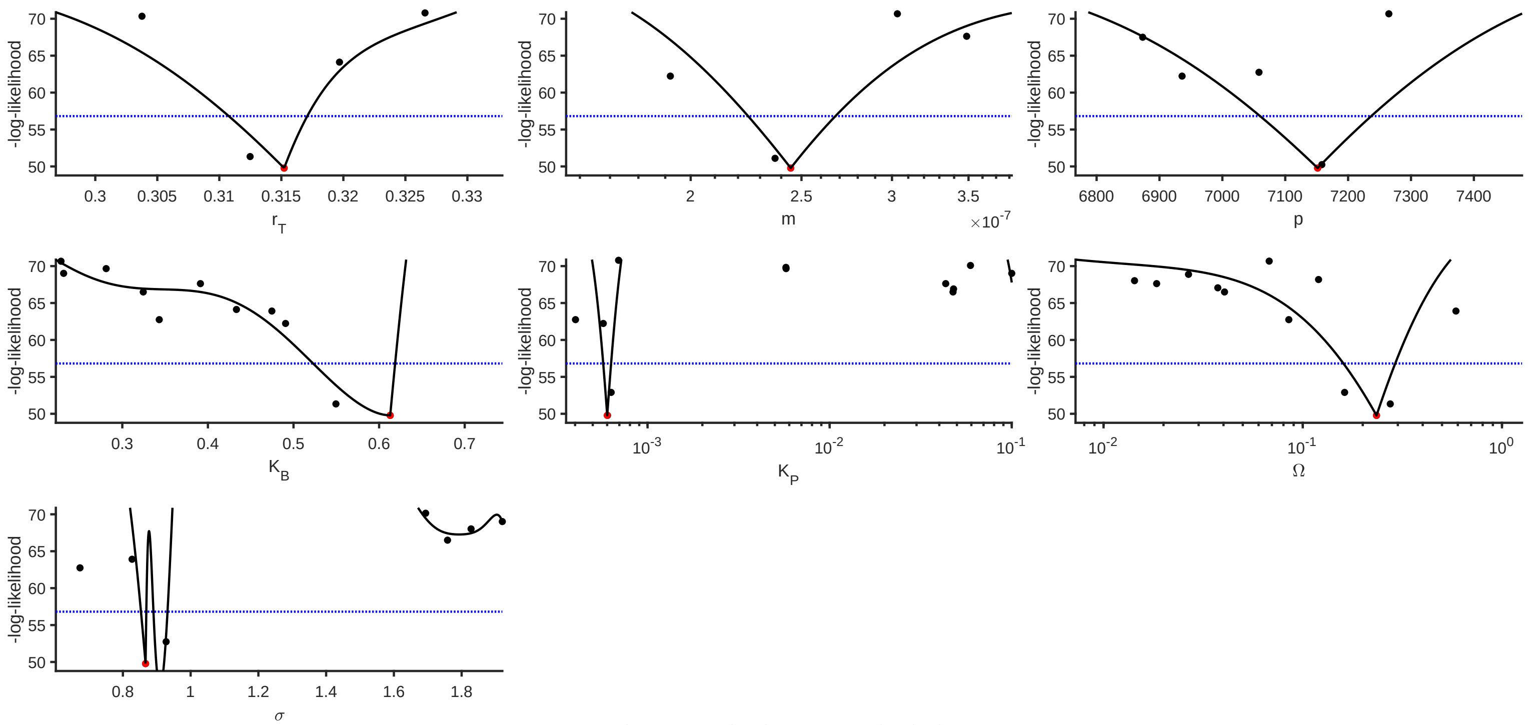

RSd14:Increased antigen presentation (SS)

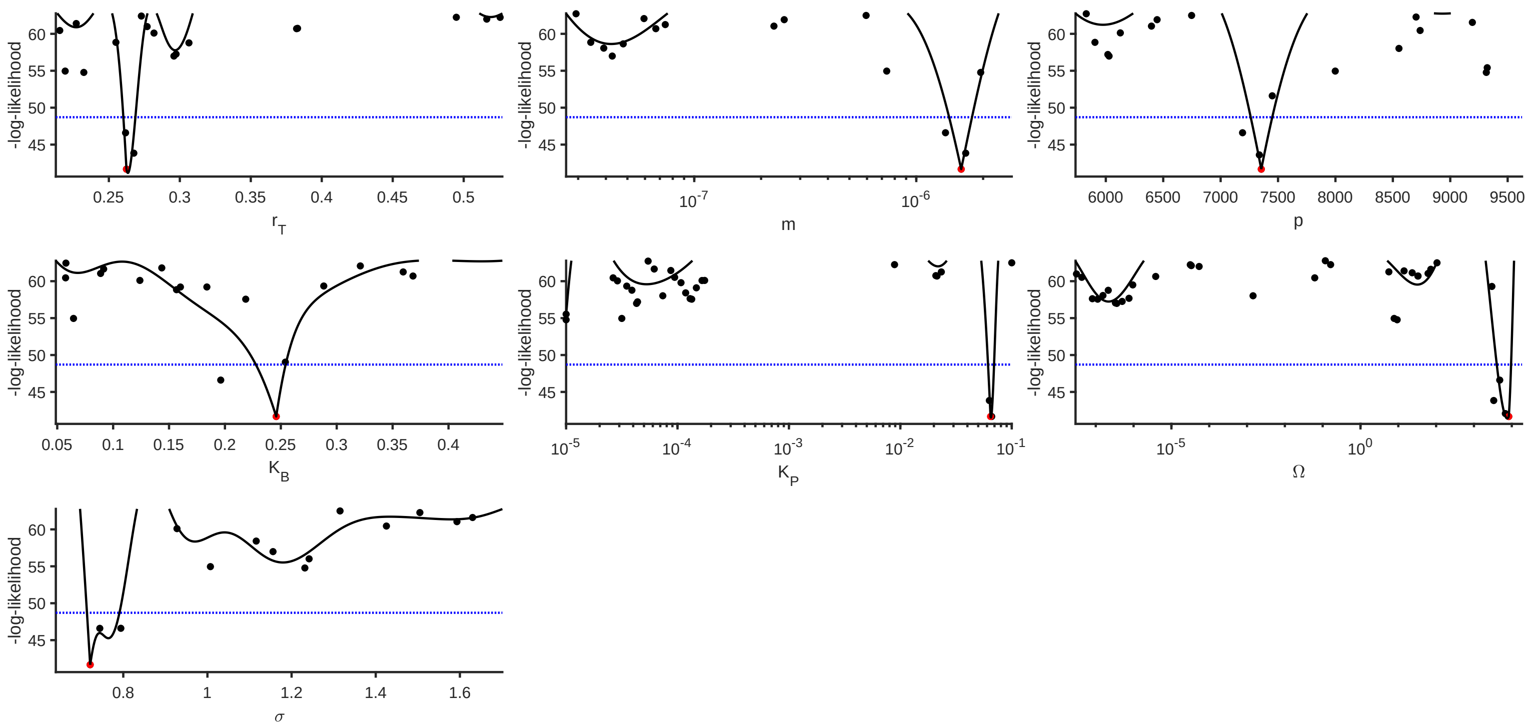

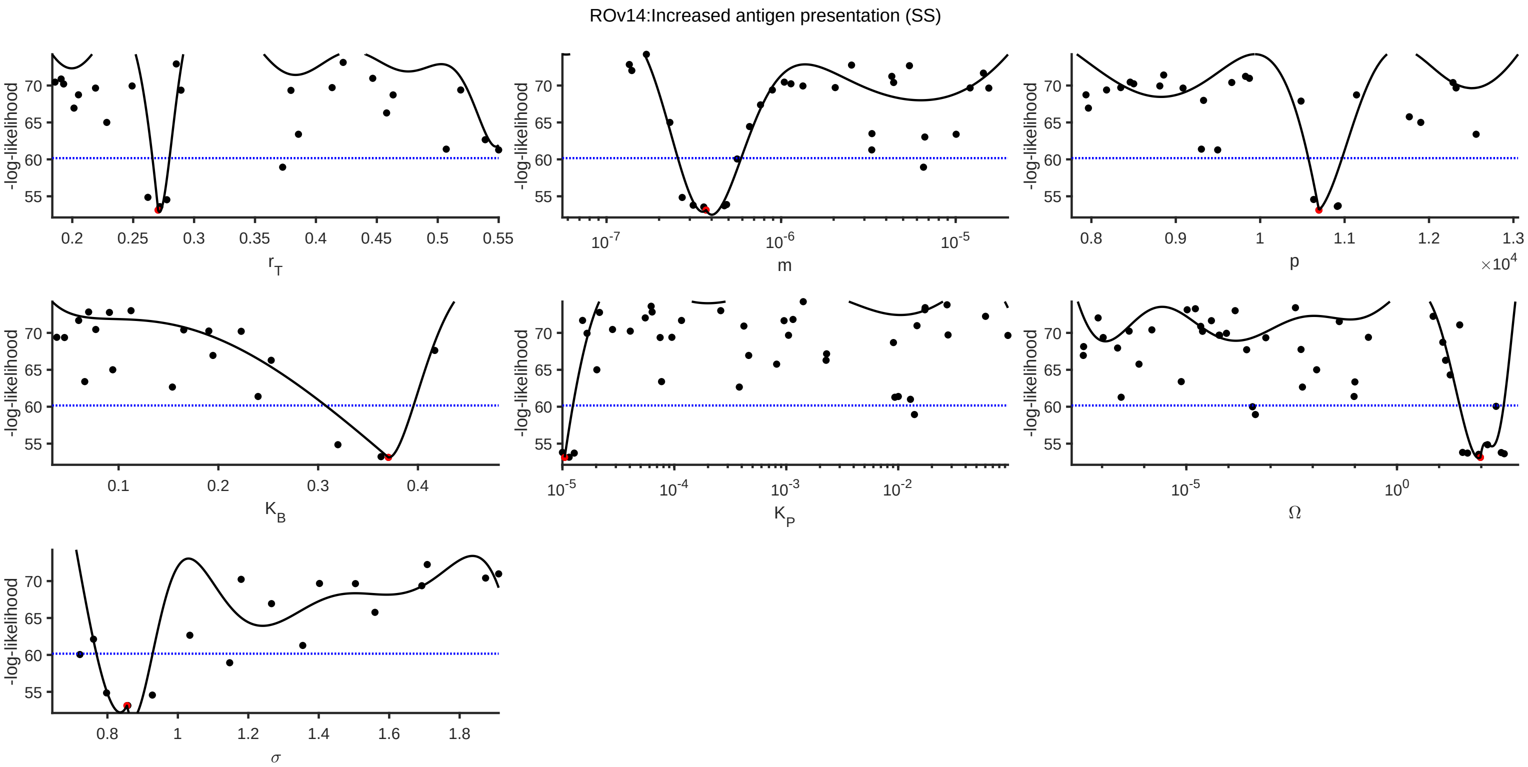
ROv14:Increased antigen presentation (SS)
-log-likelihood
r_T
m
p
$\times 10^4$
K_B
K_P
Ω
σ

9.3 Antigen presenting cell effector cell source model

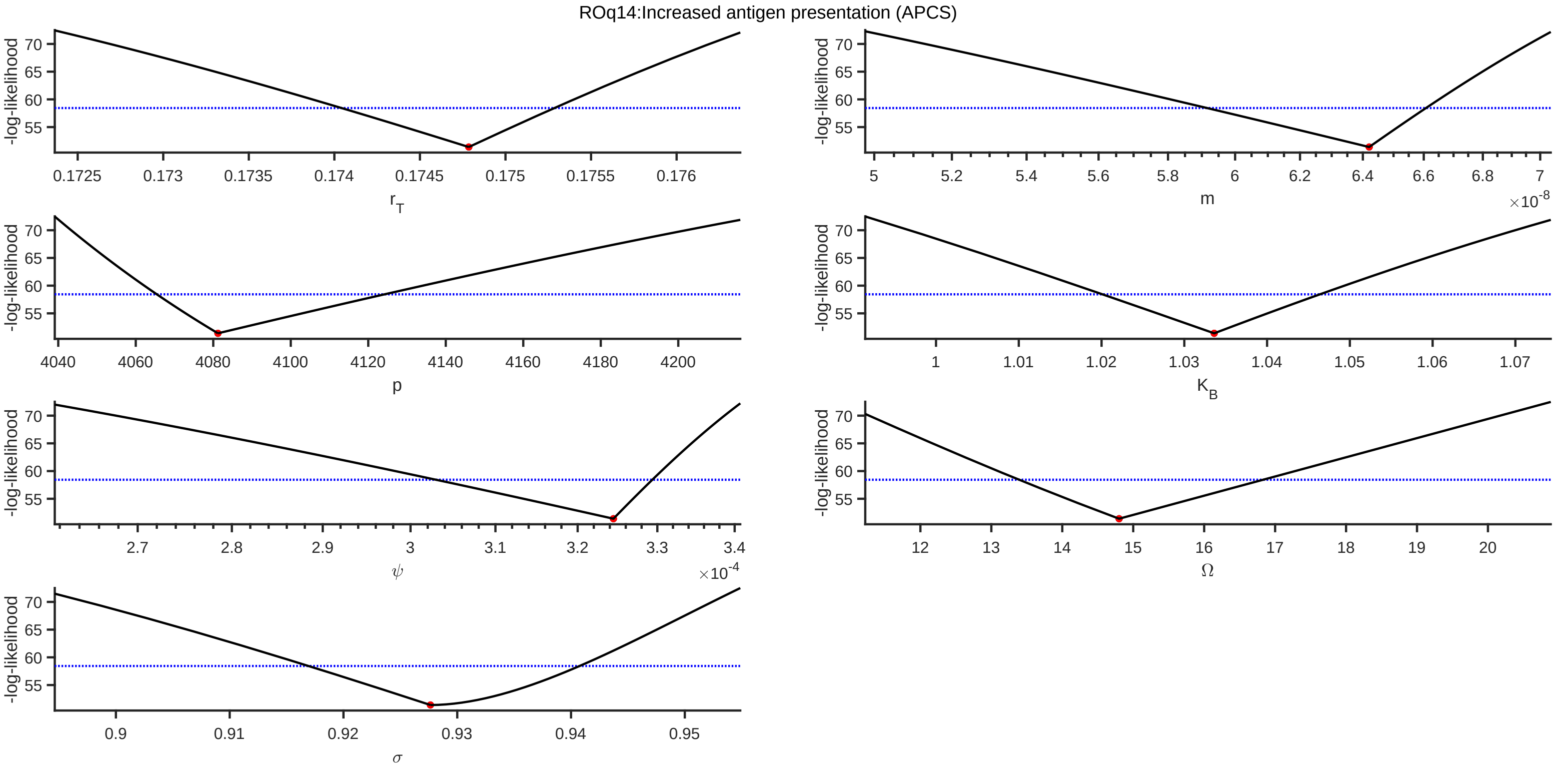

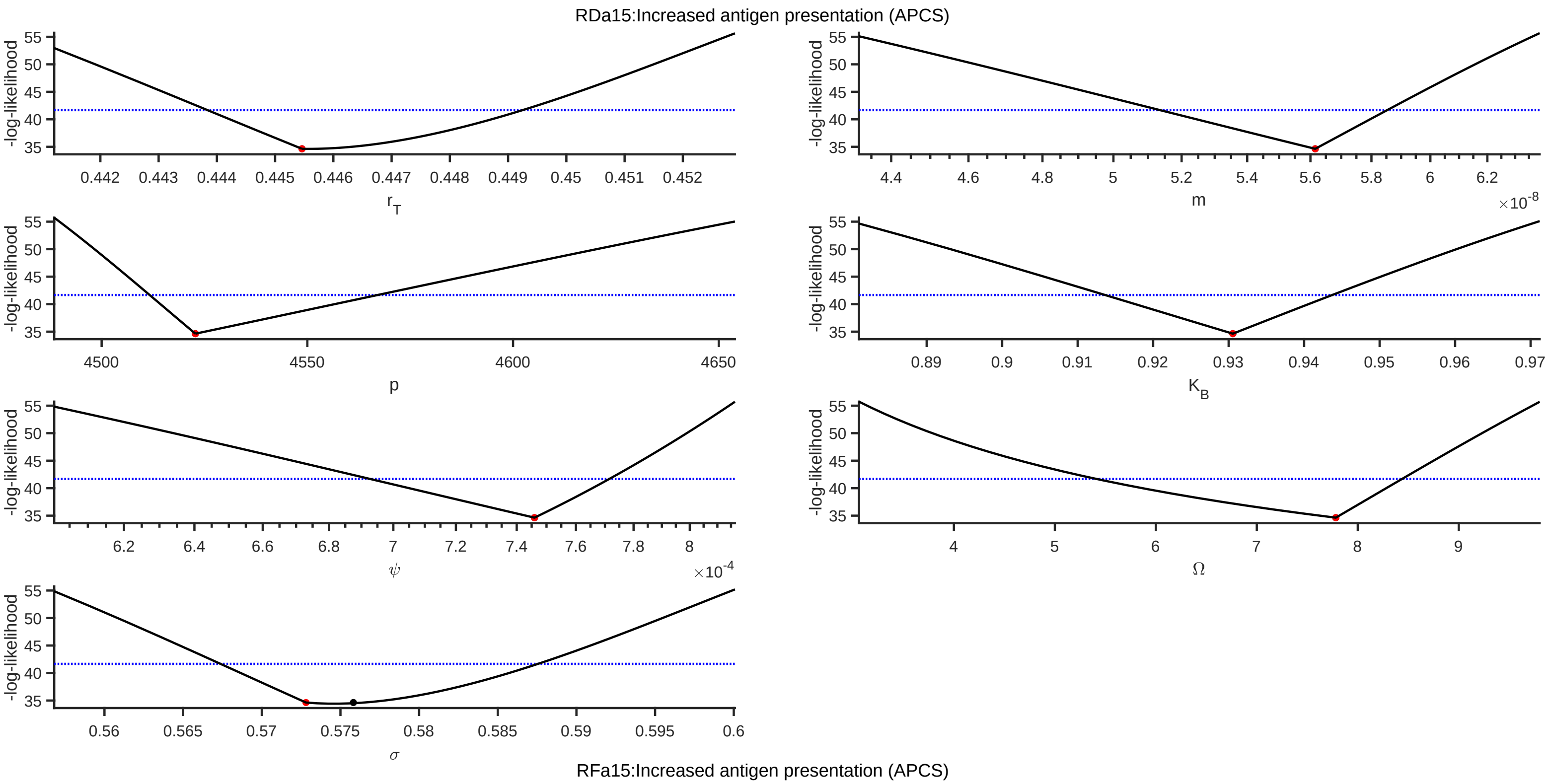
RDa15:Increased antigen presentation (APCS)
-log-likelihood
r_T
m
$\times 10^{-8}$
p
K_B
ψ
$\times 10^{-4}$
Ω
σ

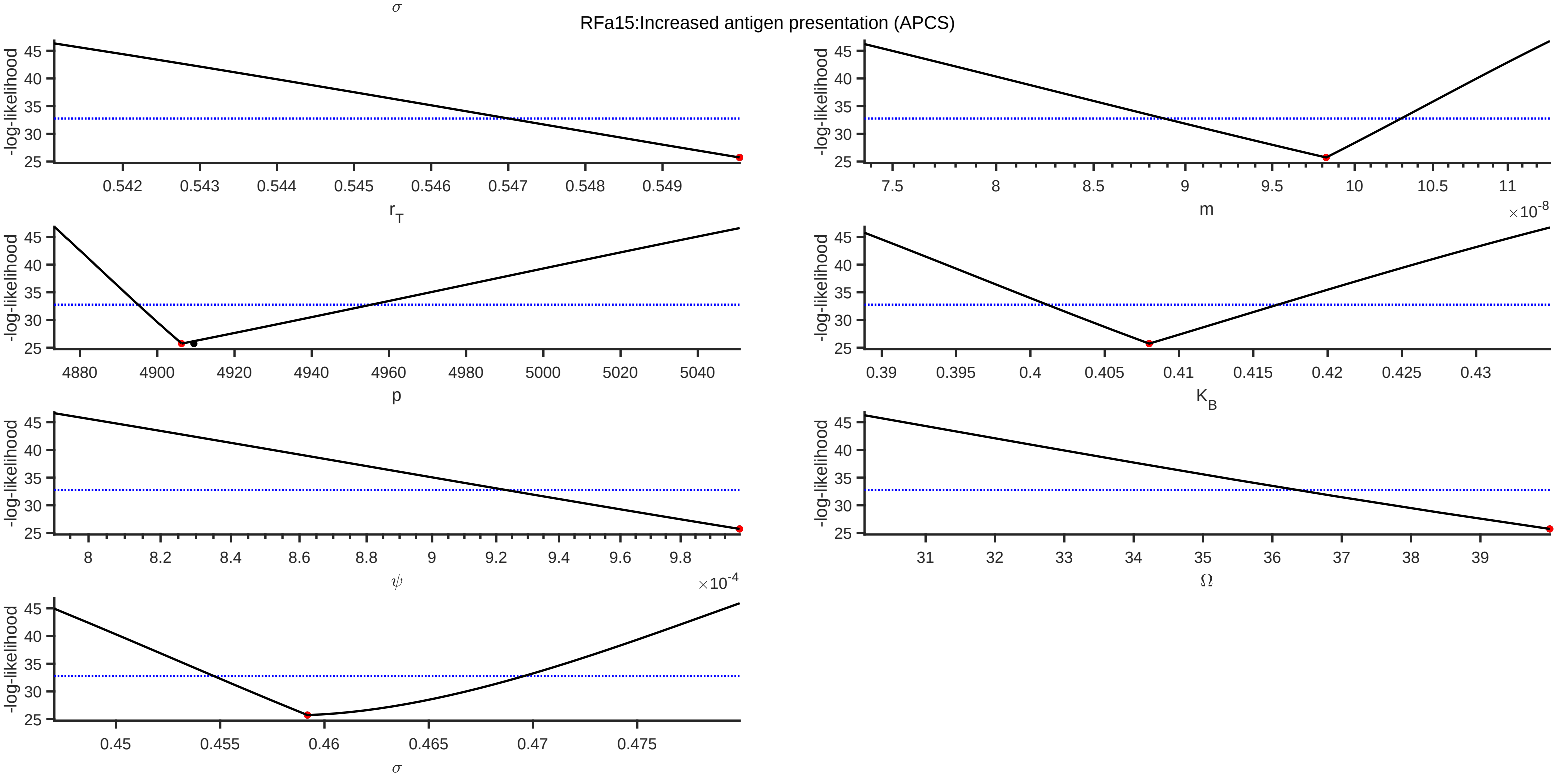
RFa15:Increased antigen presentation (APCS)
-log-likelihood
r_T
m
$\times 10^{-8}$
p
K_B
ψ
$\times 10^{-4}$
Ω
σ

RLn12:Increased antigen presentation (APCS)

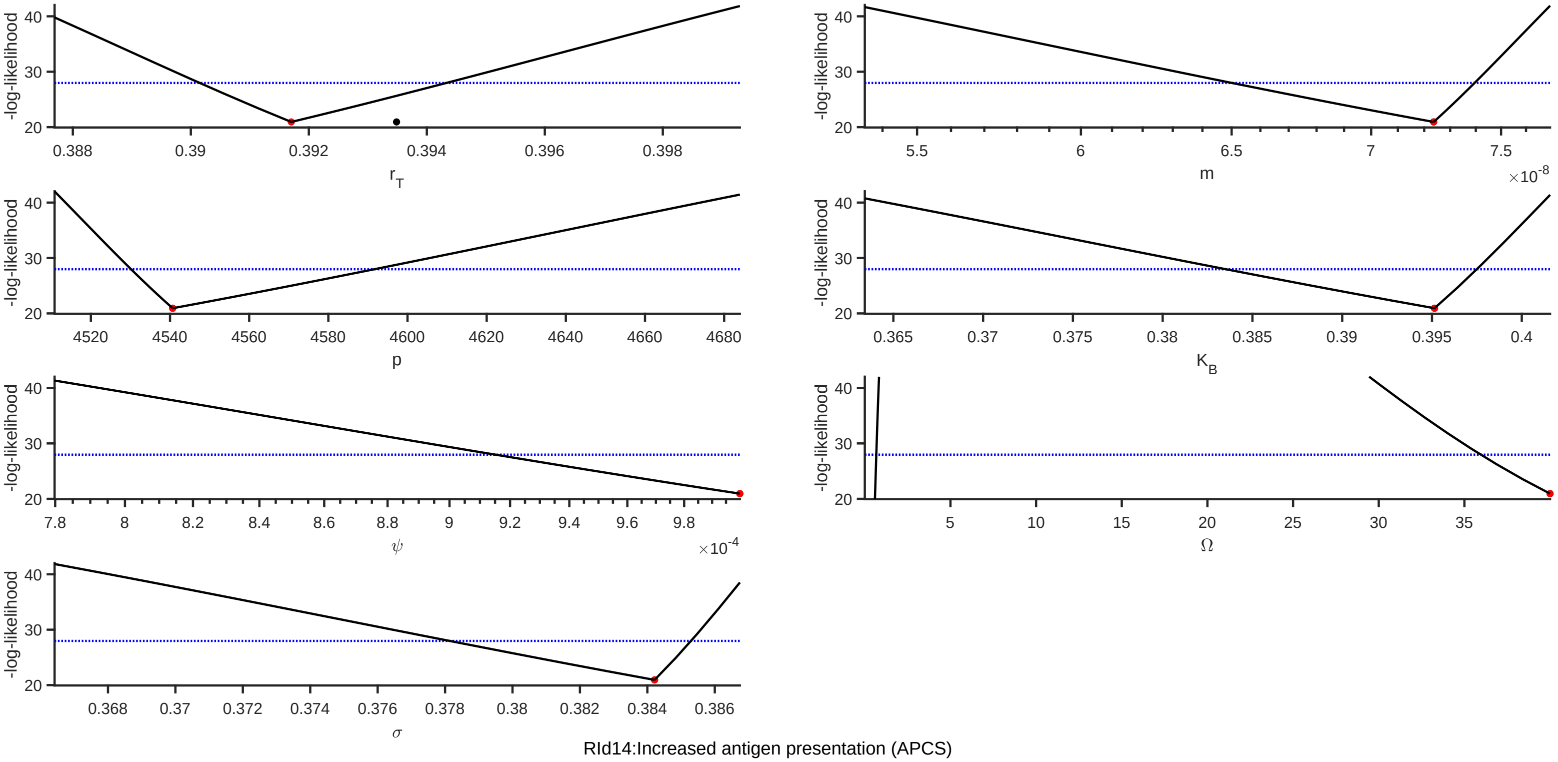

RId14:Increased antigen presentation (APCS)

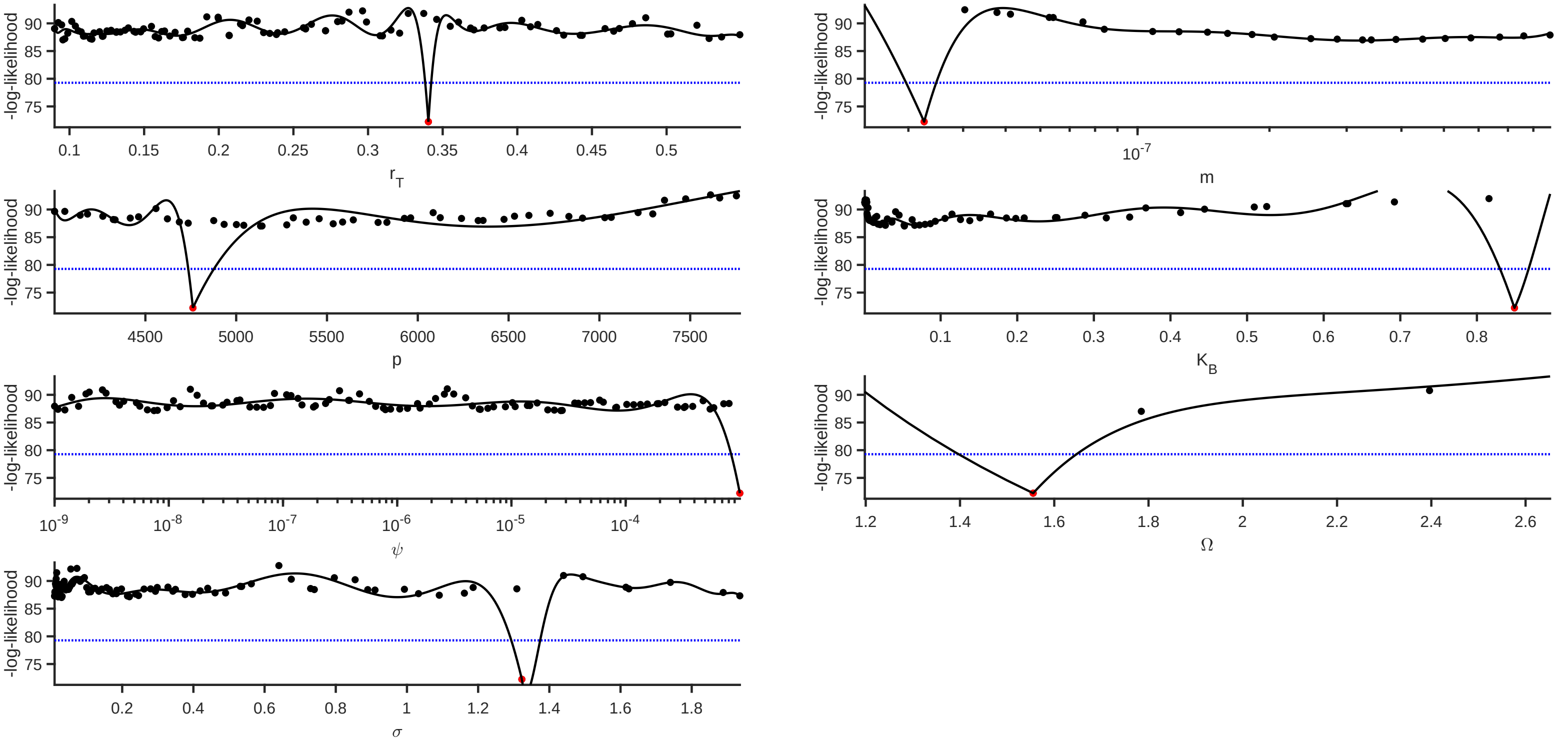

ROo13:Increased antigen presentation (APCS)

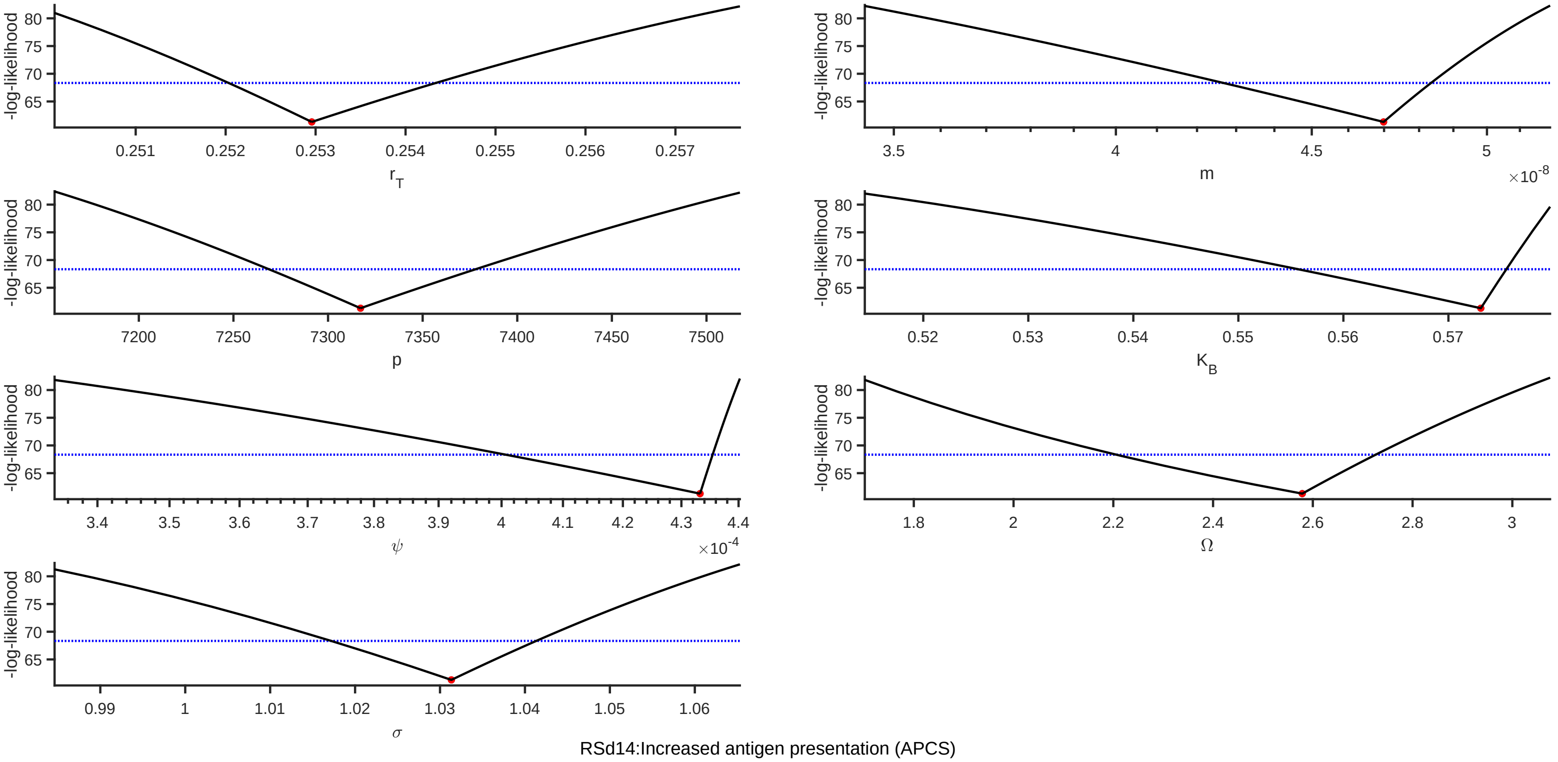

RSd14:Increased antigen presentation (APCS)

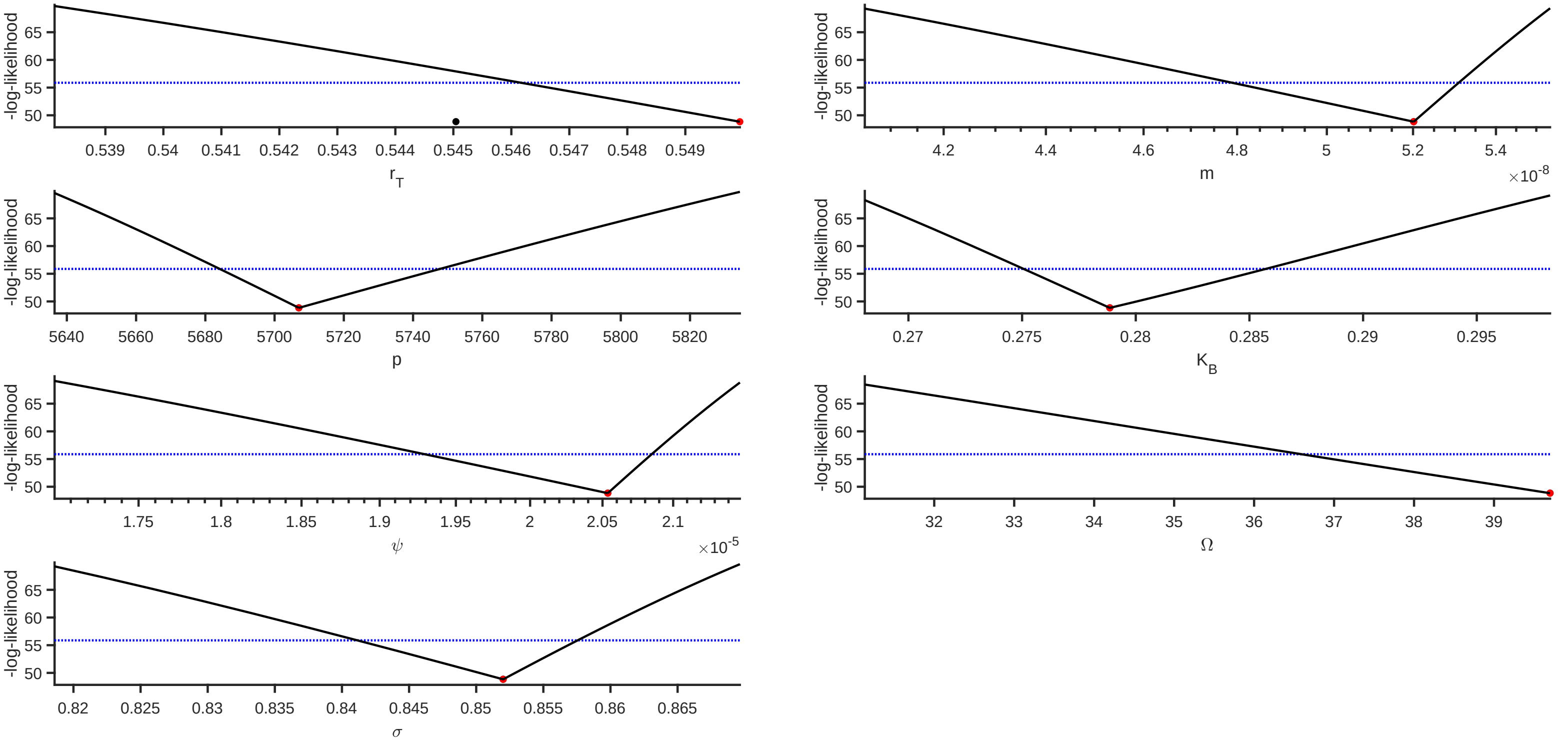

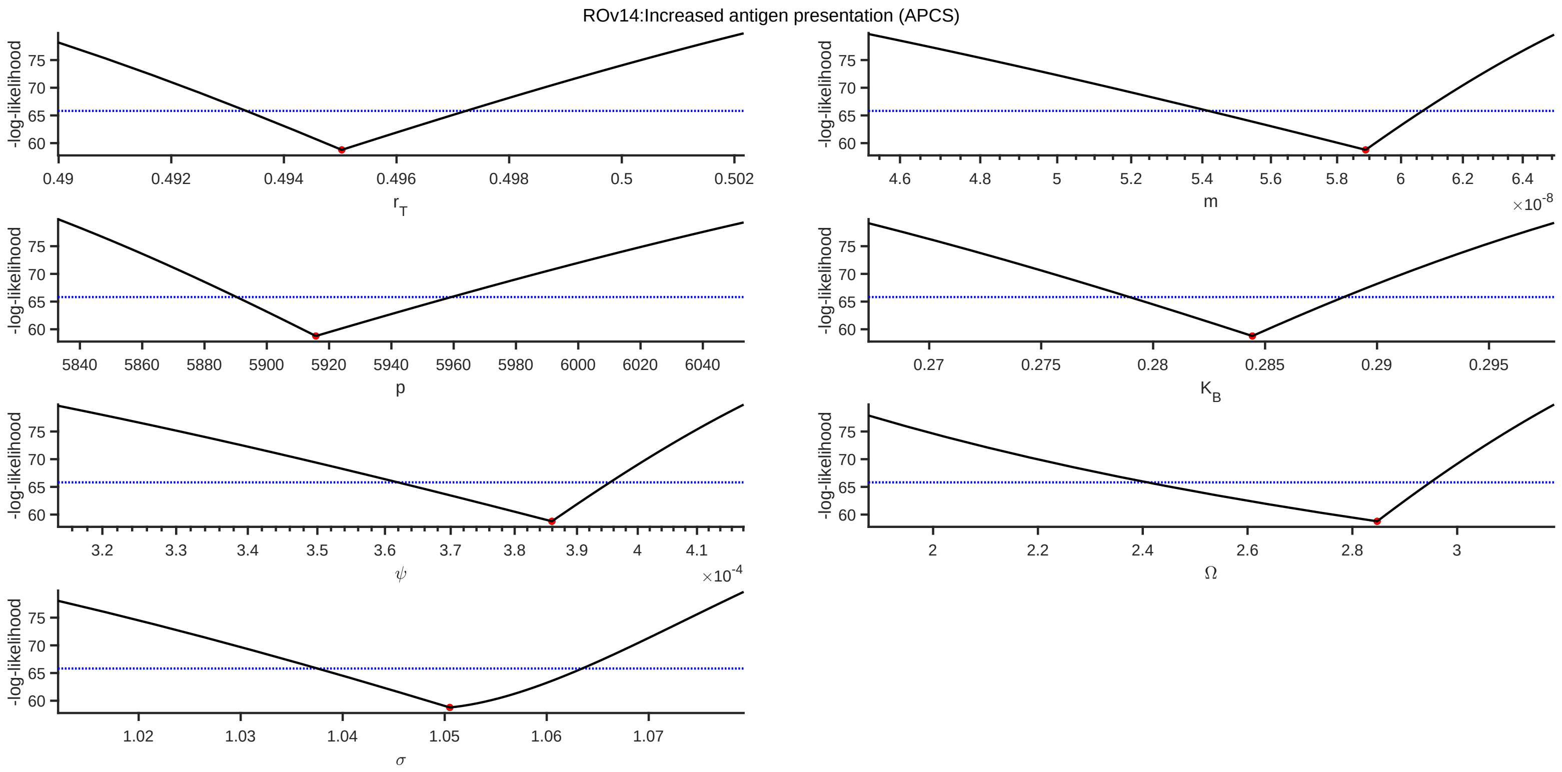
ROv14:Increased antigen presentation (APCS)
-log-likelihood
r_T
m
$\times 10^{-8}$
p
K_B
ψ
$\times 10^{-4}$
Ω
σ

10 Sensitivity anlaysis: Increased antigen presentation

10.1 Baseline effector cell source model

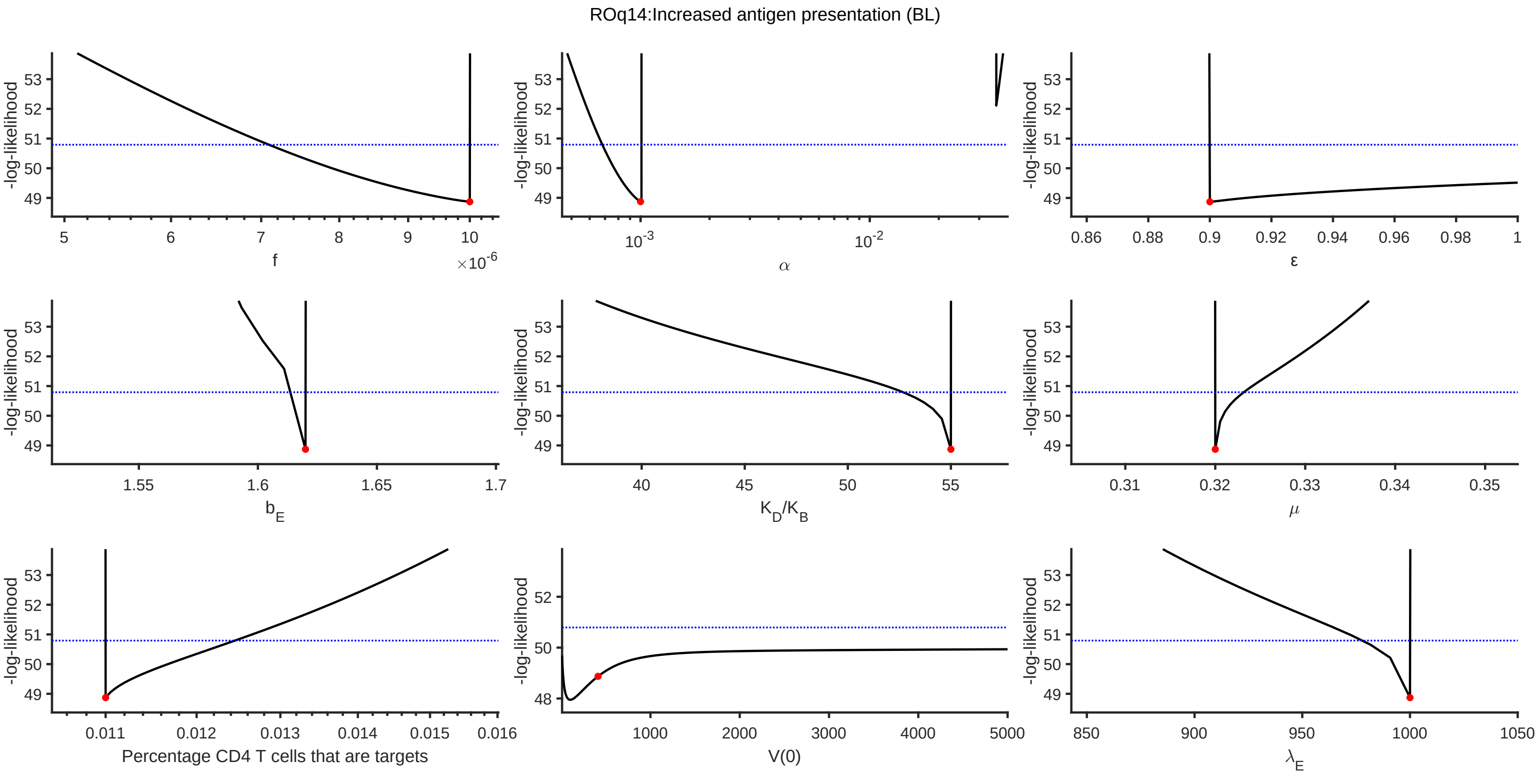

RDa15:Increased antigen presentation (BL)

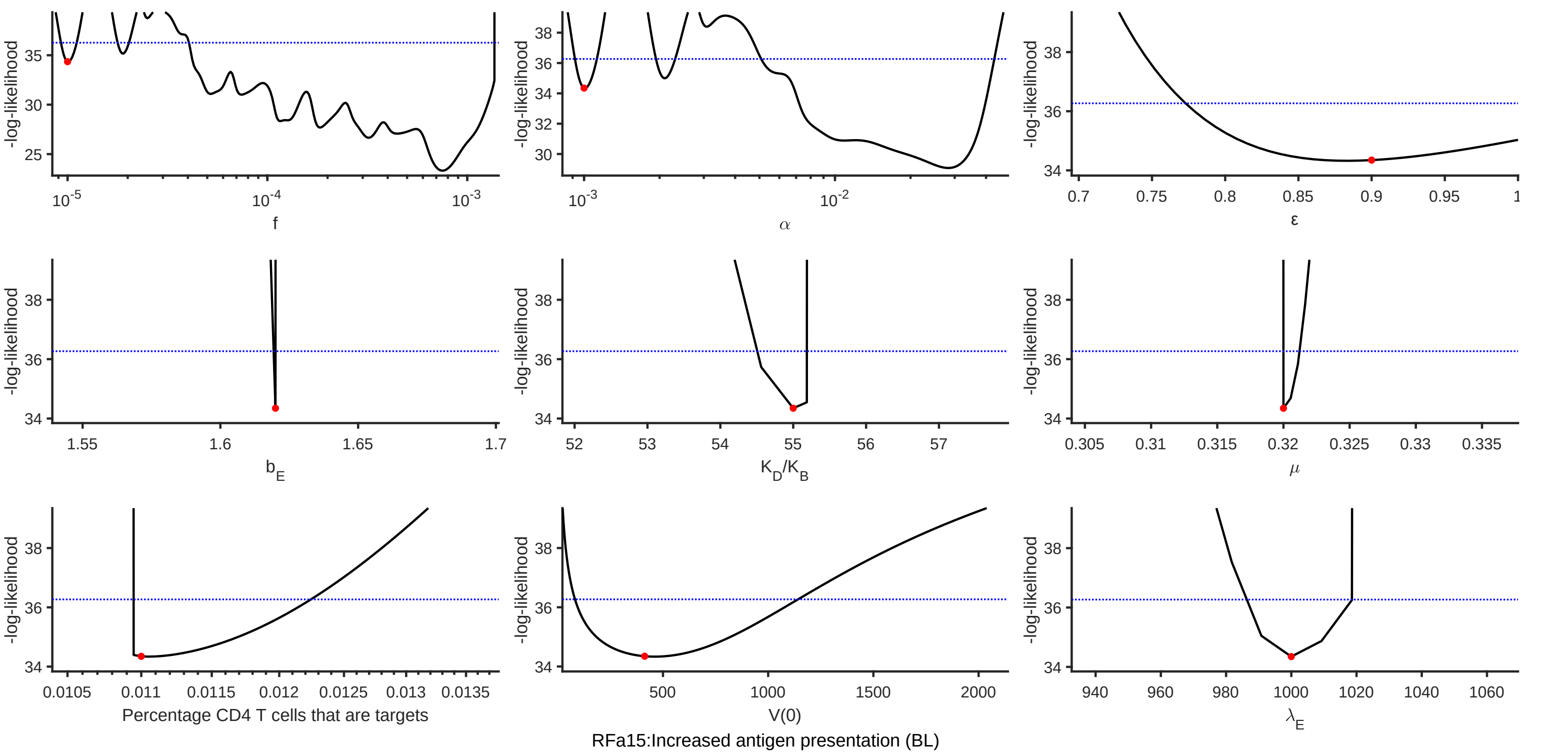

RFa15:Increased antigen presentation (BL)

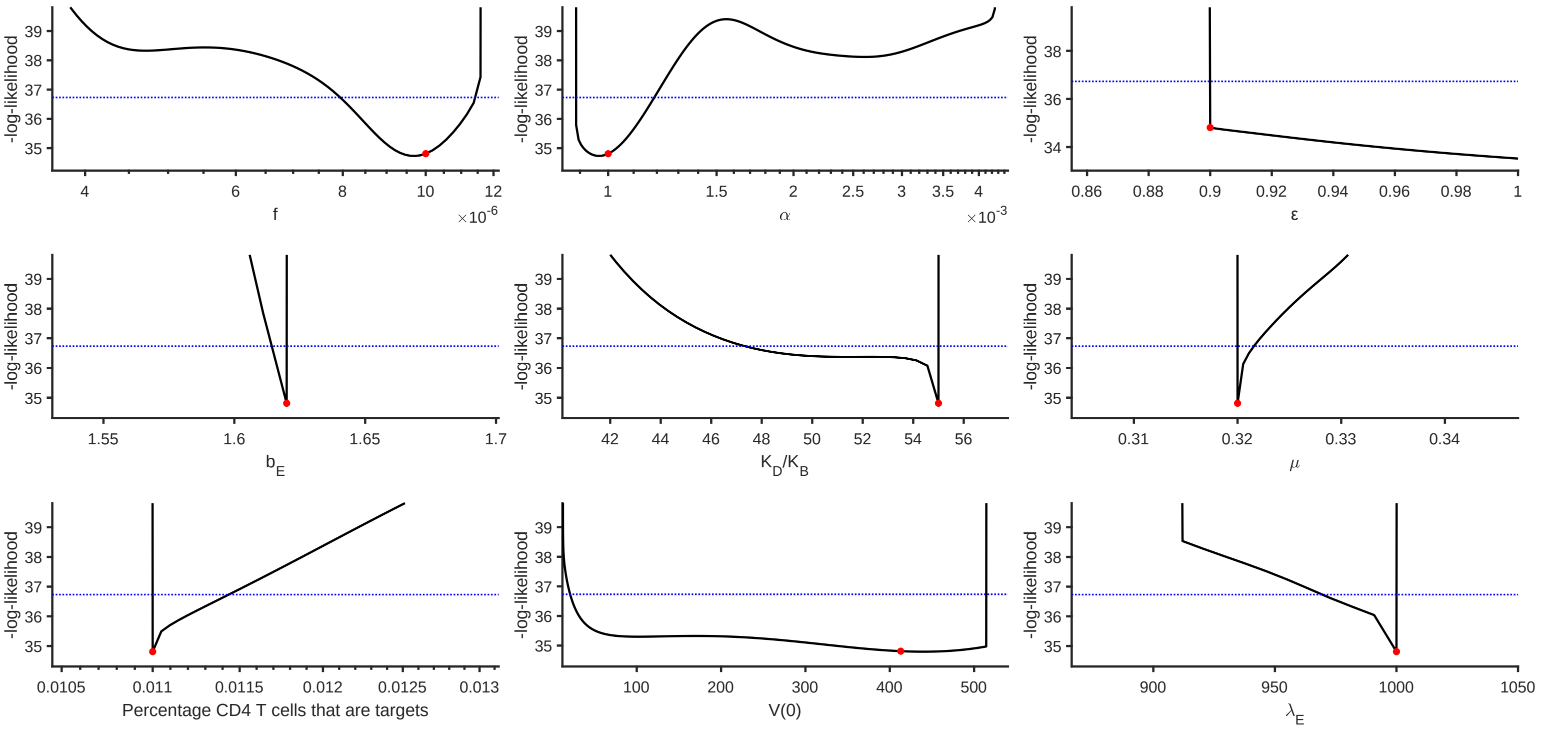

RLn12:Increased antigen presentation (BL)

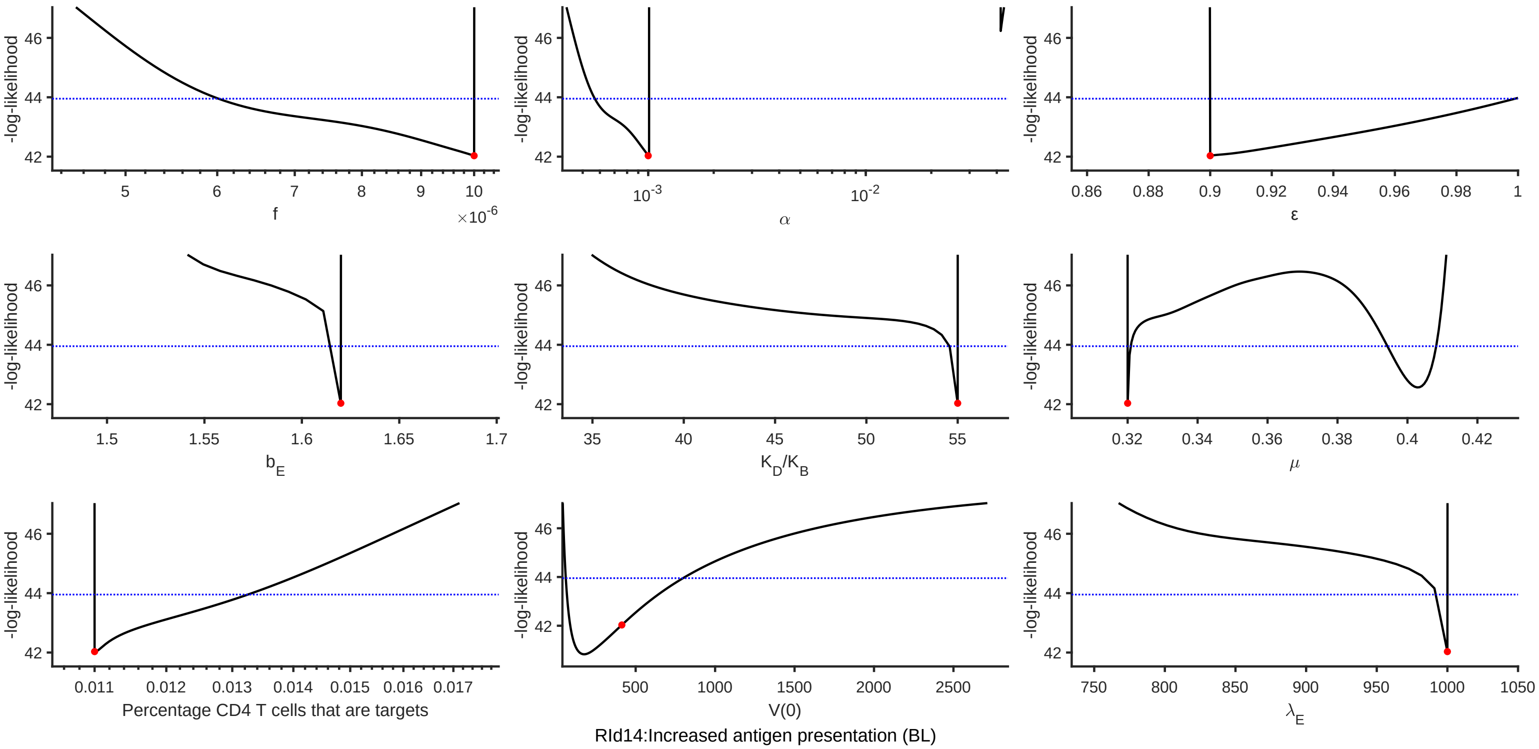

RId14:Increased antigen presentation (BL)

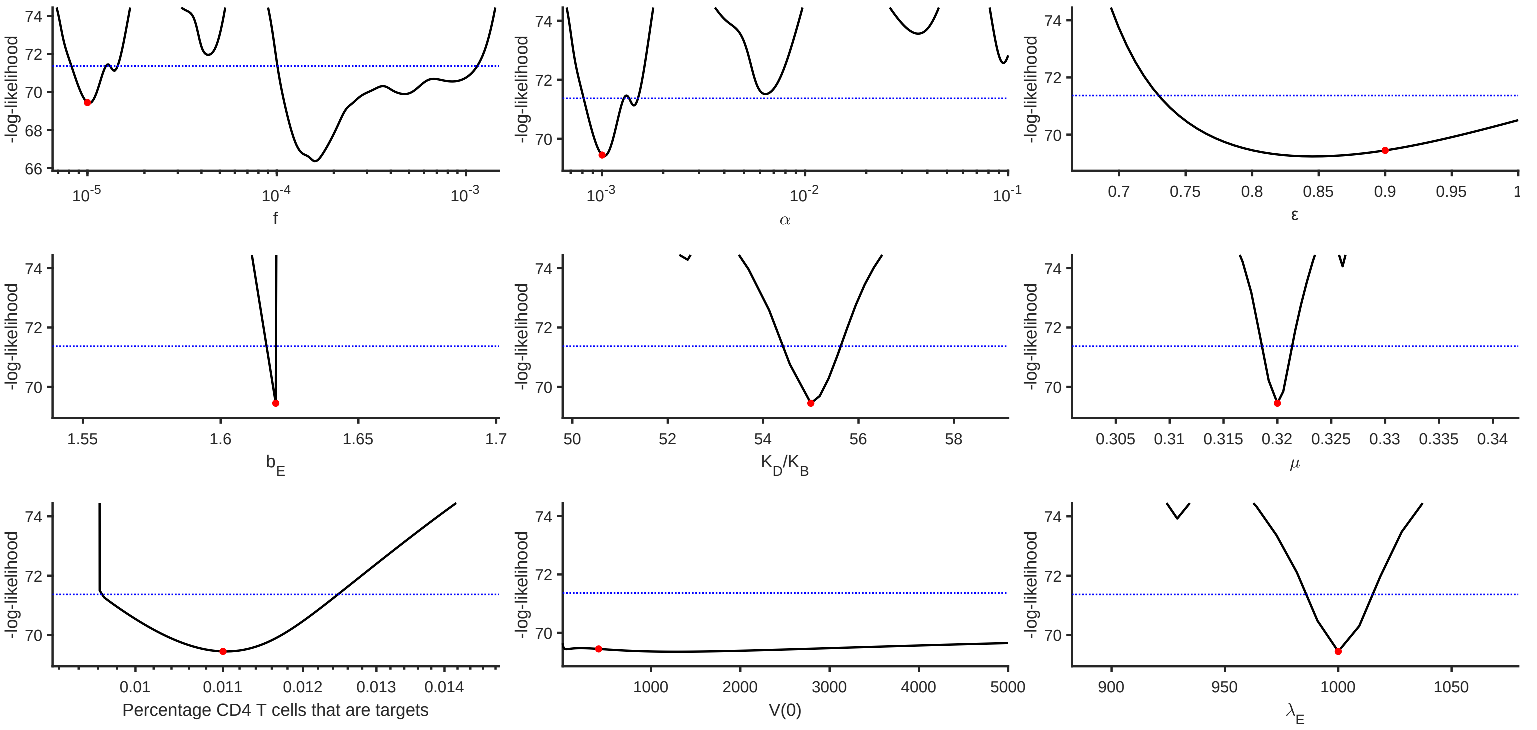

ROo13:Increased antigen presentation (BL)

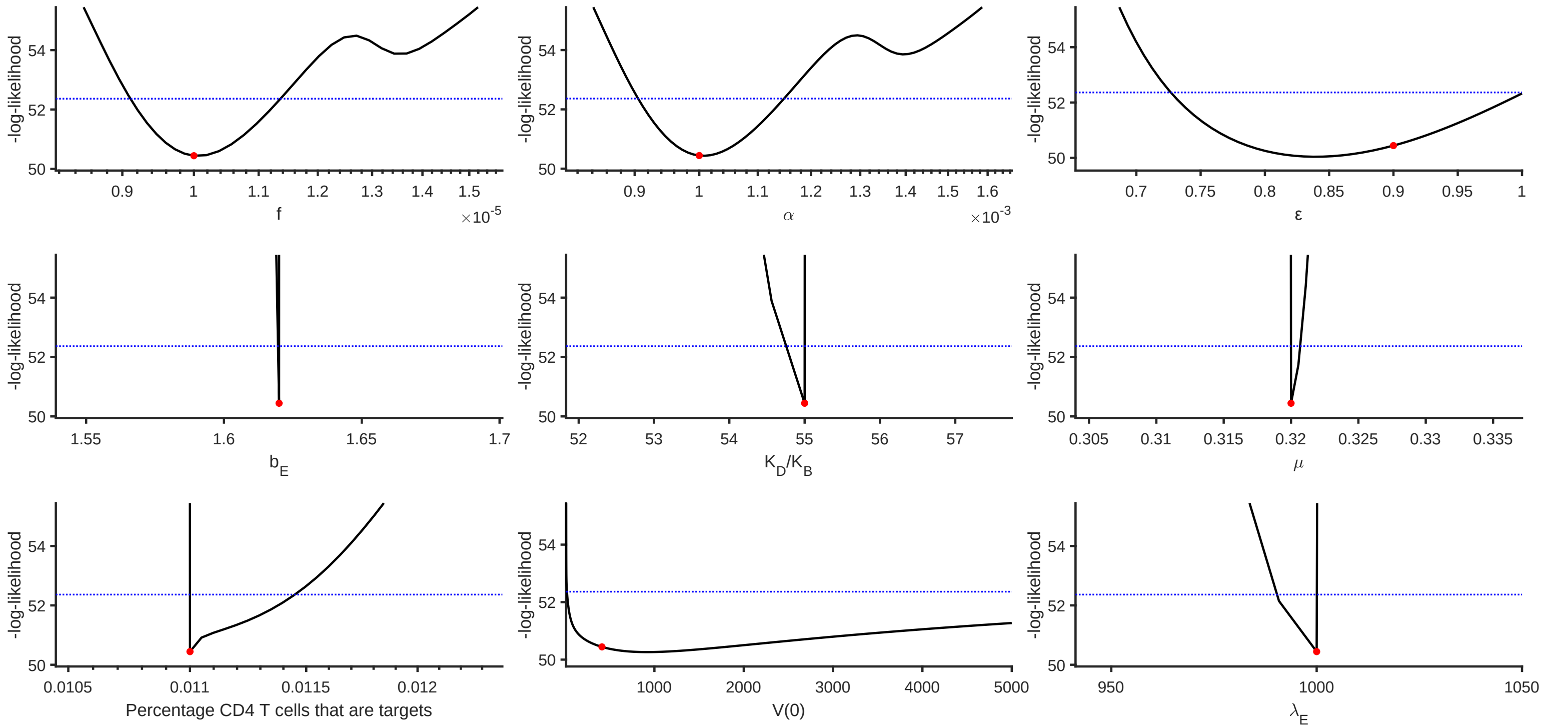

RSd14:Increased antigen presentation (BL)

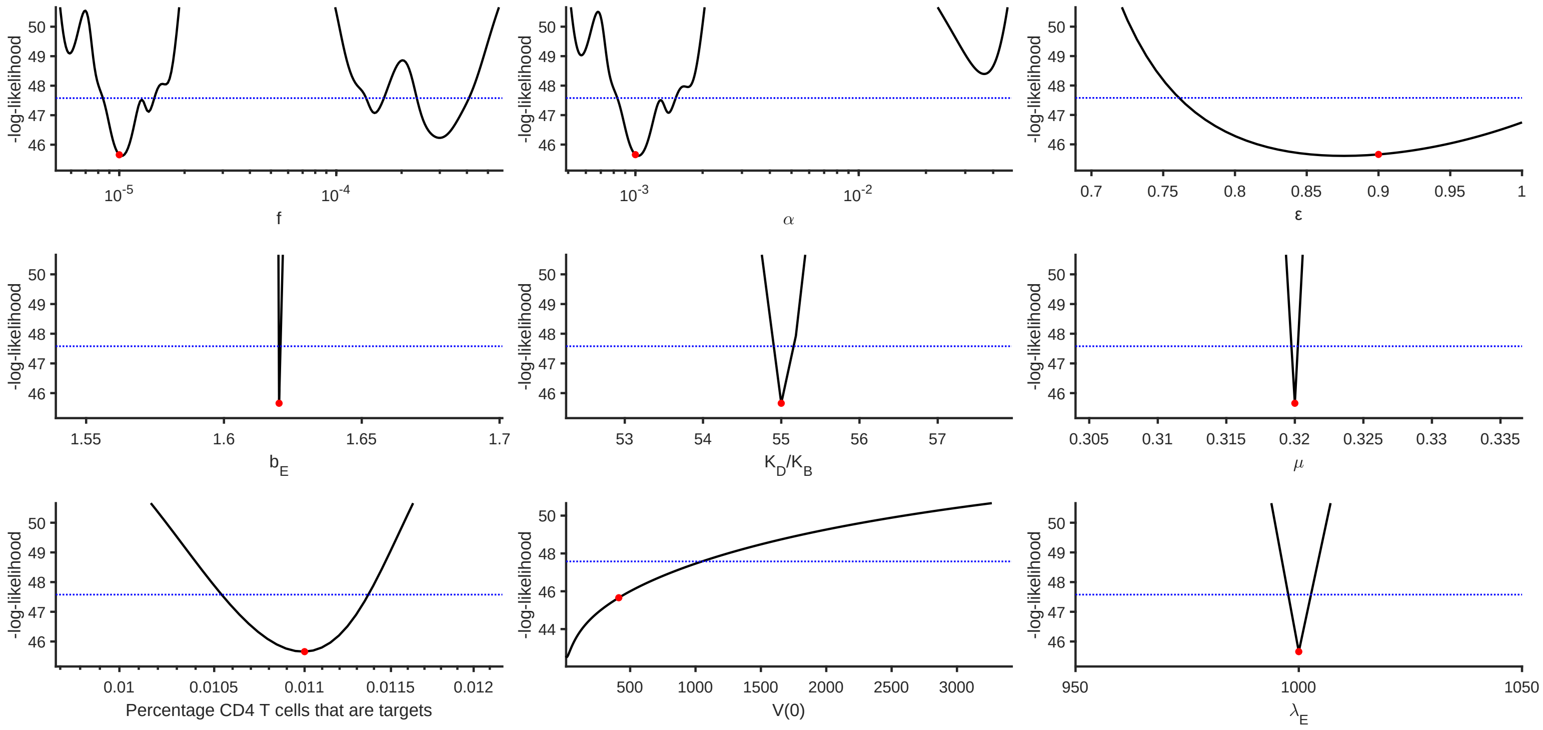

ROv14:Increased antigen presentation (BL)

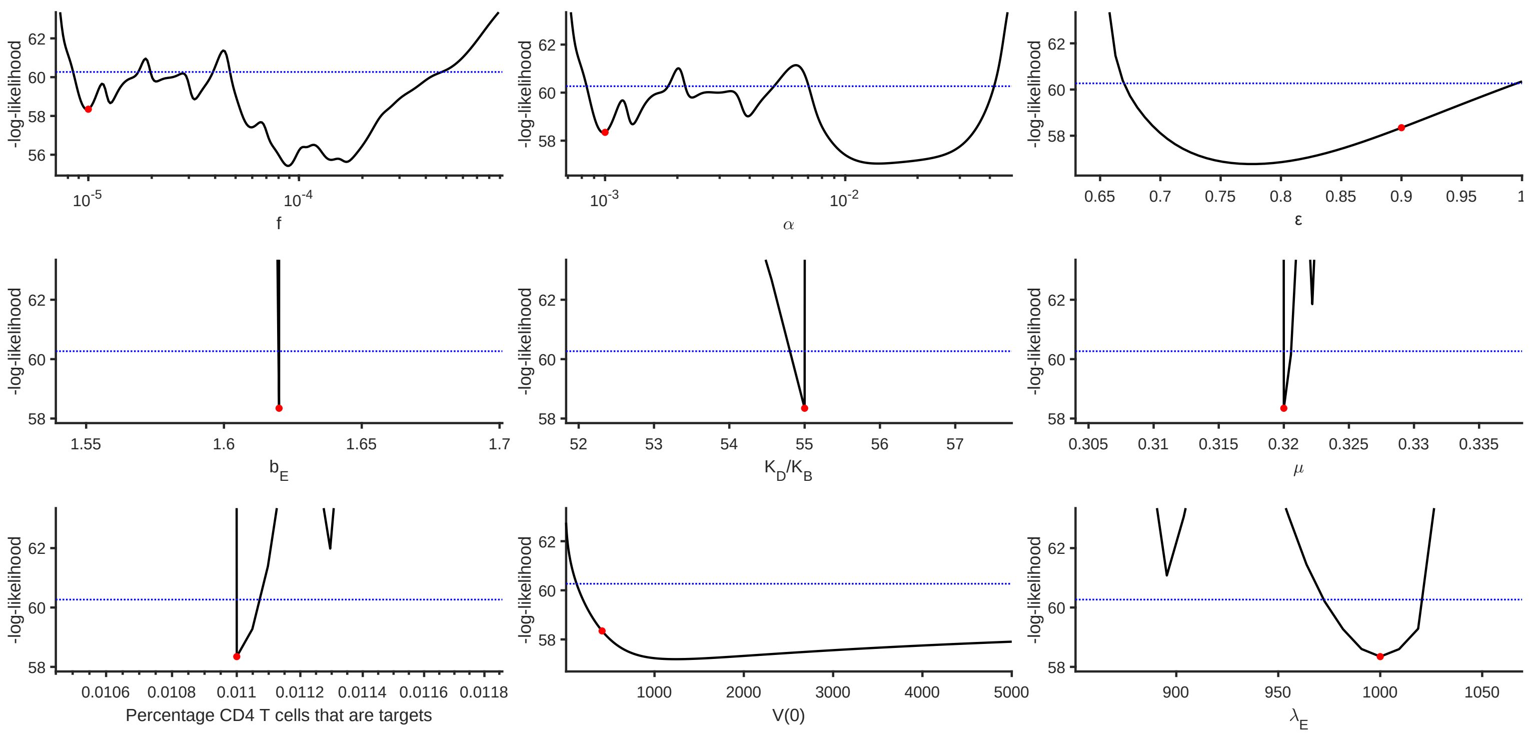

10.2 Saturated effector cell source model

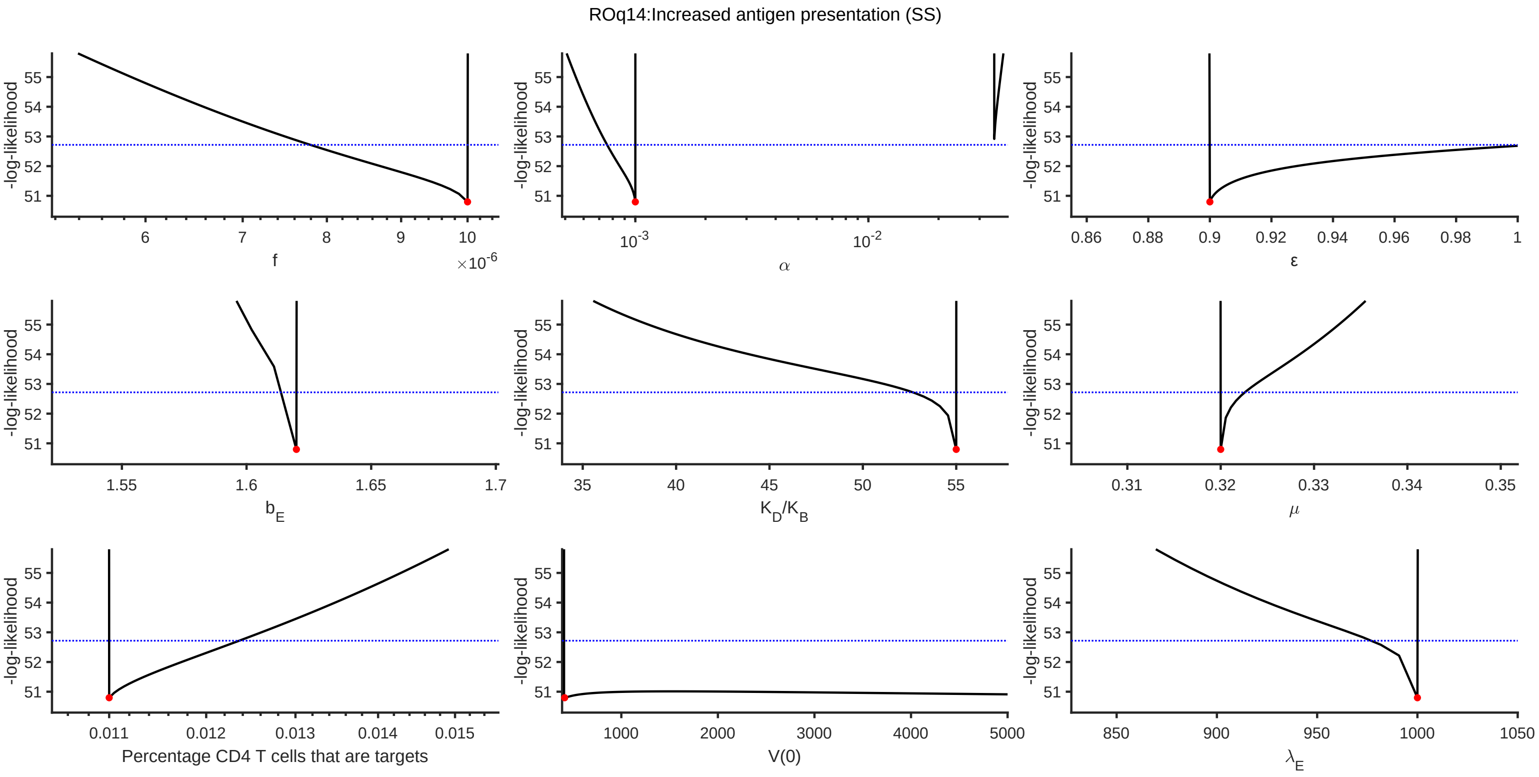

RDa15:Increased antigen presentation (SS)

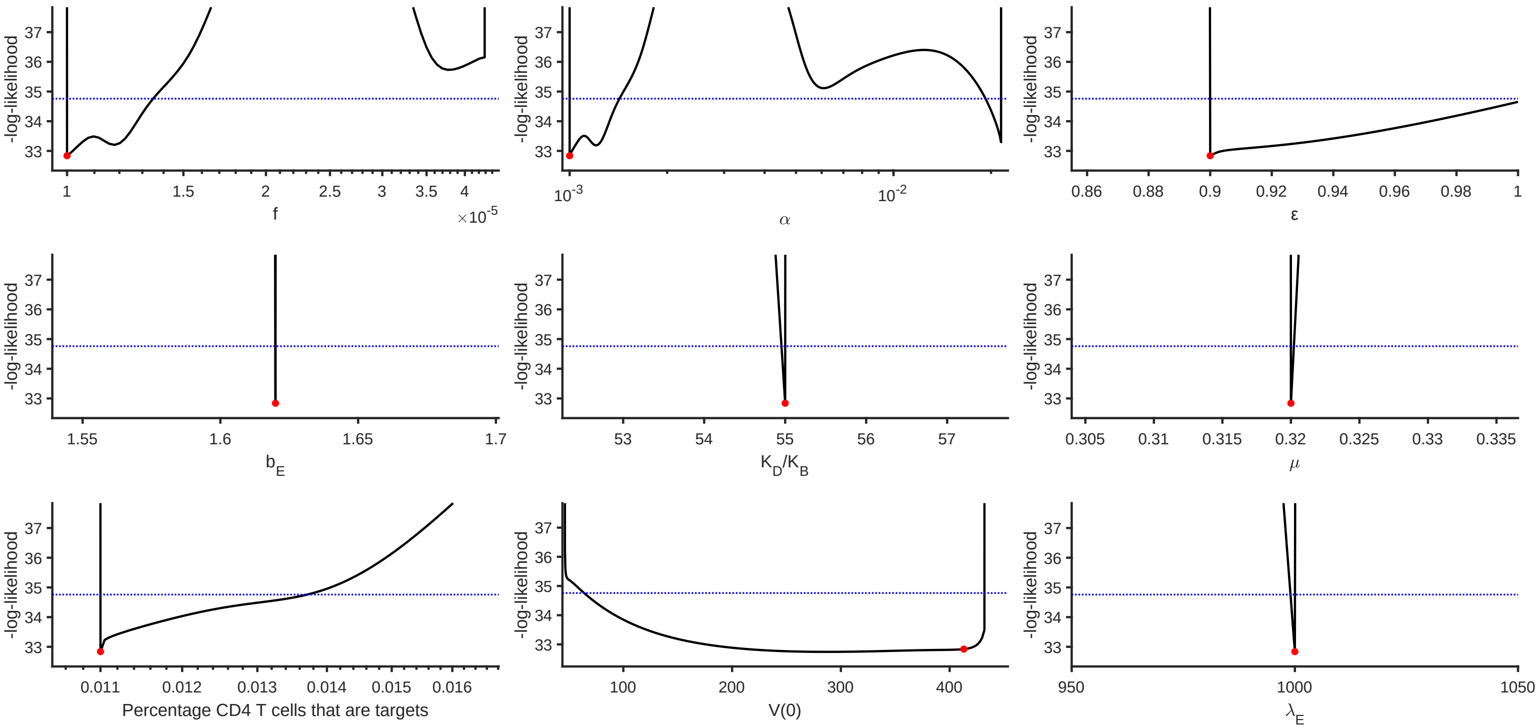

RFa15:Increased antigen presentation (SS)

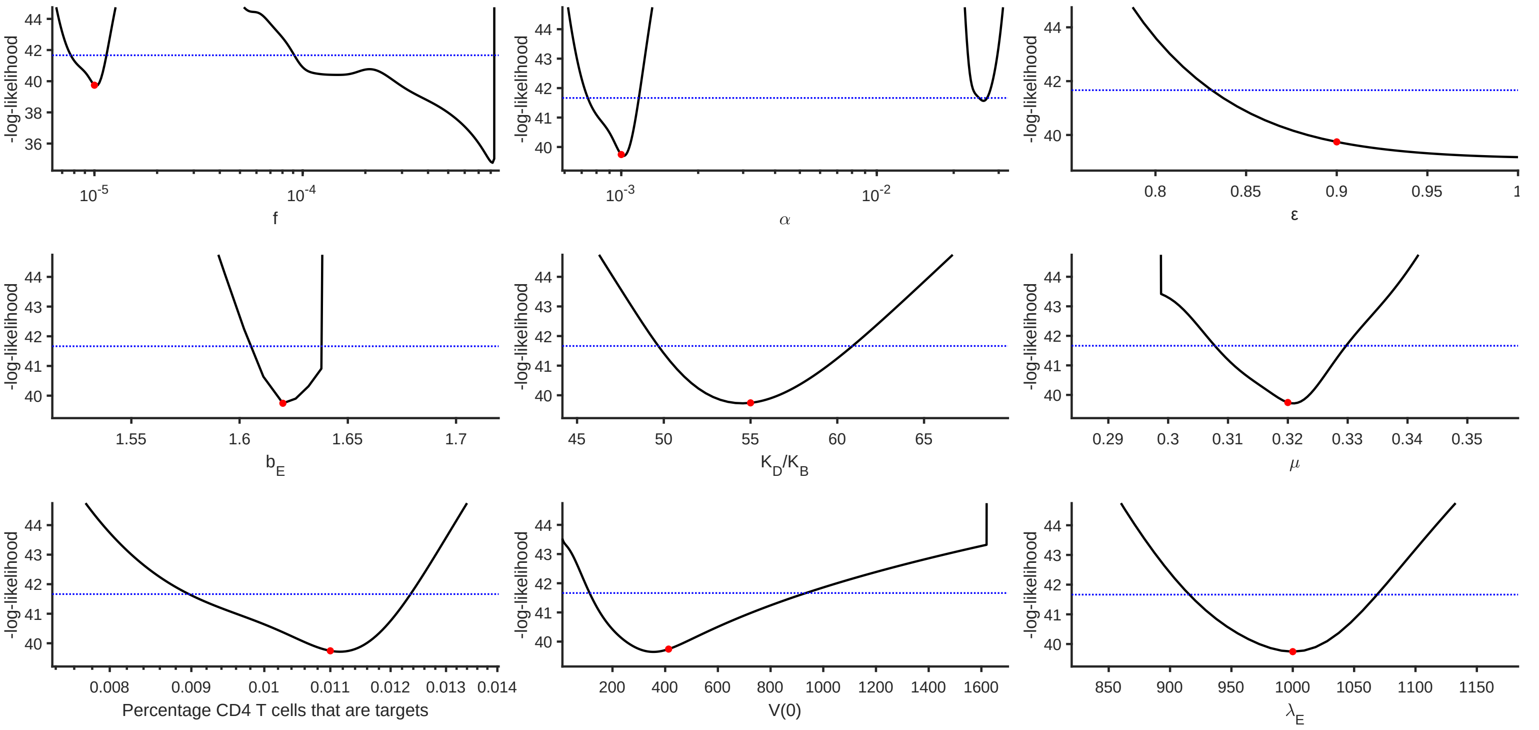

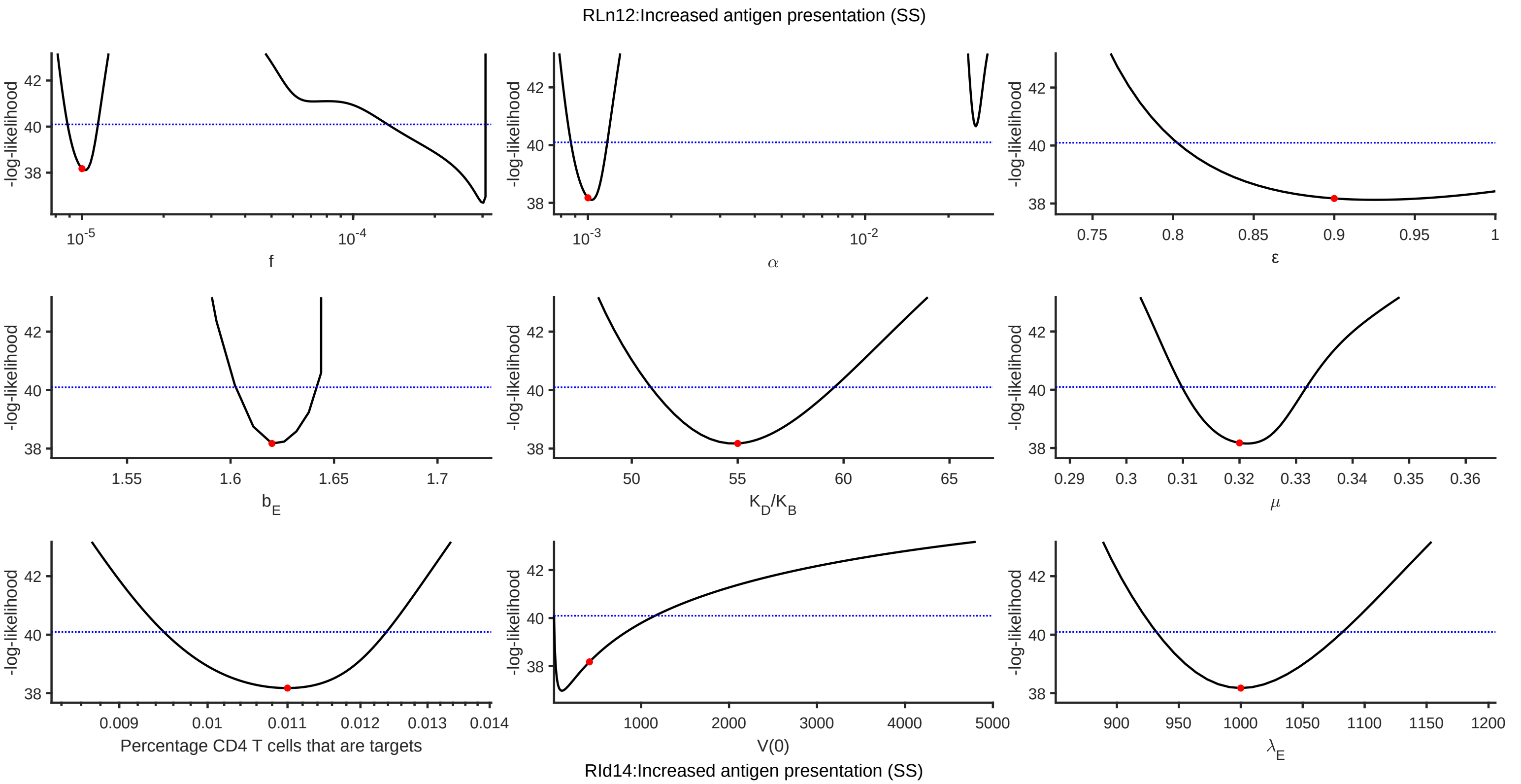
RLn12:Increased antigen presentation (SS)
-log-likelihood
f
α
ε
b_E
K_D/K_B
μ
Percentage CD4 T cells that are targets
V(0)
λ_E

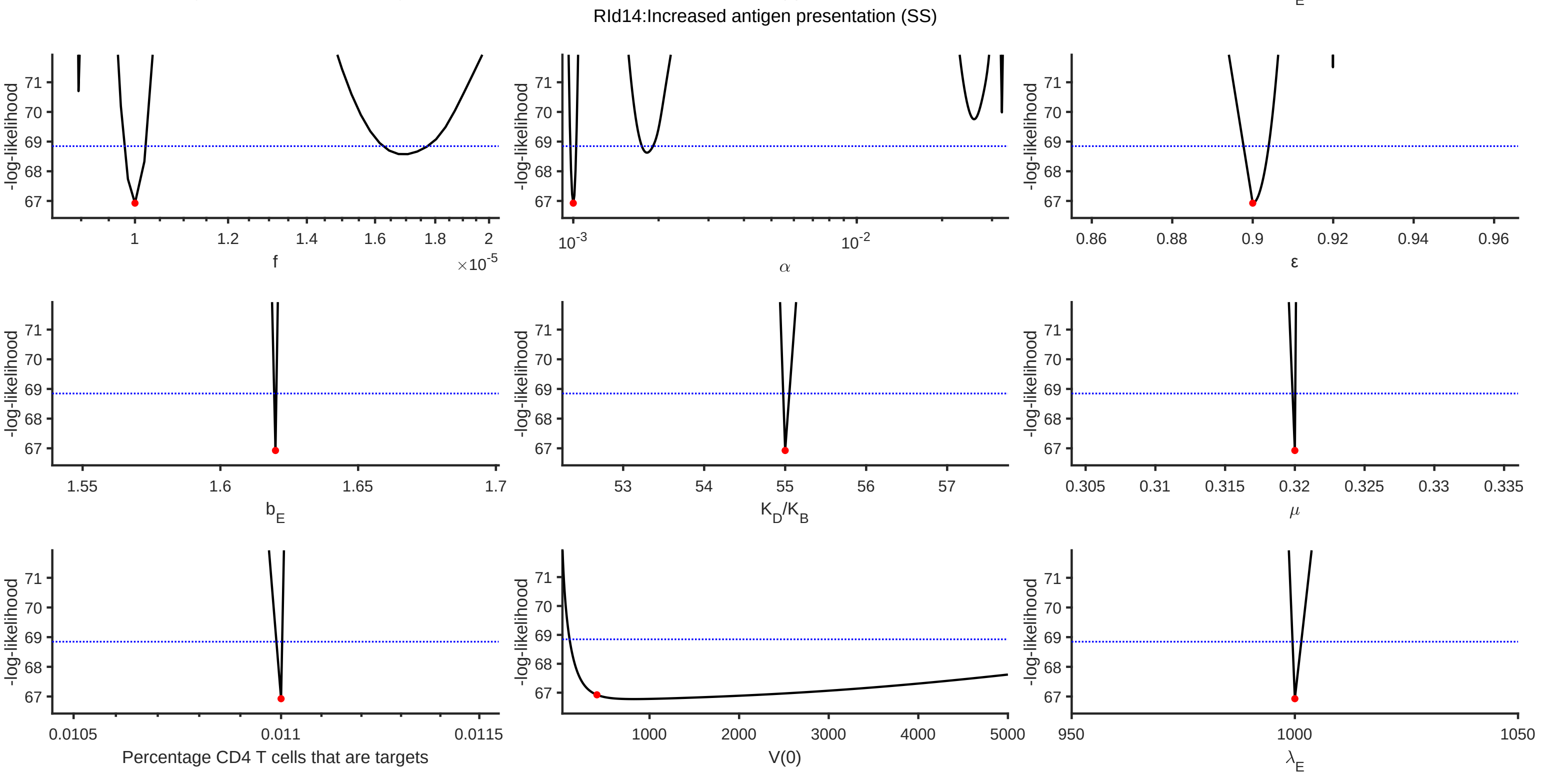
RId14:Increased antigen presentation (SS)
-log-likelihood
f
$\times 10^{-5}$
α
ε
b_E
K_D/K_B
μ
Percentage CD4 T cells that are targets
V(0)
λ_E

ROo13:Increased antigen presentation (SS)

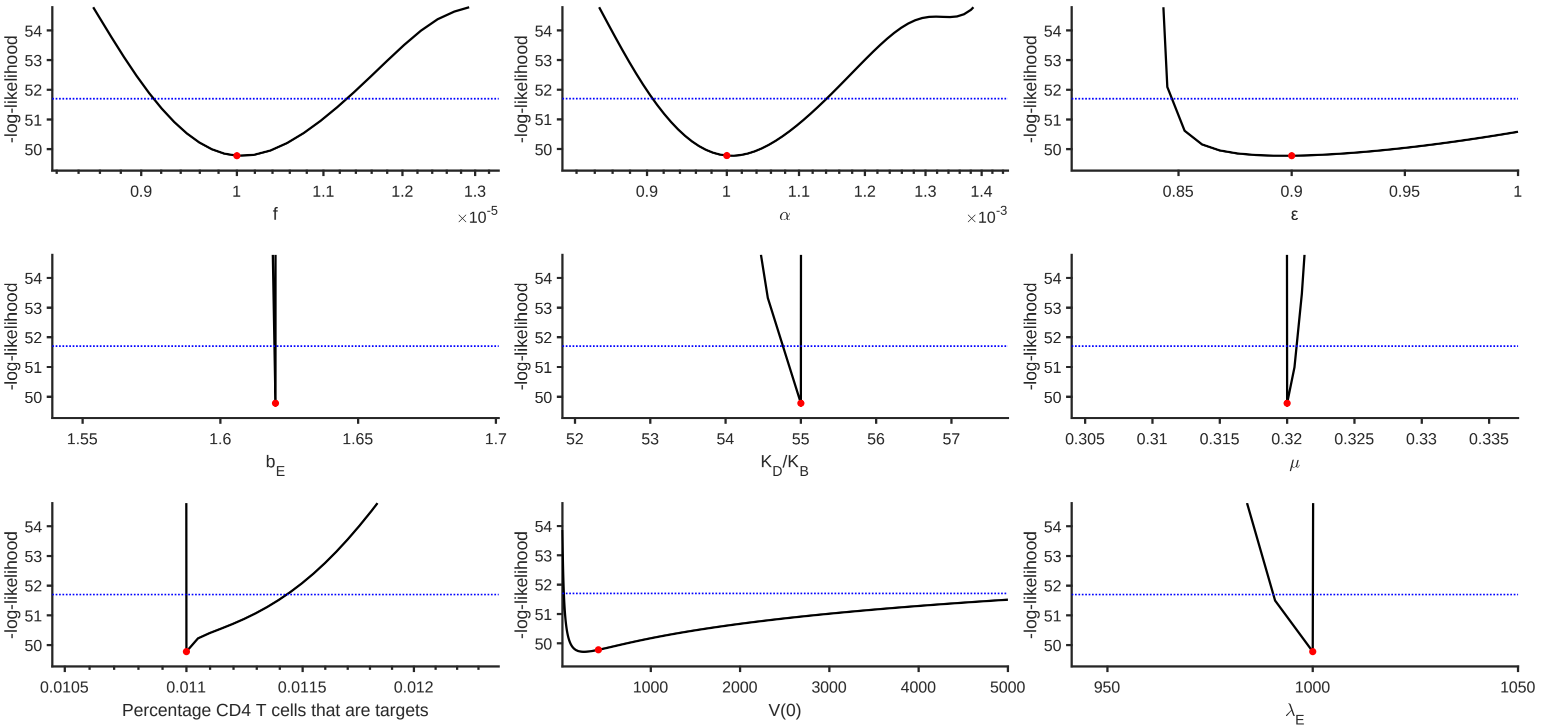

RSd14:Increased antigen presentation (SS)

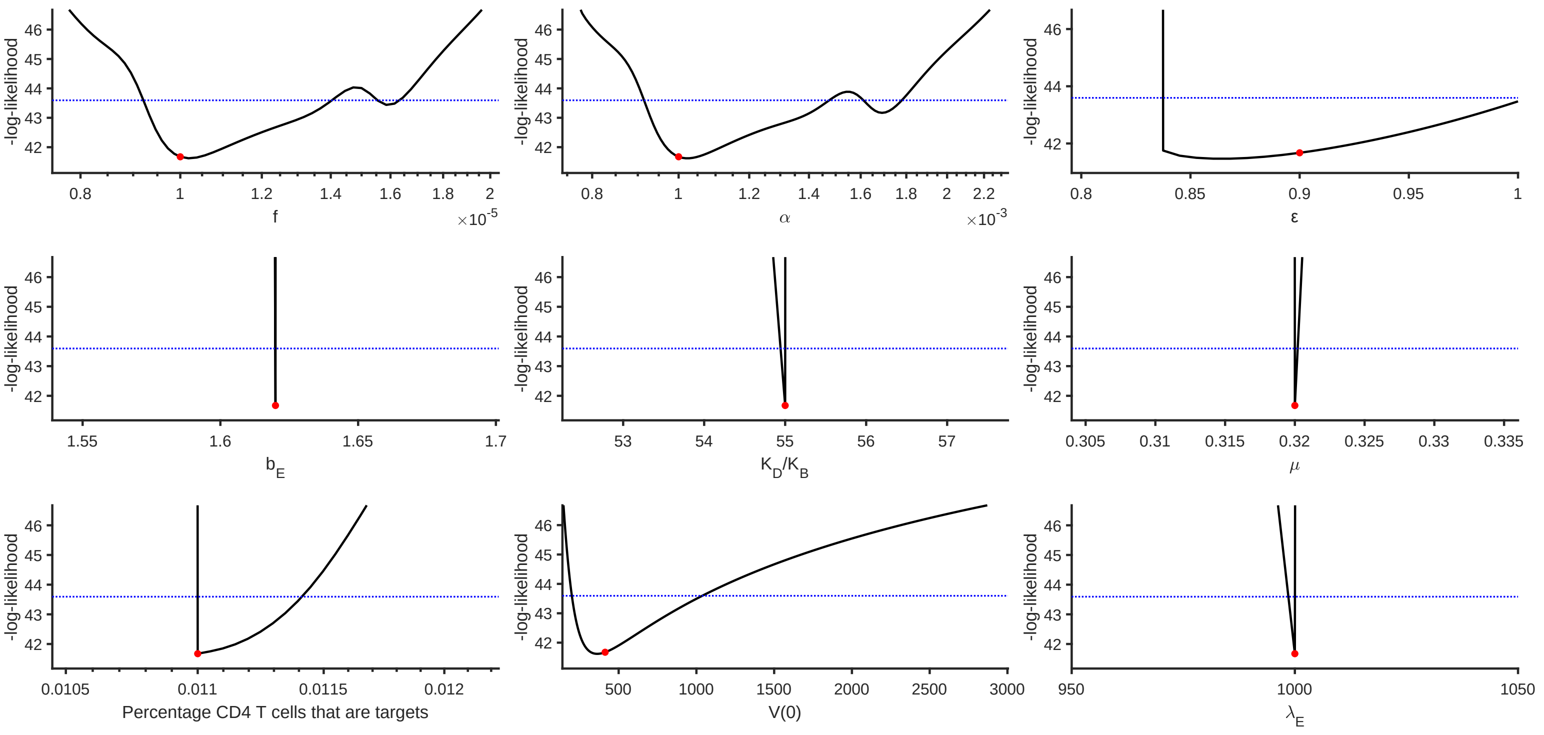

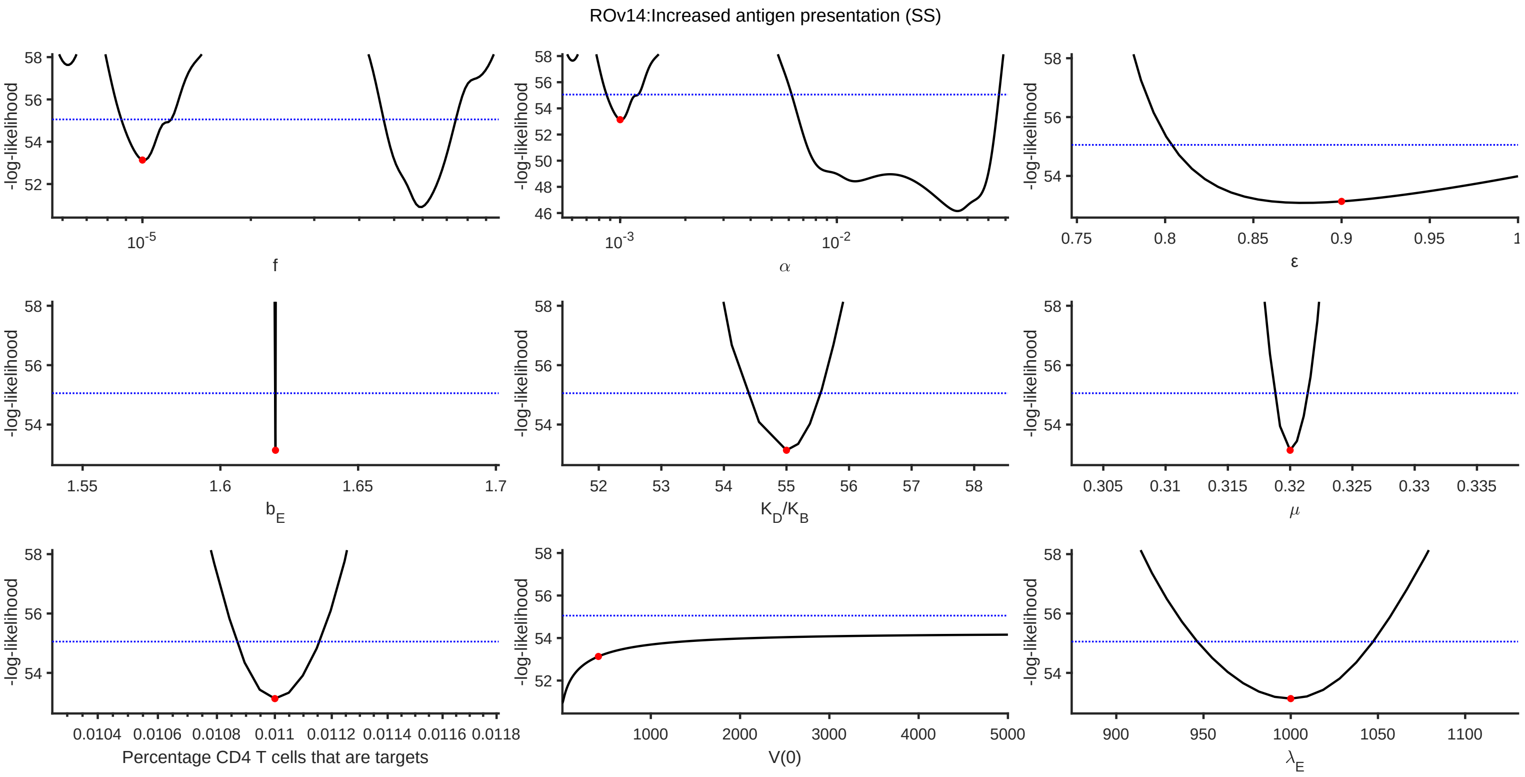
ROv14:Increased antigen presentation (SS)
-log-likelihood
f
α
ε
b_E
K_D/K_B
μ
Percentage CD4 T cells that are targets
V(0)
λ_E

10.3 Antigen presenting cell effector cell source model

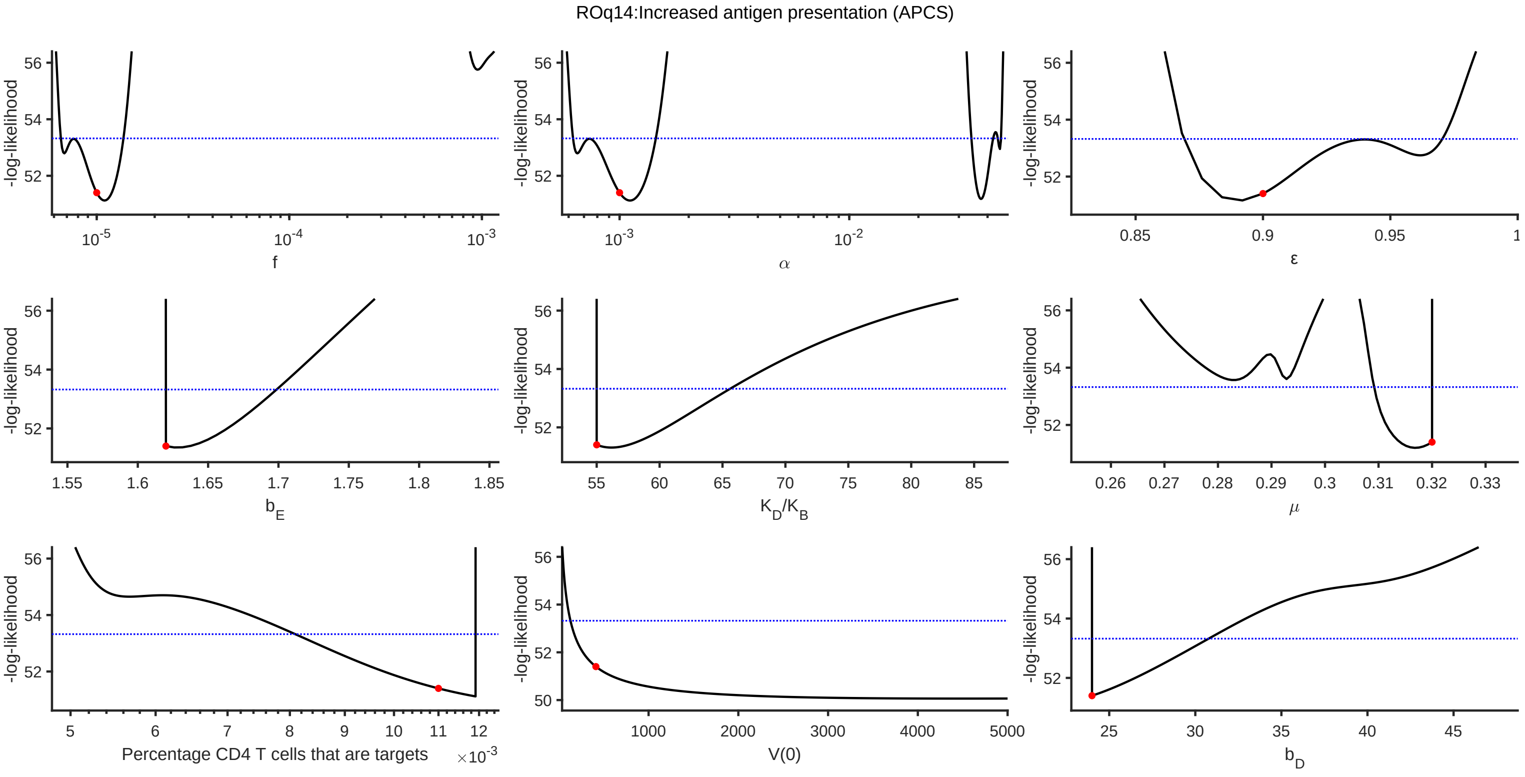

RDa15:Increased antigen presentation (APCS)

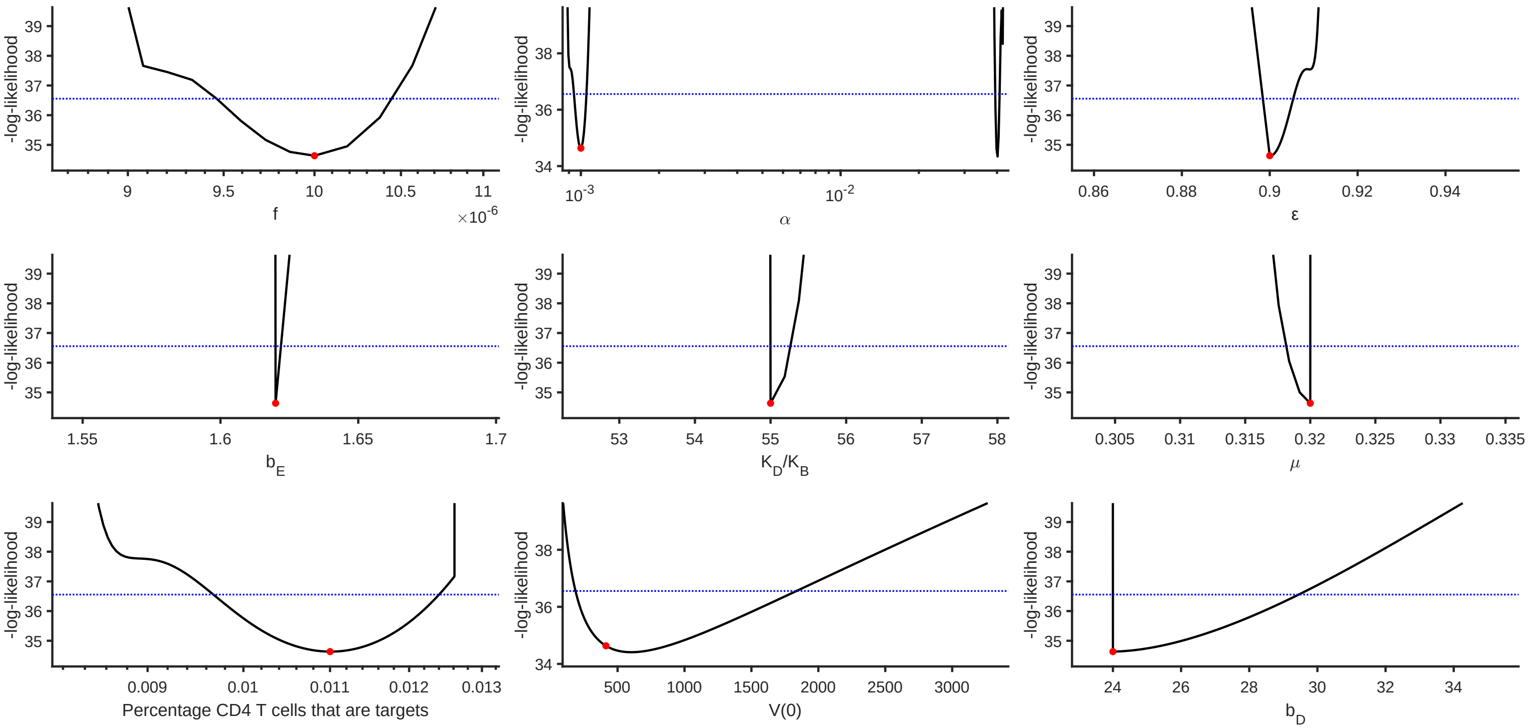

RFa15:Increased antigen presentation (APCS)

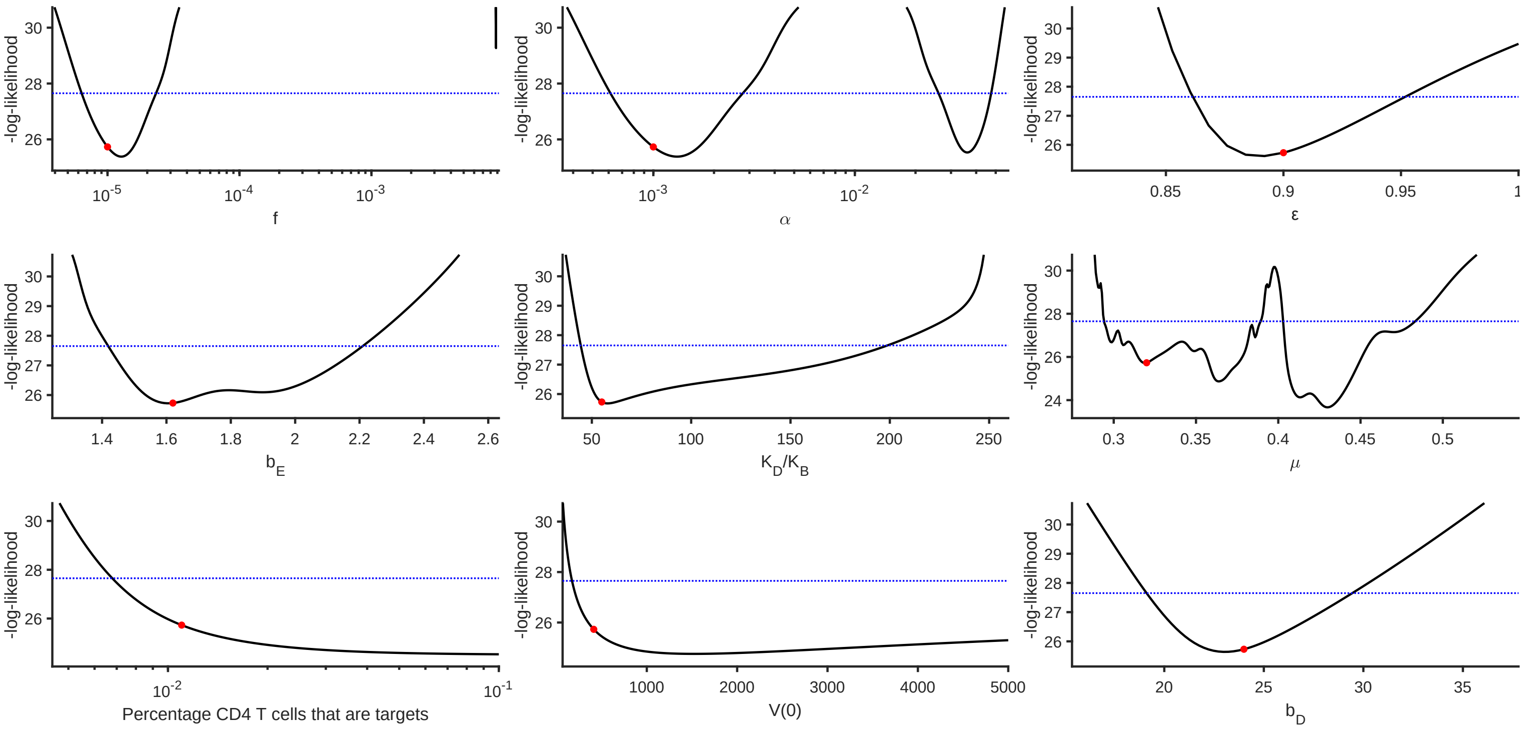

RLn12:Increased antigen presentation (APCS)

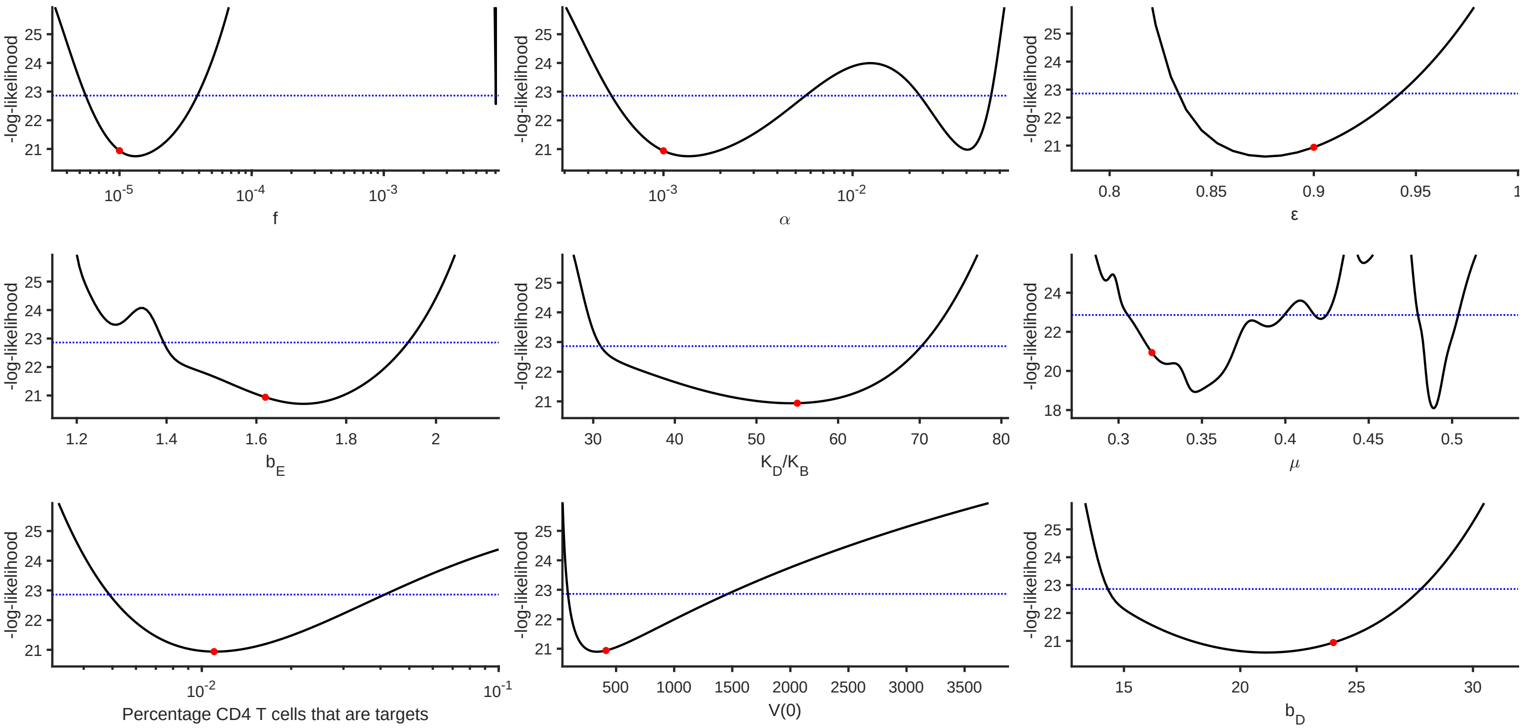

RId14:Increased antigen presentation (APCS)

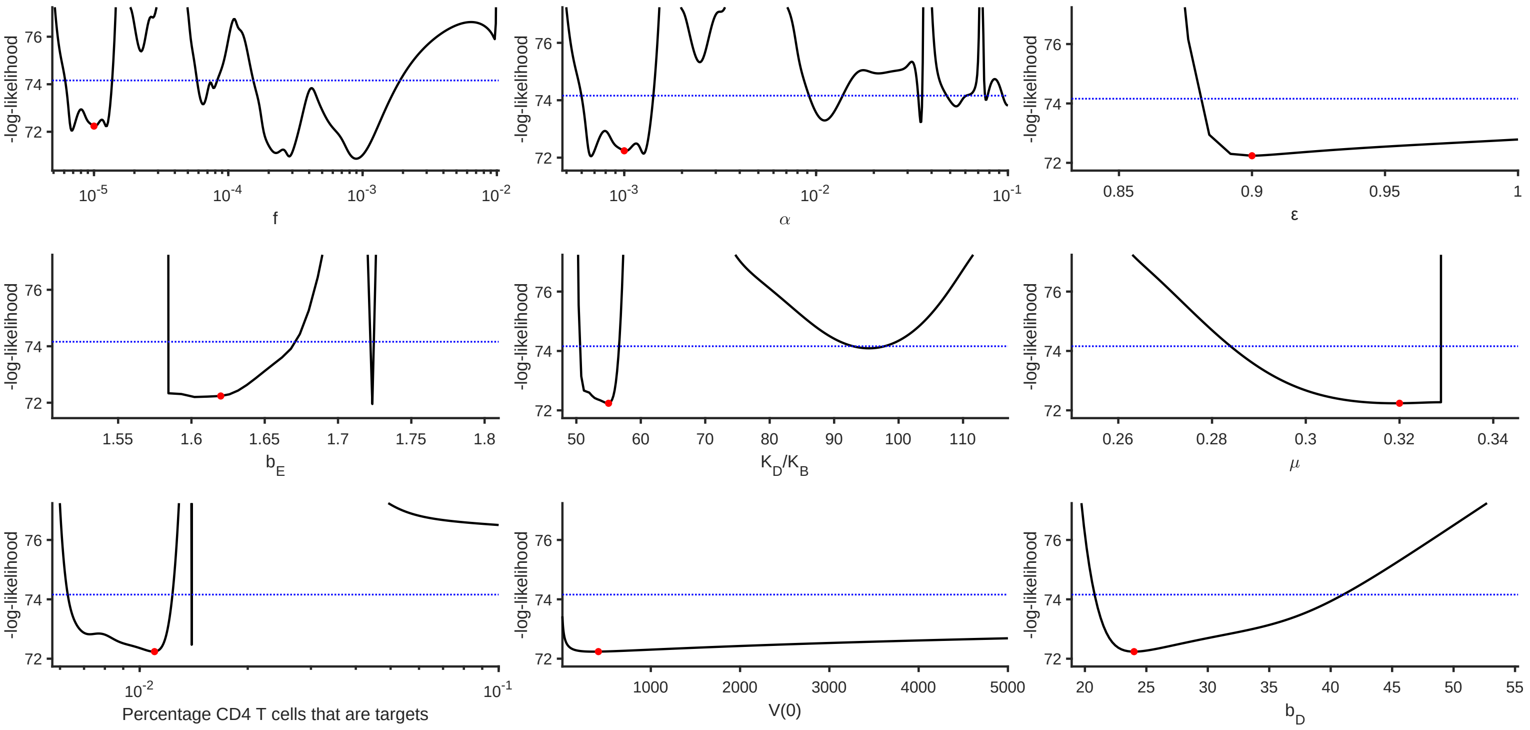

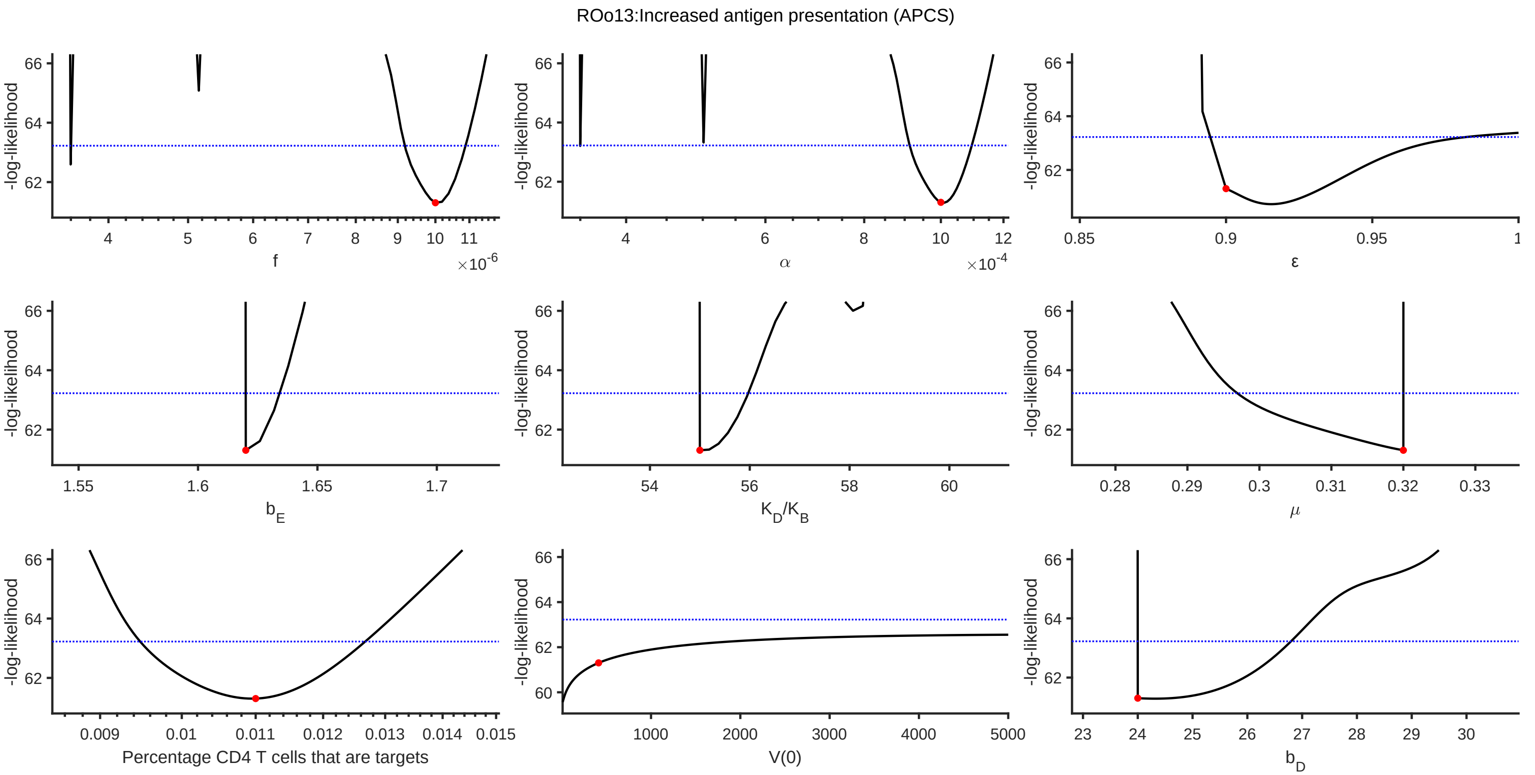
ROo13:Increased antigen presentation (APCS)
-log-likelihood
f
×10^-6
α
×10^-4
ε
b_E
K_D/K_B
μ
Percentage CD4 T cells that are targets
V(0)
b_D

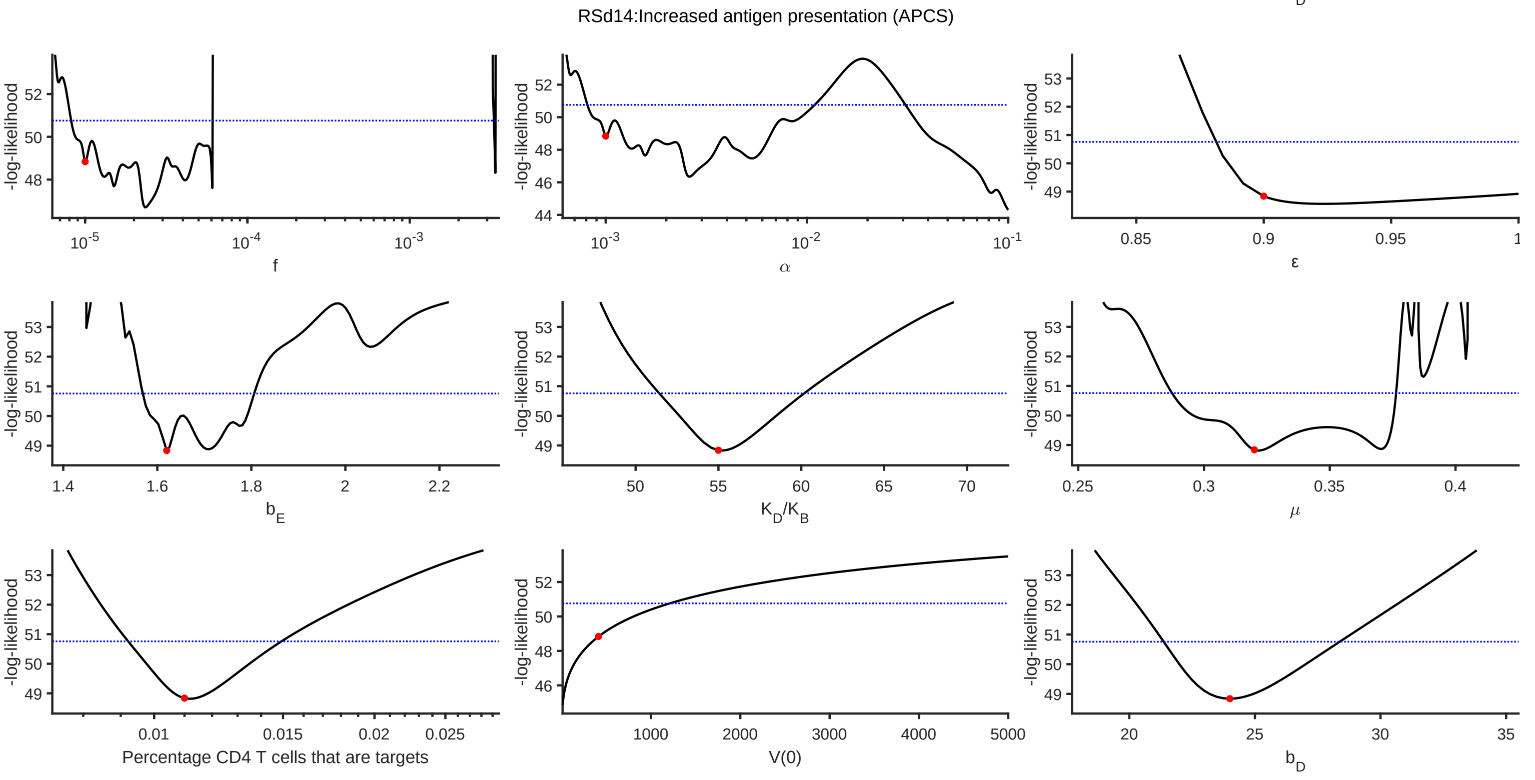
RSd14:Increased antigen presentation (APCS)
-log-likelihood
f
α
ε
b_E
K_D/K_B
μ
Percentage CD4 T cells that are targets
V(0)
b_D

ROv14:Increased antigen presentation (APCS)

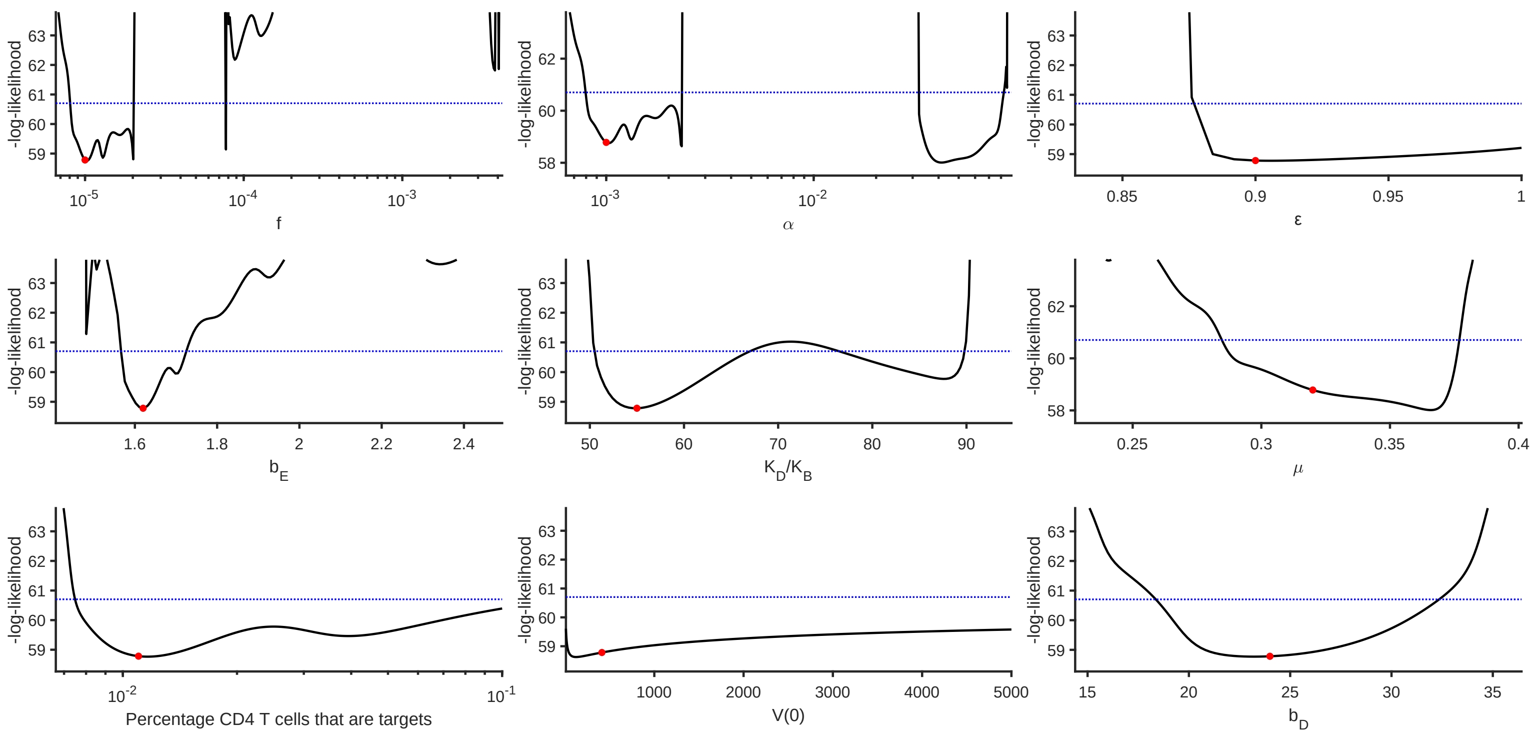

11 Likelihood profiles: Increased antigen presentation with increased viral clearance

11.1 Baseline effector cell source model

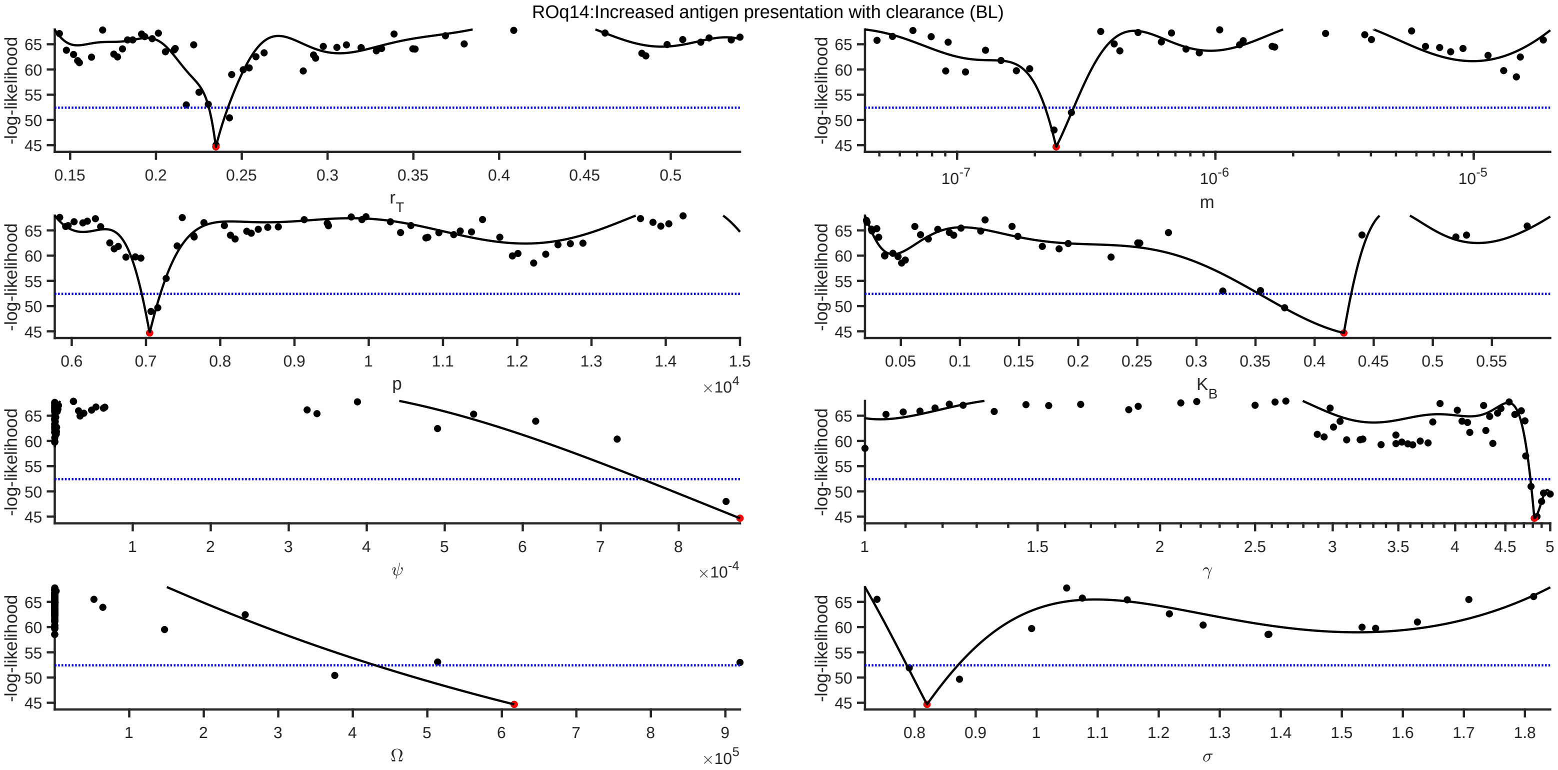

RDa15:Increased antigen presentation with clearance (BL)

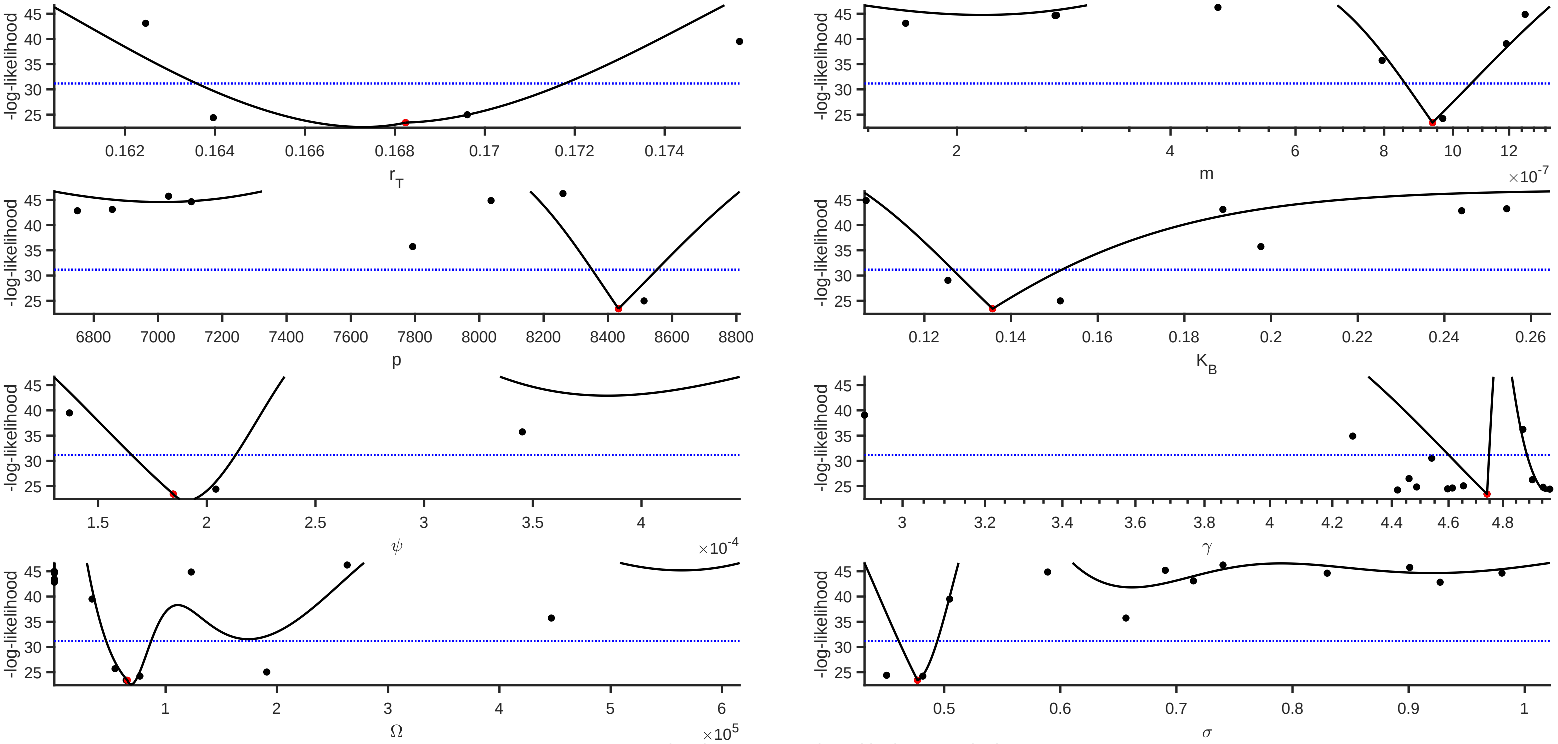

RFa15:Increased antigen presentation with clearance (BL)

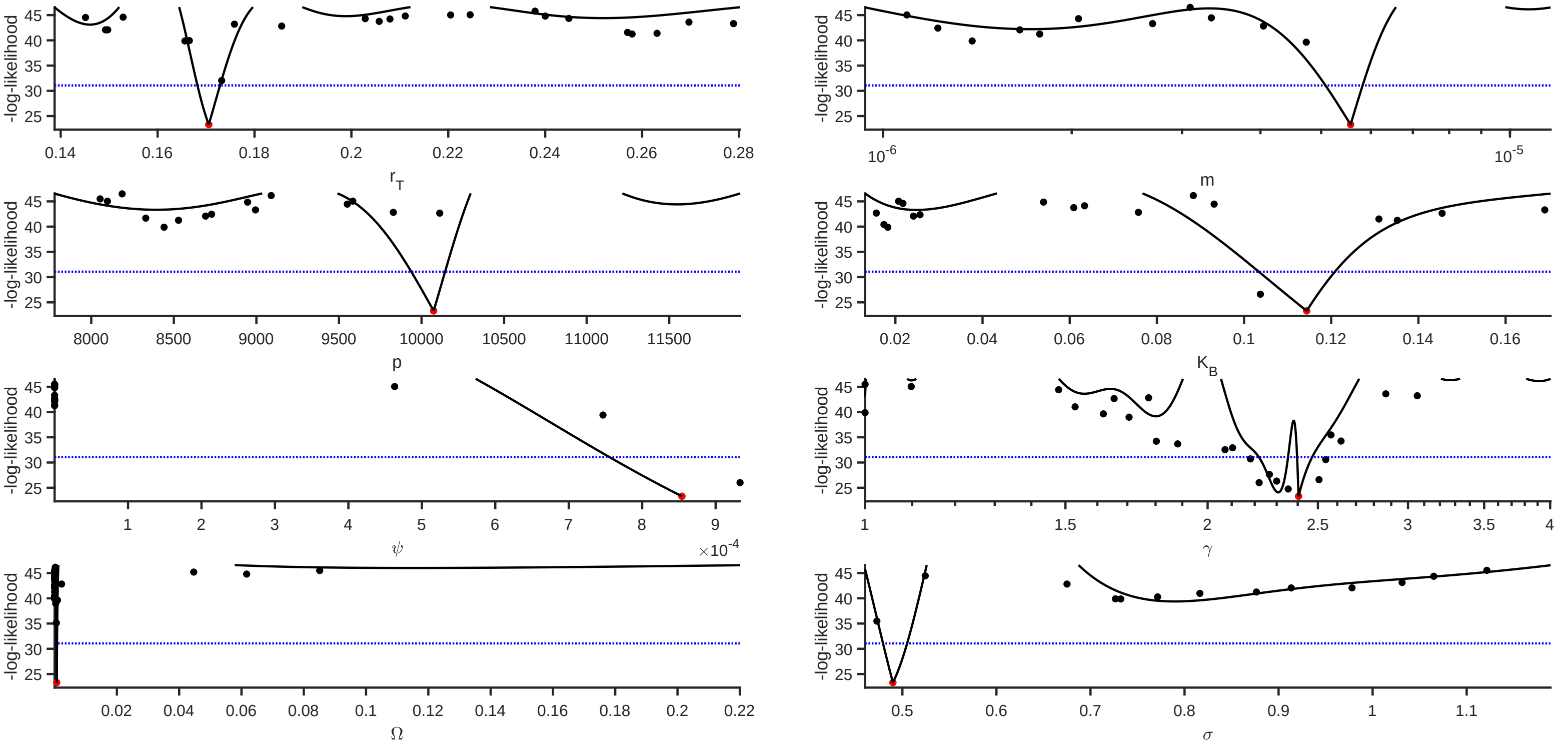

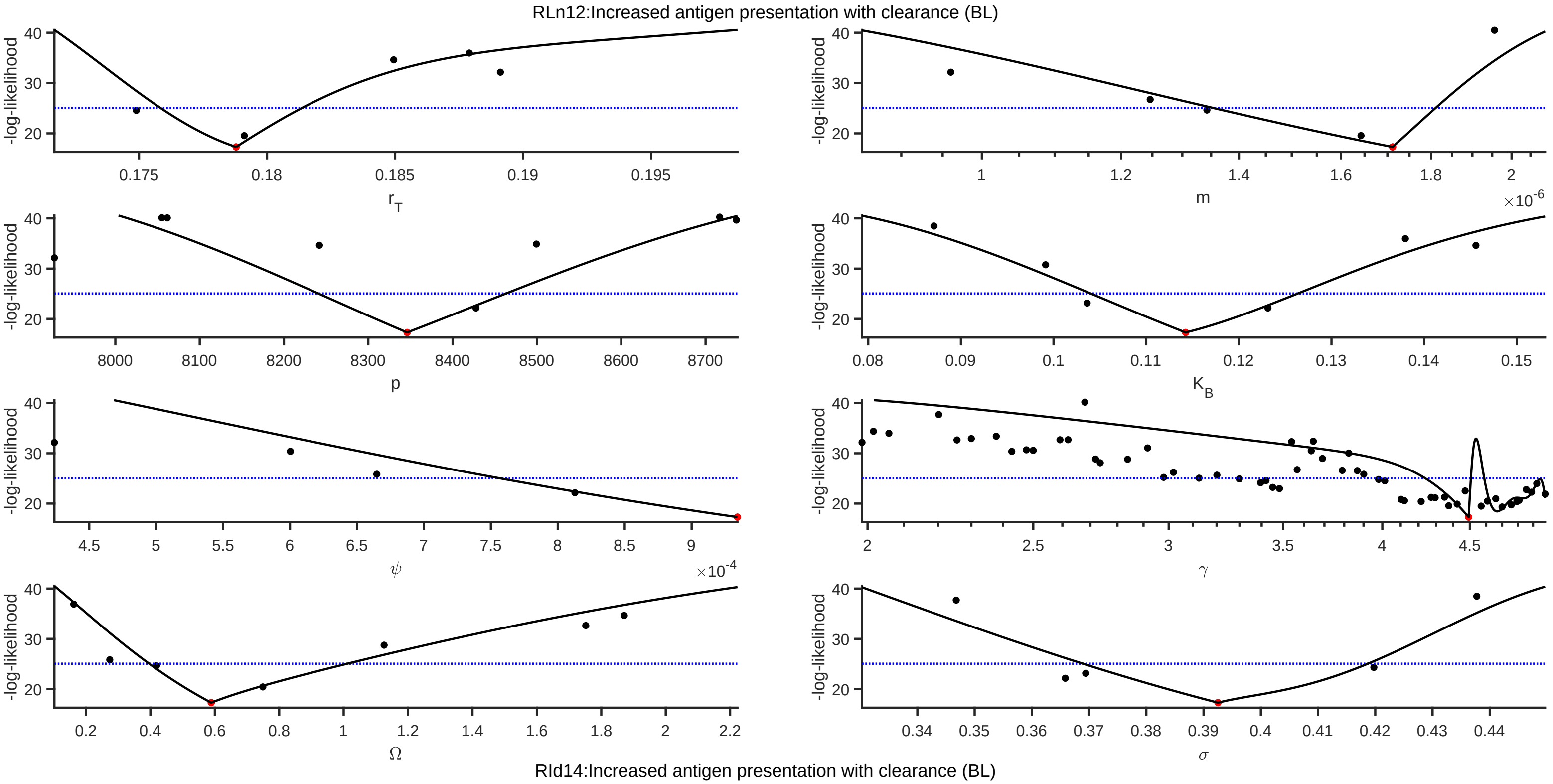
RLn12:Increased antigen presentation with clearance (BL)
-log-likelihood
r_T
m
p
K_B
ψ
γ
Ω
σ

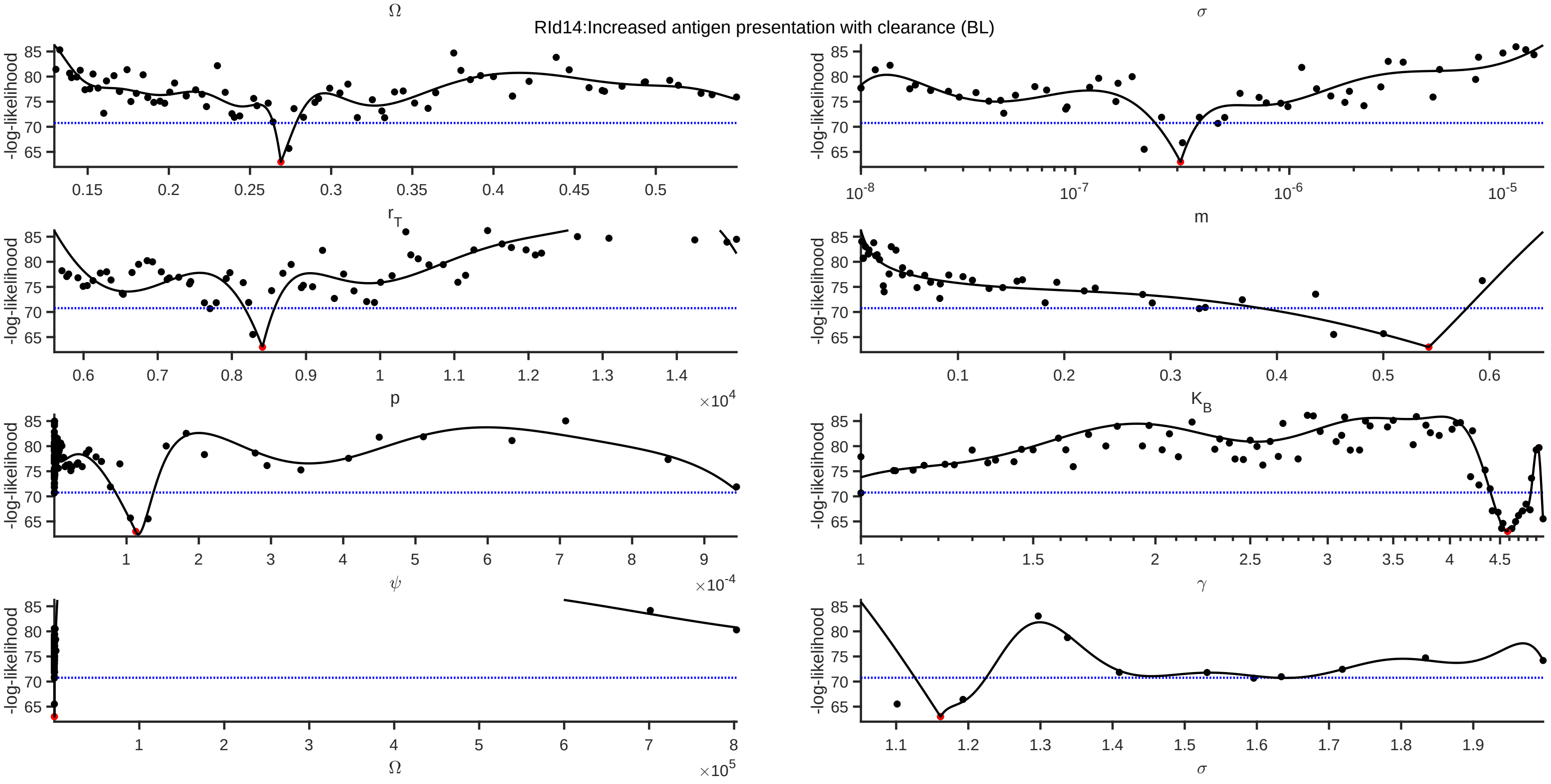
RId14:Increased antigen presentation with clearance (BL)
-log-likelihood
r_T
m
p
K_B
ψ
γ
Ω
σ

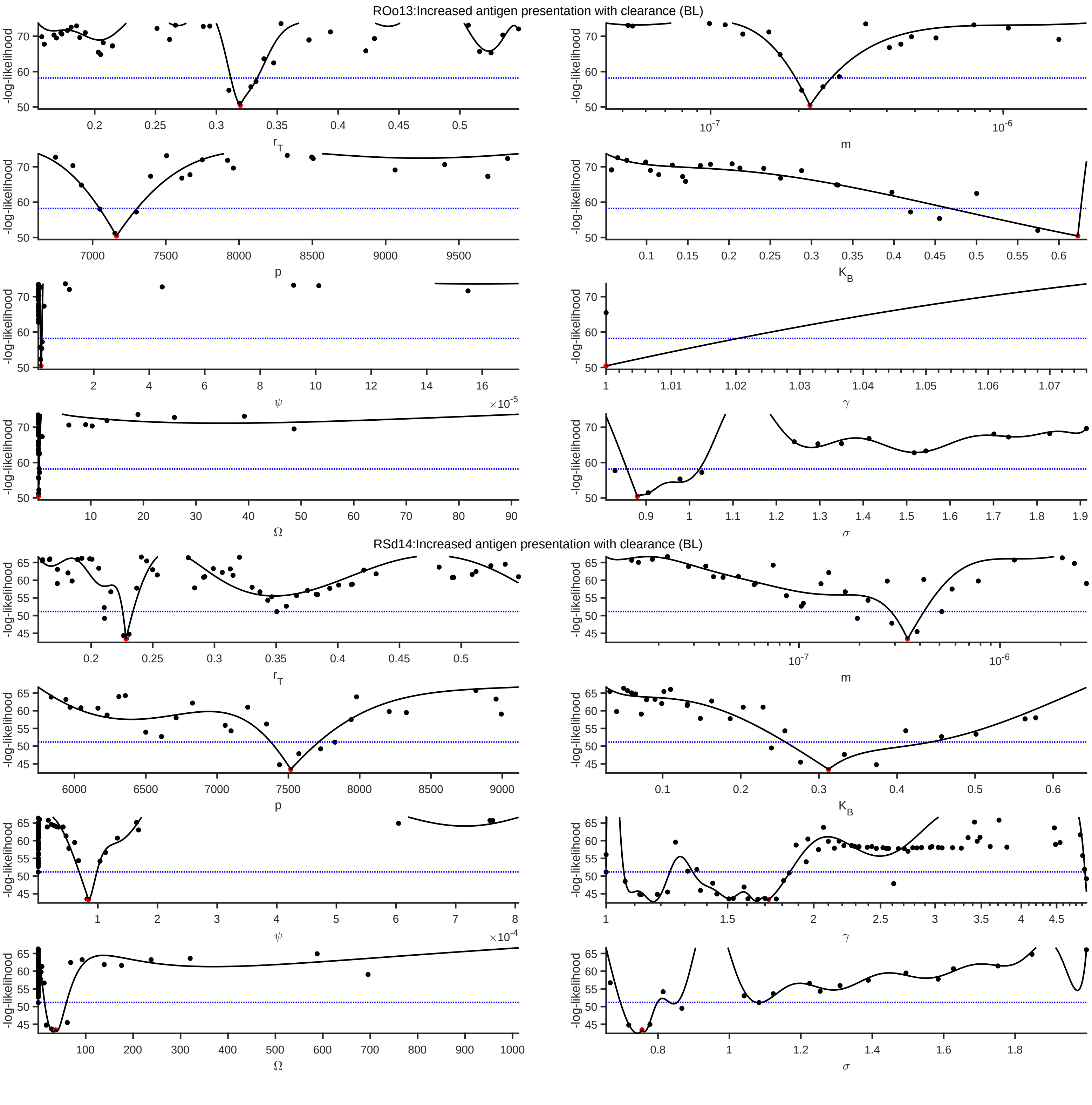
ROo13:Increased antigen presentation with clearance (BL)
-log-likelihood
r_T
m
p
K_B
ψ
γ
Ω
σ
$\times 10^{-5}$
RSd14:Increased antigen presentation with clearance (BL)
-log-likelihood
r_T
m
p
K_B
ψ
γ
Ω
σ
$\times 10^{-4}$

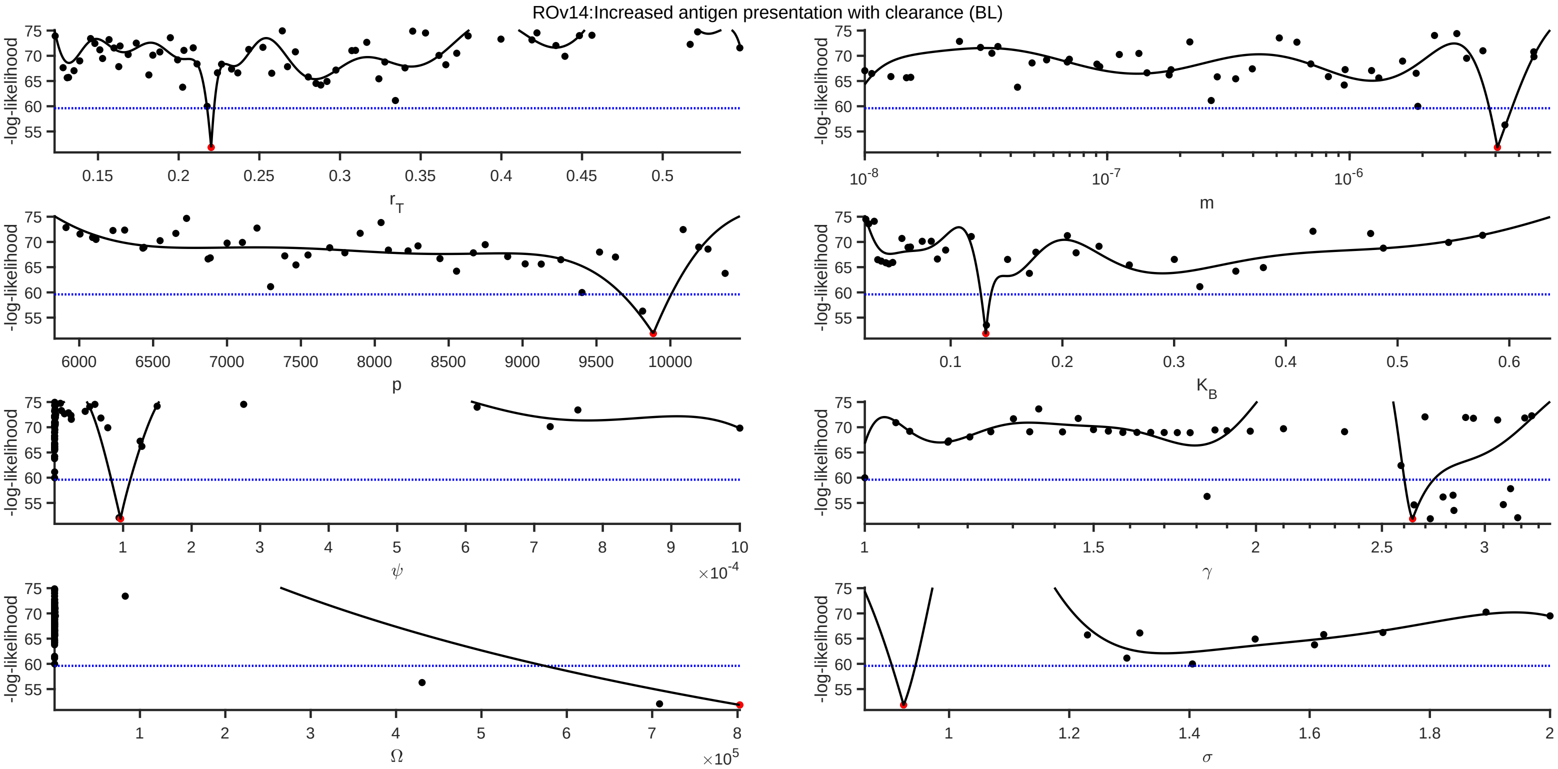
ROv14:Increased antigen presentation with clearance (BL)
-log-likelihood
r_T
m
p
K_B
ψ
γ
Ω
σ
×10^-4
×10^5

11.2 Saturated effector cell source model

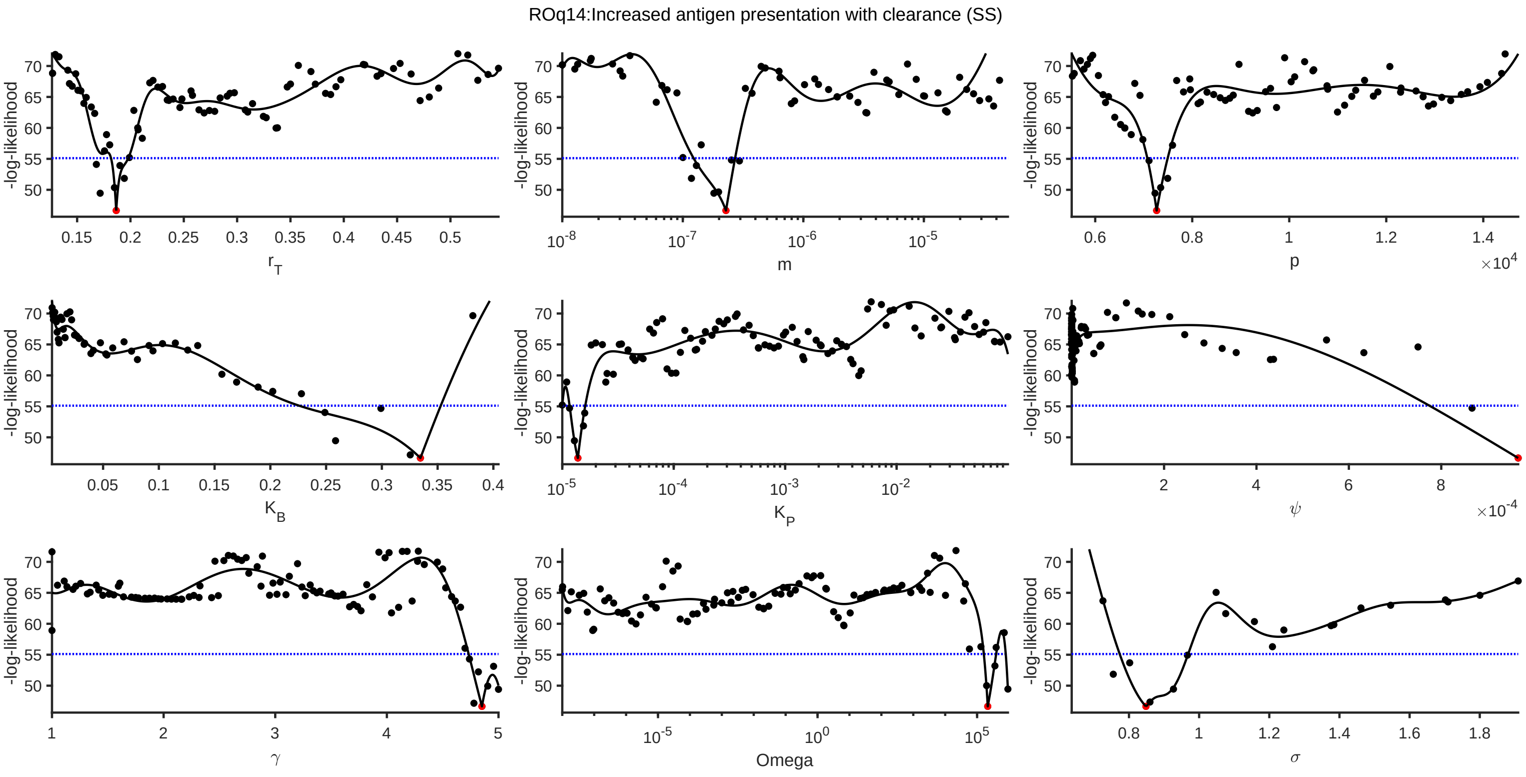

RDa15:Increased antigen presentation with clearance (SS)

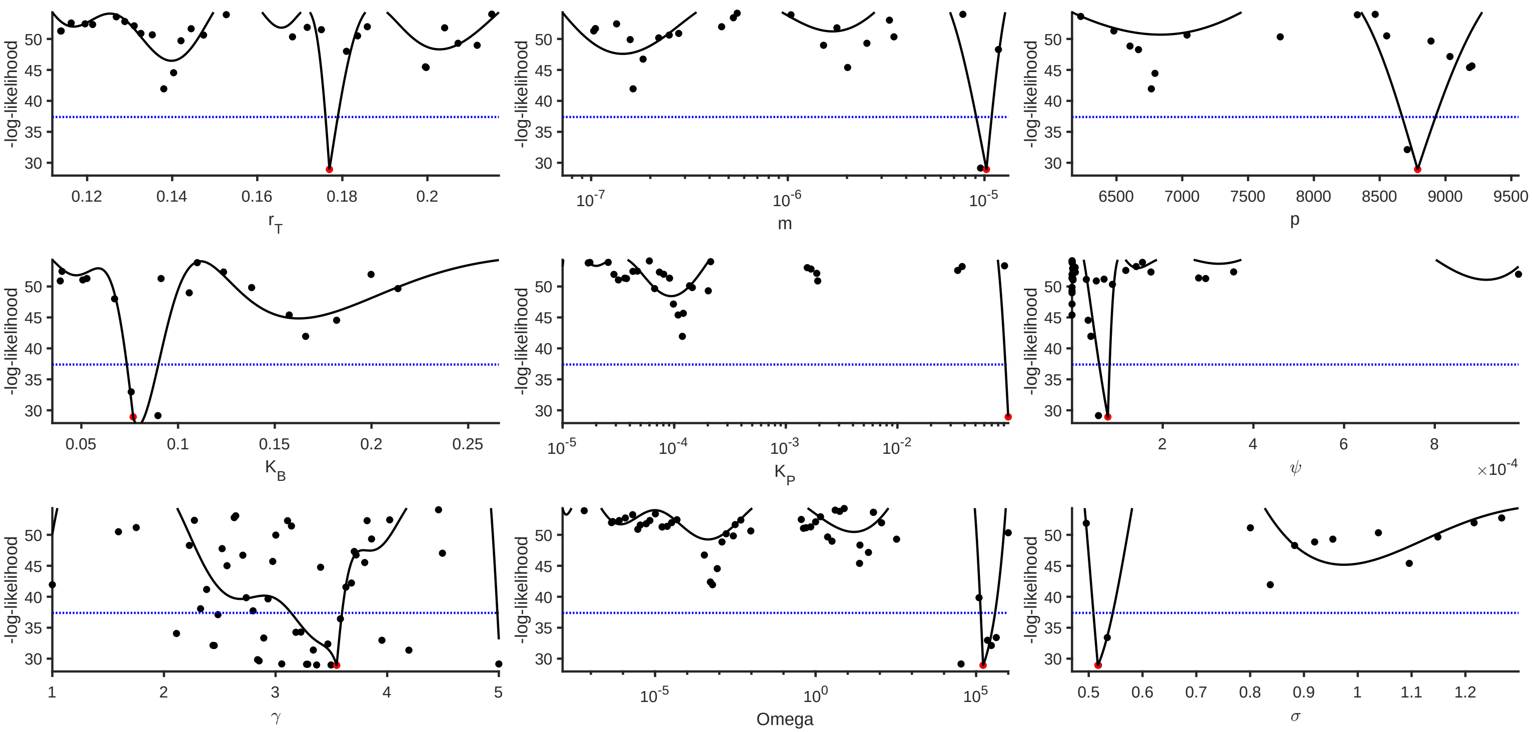

RFa15:Increased antigen presentation with clearance (SS)

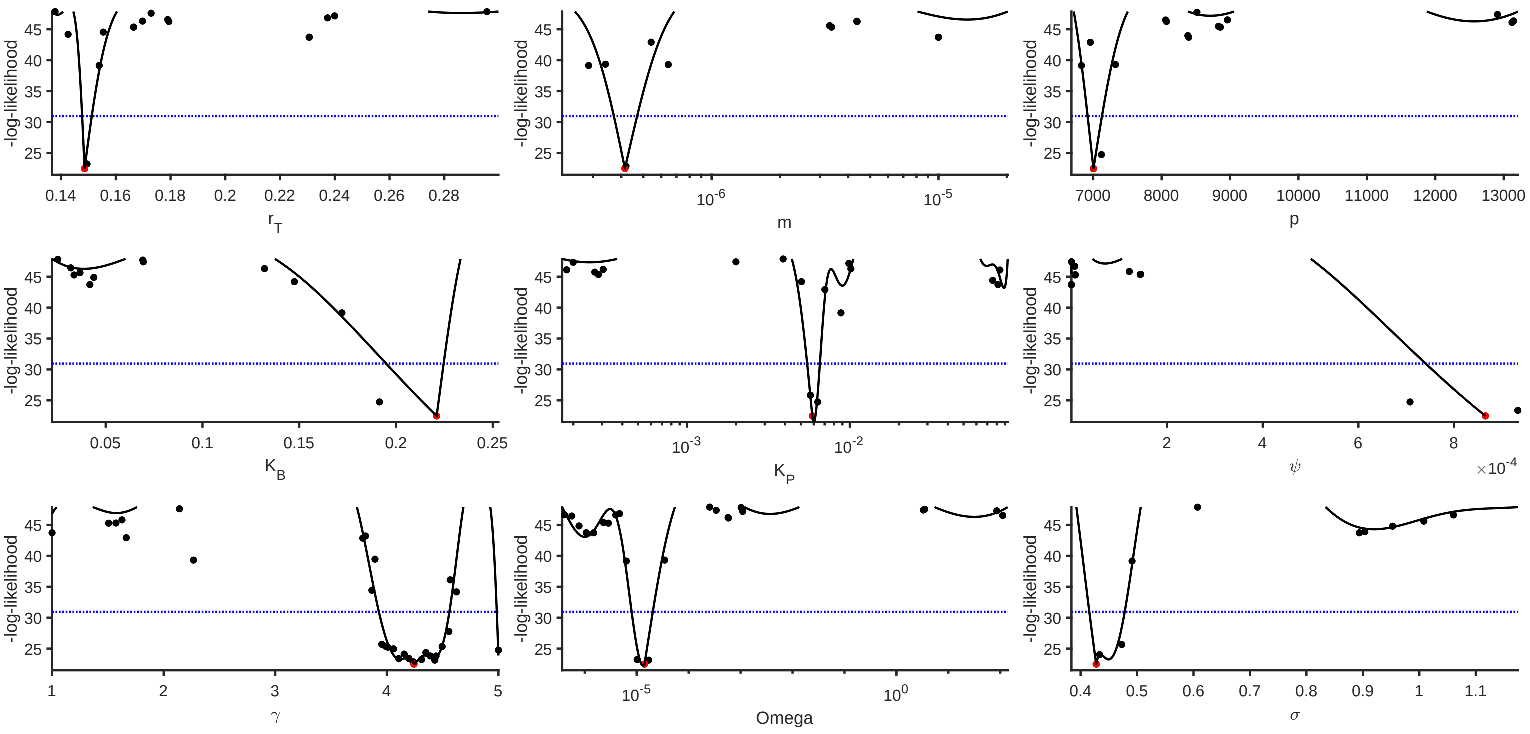

RLn12:Increased antigen presentation with clearance (SS)

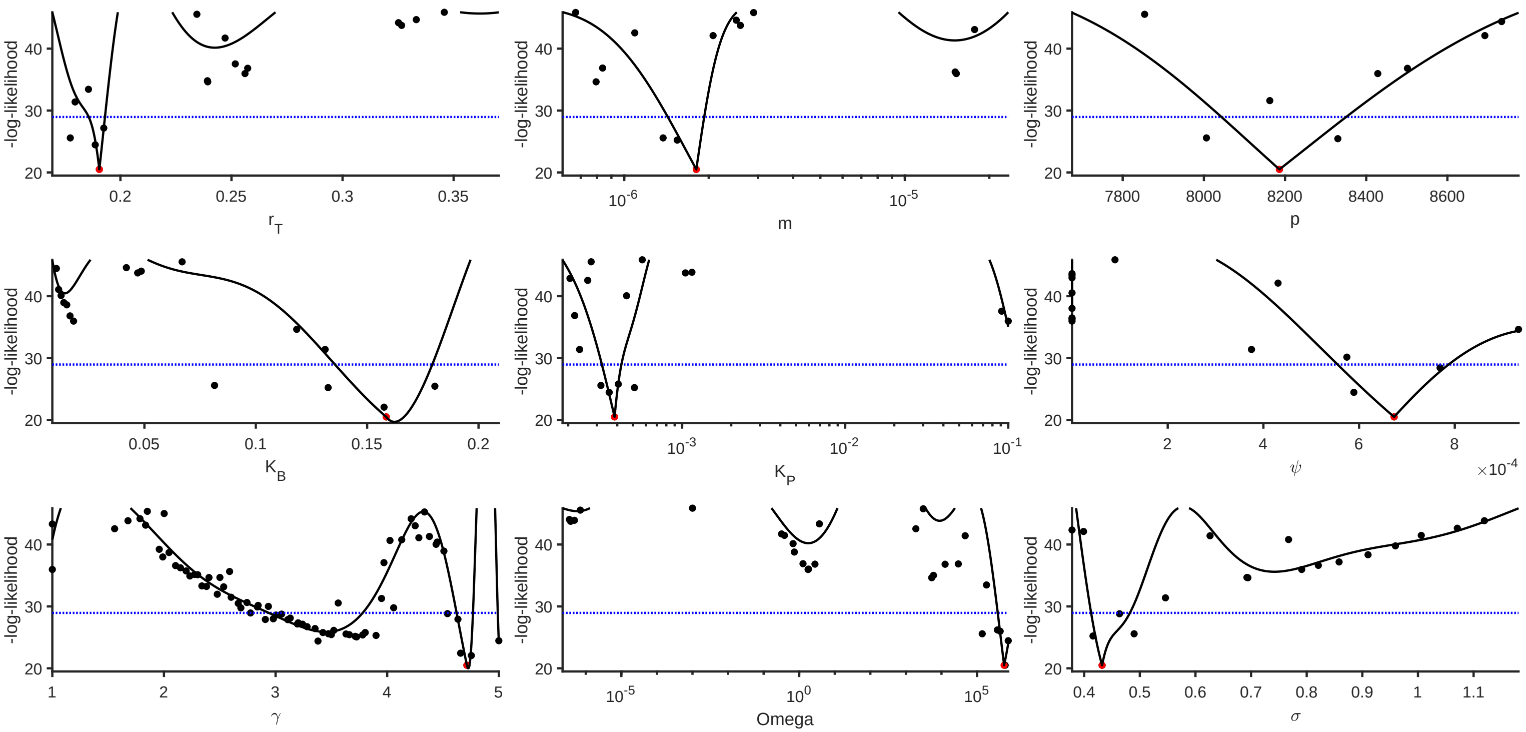

RId14:Increased antigen presentation with clearance (SS)

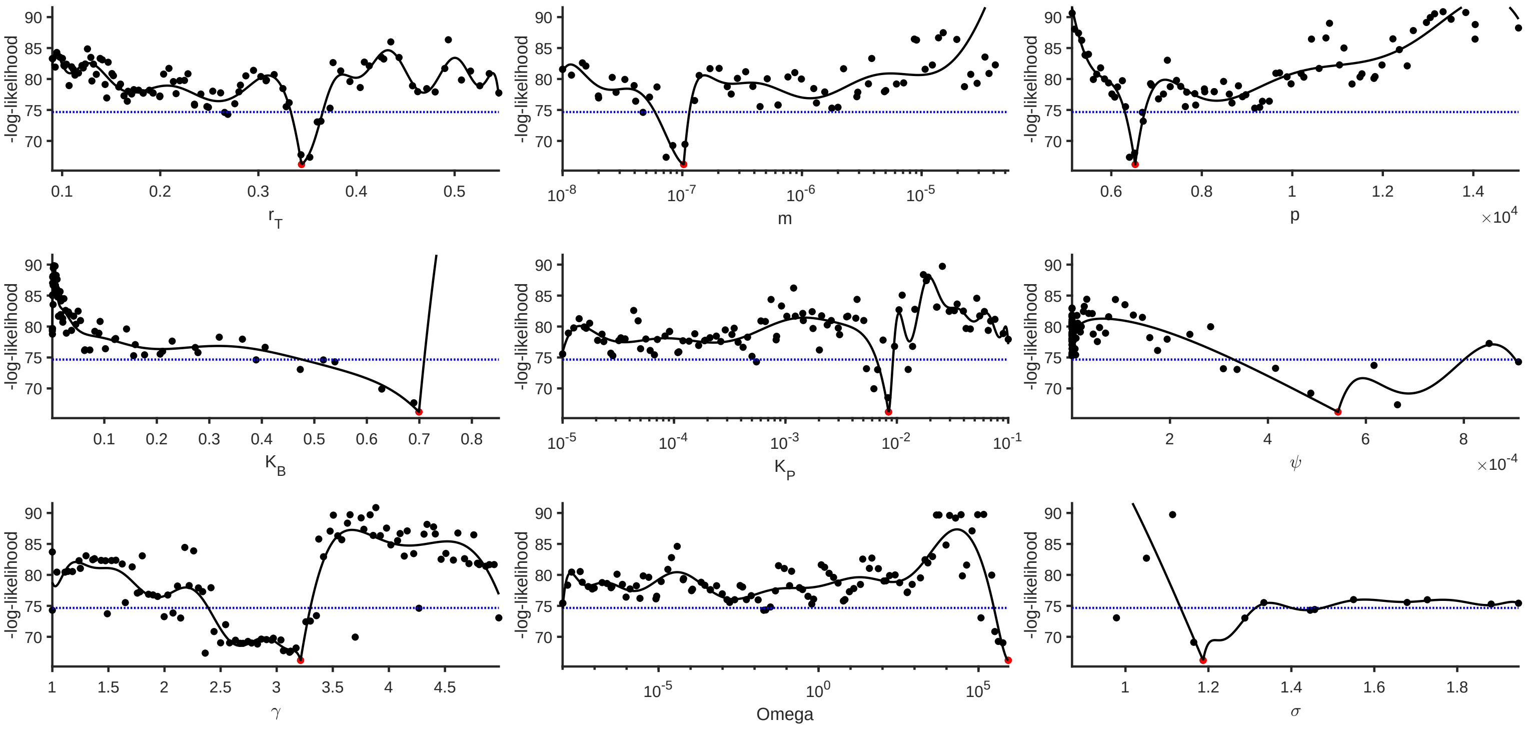

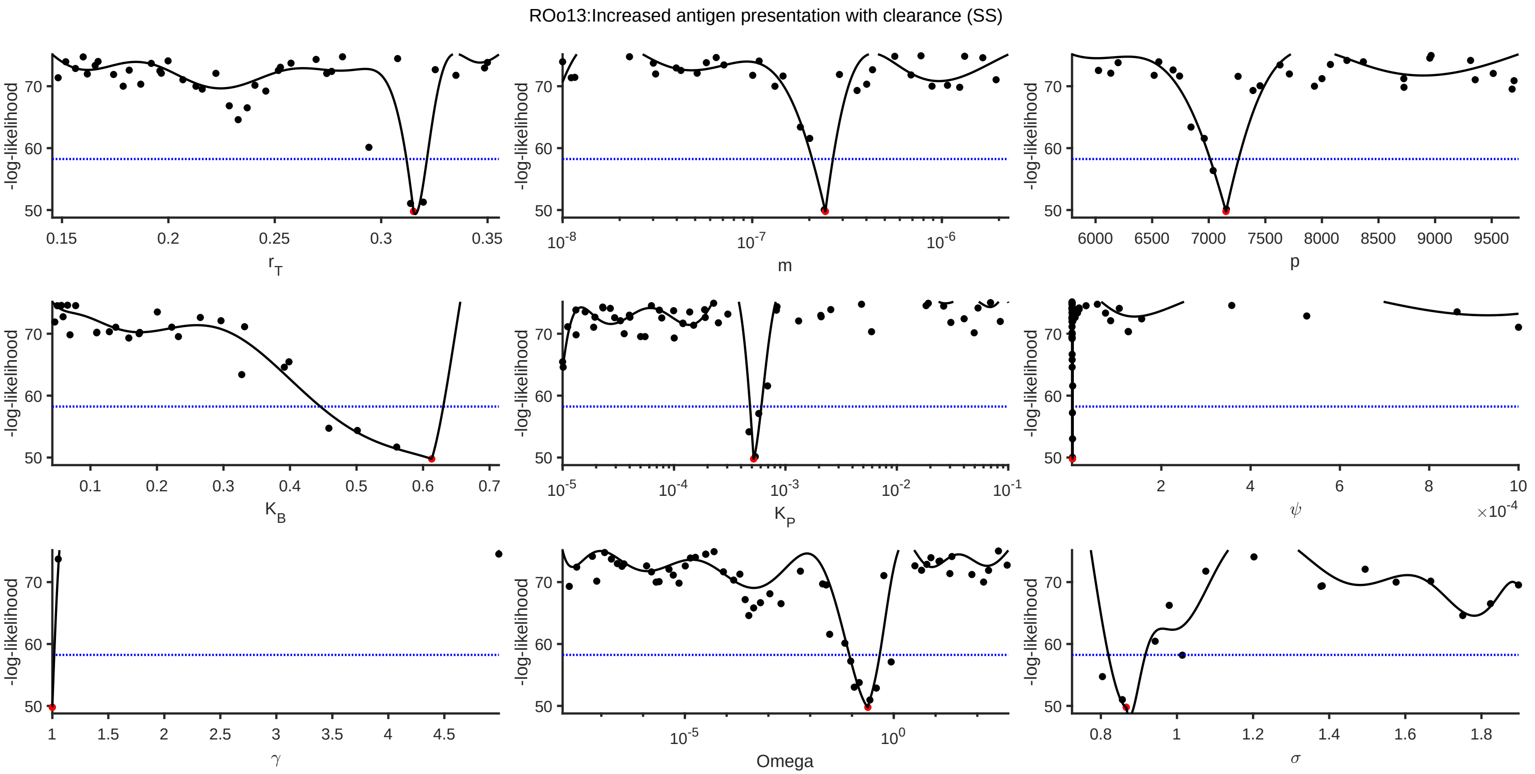
ROo13:Increased antigen presentation with clearance (SS)
-log-likelihood
r_T
m
p
K_B
K_P
ψ
×10^-4
γ
Omega
σ

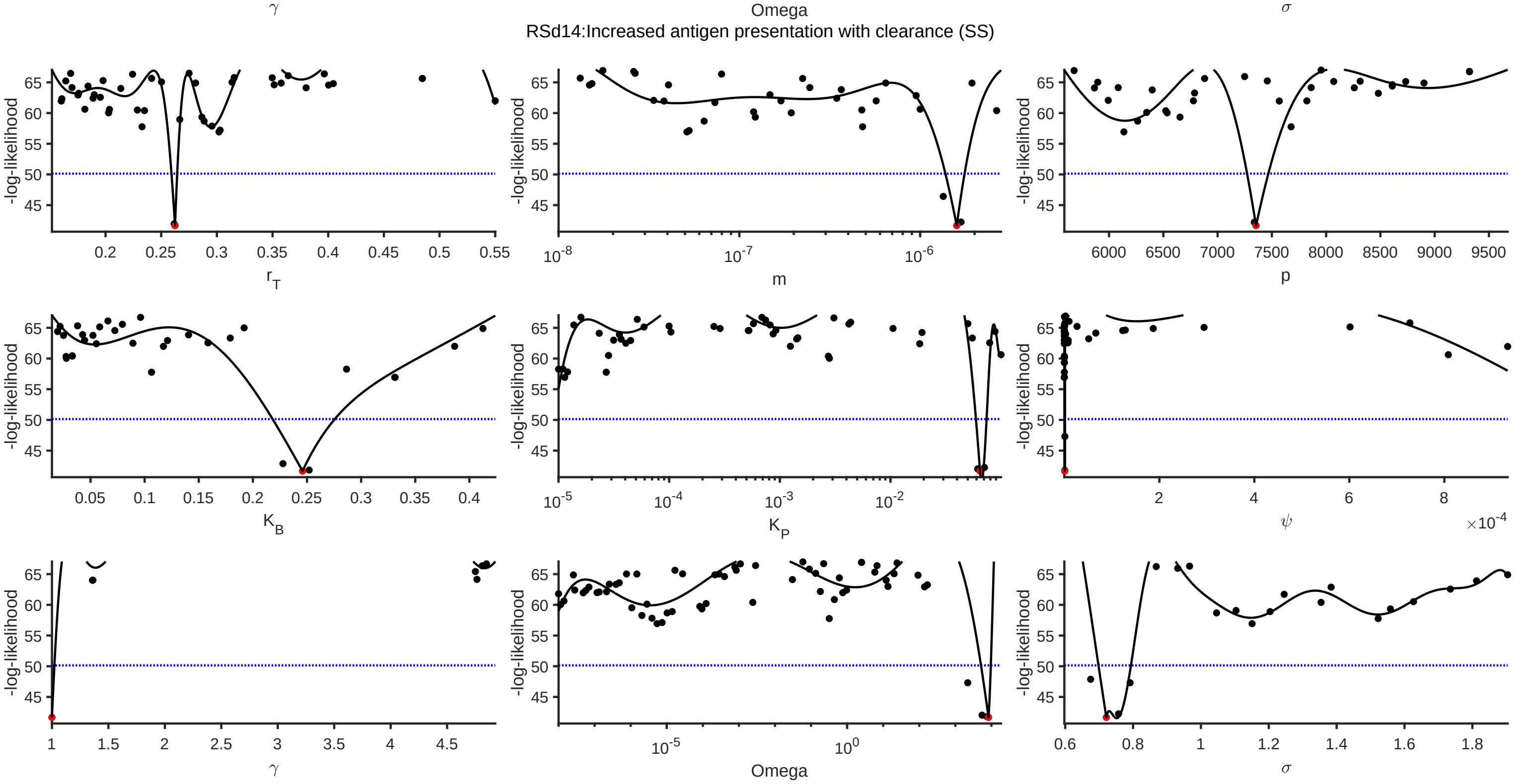
RSd14:Increased antigen presentation with clearance (SS)
-log-likelihood
r_T
m
p
K_B
K_P
ψ
×10^-4
γ
Omega
σ

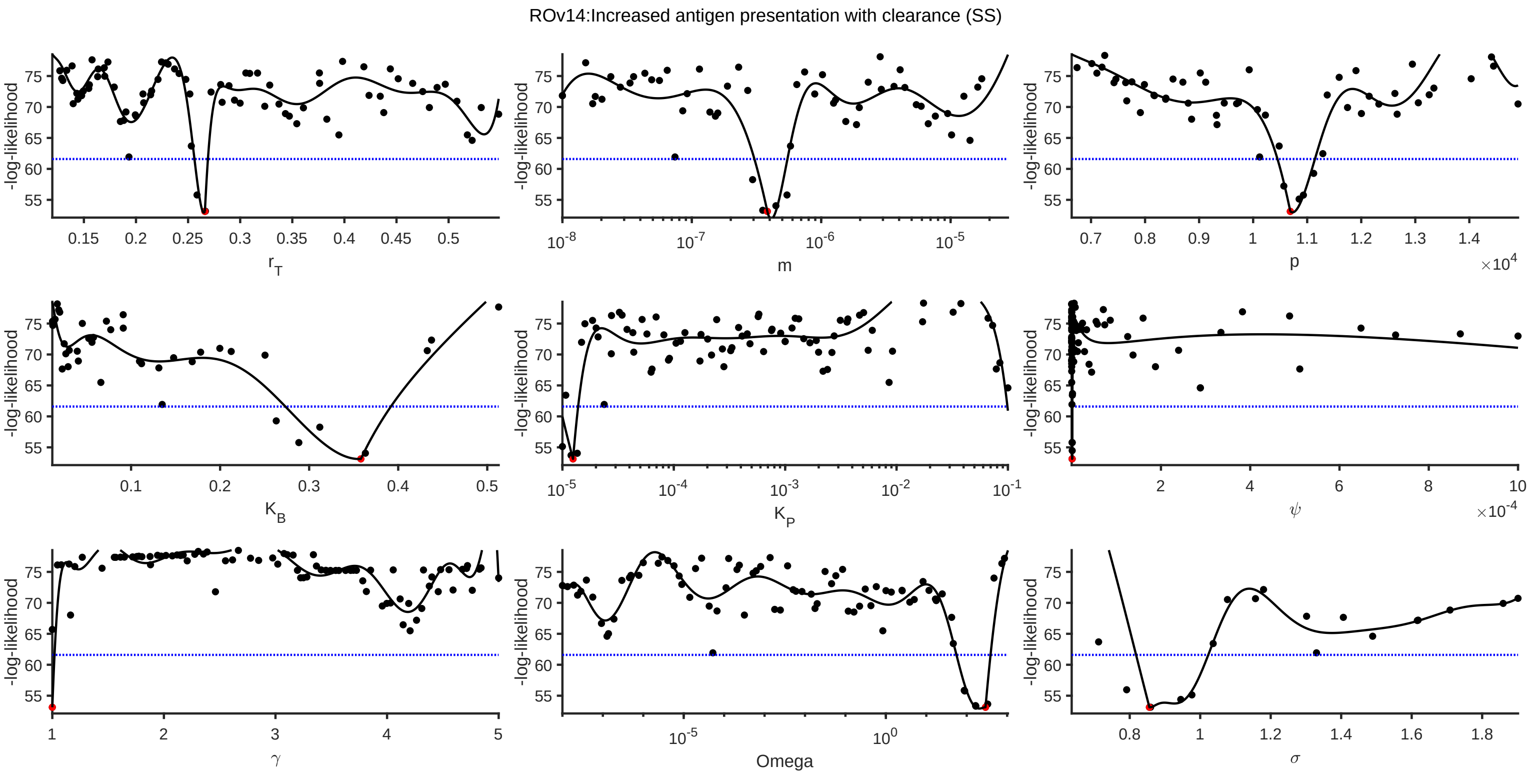
ROv14:Increased antigen presentation with clearance (SS)
-log-likelihood
r_T
m
p
$\times 10^4$
K_B
K_P
ψ
$\times 10^{-4}$
γ
Omega
σ

11.3 Antigen presenting cell effector cell source model

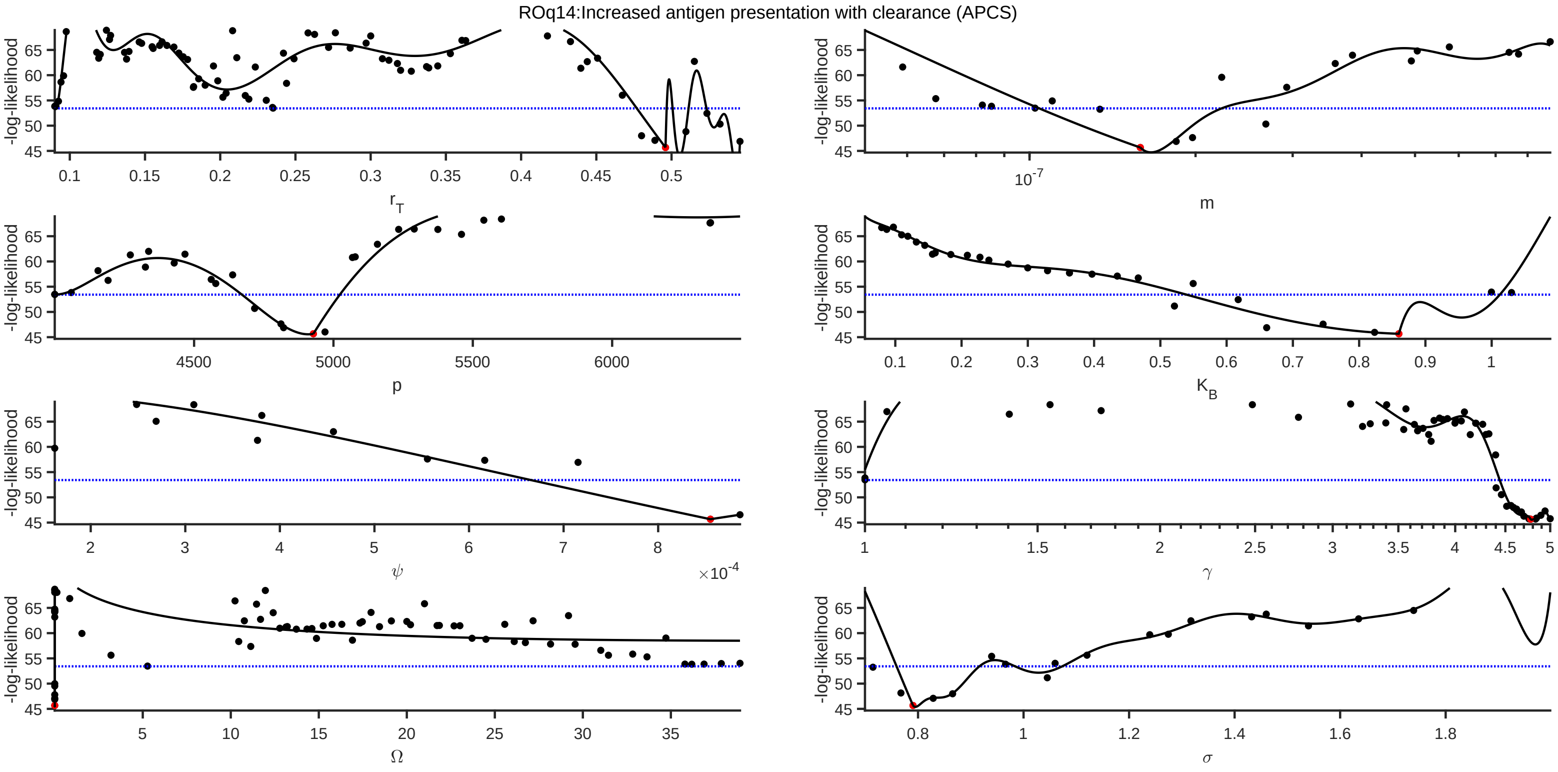

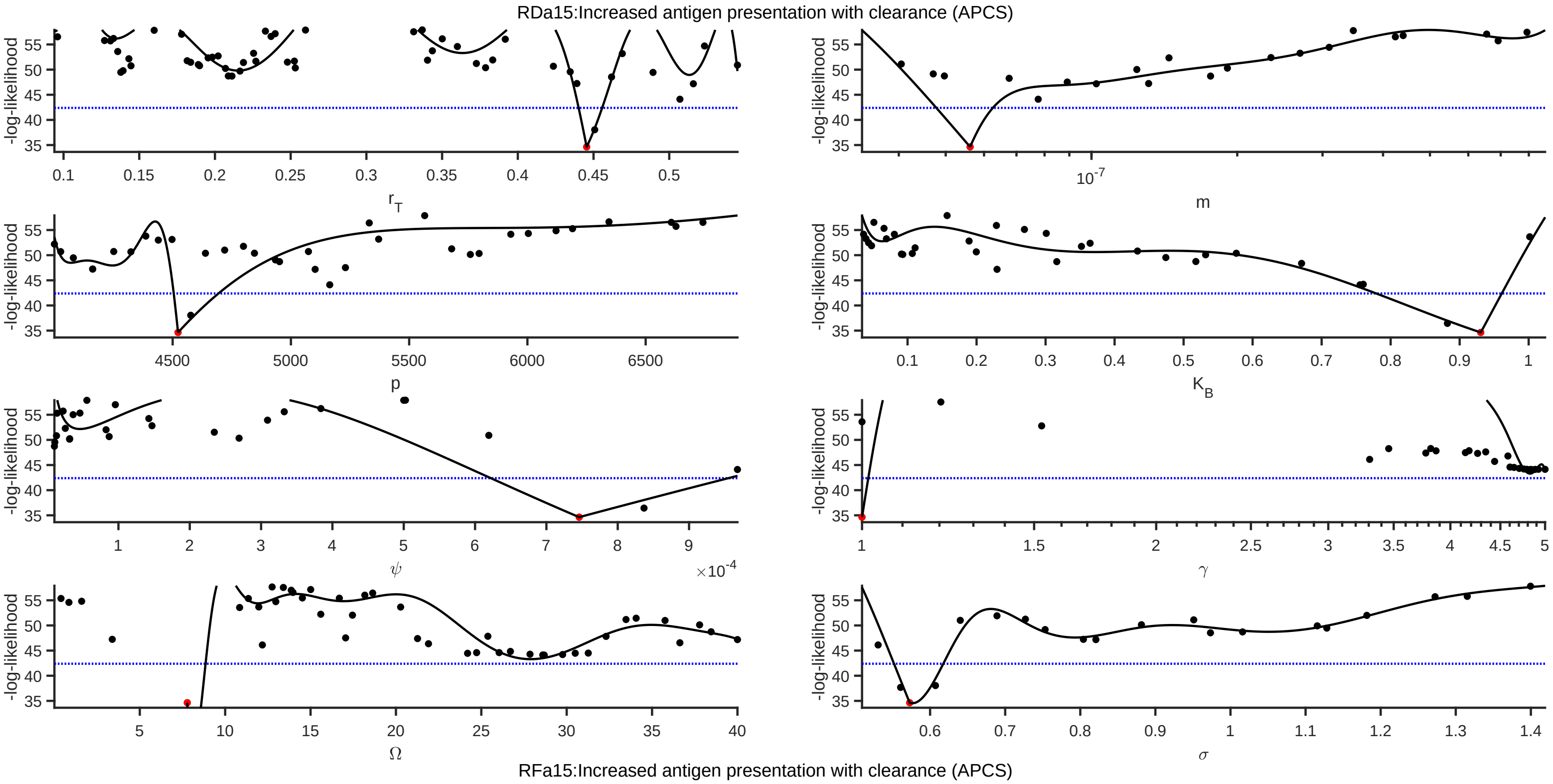
RDa15:Increased antigen presentation with clearance (APCS)
-log-likelihood
r_T
m
p
K_B
ψ
γ
Ω
σ

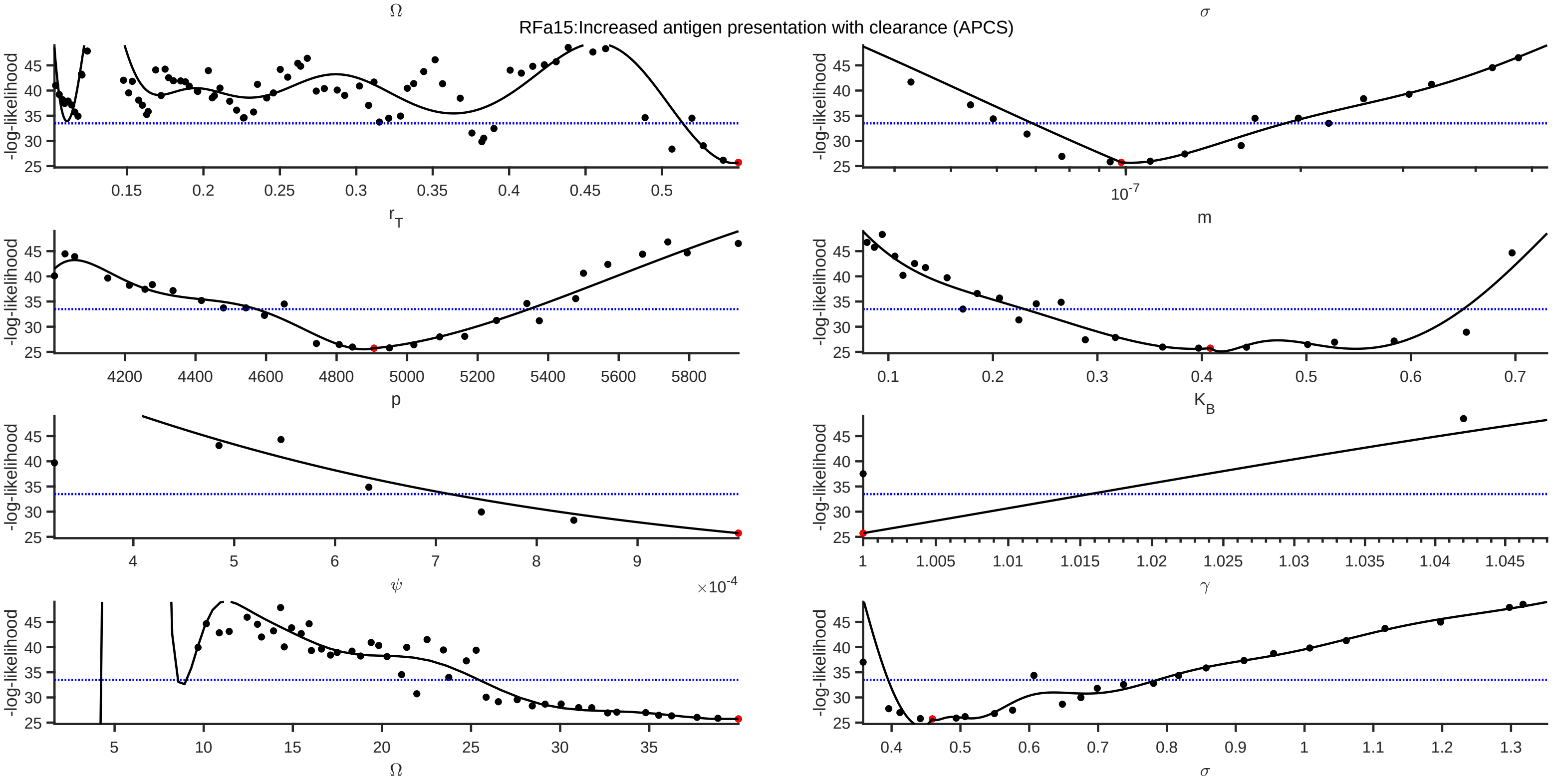
RFa15:Increased antigen presentation with clearance (APCS)
-log-likelihood
r_T
m
p
K_B
ψ
γ
Ω
σ

RLn12:Increased antigen presentation with clearance (APCS)

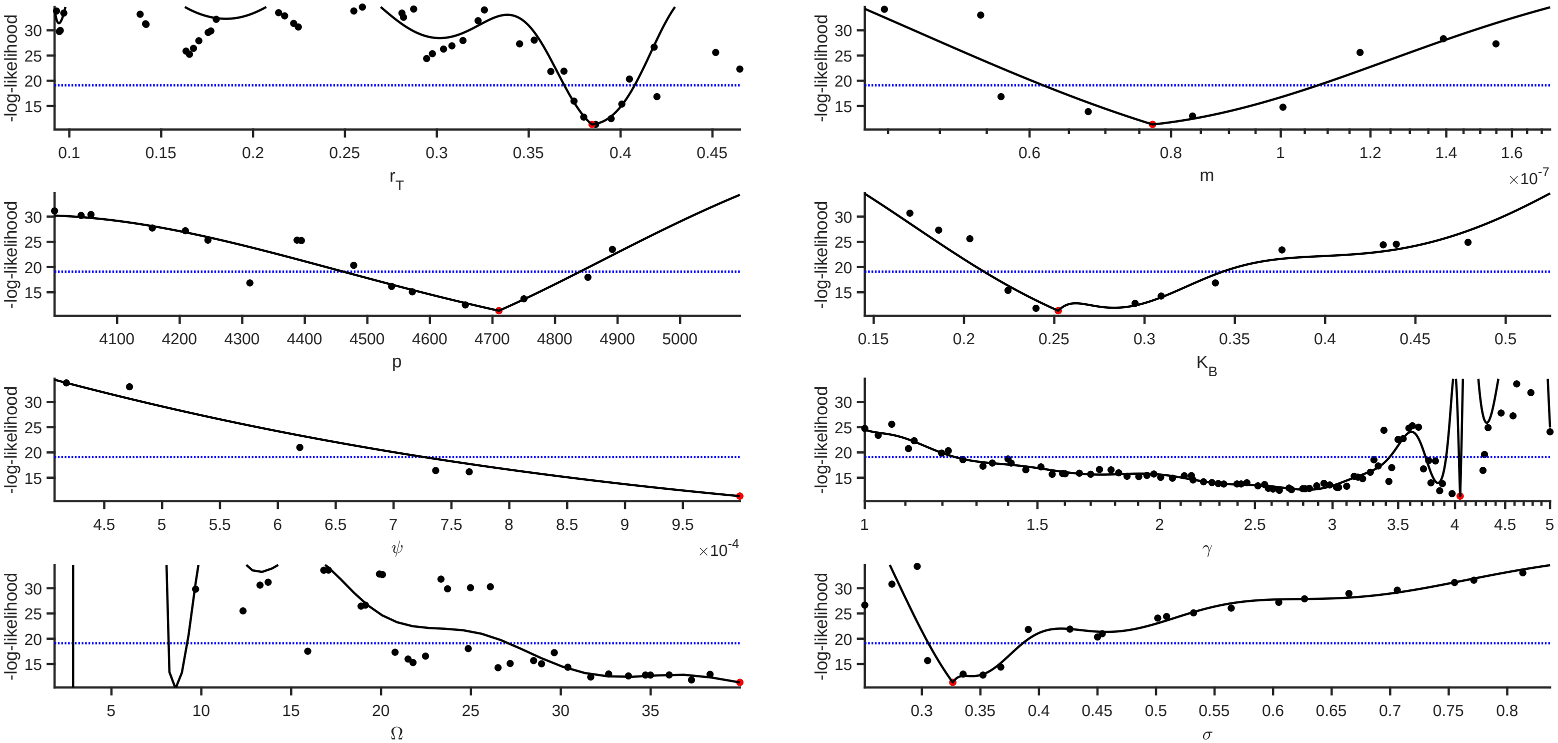

RId14:Increased antigen presentation with clearance (APCS)

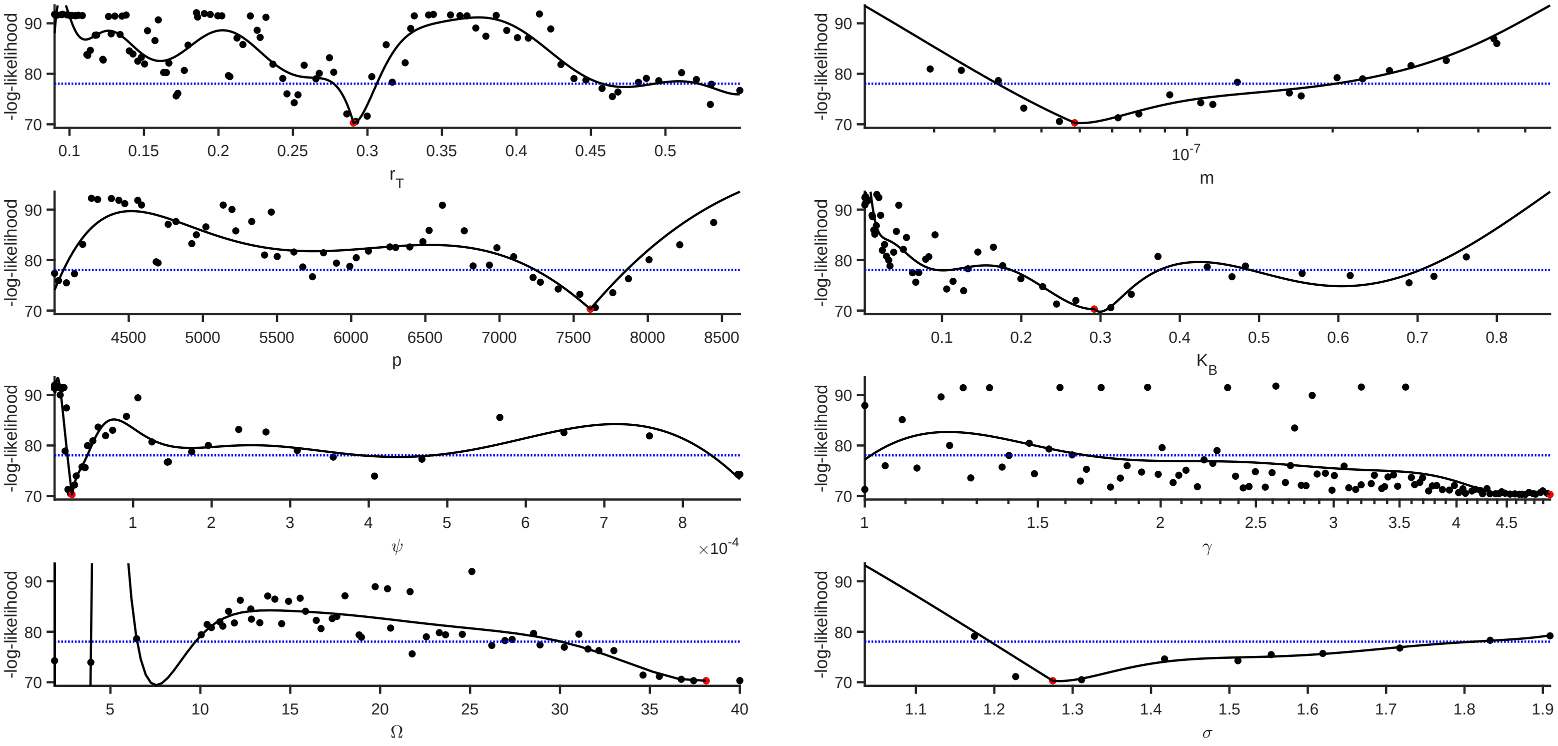

ROo13:Increased antigen presentation with clearance (APCS)

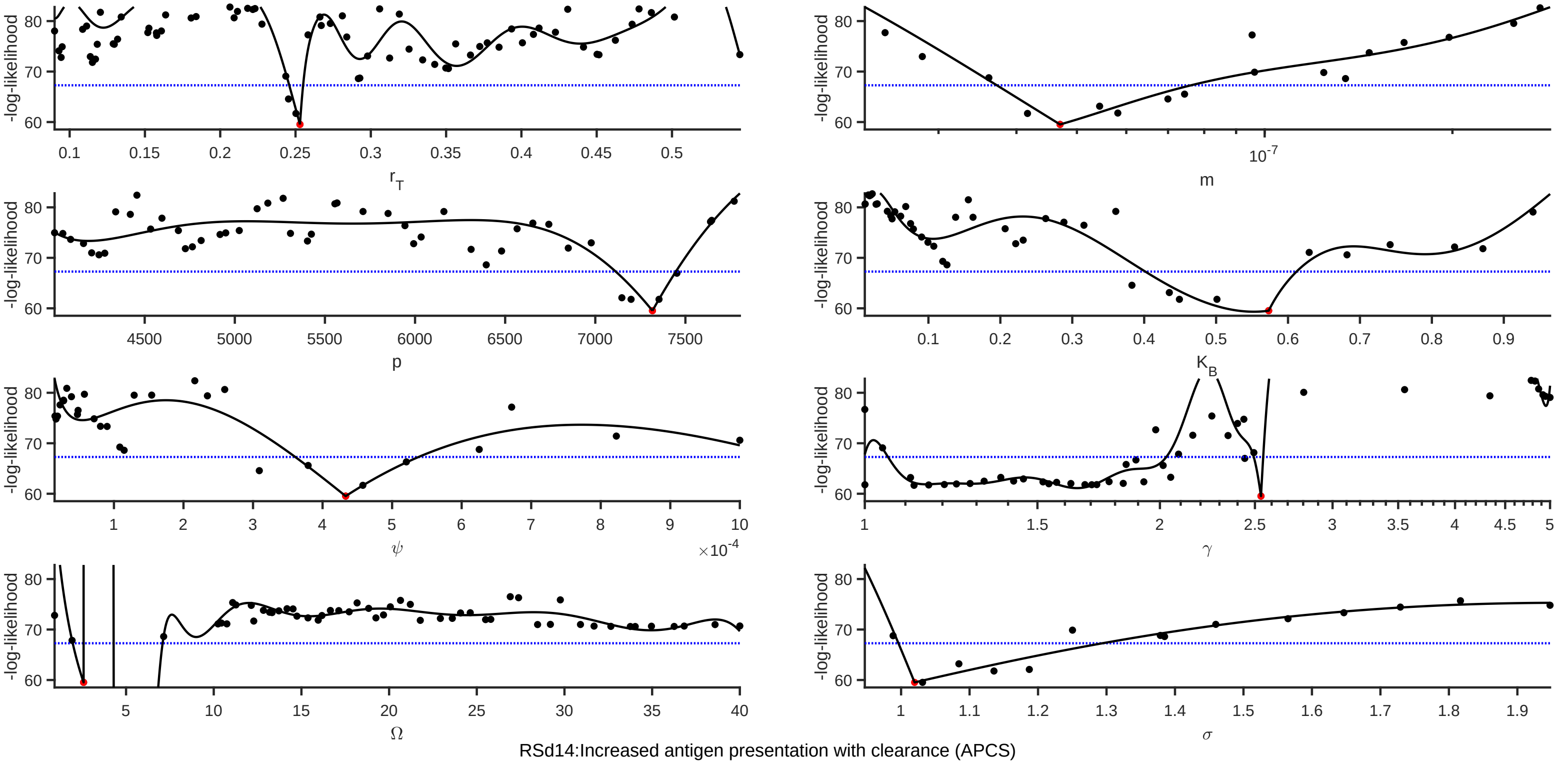

RSd14:Increased antigen presentation with clearance (APCS)

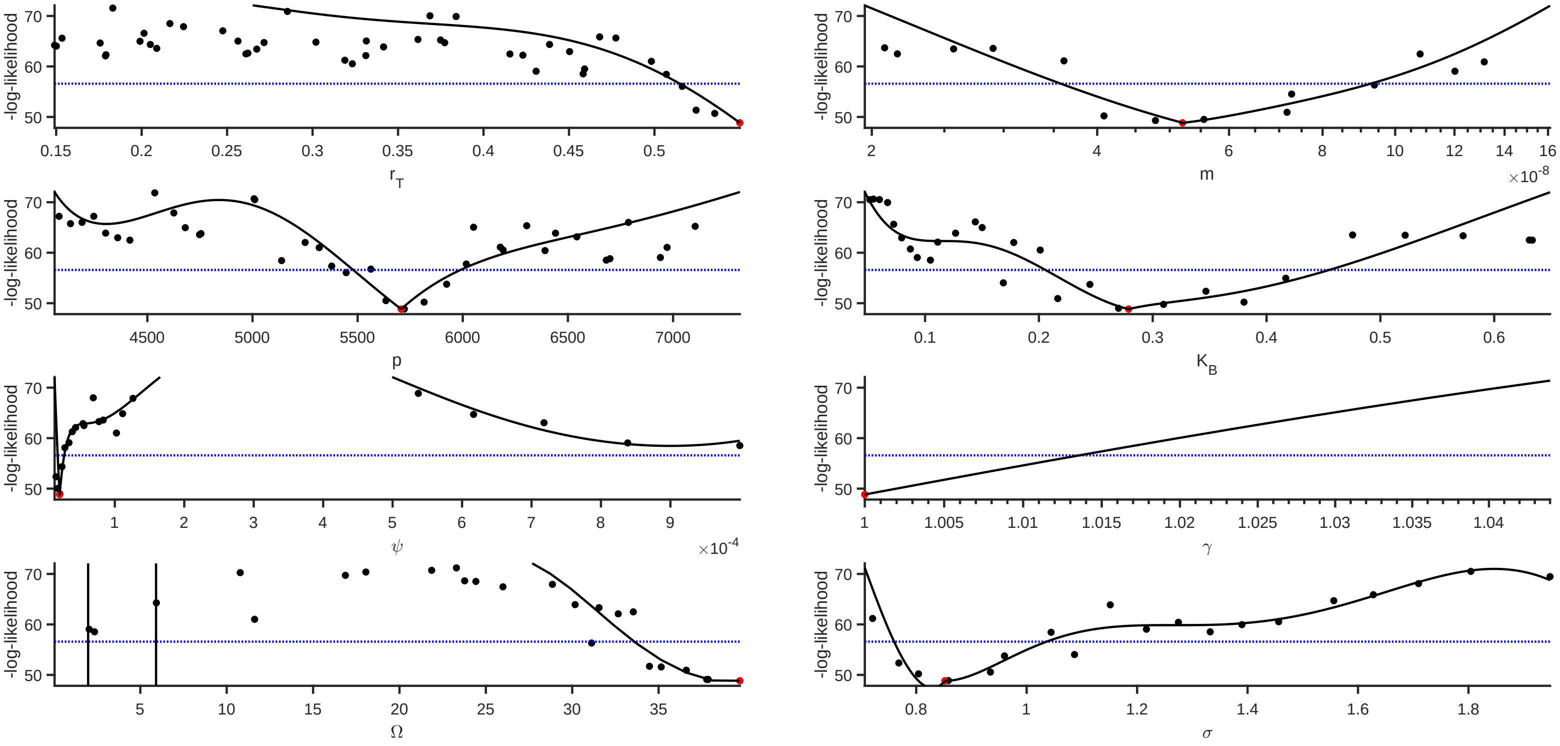

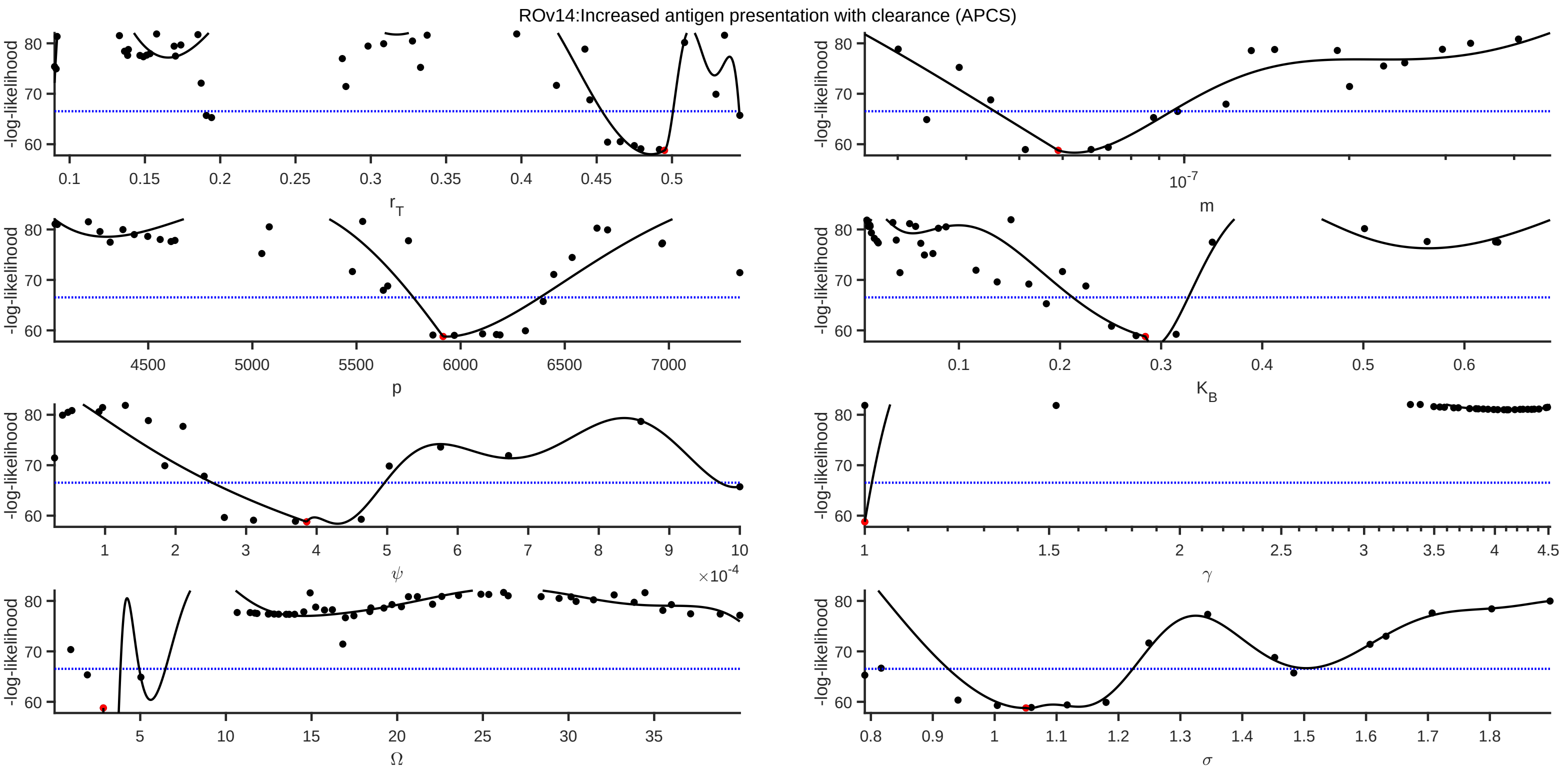
ROv14:Increased antigen presentation with clearance (APCS)
-log-likelihood
r_T
m
10^{-7}
p
K_B
ψ
$\times 10^{-4}$
γ
Ω
σ

12 Sensitivity anlaysis: Increased antigen presentation with increased viral clearance

12.1 Baseline effector cell source model

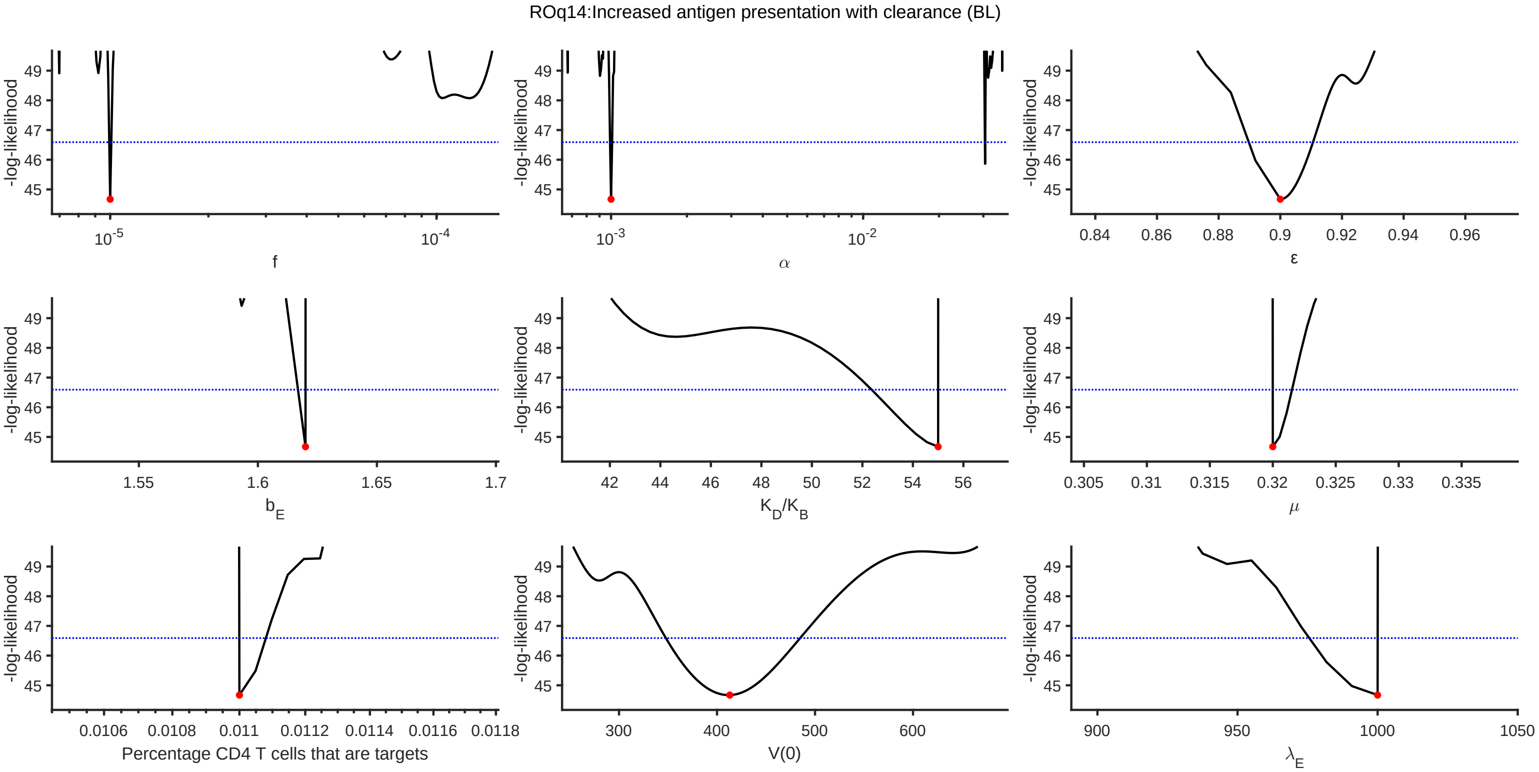

RDa15:Increased antigen presentation with clearance (BL)

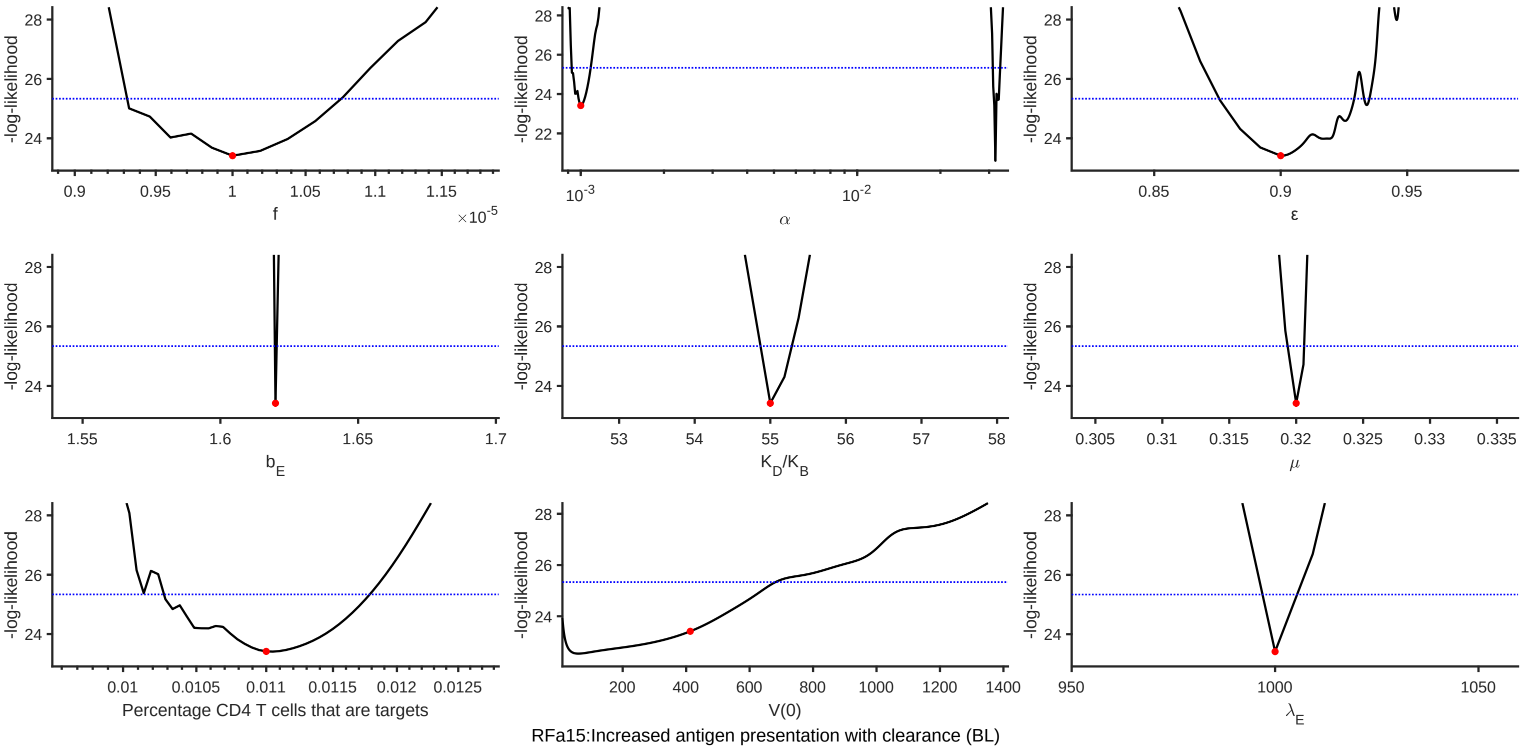

RFa15:Increased antigen presentation with clearance (BL)

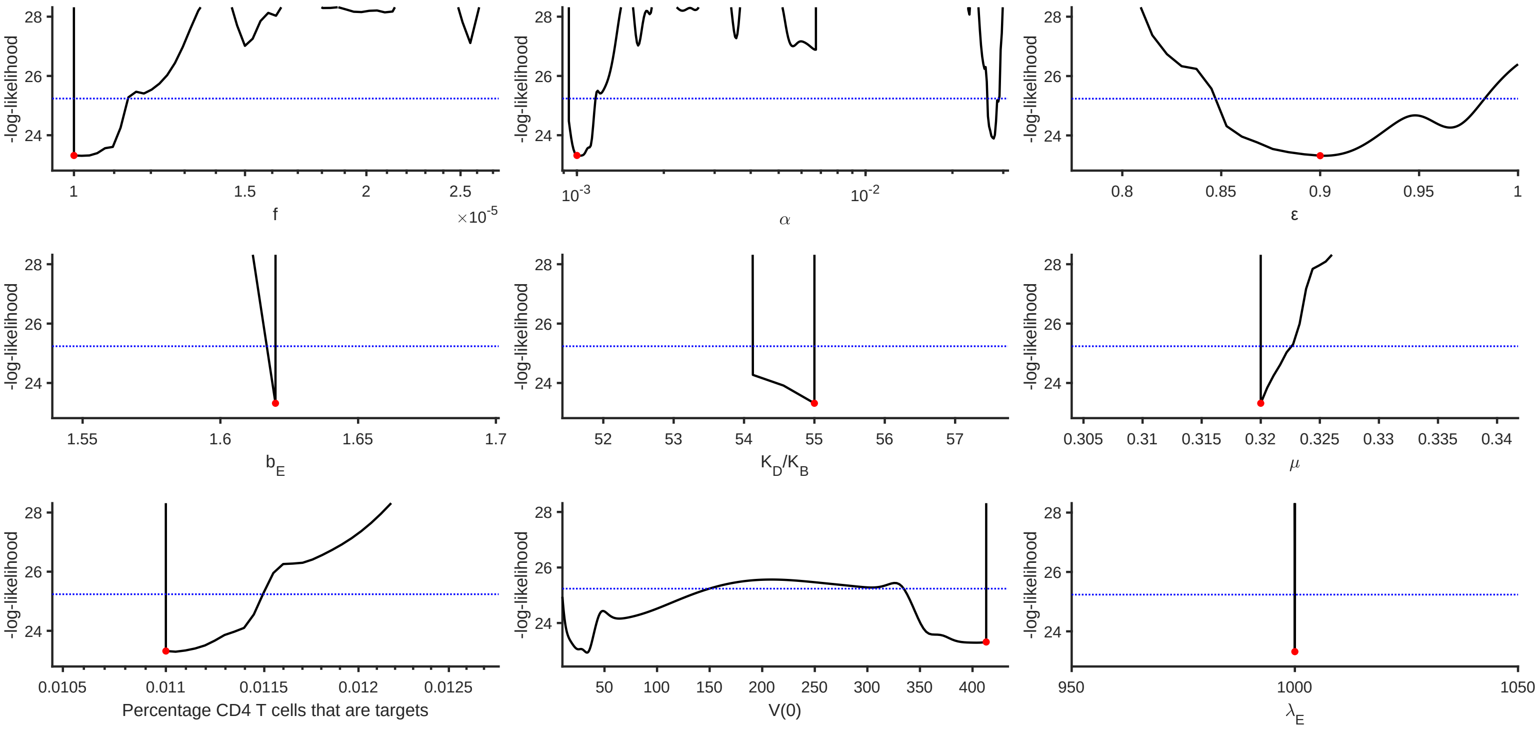

RLn12:Increased antigen presentation with clearance (BL)

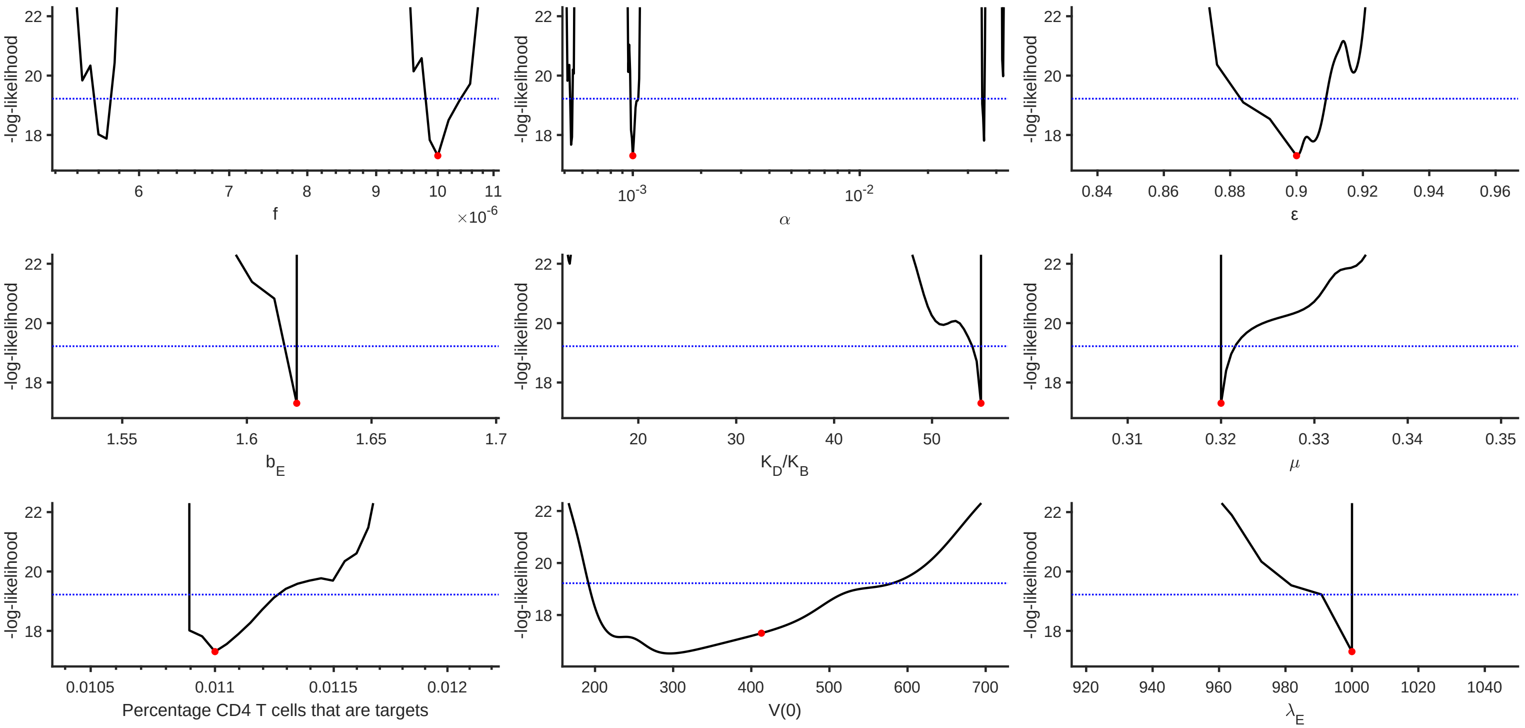

RId14:Increased antigen presentation with clearance (BL)

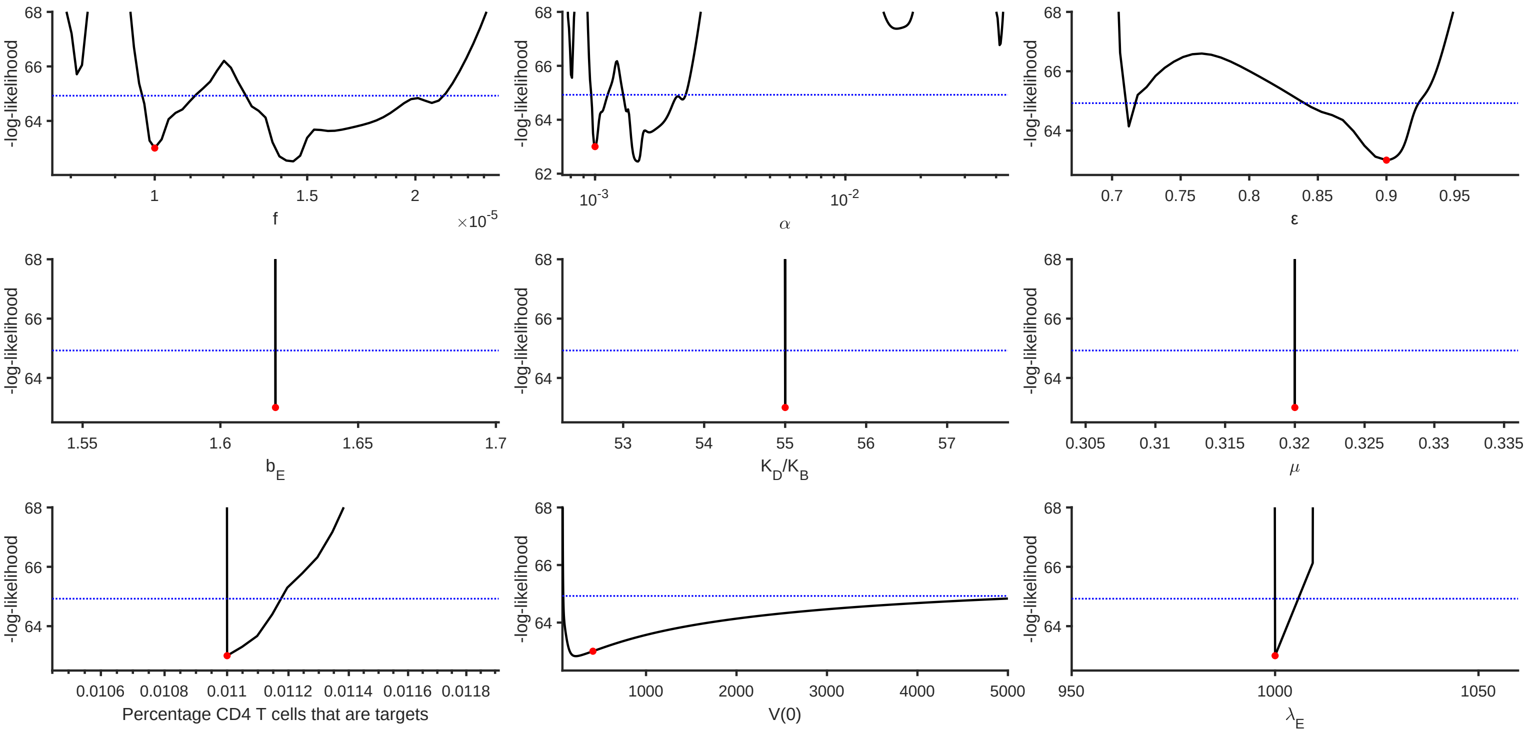

ROo13:Increased antigen presentation with clearance (BL)

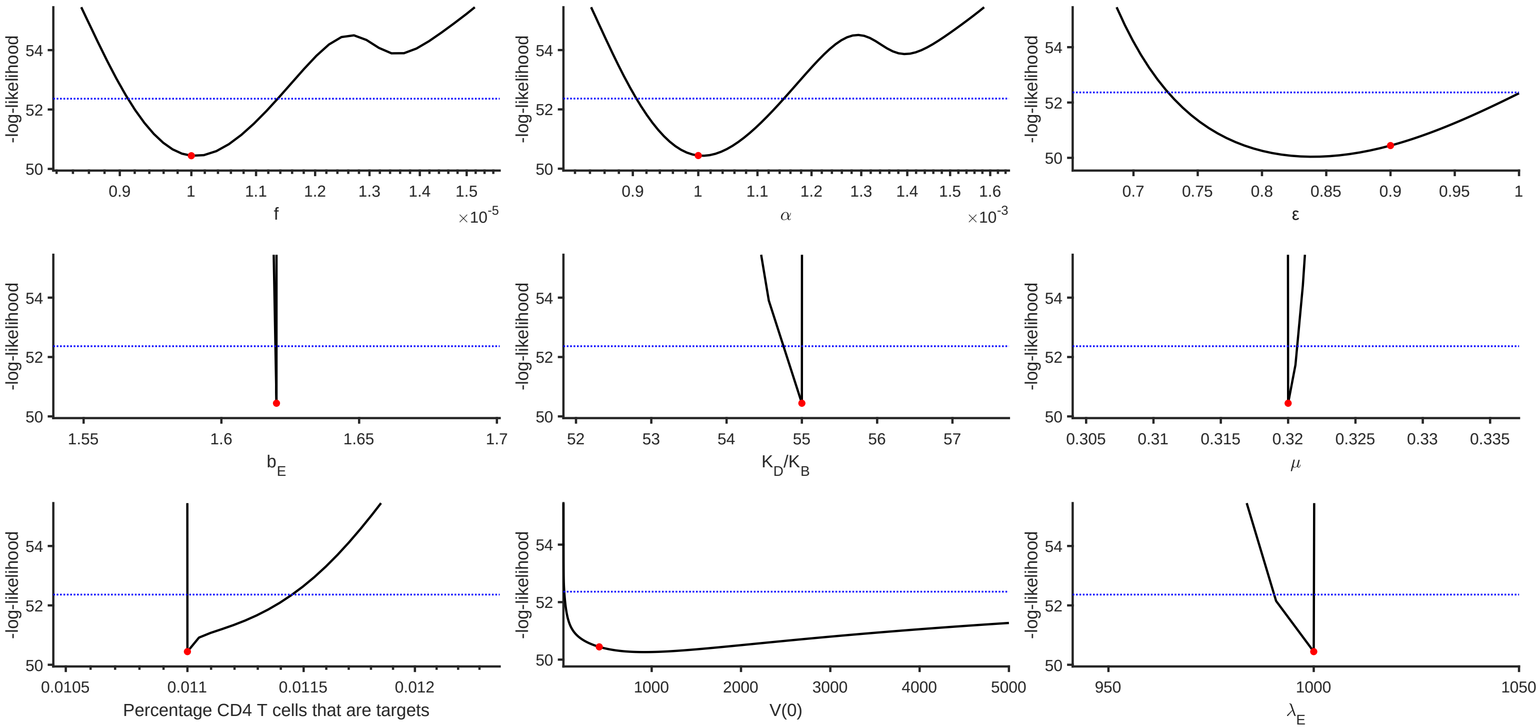

RSd14:Increased antigen presentation with clearance (BL)

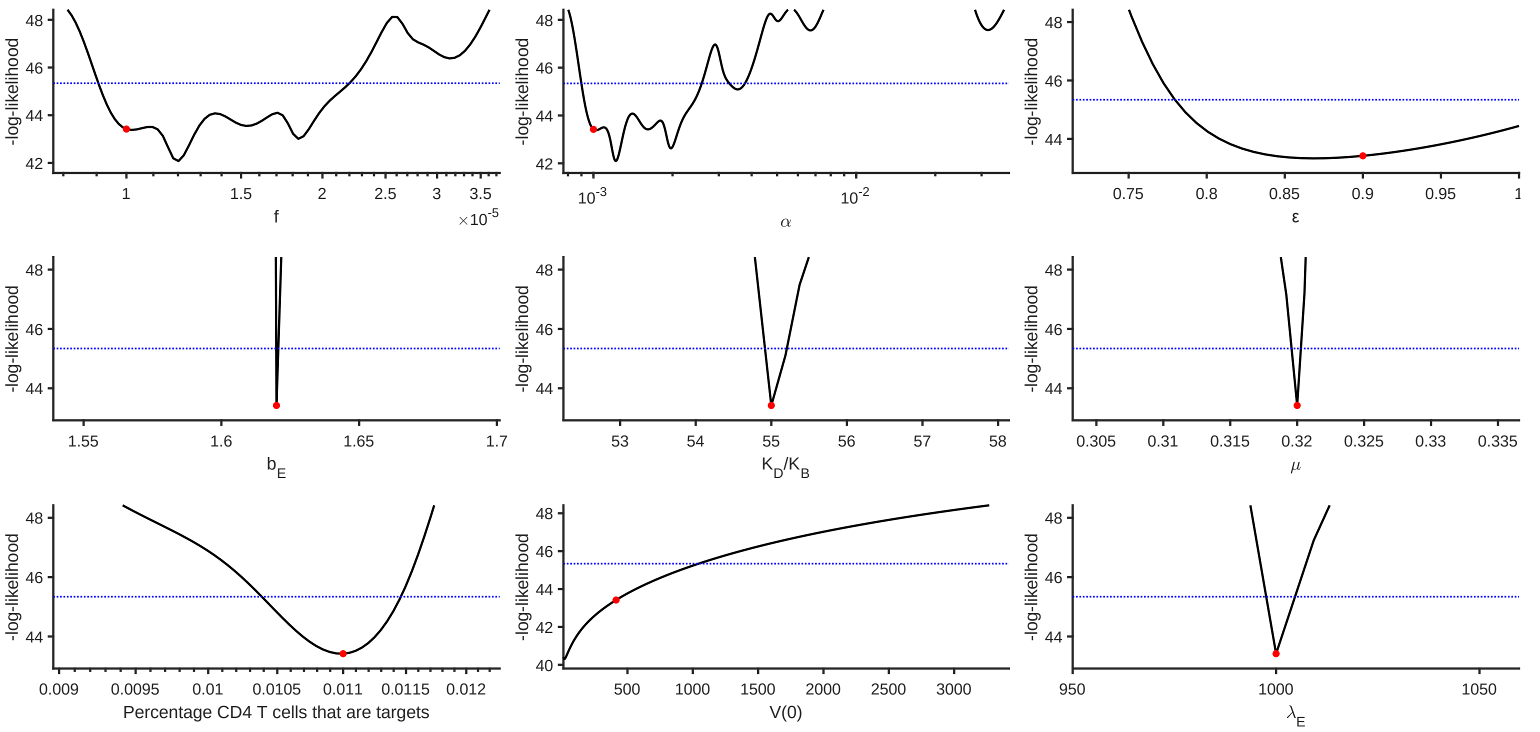

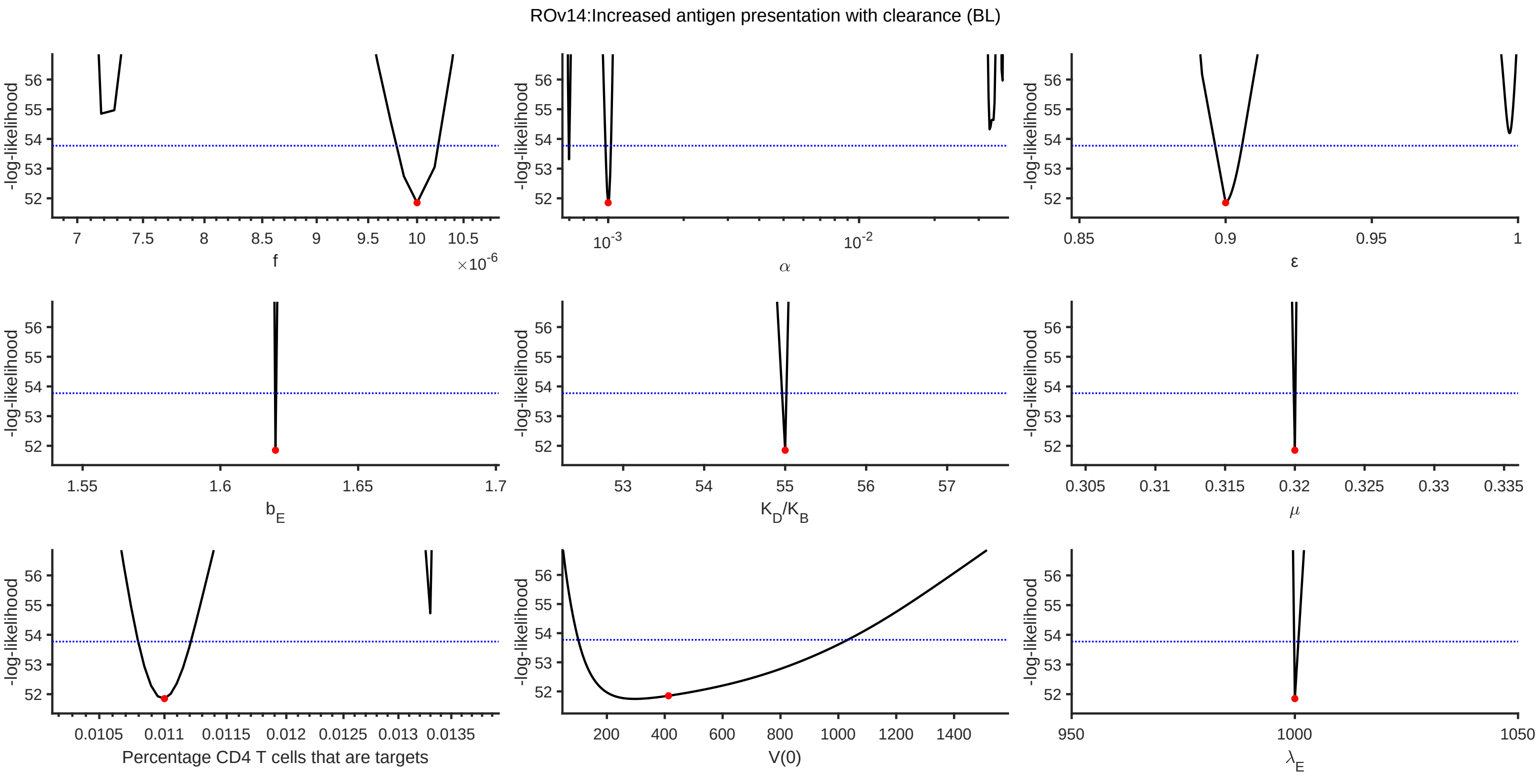
ROv14:Increased antigen presentation with clearance (BL)
-log-likelihood
f
×10^-6
α
ε
b_E
K_D/K_B
μ
Percentage CD4 T cells that are targets
V(0)
λ_E

12.2 Saturated effector cell source model

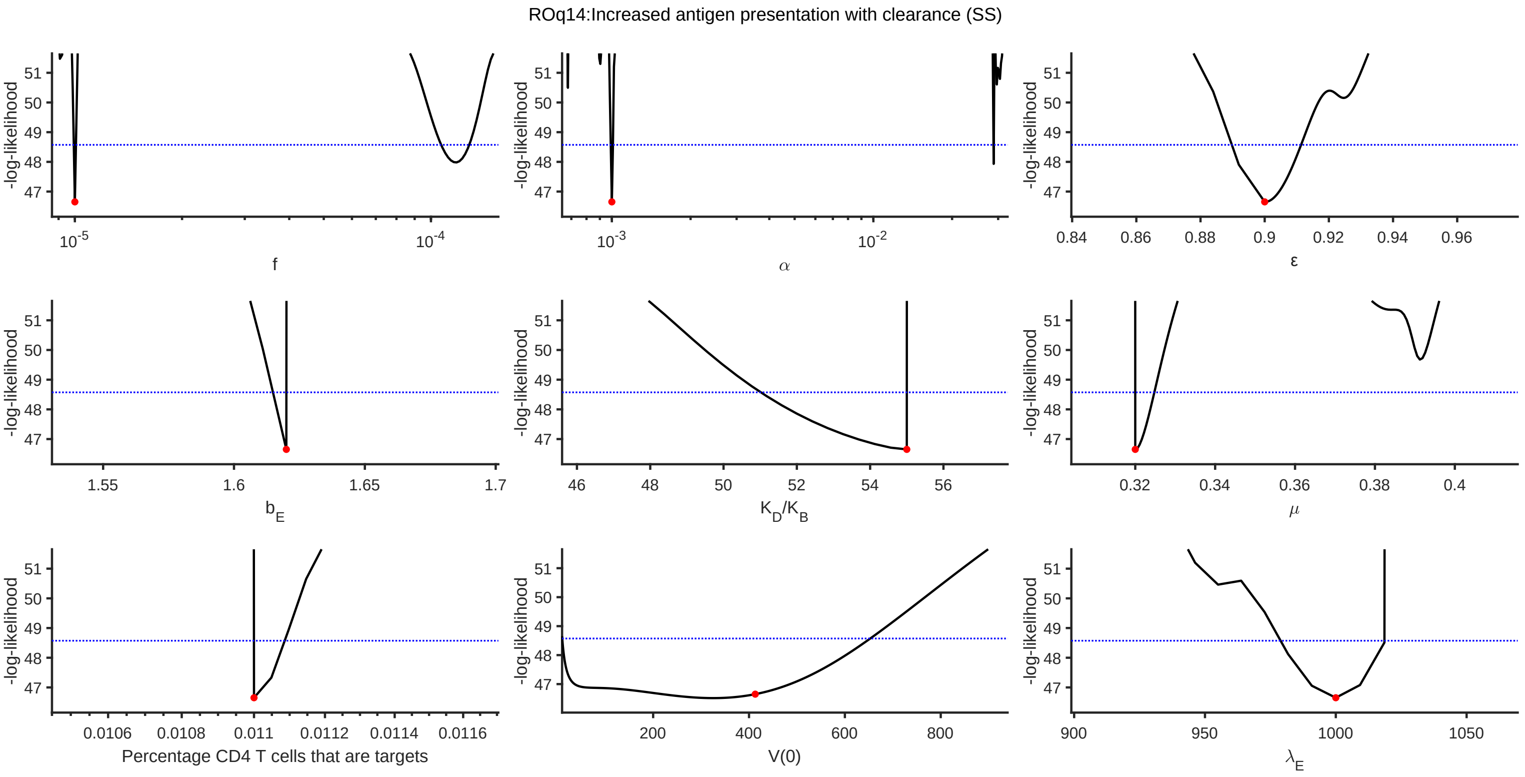

RDa15:Increased antigen presentation with clearance (SS)

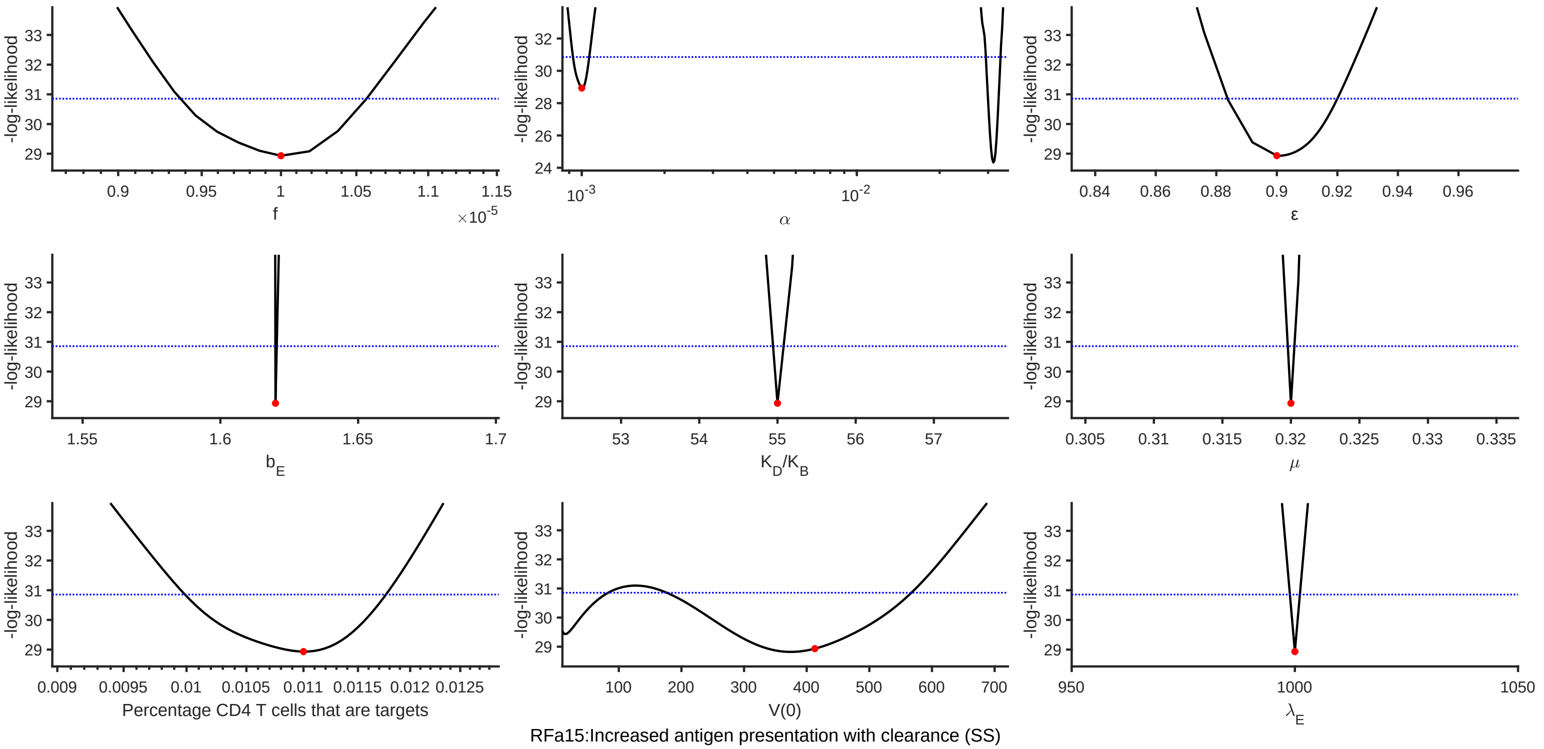

RFa15:Increased antigen presentation with clearance (SS)

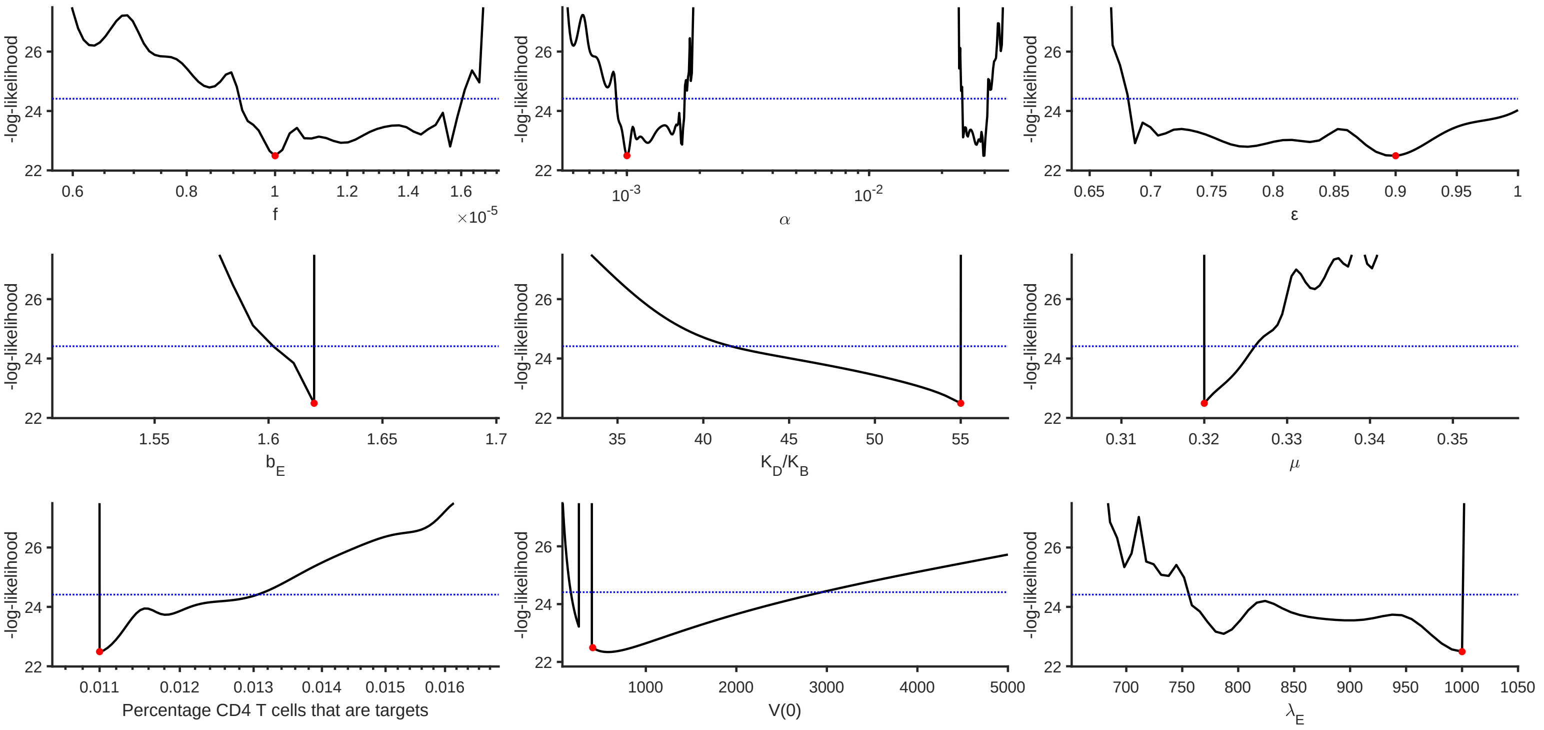

RLn12:Increased antigen presentation with clearance (SS)

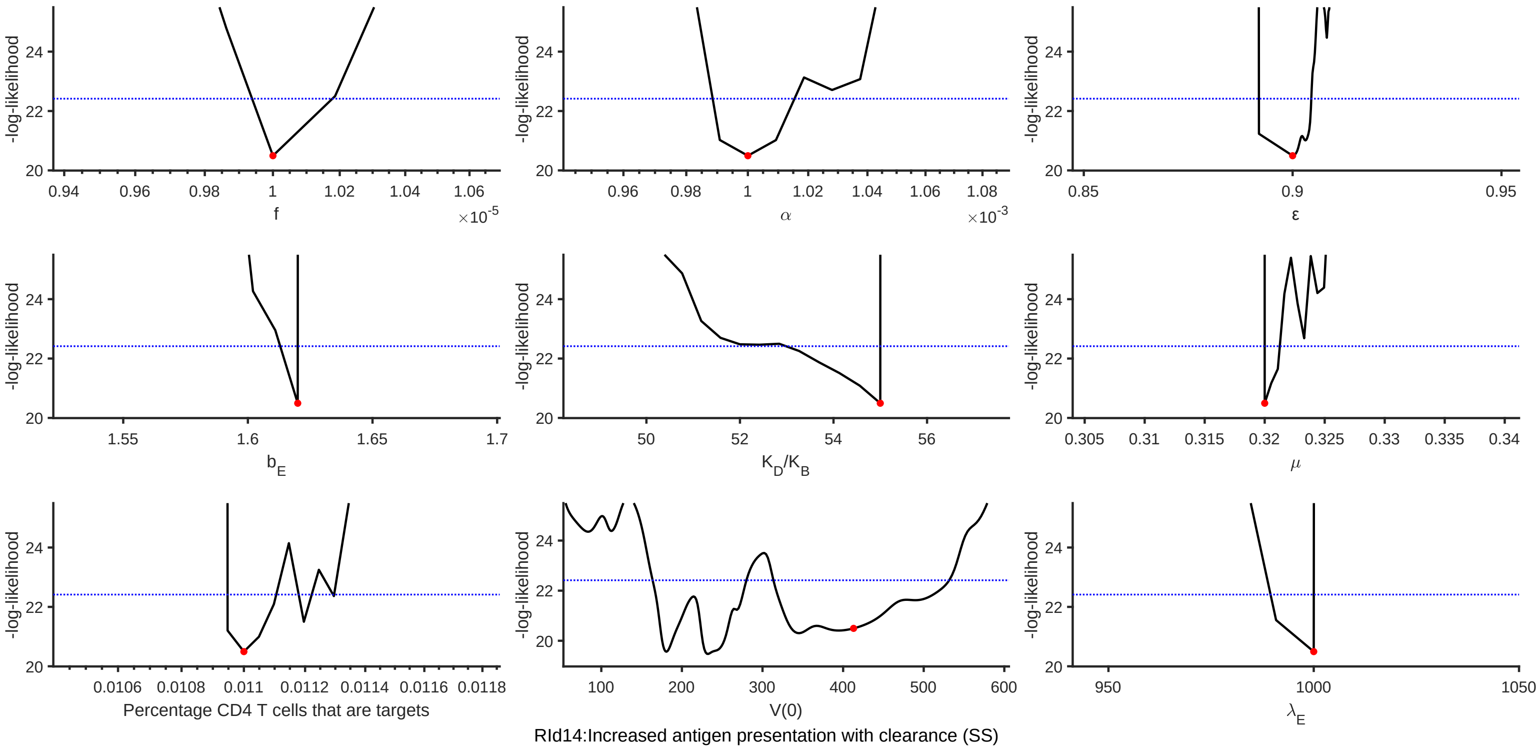

RId14:Increased antigen presentation with clearance (SS)

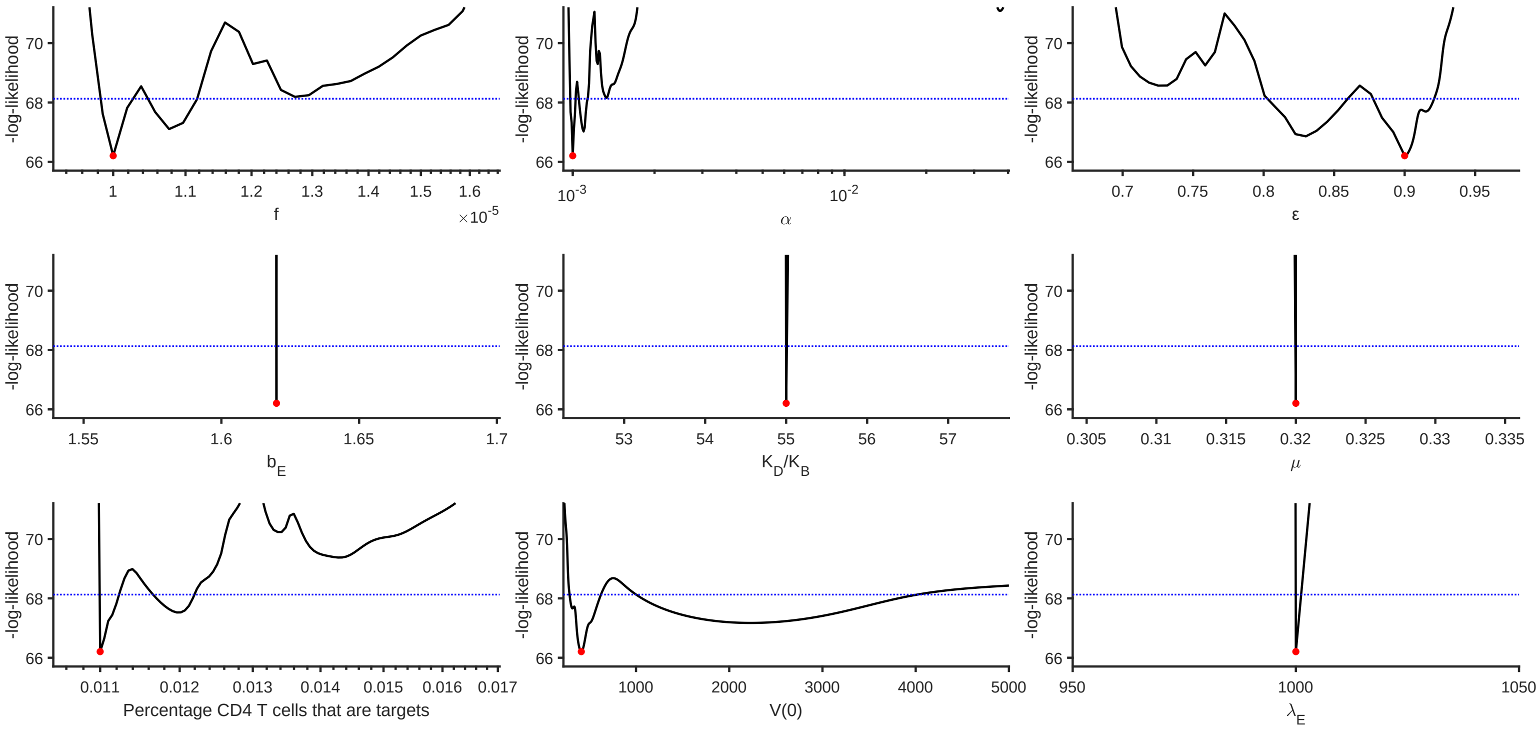

ROo13:Increased antigen presentation with clearance (SS)

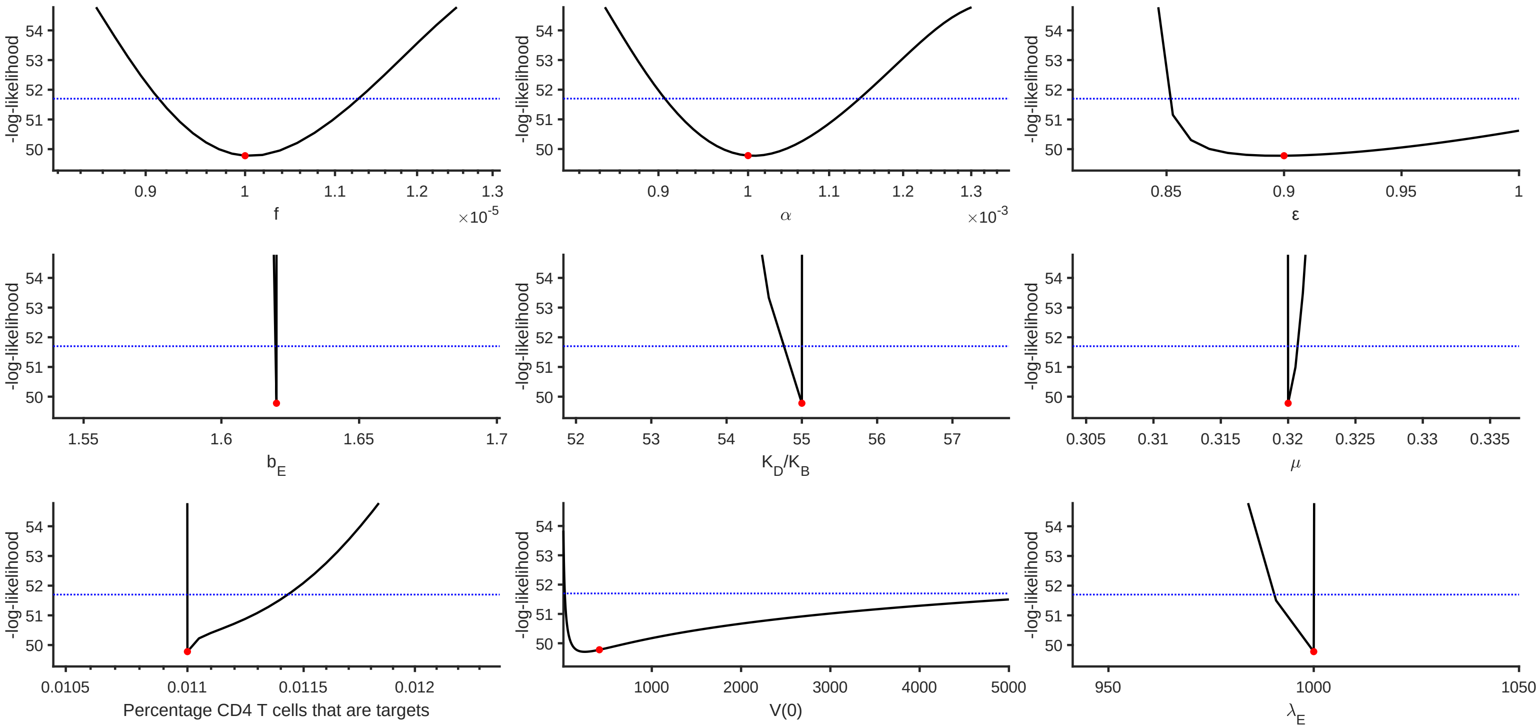

RSd14:Increased antigen presentation with clearance (SS)

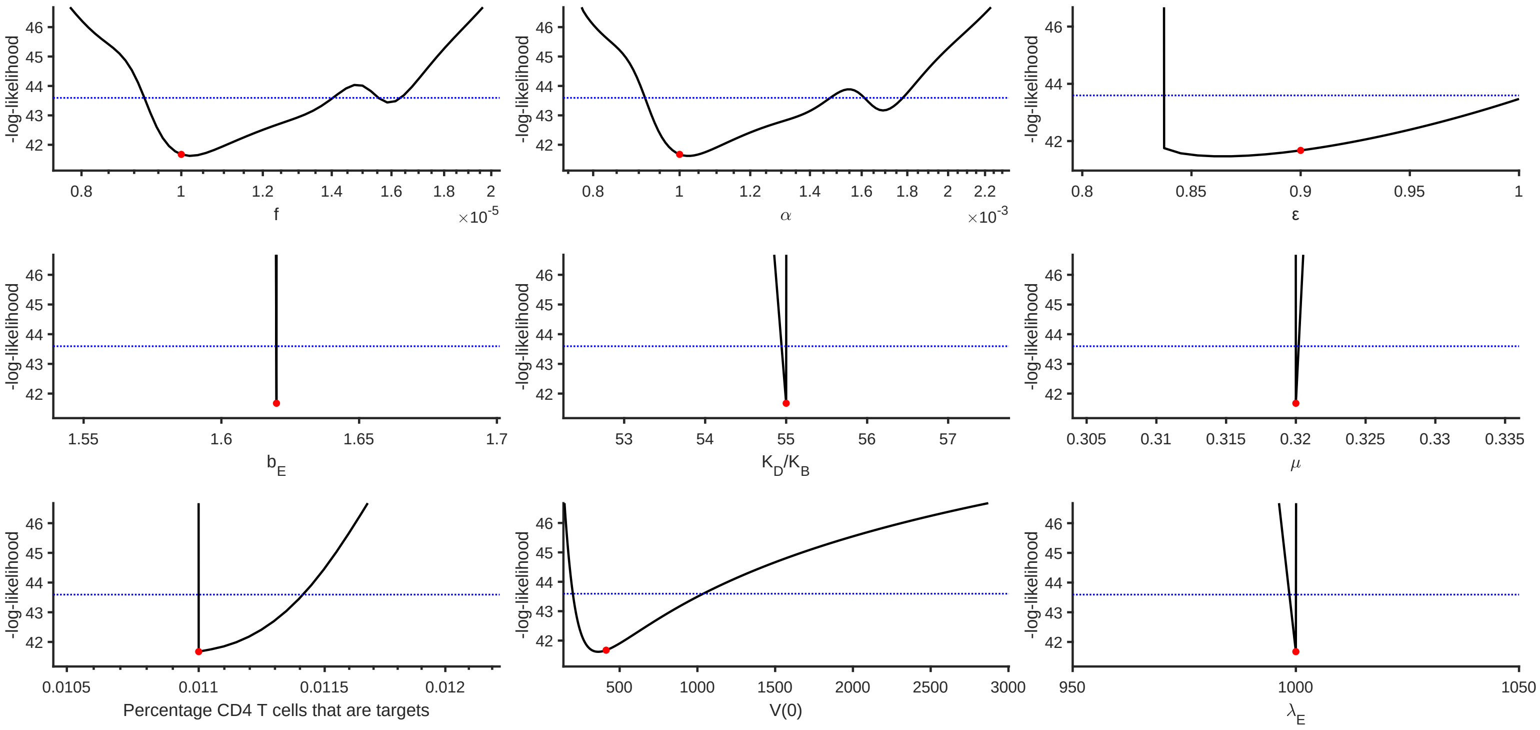

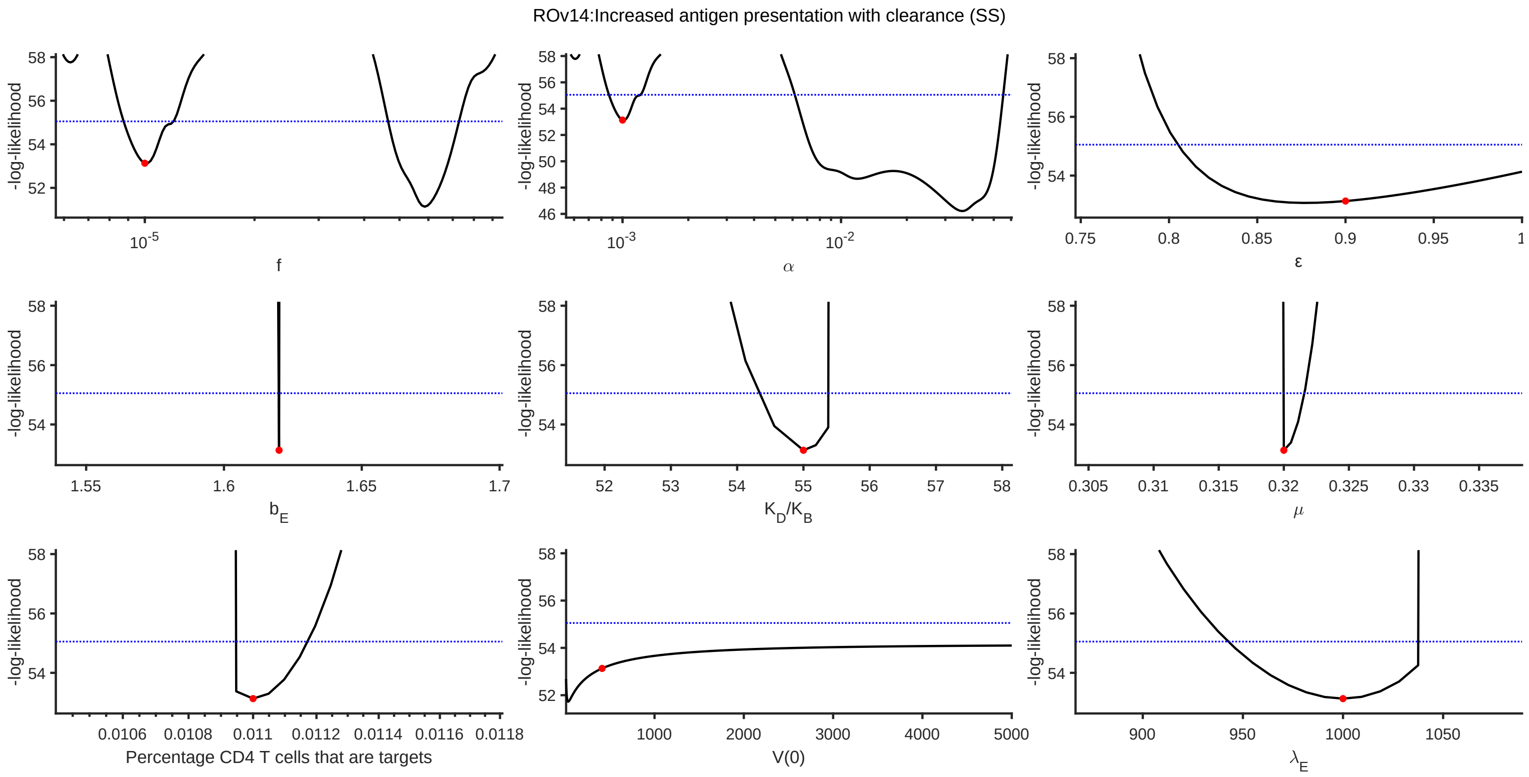

ROv14:Increased antigen presentation with clearance (SS)
-log-likelihood
f
α
ε
b_E
K_D/K_B
μ
Percentage CD4 T cells that are targets
V(0)
λ_E

12.3 Antigen presenting cell effector cell source model

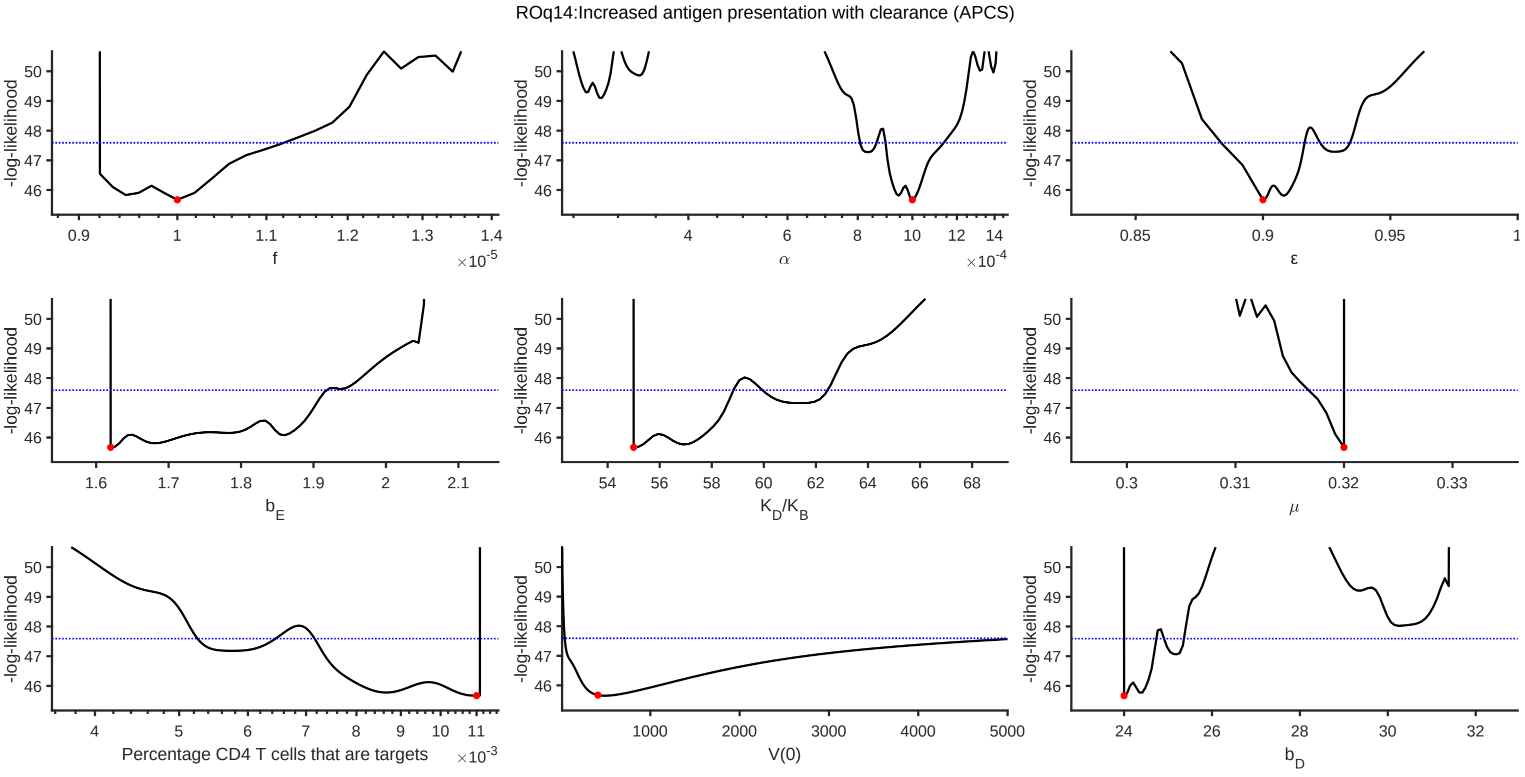

RDa15:Increased antigen presentation with clearance (APCS)

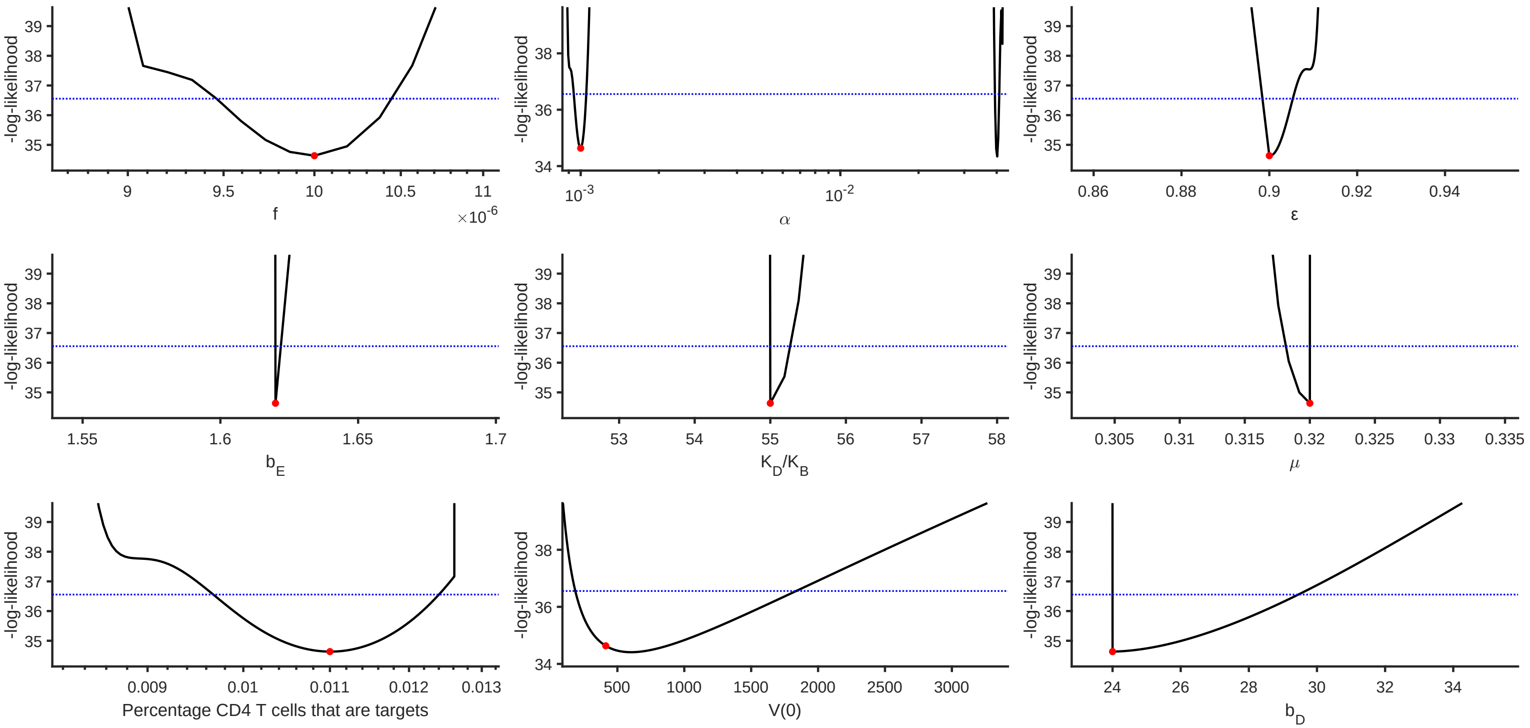

RFa15:Increased antigen presentation with clearance (APCS)

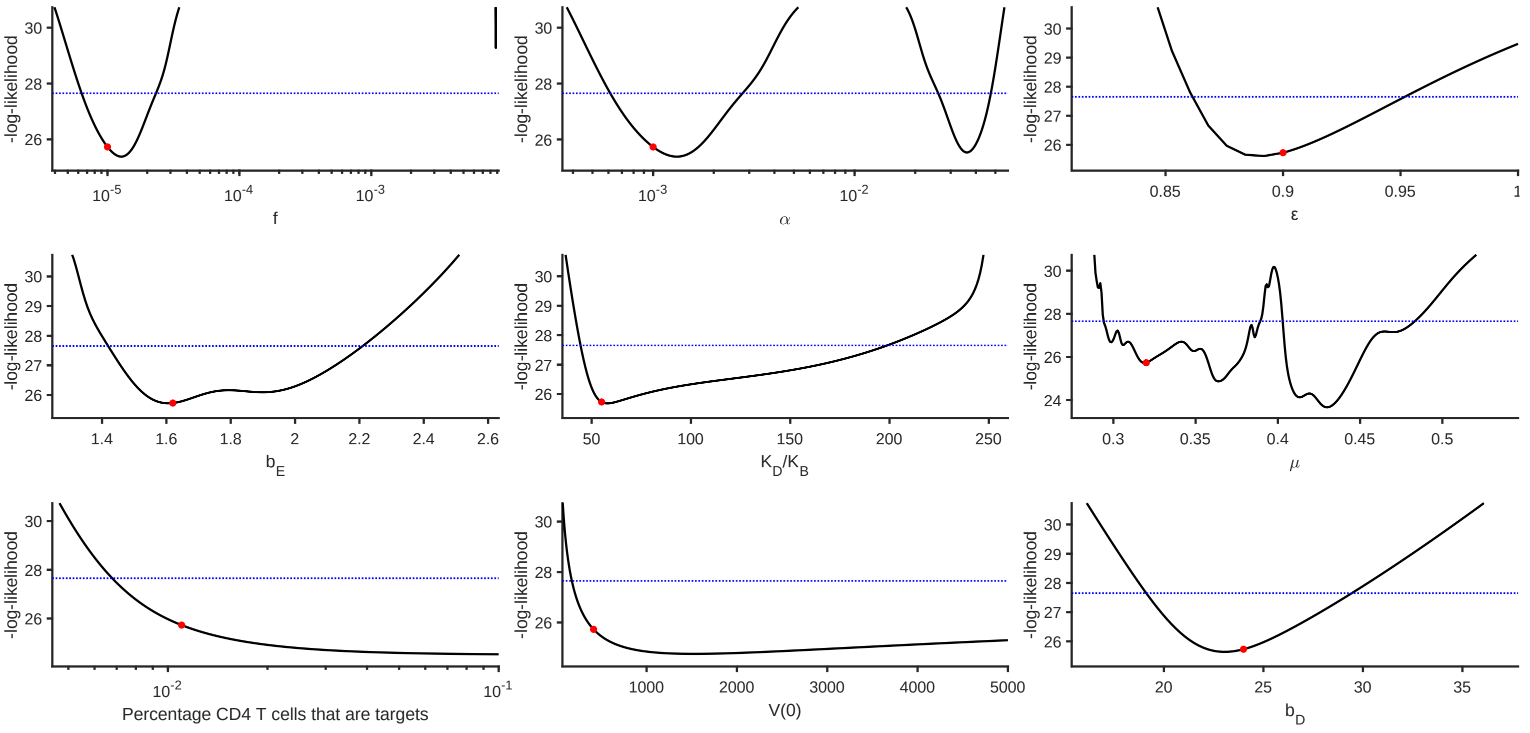

RLn12:Increased antigen presentation with clearance (APCS)

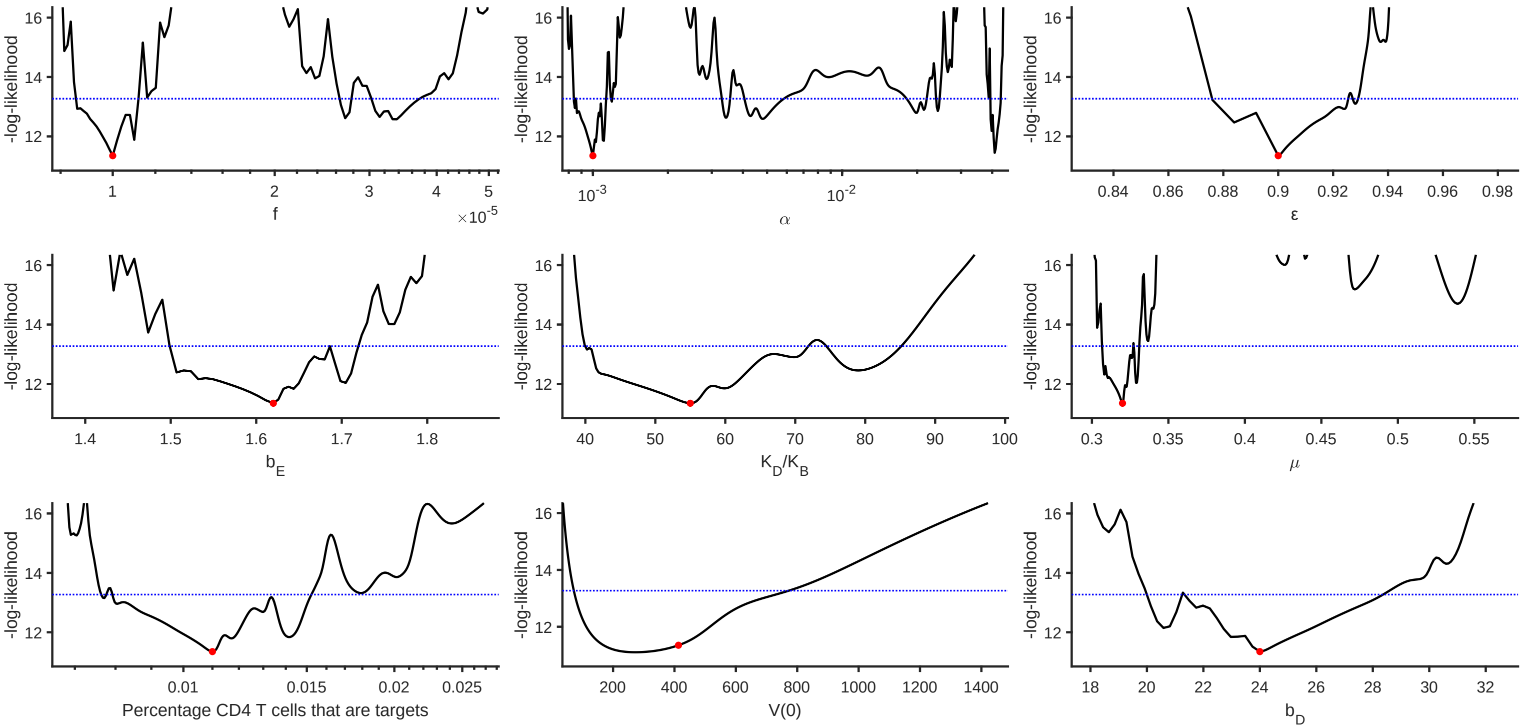

RId14:Increased antigen presentation with clearance (APCS)

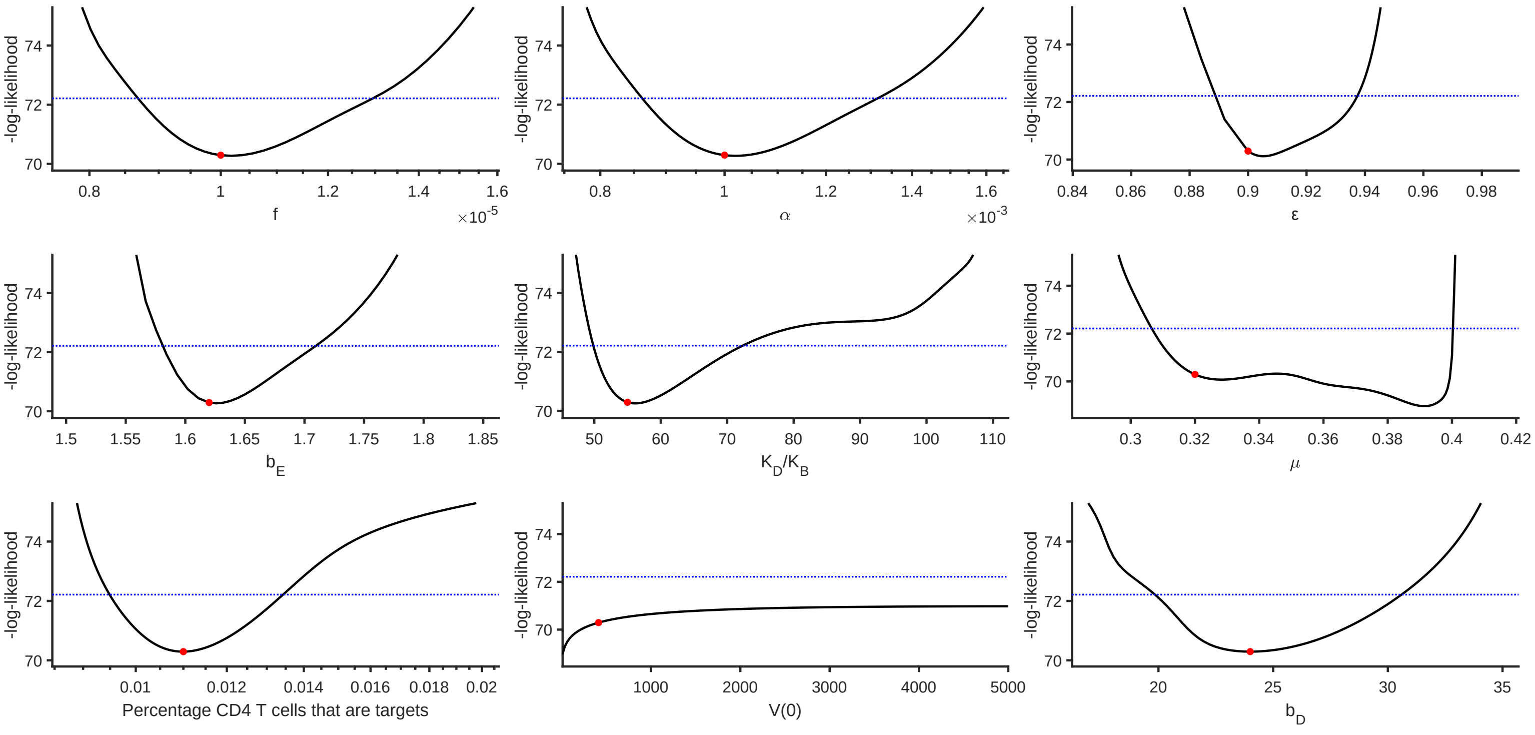

ROo13:Increased antigen presentation with clearance (APCS)

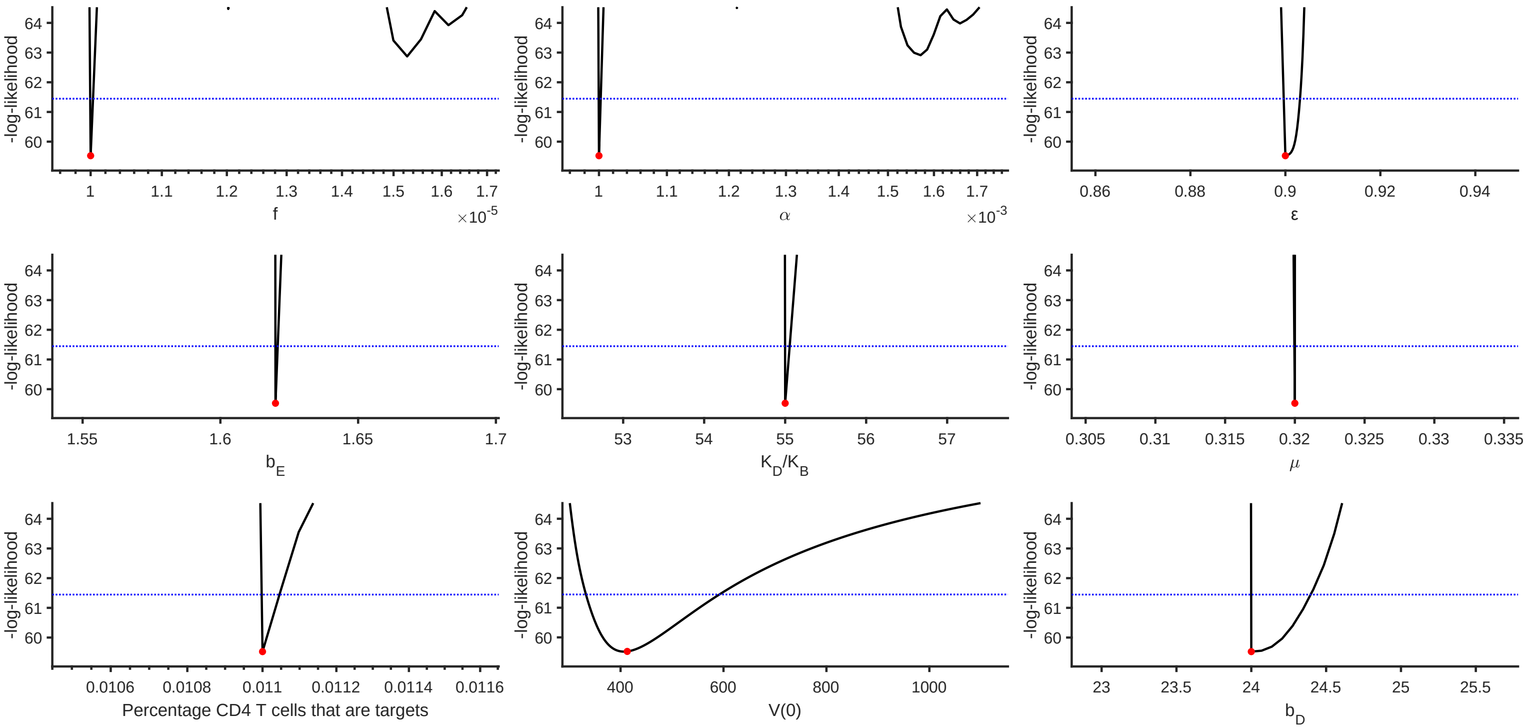

RSd14:Increased antigen presentation with clearance (APCS)

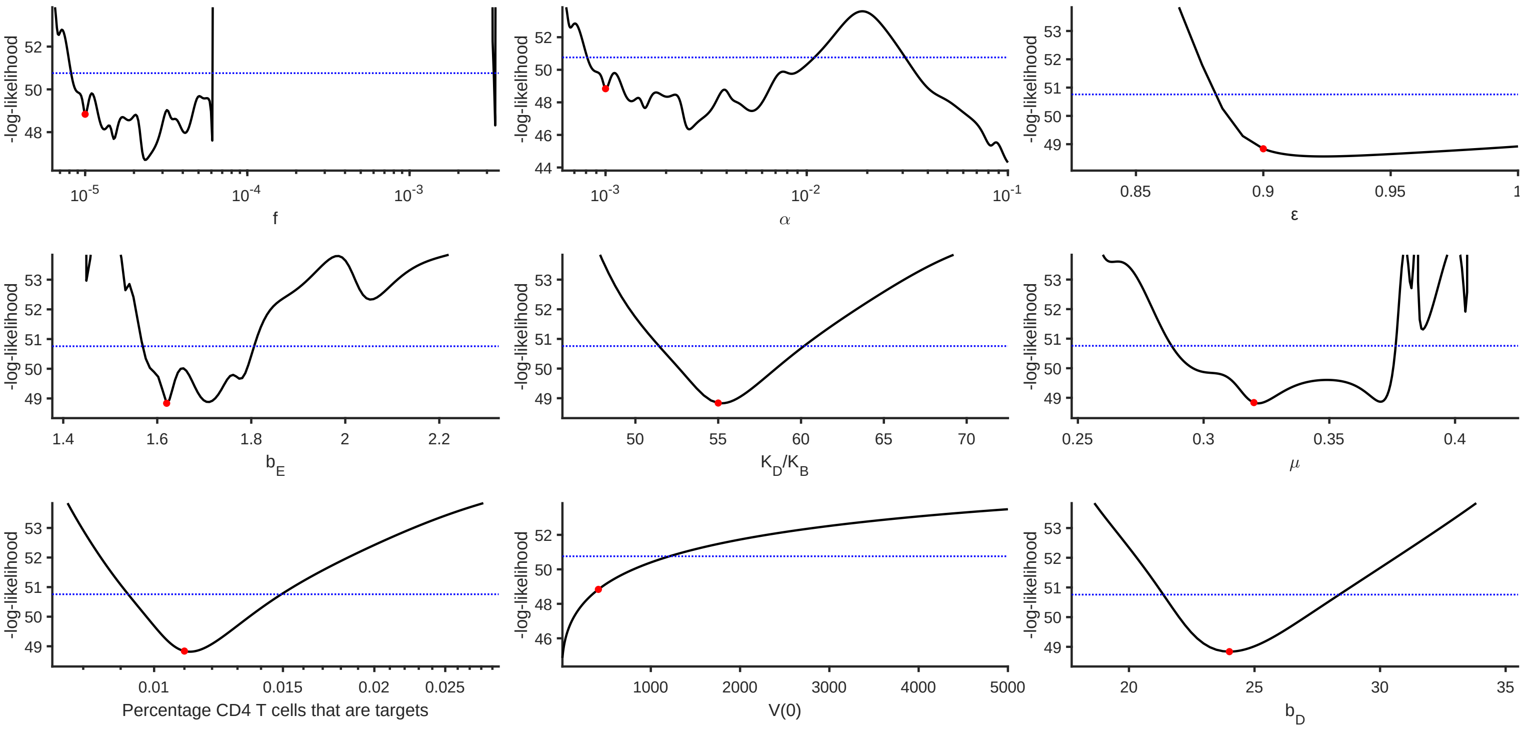

ROv14:Increased antigen presentation with clearance (APCS)

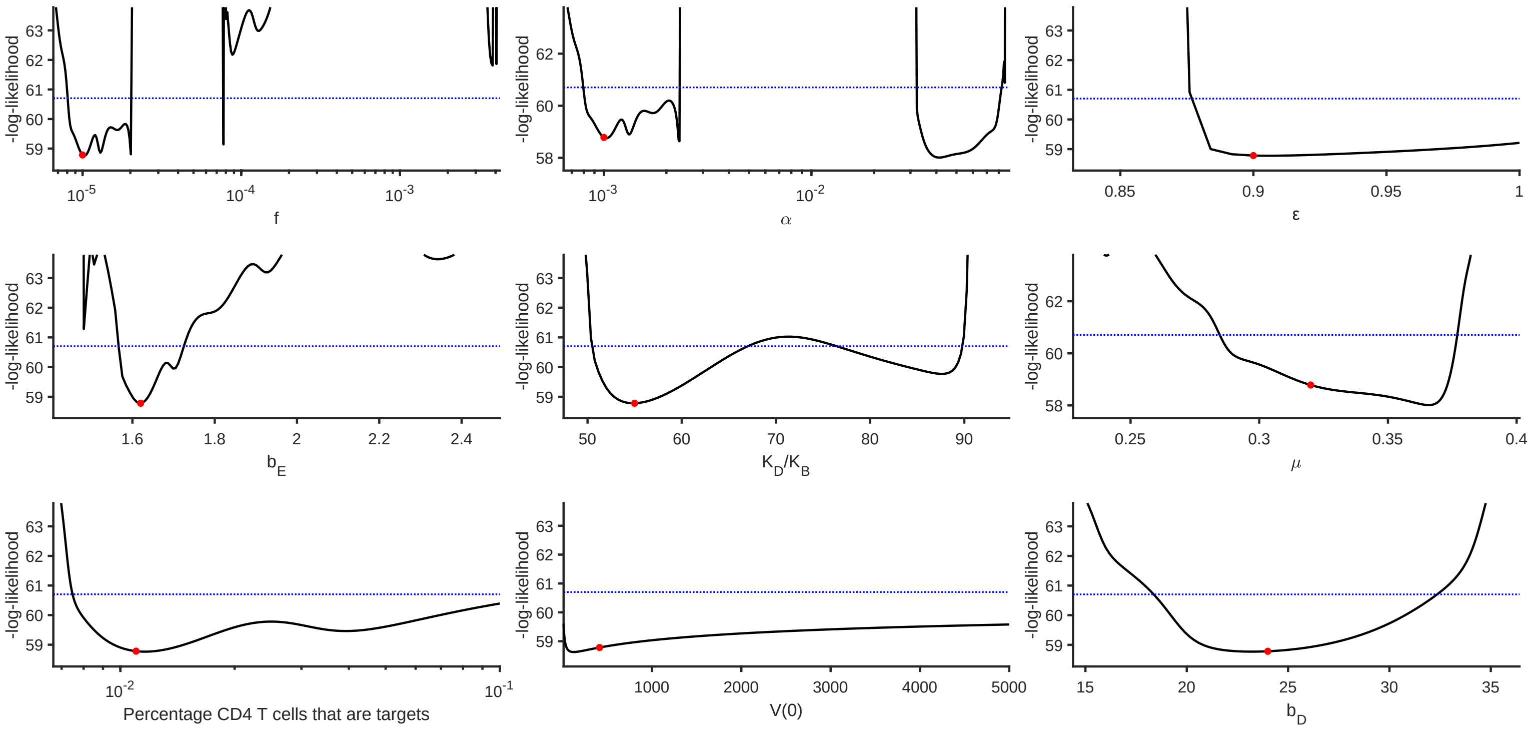

Supplement: S3 Text — (PDF) [file pcbi.1009031.s003.pdf]
