# Supplementary material for: Mechanistic basis of post-treatment control of SIV after anti-α4β7 antibody therapy
Source: PLoS Comput Biol. 2021 Jun 9;17(6):e1009031. doi: 10.1371/journal.pcbi.1009031 (PMC8189501; doi:10.1371/journal.pcbi.1009031)
Supplement: S1 Table — (PDF) [file pcbi.1009031.s004.pdf]

**S1 Table:** The aggregated log-likelihood values for the seven control macaques from the primary grid search conducted on the infected cell death rate due to viral cytopathic effects $\delta$ per day and the maximum rate of effector cell exhaustion (dE per day) for the baseline effector cell source (BL) model. The maximum log-likelihood is highlighted in yellow.

| BL Model | Infected cell death rate due to viral cytopathic effects ($\delta$ per day) | | | | | | | | | | | | | | |
|---|---|---|---|---|---|---|---|---|---|---|---|---|---|---|---|
| Maximum rate of exhaustion (dE per day) | 0.05 | 0.1 | 0.15 | 0.2 | 0.25 | 0.3 | 0.35 | 0.4 | 0.45 | 0.5 | 0.55 | 0.6 | 0.65 | 0.7 | 0.75 |
| 1 | -5669 | -5690 | -5695 | -5648 | -5673 | -5676 | -5686 | -5676 | -5665 | -5683 | -5695 | -5665 | -5745 | -5760 | -5640 |
| 1.05 | -5699 | -5743 | -5672 | -5652 | -5696 | -5641 | -5625 | -5646 | -5638 | -5643 | -5670 | -5752 | -5650 | -5721 | -5768 |
| 1.1 | -5719 | -5648 | -5664 | -5655 | -5778 | -5688 | -5676 | -5644 | -5671 | -5757 | -5680 | -5740 | -5677 | -5674 | -5703 |
| 1.15 | -5494 | -5739 | -5734 | -5678 | -5680 | -5691 | -5626 | -5675 | -5654 | -5657 | -5669 | -5708 | -5657 | -5611 | -5646 |
| 1.2 | -5424 | -5529 | -5636 | -5659 | -5723 | -5632 | -5674 | -5641 | -5773 | -5701 | -5686 | -5668 | -5700 | -5736 | -5705 |
| 1.25 | -5407 | -5406 | -5615 | -5631 | -5634 | -5604 | -5658 | -5630 | -5607 | -5722 | -5628 | -5608 | -5695 | -5721 | -5721 |
| 1.3 | -5196 | -5097 | -5044 | -5016 | -5005 | -5000 | -5002 | -5004 | -5005 | -5007 | -5010 | -5015 | -5084 | -5031 | -5053 |
| 1.35 | -1300 | -1530 | -1732 | -923 | -920 | -244 | -899 | -305 | -205 | -139 | -133 | -127 | -135 | -139 | -150 |
| 1.4 | -1816 | -1157 | -1238 | -1285 | -1094 | -1658 | -1262 | -208 | -270 | -136 | -142 | -139 | -146 | -151 | -150 |
| 1.45 | -2021 | -1625 | -1655 | -1385 | -912 | -1597 | -1778 | -905 | -255 | -139 | -141 | -145 | -144 | -146 | -150 |
| 1.5 | -1693 | -2003 | -1714 | -1453 | -1372 | -1239 | -1485 | -944 | -252 | -138 | -147 | -148 | -149 | -152 | -153 |
| 1.55 | -1988 | -2027 | -1929 | -1965 | -1495 | -1896 | -1536 | -1484 | -1220 | -148 | -147 | -148 | -149 | -152 | -154 |
| 1.6 | -2011 | -1908 | -1981 | -1897 | -1825 | -1781 | -1174 | -662 | -1468 | -155 | -149 | -149 | -151 | -152 | -154 |
| 1.65 | -2010 | -2017 | -1968 | -1886 | -2061 | -1826 | -1622 | -1316 | -1450 | -151 | -151 | -151 | -152 | -154 | -155 |
| 1.7 | -2037 | -2062 | -2036 | -1995 | -2042 | -2013 | -1751 | -1241 | -1250 | -157 | -158 | -152 | -153 | -154 | -156 |
| 1.75 | -2075 | -2057 | -2009 | -2007 | -2008 | -1988 | -1914 | -1594 | -915 | -157 | -153 | -153 | -154 | -155 | -156 |
| 1.8 | -2080 | -2425 | -2117 | -2571 | -2021 | -1874 | -1864 | -1444 | -1491 | -160 | -155 | -154 | -156 | -156 | -157 |
| 1.85 | -2041 | -2042 | -2128 | -2033 | -2132 | -2101 | -1913 | -1509 | -1209 | -161 | -161 | -155 | -156 | -156 | -158 |
| 1.9 | -2437 | -2101 | -2401 | -2908 | -2091 | -2023 | -1959 | -1876 | -939 | -161 | -156 | -156 | -156 | -158 | -158 |
| 1.95 | -2176 | -2075 | -2069 | -2505 | -2138 | -2038 | -2018 | -1352 | -268 | -158 | -157 | -157 | -157 | -157 | -159 |
| 2 | -2064 | -2427 | -2090 | -2037 | -2050 | -2363 | -1914 | -1682 | -1158 | -163 | -158 | -157 | -157 | -159 | -158 |
| 2.05 | -2157 | -2086 | -2149 | -2375 | -2058 | -2050 | -2013 | -1745 | -1275 | -163 | -158 | -158 | -159 | -159 | -159 |
| 2.1 | -2573 | -2205 | -2061 | -2133 | -2376 | -2015 | -2401 | -1868 | -925 | -163 | -159 | -158 | -160 | -160 | -159 |
| 2.15 | -2426 | -2084 | -2043 | -2050 | -2373 | -2007 | -1956 | -2001 | -1276 | -161 | -159 | -159 | -161 | -159 | -159 |
| 2.2 | -2095 | -2046 | -2139 | -2946 | -2022 | -2051 | -2008 | -1987 | -1547 | -167 | -163 | -163 | -162 | -160 | -160 |
| 2.25 | -2065 | -2170 | -2511 | -2462 | -2471 | -2024 | -1972 | -1920 | -949 | -161 | -162 | -161 | -162 | -161 | -160 |
| 2.3 | -2512 | -2132 | -2468 | -2440 | -2011 | -2378 | -2098 | -1981 | -1281 | -164 | -161 | -160 | -162 | -161 | -160 |
| 2.35 | -2465 | -2436 | -2079 | -2448 | -2612 | -2507 | -2361 | -1954 | -934 | -165 | -163 | -160 | -166 | -164 | -160 |
| 2.4 | -2617 | -3023 | -2094 | -2828 | -2040 | -2024 | -2010 | -1904 | -1275 | -170 | -162 | -164 | -163 | -161 | -161 |
| 2.45 | -2554 | -2432 | -2414 | -2421 | -2369 | -2072 | -2474 | -2310 | -1081 | -166 | -166 | -165 | -162 | -162 | -161 |
| 2.5 | -2684 | -3011 | -2520 | -2920 | -2594 | -2093 | -2524 | -1932 | -1165 | -167 | -162 | -167 | -163 | -162 | -160 |
| 2.55 | -3025 | -3002 | -2853 | -2918 | -2475 | -2500 | -2020 | -1926 | -1276 | -167 | -165 | -164 | -163 | -162 | -161 |
| 2.6 | -2493 | -2916 | -2921 | -2902 | -3371 | -2941 | -2019 | -1985 | -1312 | -168 | -164 | -168 | -164 | -162 | -161 |
| 2.65 | -2646 | -3469 | -2936 | -2879 | -3384 | -3588 | -2487 | -1935 | -1061 | -163 | -166 | -168 | -163 | -162 | -161 |
| 2.7 | -2982 | -3085 | -2558 | -2498 | -3054 | -3284 | -2389 | -1944 | -1455 | -168 | -165 | -164 | -163 | -163 | -161 |
| 2.75 | -3457 | -3357 | -3381 | -2237 | -2515 | -3350 | -2406 | -1985 | -1451 | -168 | -163 | -165 | -163 | -163 | -161 |
| 2.8 | -3077 | -3504 | -3388 | -2916 | -3018 | -2902 | -2040 | -2039 | -1299 | -169 | -166 | -165 | -163 | -163 | -161 |
| 2.85 | -3459 | -3515 | -2610 | -3379 | -3836 | -2925 | -2377 | -2021 | -1641 | -169 | -165 | -165 | -165 | -163 | -162 |
| 2.9 | -2325 | -3099 | -3319 | -2643 | -3327 | -2407 | -2022 | -2017 | -1659 | -169 | -166 | -164 | -163 | -163 | -162 |
| 2.95 | -2688 | -2674 | -2965 | -2900 | -3889 | -2676 | -2815 | -2021 | -1104 | -168 | -167 | -165 | -165 | -163 | -162 |
| 3 | -5180 | -2382 | -2403 | -2865 | -2849 | -2947 | -2037 | -2425 | -1878 | -169 | -171 | -165 | -165 | -163 | -162 |
| 3.05 | -4898 | -2740 | -2443 | -2623 | -2846 | -2975 | -2365 | -2550 | -1186 | -169 | -171 | -167 | -164 | -163 | -162 |
| 3.1 | -4589 | -2719 | -2524 | -2227 | -3318 | -2872 | -2393 | -1974 | -1758 | -170 | -167 | -165 | -165 | -163 | -162 |
| 3.15 | -5178 | -2647 | -2508 | -2465 | -2248 | -3048 | -2514 | -2047 | -1096 | -168 | -170 | -165 | -165 | -163 | -162 |
| 3.2 | -5177 | -4528 | -2279 | -2159 | -2228 | -2188 | -2859 | -2037 | -1183 | -168 | -170 | -168 | -165 | -163 | -162 |
| 3.25 | -5173 | -4707 | -2367 | -2328 | -2364 | -2257 | -2087 | -2352 | -1566 | -170 | -166 | -166 | -165 | -163 | -162 |
| 3.3 | -4544 | -5181 | -2568 | -2432 | -2322 | -2306 | -2081 | -1952 | -1939 | -170 | -170 | -168 | -165 | -163 | -162 |
| 3.35 | -5171 | -4184 | -2395 | -2441 | -2334 | -2126 | -2111 | -1941 | -1842 | -171 | -169 | -168 | -165 | -163 | -162 |
| 3.4 | -5172 | -4718 | -2504 | -2379 | -2274 | -2160 | -2082 | -1947 | -1573 | -172 | -173 | -166 | -165 | -163 | -162 |
| 3.45 | -5171 | -5173 | -5086 | -2376 | -2352 | -2155 | -2083 | -1951 | -1547 | -171 | -170 | -167 | -165 | -163 | -162 |
| 3.5 | -5182 | -5167 | -5082 | -2542 | -2246 | -2185 | -2105 | -1955 | -1585 | -172 | -167 | -167 | -165 | -164 | -162 |
| 3.55 | -5177 | -5176 | -5094 | -2581 | -2293 | -2171 | -2104 | -1993 | -1588 | -171 | -167 | -166 | -165 | -163 | -162 |
| 3.6 | -5182 | -5173 | -5094 | -2431 | -2113 | -2157 | -2134 | -1965 | -1560 | -171 | -170 | -167 | -167 | -163 | -162 |
| 3.65 | -5174 | -5186 | -5089 | -2490 | -2316 | -2301 | -2109 | -1995 | -1546 | -171 | -168 | -167 | -165 | -163 | -162 |
| 3.7 | -5200 | -5171 | -5094 | -2285 | -2304 | -2288 | -2144 | -1998 | -1550 | -171 | -169 | -167 | -166 | -163 | -162 |
| 3.75 | -5192 | -5174 | -5089 | -2663 | -2293 | -2261 | -2076 | -1998 | -1650 | -173 | -168 | -171 | -165 | -163 | -162 |
| 3.8 | -5183 | -5175 | -5089 | -2462 | -2559 | -2307 | -2130 | -1993 | -1672 | -172 | -170 | -167 | -165 | -163 | -162 |
| 3.85 | -5178 | -5173 | -5093 | -4659 | -2497 | -2267 | -2158 | -2005 | -1612 | -171 | -168 | -168 | -165 | -163 | -162 |
| 3.9 | -5188 | -5173 | -5092 | -5039 | -2427 | -2285 | -2117 | -2003 | -1606 | -173 | -170 | -168 | -166 | -164 | -162 |
| 3.95 | -5182 | -5177 | -5095 | -5040 | -2557 | -2298 | -2121 | -2010 | -1637 | -173 | -169 | -168 | -166 | -163 | -162 |
| 4 | -5186 | -5184 | -5090 | -5043 | -2364 | -2453 | -2079 | -1995 | -1638 | -173 | -171 | -169 | -165 | -164 | -162 |
| 4.05 | -5171 | -5178 | -5092 | -5039 | -2503 | -2288 | -2218 | -2021 | -1659 | -171 | -170 | -170 | -165 | -163 | -162 |
| 4.1 | -5185 | -5180 | -5093 | -5043 | -2504 | -2096 | -2140 | -2013 | -1761 | -173 | -170 | -170 | -165 | -163 | -163 |
| 4.15 | -5176 | -5164 | -5093 | -5040 | -2670 | -2311 | -2218 | -2038 | -1678 | -173 | -170 | -167 | -165 | -164 | -163 |
| 4.2 | -5178 | -5178 | -5096 | -5041 | -2574 | -2310 | -2223 | -2045 | -1709 | -172 | -169 | -168 | -166 | -163 | -162 |
| 4.25 | -5186 | -5178 | -5100 | -5037 | -2562 | -2259 | -2226 | -2021 | -1705 | -174 | -170 | -168 | -165 | -164 | -162 |
| 4.3 | -5182 | -5186 | -5100 | -5042 | -2417 | -2211 | -2144 | -2045 | -1698 | -174 | -170 | -169 | -166 | -164 | -163 |
| 4.35 | -5177 | -5196 | -5094 | -5041 | -5013 | -2262 | -2176 | -2033 | -1708 | -175 | -171 | -169 | -166 | -164 | -164 |
| 4.4 | -5199 | -5178 | -5096 | -5043 | -5014 | -2364 | -2236 | -2043 | -1719 | -173 | -171 | -169 | -165 | -164 | -162 |
| 4.45 | -5191 | -5177 | -5096 | -5042 | -5013 | -2459 | -2293 | -2025 | -1699 | -174 | -171 | -168 | -167 | -164 | -163 |
| 4.5 | -5189 | -5173 | -5093 | -5042 | -5013 | -2588 | -2302 | -2027 | -1722 | -175 | -170 | -168 | -167 | -164 | -162 |
| 4.55 | -5192 | -5174 | -5100 | -5042 | -5016 | -2423 | -2213 | -2044 | -1734 | -174 | -171 | -169 | -166 | -164 | -163 |
| 4.6 | -5199 | -5189 | -5096 | -5044 | -5017 | -2474 | -2192 | -2052 | -1699 | -174 | -171 | -171 | -166 | -164 | -163 |
| 4.65 | -5184 | -5192 | -5101 | -5048 | -5018 | -2330 | -2210 | -2050 | -1735 | -174 | -171 | -168 | -165 | -164 | -163 |
| 4.7 | -5182 | -5171 | -5098 | -5042 | -5019 | -2502 | -2059 | -2056 | -1795 | -174 | -171 | -168 | -165 | -164 | -163 |
| 4.75 | -5191 | -5180 | -5100 | -5041 | -5016 | -2535 | -2245 | -2067 | -1737 | -174 | -171 | -168 | -166 | -164 | -163 |
| 4.8 | -5185 | -5177 | -5092 | -5042 | -5018 | -2534 | -2252 | -2082 | -1789 | -176 | -172 | -170 | -166 | -164 | -162 |
| 4.85 | -5183 | -5154 | -5109 | -5045 | -5019 | -2535 | -2144 | -2061 | -1802 | -175 | -172 | -171 | -166 | -164 | -162 |
| 4.9 | -5199 | -5174 | -5100 | -5042 | -5017 | -2588 | -2284 | -2063 | -1789 | -174 | -172 | -168 | -165 | -164 | -163 |
| 4.95 | -5208 | -5176 | -5098 | -5039 | -5019 | -2535 | -2243 | -2057 | -1787 | -175 | -172 | -169 | -166 | -164 | -163 |
| 5 | -5203 | -5185 | -5090 | -5045 | -5022 | -2539 | -2345 | -2090 | -1819 | -174 | -172 | -169 | -167 | -164 | -163 |
| 5.05 | -5201 | -5171 | -5101 | -5043 | -5017 | -2597 | -2388 | -2108 | -1777 | -175 | -172 | -169 | -166 | -164 | -162 |
| 5.1 | -5204 | -5178 | -5096 | -5041 | -5023 | -4435 | -2230 | -2215 | -1766 | -174 | -172 | -170 | -167 | -164 | -163 |
| 5.15 | -5207 | -5180 | -5108 | -5043 | -5018 | -5010 | -2462 | -2137 | -1768 | -175 | -172 | -168 | -165 | -164 | -163 |
| 5.2 | -5211 | -5190 | -5097 | -5045 | -5026 | -5011 | -2448 | -2181 | -1783 | -175 | -172 | -168 | -167 | -164 | -163 |
| 5.25 | -5199 | -5182 | -5104 | -5042 | -5020 | -5011 | -2380 | -2206 | -1773 | -175 | -173 | -169 | -166 | -164 | -163 |
| 5.3 | -5199 | -5180 | -5095 | -5044 | -5017 | -5012 | -2436 | -2103 | -1822 | -176 | -172 | -168 | -166 | -164 | -163 |
| 5.35 | -5195 | -5174 | -5101 | -5044 | -5021 | -5012 | -2424 | -2143 | -1795 | -175 | -173 | -168 | -167 | -164 | -163 |
| 5.4 | -5202 | -5170 | -5098 | -5041 | -5017 | -5011 | -2290 | -2126 | -1801 | -176 | -172 | -168 | -167 | -164 | -163 |
| 5.45 | -5212 | -5172 | -5099 | -5042 | -5021 | -4451 | -2370 | -2147 | -1821 | -176 | -172 | -169 | -166 | -164 | -163 |
| 5.5 | -5208 | -5165 | -5101 | -5044 | -5021 | -5012 | -2480 | -2104 | -1800 | -176 | -174 | -169 | -166 | -164 | -163 |
| 5.55 | -5205 | -5182 | -5105 | -5045 | -5022 | -5013 | -2404 | -2133 | -1847 | -175 | -172 | -170 | -166 | -164 | -163 |
| 5.6 | -5194 | -5180 | -5100 | -5044 | -5021 | -5013 | -2301 | -2126 | -1819 | -176 | -173 | -169 | -167 | -164 | -165 |
| 5.65 | -5205 | -5173 | -5103 | -5046 | -5023 | -5015 | -2468 | -2123 | -1806 | -176 | -172 | -169 | -166 | -164 | -165 |
| 5.7 | -5205 | -5176 | -5102 | -5043 | -5021 | -4634 | -2390 | -2088 | -1828 | -176 | -173 | -169 | -168 | -164 | -163 |
| 5.75 | -5205 | -5181 | -5101 | -5041 | -5023 | -4461 | -2383 | -2098 | -1861 | -176 | -172 | -168 | -166 | -164 | -163 |
| 5.8 | -5209 | -5190 | -5105 | -5043 | -5023 | -5014 | -2163 | -2115 | -1852 | -176 | -173 | -169 | -166 | -165 | -165 |
| 5.85 | -5211 | -5185 | -5097 | -5046 | -5027 | -5013 | -2425 | -2186 | -1823 | -176 | -173 | -169 | -166 | -164 | -165 |
| 5.9 | -5219 | -5168 | -5105 | -5045 | -5020 | -5015 | -2435 | -2118 | -1855 | -176 | -173 | -169 | -166 | -164 | -165 |
| 5.95 | -5209 | -5181 | -5099 | -5048 | -5022 | -5012 | -2355 | -2153 | -1837 | -176 | -172 | -170 | -166 | -164 | -165 |
| 6 | -5215 | -5185 | -5101 | -5044 | -5021 | -5014 | -2273 | -2128 | -1865 | -176 | -172 | -169 | -166 | -164 | -163 |
