# Supplementary material for: Mechanistic basis of post-treatment control of SIV after anti-α4β7 antibody therapy
Source: PLoS Comput Biol. 2021 Jun 9;17(6):e1009031. doi: 10.1371/journal.pcbi.1009031 (PMC8189501; doi:10.1371/journal.pcbi.1009031)
Supplement: S2 Table — (PDF) [file pcbi.1009031.s005.pdf]

S2 Table: The aggregated log-likelihood values for the seven control macaques from the primary grid search conducted on the infected cell death rate due to viral cytopathic effects δ per day and the maximum rate of effector cell exhaustion (d_E per day) for the saturated effector cell source (SS) model. The maximum log-likelihood is highlighted in yellow.

SS Model	Infected cell death rate due to viral cytopathic effects (δ per day)														
Maximum rate of exhaustion (d_E per day)	0.05	0.1	0.15	0.2	0.25	0.3	0.35	0.4	0.45	0.5	0.55	0.6	0.65	0.7	0.75
1	-5708	-5675	-5660	-5634	-5627	-5669	-5688	-5596	-5617	-5642	-5661	-5682	-5727	-5681	-5747
1.05	-5512	-5676	-5607	-5653	-5628	-5681	-5692	-5628	-5623	-5658	-5635	-5643	-5714	-5717	-5755
1.1	-5506	-5653	-5659	-5617	-5626	-5672	-5655	-5639	-5645	-5654	-5713	-5681	-5688	-5673	-5639
1.15	-5437	-5641	-5652	-5652	-5619	-5646	-5651	-5663	-5644	-5648	-5634	-5773	-5732	-5711	-5713
1.2	-5367	-5410	-5466	-5626	-5646	-5631	-5616	-5650	-5593	-5594	-5636	-5732	-5706	-5727	-5656
1.25	-5401	-5421	-5478	-5607	-5615	-5593	-5653	-5604	-5649	-5687	-5632	-5645	-5688	-5717	-5729
1.3	-5203	-5120	-5054	-5021	-5008	-5004	-5003	-5007	-5008	-5009	-5010	-5014	-5020	-5027	-5041
1.35	-1617	-1296	-1145	-230	-345	-839	-161	-152	-139	-138	-134	-133	-134	-137	-148
1.4	-1613	-1558	-1436	-276	-951	-264	-167	-163	-148	-138	-140	-149	-142	-146	-154
1.45	-1659	-1559	-1476	-1284	-1201	-864	-168	-168	-171	-147	-146	-147	-152	-149	-153
1.5	-1761	-1661	-1604	-1263	-301	-169	-245	-167	-166	-147	-154	-149	-148	-155	-152
1.55	-1797	-1550	-1594	-1445	-172	-169	-169	-167	-166	-154	-152	-153	-152	-156	-157
1.6	-1815	-1850	-1717	-1351	-1166	-860	-168	-168	-166	-156	-156	-154	-153	-155	-157
1.65	-1868	-1552	-1806	-1666	-737	-493	-169	-168	-167	-156	-155	-155	-154	-156	-158
1.7	-1681	-1670	-1708	-599	-714	-720	-170	-168	-167	-156	-156	-155	-157	-157	-157
1.75	-1736	-2017	-1900	-1333	-1395	-336	-255	-168	-169	-161	-156	-156	-158	-157	-158
1.8	-1695	-1973	-1880	-1704	-1427	-171	-861	-169	-168	-158	-157	-158	-157	-157	-158
1.85	-1690	-1828	-1684	-1957	-1427	-184	-177	-169	-168	-158	-158	-158	-157	-157	-158
1.9	-1605	-1979	-1914	-1667	-1118	-172	-170	-170	-168	-161	-158	-159	-157	-157	-158
1.95	-1876	-1931	-1723	-1180	-174	-171	-191	-179	-168	-158	-158	-157	-159	-158	-158
2	-1744	-1922	-1581	-1188	-183	-171	-171	-169	-168	-159	-159	-160	-158	-158	-159
2.05	-1706	-1912	-2039	-195	-175	-171	-171	-173	-168	-159	-159	-161	-159	-158	-159
2.1	-1973	-1856	-1169	-205	-175	-171	-171	-175	-168	-160	-162	-159	-159	-158	-159
2.15	-2140	-1861	-359	-193	-175	-171	-171	-170	-169	-160	-162	-161	-159	-159	-159
2.2	-1874	-2082	-222	-314	-175	-173	-171	-170	-168	-160	-162	-159	-159	-159	-159
2.25	-2009	-1324	-231	-197	-188	-172	-171	-171	-168	-160	-160	-161	-159	-159	-159
2.3	-1972	-359	-219	-326	-318	-171	-172	-170	-170	-161	-160	-160	-159	-159	-159
2.35	-2151	-240	-360	-189	-175	-171	-171	-170	-170	-164	-160	-160	-160	-159	-159
2.4	-1653	-228	-221	-198	-360	-184	-171	-170	-169	-161	-161	-161	-161	-159	-159
2.45	-236	-235	-224	-321	-175	-171	-178	-170	-169	-161	-161	-163	-160	-159	-159
2.5	-233	-227	-214	-188	-175	-313	-171	-170	-170	-162	-161	-160	-160	-159	-159
2.55	-236	-233	-222	-186	-175	-171	-171	-171	-170	-165	-161	-163	-160	-160	-160
2.6	-232	-224	-213	-187	-175	-171	-171	-175	-170	-164	-161	-163	-160	-160	-160
2.65	-235	-227	-209	-185	-174	-172	-170	-171	-170	-165	-161	-160	-160	-160	-160
2.7	-229	-223	-221	-186	-174	-185	-171	-170	-170	-164	-162	-161	-160	-160	-160
2.75	-236	-223	-208	-185	-175	-171	-170	-170	-172	-164	-163	-164	-161	-160	-160
2.8	-235	-222	-208	-185	-174	-171	-170	-170	-170	-164	-163	-162	-161	-160	-160
2.85	-231	-221	-206	-184	-174	-171	-170	-171	-172	-164	-163	-161	-162	-160	-160
2.9	-234	-220	-206	-197	-174	-171	-170	-171	-170	-165	-165	-162	-161	-160	-160
2.95	-227	-224	-205	-184	-174	-171	-183	-170	-170	-167	-162	-164	-162	-160	-160
3	-1424	-219	-203	-184	-174	-171	-170	-170	-170	-165	-165	-163	-161	-160	-160
3.05	-688	-223	-204	-183	-174	-171	-170	-170	-169	-165	-165	-161	-161	-161	-160
3.1	-594	-223	-206	-183	-173	-171	-170	-170	-169	-165	-163	-162	-161	-160	-160
3.15	-561	-217	-204	-182	-173	-171	-180	-170	-169	-165	-166	-162	-162	-161	-161
3.2	-1083	-1657	-199	-183	-173	-171	-170	-170	-170	-166	-166	-162	-161	-162	-160
3.25	-1906	-591	-198	-183	-180	-171	-170	-170	-169	-164	-164	-162	-163	-161	-160
3.3	-502	-222	-199	-181	-174	-170	-170	-181	-169	-166	-164	-167	-161	-162	-161
3.35	-1377	-1669	-209	-181	-173	-170	-170	-170	-169	-165	-165	-163	-163	-161	-161
3.4	-688	-1535	-208	-181	-173	-170	-170	-170	-178	-165	-166	-165	-164	-161	-160
3.45	-1595	-1298	-300	-180	-183	-170	-182	-170	-169	-166	-166	-163	-164	-161	-161
3.5	-1153	-1159	-655	-179	-173	-170	-170	-170	-169	-166	-164	-163	-162	-162	-160
3.55	-2053	-631	-900	-179	-173	-170	-170	-170	-174	-165	-167	-163	-162	-161	-160
3.6	-1437	-433	-577	-246	-173	-170	-170	-170	-169	-164	-167	-165	-162	-161	-161
3.65	-2019	-1398	-1569	-187	-172	-170	-170	-170	-169	-166	-164	-164	-163	-162	-160
3.7	-1288	-891	-1129	-189	-172	-170	-170	-172	-182	-166	-166	-164	-162	-161	-160
3.75	-516	-1497	-762	-178	-172	-170	-170	-170	-169	-167	-166	-163	-162	-161	-161
3.8	-1098	-1128	-681	-536	-172	-170	-170	-170	-169	-170	-166	-163	-163	-161	-161
3.85	-1238	-354	-1992	-1150	-172	-170	-170	-170	-170	-165	-166	-163	-162	-161	-161
3.9	-544	-518	-353	-293	-172	-170	-181	-170	-171	-166	-165	-164	-164	-162	-161
3.95	-527	-594	-1002	-501	-263	-170	-170	-170	-169	-166	-165	-166	-164	-161	-161
4	-586	-1341	-989	-599	-240	-184	-170	-170	-171	-166	-164	-163	-163	-161	-161
4.05	-212	-543	-668	-1567	-172	-170	-170	-171	-169	-166	-164	-163	-163	-163	-161
4.1	-801	-907	-831	-564	-171	-170	-170	-170	-172	-168	-164	-165	-162	-161	-161
4.15	-545	-298	-1747	-421	-171	-170	-169	-170	-169	-167	-166	-163	-162	-161	-161
4.2	-704	-385	-879	-1529	-241	-170	-170	-169	-169	-166	-167	-163	-163	-161	-161
4.25	-1250	-474	-829	-1615	-188	-170	-169	-169	-169	-167	-165	-165	-162	-161	-161
4.3	-354	-305	-1121	-539	-616	-170	-169	-169	-169	-166	-164	-164	-163	-162	-161
4.35	-384	-726	-844	-1068	-1166	-170	-169	-169	-174	-166	-164	-166	-162	-162	-161
4.4	-1294	-1226	-420	-1707	-1340	-170	-170	-169	-169	-170	-164	-164	-163	-161	-161
4.45	-1325	-702	-666	-1674	-661	-170	-169	-169	-169	-167	-165	-166	-163	-162	-161
4.5	-537	-333	-889	-743	-1153	-170	-169	-170	-169	-167	-168	-164	-163	-162	-161
4.55	-508	-469	-1268	-546	-767	-170	-169	-169	-169	-171	-167	-164	-163	-162	-161
4.6	-269	-446	-863	-1564	-1660	-170	-169	-174	-169	-172	-165	-165	-163	-163	-161
4.65	-201	-1357	-893	-174	-1210	-169	-169	-169	-169	-171	-165	-164	-164	-162	-161
4.7	-1161	-536	-1252	-1483	-1809	-169	-169	-169	-169	-167	-165	-164	-164	-162	-161
4.75	-769	-190	-1301	-1692	-291	-169	-169	-169	-169	-173	-167	-164	-164	-162	-162
4.8	-377	-1187	-1143	-703	-291	-169	-169	-169	-169	-166	-169	-164	-164	-162	-161
4.85	-1252	-1026	-543	-1239	-930	-169	-169	-169	-169	-167	-167	-164	-163	-164	-162
4.9	-326	-545	-464	-874	-345	-169	-169	-169	-169	-167	-169	-164	-163	-162	-162
4.95	-718	-1195	-792	-875	-1179	-169	-169	-169	-169	-167	-167	-167	-165	-162	-161
5	-238	-546	-664	-279	-1160	-169	-169	-170	-168	-170	-168	-164	-164	-163	-162
5.05	-1028	-501	-348	-724	-756	-880	-169	-169	-169	-168	-167	-165	-165	-162	-162
5.1	-493	-1559	-1468	-837	-959	-1093	-169	-169	-169	-167	-169	-167	-166	-163	-162
5.15	-208	-863	-346	-1091	-903	-1240	-169	-169	-169	-168	-167	-165	-163	-162	-162
5.2	-1273	-505	-715	-1015	-993	-864	-169	-169	-169	-169	-168	-166	-166	-162	-161
5.25	-903	-1018	-467	-1164	-877	-732	-169	-169	-168	-172	-167	-166	-163	-162	-162
5.3	-322	-1149	-925	-515	-1089	-169	-169	-169	-172	-168	-166	-165	-166	-163	-162
5.35	-1016	-763	-1499	-339	-711	-533	-169	-169	-169	-171	-167	-164	-164	-162	-161
5.4	-895	-294	-403	-1466	-425	-285	-170	-169	-168	-173	-166	-164	-164	-163	-162
5.45	-333	-1646	-710	-1271	-901	-579	-237	-169	-180	-170	-168	-167	-165	-162	-162
5.5	-197	-184	-728	-1065	-1285	-279	-237	-169	-168	-172	-167	-166	-164	-162	-161
5.55	-295	-359	-1220	-992	-1218	-295	-238	-169	-168	-168	-167	-165	-164	-164	-163
5.6	-470	-251	-446	-623	-581	-171	-170	-169	-168	-169	-168	-165	-164	-162	-162
5.65	-652	-641	-1421	-1389	-325	-308	-215	-169	-168	-168	-167	-165	-164	-162	-162
5.7	-988	-305	-1295	-333	-512	-249	-169	-182	-168	-168	-170	-166	-164	-162	-161
5.75	-202	-524	-374	-1075	-1048	-237	-169	-169	-168	-170	-168	-168	-165	-163	-162
5.8	-188	-466	-900	-282	-1390	-258	-169	-169	-170	-171	-168	-167	-165	-162	-162
5.85	-487	-499	-754	-623	-1560	-479	-169	-169	-171	-168	-166	-168	-164	-163	-162
5.9	-188	-1415	-1030	-834	-1095	-400	-186	-169	-171	-168	-166	-165	-164	-162	-162
5.95	-196	-905	-709	-1518	-1331	-179	-245	-169	-168	-168	-166	-165	-164	-163	-162
6	-574	-180	-647	-313	-632	-421	-183	-169	-168	-168	-166	-166	-164	-162	-162
