# Supplementary material for: Mechanistic basis of post-treatment control of SIV after anti-α4β7 antibody therapy
Source: PLoS Comput Biol. 2021 Jun 9;17(6):e1009031. doi: 10.1371/journal.pcbi.1009031 (PMC8189501; doi:10.1371/journal.pcbi.1009031)
Supplement: S3 Table — (PDF) [file pcbi.1009031.s006.pdf]

S3 Table: The aggregated log-likelihood values for the seven control macaques from the primary grid search conducted on the infected cell death rate due to viral cytopathic effects δ per day and the maximum rate of effector cell exhaustion (d_E per day) for the antigen presenting cell effector cell source (APCS) model. The maximum log-likelihood is highlighted in yellow.

APCS Model		Infected cell death rate due to viral cytopathic effects (δ per day)														
Maximum rate of exhaustion (d_E per day)		0.05	0.1	0.15	0.2	0.25	0.3	0.35	0.4	0.45	0.5	0.55	0.6	0.65	0.7	0.75
	1	-5478	-5558	-5563	-5536	-5618	-5688	-5652	-5623	-5615	-5629	-5684	-5678	-5647	-5684	-5622
	1.05	-5469	-5660	-5491	-5616	-5637	-5615	-5658	-5620	-5646	-5648	-5626	-5617	-5618	-5607	-5655
	1.1	-5398	-5563	-5484	-5608	-5595	-5635	-5617	-5662	-5623	-5639	-5611	-5603	-5611	-5644	-5642
	1.15	-5365	-5400	-5433	-5441	-5630	-5616	-5659	-5637	-5649	-5637	-5604	-5619	-5682	-5692	-5729
	1.2	-5339	-5359	-5489	-5578	-5623	-5564	-5713	-5620	-5604	-5663	-5596	-5559	-5656	-5609
	1.25	-5325	-5324	-5392	-5529	-5620	-5465	-5498	-5628	-5589	-5576	-5625	-5628	-5682	-5541	-5700
	1.3	-5174	-5065	-5047	-5026	-5018	-5010	-5009	-5010	-5013	-5014	-5018	-5018	-5023	-5034	-5043
	1.35	-177	-159	-152	-154	-155	-156	-157	-159	-156	-156	-155	-157	-157	-159	-161
	1.4	-174	-156	-155	-156	-159	-158	-159	-158	-157	-157	-157	-158	-158	-159	-161
	1.45	-182	-157	-155	-157	-158	-159	-160	-159	-158	-158	-157	-157	-158	-159	-161
	1.5	-173	-158	-155	-157	-158	-159	-159	-163	-157	-158	-157	-157	-158	-159	-160
	1.55	-170	-154	-153	-155	-157	-158	-158	-158	-159	-157	-158	-157	-157	-159	-160
	1.6	-169	-150	-150	-153	-155	-157	-159	-157	-157	-156	-156	-156	-158	-159	-161
	1.65	-172	-148	-149	-152	-152	-155	-156	-156	-157	-155	-156	-156	-157	-158	-160
	1.7	-178	-147	-147	-150	-150	-152	-155	-155	-157	-155	-155	-156	-157	-158	-160
	1.75	-163	-144	-145	-146	-151	-152	-152	-154	-154	-154	-155	-155	-157	-158	-160
	1.8	-182	-142	-142	-144	-146	-151	-151	-151	-153	-153	-154	-155	-156	-157	-159
	1.85	-165	-150	-141	-145	-146	-147	-147	-149	-151	-152	-153	-155	-155	-157	-159
	1.9	-171	-144	-139	-144	-142	-146	-147	-147	-151	-150	-153	-154	-155	-157	-158
	1.95	-164	-141	-138	-141	-142	-142	-144	-146	-149	-151	-152	-154	-155	-156	-158
	2	-166	-139	-138	-140	-141	-144	-145	-149	-147	-150	-152	-153	-154	-156	-158
	2.05	-163	-138	-137	-138	-140	-141	-142	-146	-149	-148	-152	-153	-154	-156	-158
	2.1	-160	-137	-137	-137	-139	-140	-142	-145	-147	-150	-151	-155	-154	-155	-157
	2.15	-160	-138	-135	-136	-138	-139	-141	-144	-145	-149	-150	-152	-154	-155	-157
	2.2	-163	-137	-136	-136	-137	-139	-140	-143	-146	-147	-149	-152	-153	-155	-157
	2.25	-166	-137	-135	-135	-140	-140	-140	-143	-144	-147	-149	-151	-153	-155	-157
	2.3	-162	-138	-134	-135	-137	-138	-140	-142	-144	-146	-148	-151	-153	-155	-157
	2.35	-159	-135	-134	-135	-135	-138	-140	-141	-144	-145	-149	-151	-153	-154	-157
	2.4	-161	-135	-133	-134	-136	-137	-139	-140	-143	-145	-149	-151	-152	-154	-157
	2.45	-159	-137	-134	-135	-136	-137	-139	-141	-143	-145	-148	-153	-152	-154	-157
	2.5	-158	-136	-132	-134	-135	-136	-139	-140	-144	-145	-147	-150	-152	-154	-156
	2.55	-159	-134	-133	-134	-134	-137	-138	-140	-142	-144	-148	-150	-152	-154	-156
	2.6	-159	-134	-133	-134	-135	-136	-137	-139	-142	-144	-149	-150	-152	-154	-156
	2.65	-158	-133	-134	-133	-134	-136	-138	-139	-141	-144	-146	-150	-152	-154	-156
	2.7	-177	-133	-131	-133	-135	-136	-138	-139	-142	-145	-148	-149	-152	-154	-156
	2.75	-165	-133	-132	-132	-134	-136	-137	-139	-142	-144	-148	-149	-151	-154	-156
	2.8	-163	-136	-130	-132	-133	-135	-137	-140	-142	-144	-146	-149	-151	-153	-156
	2.85	-163	-133	-130	-131	-133	-136	-136	-140	-142	-143	-147	-148	-151	-153	-156
	2.9	-159	-132	-130	-131	-134	-134	-136	-140	-142	-144	-147	-148	-151	-153	-156
	2.95	-157	-132	-129	-131	-134	-134	-136	-138	-140	-142	-146	-150	-151	-153	-156
	3	-158	-133	-129	-131	-134	-134	-136	-138	-140	-144	-147	-150	-151	-153	-156
	3.05	-157	-132	-129	-130	-132	-133	-137	-139	-140	-144	-145	-148	-151	-153	-155
	3.1	-160	-133	-129	-131	-132	-133	-135	-138	-141	-142	-145	-148	-151	-153	-155
	3.15	-165	-135	-129	-131	-131	-134	-135	-138	-139	-141	-145	-148	-151	-153	-155
	3.2	-163	-136	-129	-130	-132	-134	-135	-138	-139	-143	-146	-148	-150	-153	-155
	3.25	-166	-132	-129	-130	-134	-133	-134	-137	-139	-142	-144	-147	-151	-153	-155
	3.3	-164	-132	-129	-132	-133	-132	-136	-136	-139	-141	-146	-147	-151	-153	-155
	3.35	-164	-132	-128	-129	-131	-133	-134	-136	-139	-142	-147	-148	-151	-153	-155
	3.4	-169	-132	-128	-129	-131	-132	-135	-137	-139	-141	-146	-148	-150	-153	-155
	3.45	-162	-135	-128	-129	-131	-134	-134	-136	-139	-142	-144	-148	-150	-152	-155
	3.5	-161	-131	-128	-129	-131	-132	-135	-137	-139	-141	-145	-148	-151	-153	-155
	3.55	-162	-135	-131	-129	-130	-132	-134	-138	-139	-141	-144	-148	-151	-153	-155
	3.6	-157	-132	-128	-129	-130	-132	-134	-136	-140	-140	-144	-147	-151	-153	-155
	3.65	-167	-131	-128	-129	-130	-132	-134	-135	-138	-140	-145	-148	-150	-153	-155
	3.7	-166	-133	-128	-129	-130	-133	-134	-135	-139	-140	-144	-147	-150	-153	-155
	3.75	-167	-132	-128	-129	-130	-132	-133	-136	-138	-141	-144	-147	-150	-153	-155
	3.8	-161	-136	-128	-132	-130	-132	-135	-135	-137	-140	-144	-150	-150	-153	-155
	3.85	-160	-132	-128	-129	-133	-134	-133	-137	-138	-141	-144	-146	-151	-153	-155
	3.9	-168	-132	-128	-129	-130	-132	-134	-135	-137	-141	-143	-145	-149	-152	-155
	3.95	-179	-132	-128	-129	-132	-131	-133	-135	-138	-142	-143	-147	-150	-152	-155
	4	-168	-132	-128	-128	-130	-131	-135	-136	-138	-141	-145	-145	-149	-152	-155
	4.05	-166	-132	-128	-128	-131	-131	-133	-135	-137	-141	-143	-147	-149	-152	-155
	4.1	-160	-136	-128	-128	-130	-134	-133	-137	-137	-141	-142	-146	-150	-152	-155
	4.15	-164	-132	-128	-128	-130	-131	-133	-137	-138	-140	-142	-146	-150	-153	-155
	4.2	-171	-132	-131	-128	-130	-131	-135	-135	-137	-140	-142	-145	-150	-152	-155
	4.25	-163	-132	-128	-128	-130	-134	-133	-134	-137	-139	-142	-146	-149	-152	-155
	4.3	-158	-132	-128	-128	-132	-132	-133	-136	-137	-140	-143	-145	-150	-152	-155
	4.35	-165	-137	-131	-128	-132	-133	-133	-134	-138	-141	-143	-146	-148	-152	-155
	4.4	-162	-133	-128	-128	-132	-131	-135	-135	-137	-140	-142	-145	-150	-152	-155
	4.45	-166	-133	-128	-128	-130	-131	-134	-136	-137	-139	-142	-146	-148	-152	-155
	4.5	-166	-133	-129	-128	-130	-131	-134	-136	-138	-140	-142	-146	-150	-152	-155
	4.55	-165	-133	-128	-128	-129	-131	-132	-135	-138	-139	-142	-146	-148	-152	-155
	4.6	-163	-132	-128	-128	-133	-131	-134	-135	-137	-139	-142	-145	-148	-152	-155
	4.65	-163	-136	-135	-128	-129	-131	-134	-135	-137	-142	-142	-145	-148	-152	-155
	4.7	-168	-132	-128	-128	-130	-131	-133	-134	-136	-139	-142	-145	-148	-151	-155
	4.75	-175	-133	-128	-128	-129	-131	-133	-135	-137	-140	-142	-145	-148	-152	-155
	4.8	-164	-138	-128	-129	-129	-131	-133	-134	-136	-140	-142	-144	-148	-151	-154
	4.85	-162	-138	-128	-131	-129	-131	-132	-135	-137	-140	-142	-145	-147	-151	-154
	4.9	-163	-137	-128	-128	-133	-131	-132	-136	-136	-139	-141	-146	-148	-152	-154
	4.95	-173	-136	-128	-128	-129	-131	-133	-135	-137	-139	-142	-144	-147	-151	-155
	5	-159	-134	-128	-128	-129	-131	-133	-135	-136	-139	-141	-144	-147	-152	-155
	5.05	-166	-133	-128	-128	-129	-131	-133	-137	-136	-139	-143	-144	-147	-151	-155
	5.1	-170	-136	-128	-128	-129	-131	-133	-135	-137	-140	-142	-145	-147	-151	-155
	5.15	-168	-150	-128	-128	-129	-131	-133	-134	-136	-140	-142	-146	-147	-151	-155
	5.2	-168	-137	-131	-128	-129	-133	-133	-134	-136	-139	-141	-145	-148	-150	-154
	5.25	-166	-134	-128	-128	-129	-131	-132	-134	-137	-139	-141	-144	-148	-151	-154
	5.3	-169	-132	-128	-128	-130	-131	-132	-134	-136	-138	-141	-144	-147	-151	-154
	5.35	-162	-132	-128	-128	-129	-131	-133	-134	-137	-140	-141	-144	-147	-151	-154
	5.4	-163	-133	-128	-129	-129	-131	-132	-134	-136	-138	-141	-144	-148	-150	-154
	5.45	-165	-132	-128	-128	-129	-131	-135	-134	-136	-138	-141	-145	-148	-152	-155
	5.5	-175	-132	-128	-128	-129	-131	-132	-134	-136	-138	-141	-145	-148	-152	-154
	5.55	-162	-134	-128	-128	-129	-133	-133	-134	-136	-138	-141	-144	-148	-151	-154
	5.6	-176	-134	-128	-128	-129	-132	-132	-134	-135	-138	-141	-145	-149	-151	-155
	5.65	-161	-132	-128	-128	-129	-132	-132	-135	-138	-138	-141	-144	-147	-151	-154
	5.7	-174	-132	-128	-134	-129	-131	-133	-135	-138	-139	-141	-145	-148	-151	-155
	5.75	-172	-133	-128	-134	-135	-131	-132	-134	-136	-138	-141	-144	-147	-150	-154
	5.8	-171	-133	-128	-128	-129	-131	-132	-134	-136	-138	-141	-144	-148	-152	-154
	5.85	-163	-132	-128	-125	-129	-131	-135	-134	-136	-138	-141	-145	-148	-152	-154
	5.9	-168	-136	-128	-128	-129	-131	-132	-134	-136	-139	-141	-147	-147	-151	-155
	5.95	-161	-134	-128	-128	-129	-131	-132	-134	-136	-138	-141	-145	-147	-151	-155
	6	-162	-132	-128	-128	-129	-131	-132	-133	-136	-138	-141	-144	-148	-150	-155
