# Supplementary material for: Mechanistic basis of post-treatment control of SIV after anti-α4β7 antibody therapy
Source: PLoS Comput Biol. 2021 Jun 9;17(6):e1009031. doi: 10.1371/journal.pcbi.1009031 (PMC8189501; doi:10.1371/journal.pcbi.1009031)
Supplement: S4 Table — (PDF) [file pcbi.1009031.s007.pdf]

S4 Table: The average AIC weight for the baseline effector cell source (BL) model, the saturated effector cell source (SS) model, and the effector cell source model dependent on antigen presenting cells (APCS).

Group	BL Model	SS Model	APCS Model
Control	0.555	0.074	0.371
Treated	0.726	0.218	0.056
All	0.646	0.151	0.203
