# Supplementary material for: Mechanistic basis of post-treatment control of SIV after anti-α4β7 antibody therapy
Source: PLoS Comput Biol. 2021 Jun 9;17(6):e1009031. doi: 10.1371/journal.pcbi.1009031 (PMC8189501; doi:10.1371/journal.pcbi.1009031)
Supplement: S6 Table — (PDF) [file pcbi.1009031.s009.pdf]

S6 Table: The AIC score for increased viral clearance, viral neutralization, protection, and improved antigen presentation mechanisms for the baseline effector cell source (BL) model, saturated source (SS) model, and antigen presenting cell source (APCS) model for macaque RLn12

RLn12			
Mechanism	BL Model	SS Model	APCS Model
Viral clearance	54.70	59.82	84.75
Virus neutralization	55.83	73.47	88.44
Protection	43.80	46.71	53.96
Antigen presentation	97.62	92.50	58.03
Antigen presentation with viral clearance	53.43	62.59	41.52
