# Supplementary material for: Mechanistic basis of post-treatment control of SIV after anti-α4β7 antibody therapy
Source: PLoS Comput Biol. 2021 Jun 9;17(6):e1009031. doi: 10.1371/journal.pcbi.1009031 (PMC8189501; doi:10.1371/journal.pcbi.1009031)
Supplement: S8 Table — (PDF) [file pcbi.1009031.s011.pdf]

S8 Table: The AIC score for increased viral clearance, viral neutralization, protection, and improved antigen presentation mechanisms for the baseline effector cell source (BL) model, saturated source (SS) model, and antigen presenting cell source (APCS) model for macaque RSd14

RSd14

Mechanism	BL Model	SS Model	APCS Model
Viral clearance	101.56	118.96	153.40
Virus neutralization	111.10	122.21	141.20
Protection	103.18	118.46	120.90
Antigen presentation	104.90	99.50	113.83
Antigen presentation with viral clearance	105.66	104.95	116.50
