# Supplementary material for: Mechanistic basis of post-treatment control of SIV after anti-α4β7 antibody therapy
Source: PLoS Comput Biol. 2021 Jun 9;17(6):e1009031. doi: 10.1371/journal.pcbi.1009031 (PMC8189501; doi:10.1371/journal.pcbi.1009031)
Supplement: S9 Table — (PDF) [file pcbi.1009031.s012.pdf]

S9 Table: The AIC score for increased viral clearance, viral neutralization, protection, and improved antigen presentation mechanisms for the baseline effector cell source (BL) model, saturated source (SS) model, and antigen presenting cell source (APCS) model for macaque RFa15

RFa15			
Mechanism	BL Model	SS Model	APCS Model
Viral clearance	64.09	84.56	94.55
Virus neutralization	70.11	88.08	93.63
Protection	35.90	51.98	64.51
Antigen presentation	83.18	95.60	67.57
Antigen presentation with viral clearance	65.40	66.51	70.23
