# Supplementary material for: Mechanistic basis of post-treatment control of SIV after anti-α4β7 antibody therapy
Source: PLoS Comput Biol. 2021 Jun 9;17(6):e1009031. doi: 10.1371/journal.pcbi.1009031 (PMC8189501; doi:10.1371/journal.pcbi.1009031)
Supplement: S10 Table — (PDF) [file pcbi.1009031.s013.pdf]

S10 Table: The AIC score for increased viral clearance, viral neutralization, protection, and improved antigen presentation mechanisms for the baseline effector cell source (BL) model, saturated source (SS) model, and antigen presenting cell source (APCS) model for macaque RDa15

RDa15

Mechanism	BL Model	SS Model	APCS Model
Viral clearance	62.93	76.63	90.97
Virus neutralization	62.54	83.72	94.99
Protection	92.87	87.40	87.33
Antigen presentation	82.25	81.79	85.38
Antigen presentation with viral clearance	65.60	79.40	88.04
