# Supplementary material for: Mechanistic basis of post-treatment control of SIV after anti-α4β7 antibody therapy
Source: PLoS Comput Biol. 2021 Jun 9;17(6):e1009031. doi: 10.1371/journal.pcbi.1009031 (PMC8189501; doi:10.1371/journal.pcbi.1009031)
Supplement: S11 Table — (PDF) [file pcbi.1009031.s014.pdf]

S11 Table: The AIC score for increased viral clearance, viral neutralization, protection, and improved antigen presentation mechanisms for the baseline effector cell source (BL) model, saturated source (SS) model, and antigen presenting cell source (APCS) model for macaque ROq14

ROq14

Mechanism	BL Model	SS Model	APCS Model
Viral clearance	105.45	111.67	107.45
Virus neutralization	101.97	104.05	124.69
Protection	111.53	121.59	101.83
Antigen presentation	111.27	117.67	118.92
Antigen presentation with viral clearance	108.11	114.83	110.11
