# Supplementary material for: Mechanistic basis of post-treatment control of SIV after anti-α4β7 antibody therapy
Source: PLoS Comput Biol. 2021 Jun 9;17(6):e1009031. doi: 10.1371/journal.pcbi.1009031 (PMC8189501; doi:10.1371/journal.pcbi.1009031)
Supplement: S12 Table — (PDF) [file pcbi.1009031.s015.pdf]

S12 Table: The AIC score for increased viral clearance, viral neutralization, protection, and improved antigen presentation mechanisms for the baseline effector cell source (BL) model, saturated source (SS) model, and antigen presenting cell source (APCS) model for macaque ROv14

ROv14			
Mechanism	BL Model	SS Model	APCS Model
Viral clearance	119.83	139.03	168.29
Virus neutralization	144.25	148.97	165.06
Protection	120.98	123.32	154.38
Antigen presentation	130.25	122.34	133.68
Antigen presentation with viral clearance	122.47	127.73	136.33
