# Supplementary material for: Mechanistic basis of post-treatment control of SIV after anti-α4β7 antibody therapy
Source: PLoS Comput Biol. 2021 Jun 9;17(6):e1009031. doi: 10.1371/journal.pcbi.1009031 (PMC8189501; doi:10.1371/journal.pcbi.1009031)
Supplement: S13 Table — (PDF) [file pcbi.1009031.s016.pdf]

S13 Table: The AIC weight for the baseline effector cell source (BL) model, saturated source (SS) model, and antigen presenting cell source (APCS) model for the seven IgG control macaques.

Model	RBe14	RIt11	RKs13	RSy13	RYy13	RLo14	RUs14	Average
BL Model	0.057	**0.868**	**0.999**	**0.854**	**0.658**	0.000	0.450	**0.555**
SS Model	0.015	0.011	0.001	0.131	0.336	0.000	0.026	0.074
APCS Model	**0.928**	0.121	0.000	0.015	0.006	**1.000**	**0.524**	0.371
