# Supplementary material for: Mechanistic basis of post-treatment control of SIV after anti-α4β7 antibody therapy
Source: PLoS Comput Biol. 2021 Jun 9;17(6):e1009031. doi: 10.1371/journal.pcbi.1009031 (PMC8189501; doi:10.1371/journal.pcbi.1009031)
Supplement: S15 Table — The intervals specify the range of the set of intervals to approximate the 95% confidence intervals (S1 and S3 Texts). (PDF) [file pcbi.1009031.s018.pdf]

**S15 Table**: The estimate parameters for the eight treated macaques using the viral neutralization mechanism for the three different effector cell source models. The intervals specify the range of the set of intervals to approximate the 95% confidence intervals (S1 and S3 Text).

Baseline source

| Macaque | $r_T$ | $m$ | $p$ | $K_B$ | $K_P$ | $\psi$ | $\gamma$ | $\sigma$ | ln($L$) |
|---|---|---|---|---|---|---|---|---|---|
| ROq14 | 0.22 (0.22–0.23) | 4.04E-07 (3.58E-07–4.06E-07) | 7354 ( 7353– 7396) | 3.68E-01 (3.56E-01–3.74E-01) | N/A | 9.89E-04 (8.82E-04–1.00E-03) | 2.33 (2.25–2.70) | 0.80 (0.78–0.83) | -42.93 |
| RDa15 | 0.17 (0.16–0.18) | 9.65E-07 (8.61E-07–1.54E-06) | 8416 ( 8234– 8899) | 1.33E-01 (1.19E-01–1.49E-01) | N/A | 3.06E-04 (2.75E-04–5.48E-04) | 2.02 (1.00–2.14) | 0.47 (0.44–0.51) | -23.21 |
| RFa15 | 0.33 (0.33–0.34) | 6.85E-06 (6.36E-06–7.03E-06) | 10458 (10419–10544) | 1.56E-01 (1.47E-01–1.62E-01) | N/A | 8.04E-04 (7.52E-04–8.13E-04) | 1.00 (1.00–1.00) | 0.50 (0.49–0.51) | -27.02 |
| RLn12 | 0.19 (0.18–0.19) | 1.37E-06 (1.15E-06–1.37E-06) | 8107 ( 8079– 8146) | 1.31E-01 (1.30E-01–1.35E-01) | N/A | 6.11E-04 (5.99E-04–6.30E-04) | 3.22 (1.78–3.28) | 0.42 (0.42–0.44) | -19.84 |
| RId14 | 0.33 (0.19–0.37) | 8.63E-08 (5.62E-08–1.06E-07) | 6635 ( 6403– 6844) | 6.88E-01 (2.74E-01–6.88E-01) | N/A | 9.79E-04 (6.72E-04–1.00E-03) | 1.00 (1.00–1.00) | 1.18 (1.14–1.61) | -63.35 |
| ROo13 | 0.28 (0.27–0.30) | 8.15E-08 (5.89E-08–8.48E-08) | 7105 ( 6904– 7198) | 6.42E-01 (4.22E-01–6.43E-01) | N/A | 9.88E-04 (8.93E-04–1.00E-03) | 1.00 (1.00–1.00) | 1.05 (1.01–1.25) | -57.36 |
| RSd14 | 0.25 (0.24–0.26) | 4.15E-07 (2.00E-07–4.70E-07) | 7230 ( 6935– 7338) | 2.79E-01 (1.91E-01–3.04E-01) | N/A | 6.08E-05 (4.98E-05–1.65E-04) | 1.00 (1.00–1.00) | 0.83 (0.74–1.05) | -47.47 |
| ROv14 | 0.25 (0.23–0.27) | 3.51E-07 (3.45E-07–1.85E-06) | 6968 ( 6962– 9881) | 2.32E-01 (1.19E-01–2.58E-01) | N/A | 9.00E-06 (8.30E-06–1.00E-03) | 1.00 (1.00–1.00) | 1.13 (1.09–1.45) | -64.07 |

Saturated source

| Macaque | $r_T$ | $m$ | $p$ | $K_B$ | $K_P$ | $\psi$ | $\gamma$ | $\sigma$ | ln($L$) |
|---|---|---|---|---|---|---|---|---|---|
| ROq14 | 0.20 (0.20–0.20) | 8.11E-07 (7.90E-07–8.29E-07) | 8396 ( 8371– 8423) | 2.81E-01 (2.57E-01–2.81E-01) | 1.07E-04 (1.02E-04–1.15E-04) | 9.82E-04 (9.39E-04–1.00E-03) | 1.00 (1.00–1.00) | 0.78 (0.75–0.79) | -42.64 |
| RDa15 | 0.20 (0.18–0.21) | 1.70E-06 (1.45E-06–2.45E-06) | 7449 ( 7299– 7587) | 2.62E-01 (1.72E-01–2.70E-01) | 8.61E-02 (6.03E-02–1.00E-01) | 5.64E-04 (4.45E-04–9.33E-04) | 1.00 (1.00–1.00) | 0.58 (0.52–0.72) | -32.48 |
| RFa15 | 0.15 (0.15–0.16) | 2.16E-07 (1.53E-07–3.39E-07) | 7031 ( 6768– 7072) | 2.42E-01 (2.05E-01–2.44E-01) | 1.40E-05 (1.34E-05–1.73E-05) | 9.98E-04 (9.39E-04–1.00E-03) | 4.99 (2.09–5.00) | 0.61 (0.54–0.68) | -34.66 |
| RLn12 | 0.17 (0.16–0.18) | 3.30E-06 (2.13E-06–3.34E-06) | 7927 ( 7695– 8016) | 7.84E-02 (4.42E-02–8.72E-02) | 2.39E-02 (1.08E-02–2.84E-02) | 9.83E-04 (7.44E-04–1.00E-03) | 1.96 (1.00–2.05) | 0.49 (0.48–0.71) | -27.32 |
| RId14 | 0.25 (0.24–0.29) | 3.02E-07 (1.29E-07–3.51E-07) | 8712 ( 8463– 8934) | 4.71E-01 (3.47E-01–5.01E-01) | 1.32E-05 (1.22E-05–4.84E-04) | 6.39E-04 (5.27E-04–1.00E-03) | 1.00 (1.00–1.00) | 1.18 (1.07–1.72) | -64.85 |
| ROo13 | 0.28 (0.26–0.28) | 8.18E-08 (5.72E-08–8.63E-08) | 7110 ( 6728– 7265) | 6.41E-01 (4.36E-01–6.42E-01) | 1.05E-05 (1.00E-05–1.57E-05) | 9.98E-04 (7.10E-04–1.00E-03) | 1.00 (1.00–1.00) | 1.06 (0.90–1.50) | -57.58 |
| RSd14 | 0.31 (0.27–0.53) | 2.22E-07 (5.77E-08–2.63E-07) | 6857 ( 6427– 7194) | 5.05E-01 (1.98E-01–5.63E-01) | 2.01E-05 (1.00E-05–2.23E-05) | 1.25E-04 (7.97E-05–5.00E-04) | 1.00 (1.00–1.00) | 0.88 (0.79–1.20) | -51.69 |
| ROv14 | 0.39 (0.27–0.39) | 5.93E-07 (5.93E-07–1.07E-05) | 8117 ( 8095–11540) | 2.27E-01 (9.74E-02–2.79E-01) | 1.21E-05 (1.15E-05–7.80E-02) | 7.21E-04 (6.64E-04–1.00E-03) | 1.00 (1.00–1.14) | 1.14 (1.14–1.64) | -65.12 |

APC source

| Macaque | $r_T$ | $m$ | $p$ | $K_B$ | $K_P$ | $\psi$ | $\gamma$ | $\sigma$ | ln($L$) |
|---|---|---|---|---|---|---|---|---|---|
| ROq14 | 0.51 (0.49–0.53) | 2.35E-07 (1.97E-07–3.34E-07) | 6848 ( 6757– 7128) | 3.54E-01 (2.94E-01–4.40E-01) | N/A | 9.99E-04 (9.70E-04–1.00E-03) | 1.00 (1.00–1.00) | 0.94 (0.88–1.03) | -54.31 |
| RDa15 | 0.31 (0.29–0.31) | 1.57E-07 (1.49E-07–1.91E-07) | 7117 ( 7032– 7185) | 5.35E-01 (4.55E-01–5.60E-01) | N/A | 9.99E-04 (8.71E-04–1.00E-03) | 1.00 (1.00–1.00) | 0.66 (0.64–0.80) | -39.44 |
| RFa15 | 0.51 (0.50–0.54) | 1.59E-07 (1.35E-07–3.06E-07) | 4607 ( 4530– 4657) | 2.83E-01 (2.32E-01–3.16E-01) | N/A | 1.00E-03 (9.77E-04–1.00E-03) | 5.00 (2.74–5.00) | 0.68 (0.61–0.71) | -38.76 |
| RLn12 | 0.52 (0.45–0.55) | 5.08E-08 (2.95E-08–7.18E-08) | 4243 ( 4000– 4417) | 4.45E-01 (2.84E-01–6.53E-01) | N/A | 1.00E-03 (8.77E-04–1.00E-03) | 5.00 (4.62–5.00) | 0.60 (0.55–0.87) | -36.14 |
| RId14 | 0.37 (0.37–0.37) | 6.53E-08 (6.53E-08–6.53E-08) | 6870 ( 6832– 6870) | 4.54E-01 (4.54E-01–4.54E-01) | N/A | 9.97E-04 (9.97E-04–9.97E-04) | 4.63 (4.63–4.63) | 1.31 (1.27–1.31) | -72.87 |
| ROo13 | 0.41 (0.41–0.42) | 8.02E-08 (8.02E-08–8.13E-08) | 6314 ( 6311– 6319) | 3.79E-01 (3.75E-01–3.79E-01) | N/A | 9.94E-04 (9.94E-04–1.00E-03) | 4.86 (4.76–5.00) | 1.27 (1.27–1.28) | -67.63 |
| RSd14 | 0.41 (0.41–0.42) | 8.02E-08 (7.89E-08–8.28E-08) | 6314 ( 6314– 6323) | 3.79E-01 (3.79E-01–3.98E-01) | N/A | 9.94E-04 (9.85E-04–1.00E-03) | 4.86 (4.69–5.00) | 1.15 (1.15–1.16) | -62.52 |
| ROv14 | 0.52 (0.48–0.53) | 2.79E-08 (2.59E-08–5.67E-08) | 5041 ( 5039– 5065) | 8.32E-01 (5.96E-01–8.32E-01) | N/A | 7.60E-04 (7.60E-04–1.00E-03) | 4.43 (4.43–5.00) | 1.47 (1.27–1.49) | -74.47 |
