# Supplementary material for: Mechanistic basis of post-treatment control of SIV after anti-α4β7 antibody therapy
Source: PLoS Comput Biol. 2021 Jun 9;17(6):e1009031. doi: 10.1371/journal.pcbi.1009031 (PMC8189501; doi:10.1371/journal.pcbi.1009031)
Supplement: S16 Table — The intervals specify the range of the set of intervals to approximate the 95% confidence intervals (S1 and S3 Texts). (PDF) [file pcbi.1009031.s019.pdf]

S16 Table: The estimate parameters for the eight treated macaques using the protection mechanism for the three different effector cell source models. The intervals specify the range of the set of intervals to approximate the 95% confidence intervals (S1 and S3 Text).

Baseline source

Macaque	r_T	m	p	K_B	K_P	ω	ρ	σ	$\ln(L)$
	0.22	1.25E-05	11839	1.13E-01		18.01	6.67E-02	0.92	
ROq14	(0.22–0.23)	(1.05E-05–1.28E-05)	(11746–12061)	(1.03E-01–1.23E-01)	N/A	(17.18–18.78)	(4.64E-02–2.35E-01)	(0.88–0.97)	-47.71
	0.35	4.37E-06	9688	2.26E-01		68.33	7.16E-02	0.67	
RDa15	(0.30–0.36)	(3.68E-06–8.13E-06)	(9605–12194)	(1.58E-01–2.63E-01)	N/A	(2.18–71.80)	(2.90E-02–1.39E-01)	(0.54–0.71)	-38.40
	0.17	1.36E-06	8304	2.03E-01		0.89	2.11E-02	0.31	
RFa15	(0.17–0.17)	(1.17E-06–1.42E-06)	(8145– 8375)	(1.70E-01–2.04E-01)	N/A	(0.84–0.93)	(1.47E-02–3.44E-02)	(0.29–0.36)	-9.90
	0.18	1.08E-06	7863	1.29E-01		0.63	2.19E-02	0.37	
RLn12	(0.18–0.19)	(9.04E-07–1.12E-06)	(7832– 7984)	(1.22E-01–1.38E-01)	N/A	(0.60–0.65)	(1.49E-02–2.83E-02)	(0.37–0.38)	-13.82
	0.25	1.23E-07	8159	5.59E-01		0.46	8.29E-03	1.21	
RId14	(0.25–0.26)	(1.20E-07–1.31E-07)	(8094– 8221)	(4.57E-01–5.59E-01)	N/A	(0.44–0.48)	(7.17E-03–9.93E-03)	(1.19–1.35)	-64.09
	0.26	2.06E-06	10030	2.24E-01		1.55	3.18E-03	1.09	
ROo13	(0.26–0.26)	(1.99E-06–2.06E-06)	(10016–10031)	(2.19E-01–2.29E-01)	N/A	(1.54–1.56)	(3.18E-03–3.23E-03)	(1.09–1.10)	-60.80
	0.21	1.30E-06	8573	2.06E-01		4.22	1.24E-03	0.77	
RSd14	(0.21–0.21)	(8.91E-07–1.33E-06)	(8530– 8667)	(1.99E-01–2.19E-01)	N/A	(4.16–4.32)	(1.17E-03–2.28E-03)	(0.75–0.82)	-43.51
	0.26	1.49E-07	8501	2.25E-01		0.44	1.05E-03	0.91	
ROv14	(0.26–0.26)	(1.38E-07–1.60E-07)	(8470– 8553)	(2.15E-01–2.34E-01)	N/A	(0.44–0.45)	(8.27E-04–1.17E-03)	(0.87–0.93)	-52.43

Saturated source

Macaque	r_T	m	p	K_B	K_P	ω	ρ	σ	$\ln(L)$
	0.28	1.11E-06	10021	4.04E-01	1.00E-05	11.90	3.00E-02	0.91	
ROq14	(0.25–0.28)	(1.08E-06–9.05E-06)	(9969–10123)	(9.23E-02–4.04E-01)	(1.00E-05–1.71E-03)	(11.79–67.27)	(2.63E-02–1.42E-01)	(0.90–1.38)	-51.41
	0.23	2.33E-06	7714	2.85E-01	9.99E-02	0.64	1.06E-02	0.62	
RDa15	(0.22–0.23)	(2.03E-06–2.36E-06)	(7629– 7737)	(2.57E-01–2.87E-01)	(9.96E-02–1.00E-01)	(0.62–0.65)	(8.06E-03–1.40E-02)	(0.61–0.64)	-34.32
	0.15	2.33E-06	7780	1.56E-01	2.72E-02	4.00	1.96E-02	0.37	
RFa15	(0.15–0.16)	(2.13E-06–2.48E-06)	(7763– 7880)	(1.13E-01–1.59E-01)	(2.63E-02–3.02E-02)	(3.88–4.18)	(1.29E-02–3.07E-02)	(0.37–0.39)	-16.60
	0.19	1.85E-06	7706	1.42E-01	1.27E-02	0.69	2.07E-02	0.38	
RLn12	(0.19–0.19)	(1.49E-06–1.89E-06)	(7696– 7920)	(1.34E-01–1.51E-01)	(1.22E-02–1.36E-02)	(0.65–0.73)	(1.38E-02–2.91E-02)	(0.35–0.39)	-13.95
	0.25	1.23E-07	8159	5.59E-01	1.00E-05	0.46	8.31E-03	1.21	
RId14	(0.25–0.26)	(1.11E-07–1.56E-07)	(8082– 8221)	(5.01E-01–5.59E-01)	(1.00E-05–1.05E-05)	(0.45–0.48)	(6.22E-03–1.02E-02)	(1.18–1.25)	-64.23
	0.30	7.56E-07	7836	4.73E-01	9.52E-05	1.15	7.03E-04	0.95	
ROo13	(0.30–0.30)	(6.98E-07–7.67E-07)	(7800– 7863)	(4.57E-01–4.75E-01)	(9.44E-05–9.75E-05)	(1.13–1.17)	(7.03E-04–9.91E-04)	(0.93–0.98)	-54.32
	0.23	9.55E-07	8008	3.46E-01	2.58E-04	13.26	9.18E-04	0.82	
RSd14	(0.23–0.23)	(7.76E-07–9.58E-07)	(7993– 8043)	(3.08E-01–3.46E-01)	(2.55E-04–2.85E-04)	(12.94–13.51)	(8.64E-04–1.59E-03)	(0.81–0.85)	-49.82
	0.26	2.96E-07	8459	2.38E-01	4.65E-02	0.44	1.00E-03	0.90	
ROv14	(0.26–0.26)	(2.65E-07–3.16E-07)	(8391– 8477)	(2.31E-01–2.44E-01)	(4.48E-02–4.96E-02)	(0.44–0.46)	(7.95E-04–1.12E-03)	(0.88–0.95)	-52.27

APC source

Macaque	r_T	m	p	K_B	K_P	ω	ρ	σ	$\ln(L)$
	0.45	1.33E-07	5024	8.42E-01		2.44	1.19E-02	0.74	
ROq14	(0.45–0.46)	(1.25E-07–1.64E-07)	(4963– 5057)	(7.96E-01–8.42E-01)	N/A	(2.37–2.51)	(8.67E-03–1.52E-02)	(0.73–0.76)	-42.86
	0.51	9.25E-08	5251	8.02E-01		0.45	1.27E-02	0.61	
RDa15	(0.51–0.52)	(9.06E-08–1.00E-07)	(5188– 5279)	(7.61E-01–8.02E-01)	N/A	(0.45–0.47)	(1.07E-02–1.74E-02)	(0.60–0.63)	-35.61
	0.55	9.99E-08	5454	3.26E-01		0.86	5.90E-02	0.44	
RFa15	(0.35–0.55)	(6.82E-08–1.62E-07)	(5004– 5852)	(2.38E-01–7.84E-01)	N/A	(0.52–7.19)	(2.81E-02–4.75E+00)	(0.42–0.62)	-24.20
	0.55	5.32E-08	5016	2.87E-01		5.91	7.32E-02	0.38	
RLn12	(0.39–0.55)	(3.74E-08–8.49E-08)	(4845– 5260)	(1.75E-01–5.01E-01)	N/A	(4.09–8.31)	(3.84E-02–4.75E+00)	(0.34–0.64)	-18.90
	0.35	3.89E-08	5934	7.00E-01		78.00	1.17E-01	1.44	
RId14	(0.09–0.53)	(2.95E-08–9.85E-08)	(5563– 8287)	(2.86E-01–7.08E-01)	N/A	(0.44–78.00)	(3.46E-02–4.75E+00)	(1.30–2.00)	-72.05
	0.51	2.97E-08	5463	7.68E-01		0.50	2.11E-02	1.07	
ROo13	(0.50–0.52)	(2.36E-08–4.65E-08)	(5413– 5495)	(4.26E-01–7.69E-01)	N/A	(0.47–0.52)	(2.09E-02–3.65E-02)	(1.05–1.50)	-58.39
	0.49	3.82E-08	5542	7.57E-01		0.45	2.03E-02	0.98	
RSd14	(0.46–0.49)	(2.98E-08–5.05E-08)	(5409– 5671)	(4.57E-01–7.57E-01)	N/A	(0.44–0.86)	(2.03E-02–3.61E-02)	(0.94–1.24)	-52.37
	0.35	3.90E-08	6605	6.35E-01		0.52	3.04E-02	1.23	
ROv14	(0.34–0.55)	(3.31E-08–7.62E-08)	(6517– 7412)	(2.24E-01–6.35E-01)	N/A	(0.48–0.73)	(4.55E-03–4.75E+00)	(1.13–1.49)	-69.15
