# Supplementary figures and images for: Mechanistic basis of post-treatment control of SIV after anti-α4β7 antibody therapy
Source: PLoS Comput Biol. 2021 Jun 9;17(6):e1009031. doi: 10.1371/journal.pcbi.1009031 (PMC8189501; doi:10.1371/journal.pcbi.1009031)

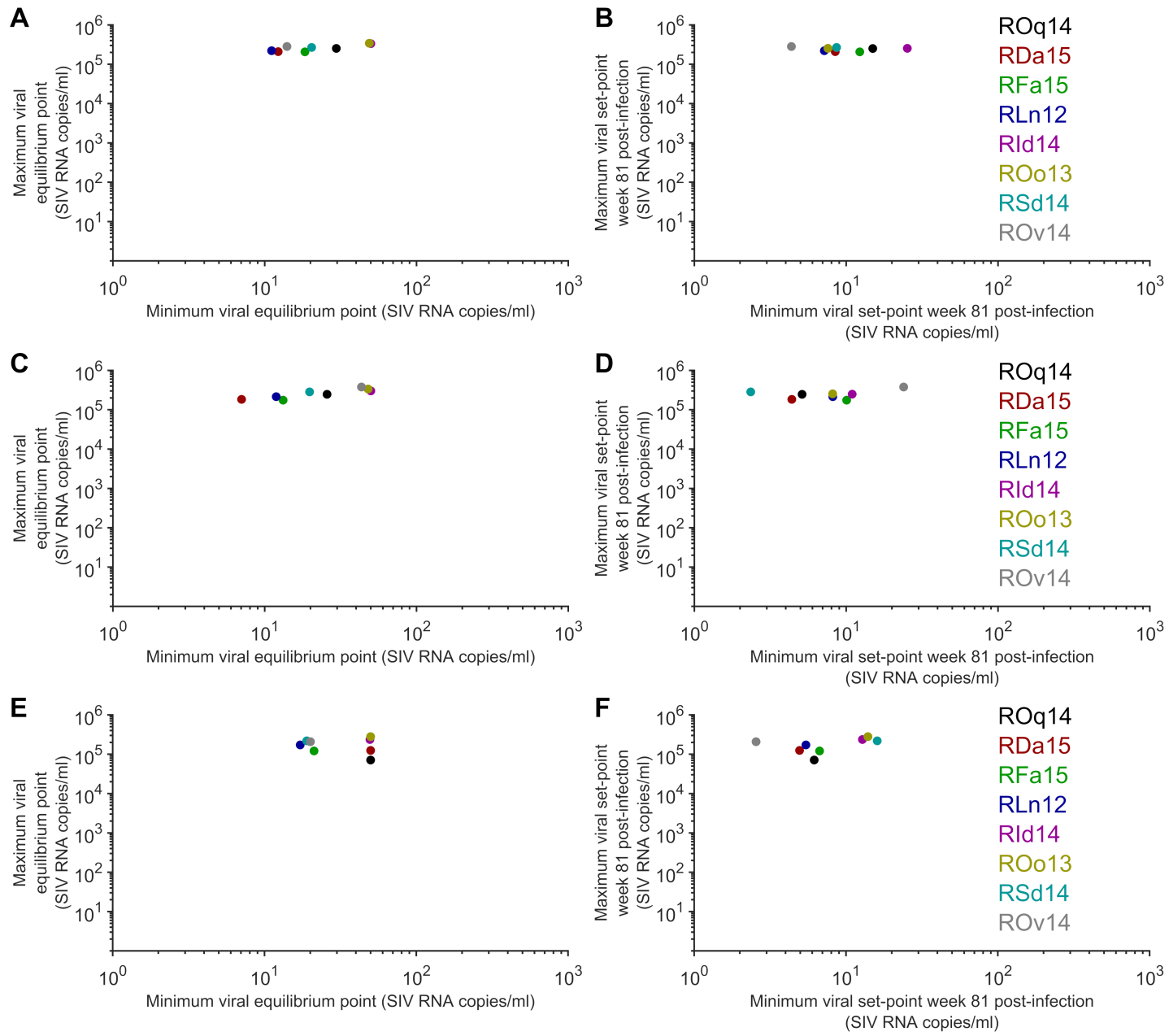

Supplement: S1 Fig — The viral load set points predicted by the model using the best-fit parameter estimates and the mechanism with the greatest AIC weight from A) and B) with the baseline cell source model; C) and D) the model with a saturated source of effector cells; E) and F) a model where the source of effector cells is dependent on the concentration of antigen presenting cells. The maximum and minimum viral load equilibrium points predicted by the model (left column) and estimated by the predicted viral load at 81 weeks p.i. (right column). Parameters for these simulations are in Tables 1 and S14–S18, for the AIC selected mechanism (S5–S12 Tables). We do not consider the alternative mechanism of increased antigen presentation with increased viral clearance in this analysis. (PDF) [file pcbi.1009031.s023.pdf]

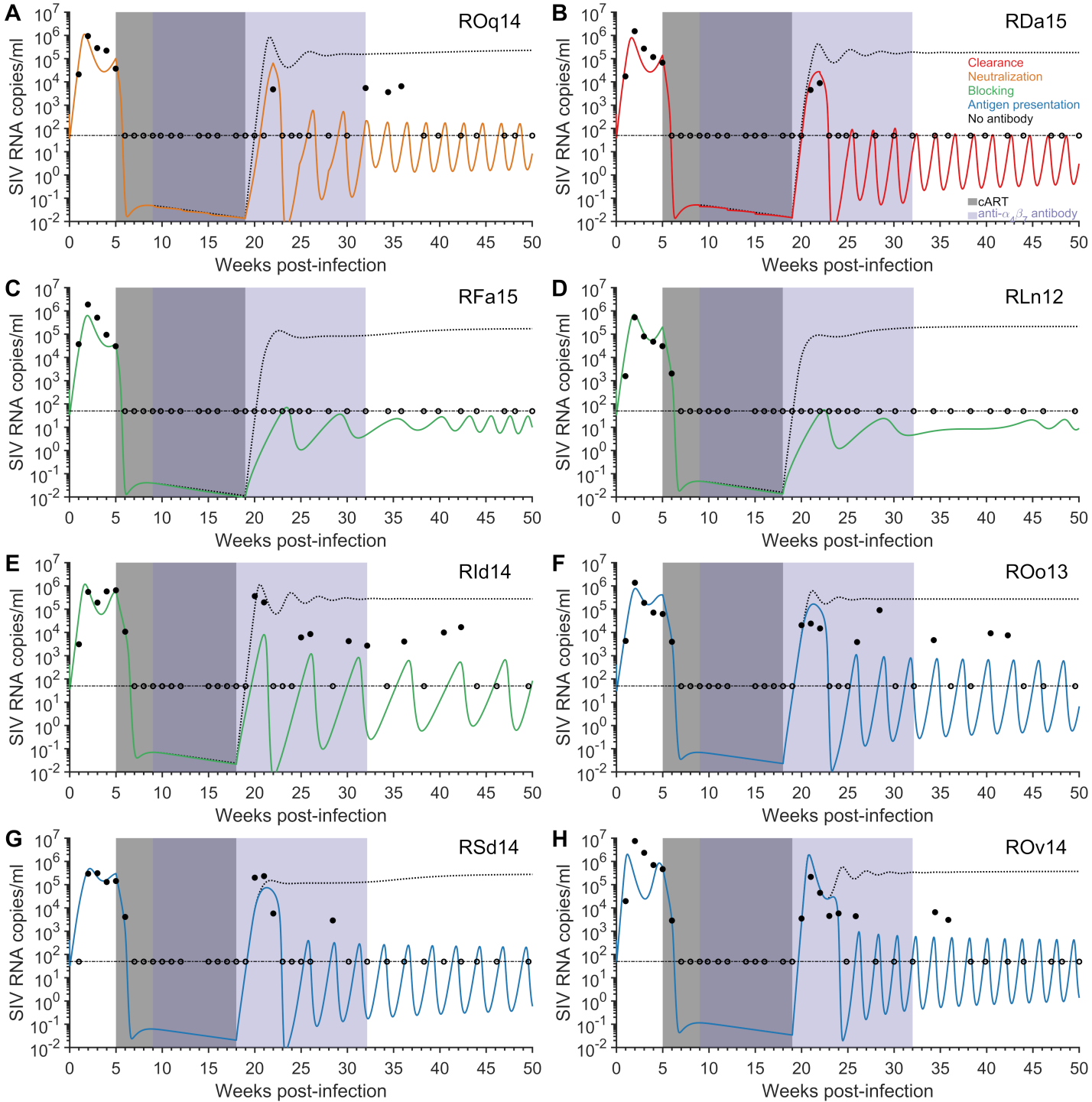

Supplement: S2 Fig — The measured (≥50 SIV RNA copies/ml solid circles and <50 SIV RNA copies/ml open circles) and model predicted viral loads (solid line) using the best-fit parameter estimates and model variation with the greatest AIC weight for each of the treated macaques and predicted viral dynamics in the absence of the anti-α4β7antibody (dotted black line), panels A)–H). The limit of detection is 50 SIV RNA copies/ml (thin horizontal dashed black line). Treatment with cART occurred between five weeks and 18/19 weeks post-infection (gray area), while eight infusions of the anti-α4β7antibody occurred between nine weeks post-infection and 32 weeks post-infection (purple area). The mechanisms considered include increased viral clearance (red line), viral neutralization (orange line), target cell protection (green line), and increased antigen presentation (without increased viral clearance) (blue line). Parameters for these simulations are in Tables 1 and S14–S18 and S2 Text, for the AIC selected mechanism (S5–S12 Tables). (PDF) [file pcbi.1009031.s024.pdf]

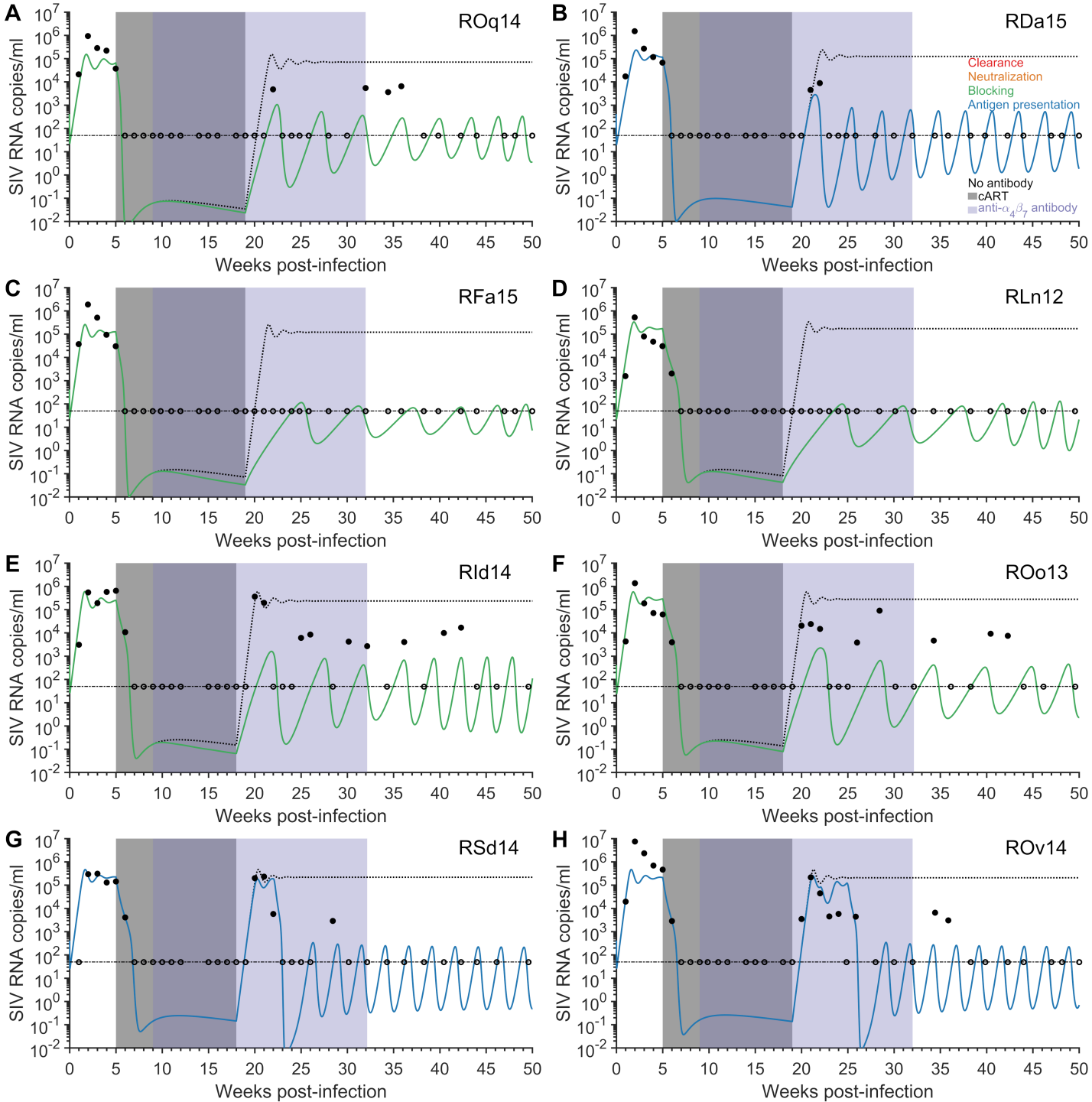

Supplement: S3 Fig — The measured (≥50 SIV RNA copies/ml solid circles and <50 SIV RNA copies/ml open circles) and model predicted viral loads (solid line) using the best-fit parameter estimates and model variation with the greatest AIC weight for each of the treated macaques and predicted viral dynamics in the absence of the anti-α4β7antibody (dotted black line), panels A)–H). The limit of detection is 50 SIV RNA copies/ml (thin horizontal dashed black line). Treatment with cART occurred between five weeks and 18/19 weeks post-infection (gray area), while eight infusions of the anti-α4β7antibody occurred between nine weeks post-infection and 32 weeks post-infection (purple area). The mechanisms considered include increased viral clearance (red line), viral neutralization (orange line), target cell protection (green line), and increased antigen presentation (without increased viral clearance) (blue line). Parameters for these simulations are in Tables 1and S14–S18 and S2 Text, for the AIC selected mechanism (S5–S12 Tables). (PDF) [file pcbi.1009031.s025.pdf]

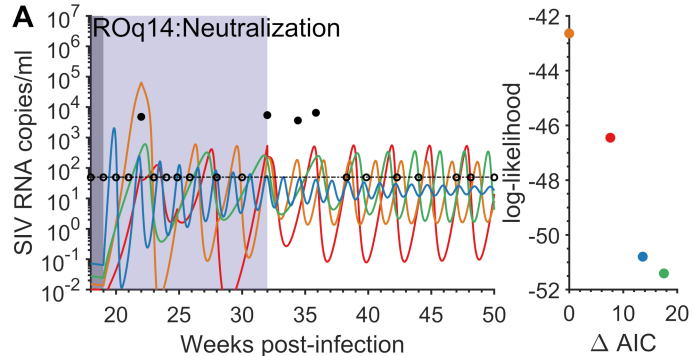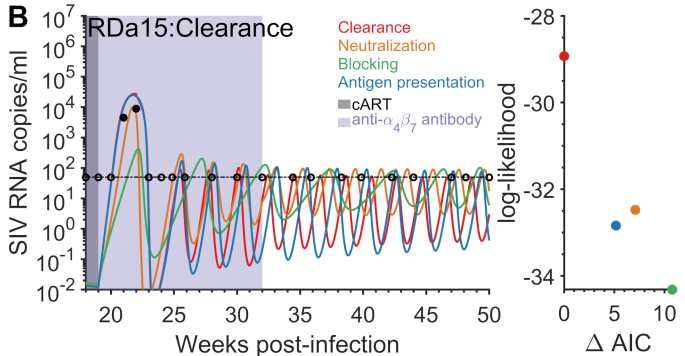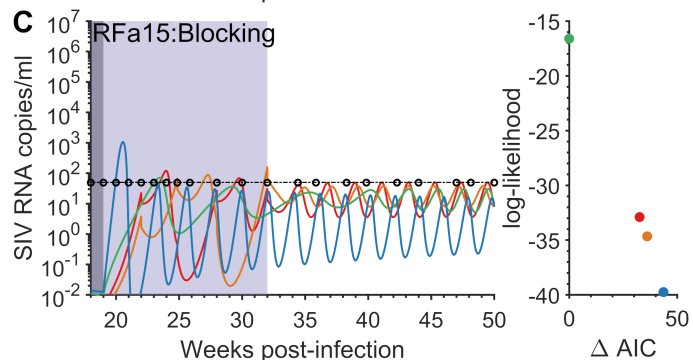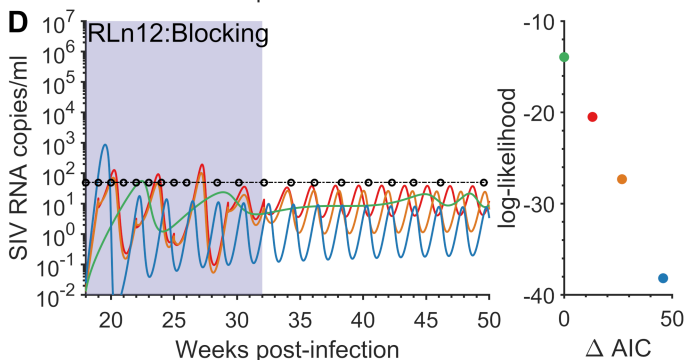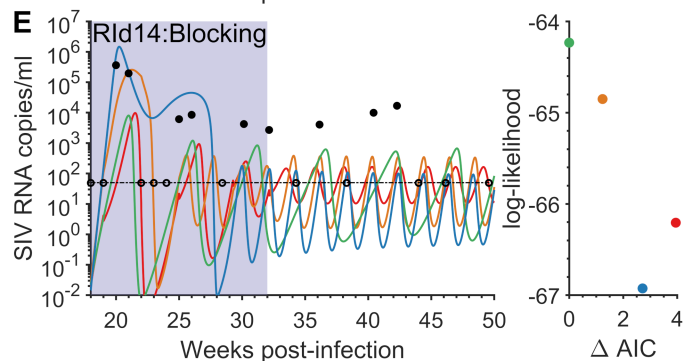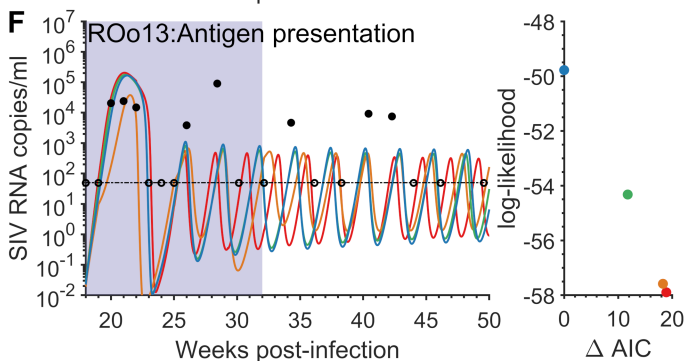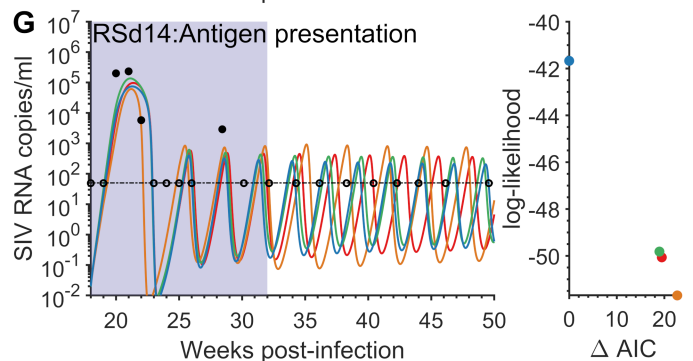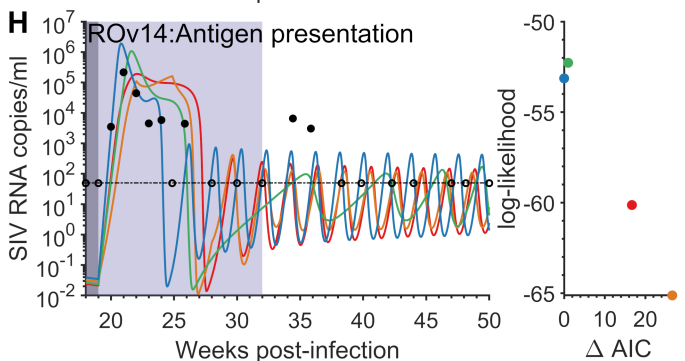

Supplement: S4 Fig — The measured (≥50 SIV RNA copies/ml solid circles and <50 SIV RNA copies/ml open circles) and model predicted viral loads for the AIC selected model (indicated after macaque) and three remaining models using the best-fit parameter estimates for the mechanisms of increased viral clearance (red line), viral neutralization (orange line), target cell protection (green line), and increased antigen presentation (without increased viral clearance) (blue line), panels A)–H). For each macaque, a scatter plot of the ΔAIC and the log-likelihood for each mechanism, panels A)–H). The limit of detection is 50 SIV RNA copies/ml (thin horizontal dashed black line). Treatment with cART occurred between five weeks and 18/19 weeks post-infection (gray area), while eight infusions of the anti-α4β7antibody occurred between nine weeks post-infection and 32 weeks post-infection (purple area). The mechanisms considered include parameters for these simulations are in Tables 1 and S14–S18 and S2 Text. (PDF) [file pcbi.1009031.s026.pdf]

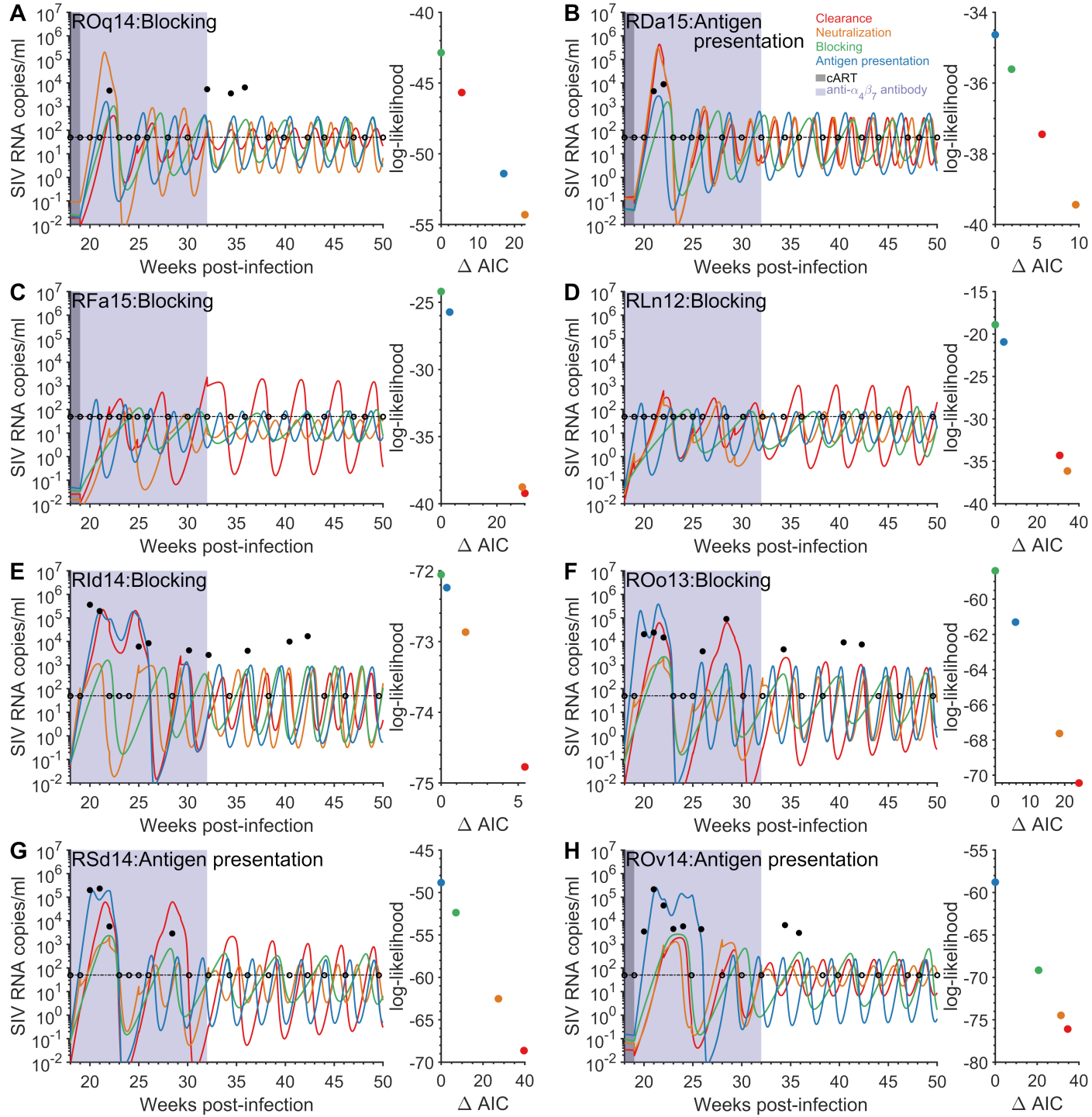

Supplement: S5 Fig — The measured (≥50 SIV RNA copies/ml solid circles and <50 SIV RNA copies/ml open circles) and model predicted viral loads for the AIC selected model (indicated after macaque) and three remaining models using the best-fit parameter estimates for the mechanisms of increased viral clearance (red line), viral neutralization (orange line), target cell protection (green line), and increased antigen presentation (without increased viral clearance) (blue line), panels A)–H). For each macaque, a scatter plot of the ΔAIC and the log-likelihood for each mechanism, panels A)–H). The limit of detection is 50 SIV RNA copies/ml (thin horizontal dashed black line). Treatment with cART occurred between five weeks and 18/19 weeks post-infection (gray area), while eight infusions of the anti-α4β7antibody occurred between nine weeks post-infection and 32 weeks post-infection (purple area). The mechanisms considered include Parameters for these simulations are in Tables 1and S14–S18 and S2 Text. (PDF) [file pcbi.1009031.s027.pdf]

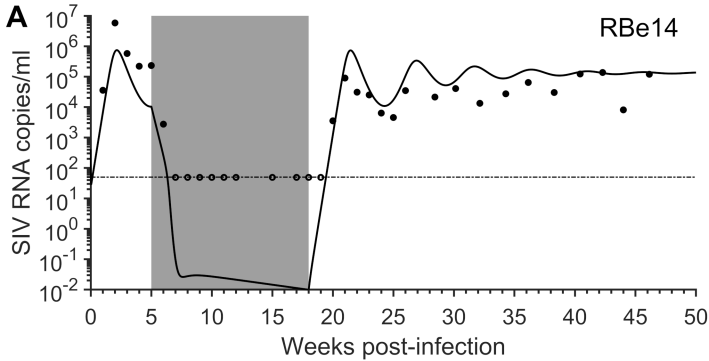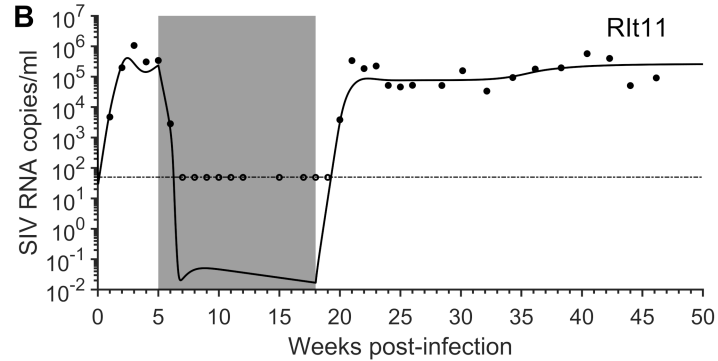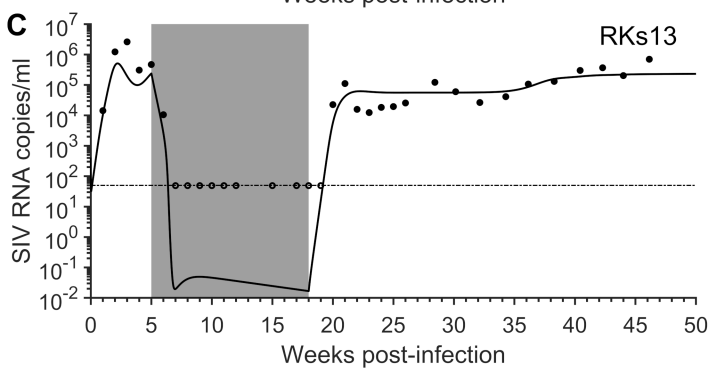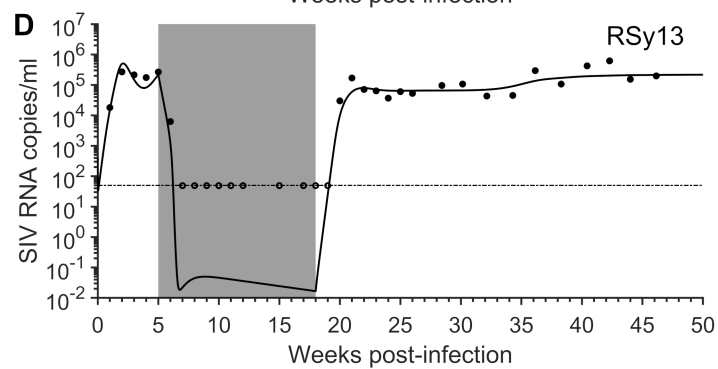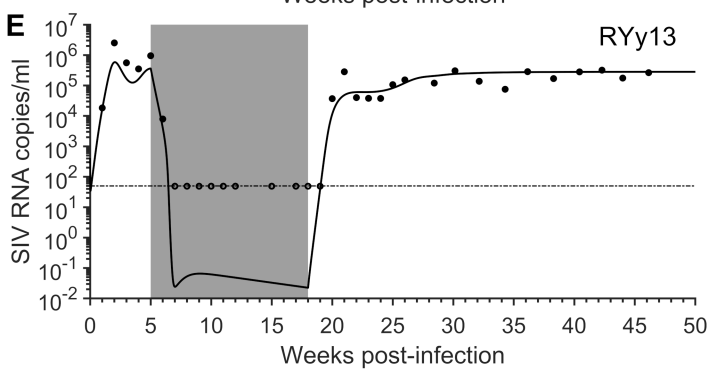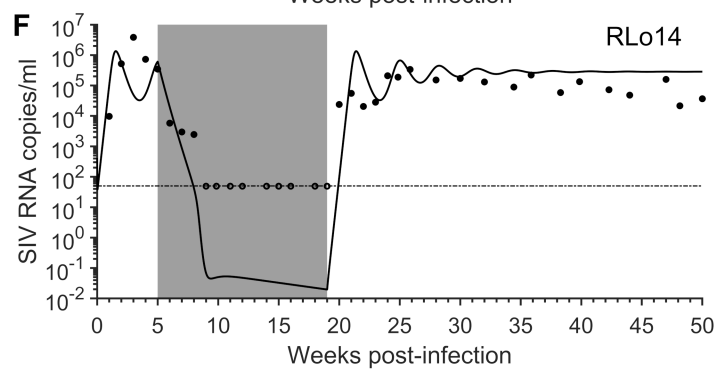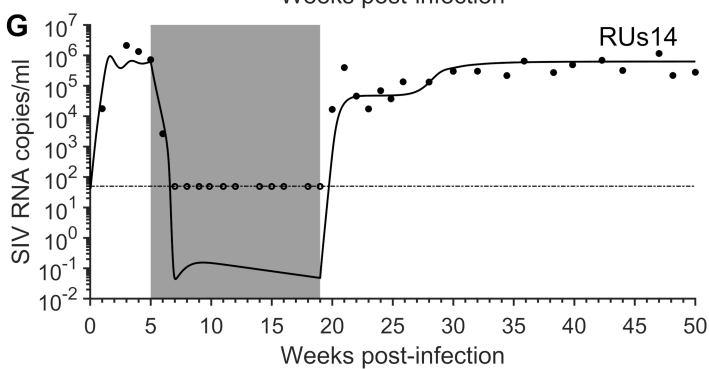

Supplement: S6 Fig — The observed (black dots) and model predicted viral load dynamics (solid line) using the best-fit parameter estimates for A)–G) each of the macaques. Parameters for these simulations are in Tables 1 and S19. (PDF) [file pcbi.1009031.s028.pdf]

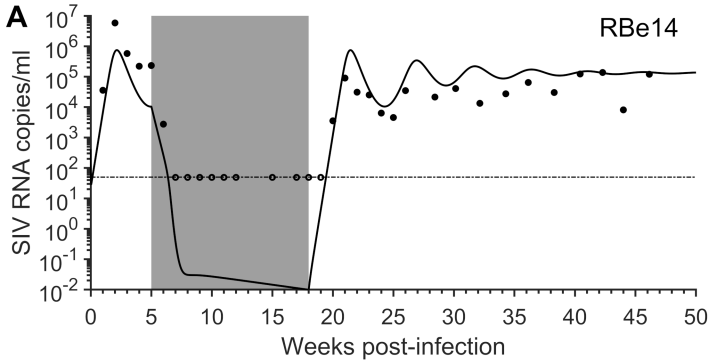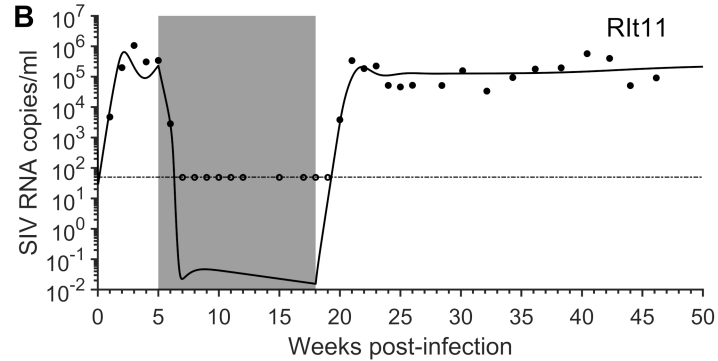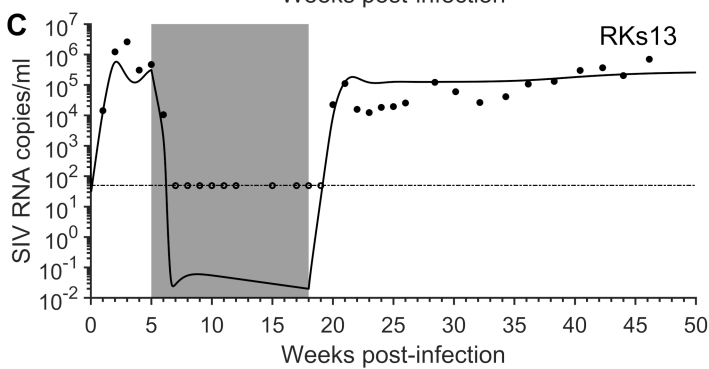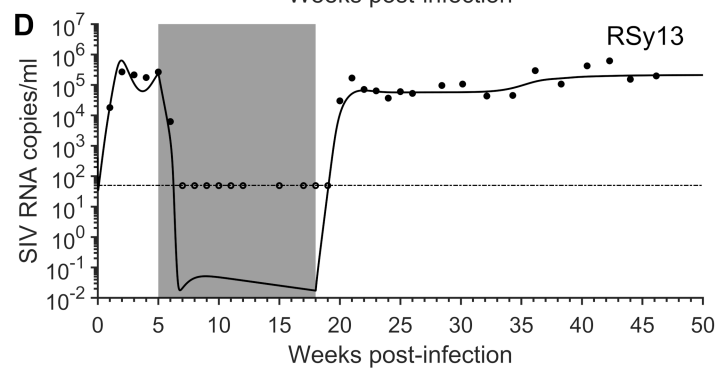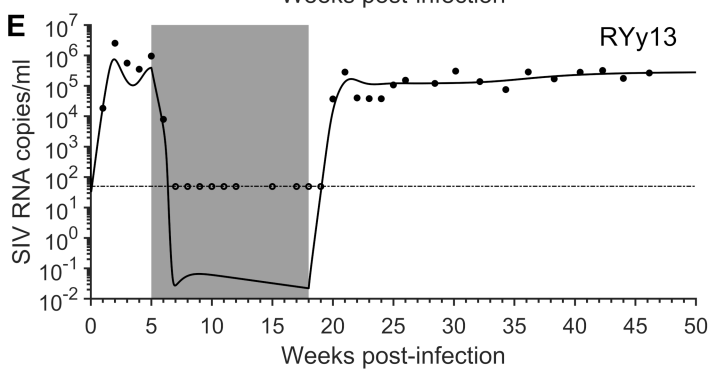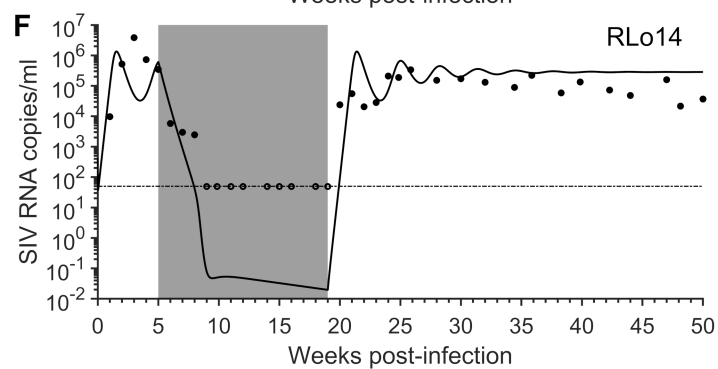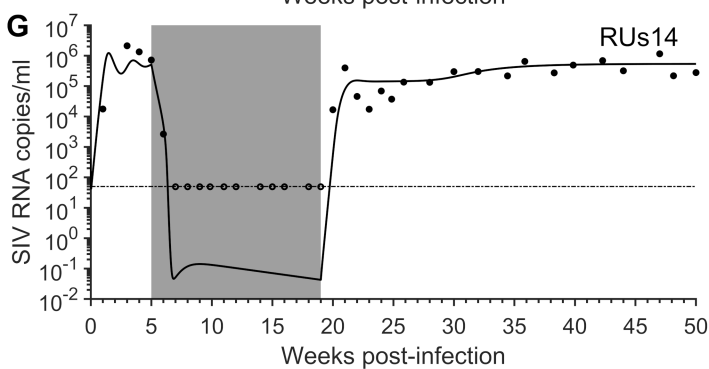

Supplement: S7 Fig — The observed (black dots) and model predicted viral load dynamics (solid line) using the best-fit parameter estimates for A)–G) each of the macaques. Parameters for these simulations are in Tables 1and S19 and S2 Text. (PDF) [file pcbi.1009031.s029.pdf]

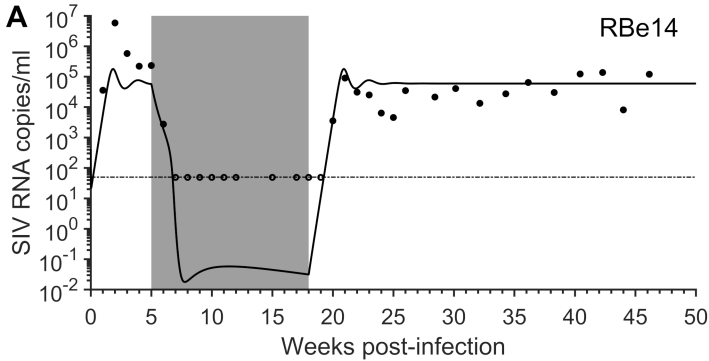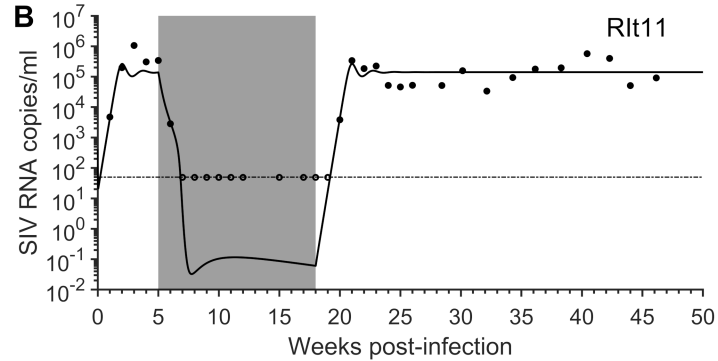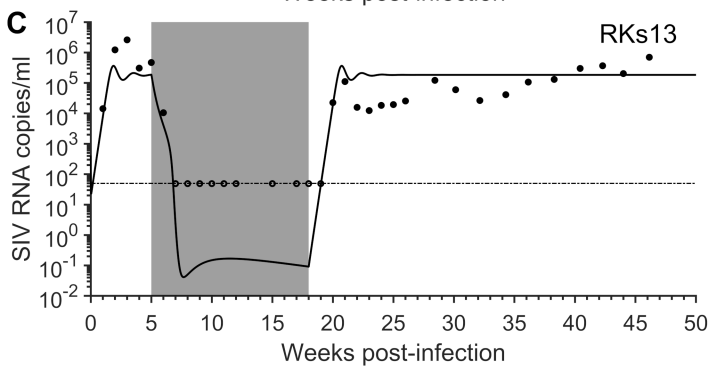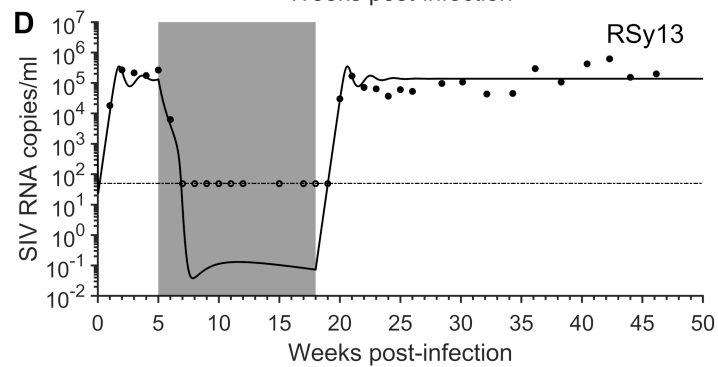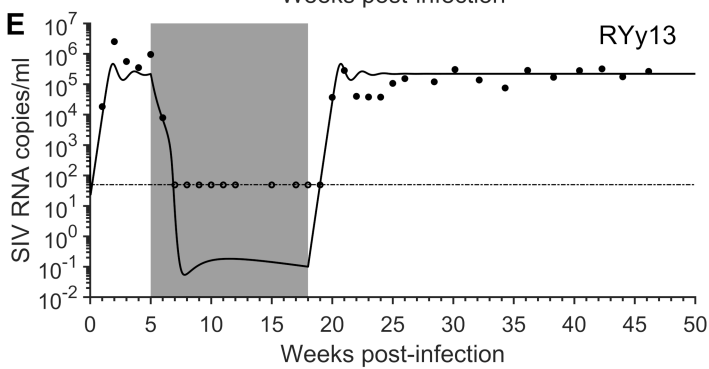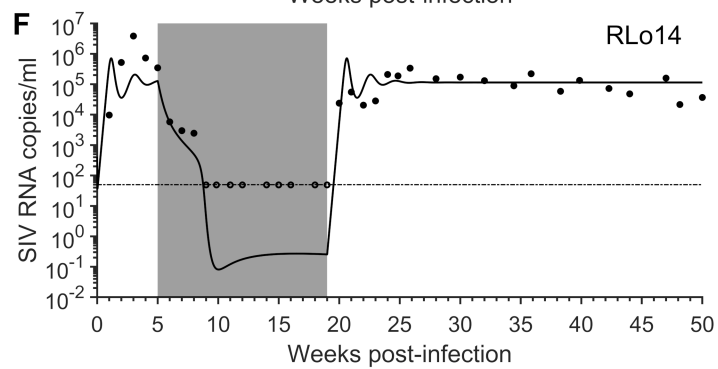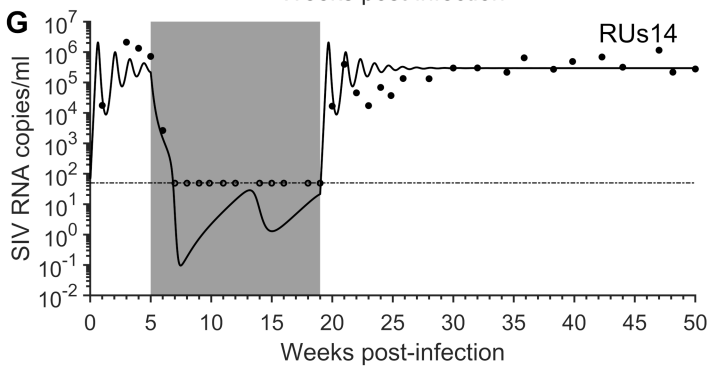

Supplement: S8 Fig — The observed (black dots) and model predicted viral load dynamics (solid line) using the best-fit estimates for A)–G) each of the macaques. Parameters for these simulations are in Tables 1 and S19. (PDF) [file pcbi.1009031.s030.pdf]

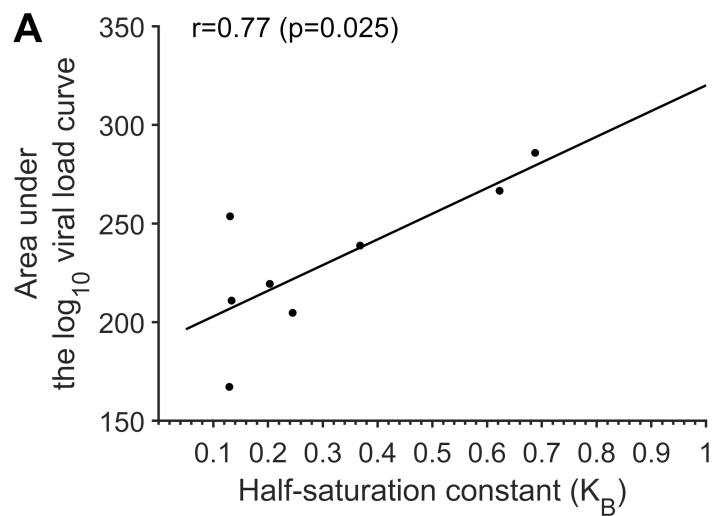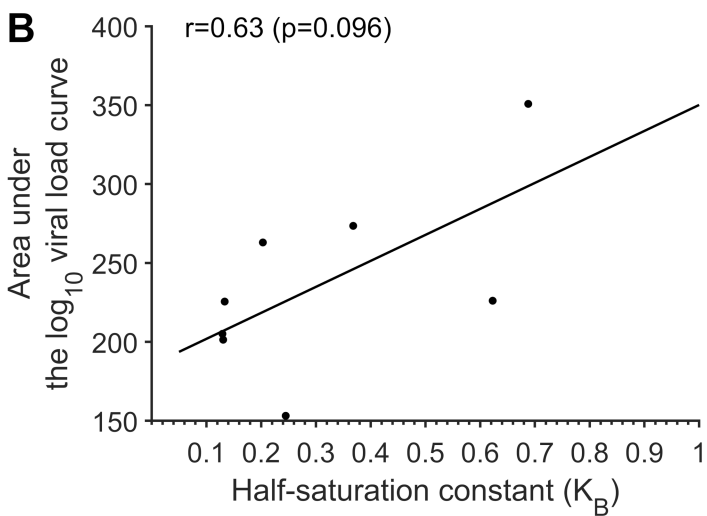

Supplement: S9 Fig — The correlation between the best parameter estimates for the half-saturation constant for effector cell proliferation for the AIC selected mechanism of the baseline model for each treated macaque and the area under the predicted log10 viral load curve for the 30 weeks following A) the removal of cART and B) the last infusion of the anti-α4β7antibody. (PDF) [file pcbi.1009031.s031.pdf]

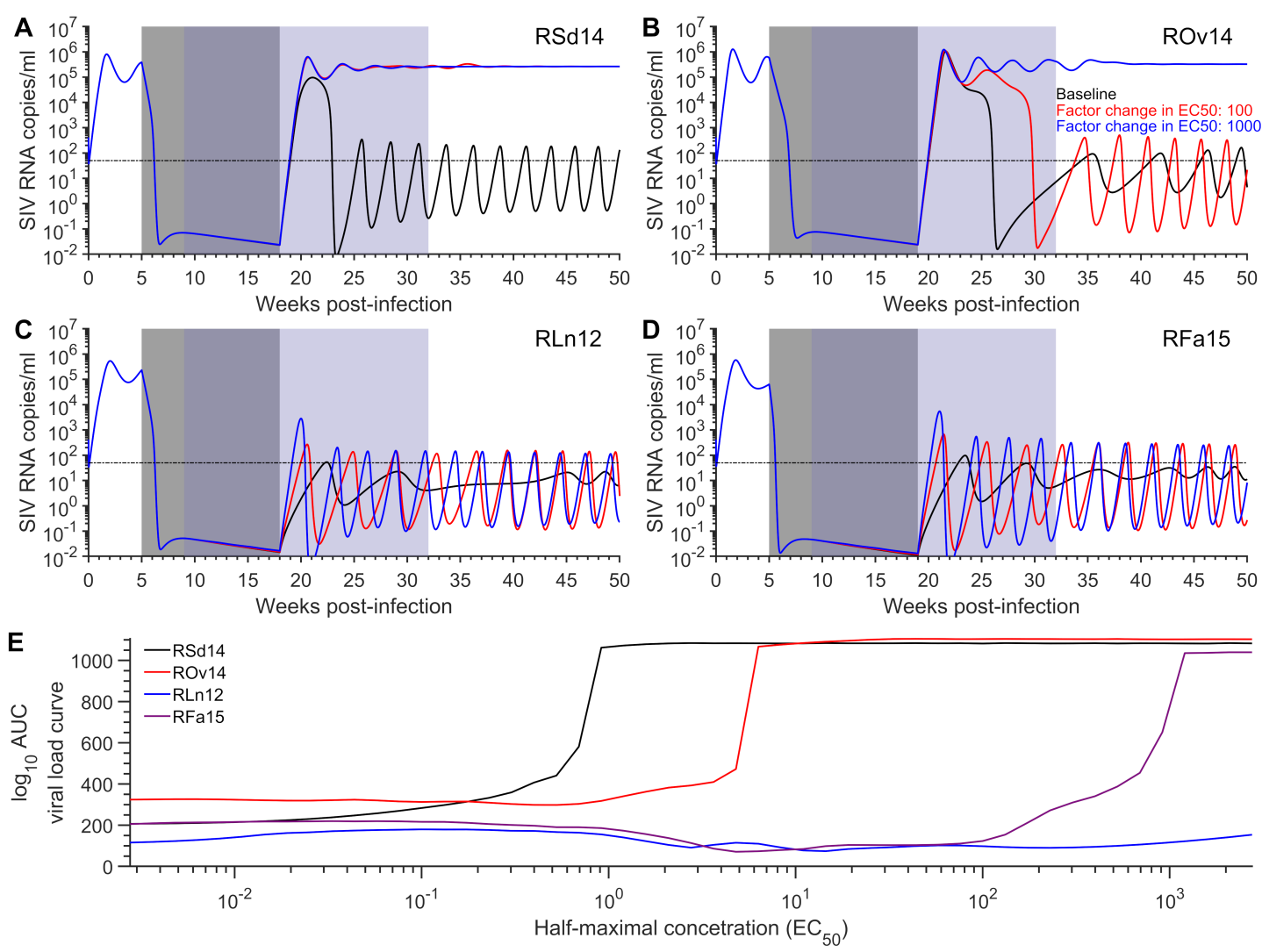

Supplement: S10 Fig — The model predicted viral load (solid line) using the best-fit parameter estimates and the baseline source model for the treated macaques A) RSd14, B) ROv14, C) RLn12, and D) RFa15 using the baseline half-maximal concentration, EC50, (black), 100-fold higher EC50 (red), and 1000-fold higher EC50 (blue). The limit of detection is 50 SIV RNA copies/ml (thin dashed black line, left panels). E) The area under the log10 predicted viral load curve for the 30 weeks after cART was stopped for the treated macaques RSd14 (black), ROv14 (red), RLn12 (blue), and RFa15 (purple). Treatment with cART occurred between five weeks and 18/19 weeks post-infection (gray area). Parameters for these simulations are in Tables 1 and S16. (PDF) [file pcbi.1009031.s032.pdf]

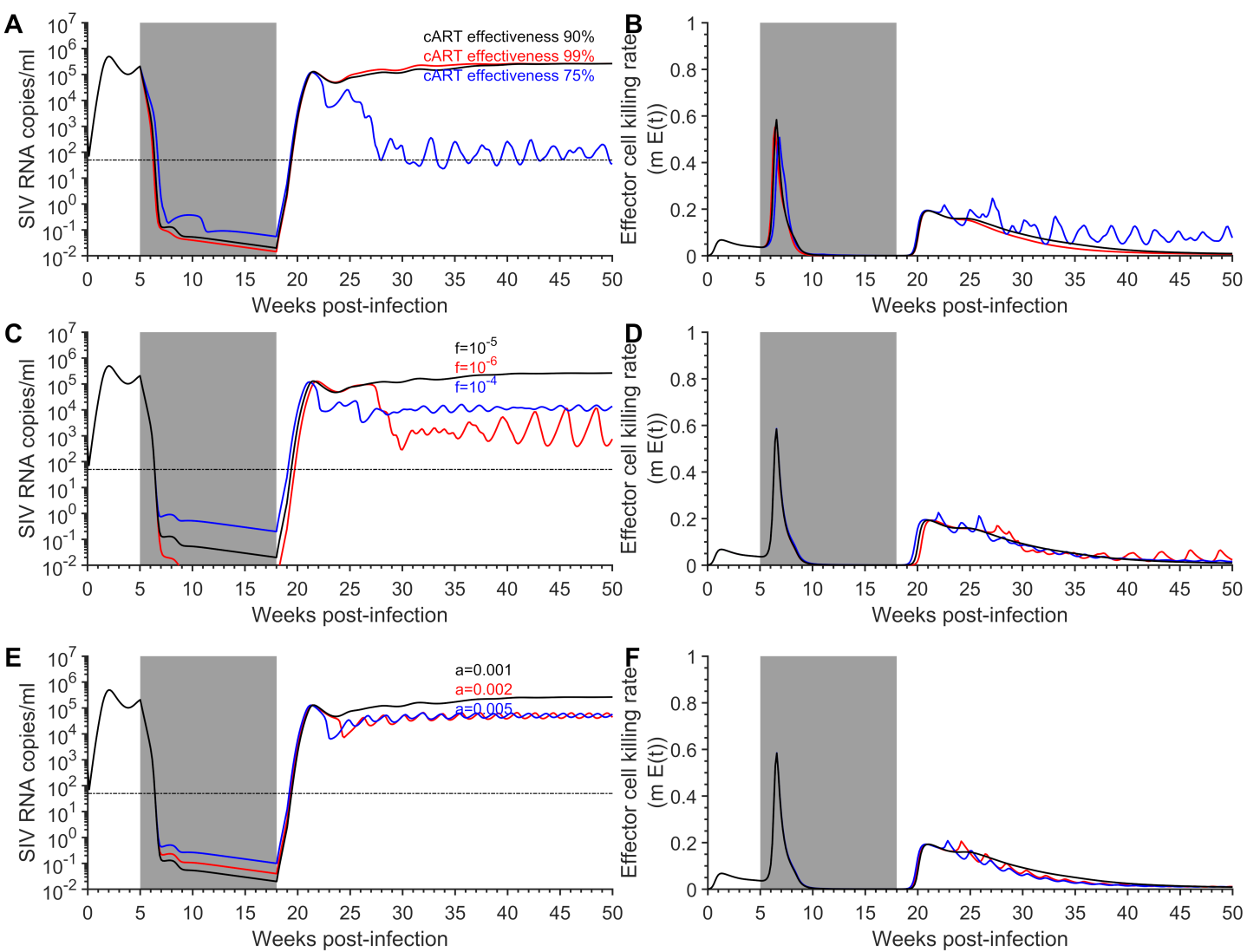

Supplement: S11 Fig — The average model predicted viral load (left panels) and the per day effector cell killing rate (right panels) using the best-fit parameter estimates under the baseline source model with the greatest AIC weight for each of the treated macaques (solid line). The sensitivity of the viral load and per day effector cell killing rate with respect to changing A)−B) the effectiveness of cART from 90% (black) to 99%(red) and 75% (blue); C)−D) the fraction of infections resulting in latency from 10−5 (black) to 10−6 (red) and 10−4 (blue); E)−F) the activation rate of latent cells from 10−3 (black) to 2 ×10^−3^ (red) and 5×10^−3^ (blue). The limit of detection is 50 SIV RNA copies/ml (thin dashed black line, left panels) and the minimum infected cell death rate is the death rate due to viral cytopathic effects (thin dashed line, right panels). Treatment with cART occurred between five weeks and 18/19 weeks post-infection (gray area). The average was calculated using the geometric mean for the seven IgG control macaques. Parameters for these simulations are in Tables 1 and S19. (PDF) [file pcbi.1009031.s033.pdf]

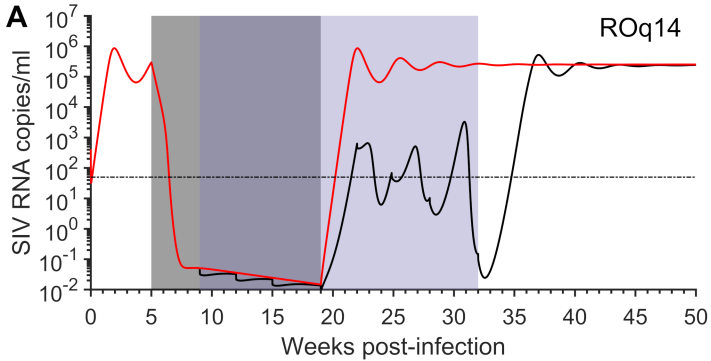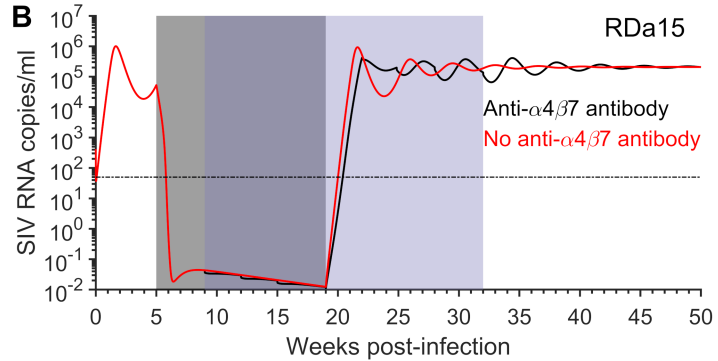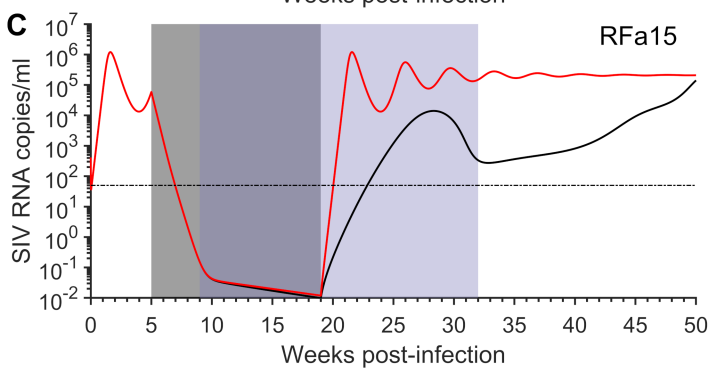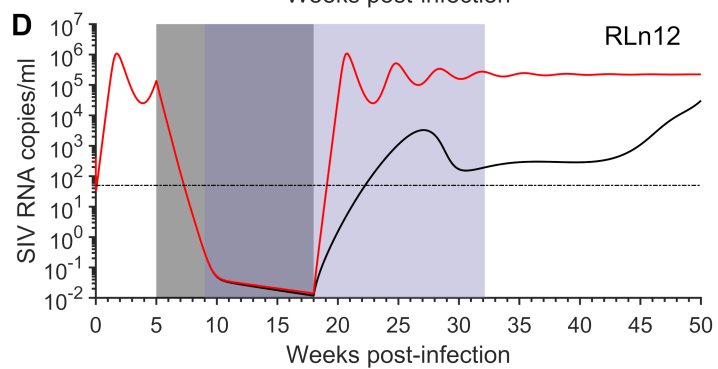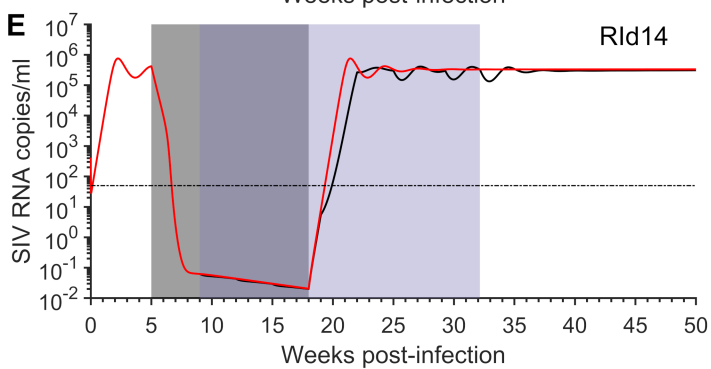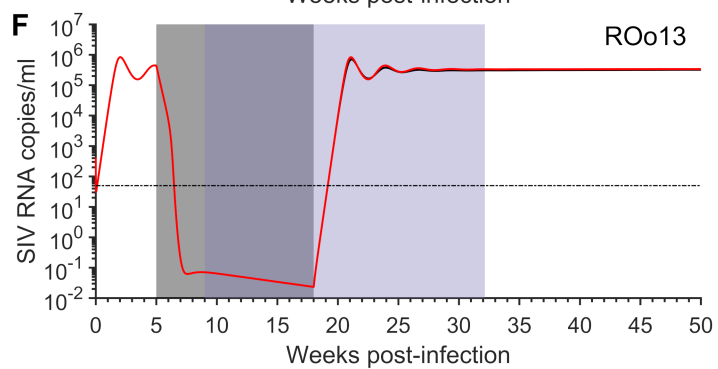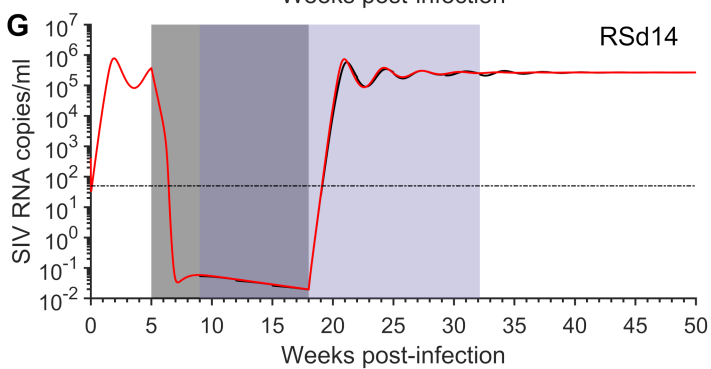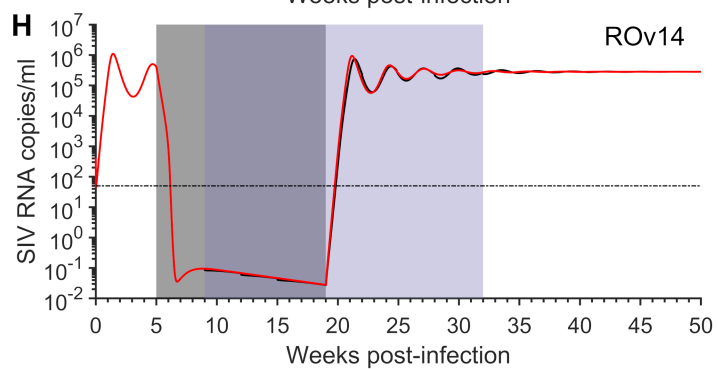

Supplement: S12 Fig — The model predicted viral loads for SIV nef-competent virus with anti-α4β7antibody therapy (black) and without anti-α4β7antibody therapy (red) using the best-fit parameter estimates and model variation with the greatest AIC weight for each of the treated macaques, panels A)–H). The limit of detection is 50 SIV RNA copies/ml (thin dashed black line). Treatment with cART occurred between five weeks and 18/19 weeks post-infection (gray area), while eight infusions of the anti-α4β7antibody occurred between nine weeks post-infection and 32 weeks post-infection (purple area). Parameters for these simulations are in Tables 1 and S14–S18, for the AIC selected model (Table 2). The effector cell killing rate (m) and the saturation constant for effector cell proliferation was adjusted for each treated macaque for the nef-competent virus (S13 Fig). (PDF) [file pcbi.1009031.s034.pdf]

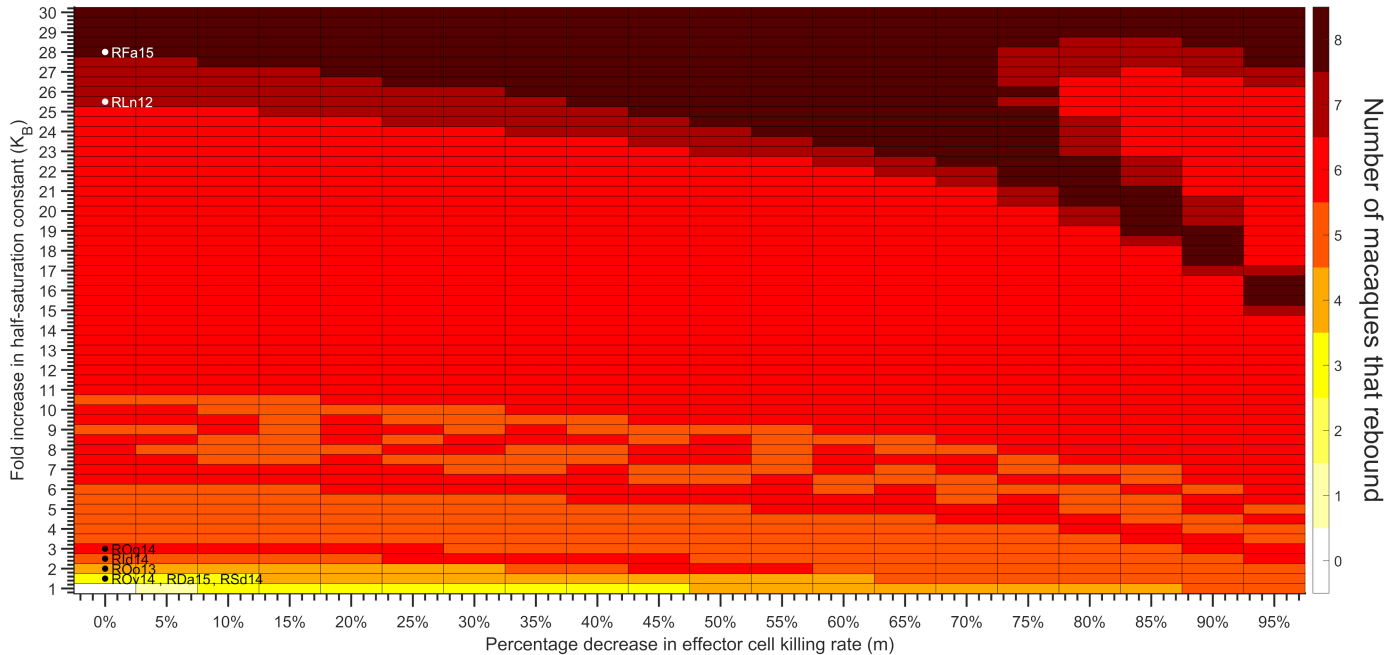

Supplement: S13 Fig — The proportion of macaques whose viral load rebounded after the removal of cART (color gradient) for various reductions in the effector cell killing rate (m) and increases in the half-saturation constant for effector cell proliferation (KB). Parameters for these simulations are in Tables 1 and S14–S18, for the AIC selected model (Table 2). Viral rebound was characterized by the model viral load not dropping below 50 RNA copies/ml any time after week 40 post-infection and had a viral load that exceeded 10,000 RNA copies/ml at week 81-post infection. (PDF) [file pcbi.1009031.s035.pdf]
